# Information quality, readability, and empathy of AI-generated public mental health information: a comparative evaluation of eight large language models

**DOI:** 10.3389/fpubh.2026.1859078

**Published:** 2026-06-26

**Authors:** Qilong Wang, Siheng Ma, Jian Sun, Xin Qi, Jianpeng Zhao, Dongmei Ma, Xiaofei Liu, Qiang An, Dongrong Zhao, Songkai He

**Affiliations:** 1 Xihua University, Chengdu, Sichuan, China; 2 Department of Psychiatry, Gansu Provincial People's Hospital, The First Clinical College of Gansu University of Chinese Medicine, Lanzhou, Gansu, China; 3 Department of Psychiatry, Xijing Hospital, Fourth Military Medical University, Xi'an, Shaanxi, China; 4 Xi‘an Medical University, Xi'an, Shaanxi, China; 5 Department of Biomedical Engineering, Fourth Military Medical University, Xi'an, Shaanxi, China

**Keywords:** empathy, information quality, large language models, patient education, psychiatry, readability

## Abstract

**Background:**

Mental disorders are a growing global health burden, yet healthcare resources remain scarce. Large language models (LLMs) may support public mental health information seeking, but their information quality, readability, and empathy in psychiatric contexts require validation.

**Method:**

We developed a test bank of 48 public mental health questions from literature, Google Trends (2004–2025), and clinical consultations. Eight LLM chatbots were compared for information quality, source transparency, readability, and empathy using established rating instruments, readability indices, and psychiatrist-rated and user-perspective empathy assessments. Statistical analysis used Kruskal–Wallis and Dunn's *post-hoc* tests, with Spearman correlations.

**Results:**

Gemini 3.0 Pro and GPT-5.2 Think showed relatively higher information quality and source transparency scores, whereas spontaneous source transparency was poor across models, with median JAMA scores of 0. No model met the sixth-grade readability standard; Claude Sonnet 4.5 generated relatively more readable responses, whereas Claude Sonnet 4.5 Think produced responses with the highest reading difficulty. In the psychiatrist-rated empathy assessment, Gemini 3.0 Pro and DeepSeek-V3 showed the highest high-empathy response rates, at 54.2% and 43.8%, respectively. User-perspective empathy ratings were generally lower, with DeepSeek-V3 and Gemini 3.0 Pro showing the highest user-perspective high-empathy response rates, at 31.2% and 27.1%, respectively. Information quality and source transparency metrics showed only weak correlations with readability metrics.

**Conclusion:**

LLMs face important challenges in psychiatric information delivery. Gemini 3.0 Pro and GPT-5.2 Think showed higher information quality and source transparency scores and better information structuring, whereas Claude Sonnet 4.5 generated relatively more readable responses. However, source opacity and high reading difficulty limit direct patient-facing use. Empathy varied across models and differed between psychiatrist-rated and user-perspective assessments, suggesting that empathic communication requires separate optimization and validation with intended users. Future work should balance information quality, source transparency, readability, traceability, safety, and emotionally appropriate responses.

## Graphical Abstract

**Figure:**
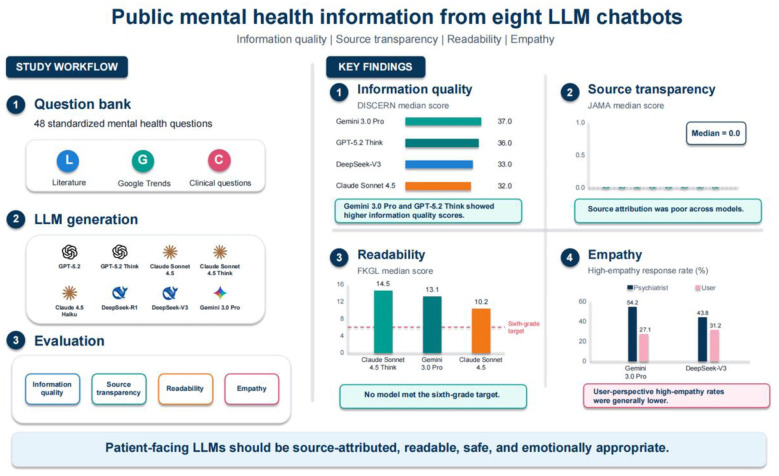

## Introduction

Mental disorders have been among the leading causes of disease globally over the past three decades (1). They remain a major contributor to the global burden of disease, accounting for approximately 14% of the total disease burden (2). According to the World Health Organization (WHO), approximately 970 million people worldwide are affected by mental disorders, with anxiety and mood disorders being the most prevalent (3). Compared to the healthy population, individuals with mental disorders often exhibit unhealthier lifestyle patterns, including higher smoking rates, more substance abuse, reduced physical activity, and poorer dietary habits (4). These factors result in individuals with mental disorders facing a mortality risk more than twice that of the healthy population, with significantly shorter life expectancy (5). The public health burden of mental disorders has been increasing in recent decades, alongside changes in global economic and social patterns (6, 7). However, global psychiatric health services are facing a critical resource shortage, with a substantial gap between supply and demand (8). In this context, LLMs may support preliminary information seeking and patient education, but they should not replace professional psychiatric assessment, treatment, or crisis intervention. The shortage of psychiatric professionals, long wait times, and stigma make it difficult for many patients to receive timely care (9). This has led more patients and families to seek help through the Internet and digital health platforms (10). Patients' health-seeking behavior is shifting from traditional “passive search” to AI-powered “intelligent interaction” (11). LLMs provide conversational interfaces that align with the communication needs of psychiatric practice. This, combined with patient demand for immediacy, privacy, and personalized support, creates potential opportunities for LLM application in psychiatry (12, 13).

LLMs have shown language-processing and response-generation capabilities that may be relevant to psychiatry, where communication, explanation, and emotional support are central to care. Existing models may have different design features and potential strengths: OpenAI ChatGPT has been widely used as a benchmark for medical question-answering (14); Anthropic Claude has been developed with an emphasis on safety-oriented responses (15); Google Gemini 3.0 Pro provides long-context processing capabilities that may support the integration of complex information (16); DeepSeek, an open-source model, has been described as having competitive reasoning and Chinese-language performance (17). However, these potential advantages do not necessarily indicate clinical information quality or patient-facing suitability. Model “hallucinations” may mislead patients (18), and information exceeding patients' health literacy may increase their anxiety (19). For public mental health information, LLM-generated responses should be high-quality, easy to understand, and emotionally appropriate. Information quality helps reduce the risk of misleading psychiatric advice, readability ensures that patients and families can understand the information, and empathy is important because mental health questions often involve distress, stigma, and safety concerns. Accordingly, information quality, source transparency, readability, and empathy represent complementary dimensions for evaluating LLM-generated mental health information. Although patients and families increasingly use LLMs for health information seeking, previous research has mainly focused on other medical specialties (20–22). In the sensitive context of psychiatry, systematic comparisons of model performance in information quality, source transparency, readability, and empathy remain limited.

Building on this rationale, we evaluated eight LLM chatbots, including GPT-5.2, GPT-5.2 Think, Claude Sonnet 4.5, Claude Sonnet 4.5 Think, Claude 4.5 Haiku, Gemini 3.0 Pro, DeepSeek-R1, and DeepSeek-V3. Using 48 standardized public mental health questions, we assessed information quality, source transparency, readability, and empathy to examine the suitability of LLM-generated responses for public mental health information seeking.

## Methodology

This study was reported in accordance with the CHART reporting guideline for studies evaluating generative AI-driven chatbots (23).

### Ethics

This study was conducted in accordance with the Declaration of Helsinki and was approved by the Ethics Committee of Gansu Provincial People's Hospital (Approval No. 2025-439). Clinical consultation questions were anonymized before analysis, and no identifiable patient information was retained. Informed consent was obtained from participants involved in the clinical question collection process. No copyrighted clinical records or proprietary patient materials were reproduced in this study.

### Research design

#### Question bank construction process

**Figure 1:**
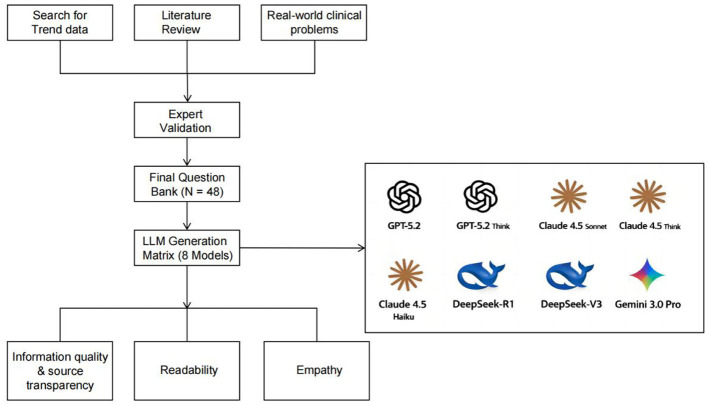
The overall experimental process of LLM chatbot evaluation.

We evaluated LLMs using common psychiatric inquiries and developed a 48-item question bank from three sources: published literature, Google Trends data, and real-world clinical questions (Figure 1). Published studies on psychiatric information needs were first reviewed to identify questions and themes commonly raised by patients and families (24–27). Google Trends was then used to retrieve high-frequency psychiatry-related search terms from 2004 to 2025. Broad seed terms, such as “mental illness” and “psychiatry,” were used to define the general search domain and were expanded to specific disorders and symptoms, such as “depression,” “schizophrenia,” “suicide,” and “hallucinations.” Related queries generated by Google Trends were reviewed and combined with common question words, including “what,” “how,” “when,” and “why,” to form questions consistent with everyday public information-seeking language. After removing duplicates and irrelevant entries, 20 questions derived from the literature review and Google Trends were retained as the public information component of the question bank.

#### Clinical question collection and processing

To supplement the public information questions, real-world clinical questions were collected during a structured 6-month question collection period at Gansu Provincial People's Hospital from June to November 2025. This was not an ethnographic observation or an audio-recorded interview study. Instead, clinical investigators prospectively summarized frequently asked, non-identifiable questions raised by patients or family members during routine outpatient consultations and inpatient ward rounds. No names, medical record numbers, dates of birth, or other identifiable information were recorded. The 50 most frequently observed clinical questions were compiled, and questions overlapping with the public information questions were removed. Finally, 28 clinical questions were retained to supplement the 20 public information questions. This produced a final 48-item question bank covering common public and clinical information needs in mental health care. The final number of 48 questions was determined pragmatically to ensure adequate coverage of key public mental health information domains, rather than based on a formal statistical sample size calculation.

#### Prompt standardization and structure

After the 20 public information questions and 28 clinical questions were combined, all 48 candidate prompts were reviewed by three psychiatrists for clinical relevance, redundancy, domain classification, and clarity. The final prompts were standardized to preserve the original patient and family information needs while ensuring identical wording across models. The prompt set included both open-ended and closed-ended questions, reflecting the natural variation of public mental health information needs. We did not convert all prompts into a single question format, because doing so could alter the original meaning of questions derived from public searches and clinical consultations. Each standardized question was submitted to all chatbots without additional prompt engineering, examples, follow-up questions, or instructions regarding response length or style. The complete prompt list is provided in Supplementary material S1.

#### Word cloud analysis of the question bank

**Figure 2:**
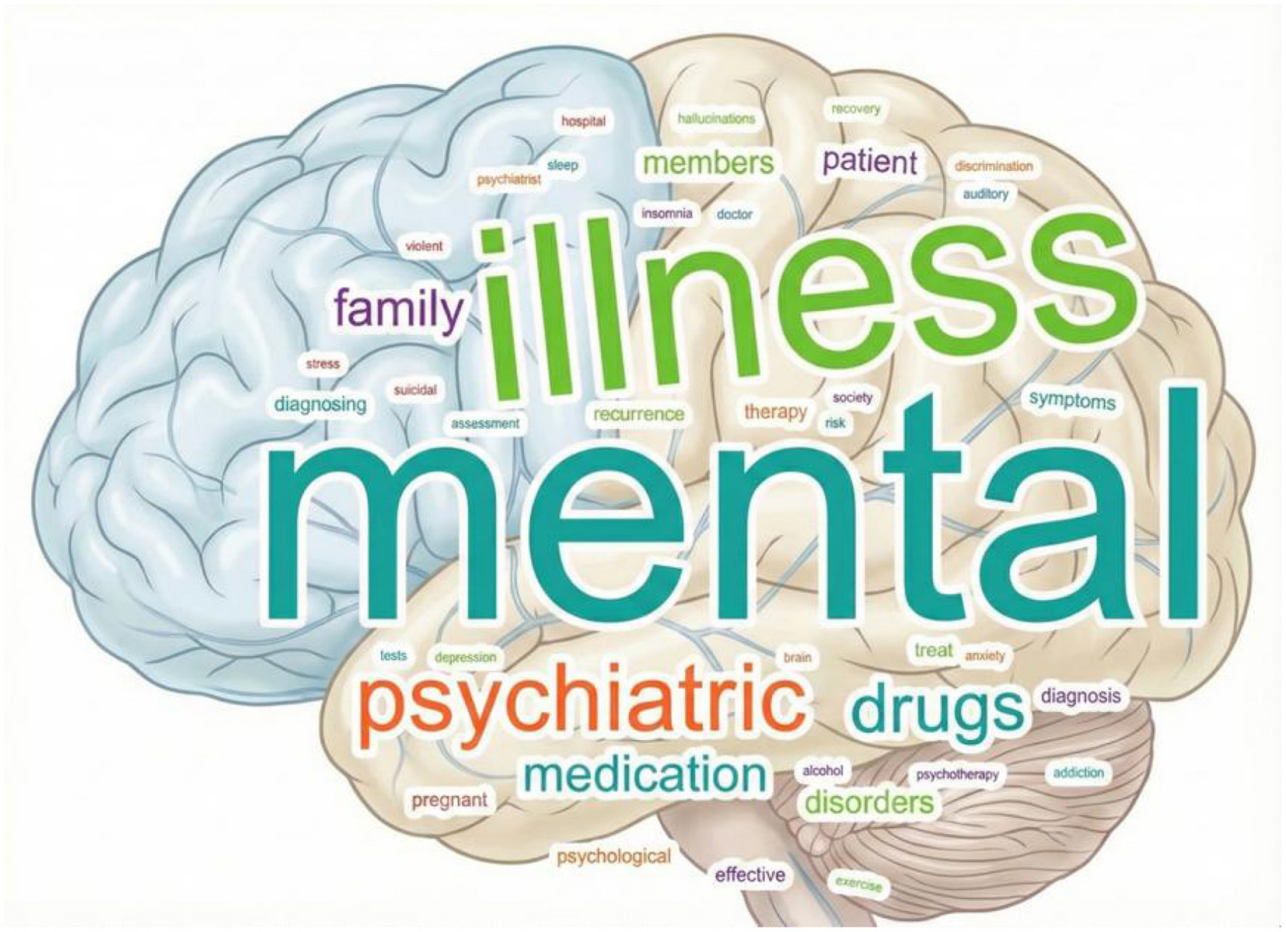
Word cloud of core keywords in the standardized mental health question bank.

To visualize the thematic distribution of the 48 standardized questions, a word cloud diagram was generated (Figure 2). After excluding non-informative terms, such as “how” and “what,” keyword font sizes were scaled by frequency. Prominent terms such as “mental illness,” “patient,” “treatment,” “medication,” “therapy,” and “family” reflect the main focus of the question bank, including clinical awareness, treatment-related concerns, and family-related issues. Medium-to-high frequency keywords, including “symptoms,” “diagnosis,” “recovery,” and “suicidal,” correspond to the six study-specific information domains. Additionally, terms such as “suicidal,” “violent,” and “discrimination” indicate coverage of high-risk behaviors and social stigma. The word cloud provides a visual summary of the thematic composition of the queries. To improve interpretability beyond the word cloud, the full list of 48 standardized questions and their domain classifications is provided in Supplementary Table S2.

#### Problem classification and knowledge framework

The 48 questions were organized into six study-specific domains of public mental health information needs: disease cognition (16.7%), clinical diagnosis (12.5%), treatment methods (20.8%), rehabilitation management (12.5%), social and family (27.1%), and special issues (10.4%). These domains were not intended to represent a formal psychiatric diagnostic taxonomy. Instead, they were developed to reflect the main information needs identified from the literature review, Google Trends data, and anonymized clinical consultation questions. The domain framework was informed by established psychiatric knowledge sources, including the Diagnostic and Statistical Manual of Mental Disorders, Fifth Edition, Text Revision (DSM-5-TR) (28), the National Institute for Health and Care Excellence (NICE) guidelines for adult depression (29), and the 2018 CANMAT/ISBD guidelines for bipolar disorder (30). By mapping the 48 inquiries against these knowledge sources, we aimed to ensure systematic coverage of the public mental health information spectrum, from symptom recognition and diagnostic understanding to treatment, rehabilitation, family support, and risk-related concerns. Reference answers were developed with reference to these materials and were used to support domain classification and evaluator judgment during performance assessment. The complete set of standardized study prompts, reference answers, and full chatbot outputs for all evaluated models is provided in Supplementary material S1.

#### Selection of LLMs and response generation protocol

This study evaluated eight mainstream LLM chatbots: GPT-5.2, GPT-5.2 Think, Claude Sonnet 4.5, Claude Sonnet 4.5 Think, Claude 4.5 Haiku, Gemini 3.0 Pro, DeepSeek-R1, and DeepSeek-V3. All models were accessed through their official web interfaces rather than third-party aggregators or API-based deployments. This study evaluated publicly deployed chatbot versions of the selected LLMs. No model was trained, fine-tuned, or modified by the study team. Because the study evaluated publicly accessible chatbot implementations rather than locally deployed base models, detailed model development parameters were not available to the investigators. Official model documentation or release pages were used to identify model versions where available. No novel base model, tuned model, or fine-tuned model was developed in this study; therefore, no training data, fine-tuning parameters, or deployment parameters are reported. Queries were conducted between 1 February 2026 and 10 February 2026 in Lanzhou, China. All prompts were submitted by a single trained investigator using a predefined, standardized question list. To ensure consistency across different query dates, the same prompt wording, model order, browser settings, and response collection procedure were used throughout the study period. Each query was copied directly from the standardized question bank without manual modification. To simulate ordinary public information-seeking behavior, each of the 48 standardized questions was submitted once to each model in a separate new chat session using identical wording. Memory and personalization were disabled when available, and web browsing, search functions, retrieval-augmented tools, and other external tools were not enabled. No regenerated or edited responses were included in the analysis. All responses were saved immediately after generation and checked by another investigator against the original prompt list to ensure completeness and consistency. Because sampling parameters such as temperature were not user-configurable in the web interfaces, the default platform settings were used. All prompts and responses were in English because the readability formulas applied in this study were developed for English-language text. No explicit instruction was given to provide references or source links; therefore, JAMA scores reflected spontaneous source transparency under patient-like questioning. Model identifiers, access platforms, source status, release or last-update information, and query details are summarized in Supplementary Table S1, with source status classified according to publicly available developer information.

#### Readability assessment methodology

Because all prompts and model responses were generated in English, readability was assessed using six widely recognized English-language readability indices through an online calculator (https://readabilityformulas.com): ARI, FRES, GFI, FKGL, CL, and SMOG.

Automated readability index (ARI) (31):


$$\begin{array}{l} \text{4}.\text{71}(\frac{\text{characters}}{\text{words}})\text{ + 0}.\text{5}(\frac{\text{words}}{\text{sentences}})\text{ - 21}.\text{43} \end{array}$$

Flesch Reading ease score (FRES) (32):


$$\begin{array}{l} \text{206}.\text{835 - 1}.\text{015 }\times \;(\frac{\text{words}}{\text{sentences}})\text{ - 84}.\text{6}(\frac{\text{syllables}}{\text{words}}) \end{array}$$

Gunning fog index (GFI) (33):


$$\begin{array}{l} \text{0}.\text{4}[(\frac{\text{words}}{\text{sentences}})\text{ + 100}(\frac{\text{complexwords}}{\text{words}})] \end{array}$$

Flesch–Kincaid grade level (FKGL) (34):


$$\begin{array}{l} \text{0}.\text{39}(\frac{\text{words}}{\text{sentences}})\text{ + 11}.\text{8}(\frac{\text{syllables}}{\text{words}})\text{ - 15}.\text{59} \end{array}$$

Coleman–Liau index (CL) (35):


$$\begin{array}{l} \text{CL }=\text{ 0}.\text{0588L - 0}.\text{296S - 15}.\text{8} \end{array}$$

where L is the average number of letters per 100 words and S is the average number of sentences per 100 words.

Simple measure of gobbledygook (SMOG) (36):


$$\begin{array}{l} \text{SMOG = 1}.\text{0430}\sqrt{\left[\text{P}\times (\text{30/N})\right]}\text{ + 3}.\text{1291} \end{array}$$

where P is the number of words with three or more syllables and N is the number of sentences.

The readability scores were compared against the sixth-grade level recommended by the American Medical Association (AMA) and the National Institutes of Health (NIH). For the FRES formula, an acceptable score was ≥80.0; for the other five grade-level formulas, acceptable scores were ≤ 6.

#### Assessment of information quality and source transparency

In this study, information quality and source transparency were operationalized as the credibility, completeness, balance, clarity, and attribution quality of LLM-generated mental health information, rather than the temporal stability or reproducibility of repeated model outputs. We used four validated instruments to assess these dimensions: DISCERN, EQIP, and GQS were used to evaluate information quality, whereas the JAMA Benchmark Criteria were used to assess source transparency and attribution.

Discussion of Information Sources in the Context of Evaluation of Reliability and Need (DISCERN): Assesses clarity, balance, and comprehensiveness of health information. The grading scheme follows existing research: 63–75 as “Excellent”, 51–62 as “Good”, 39–50 as “Average”, 27–38 as “Poor”, and 16–26 as “Very Poor” (37).

Ensuring Quality Information for Patients (EQIP): assesses patient information quality across multiple dimensions. It comprises 20 items rated as “Yes”, “Partially”, “No”, or “Not Applicable”, scored 1, 0.5, and 0 points respectively. The total score is calculated as: (sum of scores)/(number of applicable items) × 100. Scores are categorized as: 76%−100% as “High quality”, 51%−75% as “Good”, 26%−50% as “Fair”, and 0%−25% as “Poor” (38).

Global Quality Scale (GQS): assesses overall quality of health content using a 5-point scale (1 = very poor to 5 = excellent) (39).

Journal of the American Medical Association Benchmark Criteria (JAMA Benchmark Criteria): assesses four core principles: author identification, reference attribution, date clarification, and ownership declaration. Each criterion scores 0–1, with a total range of 0–4. Higher scores indicate greater source transparency and attribution quality (40).

#### Assessment of psychiatrist-rated and user-perspective empathy

We assessed empathy using a 5-point Likert scale adapted from Ayers et al. (41), who evaluated “the empathy or bedside manner provided” in responses to patient questions. Scores ranged from 1 to 5: 1 = “not empathetic”, 2 = “slightly empathetic”, 3 = “moderately empathetic”, 4 = “empathetic”, and 5 = “very empathetic”. Responses with scores ≥4 were classified as high-empathy responses, consistent with the threshold used by Ayers et al. (41). Before scoring, raters were instructed that empathy referred to whether the response would make a potential user feel understood, respected, supported, and non-judgmental. Raters were also instructed to focus on empathic communication rather than medical accuracy, readability, or source citation. Empathy was evaluated from two perspectives: psychiatrist-rated empathy and user-perspective empathy. For psychiatrist-rated empathy, two psychiatrists independently assessed all responses, and disagreements were resolved through discussion with a senior psychiatrist. For user-perspective empathy, two non-medical volunteers independently assessed perceived empathy from the viewpoint of potential users seeking mental health information, and disagreements were resolved through discussion with a third non-medical volunteer. All raters were blinded to chatbot identity. The two types of empathy scores were analyzed separately.

#### Assessment process and quality control

Two psychiatrists with more than 10 years of clinical experience independently evaluated all model responses using the predefined information quality, source transparency, and psychiatrist-rated empathy instruments. Evaluators were blinded to chatbot identity. Before consensus discussion, the overall initial agreement rate was 79.82%, and the mean ICC across manually rated assessments was 0.808, indicating good inter-rater consistency. Disagreements in psychiatrist-rated assessments were resolved through discussion with a senior psychiatrist. User-perspective empathy was independently evaluated by two non-medical volunteers from the viewpoint of potential users seeking mental health information, and disagreements were resolved through discussion with a third non-medical volunteer. Readability indices were calculated using the same online readability calculator for all responses.

#### Statistical analysis

Information quality, source transparency, readability, and empathy scores were summarized as mean ± SD and median (IQR). Normality was assessed using the Shapiro–Wilk test. Because most variables were non-normally distributed and some outcomes were ordinal or bounded scale scores, between-model differences were assessed using the Kruskal–Wallis test. When the omnibus test was significant, Dunn's *post-hoc* test with Benjamini–Hochberg false discovery rate correction was used for exploratory pairwise comparisons. Spearman's rank correlation was used to assess associations between information quality and source transparency metrics and readability metrics. Two-sided adjusted *P* < 0.05 was considered statistically significant. Because each standardized prompt was submitted once to each model, response reproducibility across repeated generations was not assessed. Statistical analyses were performed using R 4.4.2.

## Results

### Overall assessment of information quality and source transparency

**Figure 3:**
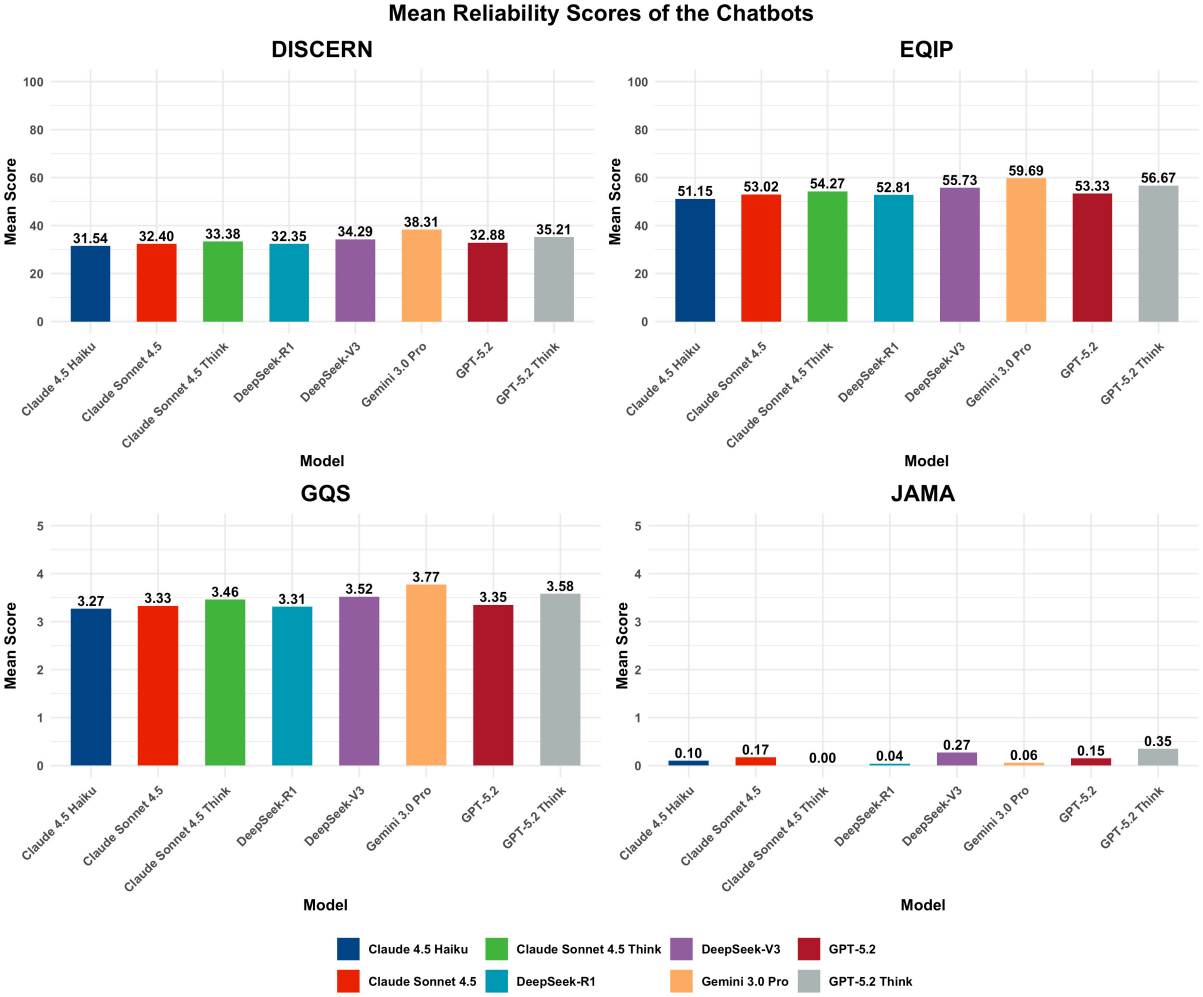
Average information quality and source transparency scores for LLMs based on DISCERN, EQIP, GQS, and JAMA.

As shown in Table 1 and Figure 3, we used DISCERN, EQIP, JAMA, and GQS to evaluate information quality and source transparency from eight LLMs. These evaluations were conducted with reference to the predefined reference answers and established psychiatric knowledge frameworks described in the Methods section. Results showed significant differences across all four metrics (all *P* < 0.001). Gemini 3.0 Pro had the highest DISCERN, EQIP, and GQS scores, with medians of 37.0, 60.0, and 4.0, respectively. Claude 4.5 Haiku had the lowest DISCERN and EQIP scores, with medians of 30.0 and 50.0. Under JAMA, all models showed zero transparency (median = 0.0). GPT-5.2 Think showed higher information quality and source transparency scores than GPT-5.2, whereas the difference between Claude Sonnet 4.5 Think and Claude Sonnet 4.5 was less consistent.

**Table 1:** Information quality and source transparency scores across AI chatbots.

Program	DISCERN, median (IQR)	EQIP, median (IQR)	GQS, median (IQR)	JAMA, median (IQR)
Claude 4.5 Haiku	30.0 (28.0, 35.3)	50.0 (48.8, 55.0)	3.0 (3.0, 4.0)	0.0 (0.0, 0.0)
Claude Sonnet 4.5	32.0 (31.0, 33.0)	55.0 (50.0, 55.0)	3.0 (3.0, 4.0)	0.0 (0.0, 0.0)
Claude Sonnet 4.5 Think	32.5 (30.0, 37.0)	55.0 (50.0, 55.0)	3.0 (3.0, 4.0)	0.0 (0.0, 0.0)
DeepSeek-R1	31.0 (30.0, 33.0)	50.0 (50.0, 55.0)	3.0 (3.0, 4.0)	0.0 (0.0, 0.0)
DeepSeek-V3	33.0 (32.0, 35.5)	55.0 (50.0, 60.0)	4.0 (3.0, 4.0)	0.0 (0.0, 1.0)
Gemini 3.0 Pro	37.0 (32.0, 45.0)	60.0 (58.8, 65.0)	4.0 (4.0, 4.0)	0.0 (0.0, 0.0)
GPT-5.2	32.0 (31.0, 33.0)	50.0 (50.0, 55.0)	3.0 (3.0, 4.0)	0.0 (0.0, 0.0)
GPT-5.2 Think	36.0 (33.0, 37.0)	55.0 (53.8, 60.0)	4.0 (3.0, 4.0)	0.0 (0.0, 1.0)
H	60.4	63.3	38.6	39.1
*P*	<0.001	<0.001	<0.001	<0.001

IQR, interquartile range.

### Comparison of information quality and transparency differences between models

Table 2 shows exploratory Dunn's *post-hoc* pairwise comparisons following significant Kruskal–Wallis tests, identifying model pairs that contributed to the overall between-model differences across four information quality and source transparency metrics. Key findings were as follows: Gemini 3.0 Pro and GPT-5.2 Think scored significantly higher than Claude 4.5 Haiku, Claude Sonnet 4.5, and DeepSeek-R1 on DISCERN, EQIP, and GQS (all *P* < 0.05). In the GPT series, GPT-5.2 Think scored higher than GPT-5.2 on DISCERN, EQIP, and JAMA (all *P* < 0.05). However, Claude Sonnet 4.5 Think showed no significant differences from Claude Sonnet 4.5 (all *P* > 0.05). Claude Sonnet 4.5 Think scored higher than Claude 4.5 Haiku on DISCERN and EQIP (both *P* < 0.05), while no significant difference was found between Claude Sonnet 4.5 and Claude 4.5 Haiku. In the DeepSeek series, DeepSeek-V3 scored higher than DeepSeek-R1 on DISCERN and JAMA (all *P* < 0.01).

**Table 2:** Exploratory Dunn *post-hoc* pairwise comparisons for information quality and source transparency scores.

Comparison	DISCERN	EQIP	JAMA	GQS
Claude 4.5 Haiku vs. Claude Sonnet 4.5	0.1597	0.1404	0.4659	0.6286
Claude 4.5 Haiku vs. Claude Sonnet 4.5 Think	0.0478	0.0123	0.2267	0.1217
Claude Sonnet 4.5 vs. Claude Sonnet 4.5 Think	0.5990	0.3480	0.0507	0.3227
Claude 4.5 Haiku vs. DeepSeek-R1	0.4056	0.2466	0.4871	0.7345
Claude Sonnet 4.5 vs. DeepSeek-R1	0.5864	0.7311	0.1617	0.8377
Claude Sonnet 4.5 Think vs. DeepSeek-R1	0.2621	0.2008	0.6037	0.2496
Claude 4.5 Haiku vs. DeepSeek-V3	0.0003	0.0041	0.0558	0.0391
Claude Sonnet 4.5 vs. DeepSeek-V3	0.0326	0.2022	0.24	0.1304
Claude Sonnet 4.5 Think vs. DeepSeek-V3	0.1247	0.7267	0.0011	0.6560
DeepSeek-R1 vs. DeepSeek-V3	0.005	0.1124	0.0064	0.0945
Claude 4.5 Haiku vs. Gemini 3.0 Pro	<0.0001	<0.0001	0.6279	<0.0001
Claude Sonnet 4.5 vs. Gemini 3.0 Pro	0.0002	<0.0001	0.255	0.0002
Claude Sonnet 4.5 Think vs. Gemini 3.0 Pro	0.0013	0.0003	0.5103	0.0099
DeepSeek-R1 vs. Gemini 3.0 Pro	<0.0001	<0.0001	0.7711	0.0001
DeepSeek-V3 vs. Gemini 3.0 Pro	0.1244	0.0015	0.0127	0.0434
Claude 4.5 Haiku vs. GPT-5.2	0.058	0.1761	0.6540	0.5500
Claude Sonnet 4.5 vs. GPT-5.2	0.6268	0.8908	0.7996	0.8687
Claude Sonnet 4.5 Think vs. GPT-5.2	0.9179	0.2910	0.0972	0.428
DeepSeek-R1 vs. GPT-5.2	0.2988	0.7825	0.272	0.7638
DeepSeek-V3 vs. GPT-5.2	0.1054	0.1716	0.1741	0.1772
Gemini 3.0 Pro vs. GPT-5.2	0.0010	<0.0001	0.3423	0.0003
Claude 4.5 Haiku vs. GPT-5.2 Think	<0.0001	<0.0001	0.0027	0.0119
Claude Sonnet 4.5 vs. GPT-5.2 Think	0.0007	0.0125	0.0275	0.0488
Claude Sonnet 4.5 Think vs. GPT-5.2 Think	0.0052	0.1427	<0.0001	0.3407
DeepSeek-R1 vs. GPT-5.2 Think	0.0001	0.0039	0.0002	0.0310
DeepSeek-V3 vs. GPT-5.2 Think	0.2681	0.2351	0.3603	0.6858
Gemini 3.0 Pro vs. GPT-5.2 Think	0.6809	0.0583	0.0004	0.1405
GPT-5.2 vs. GPT-5.2 Think	0.0040	0.0089	0.0145	0.0617

Responses with lower information quality and source transparency scores generally deviated from the predefined reference answers in several recurring ways. These deviations most commonly involved incomplete coverage of key psychiatric information, limited discussion of uncertainty or indications for professional help, insufficient explanation of treatment or rehabilitation options, and lack of source attribution. In this study, such deviations were reflected primarily in lower DISCERN, EQIP, and JAMA scores rather than in a separate item-level accuracy classification. Potentially harmful or misleading response patterns were reviewed qualitatively during expert assessment, but no dedicated harm-classification instrument was used.

### Domain-specific analysis of information quality and source transparency

**Figure 4:**
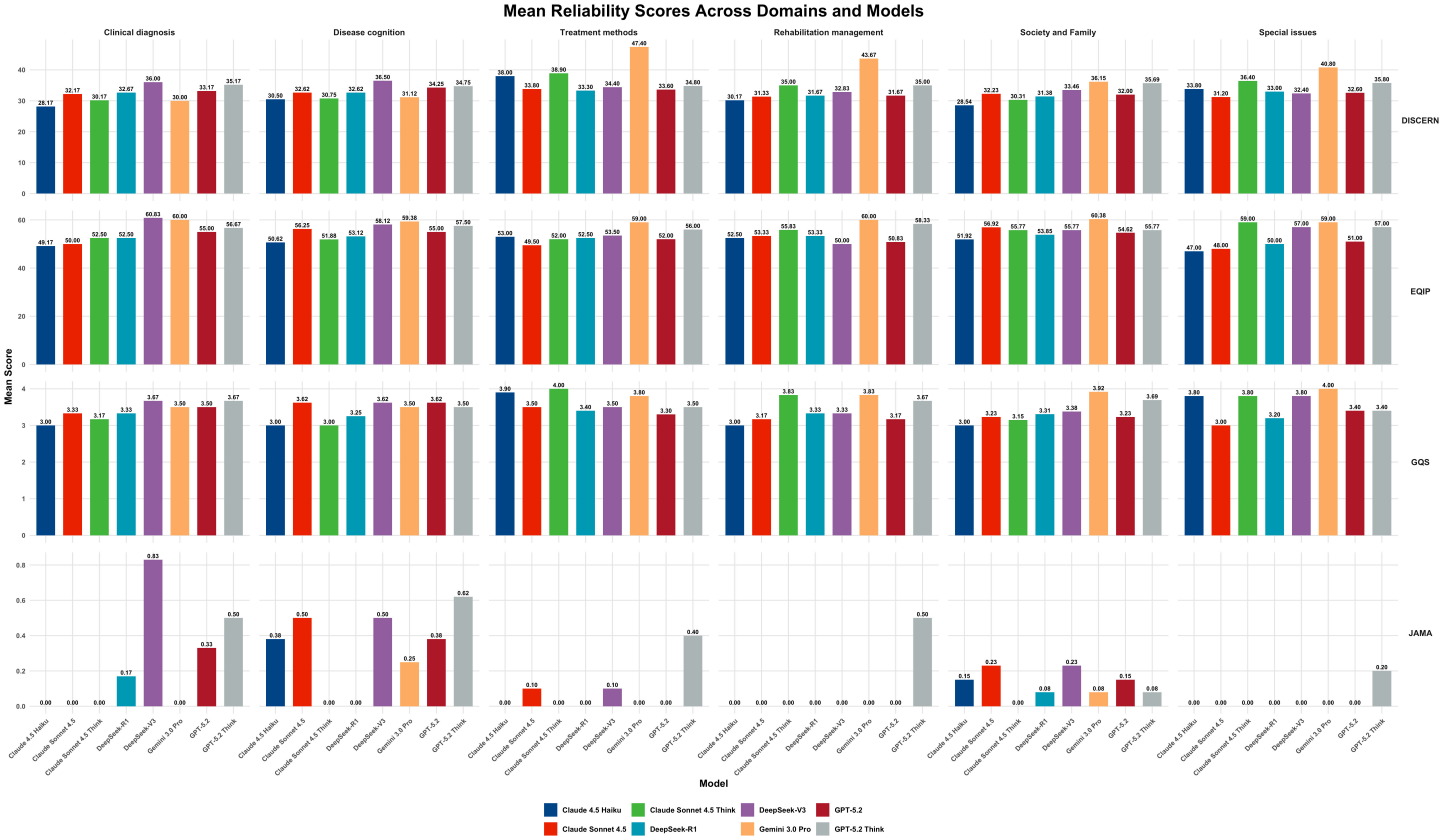
Average information quality and source transparency scores for LLMs across DISCERN, EQIP, GQS, and JAMA in different mental health information domains.

Figure 4 shows mean DISCERN, EQIP, GQS, and JAMA scores for each LLM across six clinical domains: clinical diagnosis, disease cognition, rehabilitation management, social and family aspects, special issues, and treatment methods. Overall, models showed modest information quality performance across DISCERN, EQIP, and GQS, while JAMA scores remained critically low across all categories, indicating poor spontaneous source transparency. Using the highest-scoring model as a benchmark, mean DISCERN scores were higher in “Rehabilitation Management” and “Treatment Methods” (43.67 and 47.4 points) and lower in “Clinical Diagnosis” and “Disease Cognition” (36.0 and 36.5 points). Mean GQS scores followed a similar pattern, with Gemini 3.0 Pro scoring high in rehabilitation and social support (3.83 and 3.92), while DeepSeek-V3 performed better in diagnosis and cognition (3.67 and 3.62). Mean EQIP scores were relatively stable (58.12–60.83 points). Mean JAMA scores showed minor peaks for DeepSeek-V3 in diagnosis (0.83) and GPT-5.2 Think in cognition (0.62), with most other domains near zero.

### Overall assessment of model readability

**Figure 5:**
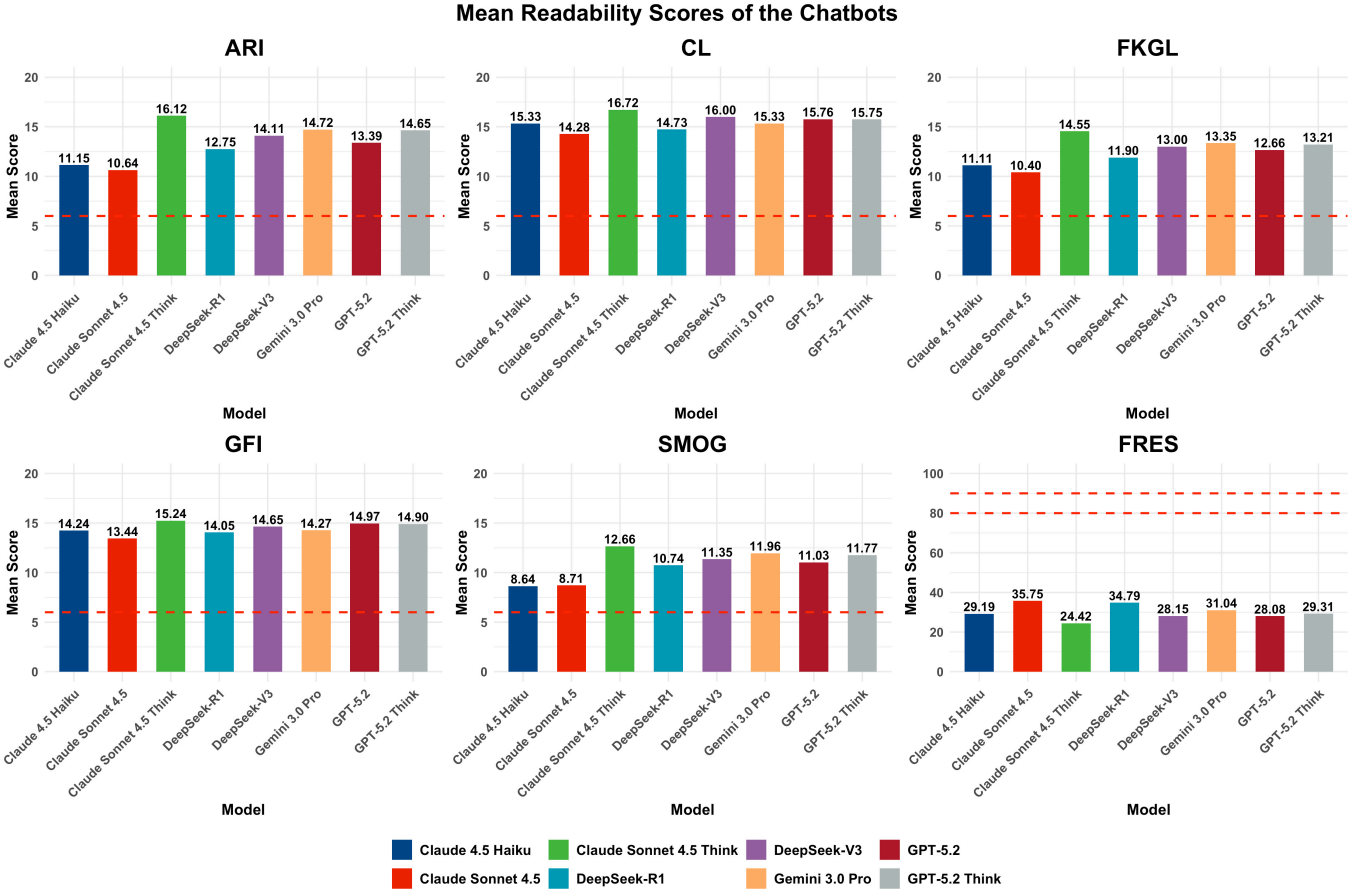
Mean readability scores of LLM-generated responses based on ARI, GFI, FKGL, CL, SMOG, and FRES indices. Reference lines indicate the recommended readability thresholds for patient education materials: grade level ≤ 6 for ARI, GFI, FKGL, CL, and SMOG, and FRES ≥80 for reading ease. ARI, automated readability index; GFI, gunning fog index; FKGL, Flesch–Kincaid grade level; CL, Coleman–Liau index; SMOG, simple measure of gobbledygook; FRES, Flesch reading ease score.

As shown in Table 3 and Figure 5, readability was evaluated using six indicators: ARI, FRES, GFI, FKGL, CL, and SMOG. No LLM met the sixth-grade readability standard. Median FRES scores were well below the recommended range of 80–90. Median values for ARI, GFI, FKGL, CL, and SMOG indicated text complexity exceeding standards for general readers (all median > 6). Specifically, Claude Sonnet 4.5 had the lowest scores on ARI (10.2), CL (13.9), FKGL (10.2), SMOG (8.7), and GFI (13.1), while achieving the highest FRES score (38.0), indicating relatively lower text complexity. In contrast, Claude Sonnet 4.5 Think had the highest scores on five metrics: ARI (15.8), CL (16.7), FKGL (14.5), GFI (15.5), and SMOG (12.2), but the lowest FRES score (24.5). Results show that the reasoning-enhanced Claude Sonnet 4.5 Think generates the most complex text, creating the greatest barrier to comprehension.

**Table 3:** Readability scores across AI chatbots.

Program	ARI, median (IQR)	CL, median (IQR)	FKGL, median (IQR)	FRES, median (IQR)	GFI, median (IQR)	SMOG, median (IQR)
Claude 4.5 Haiku	10.7 (9.9, 12.2)	15.0 (13.8, 16.6)	10.9 (9.9, 12.4)	30.0 (22.5, 37.2)	14.1 (12.8, 15.9)	8.7 (7.8, 9.2)
Claude Sonnet 4.5	10.2 (9.4, 11.9)	13.9 (12.8 ,15.8)	10.2 (9.1, 11.8)	38.0 (23.8, 45.3)	13.1 (11.7, 16.0)	8.7 (8.1, 9.3)
Claude Sonnet 4.5 Think	15.8 (14.4, 17.3)	16.7 (15.0, 17.7)	14.5 (13.1, 15.8)	24.5 (14.8, 34.5)	15.5 (13.5, 16.4)	12.2 (11.2, 13.7)
DeepSeek-R1	12.8 (11.5, 14.2)	14.6 (13.2, 16.2)	11.7 (10.5, 13.5)	36.5 (24.0, 42.5)	13.8(12.1, 15.9)	10.8 (9.9, 11.6)
DeepSeek-V3	13.3 (11.9, 16.3)	15.6 (14.0, 17.8)	12.5 (11.2, 14.7)	30.5 (17.0, 39.0)	14.6 (13.2, 16.3)	11.0 (10.1, 12.5)
GPT-5.2	13.6 (12.1, 14.7)	15.6 (14.1, 17.5)	12.9 (11.1, 13.9)	29.0 (17.8, 39.0)	14.9 (13.3, 16.5)	11.3 (9.9, 11.9)
GPT-5.2 Think	14.8 (12.6, 16.2)	15.3 (14.0, 17.5)	13.1 (11.5, 14.8)	31.5 (20.8, 40.0)	14.8 (13.4, 16.5)	11.7 (10.4, 12.8)
Gemini 3.0 Pro	14.5 (13.2, 16.3)	15.1 (13.7, 17.1)	13.1 (12.1, 15.0)	32.5 (21.0, 40.0)	14.4 (13.0, 15.8)	11.7 (10.8, 13.2)
6th grade level score	6	6	6	80–90	6	6

IQR, interquartile range.

### Comparison of readability differences across models

Table 4 shows exploratory Dunn's *post-hoc* pairwise comparisons following significant Kruskal–Wallis tests, identifying model pairs that contributed to the overall between-model differences across six readability metrics: ARI, FRES, GFI, FKGL, CL, and SMOG. Claude Sonnet 4.5 showed significantly more favorable readability scores than Claude Sonnet 4.5 Think across all six metrics (all *P* < 0.05), with lower grade-level scores and a higher FRES score. Claude 4.5 Haiku and DeepSeek-R1 also showed lower readability-difficulty scores than Claude Sonnet 4.5 Think on ARI, FKGL, CL, and SMOG (all *P* < 0.05). In addition, Claude Sonnet 4.5 showed lower scores on these four grade-level metrics than DeepSeek-V3, GPT-5.2, and GPT-5.2 Think (all *P* < 0.05), indicating relatively easier readability.

**Table 4:** Exploratory Dunn *post-hoc* pairwise comparisons for readability scores.

Comparison	ARI	FRES	GFI	FKGL	CL	SMOG
Claude 4.5 Haiku vs. Claude Sonnet 4.5	0.4931	0.0966	0.3330	0.2865	0.0981	0.8361
Claude 4.5 Haiku vs. Claude Sonnet 4.5 Think	< 0.0001	0.1860	0.1899	< 0.0001	0.0250	< 0.0001
Claude Sonnet 4.5 vs. Claude Sonnet 4.5 Think	< 0.0001	0.0016	0.0105	< 0.0001	< 0.0001	< 0.0001
Claude 4.5 Haiku vs. DeepSeek-R1	0.0043	0.1249	0.6989	0.0959	0.3260	< 0.0001
Claude Sonnet 4.5 vs. DeepSeek-R1	0.0004	0.8872	0.4781	0.0049	0.4939	< 0.0001
Claude Sonnet 4.5 Think vs. DeepSeek-R1	< 0.0001	0.0028	0.0783	< 0.0001	0.0009	< 0.0001
Claude 4.5 Haiku vs. DeepSeek-V3	< 0.0001	0.8741	0.6053	0.0002	0.3237	< 0.0001
Claude Sonnet 4.5 vs. DeepSeek-V3	< 0.0001	0.0751	0.1099	< 0.0001	0.0054	< 0.0001
Claude Sonnet 4.5 Think vs. DeepSeek-V3	0.0012	0.2608	0.3867	0.0042	0.2786	0.0056
DeepSeek-R1 vs. DeepSeek-V3	0.0295	0.0863	0.4076	0.0536	0.0381	0.2257
Claude 4.5 Haiku vs. Gemini 3.0 Pro	< 0.0001	0.7452	0.8534	< 0.0001	0.9644	< 0.0001
Claude Sonnet 4.5 vs. Gemini 3.0 Pro	< 0.0001	0.1825	0.2518	< 0.0001	0.0897	< 0.0001
Claude Sonnet 4.5 Think vs. Gemini 3.0 Pro	0.0557	0.1081	0.2192	0.0577	0.0255	0.2750
DeepSeek-R1 vs. Gemini 3.0 Pro	0.0004	0.2741	0.6662	0.0038	0.3334	0.0038
DeepSeek-V3 vs. Gemini 3.0 Pro	0.2052	0.5722	0.6375	0.3635	0.3177	0.1157
Claude 4.5 Haiku vs. GPT-5.2	< 0.0001	0.854	0.3138	0.0012	0.4806	< 0.0001
Claude Sonnet 4.5 vs. GPT-5.2	< 0.0001	0.0832	0.0369	< 0.0001	0.0170	< 0.0001
Claude Sonnet 4.5 Think vs. GPT-5.2	< 0.0001	0.2760	0.6794	0.0011	0.1282	0.0012
DeepSeek-R1 vs. GPT-5.2	0.2260	0.0834	0.1878	0.1294	0.0913	0.4316
DeepSeek-V3 vs. GPT-5.2	0.3285	0.9490	0.6367	0.6490	0.7349	0.6539
Gemini 3.0 Pro vs. GPT-5.2	0.0253	0.5508	0.3792	0.1812	0.4956	0.0415
Claude 4.5 Haiku vs. GPT-5.2 Think	< 0.0001	0.9078	0.3674	< 0.0001	0.5321	< 0.0001
Claude Sonnet 4.5 vs. GPT-5.2 Think	< 0.0001	0.0966	0.0475	< 0.0001	0.0212	< 0.0001
Claude Sonnet 4.5 Think vs. GPT-5.2 Think	0.0322	0.1559	0.6296	0.0183	0.1007	0.1109
DeepSeek-R1 vs. GPT-5.2 Think	0.0011	0.1495	0.2331	0.0141	0.1062	0.0195
DeepSeek-V3 vs. GPT-5.2 Think	0.2945	0.8434	0.6889	0.6381	0.6551	0.2834
Gemini 3.0 Pro vs. GPT-5.2 Think	0.7968	0.8533	0.4427	0.6570	0.5230	0.6140
GPT-5.2 vs. GPT-5.2 Think	0.0426	0.8225	0.8678	0.3607	0.9240	0.1323

### Domain-specific analysis of readability

**Figure 6:**
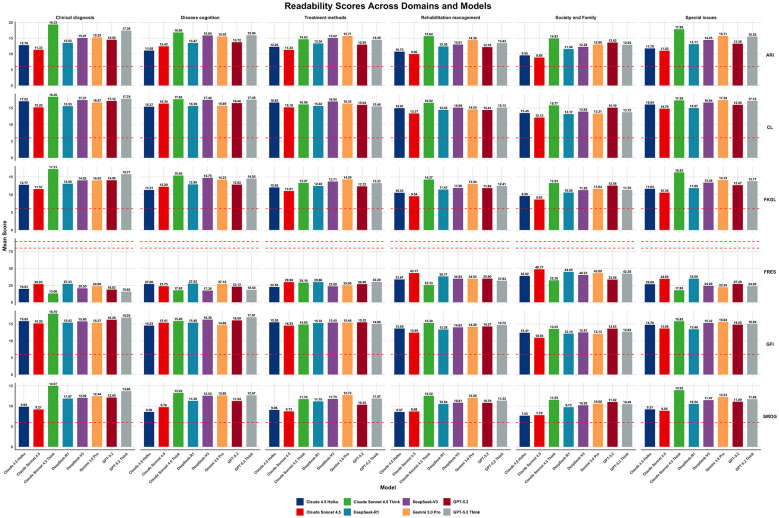
Mean readability scores of LLM-generated responses across mental health knowledge domains based on ARI, FRES, GFI, FKGL, CL, and SMOG indices.

Figure 6 shows mean readability scores across six clinical domains: clinical diagnosis, disease cognition, rehabilitation management, social and family aspects, special issues, and treatment methods. Results: in five domains—clinical diagnosis, rehabilitation management, social and family aspects, special issues, and treatment methods—Claude Sonnet 4.5 showed the best readability, with advantages across all six metrics (ARI, CL, FKGL, FRES, GFI, and SMOG). In the disease cognition domain, Claude 4.5 Haiku had the most readable output. Its mean scores were: ARI 11.08, CL 15.37, FKGL 11.31, FRES 27.0, GFI 14.53, and SMOG 8.56.

### Assessment of psychiatrist-rated and user-perspective empathy in LLM-generated psychiatric advice

**Figure 7:**
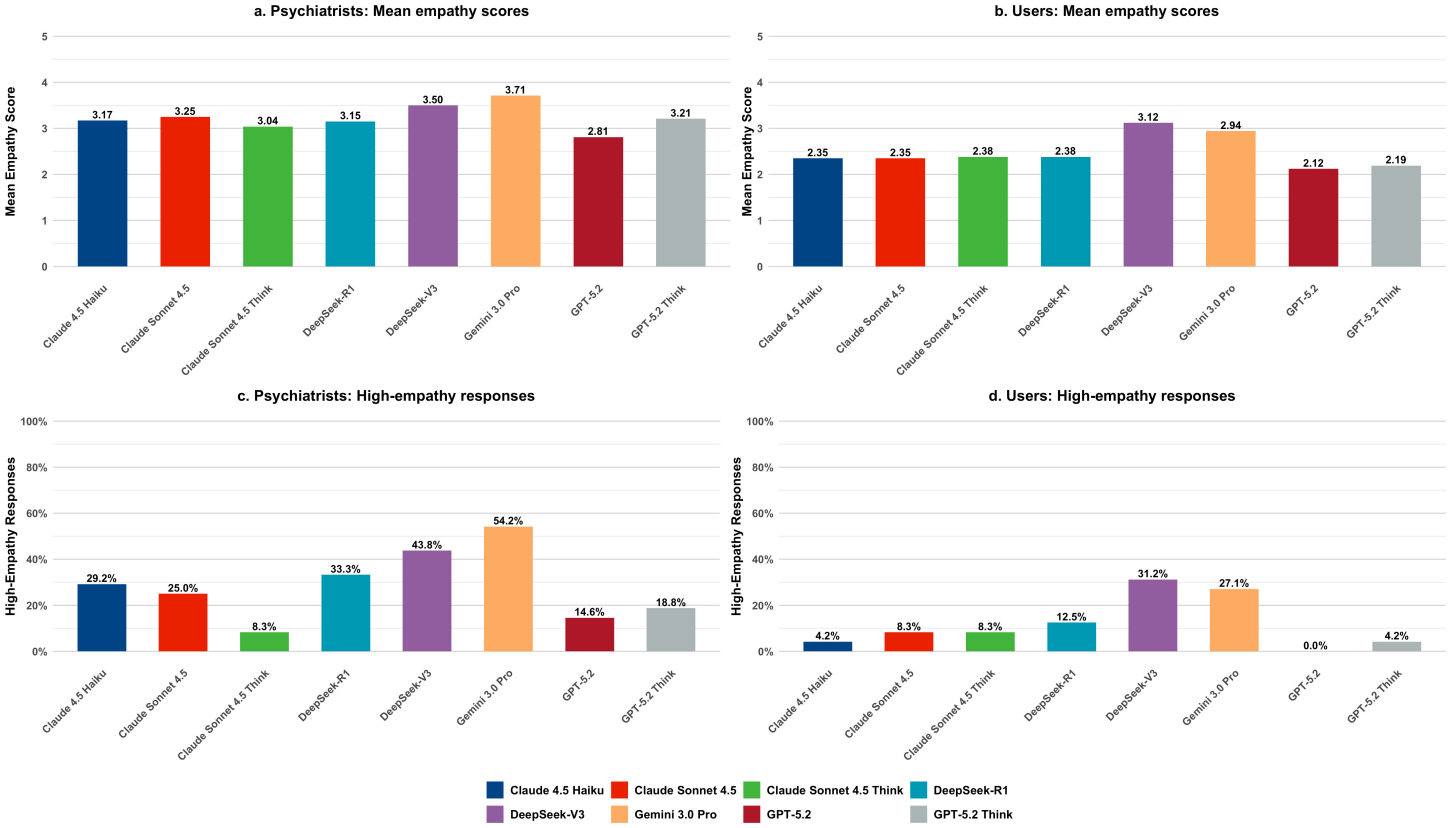
Psychiatrist-rated and user-perspective empathy assessment of LLM responses. **(a, b)** Show mean empathy scores rated by psychiatrists and user-perspective raters, respectively. **(c, d)** Show the corresponding proportions of high-empathy responses. High-empathy responses were defined as responses with empathy scores ≥4 on the 1–5 Likert scale. User-perspective raters refer to non-medical volunteers who evaluated responses from the viewpoint of potential users seeking mental health information.

As shown in Table 5 and Figure 7, empathy performance varied across models and differed across rating perspectives. In the psychiatrist-rated empathy assessment, Gemini 3.0 Pro, DeepSeek-V3, and DeepSeek-R1 showed the highest empathy performance, with median scores of 4.0 (3.0–4.0), 3.0 (3.0–4.0), and 3.0 (3.0–4.0), respectively. Their proportions of high-empathy responses were 54.2%, 43.8%, and 33.3%, respectively. In contrast, Claude Sonnet 4.5 Think and GPT-5.2 showed lower psychiatrist-rated high-empathy response rates, at 8.3% and 14.6%, respectively.

**Table 5:** Psychiatrist-rated and user-perspective empathy scores across AI chatbots.

Program	Psychiatrist-rated empathy score, median (IQR)	User-perspective empathy score, median (IQR)	Psychiatrist-rated high-empathy responses, *n* (%)	User-perspective high-empathy responses, *n* (%)
Gemini 3.0 Pro	4.0 (3.0–4.0)	3.0 (2.0–4.0)	26 (54.2%)	13 (27.1%)
DeepSeek-V3	3.0 (3.0–4.0)	3.0 (2.0–4.0)	21 (43.8%)	15 (31.2%)
DeepSeek-R1	3.0 (3.0–4.0)	2.0 (2.0–2.25)	16 (33.3%)	6 (12.5%)
Claude 4.5 Haiku	3.0 (3.0–4.0)	2.0 (2.0–3.0)	14 (29.2%)	2 (4.2%)
Claude Sonnet 4.5	3.0 (3.0–3.25)	2.0 (2.0–2.25)	12 (25.0%)	4 (8.3%)
GPT-5.2 Think	3.0 (3.0–3.0)	2.0 (2.0–2.0)	9 (18.8%)	2 (4.2%)
GPT-5.2	3.0 (2.0–3.0)	2.0 (2.0–2.0)	7 (14.6%)	0 (0.0%)
Claude Sonnet 4.5 Think	3.0 (3.0–3.0)	2.0 (2.0–3.0)	4 (8.3%)	4 (8.3%)

IQR, interquartile range.

In the user-perspective empathy assessment, overall empathy ratings were lower than psychiatrist-rated scores. DeepSeek-V3 and Gemini 3.0 Pro showed the highest user-perspective high-empathy response rates, at 31.2% and 27.1%, respectively, whereas GPT-5.2 received no high-empathy responses from user-perspective raters. These findings suggest that user-perspective raters appeared to apply stricter standards when judging whether a response felt emotionally supportive, understanding, and non-judgmental.

Table 6 presents exploratory Dunn's *post-hoc* pairwise comparisons following significant omnibus Kruskal–Wallis tests, identifying major model-pair differences in psychiatrist-rated and user-perspective empathy scores. In both rating perspectives, Gemini 3.0 Pro had significantly higher empathy scores than all models except DeepSeek-V3, consistent with the score distributions shown in Table 5 and Figure 7. The difference between Gemini 3.0 Pro and DeepSeek-V3 was not significant in either psychiatrist-rated or user-perspective assessment. In the psychiatrist-rated assessment, GPT-5.2 Think scored significantly higher than GPT-5.2 (*P* = 0.0192), whereas this difference was not significant in the user-perspective assessment (*P* = 0.9174). DeepSeek-V3 scored significantly higher than DeepSeek-R1 in both psychiatrist-rated and user-perspective assessments (*P* = 0.0371 and *P* < 0.0001, respectively). No significant differences were observed among the Claude variants in either rating perspective.

**Table 6:** Exploratory Dunn *post-hoc* pairwise comparisons for psychiatrist-rated and user-perspective empathy scores.

Comparison	Psychiatrist-rated empathy	User-perspective empathy
Claude 4.5 Haiku vs. Claude Sonnet 4.5	0.7422	0.9002
Claude 4.5 Haiku vs. Claude Sonnet 4.5 Think	0.3454	0.9536
Claude Sonnet 4.5 vs. Claude Sonnet 4.5 Think	0.1714	0.9249
Claude 4.5 Haiku vs. DeepSeek-R1	1.0000	0.9506
Claude Sonnet 4.5 vs. DeepSeek-R1	0.7333	0.9196
Claude Sonnet 4.5 Think vs. DeepSeek-R1	0.3592	0.9377
Claude 4.5 Haiku vs. DeepSeek-V3	0.0403	<0.0001
Claude Sonnet 4.5 vs. DeepSeek-V3	0.1098	<0.0001
Claude Sonnet 4.5 Think vs. DeepSeek-V3	0.0034	<0.0001
DeepSeek-R1 vs. DeepSeek-V3	0.0371	<0.0001
Claude 4.5 Haiku vs. Gemini 3.0 Pro	0.0028	0.0017
Claude Sonnet 4.5 vs. Gemini 3.0 Pro	0.0099	0.0008
Claude Sonnet 4.5 Think vs. Gemini 3.0 Pro	<0.0001	0.0023
DeepSeek-R1 vs. Gemini 3.0 Pro	0.0029	0.0011
DeepSeek-V3 vs. Gemini 3.0 Pro	0.3509	0.3708
Claude 4.5 Haiku vs. GPT-5.2	0.0189	0.1971
Claude Sonnet 4.5 vs. GPT-5.2	0.0058	0.2643
Claude Sonnet 4.5 Think vs. GPT-5.2	0.1998	0.1715
DeepSeek-R1 vs. GPT-5.2	0.0202	0.2473
DeepSeek-V3 vs. GPT-5.2	<0.0001	<0.0001
Gemini 3.0 Pro vs. GPT-5.2	<0.0001	<0.0001
Claude 4.5 Haiku vs. GPT-5.2 Think	0.9694	0.2814
Claude Sonnet 4.5 vs. GPT-5.2 Think	0.7409	0.3679
Claude Sonnet 4.5 Think vs. GPT-5.2 Think	0.3498	0.2628
DeepSeek-R1 vs. GPT-5.2 Think	0.9987	0.3447
DeepSeek-V3 vs. GPT-5.2 Think	0.0390	<0.0001
Gemini 3.0 Pro vs. GPT-5.2 Think	0.0029	<0.0001
GPT-5.2 vs. GPT-5.2 Think	0.0192	0.9174

### Correlation analysis of information quality, source transparency, and readability metrics

**Figure 8:**
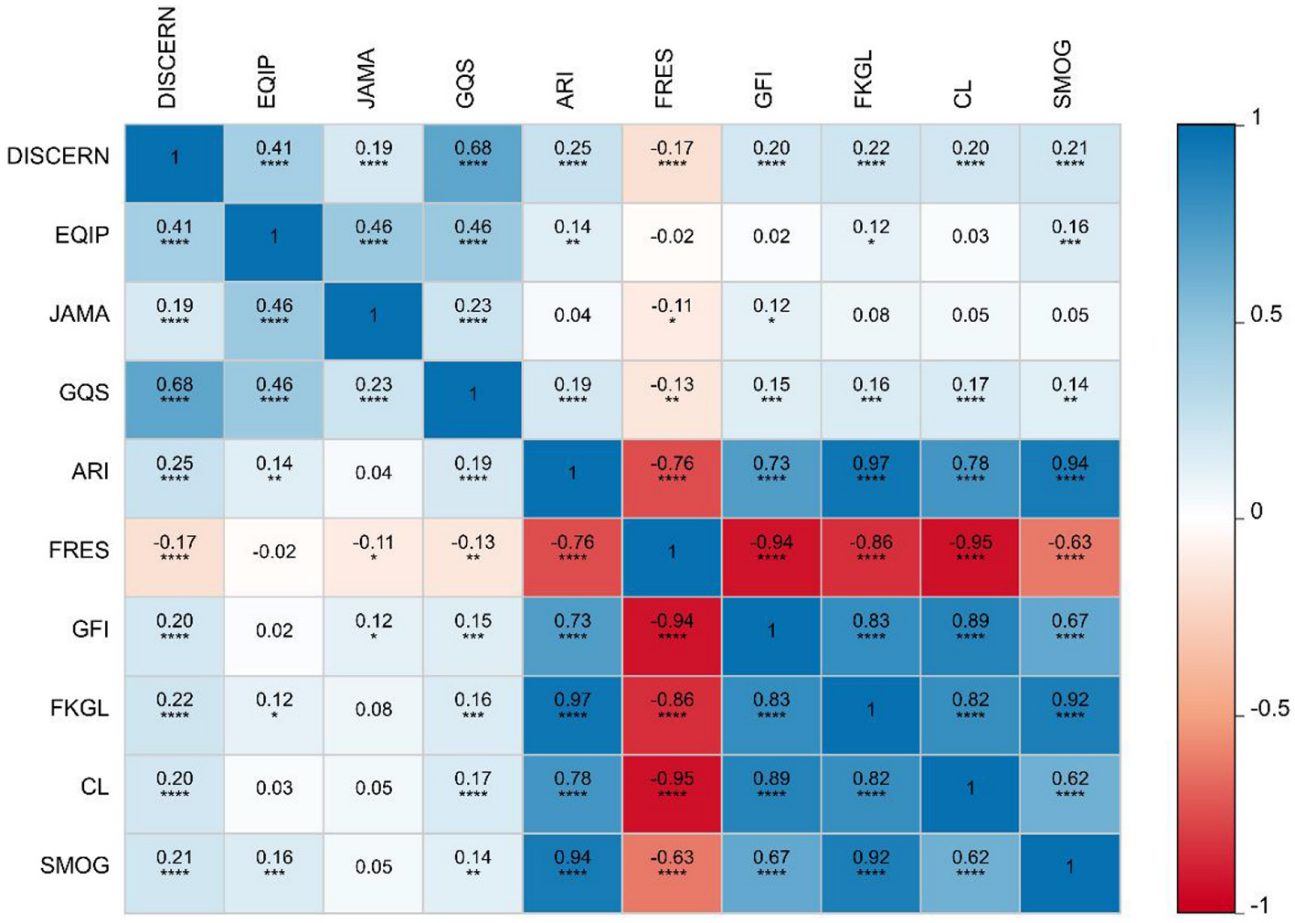
Spearman correlation matrix of information quality, source transparency, and readability metrics. Asterisks indicate statistical significance: *P < 0.05, **P < 0.01, ***P < 0.001, ****P < 0.0001.

Spearman correlation analysis was performed to examine the associations between information quality and source transparency metrics and readability metrics. As shown in Figure 8, the information quality and source transparency metrics showed modest positive correlations with one another (*r* = 0.19–0.46, all *P* < 0.0001). Readability metrics showed stronger internal correlations. ARI, GFI, FKGL, CL, and SMOG were strongly positively correlated (*r* = 0.62–0.97, all *P* < 0.0001), whereas FRES was strongly negatively correlated with these grade-level readability indices (*r* = −0.94 to −0.63, all *P* < 0.0001), consistent with the opposite scoring direction of FRES. In contrast, correlations between information quality and source transparency metrics and readability metrics were weak. Only DISCERN and GQS showed weak associations with selected readability metrics (*r* = 0.14–0.22, *P* < 0.01). These findings suggest that higher information quality and source transparency scores were not strongly associated with easier readability, indicating that information quality, source transparency, and readability represent relatively independent dimensions of LLM-generated mental health information.

### Overall item-level patterns of information quality, source transparency, and readability

**Figure 9:**
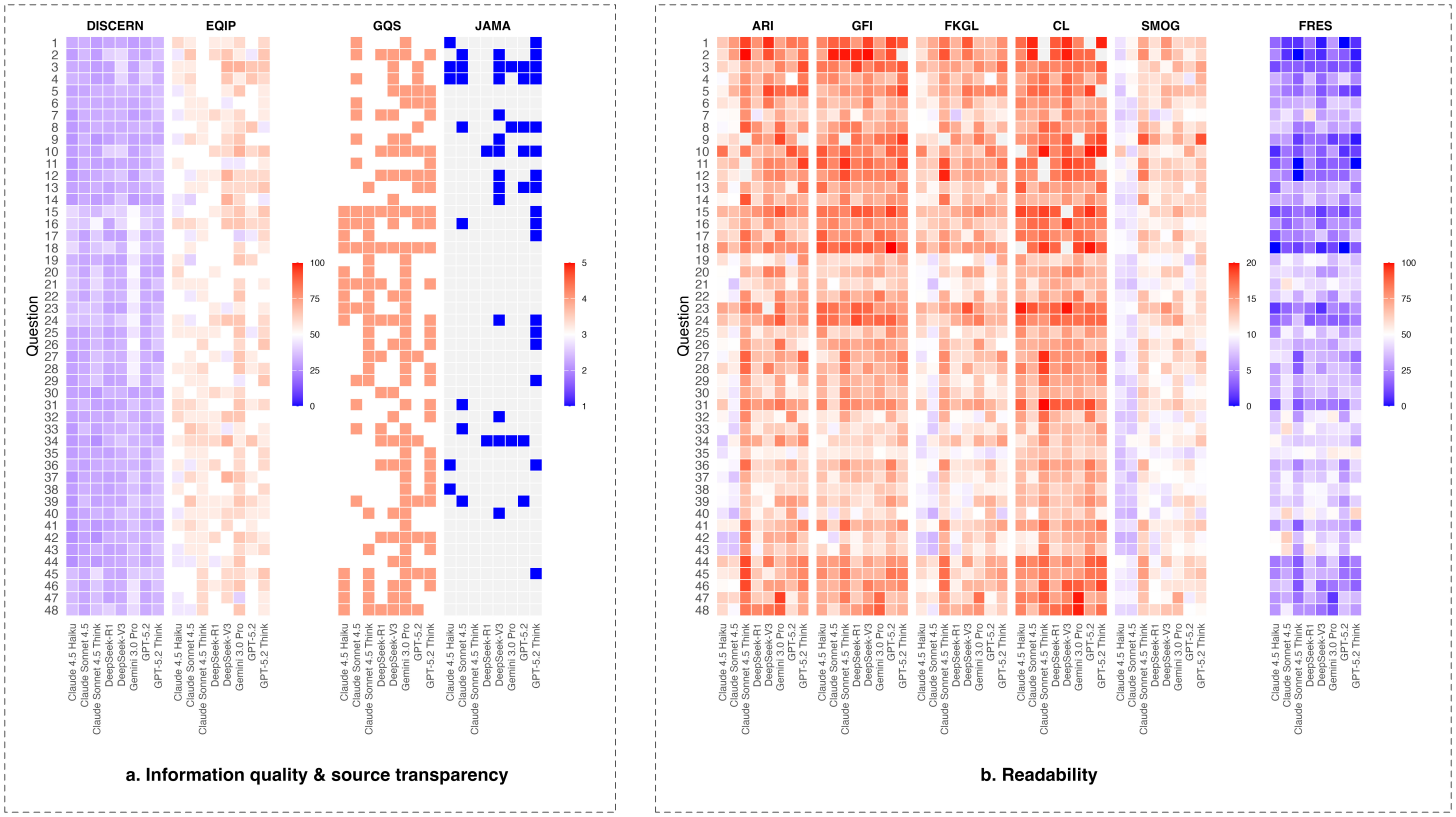
Item-level heatmap of information quality, source transparency, and readability scores across 48 standardized mental health questions and eight LLMs. **(a)** Shows information quality and source transparency metrics, and **(b)** shows readability metrics. The 48 questions were grouped into six domains: disease cognition, questions 1–8; clinical diagnosis, questions 9–14; treatment methods, questions 15–24; rehabilitation management, questions 25–30; social and family aspects, questions 31–43; and special issues, questions 44–48. The heatmap provides an exploratory overview of score variation across questions, models, and clinical domains.

Figure 9 presents the item-level distribution of information quality, source transparency, and readability scores across the 48 standardized questions and eight LLMs. JAMA scores remained consistently low across questions and models, indicating poor spontaneous source transparency. DISCERN and EQIP scores showed greater variation across items, suggesting heterogeneity in the completeness and structure of model responses. For readability, most responses exceeded the recommended sixth-grade reading level across domains. Overall, the item-level findings were consistent with the correlation analysis, showing variation in information quality, source transparency, and readability performance but no clear pattern indicating that higher information quality and source transparency scores corresponded to easier readability.

## Discussion

Unlike previous studies that evaluated single models or broad medical domains (42–44), we benchmarked eight mainstream LLMs on common psychiatric questions, assessing information quality, source transparency, readability, and empathy. By examining performance across psychiatric subdomains (e.g., diagnosis, treatment, rehabilitation), we provide evidence for context-specific model selection and safer use of LLM-generated psychiatric information.

### Information quality, source transparency, and variation by domain

We used three information quality metrics—DISCERN, EQIP, and GQS—to compare model performance on psychiatric queries (37–39). *Post-hoc* analyses suggested that Gemini 3.0 Pro and GPT-5.2 Think generally showed higher DISCERN, EQIP, and GQS scores than Claude 4.5 Haiku, Claude Sonnet 4.5, and DeepSeek-R1, indicating more structured, balanced, and comprehensive psychiatric health information. These differences may partly reflect how models represent clinical concepts and their associations.

However, source transparency remained a key weakness. On JAMA, Gemini 3.0 Pro scored zero, while GPT-5.2 Think had the highest score, indicating that high general quality scores do not guarantee adequate author attribution or references. Overall, JAMA performance was extremely low across models (median = 0; mean scores 0.35, 0.27, 0.17, 0.15, 0.10, 0.06, 0.04, and 0), consistent with prior evidence of limited source transparency in AI health information (45–47). Without verifiable sources, chatbots may spread incomplete or misleading claims, delaying care or causing inappropriate self-management in psychiatry. This failure may also result from systems being positioned as “wellness” tools rather than medical devices (48).

Beyond transparency, mental health applications carry safety risks. Over-agreement may validate delusional beliefs instead of supporting reality-testing, especially with hallucinated details (49). This highlights the need for source normativity, uncertainty disclosure, and safety guardrails with clinically grounded knowledge (49).

Model-level trends varied across model families and metrics. Exploratory pairwise comparisons suggested that GPT-5.2 Think showed higher DISCERN, EQIP, and JAMA scores than GPT-5.2, whereas Claude Sonnet 4.5 Think did not show consistent advantages over Claude Sonnet 4.5. In the DeepSeek series, DeepSeek-V3 showed higher scores than DeepSeek-R1 on selected information quality and source transparency metrics. These findings suggest that reasoning-enhanced or newer model versions do not uniformly improve psychiatric information quality, and performance gains should be verified empirically for each model and metric.

Across six problem domains, performance was weakest for “clinical diagnosis” and “disease cognition,” differing from osteoarthritis evaluations (50). Psychiatric diagnosis and disease understanding involve nuanced symptom descriptions and terminology, potentially affected by limited high-quality training data. Model-level trends also suggested domain-specific strengths. DeepSeek-V3 showed relatively better performance in “clinical diagnosis” and “disease cognition,” whereas Gemini 3.0 Pro showed relatively stronger performance in management-related domains, including “treatment methods,” “rehabilitation management,” “social and family,” and “special issues.” These results indicate that current LLMs show task- and domain-specific strengths rather than uniform excellence, supporting a model-selection strategy aligned with specific informational needs (e.g., diagnostic support vs. rehabilitation planning).

### Text readability performance and domain variation

We assessed the readability of LLM-generated psychiatric information and found that all models exceeded the sixth-grade level recommended by the American Medical Association, indicating text complexity above the comfortable reading range for the public. Consistent with prior findings (51–53), this is clinically relevant because complex health information can hinder patients and families from understanding disease-related knowledge. Among evaluated models, Claude Sonnet 4.5 generally produced more readable responses, though still below recommended standards. Claude Sonnet 4.5 Think generated the most complex text, suggesting enhanced thinking does not improve patient-facing clarity. These results point to a persistent gap between professional content and public comprehensibility. Since effective communication affects disease understanding and treatment adherence, readability optimization should be a core objective in future model development. Practical approaches include prompt strategies or post-processing that simplify language while preserving accuracy, and personalization methods that adapt text complexity to users' health literacy. Readability varied across six domains. Claude Sonnet 4.5 was more readable in clinical diagnosis, rehabilitation management, social and family support, special issues, and treatment approaches, while Claude 4.5 Haiku was more readable in disease cognition. Despite these differences, readability remained a constraint across models, highlighting the need for systematic simplification.

### Weak coupling between information quality and readability

Information quality and source transparency metrics showed modest internal correlations, but their associations with readability metrics were weak, consistent with prior findings (54–56). High-quality or well-structured information can still be difficult to read, meaning improvements in information quality do not automatically improve accessibility. Future health communication systems should treat information quality, source transparency, and readability as separate optimization targets and report them independently.

### Empathy and safety in psychiatric responses

Empathy is key to whether psychiatric guidance is understood, trusted, and accepted (57, 58). In the psychiatrist-rated empathy assessment, Gemini 3.0 Pro and DeepSeek-V3 showed the highest proportions of high-empathy responses, at 54.2% and 43.8%, respectively, while Claude Sonnet 4.5 Think and GPT-5.2 showed lower proportions, at 8.3% and 14.6%, respectively. In the user-perspective assessment, perceived empathy was generally lower across models. DeepSeek-V3 and Gemini 3.0 Pro still showed relatively better performance, with high-empathy response rates of 31.2% and 27.1%, respectively, whereas GPT-5.2 received no high-empathy responses from user-perspective raters. These findings suggest that empathy performance may depend on alignment and communication style rather than fluency or reasoning alone (59), and that clinicians and potential users may evaluate empathy from different perspectives. Psychiatrists may place greater weight on clinically appropriate reassurance, professional boundaries, and risk communication, whereas patients, family members, and members of the general public may place greater weight on emotional validation, warmth, non-judgmental tone, and whether the response feels personally supportive. These observations align with human–AI medical communication research: Ayers et al. used a 1–5 scale and the same ≥4 threshold, concluding that empathy and information quality should be evaluated as distinct dimensions (41). Broader psychotherapy evidence also supports the role of empathy in therapeutic processes (60).

However, both psychiatrist-rated and user-perspective empathy scores require caution because empathy ratings are subjective and may be influenced by response length, tone, and rater background. Ayers et al. noted that their empathy/quality scoring is not fully validated and that longer responses may appear more empathetic without addressing accuracy or fabrication risks (41). In psychiatry, empathy must be paired with boundary management and risk controls: when information quality, source transparency, and safety are insufficient, empathetic framing may increase acceptability of harmful content, highlighting the dual challenge of empathy and safety (13). Debates about the authenticity and potential adverse effects of “LLM empathy” support shifting optimization from “human-like warmth” to verifiable, controllable communication aligned with clinical ethics (61).

### Ethical and clinical governance considerations

LLM-generated mental health information also raises ethical concerns regarding accountability and the clinician–patient relationship (62). If inaccurate or unsafe LLM advice contributes to harm, responsibility should not be attributed solely to patients or to the model itself. Developers, platform providers, and healthcare organizations that deploy or recommend these tools should be responsible for appropriate validation, risk disclosure, monitoring, and escalation pathways. In psychiatric contexts, LLMs should be framed as supportive information tools rather than autonomous clinical decision-makers or substitutes for professional care. Carlbring and Andersson emphasized that the distinctive risk of LLMs lies in their interactivity, as fluent and responsive systems may appear personal or authoritative and may reinforce psychotic thinking through sycophancy, that is, excessive agreement or flattery (63). Therefore, safer psychiatric LLM systems should validate distress without endorsing delusional beliefs, encourage grounding and human help-seeking, and maintain clear role boundaries that support clinician judgment rather than replace it.

### Practical recommendations and future directions

Model deployment in psychiatry should target a multidimensional quality profile, including information quality, source transparency, readability, empathy, safety, and boundary management. For patient-facing psychoeducation, systems should prioritize readability while maintaining clinically verifiable guidance. For professional-facing support (e.g., differential diagnosis summaries or management overviews), information quality and structure may be prioritized but should still be paired with plain-language outputs for patients and caregivers. Developers should strengthen source transparency and implement empathy-aware guardrails that operationalize emotional validation, responses to distress, appropriate escalation, and boundary setting while avoiding generic consolatory language that can mask factual errors. Future research should retain the pragmatic ≥4/5 threshold for comparability (41), while decomposing empathy into measurable components and validating them with patient and clinician outcomes (60), testing stability in multi-turn dialogues where escalation is most relevant (13), and controlling for confounders such as response length and tone (41). Together, these steps can improve engagement, comprehension, and safer help-seeking in psychiatric contexts. From a public health and regulatory perspective, these findings support the need for transparent source attribution, health-literacy-sensitive language standards, and safety safeguards before LLM-generated mental health information is deployed for patient-facing use.

### Strengths and limitations of the study

This study has three key strengths. First, it provides one of the first multidimensional comparisons of eight mainstream LLM chatbots for public mental health information, covering information quality, source transparency, readability, and empathy. Second, the question bank was developed from multiple sources, including a literature review, Google Trends data, and anonymized real-world clinical questions. Compared with question banks based solely on researcher-generated prompts or published literature, this approach better reflected both public search behavior and common clinical information needs. Third, by jointly evaluating information quality, source transparency, readability, and empathy, this study highlights the need to consider these dimensions together when assessing the suitability of LLM-generated responses for public mental health information.

Several limitations should be acknowledged. First, this benchmark focused on public mental health questions and may not generalize to other medical specialties, clinical decision-making scenarios, or multi-turn patient–clinician interactions. Second, each model was evaluated using a single response per question at one time point; therefore, response variability, prompt sensitivity, temporal reliability, and reproducibility across repeated generations could not be assessed. Although this study evaluated information quality, credibility, and source transparency, it did not assess the stability of model outputs over time. Third, the question bank included both open-ended and closed-ended questions. Although identical wording was used across all models without additional prompt engineering, question structure may still have influenced response length, content scope, readability, and information quality scores. Fourth, although potentially harmful or misleading response patterns were considered during expert review, no dedicated harm-classification instrument was used; therefore, safety-related findings should be interpreted cautiously. Fifth, empathy ratings were subjective and may have been influenced by response length, tone, and rater background. Although the 5-point empathy scale was adapted from previous human–AI medical communication research, it remains a simplified summary measure rather than a fully validated multidimensional empathy instrument for psychiatric communication. Although we added user-perspective ratings to complement psychiatrist-rated empathy assessment, the number and diversity of user-perspective raters were limited. Finally, because LLMs are rapidly updated, performance measured during a specific query period may not reflect future model behavior.

## Conclusion

This study systematically evaluated eight mainstream LLMs on psychiatric health queries, covering information quality, source transparency, readability, and empathy. GPT-5.2 Think and Gemini 3.0 Pro showed higher information quality and source transparency scores and better information structuring, while Claude Sonnet 4.5 generated relatively more readable responses. However, important gaps remain in readability, source transparency, and patient-facing suitability. Empathy varied across models and differed between psychiatrist-rated and user-perspective assessments, indicating that empathic communication should be evaluated alongside information quality, source transparency, and readability and validated with intended users. Future development should prioritize readable, source-attributed, safe, and emotionally appropriate responses to better support patient education and decision-making in psychiatry.

## Data Availability

The raw data supporting the conclusions of this article will be made available by the authors, without undue reservation.
