# Supplementary material for: Information quality, readability, and empathy of AI-generated public mental health information: a comparative evaluation of eight large language models
Source: Front Public Health. 2026 Jun 26;14:1859078. doi: 10.3389/fpubh.2026.1859078 (PMC13350173; doi:10.3389/fpubh.2026.1859078)
Supplement: Supplementary file 1 [file Supplementary_file_1.docx]

**Supplementary Material 1: Standardized study prompts, reference answers, and complete chatbot outputs for the eight LLMs.**

**LLM Model: Claude Sonnet 4.5 Think**

**Q1. What is a mental illness?**

A mental illness refers to a category of mental health conditions that affect an individual's thinking, emotions, behaviors, and social functioning. These illnesses may lead to mood disorders, cognitive impairments, and behavioral problems, and in severe cases, can impact a person's daily life and interpersonal relationships.

Mental illnesses are diverse, including but not limited to:

**Depression**: Characterized by persistent sadness, loss of interest and pleasure, possibly accompanied by fatigue, insomnia, or hypersomnia.

**Anxiety disorders**: Including generalized anxiety disorder, social anxiety disorder, etc., mainly manifested by excessive worry and fear.

**Schizophrenia**: A severe mental illness that may cause hallucinations, delusions, and disorganized thinking.

**Bipolar disorder**: Marked by extreme mood swings, including depressive and manic episodes.

**Obsessive-Compulsive Disorder (OCD)**: Characterized by recurring obsessive thoughts and compulsive behaviors that individuals feel unable to control.

The causes of mental illnesses are complex and may be related to multiple factors such as genetics, environment, and psychological factors. Treatment usually includes psychotherapy, medication, and supportive interventions. Importantly, mental illnesses are treatable, and many people can resume normal lives through appropriate treatment.

**Q2. What are the common types of mental illnesses?**

Mental illnesses come in various types, and the following are some common ones:

**Depression**: Manifested by persistent sadness, loss of interest, fatigue, changes in appetite, and low self-esteem.

**Anxiety disorders**: Including generalized anxiety disorder, social anxiety disorder, and panic disorder, with the main feature of excessive worry and fear.

**Bipolar disorder**: Characterized by extreme mood swings, including depressive episodes and manic episodes.

**Schizophrenia**: A severe mental illness characterized by hallucinations, delusions, disorganized thinking, and impaired social functioning.

**Obsessive-Compulsive Disorder (OCD)**: Manifested by recurring obsessive thoughts and compulsive behaviors, which patients often feel unable to control.

**Post-Traumatic Stress Disorder (PTSD)**: After experiencing a traumatic event, patients may experience flashbacks, nightmares, and severe anxiety.

**Attention-Deficit/Hyperactivity Disorder (ADHD)**: Mainly manifested by inattention, impulsivity, and hyperactivity.

**Eating disorders**: Including anorexia nervosa, bulimia nervosa, and binge-eating disorder, mainly related to eating behaviors and body image.

**Personality disorders**: Including borderline personality disorder, antisocial personality disorder, etc., manifested by enduring patterns of thinking and behavior that affect interpersonal relationships and social functioning.

The symptoms and severity of these illnesses vary, and professional assessment and treatment are usually required. If you or someone you know has related symptoms, it is advisable to seek help from a mental health professional.

**Q3. Is anxiety disorder a type of mental illness?**

Yes, anxiety disorder is a type of mental illness. It is a common mental health problem characterized by persistent anxiety, fear, or worry that may affect a person's daily life and functioning. It includes various subtypes such as generalized anxiety disorder, social anxiety disorder, and panic disorder. Symptoms of anxiety disorder may include rapid heartbeat, sweating, trembling, fatigue, and difficulty concentrating. Treatment usually involves psychotherapy, medication, or a combination of both.

**Q4. Is depression a type of mental illness?**

Yes, depression is a type of mental illness. It is a common mental health disorder mainly characterized by persistent low mood, loss of interest, fatigue, difficulty concentrating, and changes in sleep and eating habits. Depression not only affects an individual's emotional and psychological state but may also significantly impact daily life, work, and interpersonal relationships. Treatment usually includes psychotherapy, medication, or a combination of both.

**Q5. What are the main causes of mental illnesses?**

The occurrence of mental illnesses is usually caused by the interaction of multiple factors, mainly including the following aspects:

- **Genetic factors**: Individuals with a family history of mental illnesses have a higher risk of developing the condition, indicating that genetics plays an important role in mental illnesses.
- **Biochemical factors**: Imbalances in neurotransmitters in the brain (such as dopamine, serotonin, etc.) may lead to changes in mood and behavior, thereby triggering mental illnesses.
- **Environmental factors**: Living environment, social support, economic status, educational level, etc., can all affect mental health. Experiencing negative events such as trauma, abuse, or loss of loved ones can also increase the risk of illness.
- **Psychological factors**: An individual's personality traits, ability to cope with stress, and cognitive styles are psychological factors that can affect mental health. For example, long-term emotional states such as anxiety and depression may lead to the development of mental illnesses.
- **Social factors**: Social environmental factors such as social isolation, discrimination, and life stress may also have a negative impact on mental health.
- **Physical factors**: Certain physical illnesses (such as chronic diseases, endocrine disorders, etc.) may also be associated with mental illnesses.

These factors are often intertwined, leading to the occurrence and development of mental illnesses. Therefore, understanding the causes of mental illnesses requires considering biological, psychological, and social factors comprehensively.

**Q6. Can insomnia or long-term staying up late increase the risk of mental illness?**

Insomnia and long-term staying up late can indeed increase the risk of mental illness. Studies have shown that insufficient sleep is associated with various mental health problems, including anxiety, depression, and other mood disorders. The following are key points:

- **The relationship between sleep and mental health**: Sleep is crucial for brain function. Lack of sleep can affect emotional regulation, cognitive function, and stress-coping abilities.
- **The impact of insomnia**: Long-term insomnia may lead to imbalances in neurochemicals in the brain, thereby increasing the risk of anxiety and depression.
- **The consequences of staying up late**: Staying up late not only affects physical health but may also cause mood swings and mental exhaustion, increasing the incidence of mental illnesses.
- **Disruption of the biological clock**: Long-term staying up late disrupts the biological clock, affecting hormone secretion and the body's natural rhythm, further exacerbating mental health problems.

Therefore, maintaining good sleep habits and a regular schedule is very important for maintaining mental health. If you or someone you know is experiencing insomnia or staying up late, seeking professional help and advice is crucial.

**Q7. How to judge if you may have a mental illness?**

You can self-assess whether you may have a mental illness from the following aspects:

- **Emotional changes**: Notice if you often feel extremely sad, anxious, angry, or have mood swings. Do these emotions affect your daily life and interpersonal relationships?
- **Behavioral changes**: Observe if you have obvious behavioral changes, such as loss of interest, social withdrawal, excessive reliance on drugs or alcohol, or impulsive behaviors.
- **Thinking patterns**: Reflect on whether you have negative self-evaluation, excessive worry, difficulty concentrating, or abnormal thinking such as hallucinations or delusions.
- **Physical symptoms**: Mental illnesses are often accompanied by physical symptoms such as insomnia, changes in appetite, and fatigue. If these symptoms persist and affect the quality of life, they may require attention.
- **Interpersonal relationships**: Evaluate if there are problems in your relationships with others, such as strained relationships with family and friends, or discomfort in work and social settings.
- **Quality of life**: Consider if your quality of life has declined, if you have difficulty completing daily tasks, or if you feel life is meaningless.

If you find obvious problems in these aspects, it is advisable to seek professional mental health assessment and help. Psychiatrists or psychologists can provide more in-depth assessment and support. Remember, seeking help in a timely manner is very important—mental health is just as important as physical health.

**Q8. Can self-assessment scales accurately determine if one has a mental illness?**

Self-assessment scales can provide certain references in judging whether one has a mental illness, but they cannot replace professional diagnosis. The following are key points:

- **Limitations of self-assessment**: Self-assessment scales are usually based on personal subjective feelings and may be affected by emotions, cognitive biases, or the level of understanding of symptoms. Therefore, the results may not be sufficiently accurate.
- **The importance of professional diagnosis**: The diagnosis of mental illnesses usually requires a comprehensive assessment by professional mental health workers, including clinical interviews, observations, and standardized tests. Professionals can consider more factors such as medical history, family background, and social environment.
- **The role of self-assessment scales**: Although self-assessment scales cannot replace professional diagnosis, they can help individuals identify potential mental health problems and prompt them to seek professional help. They can serve as preliminary screening tools to help people understand their mental state.
- **Interpretation of results**: If the results of a self-assessment scale indicate a possible mental health problem, it is recommended to consult a mental health professional in a timely manner for further assessment and diagnosis.

In summary, self-assessment scales can be used as tools to understand one's mental health status but cannot be used alone to diagnose mental illnesses. Professional assessment and diagnosis are still necessary.

**Q9. Which department should I consult if I suspect I have a mental illness?**

If you suspect you have a mental illness, it is recommended to consult the **psychiatry department**. Psychiatrists specialize in addressing various mental health issues, including anxiety, depression, schizophrenia, etc. If you are unsure whether you need to see a psychiatrist, you can first consult a general practitioner or family doctor, who can conduct a preliminary assessment based on your symptoms and recommend the appropriate department.

**Q10. How to distinguish mental illnesses from other physical illnesses?**

Distinguishing mental illnesses from other physical illnesses usually involves the following aspects:

**Symptoms**

- **Mental illnesses**: Mainly manifested by abnormalities in mood, thinking, behavior, etc. For example, depression may present as persistent sadness, loss of interest, fatigue; anxiety disorder may present as excessive worry, tension, etc.
- **Physical illnesses**: Usually manifested by physical symptoms such as pain, fever, fatigue, indigestion. These symptoms can usually be confirmed through medical examinations (such as blood tests, imaging tests).

**Etiology**

- **Mental illnesses**: May be related to multiple factors such as genetics, environment, and psychological factors, often without clear physical lesions.
- **Physical illnesses**: Usually have clear physical causes such as infection, inflammation, tumors, and can be diagnosed through medical means.

**Diagnostic methods**

- **Mental illnesses**: Usually rely on clinical assessment, including medical history, symptom evaluation, and psychological tests. There are no specific biomarkers for diagnosis.
- **Physical illnesses**: Can be diagnosed through laboratory tests, imaging tests, etc., usually with clear biomarkers.

**Treatment methods**

- **Mental illnesses**: Treatment methods include psychotherapy, medication (such as antidepressants, anti-anxiety drugs), etc., which usually require comprehensive management.
- **Physical illnesses**: Treatment methods may include medication, surgery, physical therapy, etc., usually targeting specific physical problems.

**Mutual influence**

There is an interactive relationship between mental illnesses and physical illnesses. For example, long-term physical illnesses may lead to depression or anxiety, and mental illnesses may also affect physical health (such as causing decreased immune system function).

In practical situations, mental illnesses and physical illnesses may coexist, which is called comorbidity. Therefore, professional medical assessment and diagnosis are very important. If you have doubts, it is recommended to consult a professional mental health or medical expert.

**Q11. Why do hospitals require multiple examinations for diagnosing mental illnesses?**

Hospitals conduct multiple examinations when diagnosing mental illnesses mainly for the following reasons:

- **Complexity of symptoms**: Symptoms of mental illnesses are often complex and diverse, involving emotions, cognition, behavior, and other aspects. Through multiple examinations, the patient's condition can be comprehensively evaluated to ensure that no important information is missed.
- **Excluding other illnesses**: Many symptoms of mental illnesses may be similar to those of other physical illnesses, such as thyroid dysfunction, neurological diseases, etc. Therefore, physical examinations and laboratory tests can help exclude these potential physical causes.
- **Individual differences**: Each patient's condition and background are different, and different assessment tools and methods may be required. Multiple examinations can provide personalized diagnosis based on the patient's specific situation.
- **Comprehensive assessment**: Mental health assessment usually includes multiple methods such as psychological assessment, clinical interviews, and standardized questionnaires. This comprehensive assessment helps doctors better understand the patient's mental state and living environment.
- **Formulating treatment plans**: Accurate diagnosis is the basis for formulating effective treatment plans. Multiple examinations can provide necessary information to help doctors select the most appropriate treatment method.
- **Monitoring changes in condition**: During treatment, regular multiple examinations can help doctors monitor changes in the patient's condition, evaluate treatment effects, and adjust treatment plans as needed.

Through these examinations, doctors can diagnose mental illnesses more accurately, thereby providing patients with more effective treatment and support.

**Q12. Is psychological assessment helpful for diagnosing mental illnesses?**

Psychological assessment is indeed of great help in diagnosing mental illnesses. The following are key points:

- **Objective assessment**: Psychological assessment provides a standardized method to evaluate an individual's mental state and behavioral characteristics, helping professionals understand the patient's situation more objectively.
- **Identifying symptoms**: Through various scales and questionnaires, psychological assessment can help identify specific symptoms and problems, such as anxiety, depression, attention deficit, which are common manifestations of mental illnesses.
- **Diagnostic support**: Although psychological assessment cannot be used alone for diagnosis, it can be part of clinical assessment to help doctors confirm or rule out certain mental illness diagnoses.
- **Personalized treatment**: Assessment results can provide a basis for formulating personalized treatment plans, helping doctors select the most appropriate intervention measures.
- **Monitoring progress**: Regular psychological assessment can help monitor the patient's treatment progress, evaluate treatment effects, and adjust treatment plans as needed.

In summary, psychological assessment is an important tool for mental health assessment, but it should be used in conjunction with clinical interviews and other assessment methods to obtain a comprehensive diagnosis and treatment plan.

**Q13. What should I do if different psychiatrists give different diagnostic results?**

When different psychiatrists provide different diagnostic results, you can consider the following steps to handle the situation:

- **Seek a second opinion**: If you are uncertain or dissatisfied with a diagnosis, you can consider seeking the opinion of another psychiatrist. Ensure you choose a professional with a good reputation and experience.
- **Document symptoms in detail**: When meeting with doctors, record your symptoms, medical history, and any relevant life events in detail. This can help doctors better understand your situation and make a more accurate diagnosis.
- **Communicate with doctors**: Maintain open communication with each doctor, ask about their diagnostic basis and treatment recommendations. Understanding the perspectives and reasons of different doctors can help you make a more informed decision.
- **Consider comprehensive assessment**: Sometimes, mental health problems may be complex and require a multidisciplinary assessment. Consider consulting psychologists, social workers, or other professionals to obtain a more comprehensive assessment.
- **Focus on treatment effects**: If you have already started a certain treatment, observing its effect is also important. If the treatment is effective, it may indicate the diagnosis is correct; if there is no improvement, re-evaluation may be needed.
- **Trust your intuition**: Ultimately, your feelings about your own health are the most important. If you feel uneasy about a diagnosis or treatment plan, continue to find the right professionals and plan for you.

When dealing with different diagnostic results, maintaining an open mind and a positive attitude is key. Mental health is a complex field, and finding the right support and treatment plan may take time and patience.

**Q14. Will visiting a psychiatrist be considered as having a mental illness?**

Visiting a psychiatrist does not necessarily mean a person is considered to have a mental illness. Psychiatrists provide services including assessment and treatment of various mental health issues, such as anxiety, depression, stress management, emotional regulation, etc. Many people may seek help due to life stress, emotional distress, or other mental health problems, which do not necessarily constitute a mental illness.

In many cultures, there is still a certain stigma associated with seeking mental health support, but more and more people are realizing that mental health is just as important as physical health. Visiting a psychiatrist can be regarded as a positive act of self-care aimed at improving personal mental health and quality of life.

If you or someone you know is considering visiting a psychiatrist, it is important to understand that this is a way to seek help, not a negative label on the individual.

**Q15. What are the main methods for treating mental illnesses?**

The main methods for treating mental illnesses include the following:

- **Psychotherapy**: Also known as talk therapy, it mainly helps patients understand and cope with their emotional and behavioral problems through dialogue with mental health professionals. Common psychotherapy methods include:
  - Cognitive Behavioral Therapy (CBT)
  - Psychoanalytic Therapy
  - Interpersonal Therapy
  - Family Therapy
- **Medication**: Using drugs to help control symptoms. Common drugs include:
  - Antidepressants
  - Anti-anxiety drugs
  - Mood stabilizers
  - Antipsychotic drugs
- **Supportive therapy**: Providing emotional support and practical help through support groups or community resources to help patients establish social connections and coping strategies.
- **Lifestyle changes**: Including improving diet, increasing exercise, maintaining a regular schedule, and reducing stress, all of which help improve mental health.
- **Alternative therapies**: Some patients may try alternative therapies such as meditation, yoga, art therapy, or music therapy as supplements to traditional treatment.
- **Inpatient treatment**: For severe mental illnesses, inpatient treatment may be required to provide more intensive care and monitoring.

The effectiveness of each method varies from person to person, and a personalized treatment plan usually needs to be formulated based on the patient's specific situation.

**Q16. Which is more effective, psychiatric medication or psychotherapy?**

The effectiveness of psychiatric medication versus psychotherapy varies depending on individual differences, disease type, and severity. The following is a comparison of the two:

**Medication**

- **Indications**: Usually used to treat severe mental illnesses such as depression, anxiety disorder, schizophrenia, etc.
- **Effect**: Drugs can quickly relieve symptoms, especially during acute episodes.
- **Side effects**: May have side effects and need to be used under the guidance of a doctor.

**Psychotherapy**

- **Indications**: Suitable for various psychological problems, including mild to moderate depression, anxiety, post-traumatic stress disorder, etc.
- **Effect**: Helps patients better cope with problems by changing thinking patterns and behaviors, which usually takes a longer time to take effect.
- **Persistence**: The effects of psychotherapy are often more lasting, helping patients establish coping mechanisms.

**Combined treatment:**

Many studies have shown that the combination of medication and psychotherapy usually achieves better results, especially in the treatment of severe or complex mental illnesses.

In summary, the choice of treatment method should be based on the patient's specific situation, the severity of symptoms, and personal preferences. Discussing with a professional mental health doctor to formulate a personalized treatment plan is the most effective way.

**Q17. Is exercise effective for mental illnesses?**

Exercise does have a positive effect on the treatment of mental illnesses. Studies have shown that regular physical activity can improve mental health, reduce symptoms of anxiety and depression, and enhance emotional stability. The following are specific aspects of the effectiveness of exercise for mental illnesses:

- **Releasing endorphins**: Exercise can promote the release of endorphins, a natural "feel-good hormone" that can improve mood and reduce feelings of pain.
- **Reducing anxiety and depression**: Multiple studies have found that regular exercise can significantly reduce symptoms of anxiety and depression, and in some cases, is comparable to medication.
- **Improving sleep**: Exercise helps improve sleep quality, and good sleep is crucial for mental health.
- **Enhancing self-confidence and self-efficacy**: By setting and achieving exercise goals, individuals can enhance self-confidence and self-efficacy, which has a positive impact on mental health.
- **Social interaction**: Participating in group sports or fitness activities can promote social interaction, reduce loneliness, and strengthen the social support network.
- **Coping with stress**: Exercise is considered an effective stress management tool that can help individuals better cope with stress and challenges in life.

Although exercise is beneficial for many people, it is not the only treatment method for all mental illnesses. For severe mental illnesses, professional medical intervention and treatment are still needed. In summary, combining exercise, psychotherapy, and medication usually achieves better results.

**Q18. What types of drugs are commonly used to treat mental illnesses?**

Commonly used drugs for treating mental illnesses can be mainly divided into the following categories:

- **Antidepressants**: Used to treat depression and anxiety disorders. Common types include Selective Serotonin Reuptake Inhibitors (SSRIs, such as fluoxetine, sertraline) and Tricyclic Antidepressants (such as amitriptyline).
- **Antipsychotics**: Used to treat schizophrenia, bipolar disorder, etc. Divided into typical antipsychotics (such as chlorpromazine, haloperidol) and atypical antipsychotics (such as risperidone, olanzapine).
- **Mood stabilizers**: Mainly used to treat bipolar disorder. Common ones include lithium salts and certain antiepileptic drugs (such as sodium valproate, lamotrigine).
- **Anti-anxiety drugs**: Used to relieve anxiety symptoms. Common ones include benzodiazepines (such as diazepam, alprazolam).
- **Sedatives**: Used to relieve anxiety and insomnia in the short term. Common ones include barbiturates and certain antihistamines.

The use of these drugs needs to be under the guidance of a doctor to ensure safety and effectiveness. Different mental illnesses and the patient's specific situation may require different medication treatment plans.

**Q19. How long does it usually take for psychiatric drugs to take effect?**

The time it takes for psychiatric drugs to take effect varies depending on the type of drug, individual differences, and specific conditions. Generally speaking:

- **Antidepressants**: Usually take 2 to 6 weeks to show significant effects. Some drugs may start to relieve symptoms within a few days, but full efficacy usually takes longer.
- **Anti-anxiety drugs**: Such as benzodiazepines, may take effect within hours, but long-term use may lead to dependence. Other types of anti-anxiety drugs (such as SSRIs) also take several weeks to work.
- **Antipsychotics**: Usually start to take effect within a few days to a few weeks, but full efficacy may take several weeks.
- **Mood stabilizers**: Such as lithium salts, may take several weeks to achieve stable effects.

Everyone's response may be different. Therefore, when using psychiatric drugs, it is very important to communicate with the doctor regularly to evaluate efficacy and adjust the dosage.

**Q20. Will taking psychiatric drugs damage brain function?**

The impact of taking psychiatric drugs on brain function varies depending on the type of drug, dosage, duration of use, and individual differences. The following are key points:

- **Drug type**: Different types of psychiatric drugs (such as antidepressants, antipsychotics, anti-anxiety drugs, etc.) have different effects on the brain. For example, some antidepressants may improve mood and cognitive function, while some antipsychotics may cause cognitive decline when used long-term.
- **Short-term vs. long-term effects**: Short-term use of psychiatric drugs is usually safe and can effectively relieve symptoms. However, long-term use of certain drugs may lead to side effects, including cognitive decline, memory problems, or other neurological effects.
- **Individual differences**: Everyone's response to drugs is different. Some people may experience side effects, while others may have no obvious adverse reactions.
- **Monitoring and adjustment**: Doctors usually monitor the patient's response regularly and adjust the drug dosage or replace the drug as needed to minimize side effects and improve treatment effects.

In summary, psychiatric drugs are very important in the treatment of mental illnesses, but their use needs to be under the guidance of a doctor to ensure safety and effectiveness. If you have any concerns or side effects, it is recommended to communicate with your doctor in a timely manner.

**Q21. How to cope with side effects of psychiatric drugs, such as weight gain and drowsiness?**

To cope with side effects of psychiatric drugs such as weight gain and drowsiness, the following strategies can be adopted:

**1. Communicate with your doctor**

- Regular follow-up: Discuss side effects with your doctor, who may need to adjust the drug dosage or replace the drug.
- Report side effects: Promptly feedback any discomfort to your doctor for appropriate adjustments.

**2. Diet management**

- Healthy diet: Choose low-calorie, high-fiber foods such as fruits, vegetables, and whole grains, and avoid high-sugar and high-fat foods.
- Portion control: Pay attention to portion sizes and avoid overeating.

**3. Increase exercise**

- Regular exercise: At least 150 minutes of moderate-intensity exercise per week, such as brisk walking, swimming, or cycling, can help control weight and improve mood.
- Daily activities: Increase daily activity levels, such as walking, taking the stairs, etc.

**4. Improve sleep**

- Regular schedule: Maintain a regular schedule to ensure adequate sleep.
- Pre-sleep relaxation: Try meditation, deep breathing, or light reading to help fall asleep.

**5. Psychological support**

- Psychotherapy: Consider cognitive behavioral therapy (CBT) or other psychotherapy to help cope with emotional issues caused by side effects.
- Support groups: Join support groups to communicate with others who have similar experiences and share coping strategies.

**6. Monitor weight**

- Regular weighing: Monitor weight changes regularly and adjust diet and exercise plans in a timely manner.
- Food journal: Keep a food diary to help identify foods that may lead to weight gain.

**7. Drug alternatives**

- Ask about alternative drugs: Some drugs have fewer side effects; discuss with your doctor if there are alternative options.

**8. Nutritional supplements**

- Vitamins and minerals: Ensure adequate nutrient intake; consider vitamin and mineral supplements if necessary.

Before taking any measures, it is best to consult a doctor or professional to ensure the measures are suitable for your health condition.

**Q22. Can taking psychiatric drugs lead to addiction?**

Whether taking psychiatric drugs can lead to addiction depends on the specific type of drug and individual differences. The following are common situations:

- **Antidepressants**: Most antidepressants (such as SSRIs and SNRIs) usually do not cause physical dependence or addiction, but withdrawal symptoms may occur when stopping the drug.
- **Anti-anxiety drugs**: Certain anti-anxiety drugs (such as benzodiazepines) may cause dependence and addiction, especially with long-term use. Therefore, doctors usually recommend short-term use and gradual dosage reduction when stopping the drug.
- **Antipsychotics**: These drugs generally do not cause addiction, but may have other side effects and risks of long-term use.
- **Mood stabilizers**: Such as lithium salts, usually do not cause addiction, but blood drug concentration needs to be monitored regularly.

In summary, the use of psychiatric drugs should be under the guidance of a doctor, following prescriptions and recommendations to reduce the risk of addiction. If you have any concerns or discomfort, it is very important to communicate with your doctor in a timely manner.

**Q23. What is Modified Electroconvulsive Therapy (MECT)?**

Modified Electroconvulsive Therapy (MECT), also known as non-convulsive electroconvulsive therapy, is a non-invasive treatment method used to treat depression and other mental illnesses. Unlike traditional Electroconvulsive Therapy (ECT), MECT does not cause convulsions in patients, so it is considered a safer and better-tolerated option.

**Key features:**

- **Non-invasive**: No surgery or anesthesia is required during treatment, and patients can resume daily activities immediately after treatment.
- **Principle**: By applying electric current or magnetic fields to specific areas of the scalp, it stimulates neural activity, regulates mood and cognitive function.
- **Indications**: Mainly used to treat severe depression, especially in patients who do not respond to drug treatment. In addition, its efficacy for other mental illnesses such as anxiety disorder, obsessive-compulsive disorder, and post-traumatic stress disorder is also being studied.
- **Course of treatment**: Multiple treatments are usually required, and the course may last several weeks. The specific number and frequency depend on the patient's condition.
- **Side effects**: Relatively few, may include headache, scalp discomfort, etc., which are usually mild and temporary.

MECT provides a new treatment option for many patients, especially those who do not respond to traditional treatment methods.

**Q24. What diseases is Transcranial Magnetic Stimulation (TMS) mainly used to treat?**

Transcranial Magnetic Stimulation (TMS) is a non-invasive brain stimulation technology mainly used to treat the following diseases:

- **Depression**: TMS is widely used to treat severe depression, especially in patients who do not respond to traditional drug treatment.
- **Anxiety disorder**: Some studies have shown that TMS can help relieve anxiety symptoms.
- **Obsessive-Compulsive Disorder (OCD)**: TMS is used to treat OCD in some cases, especially when other treatment methods are ineffective.
- **Post-Traumatic Stress Disorder (PTSD)**: TMS may be helpful for PTSD patients, especially when drug treatment is not effective.
- **Chronic pain**: TMS is also used to treat certain types of chronic pain, such as fibromyalgia.
- **Post-stroke rehabilitation**: TMS can be used to promote the rehabilitation of stroke patients and help improve motor function.
- **Alzheimer's disease and other neurodegenerative diseases**: Although research is still ongoing, TMS may have potential in improving cognitive function.
- **Migraine**: Some studies have shown that TMS can be used to relieve migraine attacks.

The specific indications and effects of TMS vary from person to person. A professional doctor's assessment and recommendation are usually required before treatment.

**Q25. Can I stop taking psychiatric drugs on my own after symptoms improve?**

Whether you can stop taking psychiatric drugs on your own after symptoms improve is a complex issue, and it is usually not recommended for patients to decide to stop taking drugs on their own. The following are factors to consider:

- **Doctor's advice**: Stopping the drug should be under the guidance of a doctor. The doctor will evaluate whether it is suitable to stop taking the drug based on your condition, type of drug, and treatment response.
- **Gradual dosage reduction**: Many psychiatric drugs need to be gradually reduced rather than stopped suddenly to avoid withdrawal symptoms or recurrence of the condition.
- **Symptom monitoring**: Even if symptoms improve, regular monitoring is still needed to ensure there are no signs of recurrence.
- **Individual differences**: Everyone's response to drugs is different. Some people may need longer-term treatment to stabilize their condition.
- **Psychological support**: After stopping the drug, additional psychological support or treatment may be needed to help cope with possible mood swings.

In summary, stopping psychiatric drugs should be under the guidance of a professional doctor to ensure safety and effectiveness.

**Q26. How long do I need to continue taking psychiatric drugs after the condition stabilizes?**

The duration of continued use of psychiatric drugs after the condition stabilizes varies from person to person and usually depends on the following factors:

- **Type of condition**: Different mental illnesses (such as depression, anxiety disorder, schizophrenia, etc.) have different degrees of dependence on drugs, and treatment plans will also vary.
- **Degree of condition stability**: If the condition is stable under drug treatment, the doctor may recommend continuing to take the drug for a period of time to prevent recurrence. Generally speaking, the doctor may recommend continuing to take the drug for 6 months to 1 year after the symptoms completely disappear.
- **Doctor's advice**: Following the doctor's advice is very important. The doctor will decide whether to continue medication based on your specific situation, drug side effects, and your response.
- **Gradual dosage reduction**: When deciding to stop taking the drug, it is usually recommended to gradually reduce the dosage rather than stop suddenly to reduce the risk of recurrence and withdrawal symptoms.

In summary, it is recommended to have a detailed discussion with your attending doctor to formulate a personalized treatment plan suitable for you.

**Q27. Can mental illnesses be completely cured?**

The cure of mental illnesses varies depending on individual differences and types of diseases. Certain mental illnesses, such as depression and anxiety disorder, can be significantly improved through appropriate treatment (such as psychotherapy, medication, and lifestyle changes), and even achieve complete remission in some cases. However, other mental illnesses, such as bipolar disorder or schizophrenia, may require long-term management. Although symptoms can be controlled, they may not be completely cured.

Importantly, early intervention and continuous support can significantly improve treatment outcomes. Everyone's situation is unique, so cooperating with professional mental health workers to formulate a personalized treatment plan is very important.

**Q28. Will mental illnesses recur after recovery?**

Whether mental illnesses will recur after recovery usually depends on multiple factors, including the individual's condition, effectiveness of treatment, strength of the support system, and living environment. The following are key points:

- **Type of disease**: Different types of mental illnesses (such as depression, anxiety disorder, bipolar disorder, etc.) have different recurrence risks. Some diseases have a higher recurrence rate, while others are relatively lower.
- **Treatment and management**: Continuous treatment and management (such as medication, psychotherapy, lifestyle changes, etc.) can significantly reduce the risk of recurrence. Following the doctor's advice and regular re-examinations are very important.
- **Support system**: Support from family, friends, and professionals can help individuals better cope with stress and challenges, thereby reducing the possibility of recurrence.
- **Lifestyle**: A healthy lifestyle, including a regular schedule, balanced diet, appropriate exercise, and good stress management, can enhance psychological resilience and reduce the risk of recurrence.
- **Early identification**: Timely identifying early signs of recurrence and taking measures can effectively prevent the deterioration of the condition.

In summary, although mental illnesses may recur after recovery, through appropriate treatment and support, many people can effectively manage their symptoms and live a fulfilling life. If you or someone you know is experiencing such a situation, seeking professional help is very important.

**Q29. How to prevent the recurrence of mental illnesses?**

Preventing the recurrence of mental illnesses is a complex process that usually requires a combination of multiple strategies. The following are effective methods:

- **Regular medical visits**: Regularly meet with mental health professionals for assessment and treatment adjustments. Follow the doctor's advice and take medication on time.
- **Establish a support system**: Maintain contact with family, friends, and support groups, share feelings and experiences, and obtain emotional support.
- **Healthy lifestyle**:
  - Balanced diet: Intake sufficient nutrients and avoid excessive sugar and caffeine.
  - Regular exercise: Regular exercise helps improve mood and reduce anxiety.
  - Adequate sleep: Maintain good sleep habits and ensure sufficient rest every night.
- **Cope with stress**: Learn effective stress management skills such as meditation, deep breathing, yoga, etc., to help relieve anxiety and stress.
- **Identify triggers**: Understand the triggers that may lead to recurrence and develop coping strategies. For example, avoid overwork, mood swings, or interpersonal conflicts.
- **Set realistic goals**: Set achievable short-term and long-term goals, maintain a positive attitude towards life, and enhance self-confidence.
- **Educate yourself**: Learn about your illness, acquire relevant knowledge, and enhance self-management capabilities.
- **Avoid drugs and alcohol**: Avoid using substances that may worsen symptoms, such as alcohol and drugs.

Through these methods, the risk of recurrence of mental illnesses can be effectively reduced. If you or someone around you is experiencing mental health problems, seeking professional help is very important.

**Q30. Will mental illnesses be inherited by the next generation?**

The heritability of mental illnesses is a complex issue. Studies have shown that certain mental illnesses do have a genetic predisposition. The following are key points:

- **Genetic factors**: Many mental illnesses, such as depression, anxiety disorder, bipolar disorder, and schizophrenia, have been found to be more common in families. This indicates that genetic factors may play a role in the development of these diseases.
- **Environmental factors**: In addition to genetics, environmental factors also have an important impact on the occurrence of mental illnesses. Life experiences, family environment, social support, and stress can all affect whether a person develops a mental illness.
- **Polygenic influence**: Mental illnesses are usually not determined by a single gene but by the complex interaction of multiple genes and environmental factors. This means that even if there is a family history, the next generation will not necessarily inherit the same disease.
- **Risk assessment**: If there is a history of mental illness in the family, the risk for the next generation may increase, but this does not mean they will definitely develop the illness. Scientists are working to identify specific genes and biomarkers associated with mental illnesses to better understand the genetic mechanisms of these diseases.

In summary, the heritability of mental illnesses is a multi-factor process. Genetic factors may indeed increase the risk, but environment and personal experiences are equally important. If you have relevant concerns, it is recommended to consult a professional mental health expert.

**Q31 How can people with mental illnesses better reintegrate into society?**

The social reintegration of people with mental illnesses is a complex and important process that involves support and intervention in multiple aspects. Here are some effective strategies and suggestions to help people with mental illnesses better integrate into society:

**Psychological Treatment and Support**

- Provide professional psychological treatments, such as Cognitive Behavioral Therapy (CBT) or supportive psychotherapy, to help patients understand and manage their symptoms.
- Establish support groups to allow patients to communicate with others who have similar experiences, share experiences and emotions, and reduce loneliness.

**Medication Treatment**

- Ensure patients follow the doctor's medication plan, attend regular follow-up visits, and adjust medications to control symptoms and reduce the risk of recurrence.

**Vocational Training and Employment Support**

- Offer vocational training and skill enhancement courses to help patients acquire skills needed for employment.
- Cooperate with enterprises to create suitable job opportunities for people with mental illnesses and provide a flexible working environment.

**Social Skills Training**

- Conduct social skills training to help patients improve their interpersonal communication abilities, enhance self-confidence, and adapt to social life.

**Family Support**

- Educate patients' family members to help them understand mental illnesses and provide emotional support and practical assistance.
- Encourage family members to participate in the treatment process and strengthen the family support network.

**Resource Utilization**

- Utilize community resources, such as mental health centers, rehabilitation institutions, and volunteer organizations, to provide necessary support and services.
- Organize community activities to promote interaction between patients and society and enhance their sense of belonging.

**Education and Publicity**

- Improve public awareness of mental illnesses, reduce discrimination and prejudice, and create a more inclusive social environment.
- Through publicity activities, encourage society to understand and accept people with mental illnesses.

**Personalized Support Plans**

- Develop personalized rehabilitation plans based on patients' specific needs and conditions to ensure each patient receives suitable support.

Through the above measures, people with mental illnesses can gradually reintegrate into society in a safer and more supportive environment, improve their quality of life, and rebuild self-confidence and independence.

**Q32 How to respond to discrimination and remarks about people with mental illnesses?**

To respond to discrimination and remarks about people with mental illnesses, you can adopt the following strategies:

- **Education and Publicity**: Help others understand the nature of mental illnesses and eliminate misunderstandings and prejudices by sharing scientific knowledge and real cases. You can use social media, community activities, or lectures for publicity.
- **Active Communication**: If people around you have prejudices against mental illnesses, try to have open dialogues with them. Share personal experiences or relevant information to help them recognize that people with mental illnesses are ordinary people worthy of respect and understanding.
- **Seek Support**: Share your feelings with friends, family, or support groups and seek their understanding and support. Joining relevant support groups can provide more resources and suggestions.
- **Stay Calm**: When facing discrimination and negative comments, try to stay calm and not let emotions dominate your response. You can choose not to respond or defuse the embarrassment with humor.
- **Advocate Inclusion**: Actively advocate for an inclusive and understanding attitude in daily life. Influence others through your own actions and encourage more people to pay attention to and support mental health issues.
- **Professional Help**: If discrimination and remarks cause you psychological distress, consider seeking help from a professional psychologist to learn coping skills and enhance psychological resilience.

Through the above methods, you can effectively respond to discrimination and remarks about people with mental illnesses and promote social understanding and support for mental health.

**Q33 Can you have a romantic relationship with someone who has a mental illness?**

It is completely possible to have a romantic relationship with someone who has a mental illness, but some important factors need to be considered. First and foremost, understanding and support are key. Mental illnesses may affect a person's emotions, behaviors, and ways of thinking, so partners need to have empathy and patience.

Here are some suggestions to help you build a smoother relationship with someone who has a mental illness:

- **Understand the Condition**: Learn about your partner's mental illness, including symptoms, treatment methods, and potential triggers. This will help you better understand their experiences.
- **Communication**: Maintain open and honest communication. Encourage your partner to share their feelings and needs, and express your own feelings as well.
- **Set Boundaries**: Establish healthy boundaries in the relationship to ensure both parties feel safe and respected.
- **Support Treatment**: Encourage your partner to seek professional help and provide support when they need it. Understand their treatment plan and respect their choices.
- **Take Care of Yourself**: While supporting your partner, also pay attention to your own emotional and mental health. Ensure you have a sufficient support system to cope with potential challenges.
- **Patience and Understanding**: Mental illnesses may lead to emotional fluctuations and behavioral changes, so being patient and understanding is crucial.

In summary, although having a romantic relationship with someone who has a mental illness may face some challenges, building a healthy relationship is completely possible through understanding, communication, and support.

**Q34 Can people with mental illnesses get married?**

People with mental illnesses **can get married**. Legal and social regulations generally do not restrict the right to marry of people with mental illnesses. Everyone has the right to choose their partner and establish a family, and mental illness does not hinder this right.

However, people with mental illnesses may need to consider the following points before getting married:

- **Health Status**: Patients should understand their own condition and, if necessary, seek professional medical advice to ensure they can properly manage their health during married life.
- **Partner's Understanding and Support**: Choosing someone who understands and supports you is very important. A partner's support can help patients better cope with the challenges brought by the illness.
- **Communication**: Open and honest communication is key in marriage. Patients should share their conditions and feelings with their partners so that both parties can face potential difficulties together.
- **Legal Issues**: In some cases, mental illness may affect legal capacity, such as when signing contracts or making legal decisions. Therefore, it is necessary to understand relevant legal regulations.

In conclusion, people with mental illnesses can get married, but it is very important to consider factors such as health, partner's support, and communication before entering into marriage.

**Q35 How to get along better with family members who have a mental illness?**

Getting along with family members who have a mental illness may face many challenges, but through understanding, patience, and support, a healthier relationship can be established. Here are some suggestions to help you get along better with them:

- **Learn About Mental Illness**: Acquire knowledge about the mental illness they are suffering from, including symptoms, treatment methods, and potential impacts. This will help you better understand their behaviors and feelings.
- **Maintain Communication**: Keep open communication with family members and listen to their feelings and needs. Avoid judgment or criticism and try to respond with understanding and support.
- **Set Boundaries**: While supporting them, also set healthy boundaries to protect your own emotional and mental health. Clarify the scope of support you can provide and seek help when needed.
- **Encourage Treatment**: If they have not yet received treatment, encourage them to seek professional help. You can accompany them to see a doctor or psychologist to help them overcome their fear of treatment.
- **Provide Support**: Offer practical support when they need it, such as accompanying them to activities, helping them manage daily affairs, or providing comfort when they feel anxious.
- **Take Care of Yourself**: Taking care of a family member with a mental illness can be exhausting, so make sure you also take care of your own physical and mental health. Rest regularly, engage in activities you enjoy, and maintain social connections.
- **Seek Support**: Join a support group or seek help from professionals to share your experiences and feelings. This can help you gain new perspectives and coping strategies.
- **Be Patient**: The recovery process from mental illness may be long and tortuous. Be patient and understanding, and give them time and space to cope with their own challenges.

Through these methods, you can better support your family members while also taking care of yourself. It is important to remember that building a healthy relationship takes time and effort.

**Q36 How to judge if a family member is experiencing a recurrence of a mental illness?**

You can judge if a family member is experiencing a recurrence of a mental illness by observing changes in the following aspects:

- **Emotional Changes**: Pay attention to whether the family member's emotions have obvious fluctuations, such as suddenly becoming abnormally anxious, depressed, irritable, or low in mood.
- **Behavioral Changes**: Observe if their behaviors are abnormal, such as social withdrawal, loss of interest, changes in eating habits, or sleep patterns (such as insomnia or excessive sleepiness).
- **Thinking Changes**: Be alert for signs of hallucinations, delusions, or confused thinking, such as hearing non-existent voices or having distorted perceptions of reality.
- **Daily Functioning**: Assess if their performance at work, study, or family life has declined, and if they have difficulty completing daily tasks.
- **Physical Symptoms**: Recurrence of some mental illnesses may be accompanied by physical symptoms, such as fatigue, headaches, or other discomforts.
- **Medication Adherence**: If the family member is receiving treatment, pay attention to whether they are taking medications on time and if there are any missed doses or discontinuations.
- **Social Interaction**: Observe if their interaction with others has decreased and if they have become more isolated.

If you notice the above signs, it is recommended to communicate with a professional psychologist or psychiatrist in a timely manner for evaluation and necessary intervention. Early identification and intervention can help improve the condition and reduce the impact of recurrence.

**Q37 What to do when a family member experiences auditory hallucinations?**

When a family member experiences auditory hallucinations, handling the situation requires caution and sensitivity. Here are some suggestions:

- **Stay Calm**: First, stay calm and patient. Auditory hallucinations can be confusing and frightening, and your calmness can help ease the tension.
- **Listen and Understand**: Listen to their feelings and do not immediately deny or question their experiences. Let them know you care about their feelings and are willing to listen.
- **Avoid Arguments**: Do not try to argue with them or correct the content of their auditory hallucinations, as this may increase their anxiety and unease.
- **Provide Support**: Ask them what kind of support they need, whether they want you to accompany them, or if they need professional help.
- **Seek Professional Help**: If the auditory hallucinations persist or affect their daily life, it is recommended to seek help from a mental health professional. A doctor or psychologist can provide an assessment and treatment plan.
- **Prioritize Safety**: If the auditory hallucinations are accompanied by tendencies of self-harm or harming others, be sure to seek emergency help immediately.
- **Understand the Cause**: Learn about the possible causes of the auditory hallucinations, such as stress, anxiety, depression, or other mental health issues, to help you better understand and support them.
- **Maintain Contact**: Communicate with them regularly, stay in touch, and help them feel supported and understood.

When dealing with auditory hallucinations, it is important to show compassion and understanding while encouraging them to seek professional help.

**Q38 What to do if a person with a mental illness engages in suicidal behavior?**

When a person with a mental illness engages in suicidal behavior, taking timely and effective measures is crucial. Here are some suggestions:

- **Immediate Intervention**: If you find the patient has suicidal tendencies or behaviors, first ensure their safety. Try to place them in a safe environment and avoid access to items that may cause self-harm.
- **Listen and Support**: Have an open dialogue with the patient, listen to their feelings and thoughts. Express your concern and support, and let them know they are not alone.
- **Seek Professional Help**: Encourage the patient to seek help from a mental health professional, such as a psychologist or psychiatrist. Professional intervention can provide necessary treatment and support.
- **Contact Emergency Services**: If the patient is in a critical condition or you believe they may attempt suicide immediately, be sure to contact emergency services or take them to the hospital.
- **Develop a Safety Plan**: Work with the patient to develop a safety plan, including identifying triggers, coping strategies, and emergency contact information. This can help them find support during a crisis.
- **Educate Yourself and Others**: Learn about mental illness and suicide risk-related knowledge to better understand the patient's situation and provide effective support.
- **Maintain Contact**: After the patient receives treatment, continue to stay in touch, regularly ask about their condition, and provide emotional support.
- **Take Care of Yourself**: Taking care of a person with a mental illness can be stressful for caregivers. Ensure you also receive the necessary support and care.

When handling such situations, staying calm and patient is very important. Each patient's situation is unique, so appropriate measures should be taken based on specific circumstances.

**Q39 What to do if a person with a mental illness shows violent tendencies?**

When a person with a mental illness shows violent tendencies, handling the situation requires caution and professionalism. Here are some suggestions:

- **Stay Calm**: First, stay calm and rational, and avoid provoking the patient. Your emotions will affect their response.
- **Assess Danger**: Observe the patient's behavior and emotions to assess if they pose a threat to themselves or others. If there is an immediate danger, professional help should be sought.
- **Seek Professional Help**: Contact a mental health professional, such as a psychologist or psychiatrist. They can provide a professional assessment and intervention.
- **Provide Support**: Try to communicate with the patient, express concern and understanding. Listen to their feelings and help them express emotions instead of suppressing them.
- **Develop a Safety Plan**: If the patient has violent tendencies, develop a safety plan that includes emergency contact information and a safe location.
- **Avoid Confrontation**: When communicating with the patient, avoid confrontation and accusations. Use gentle language and non-threatening body language.
- **Educate Yourself**: Learn about the patient's mental illness and the behaviors it may cause to help you better understand and respond.
- **Prioritize Self-Protection**: If the situation becomes dangerous, ensuring your own safety is the top priority. If necessary, seek help from the police or other emergency services.
- **Follow-Up**: After the crisis, continue to pay attention to the patient's condition to ensure they receive the necessary treatment and support.

Handling violent tendencies in people with mental illnesses requires patience and professional knowledge, and seeking help in a timely manner is key.

**Q40 What to do if a person with a mental illness refuses to see a doctor?**

Persuading a person with a mental illness to see a doctor can be a sensitive and complex process. Here are some suggestions to help you communicate more effectively:

- **Build Trust**: First, ensure the patient feels safe and understood. Listen to their feelings and concerns without rushing to give advice or make judgments.
- **Express Concern**: Express your concern for them in a warm and caring tone. You can say: "I care deeply about your health and hope you can get the help you need."
- **Provide Information**: Sometimes, patients refuse to see a doctor due to misunderstandings or fears about treatment. Provide accurate information about mental health and treatment to help them understand the benefits of seeing a doctor.
- **Share Personal Experiences**: If appropriate, share your own or others' positive experiences of seeing a doctor, emphasizing how treatment has improved quality of life.
- **Take Small Steps**: Suggest they first have a simple consultation instead of immediately undergoing comprehensive treatment. This can reduce their psychological burden.
- **Respect Their Choice**: If they still refuse, respect their decision. Coercion may be counterproductive and lead to increased resistance.
- **Seek Professional Help**: If the situation is serious, consider seeking help from a professional, such as a psychologist or psychiatrist, who may have more effective strategies to persuade the patient.
- **Offer Support**: Indicate that you are willing to accompany them to see the doctor or help them find suitable medical resources, so they do not feel alone.
- **Pay Attention to Crisis Situations**: If the patient's condition is critical, ensure professional help is sought in a timely manner to protect their safety.

Through patience and understanding, gradually guiding the patient to accept treatment is likely to yield better results.

**Q41 Should family members with mental illnesses be allowed to manage money independently?**

Allowing family members with mental illnesses to manage money independently is a complex and sensitive issue that involves considering multiple aspects. Here are some factors to weigh:

- **Type and Severity of Mental Illness**: Different mental illnesses have different impacts on an individual's cognition, judgment, and self-management abilities. For example, severe depression, schizophrenia, or bipolar disorder may affect a person's decision-making ability, while mild anxiety or depression may not.
- **Individual's Ability and Willingness**: Some people with mental illnesses can still effectively manage their finances. Understanding their abilities and willingness is key. You can assess their understanding and confidence in financial management through communication with them.
- **Support System**: If the family member has a good support system (such as family, friends, or professionals), they may be more capable of managing finances independently. Conversely, if support is lacking, more supervision and assistance may be needed.
- **Financial Education**: Providing financial management education and resources can help family members with mental illnesses better understand and manage their money. This may include basic knowledge of budgeting, saving, and expense management.
- **Legal and Ethical Considerations**: In some cases, legal guardianship or financial power of attorney may be required to protect the individual's interests. This usually involves legal procedures and requires the participation of professionals.
- **Risk Assessment**: Considering potential risks, such as impulsive spending, fraud, or poor financial management, it is necessary to assess these risks and take corresponding measures.

In general, whether to allow a family member with a mental illness to manage money independently should be evaluated based on individual circumstances, and may require combining the opinions and suggestions of professionals. When making a decision, respecting their autonomy and dignity is also very important.

**Q42 What are the fastest ways to find a missing person with a mental illness?**

When looking for a missing person with a mental illness, fast and effective methods include the following steps:

- **Call the Police Immediately**: Contact the local police as soon as possible and provide detailed information about the patient, including name, age, physical characteristics, clothing, time and place of disappearance, etc.
- **Contact Family and Friends**: Notify the patient's family, friends, and acquaintances, and ask if they know the patient's whereabouts or have seen the patient.
- **Check Familiar Places**: Understand the patient's daily activities and frequently visited places, such as parks, stores, hospitals, etc., and search on-site.
- **Use Social Media**: Post a missing person notice on social media with the patient's photo and detailed information to expand the search scope.
- **Contact Mental Health Institutions**: Contact the patient's treating doctor or mental health institution and ask if they have any suggestions or resources to help with the search.
- **Post Missing Person Notices**: Put up missing person notices in public places such as communities, hospitals, and schools to increase visibility.
- **Use Volunteers and Community Resources**: Seek help from volunteers and organize community search teams to expand the search scope and efficiency.
- **Maintain Contact**: Keep in touch with the police and other relevant institutions and update information and progress in a timely manner.

During the search process, stay calm and patient, and try to avoid excessive anxiety to search more effectively.

**Q43 As a family member of a person with a mental illness, how to relieve your own stress?**

As a family member of a person with a mental illness, facing stress and challenges is very common. Here are some suggestions to relieve your own stress:

- **Seek Support**: Share your feelings with other family members or friends, join a support group, and learn about others' experiences and coping strategies.
- **Acquire Knowledge**: Learn about mental illness-related knowledge to help you better understand the patient's condition and needs, thereby reducing anxiety caused by uncertainty.
- **Take Care of Yourself**: Ensure you have time for self-care, including exercise, a healthy diet, and adequate sleep. Engage in relaxation activities regularly, such as meditation, yoga, or walking.
- **Set Boundaries**: Learn to set reasonable boundaries and avoid taking on excessive responsibilities. Clarify your abilities and limitations and seek professional help when necessary.
- **Professional Counseling**: Consider seeking psychological counseling or therapy. Professionals can provide effective coping strategies and emotional support.
- **Maintain Communication**: Keep open communication with the patient, listen to their feelings, and express your own feelings to establish a mutually understanding and supportive relationship.
- **Engage in Activities**: Participate in hobbies or social activities to help yourself relax and distract your attention.
- **Record Emotions**: By keeping a diary or recording emotions, help yourself sort out your thoughts, identify stressors, and find coping methods.
- **Seek Professional Help**: If you feel overly stressed, seek help from a psychologist or therapist in a timely manner for professional psychological counseling.

Remember, taking care of yourself is not selfish, but rather to better support others. By adopting positive coping measures, you can manage stress more effectively and improve your own mental health.

**Q44 If a child shows "being quiet, irritable, and reluctant to go to school", could there be mental health issues?**

If a child shows "being quiet, irritable, and reluctant to go to school", it may indeed be related to mental health issues. These behaviors may be caused by a variety of factors, including:

- **Emotional Problems**: Emotional disorders such as anxiety and depression may cause children to show social withdrawal, irritability, and decreased interest in learning.
- **Social Difficulties**: Children may feel uncomfortable interacting with peers or adults, leading them to be unwilling to speak or participate in social activities.
- **Learning Disabilities**: If a child encounters difficulties in learning, they may feel frustrated, thereby developing a reluctance to go to school.
- **Family Environment**: Tense family relationships, parental pressure, or an unhealthy family atmosphere may also affect a child's emotions and behaviors.
- **Developmental Issues**: Certain developmental disorders, such as autism spectrum disorder, may cause children to have difficulties in social interaction and communication.

If these behaviors persist and affect the child's daily life and learning, it is recommended to seek professional psychological counseling or evaluation to identify potential problems in a timely manner and take appropriate intervention measures. Early intervention can help children better cope with these challenges.

**Q45 Can psychiatric medications be taken during pregnancy or while preparing for pregnancy?**

Taking psychiatric medications during pregnancy or while preparing for pregnancy requires careful handling, as certain medications may have adverse effects on the fetus. Here are some key points:

- **Type of Medication**: Different psychiatric medications have different effects on pregnant women and fetuses. For example, some antidepressants (such as SSRIs) are relatively safe during pregnancy, while other medications (such as lithium and certain antipsychotics) may increase the risk of fetal malformations or other hazards.
- **Risk-Benefit Assessment**: Doctors usually conduct a risk-benefit assessment based on the pregnant woman's specific situation (such as the severity of the mental health condition, the potential risks of the medication, etc.). In some cases, continuing to take the medication may be less risky than discontinuing it.
- **Preconception Period**: If you plan to become pregnant, it is recommended to discuss medication use with your doctor before conception. The doctor may suggest adjusting the medication or finding an alternative treatment plan.
- **Monitoring and Support**: If a pregnant woman needs to continue taking psychiatric medications, the doctor will closely monitor the health of the pregnant woman and the fetus and provide necessary support.

In summary, whether psychiatric medications can be taken during pregnancy or while preparing for pregnancy should be evaluated and guided by a professional doctor based on individual circumstances. Be sure to make any medication adjustments under the doctor's advice.

**Q46 Can psychiatric medications be taken simultaneously with other medications?**

Whether psychiatric medications can be taken simultaneously with other medications usually depends on the specific types of medications, the patient's health status, and the doctor's advice. Here are some factors to consider:

- **Drug Interactions**: Certain psychiatric medications may interact with other medications, leading to increased side effects or reduced efficacy. For example, when antidepressants are used together with certain painkillers or antibiotics, they may affect each other's metabolism.
- **Patient's Health Status**: The patient's overall health status, other illnesses, and medications being taken will all affect whether psychiatric medications can be taken simultaneously.
- **Doctor's Guidance**: Before starting any new medication treatment, it is very important to consult a doctor. The doctor will assess potential drug interactions and risks based on the patient's specific situation.
- **Monitor Side Effects**: If the doctor decides to use multiple medications simultaneously, the patient needs to regularly monitor for possible side effects and communicate with the doctor in a timely manner.

In summary, psychiatric medications can be taken simultaneously with other medications, but this must be done under the doctor's guidance to ensure safety and effectiveness.

**Q47 Can alcohol, coffee, or strong tea be consumed while taking psychiatric medications?**

The safety of consuming alcohol, coffee, or strong tea while taking psychiatric medications depends on the specific type of medication and the individual's health status. Here are some general guidelines:

- **Alcohol**: Many psychiatric medications (such as antidepressants, anxiolytics, and antipsychotics) may interact with alcohol, increasing the risk of side effects such as drowsiness, dizziness, and decreased cognitive function. Therefore, it is usually recommended to avoid alcohol while taking these medications.
- **Coffee and Strong Tea**: Coffee and strong tea contain caffeine, which may affect the effectiveness of certain psychiatric medications. For example, caffeine may increase anxiety or affect sleep quality. For certain medications (such as some antidepressants), caffeine intake may interfere with the drug's metabolism.
- **Individual Differences**: Everyone reacts differently to medications and stimulants. Therefore, while taking psychiatric medications, it is best to consult a doctor or pharmacist to understand the specific drug interactions and recommendations.

In summary, while taking psychiatric medications, it is best to avoid alcohol and use coffee and strong tea with caution. Be sure to communicate with your doctor to obtain personalized advice.

**Q48 Are there any dietary taboos for people with mental illnesses while taking medications?**

Dietary taboos for people with mental illnesses while taking medications mainly depend on the type of medication used and the patient's specific situation. Here are some common dietary taboos and suggestions:

- **Avoid Alcohol**: Many psychiatric medications interact with alcohol, which may increase side effects or reduce efficacy.
- **Limit Caffeine**: Caffeine in coffee, tea, and certain soft drinks may affect the effectiveness of medications, especially antidepressants and anxiolytics.
- **Be Cautious with High-Salt Foods**: Certain antipsychotic medications may cause weight gain and edema. Limiting high-salt foods helps control these side effects.
- **Avoid High-Sugar and High-Fat Foods**: These foods may lead to weight gain and affect overall health, especially in patients taking medications long-term.
- **Follow the Doctor's Dietary Advice**: Certain medications may require specific dietary coordination. For example, patients taking monoamine oxidase inhibitors (MAOIs) need to avoid foods containing tyramine (such as certain cheeses, pickled foods, and red wine).
- **Maintain a Balanced Diet**: Ensure adequate nutrient intake, including fruits, vegetables, whole grains, and protein, to support physical and mental health.
- **Consult Professionals**: Before changing dietary habits, it is best to consult a doctor or dietitian to ensure the diet is compatible with medication treatment.

In summary, people with mental illnesses should pay special attention to their diet while taking medications to avoid adverse reactions with the drugs and promote overall health.

**LLM Model: DeepSeek-V3**

**Q1. What is Mental Illness?**

Mental Illness, also known as Mental Disorder, refers to a range of health conditions that affect a person's thinking, emotions, behavior, or mood. It is not merely feeling down during difficult times but a clinically significant behavioral or psychological syndrome associated with distress or impairment in functioning.

**Core Characteristics:**

- Clinically significant disturbance: This disturbance is not a temporary emotional fluctuation or a normal reaction to life events, but a significant functional abnormality in cognition, emotional regulation, or behavior.
- Associated with distress or functional impairment: It causes great distress to individuals (such as anxiety, depression, fear) and severely impairs their functioning in important areas of life such as social interaction, occupation, or other key activities (e.g., inability to work, study, or maintain interpersonal relationships).

**Common Types of Mental Illness:**

- Anxiety Disorders: Such as Generalized Anxiety Disorder, Panic Disorder, Phobias, Obsessive-Compulsive Disorder (OCD). Characterized by excessive fear and anxiety disproportionate to the actual situation.
- Depressive Disorders: Such as Major Depressive Disorder. Core features include persistent low mood and loss of interest or pleasure.
- Bipolar and Related Disorders: Patients experience extreme mood swings between depression and mania (elevated mood and excessive energy).
- Schizophrenia Spectrum and Other Psychotic Disorders: Patients may experience hallucinations (e.g., hearing non-existent voices), delusions (firmly believing in unrealistic ideas), and disorganized thinking and speech.
- Trauma- and Stressor-Related Disorders: Such as Post-Traumatic Stress Disorder (PTSD), triggered by experiencing or witnessing a terrifying traumatic event.
- Eating Disorders: Such as Anorexia Nervosa, Bulimia Nervosa, involving severe disturbances in eating behaviors and related thoughts and emotions.
- Personality Disorders: Persistent, rigid, and maladaptive patterns of thinking and behavior that cause significant problems in interpersonal and social functioning.
- Neurodevelopmental Disorders: Such as Attention-Deficit/Hyperactivity Disorder (ADHD), Autism Spectrum Disorder, which usually emerge during developmental stages.

**Etiology:**

Mental illness is not caused by a single factor but typically results from the complex interaction of biological, psychological, and environmental factors.

- Biological Factors: Including imbalances in brain chemicals (neurotransmitters), genetics and family history, abnormalities in brain structure or function, infections, or congenital defects.
- Psychological Factors: Such as an individual's personality, long-term exposure to significant psychological stress, and childhood trauma or abuse.
- Environmental Factors: Such as major life events (loss of a loved one, unemployment), socioeconomic status, and exposure to toxic substances.

**Important Concepts:**

- It is not a personal fault or character flaw: Mental illness is a real medical condition, just like heart disease or diabetes, and cannot be overcome by simply "cheering up."
- It is treatable: Through psychotherapy (e.g., Cognitive Behavioral Therapy), medication, social support, and other interventions, most people with mental illness can effectively manage their symptoms and resume normal lives.
- Stigma must be eliminated: Misunderstanding and discrimination against mental illness in society often prevent patients from seeking help. Understanding and empathy are crucial.

In summary, mental illness is a common health issue affecting millions of people's daily lives. However, with proper diagnosis and treatment, recovery and a high quality of life are entirely possible. If you or someone you know is experiencing mental health distress, seeking professional help is the most important step toward recovery.

**Q2. What are the Common Types of Mental Illness?**

Here is an overview of the common types of mental illness (or psychological disorders).

Mental illness encompasses a wide range of conditions, which are mainly classified into the following major common types based on symptoms, etiology, and impact on functioning:

**1. Anxiety Disorders**

This is the most common category of mental illness, characterized by excessive, uncontrollable fear, worry, and anxiety that severely impacts daily life.

- Generalized Anxiety Disorder: Persistent and excessive worry about daily affairs.
- Panic Disorder: Recurrent sudden episodes of intense fear (panic attacks), accompanied by physical symptoms such as palpitations, sweating, and trembling.
- Social Anxiety Disorder: Intense fear of social or public situations, fear of being judged or embarrassed.
- Specific Phobias: Irrational, intense fear of specific objects or situations (e.g., heights, animals, flying).
- Separation Anxiety Disorder: Excessive anxiety when separated from attachment figures (common in children but can also occur in adults).

**2. Depressive Disorders**

Core features include persistent low mood, loss of interest, and decreased energy.

- Major Depressive Disorder: Severe depressive mood lasting at least two weeks, loss of interest in almost all activities, accompanied by changes in sleep, appetite, and concentration.
- Persistent Depressive Disorder: A chronic, longer-lasting (at least two years) form of depression with potentially milder symptoms.
- Seasonal Affective Disorder: Depressive episodes associated with specific seasons (usually autumn and winter).

**3. Bipolar and Related Disorders**

Characterized by extreme mood swings between elevated mood (mania or hypomania) and low mood (depression).

- Bipolar I Disorder: At least one full manic episode.
- Bipolar II Disorder: At least one hypomanic episode and one major depressive episode, with no full manic episodes.

**4. Schizophrenia Spectrum and Other Psychotic Disorders**

These disorders are characterized by severe disturbances in thinking, perception, and behavior, where patients may lose touch with reality.

**Core Symptoms:**

- Positive Symptoms: Such as hallucinations (hearing or seeing non-existent things), delusions (firmly held false beliefs), and disorganized thinking and speech.
- Negative Symptoms: Such as emotional blunting, poverty of speech, anhedonia (inability to experience pleasure), and social withdrawal.
- Cognitive Symptoms: Problems with attention, memory, and executive function.

**5. Obsessive-Compulsive and Related Disorders**

Characterized by recurrent, intrusive unwanted thoughts (obsessions) and/or repetitive behaviors or mental acts (compulsions).

- Obsessive-Compulsive Disorder (OCD): For example, repeated checking, cleaning, or counting to relieve anxiety caused by obsessions.
- Body Dysmorphic Disorder: Preoccupation with perceived physical flaws.
- Hoarding Disorder: Difficulty discarding items, leading to excessive accumulation that affects living space and safety.

**6. Trauma- and Stressor-Related Disorders**

Triggered by experiencing or witnessing traumatic or stressful events.

- Post-Traumatic Stress Disorder (PTSD): After a traumatic event, experiencing flashbacks, nightmares, severe anxiety, and uncontrollable recollection of the event.
- Acute Stress Disorder: Symptoms similar to PTSD but occurring within 3 days to 1 month after the traumatic event.
- Adjustment Disorders: Excessive emotional or behavioral reactions to identifiable stressors (e.g., divorce, unemployment).

**7. Dissociative Disorders**

Involve disturbances or disruptions in consciousness, memory, identity, or perception, often as a coping mechanism for trauma.

- Dissociative Identity Disorder: Formerly known as multiple personality disorder, characterized by the presence of two or more distinct personality states.
- Dissociative Amnesia: Inability to recall important personal information, usually traumatic or stressful.

**8. Somatic Symptom and Related Disorders**

Patients experience intense anxiety and symptoms related to the body, whose severity cannot be fully explained by medical conditions.

- Illness Anxiety Disorder: Preoccupation with fearing or believing one has a serious illness (formerly known as hypochondriasis).
- Conversion Disorder: Presence of symptoms related to voluntary motor or sensory function, suggesting a neurological disorder but inconsistent with medical findings.

**9. Feeding and Eating Disorders**

Involve severe disturbances in eating behaviors.

- Anorexia Nervosa: Achieving a significantly low body weight through dieting, excessive exercise, etc., accompanied by intense fear of weight gain.
- Bulimia Nervosa: Recurrent episodes of binge eating followed by compensatory behaviors (e.g., vomiting, misuse of laxatives) to prevent weight gain.
- Binge-Eating Disorder: Recurrent episodes of binge eating without subsequent compensatory behaviors.

**10. Substance-Related and Addictive Disorders**

Involve excessive use of or dependence on mood-altering substances (e.g., alcohol, opioids, stimulants) leading to clinically significant impairment. Also includes behavioral addictions such as Gambling Disorder.

**11. Neurodevelopmental Disorders**

These disorders usually emerge during developmental stages (childhood or adolescence).

- Attention-Deficit/Hyperactivity Disorder (ADHD): Persistent patterns of inattention and/or hyperactivity-impulsivity.
- Autism Spectrum Disorder: Persistent deficits in social communication and interaction, accompanied by restricted, repetitive patterns of behavior.
- Specific Learning Disorders: Difficulties in specific academic areas such as reading, writing, or mathematics.

**12. Personality Disorders**

Persistent, rigid, and maladaptive inner experiences and behavioral patterns that deviate from personal cultural expectations, causing functional impairment and distress.

- Cluster A: Odd, eccentric behavior (e.g., Paranoid, Schizoid, Schizotypal Personality Disorders).
- Cluster B: Dramatic, emotional, or unstable behavior (e.g., Borderline, Narcissistic, Histrionic, Antisocial Personality Disorders).
- Cluster C: Anxious, fearful behavior (e.g., Avoidant, Dependent, Obsessive-Compulsive Personality Disorders).

**Important Notes:**

- The above list is not exhaustive, and many patients may have more than one mental illness (comorbidity).
- This information is for educational purposes only and cannot be used for self-diagnosis. Diagnosis of mental illness is a complex process that must be conducted by trained professionals (e.g., psychiatrists, clinical psychologists) based on detailed assessments and diagnostic criteria (such as DSM-5 or ICD-11).
- If you or someone you know is experiencing mental health distress, seeking professional help is the most important step toward recovery. Most mental illnesses are effectively treatable and manageable.

**Q3. Is Anxiety Disorder a Type of Mental Illness?**

Yes, anxiety disorder is a type of mental illness (or mental disorder).

This is a very clear medical classification. To explain it more clearly, I have compiled a detailed explanation for you.

**Anxiety Disorder: Detailed Explanation of Being a Mental Disorder**

Anxiety Disorders are a group of mental disorders characterized by excessive, persistent fear, anxiety, and related behavioral disturbances. This anxiety is usually disproportionate to the actual situation, causing significant distress and severely interfering with patients' daily life, social interactions, and occupational functioning.

**1. Official Medical Classifications**

Anxiety disorders are clearly classified by the two major global authoritative diagnostic systems:

- International Classification of Diseases, 11th Revision (ICD-11): Published by the World Health Organization (WHO), it lists "Anxiety or Fear-Related Disorders" as an independent chapter.
- Diagnostic and Statistical Manual of Mental Disorders, 5th Edition (DSM-5): Published by the American Psychiatric Association (APA), where "Anxiety Disorders" is a major diagnostic category.

**2. Common Types of Anxiety Disorders**

| **Type** | **Core Characteristics** |
| --- | --- |
| Generalized Anxiety Disorder (GAD) | Persistent and uncontrollable excessive worry and tension about daily events or activities. |
| Panic Disorder | Sudden, recurrent episodes of intense fear (panic attacks), accompanied by physical symptoms such as palpitations, a sense of suffocation, and fear of dying. |
| Social Anxiety Disorder | Significant and persistent fear of one or more social situations, fear of being judged by others or engaging in embarrassing behaviors. |
| Specific Phobias | Intense, irrational fear of specific objects or situations (e.g., heights, animals, flying). |
| Separation Anxiety Disorder | Excessive anxiety disproportionate to developmental level when separated from attachment figures (common in children, also seen in adults). |
| Agoraphobia | Fear of being in places or situations where escape is difficult or help is unavailable (e.g., public transportation, crowded places). |

**3. Key Distinction: Pathological Anxiety vs. Normal Anxiety**

| **Feature** | **Normal Anxiety** | **Pathological Anxiety (Anxiety Disorder)** |
| --- | --- | --- |
| Severity | Proportionate to real threat | Excessive, disproportionate to actual situation |
| Duration | Brief, resolves with removal of stressor | Persistent (usually lasting more than 6 months) |
| Impact on Functioning | Mild, may enhance performance | Severe impairment in social, occupational, and other important functions |
| Controllability | Self-regulatable | Difficult to control, feeling helpless |

**4. Treatment Methods**

Anxiety disorders are treatable. Main methods include:

**Psychotherapy:**

- Cognitive Behavioral Therapy (CBT): The gold standard. Helps patients identify and change distorted thinking patterns and unsafe behavioral patterns.
- Exposure Therapy: Highly effective for phobias and panic disorder.

**Medication:**

- SSRIs/SNRIs (Selective Serotonin Reuptake Inhibitors/Serotonin-Norepinephrine Reuptake Inhibitors): First-line medications, such as Sertraline, Paroxetine, Venlafaxine, etc.
- Benzodiazepines: Rapid onset of action but usually used short-term due to addiction risk.

**Lifestyle Adjustments:**

- Regular exercise, mindfulness meditation, adequate sleep, and a healthy diet can all effectively assist in relieving anxiety symptoms.

**Important Notes**

Defining anxiety disorder as a "mental illness" is not stigmatization but rather to understand, diagnose, and treat it more scientifically. Just as the body can catch colds and fevers, the brain and mind can also "get sick"—this is very common and nothing to be ashamed of. If you or someone you know is struggling with this, seeking professional help from a psychiatrist or psychotherapist is the most important step toward recovery.

I hope this detailed explanation helps you fully understand anxiety disorders. Simply put, classifying it as a mental illness means it has received official recognition from the medical community, with a mature and scientific system for diagnosis and treatment—this is crucial for patients to receive proper help.

**Q4. Is Depression a Type of Mental Illness?**

Yes, depression (Depression) is a type of mental illness, more commonly referred to as a mental disorder or psychological disorder in professional fields.

This is a very clear medical classification. Here is a detailed explanation:

**1. Professional Definition and Classification**

In the world's authoritative disease classification systems, depression is clearly categorized as a mental disorder:

- In the World Health Organization (WHO)'s International Classification of Diseases (ICD-11), depression is classified under the chapter "Mood Disorders."
- In the American Psychiatric Association (APA)'s Diagnostic and Statistical Manual of Mental Disorders (DSM-5), depression is listed as the main type of "Depressive Disorders."

**2. Why is it a Mental Illness?**

Mental illness refers to clinically significant disturbances in a person's thinking, emotions, or behavior, causing distress or impairment in social functioning (such as work, study, or interpersonal skills). Depression fully meets this definition:

- Emotional disturbance: Persistent low mood, loss of interest, and inability to feel pleasure.
- Cognitive disturbance: May include pessimism, self-blame, feelings of worthlessness, and even suicidal thoughts.
- Physiological disturbance: Accompanied by sleep problems (insomnia or hypersomnia), significant changes in appetite and weight, decreased energy, and difficulty concentrating.
- Functional impairment: These symptoms are severe enough to affect patients' daily lives, work, and interpersonal relationships.

**3. Correcting a Common Misconception**

Many people immediately associate "mental illness" with severe mental disorders such as "schizophrenia," leading to stigmatization and fear. However, mental illness is a very broad category—just as "physical illness" includes colds, fractures, diabetes, and cancer, mental illness also includes:

- Common mental disorders: Such as depression and anxiety disorders, which are very prevalent.
- Severe mental disorders: Such as schizophrenia and bipolar disorder.

Defining depression as a mental illness is not to "label" patients but to:

- Promote scientific understanding: Recognize it as a real, biologically based illness, not "weak character," "being unable to cheer up," or "seeking attention."
- Enable effective treatment: Clarifying its medical nature means it requires and can be intervened in and cured through scientific methods (such as psychotherapy and medication).
- Eliminate prejudice: Promote public understanding, reduce patients' stigma, and encourage them to actively seek professional help.

**Summary:**

Depression is indeed a common mental disorder. It is caused by the interaction of biological, psychological, and social environmental factors, and is by no means a personal fault or defect. Recognizing this is the first step toward scientific understanding and effective treatment.

If you or someone around you is experiencing depressive symptoms, seeking professional help from a psychiatrist or psychotherapist is the best approach.

**Q5. What are the Main Causes of Mental Illness?**

This is a very important and complex question. Modern psychiatry and psychology generally believe that mental illness is not caused by a single factor but by the complex interaction of biological, psychological, social, and environmental factors. This is known as the Biopsychosocial Model.

The main causal factors can be divided into the following categories:

**1. Biological Factors**

These refer to internal factors related to the body's physiological structure and function.

- Genetic and Genetic Susceptibility: Many mental illnesses, such as schizophrenia, bipolar disorder, depression, and anxiety disorders, have obvious family aggregation. Individuals may inherit a "susceptibility" to a certain disease through genetics, but this does not mean they will definitely develop the illness—usually, environmental factors are needed to trigger it.
- Imbalances in Brain Chemicals: Neurotransmitters in the brain (such as serotonin, dopamine, norepinephrine) are responsible for transmitting information between neurons. Abnormal levels or dysfunction of these chemicals are closely related to various mental illnesses. For example, depression is often associated with low serotonin levels.
- Abnormalities in Brain Structure and Function: Through brain imaging techniques (such as fMRI, PET), it has been found that certain brain regions (such as the prefrontal cortex, amygdala, hippocampus) in patients with mental illnesses differ from those in healthy people in structure, size, or activity level.
- Congenital and Prenatal Factors: Infections during pregnancy, malnutrition, exposure to toxic substances or alcohol, and complications during childbirth (such as hypoxia) may increase the risk of mental illness in children in the future.
- Physical Illnesses and Injuries: Brain trauma, infections, strokes, tumors, endocrine disorders (such as thyroid dysfunction), and certain chronic diseases can directly or indirectly trigger mental symptoms.

**2. Psychological Factors**

These involve an individual's thinking, emotional, and behavioral patterns.

- Personality Traits: Certain personality traits may be risk factors for mental illness. For example, individuals with high neuroticism (tendency to experience negative emotions) are more likely to develop anxiety and depressive disorders.
- Cognitive Patterns: Persistently holding negative self-perceptions and pessimistic views of the world and the future (known as the "negative cognitive triad") is a core feature of depression. Distorted thinking patterns are also common in other illnesses.
- Childhood Trauma and Adverse Experiences: Abuse (physical, emotional, sexual), neglect, loss of parents, and severe family dysfunction during childhood are extremely high-risk factors for various mental disorders later in life (such as PTSD, Borderline Personality Disorder, depression).
- Coping Skills and Psychological Resilience: Lack of effective stress management and emotional regulation skills, and low psychological resilience, may make individuals more vulnerable to psychological problems when facing adversity.

**3. Social and Environmental Factors**

These refer to external pressures and influences from society and the environment.

- Life Stress Events: Major negative life events are common triggers, such as the death of a loved one, divorce, unemployment, financial difficulties, or experiencing a major accident or crime.
- Social Support System: Lack of stable, good family relationships, friendships, and social support networks can make people more vulnerable and less able to cope with stress. Strong social support, on the other hand, is a protective factor against psychological problems.
- Socioeconomic Status: Poverty, low social status, unemployment, and low educational level are associated with higher incidence of mental illness, which may be related to long-term exposure to life stress and resource scarcity.
- Cultural and Social Environment: The cultural background, social stigma, discrimination (based on race, gender, sexual orientation, etc.), war, and immigration adaptation issues all have a profound impact on mental health.

**Key Summary: Vulnerability-Stress Model**

To better understand how these factors interact, we can refer to a classic framework: the Vulnerability-Stress Model.

- Vulnerability: An individual's innate or acquired inherent tendency to develop illness (mainly from biological and psychological factors, such as genetic genes, childhood trauma).
- Stress: External life events or pressures that trigger the illness (mainly from social and environmental factors, such as unemployment, breakup).

This model suggests: When an individual's internal vulnerability is low, they may remain healthy even when facing significant stress. When an individual's internal vulnerability is high, even a minor stressor may become the "straw that breaks the camel's back," triggering mental illness.

In short, mental illness is the result of the interaction of all the above factors, rarely due to a single cause. Understanding this helps us reduce stigma toward patients and recognize that prevention and intervention need to be carried out at multiple levels.

If you or someone you know is experiencing psychological distress, seeking professional help (such as from a psychiatrist or psychotherapist) is crucial.

**Q6. Does Insomnia or Chronic Sleep Deprivation Increase the Risk of Mental Illness?**

Yes, insomnia and chronic sleep deprivation significantly increase the risk of developing mental illness. This is not just a feeling of fatigue but is supported by solid neuroscientific and clinical research.

Numerous studies have shown a strong bidirectional relationship between sleep disturbances and mental health problems: sleep problems are both early symptoms and warning signs of mental illness, and independent risk factors that drive their occurrence and development.

**How Do Sleep Disturbances Increase the Risk of Mental Illness?**

The underlying mechanisms are complex and interrelated, mainly involving the following aspects:

**Imbalances in Neurotransmitters**

- Dysfunction of the Dopamine System: Chronic sleep deprivation leads to overactivation of the brain's dopamine system, which is closely related to positive symptoms (such as hallucinations, delusions) of mental illnesses like schizophrenia and bipolar disorder. Lack of sleep makes the brain "overexcited," prone to distorted perceptions.
- Decreased Serotonin Levels: Sleep is crucial for regulating serotonin (5-HT), a key mood stabilizer. Insomnia disrupts serotonin balance, and low serotonin levels are a core feature of depression and anxiety disorders.

**Impaired Brain "Waste Clearance" System**

During deep sleep, the brain activates the "glymphatic system," an efficient cleaning system that removes metabolic waste (such as beta-amyloid) accumulated during the day.

Chronic sleep deprivation severely hinders this process, leading to the accumulation of neurotoxins. This not only increases the risk of neurodegenerative diseases (such as Alzheimer's disease) in the future but also creates a breeding ground for various mental disorders through neuroinflammation and cellular damage.

**Impaired Function of Emotional Regulation Centers**

Sleep deprivation impairs the function of the prefrontal cortex (responsible for rational thinking, decision-making, and emotional control).

At the same time, it enhances the response of the amygdala (the brain's fear and emotional center).

The result: You are more likely to be emotional, impulsive, negative, and have difficulty recovering from negative emotions, greatly increasing the risk of depression and anxiety.

**Dysregulation of the Endocrine and Stress Systems**

Staying up late activates the body's hypothalamic-pituitary-adrenal (HPA) axis, leading to abnormally elevated levels of the stress hormone cortisol at night.

Long-term high cortisol levels are toxic to the hippocampus (related to memory and emotional regulation) and keep the body in a constant "fight-or-flight" stress state—a typical physiological feature of anxiety disorders, PTSD, and depression.

**Disruption of Biological Rhythms (Circadian Rhythms)**

The body follows an internal 24-hour biological clock that regulates hormone secretion, body temperature, sleep-wake cycles, etc.

Chronic sleep deprivation completely disrupts this sophisticated rhythm system. For susceptible individuals, this disruption itself is a powerful trigger for bipolar disorder (extreme mood swings between depression and mania) and major depressive disorder.

**Association with Specific Mental Illnesses**

- Depression: Insomnia is one of the core symptoms of depression and the strongest predictor of depressive episodes. Approximately 75% of depression patients experience insomnia. Chronic sleep-deprived individuals are several times more likely to develop depression than those with healthy sleep.
- Anxiety Disorders: Lack of sleep directly amplifies anxiety and worry, forming a vicious cycle of "can't sleep because of anxiety, more anxious because can't sleep."
- Bipolar Disorder: Reduced sleep is often a precursor and driver of manic or hypomanic episodes. For diagnosed patients, maintaining a stable sleep-wake cycle is key to preventing recurrence.
- Schizophrenia: Sleep disorders are usually prodromal symptoms of schizophrenia, and patients may experience severe insomnia long before diagnosis. Sleep disturbances also exacerbate symptoms such as hallucinations and delusions.
- Attention-Deficit/Hyperactivity Disorder (ADHD): Sleep problems are highly overlapping with and mutually exacerbating ADHD symptoms; inattention, impulsivity, and hyperactivity may worsen when sleep-deprived.

**Important Notes and Recommendations**

- Bidirectional Relationship: It is important to understand that this relationship is bidirectional. Not only does staying up late cause mental illness, but existing mental health problems can also severely disrupt sleep. They often form a difficult-to-break vicious cycle.
- Individual Differences: Not everyone who stays up late will develop a mental illness; genetic susceptibility, environmental stress, personal psychological resilience, and other factors jointly determine the final risk.
- Early Intervention: If you are struggling with chronic insomnia or staying up late and it has started to affect your daytime mood and function, be sure to treat it as an important health alert.

**Recommended Actions:**

- Prioritize Sleep: Treat sleep as important as a healthy diet and exercise. Aim for 7-9 hours of quality sleep each night.
- Establish a Regular Schedule: Try to go to bed and wake up at the same time every day, even on weekends.
- Seek Professional Help: If self-adjustment is difficult or emotional problems have already emerged, do not hesitate to consult a psychiatrist or psychotherapist. Cognitive Behavioral Therapy for Insomnia (CBT-I) is the first-line treatment and is highly effective.

In short, treating sleep well means treating your brain and mental health well. Investing in sleep is one of the most important and effective health investments you can make.

**Q7. How to Determine if You May Have a Mental Illness?**

Determining whether you may have a mental illness requires careful consideration. I will provide you with a comprehensive reference framework to assist with preliminary self-observation, but please remember: this cannot replace a diagnosis from a professional doctor.

Let me compile a detailed guide for you:

**How to Determine if You May Have a Mental Illness: A Self-Observation Guide**

**Warning Signs to Pay Attention To**

**Emotional Changes**

- Persistent low mood: Feeling sad, empty, or hopeless for a long time (lasting more than 2 weeks)
- Extreme mood swings: Mood fluctuations far beyond the normal range, affecting daily life
- Inability to experience pleasure: Losing interest in activities you once enjoyed (anhedonia)
- Excessive anxiety: Persistent worry, tension, or panic that is difficult to control

**Cognitive Changes**

- Difficulty concentrating: Decreased work efficiency, frequent distraction
- Memory loss: Especially impairment of recent memory
- Difficulty making decisions: Struggling to make even small decisions
- Negative thinking patterns: Persistent self-blame, feelings of worthlessness, or pessimistic views
- Changes in sense of reality: Experiencing unusual beliefs or perceptual experiences

**Behavioral Changes**

- Social withdrawal: Avoiding contact with others, including relatives and friends
- Decline in daily functioning: Significant reduction in ability to work, study, or perform household chores
- Changes in sleep patterns: Oversleeping or insomnia, significant changes in appetite
- Neglect of self-care: Ignoring personal hygiene and appearance

**Physical Symptoms**

- Unexplained pain: Headaches, stomachaches, or other physical discomfort
- Persistent fatigue: Always feeling tired, even after rest
- Significant changes in sexual desire: Marked decrease or abnormal increase in sexual interest

**Self-Assessment Dimensions**

**Duration**

Symptoms lasting more than two weeks and occurring almost every day require special attention.

**Degree of Functional Impact**

Do the symptoms significantly affect your:

- Work or academic performance
- Quality of interpersonal relationships
- Daily living abilities
- Self-care abilities

**Level of Distress**

Subjective level of distress caused by the symptoms (rated on a scale of 0-10)

**Situations Requiring Immediate Help**

Seek professional help immediately if you experience:

- Thoughts of harming yourself or others
- Hallucinations or delusions
- Complete inability to perform daily activities
- Worsening substance abuse
- Extreme mood swings leading to loss of behavioral control

**Professional Help-Seeking Channels**

**Initial Assessment Options**

- Counselor: Provides preliminary assessment and psychological counseling
- Psychiatrist: Has medical diagnostic rights and prescribing authority
- Department of Psychology in a general hospital: Suitable for cases accompanied by physical symptoms

**Preparing for the Visit**

Before seeing a professional, you can prepare:

- Start time and changes of symptoms
- Specific examples of impact on life
- Family mental health history
- Current medications and health status

**Professional Diagnostic Process**

Formal diagnosis of mental illness requires:

- Clinical interview: Detailed understanding of symptoms and history
- Standardized assessment: Use of professional scales and tools
- Ruling out physical causes: Physical examination if necessary
- Comprehensive judgment: Based on diagnostic criteria (such as DSM-5 or ICD-11)

**Important Reminders**

- Self-observation is only the first step and cannot replace professional diagnosis
- Like physical illnesses, mental illnesses respond better to early intervention
- Seeking help is a sign of strength, not weakness
- Many mental health problems can be significantly improved with treatment

**Help Resources**

- National Mental Health Helpline: 12320 (China)
- Crisis Intervention Hotline: Hope 24 Hotline 400-161-9995

Note: The above information is for reference only and cannot replace professional medical advice. If you have any doubts, please seek professional help in a timely manner.

I have prepared this detailed guide covering the entire process from self-observation to professional help-seeking. It lists specific warning signs to watch for, assessment dimensions, and when to seek immediate help.

**Q8. Can Self-Assessment Scales Accurately Determine if Someone Has a Mental Illness?**

This is a very important and common question. The simple and direct answer is: No.

Self-assessment scales (such as common online depression, anxiety, and personality disorder tests) cannot be used as the final basis for diagnosing mental illness. They can only serve as a preliminary screening tool or reference for self-understanding, and must never replace clinical assessment by a professional psychiatrist or psychotherapist.

Below I will explain in detail why they cannot, and the correct way to use self-assessment scales.

**Why Can't Self-Assessment Scales Make Accurate Diagnoses?**

**Lack of Professionalism (Reliability and Validity Issues):**

- Many popular online tests may not be developed by professionals, and their scientific validity is questionable.
- Even professional scales (such as PHQ-9 for depression, GAD-7 for anxiety) need to be administered and interpreted by trained personnel in specific settings.
- Results of self-assessment scales are easily affected by the tester's current mood, environment, self-perception biases (such as excessive introspection or denial), leading to unstable results.

**Inability to Conduct Differential Diagnosis:**

- Symptoms of many mental illnesses overlap. For example, inattention may be a symptom of depression, anxiety disorder, or ADHD.
- Only psychiatrists can rule out other possibilities (such as thyroid dysfunction that can also cause emotional problems) through interviews, observations, medical history collection, and even necessary physical examinations to make an accurate "differential diagnosis."

**Inability to Assess Severity and Functional Impairment:**

- Diagnosing mental illness requires not only identifying symptoms but also assessing their severity, duration, and the extent of impairment to an individual's social functioning (such as work, study, interpersonal relationships).
- Self-assessment scales usually only calculate scores and cannot distinguish between significant differences such as "being unable to go to work due to low mood" and "slightly decreased work efficiency due to low mood."

**Risk of Misdiagnosis and Underdiagnosis:**

- Self-stigmatization: An inaccurately high score may label a person with an incorrect "illness label," leading to unnecessary fear, anxiety, and self-suggestion, which may worsen psychological burden.
- False sense of security: An inaccurately low score may make a person ignore actual serious problems, miss the best opportunity for intervention, and delay treatment.

**What is the Correct Role and Purpose of Self-Assessment Scales?**

Self-assessment scales are not useless; their value lies in:

- Preliminary screening and awareness raising: They can help you realize "my recent emotional state seems abnormal" and serve as a "warning light" to pay attention to your mental health.
- Self-tracking tool: During professional treatment, you can fill out the same scale regularly to track changes in condition and treatment effectiveness, providing reference for doctors to adjust treatment plans.
- Starting point for communication: Scale results can serve as an entry point for communication with professionals. You can tell the doctor: "Doctor, I recently took this test and got a high score. I'm worried, so I came to consult you."

**If You Are Concerned About Your Mental Health, the Correct Steps Are:**

1. Self-awareness: Pay attention to persistent (usually more than two weeks) changes in your emotions, thinking, and behavior.
2. Conduct preliminary self-screening using reliable scales (optional but cautious): Choose scales recommended by hospitals or authoritative mental health institutions.
3. Seek professional help: This is the most crucial and indispensable step. Please visit:
   - Psychiatry department (or mental health department) of a regular hospital: Psychiatrists have prescribing authority and can provide medical diagnosis and medication treatment.
   - Professional psychological counseling/therapy institutions: Counselors/therapists can provide psychological assessment and counseling/therapy.

**Summary:**

You can think of self-assessment scales as a "self-check thermometer." A high thermometer reading indicates you may have a fever, but it cannot tell you whether you have the flu, COVID-19, or a common cold. Similarly, a high score on a self-assessment scale suggests your psychological "emotional temperature" may be abnormal, but the specific problem and its severity must be ultimately determined and treated by a professional "mental health doctor."

Please take good care of your mental health. If you have any doubts, seeking professional help is the most responsible and effective choice.

**Common Mental Health Self-Assessment Scales (For Reference Only)**

Important note: The following scales are for popular science and preliminary self-screening reference only and cannot be used as diagnostic basis. Interpretation of results requires professional assessment.

**1. Depression Self-Assessment**

- Patient Health Questionnaire-9 (PHQ-9)
- Purpose: Assess the frequency and severity of depressive symptoms in the past two weeks.
- Scoring: Rated 0-4 points per item, total score 0-27 points.
  - 0-4 points: No depressive symptoms
  - 5-9 points: Mild depression
  - 10-14 points: Moderate depression
  - 15-19 points: Moderate-severe depression
  - 20-27 points: Severe depression

(It is usually recommended that those with a score ≥10 seek professional attention)

**2. Anxiety Self-Assessment**

- Generalized Anxiety Disorder Scale-7 (GAD-7)
- Purpose: Assess the severity of anxiety symptoms in the past two weeks.
- Scoring: Rated 0-3 points per item, total score 0-21 points.
  - 0-4 points: No anxiety
  - 5-9 points: Mild anxiety
  - 10-14 points: Moderate anxiety
  - 15-21 points: Severe anxiety

**3. Stress Self-Assessment**

- Perceived Stress Scale (PSS-10)
- Purpose: Assess the degree to which individuals perceive life situations as uncontrollable, unpredictable, and overwhelming.
- Scoring: Rate 10 items; higher total score indicates higher perceived stress level.

**4. Sleep Quality Self-Assessment**

- Pittsburgh Sleep Quality Index (PSQI)
- Purpose: Assess sleep quality in the past month.
- Scoring: Includes 7 components, total score 0-21 points.
  - Total score ≤5: Good sleep quality
  - Total score >5: Poor sleep quality

**How to Use This Information?**

If you feel worried after self-testing (high score or persistent symptoms), the most important next step is to make an appointment with a psychiatrist or psychotherapist.

You can record your self-test results and specific feelings to use as a starting point for communication with professionals.

Do not "diagnose" or "medicate" yourself based solely on scores.

I have compiled a list of common mental health self-assessment scales, including their purposes and simple scoring instructions. Please remember that this information is for your reference and preliminary understanding only and must never replace professional medical diagnosis. If you have any doubts about the test results or your own state, be sure to seek professional help.

**Q9. Which Department Should I Visit if I Suspect I Have a Mental Illness?**

If you or someone around you suspects having a mental illness, you should visit the following departments:

**Preferred Department: Psychiatry**

This is the most direct and appropriate department. Psychiatrists are medical doctors with prescribing authority, capable of:

- Conducting medical diagnosis: Assessing the condition based on professional diagnostic criteria (such as ICD-11 or DSM-5).
- Prescribing medication treatment: Such as antidepressants, anxiolytics, mood stabilizers, etc.
- Developing comprehensive treatment plans: Determining whether psychological treatment, physical therapy, etc., are needed.
- Managing severe mental disorders: Such as schizophrenia, bipolar disorder, major depressive disorder, etc.

In hospitals, this department may also be called:

- Clinical Psychology Department (but usually focuses on medication treatment, staffed by psychiatrists)
- Psychosomatic Medicine Department
- Mental Health Department
- Psychological Health Department

**Another Important Option: Psychology Department / Clinical Psychology Department**

This department is usually led by psychotherapists or clinical psychologists, who specialize in:

- Conducting psychological assessments: Assessing emotional, cognitive, and behavioral states through interviews and scales.
- Providing psychological counseling and therapy: Such as Cognitive Behavioral Therapy (CBT), psychoanalysis, humanistic therapy, etc.
- Conducting psychological counseling: For developmental issues and mild to moderate emotional distress.

Note: Psychotherapists do not have prescribing authority. They usually collaborate with psychiatrists and will recommend referral to psychiatry for patients requiring medication.

**How to Choose? A Simple Decision-Making Process:**

If you are unsure about the severity of the problem or symptoms have significantly affected your life:

- Priority is "Psychiatry." Doctors can make an authoritative diagnosis and determine the most suitable treatment plan (whether medication is needed, whether to combine with psychological treatment). This is the safest choice.

If the problem is mainly emotional distress, stress management, interpersonal relationships, etc., and you hope to first resolve it through conversation:

- You can try making an appointment for psychological treatment or counseling in the "Psychology Department."

For adolescents and children:

- You can prioritize hospitals with a Child and Adolescent Psychiatry Department or Child Psychology Department, where doctors and therapists are more experienced in handling children's developmental issues.

If accompanied by obvious physical discomfort:

- For example, insomnia, palpitations, gastrointestinal discomfort, chronic pain, etc., after ruling out organic diseases, you can choose the Psychosomatic Medicine Department.

In general hospitals:

- If you are unsure, you can first register for the "Psychiatry" department. Nurses at the hospital's triage desk will also provide the most accurate guidance based on your description.

**Important Notes:**

- Do not be afraid to seek medical help: Mental health problems are like colds and fevers—they are illnesses, and seeking professional help is a brave and correct choice.
- Initial consultation: During the first visit, the doctor will conduct a detailed interview and may ask you to fill out some assessment scales. Please truthfully describe your feelings and experiences.
- Emergency situations: If you have strong thoughts or behaviors of harming yourself or others, immediately go to the emergency department of a general hospital for urgent intervention, or call 110 or 120 for help.

**Summary: When in doubt, directly choosing psychiatry is the most common and safe option.**

**Q10. How to Distinguish Between Mental Illness and Other Physical Illnesses?**

This is a very important and professional medical question. Distinguishing between mental illness (psychological disorder) and other physical illnesses (somatic illnesses) is a core part of clinical diagnosis, and it can sometimes be very complex because symptoms of the two may overlap or interact with each other.

The core difference is: Mental illness mainly originates from brain dysfunction, manifested as abnormalities in thinking, emotions, and behavior; while physical illness originates from pathological changes in other organs and systems of the body.

The following is a systematic method and key points for distinction:

**I. Core Differentiation Dimensions**

| **Differentiation Dimension** | **Mental Illness (Psychological Disorder)** | **Physical Illness (Somatic Illness)** |
| --- | --- | --- |
| Main Origin | Brain function, psychological processes, social environmental factors | Infection, injury, degeneration, genetics, immune system abnormalities, etc. |
| Core Symptoms | Emotional disorders (e.g., persistent low mood, excessive elevation), thinking abnormalities (e.g., delusions, obsessions), cognitive deficits (e.g., memory, attention decline), behavioral disorders (e.g., hallucinations, social withdrawal) | Pain, fever, swelling, numbness, fatigue, organ dysfunction (e.g., palpitations, cough, diarrhea) |
| Objective Examinations | Usually lack specific biological markers. Brain CT/MRI, blood tests, etc., are mostly normal. Diagnosis mainly relies on clinical symptom assessment. | Usually have objective evidence. For example, imaging findings of tumors, elevated infection indicators in blood tests, abnormal electrocardiogram, etc. |
| Symptom Manifestation | Symptoms are often subjective and may fluctuate due to environment, emotions, and psychological suggestion. | Symptoms are more objective and stable, related to specific physiological activities or body positions. |
| Social Function | Impact on social and occupational functions is often very prominent and direct. | May first affect physiological functions, and only affect social functions when severe. |

**II. Key Diagnostic and Differential Process ("Rule Out" Principle)**

Clinically, when a person presents with unexplained symptoms, the primary task of doctors (especially psychiatrists) is not to directly diagnose a mental illness, but to follow the "exclusion principle":

**Comprehensive Physical Examination and Medical History Collection:**

- Doctors will conduct a detailed inquiry about the medical history, including when symptoms started, how they changed, and any triggering factors.
- Perform a comprehensive physical examination, measuring basic vital signs such as blood pressure and heart rate.

**Laboratory and Imaging Examinations:**

This is the most critical step in distinguishing the two. Doctors will order a series of examinations to "rule out" the possibility of somatic illness.

Common examinations include:

- Blood tests: Routine blood test, liver and kidney function, thyroid function, vitamin levels (such as B12), electrolytes, blood glucose, hormone levels, autoantibodies, etc.
- Imaging examinations: Brain CT or MRI (to rule out tumors, strokes, cerebral atrophy, etc.), electrocardiogram (to rule out heart problems).
- Others: Electroencephalogram (EEG) to rule out epilepsy, etc.

**Examples:**

- Hypothyroidism can cause extreme fatigue and depressive mood, resembling depression.
- Brain tumors can cause personality changes, hallucinations, or cognitive decline, resembling schizophrenia or dementia.
- Arrhythmia can trigger palpitations, shortness of breath, and a sense of impending death, resembling panic attacks.

**Specialist Assessment and Multidisciplinary Consultation:**

- If initial examinations reveal abnormalities, refer to the corresponding specialist (such as neurology, cardiology, endocrinology).
- If all somatic examinations are normal but symptoms persist, a diagnosis of mental health problems is more likely, and referral to a psychiatrist or psychotherapist is recommended.

**Psychiatric Assessment:**

Psychiatrists will systematically assess the patient's emotional, thinking, and behavioral patterns using structured clinical interviews and standardized psychological assessment scales (such as PHQ-9 for depression, GAD-7 for anxiety), and finally make a diagnosis based on diagnostic criteria (such as DSM-5 or ICD-11).

**III. "Gray Areas" and Comorbidity to Be Alert To**

**Psychosomatic Diseases:**

This is the most typical overlapping area. Psychological stress or emotional problems lead to real somatic symptoms. For example:

- Chronic anxiety and stress may trigger irritable bowel syndrome (IBS), tension headaches, hypertension, or eczema.

Such diseases have objective manifestations of somatic symptoms, and their occurrence and development are closely related to psychological factors.

**Somatic Symptom Disorder:**

Patients have very distressing and prominent somatic symptoms (such as pain, fatigue), but these symptoms cannot be fully explained by known somatic illnesses.

Patients are not "pretending"; they truly experience pain, but the root cause is highly related to psychological factors.

**Comorbidity:**

A person can completely have both somatic and mental illnesses, and the two interact with each other.

For example: A cancer patient (somatic illness) is likely to develop depression (mental illness) at the same time; while long-term depression also increases the risk of cardiovascular disease.

**Summary and Core Recommendations**

If you or someone around you experiences discomfort symptoms, follow this path:

1. First see a general practitioner or internist: Conduct a comprehensive physical examination to rule out all possible somatic illnesses. This is an essential first step; do not arbitrarily conclude it is a "psychological problem" and delay treatment.
2. Follow the doctor's advice for screening: Actively cooperate with blood tests, imaging examinations, etc.
3. If all somatic examinations are normal but symptoms are still distressing, take these normal examination reports to consult a psychiatrist. The psychiatrist will conduct a professional psychiatric assessment based on all information.
4. Maintain an open mind: Understand that the mind and body are closely connected as a whole. Both somatic and mental illnesses are real medical problems that need to be faced up to and treated scientifically.

Ultimately, distinguishing between mental illness and physical illness is a rigorous clinical process that requires medical professionals to make an accurate judgment with the help of objective examinations and personal assessments. Self-diagnosis is dangerous and unreliable.

**How to Distinguish Between Mental Illness and Physical Illness**

**Core Definition**

- Mental illness: Brain dysfunction
- Physical illness: Pathological changes in organ systems

**Differentiation Dimensions**

| **Origin** | **Mental: Psychology, society, brain function** | **Physical: Infection, injury, degeneration, genetics** |
| --- | --- | --- |
| Core Symptoms | Mental: Abnormalities in emotions, thinking, cognition, behavior | Physical: Pain, fever, swelling, dysfunction |
| Objective Examinations | Mental: Usually no specific biological markers | Physical: Usually have objective evidence (imaging, blood indicators) |
| Symptom Characteristics | Mental: Subjective, prone to fluctuation | Physical: Objective, relatively stable |

**Diagnostic Process (Exclusion Method)**

1. Comprehensive physical examination and medical history collection
2. Laboratory and imaging examinations
   - Blood tests (thyroid function, vitamin levels, etc.)
   - Brain CT/MRI
   - Electrocardiogram
   - Electroencephalogram
3. Specialist assessment and consultation
   - Abnormal findings: Refer to corresponding specialist
   - No abnormalities: Refer to psychiatry
4. Psychiatric assessment
   - Clinical interview
   - Standardized scales
   - Based on diagnostic criteria (DSM-5/ICD-11)

**Gray Areas and Comorbidity**

- Psychosomatic diseases
  - Psychological stress leads to real somatic symptoms
  - Examples: Irritable bowel syndrome, tension headaches
- Somatic symptom disorder
  - Distressing somatic symptoms cannot be explained by somatic illnesses
  - Root cause related to psychological factors
- Comorbidity
  - Having both types of diseases
  - Example: Cancer patient with comorbid depression

**Core Recommendations**

- Medical path
  - First see a general practitioner/internist
  - Complete comprehensive physical examination
  - Consult psychiatry with normal reports
- Maintain an open mind
- Important principles
  - Strictly prohibit self-diagnosis
  - Mind and body are interconnected
  - Both are medical problems requiring treatment

I have created a detailed mind map for you, clearly organizing the core differences, diagnostic processes, and complex situations that need attention between mental illness and physical illness. You can use this framework to systematically understand this important medical differentiation issue.

**Q11. Why Do Hospitals Require Multiple Examinations to Diagnose Mental Illness?**

Of course. Hospitals require multiple examinations to diagnose mental illness not because doctors are "uncertain" or engaging in "overtreatment," but on the contrary, it is a rigorous, scientific, and responsible medical process.

The core reason is: There is no single "gold standard" test (such as blood test or imaging) for diagnosing mental illness, and a comprehensive assessment must be conducted to rule out other possibilities and ensure diagnostic accuracy.

The following are the key reasons for requiring multiple detailed examinations:

**1. Ruling Out Somatic Illnesses (Organic Problems)**

Many physical illnesses first present with mental symptoms. If these conditions are not ruled out first, misdiagnosis may occur, leading to incorrect treatment and delaying the treatment of the actual illness.

Examples:

- Abnormal thyroid function (hyperthyroidism or hypothyroidism) can cause anxiety, depression, mood instability, and mental excitement.
- Brain tumors, infections, or epilepsy may cause hallucinations, delusions, and personality changes.
- Vitamin B12 deficiency, systemic lupus erythematosus, etc., can also mimic mental symptoms.

Therefore, the following are needed: Physical examination, blood tests (such as routine blood test, thyroid function, vitamin levels), electroencephalogram (EEG), CT or MRI brain scans, etc., to ensure there are no organic lesions in the brain and body.

**2. Conducting Differential Diagnosis**

Different mental illnesses may have similar symptoms, but their causes, courses, and treatment methods are completely different. Multiple examinations help doctors make accurate "differential diagnoses."

Examples:

- Symptoms of depressive episodes in depression and bipolar disorder are very similar. However, if a patient has unrecognized hypomanic episodes, misdiagnosing as depression and using antidepressants may induce mania and worsen the condition. This requires distinguishing through detailed medical history tracing and emotional assessment scales.
- Both anxiety disorders and early schizophrenia may present with suspicion and tension, but the treatment directions are completely different.

Therefore, the following are needed: Detailed clinical interviews, use of different psychological assessment scales (such as depression scales, anxiety scales, mania scales) to quantify and analyze symptoms.

**3. Assessing Comorbidity**

Many patients do not have only one mental illness at the same time; this situation is called "comorbidity." Comorbidity complicates the condition and requires simultaneous or prioritized treatment.

Example: A person may have both depression and anxiety disorder, or Attention-Deficit/Hyperactivity Disorder (ADHD) with Oppositional Defiant Disorder. Treating only one of them often yields unsatisfactory results.

Therefore, the following are needed: Comprehensive mental state examination and multiple assessments to depict a complete disease profile.

**4. Establishing Objective and Baseline Assessments**

Psychological tests and scales provide relatively objective indicators, which can reduce biases from subjective judgments.

Roles:

- Quantify severity: Convert subjective feelings into scores to assess the severity of the condition.
- Establish treatment baseline: Conduct assessments before treatment; rechecks later can be compared with this to objectively measure whether treatment is effective.
- Assess cognitive function: Use specialized neuropsychological tests (such as memory, attention, executive function tests) to assess whether brain cognitive function is impaired, which is important in dementia, brain injury, or schizophrenia.

**5. Developing Individualized Treatment Plans**

Accurate diagnosis is the premise of effective treatment. Different diagnoses correspond to completely different treatment strategies (medication treatment, psychological treatment, or a combination of both).

Examples:

- Diagnosis of depression may recommend antidepressants and Cognitive Behavioral Therapy (CBT).
- Diagnosis of schizophrenia mainly relies on antipsychotic medications.

Results of physical examinations (such as liver function, kidney function, electrocardiogram) also directly determine whether a doctor can safely use a certain medication and the dosage.

**Summary: An Analogy**

Diagnosing mental illness is like a detective solving a case:

- Clinical interview (listening to the patient and family members) is the testimony of eyewitnesses.
- Physical and laboratory examinations are forensic evidence at the scene, used to rule out other suspects (somatic illnesses).
- Psychological assessment scales are lie detectors and evidence analysis tools, used to verify testimony and quantify clues.
- Doctors are detectives who integrate all clues to make the most reasonable judgment (diagnosis) and develop a capture plan (treatment plan).

Therefore, multiple examinations are to be responsible for the patient, ensure diagnostic accuracy, avoid misdiagnosis and mistreatment, and lay the most scientific foundation for subsequent effective treatment. This is a complete process of comprehensively understanding an individual's physical, psychological, and social aspects.

**Q12. Is Psychological Assessment Helpful for Diagnosing Mental Illness?**

This is a very important and common question. Simply put: Psychological assessment is very helpful for diagnosing mental illness, but it cannot be used as the sole diagnostic tool.

Let me explain in detail the role, limitations, and correct use of psychological assessment.

**Core Role of Psychological Assessment (How It Helps Diagnosis)**

Psychological assessment is more like a powerful "radar" and "microscope," providing objective, quantitative data support for clinicians.

- Provide objective assessment basis: Through standardized questionnaires and scales (such as SCL-90, MMPI, HAMD, HAMA), subjective feelings of patients can be converted into comparable scores, reducing subjectivity of doctors' judgments based solely on personal experience.
- Screen and identify problems: Quickly assess whether a person has psychological distress and the possible direction of the distress (such as emotional problems, anxiety problems, personality traits). This is highly efficient in initial consultations.
- Quantify symptom severity: Not only determine "whether there are" symptoms but also "how severe" they are. For example, the Hamilton Depression Scale (HAMD) can quantitatively assess the severity of depressive symptoms, helping doctors determine whether it is mild, moderate, or severe depression.
- Assist in differential diagnosis: Some complex scales (such as the Minnesota Multiphasic Personality Inventory, MMPI) can help distinguish between different mental disorders. For example, helping distinguish between depression and anxiety disorder, or a certain personality disorder.
- Monitor treatment effectiveness: Repeating the same assessment before and after treatment can objectively assess whether treatment (such as medication or psychological treatment) is effective and whether symptoms have been alleviated through changes in scores.

**Limitations of Psychological Assessment (Why It Cannot Be Used Alone for Diagnosis)**

Despite being very useful, psychological assessment has inherent limitations and must never be separated from clinical diagnosis.

- Inability to make independent diagnoses: Diagnosis of mental illness is a complex clinical process that must be completed by trained psychiatrists or psychotherapists. Diagnosis requires considering, based on international standards (such as ICD-11 or DSM-5):
  - Medical history collection: Personal growth history, family history, stress events, etc.
  - Mental state examination: Doctors communicate face-to-face with patients, observing their speech, thinking, emotions, behavior, cognitive function, etc.
  - Physical examination and laboratory tests: Ruling out similar mental symptoms caused by somatic illnesses (such as abnormal thyroid function, brain lesions, etc.).
  - Psychological assessment results: As one of the important references.
- Possibility of "feigning" or "concealing": Patients may intentionally or unintentionally exaggerate symptoms (to seek more attention or diagnosis) or conceal symptoms (due to stigma or fear of being labeled), leading to distorted results.
- Cultural and understanding biases: Items of some scales may be affected by cultural background and educational level, and different people may have different understandings of questions, affecting the accuracy of answers.
- Risk of "labeling": If non-professionals misinterpret assessment results, it is easy to "label" people, causing unnecessary psychological burden and misunderstandings. For example, popular online tests such as "test if you have depression" are more entertaining than clinically valuable and must never be used as diagnostic basis.

**An Appropriate Analogy**

Comparing psychological assessment to a "thermometer" is very appropriate:

- A thermometer can tell you if you have a fever (screening function) and show how high the fever is (quantifying severity).
- But a thermometer cannot tell you whether the fever is caused by a cold, pneumonia, or other infections (differential diagnosis).
- The final diagnosis of the cause and development of a treatment plan (what medicine to prescribe, whether hospitalization is needed) must be determined by a doctor based on various information such as temperature, routine blood test, chest X-ray, and auscultation (comprehensive clinical diagnosis).

**Summary and Recommendations**

| **What Psychological Assessment Can Do ✅** | **What Psychological Assessment Cannot Do ❌** |
| --- | --- |
| Quickly screen and identify problems | Make final diagnoses independently |
| Provide objective quantitative data | Replace doctors' clinical interviews and observations |
| Assess symptom severity | Explain the underlying causes of symptoms |
| Assist in differential diagnosis | Prevent test-takers from feigning or concealing |
| Monitor treatment progress and effectiveness | Be used for self-diagnosis (especially online tests) |

**Recommendations for You:**

If you or someone you care about is facing psychological distress, professional psychological assessment is an excellent starting point for seeking help. You can:

- Visit the psychiatry (psychology) department of a regular hospital or a professional psychological counseling institution, where assessments are conducted under the guidance of professionals.
- Treat assessment results as important information for communication with doctors, not as a "diagnosis certificate."
- Do not take online tests and diagnose yourself, as this is likely to increase anxiety and lead to misjudgment.
- Trust the comprehensive judgment of doctors, who will combine your scale scores, personal narratives, and their professional observations to give the most prudent diagnosis and treatment plan.

In short, psychological assessment is an indispensable and important auxiliary tool in modern psychiatry, but it must be correctly used in a professional clinical environment and interpreted by qualified professionals.

**Q13. What to Do If Different Psychiatrists Give Different Diagnoses?**

This is a very important and common question. When different psychiatrists give different diagnoses, it can indeed be confusing, anxiety-provoking, and even helpless. Please remember that this does not necessarily mean one of the doctors is "wrong," but more likely reflects the complexity of mental health problems.

Handling this situation requires a systematic, calm approach. Here is a detailed step-by-step guide and recommendations:

**Why Do Diagnostic Discrepancies Occur?**

- Complexity of the condition itself: Many mental health symptoms overlap (for example, anxiety and depression often coexist), and patients' symptoms may evolve over time.
- Differences in information: Different doctors may obtain medical history information of varying depth and perspectives. Patients' expressions and states may also change at different times.
- Doctors' professional backgrounds and perspectives: Some doctors may focus more on biological factors, while others on psychosocial factors, which can affect the focus of diagnosis.
- Use of diagnostic criteria: Although there are global standards such as the International Classification of Diseases (ICD) and the Diagnostic and Statistical Manual of Mental Disorders (DSM), there is still room for subjective judgment in doctors' clinical understanding and application.

**How to Handle and Respond: Step-by-Step Guide**

**Step 1: Stay Calm and Avoid Panic**

First, do not immediately think your situation is "incurable" or "extremely complex." Diagnostic discrepancies are not uncommon in the initial stage of psychiatric consultation; this is a process of clarifying the problem, not the end.

**Step 2: Systematically Organize Information**

Collate the information you received from each doctor:

- What are the diagnostic names? Record them accurately.
- What are the main bases of the doctors? What symptoms, experiences, or manifestations of yours did they emphasize?
- What are the treatment recommendations? Do they suggest medication, psychological treatment, or a combination of both? What are the differences in the types and dosages of medications?

**Step 3: Seek a "Third Opinion" (Gold Standard)**

This is the most critical step. Find a reputable, experienced senior expert (usually a chief physician or professor level) in the field.

How to choose: You can look for them through the psychiatry or clinical psychology department of large Grade A tertiary hospitals or university-affiliated hospitals.

Prepare well: When seeing the third doctor, bring all your previous diagnostic records (but do not actively guide the doctor), and objectively and comprehensively state all your distress and medical history, allowing the expert to make an independent judgment based on the most complete information.

**Step 4: Prioritize "Symptoms and Function" Over "Labels"**

Sometimes, instead of obsessing over the label of "whether I have depression or bipolar disorder," it is better to focus on specific symptoms and solutions.

Discuss with the doctor: "Regardless of the diagnosis, my most distressing symptoms are insomnia, low mood, and inability to work. What methods can we prioritize to solve these problems?"

Function-oriented: The fundamental purpose of treatment is to improve social function and quality of life. A good treatment plan should directly address the specific difficulties you are facing.

**Step 5: Consider Conducting a More Comprehensive Assessment**

You can ask the doctor if a more comprehensive assessment is needed to provide objective reference:

- Psychological scales: Complete a series of standardized psychological assessment scales (such as MMPI, SCL-90) to provide quantitative basis for diagnosis.
- Physical examination: Rule out mental symptoms caused by thyroid function, neurological problems, or other somatic illnesses.
- Multidisciplinary consultation (MDT): In some large hospitals, you can apply for a joint consultation by multiple experts with different specialties (psychiatrists, psychotherapists, etc.) to reach a comprehensive conclusion.

**Step 6: Understand That Diagnosis Is a Dynamic Process**

Psychiatric diagnosis is not as clear-cut as taking an X-ray for a fracture. It is more like a dynamic process of hypothesis-verification-revision.

- The initial diagnosis is only a preliminary hypothesis.
- Doctors will verify this hypothesis based on treatment response. If the treatment effect is not good, it is precisely an important signal prompting doctors to re-evaluate and revise the diagnosis.

Therefore, the initial diagnostic discrepancy can be gradually clarified through subsequent treatment responses.

**Step 7: Establish a Trusting Relationship and Maintain Communication**

Choose a doctor with whom you feel most understood and can communicate smoothly, and establish a long-term, stable therapeutic relationship.

Attend regular follow-up visits and provide detailed feedback to the doctor:

- Feelings and side effects after taking medication.
- Minor changes in mood, sleep, energy, etc.
- New pressures or events in life.

This continuous feedback is the most valuable information for doctors to adjust diagnosis and treatment plans.

**Summary: Action Checklist**

- Don't panic: Accept that this is a common situation.
- Organize: Record all previous diagnoses and recommendations.
- Seek help: Consult a more senior expert for a "third opinion."
- Focus: Pay attention to specific symptoms and functional improvement rather than diagnostic labels.
- Assess: Conduct psychological scales and physical examinations if necessary.
- Be patient: Understand that diagnosis is a dynamic process that is continuously revised with treatment.
- Trust: Choose a doctor, establish a long-term cooperative relationship, and maintain open communication.

Ultimately, the most suitable diagnosis and treatment plan for you is the one that can most effectively alleviate your pain and help you restore social function. You yourself are the most important participant and decision-maker in this process.

I hope this detailed guide helps you clarify your thinking and find the right direction.

**Q14. Will Visiting a Psychiatrist Be Considered as Having a "Mental Illness"?**

This is a very important and common question. The simple and direct answer is: No. Visiting a psychiatrist does not mean being identified as having a "mental illness."

This is a common misunderstanding. I will explain the reasons, differences, and why seeking professional help is a wise and brave act in detail for you.

**Core Difference: Mental Health vs. Mental Illness**

We can understand this with an analogy:

- Visiting a general practitioner/internist: It may be for a cold, fever, physical examination, chronic disease management (such as hypertension, diabetes), or just consulting about health issues. You will not be automatically considered seriously ill just because you visited a doctor.
- Visiting a psychiatrist: Similarly, the reasons are very broad, not limited to severe mental illness.

The scope of psychiatric diagnosis and treatment is extremely wide, including but not limited to:

- Common psychological distress: Low mood, persistent anxiety, excessive stress, insomnia, inattention, interpersonal relationship problems, etc. These are experienced by most people at some stage in their lives, just like catching a cold.
- Psychological disorders: Such as depression, anxiety disorder, obsessive-compulsive disorder, post-traumatic stress disorder (PTSD), eating disorders, etc. These are medically diagnosed conditions that require professional intervention.
- Severe mental disorders: Such as schizophrenia, bipolar disorder, etc. This is what the public usually understands as "mental illness," but they account for only a small part of all mental health problems.

Therefore, visiting a psychiatrist is more like a "mental health check-up" or "seeing a doctor for an emotional cold," aiming to assess the current state, obtain professional guidance, and conduct effective intervention and treatment—not to "claim a mental illness label."

**Why Does This Misunderstanding and Stigma Exist?**

- Historical reasons: In the past, psychiatry was underdeveloped, and society had insufficient understanding of psychological problems, often equating people with mental health problems with negative labels such as "madmen" and "dangerous."
- Language confusion: The term "mental illness" in Chinese is often generalized and stigmatized, covering all categories from mild emotional problems to severe disorders, leading to conceptual confusion.
- Fear and ignorance: People tend to fear what they do not understand. Mental health problems are invisible and intangible, making them more difficult to understand than physical illnesses, thus more prone to misunderstanding and rejection.

**Professional Perspective: Diagnosis Is a Rigorous Medical Process**

Psychiatrists make diagnoses in accordance with strict international standards (such as ICD-11 or DSM-5). Visiting a doctor is only the beginning of this process.

- Assessment: Doctors will comprehensively understand your situation through detailed interviews, psychological assessments, etc.
- Differentiation: Distinguish between temporary emotional reactions, personality traits, or "disorders" that meet medical diagnostic criteria.
- Diagnosis: Only when all diagnostic criteria are met will the doctor give a clear medical diagnosis. Many people may not receive a "disease diagnosis" after visiting a doctor, but instead receive recommendations such as psychological counseling and lifestyle adjustments.

**How to Correctly View and Respond?**

- Change your mindset: Regard psychiatry as a department for maintaining "mental health," not for "mental illness." Caring for mental health is as important as caring for physical health and is a responsible act for yourself.
- Confidentiality principle: Your medical records and diagnosis are personal medical privacy, protected by law. Without your consent, doctors have no right to inform anyone (except in special cases such as the risk of self-harm or harm to others).
- Focus on self-growth: The ultimate purpose of visiting a doctor is to help you live better, improve quality of life and psychological resilience, not to define who you are.
- Respond to external doubts: If others are biased because you visited a psychiatrist, that is their cognitive limitation, not your problem. You do not need to explain your choice to everyone. You can simply respond: "I'm just asking a professional doctor for help with some sleep/emotional problems; this is very normal."

**Summary:**

Visiting a psychiatrist is a healthy behavior of caring for yourself and actively solving problems. It will never label you as "mentally ill" just because you walked into the clinic. The real "illness" is being ashamed to seek help and letting problems worsen. Daring to face problems and seek scientific help is a sign of strength and wisdom.

If you or someone around you is experiencing emotional distress, please bravely take this step. Professional help can bring great changes.

**Q15. What are the main treatment methods for mental illnesses?**

Treating mental illnesses is a complex process that usually requires comprehensive approaches. Modern psychiatry advocates the biopsychosocial medical model, meaning effective treatment plans often combine multiple interventions rather than relying on a single method.

The main treatment methods can be summarized into the following categories:

**I. Biomedical Treatment (Somatic Treatment)**

This type of treatment primarily targets the biological basis of the brain, regulating nervous system function through physical or chemical means.

**Pharmacotherapy**

- Antidepressants: Used to treat depression, anxiety disorders, etc. Common types include SSRIs (Selective Serotonin Reuptake Inhibitors) and SNRIs.
- Antipsychotics: Used to treat schizophrenia, manic episodes of bipolar disorder, etc., helping to eliminate or reduce symptoms such as hallucinations and delusions.
- Mood stabilizers: Mainly used to treat and prevent manic and depressive episodes of bipolar disorder.
- Anxiolytics: Used to quickly relieve anxiety symptoms, but long-term use is generally not recommended to avoid dependence.
- Sedative-hypnotics: Used to treat severe sleep disorders.

**Physical Therapy**

- Modified Electroconvulsive Therapy (MECT): A highly effective treatment, especially for severe, drug-refractory depression, manic episodes, and schizophrenia. Performed under anesthesia, it is safe and effective.
- Repetitive Transcranial Magnetic Stimulation (rTMS): A non-invasive brain stimulation technique that uses magnetic fields to stimulate specific brain regions. Commonly used to treat depression and anxiety disorders with minimal side effects.
- Vagus Nerve Stimulation (VNS) and Deep Brain Stimulation (DBS): Exploratory treatment methods mainly used for refractory cases.

**II. Psychotherapy ("Talk Therapy")**

Psychotherapy helps patients understand emotional, thinking, and behavioral patterns and learn new coping skills through establishing a professional relationship with a therapist.

| **Therapy Type** | **Core Idea** | **Main Applications** |
| --- | --- | --- |
| Cognitive Behavioral Therapy (CBT) | Our emotions and behaviors are influenced by cognitions (thoughts). Improve emotions and behaviors by identifying and changing irrational negative thoughts and beliefs. | Depression, anxiety disorders, obsessive-compulsive disorder, post-traumatic stress disorder, etc. |
| Psychoanalysis/Psychodynamic Therapy | Focus on unconscious conflicts and the impact of early childhood experiences on current behaviors and psychological issues. Achieve insight and change by exploring these deep-seated roots. | Long-term interpersonal difficulties, personality issues, chronic emotional problems |
| Interpersonal Therapy (IPT) | Focus on current interpersonal relationships and social functioning, believing that improving interpersonal relationships can alleviate psychological symptoms. | Depression (especially related to grief, role conflicts) |
| Dialectical Behavior Therapy (DBT) | A development of CBT that emphasizes the balance between acceptance and change. Teaches emotion regulation, distress tolerance, interpersonal effectiveness, and mindfulness skills. | Borderline personality disorder, long-term suicidal tendencies, self-harm behaviors |
| Family Therapy/Marital Therapy | Does not view the individual as the problem but intervenes with the family or marital system as a whole to improve communication and interaction patterns among members. | Family conflicts, child and adolescent behavioral problems, marital crises |

**III. Social Support and Rehabilitation Training**

This part focuses on restoring patients' social functioning and improving their quality of life, helping them reintegrate into society.

- Community support services: Including community rehabilitation centers, halfway houses, etc., providing transitional living support and skills training.
- Vocational rehabilitation: Offering vocational skills training, employment counseling, and supported employment to help patients regain and maintain employment.
- Peer support: "Peer support specialists" with similar rehabilitation experiences provide experience sharing, emotional support, and role modeling.
- Mental health education: Popularizing disease knowledge, treatment methods, and identification of relapse warning signs to patients and their families, enhancing treatment adherence and self-management capabilities.

**Treatment Principles and Trends**

- Individualized treatment: There is no "one-size-fits-all" optimal plan. Treatment plans need to be tailored to the patient's specific diagnosis, severity, personal preferences, physical condition, cultural background, etc.
- Comprehensive intervention: The combination of pharmacotherapy and psychotherapy is usually the most effective treatment model for most mental illnesses. Medications can quickly control severe symptoms, creating conditions for psychotherapy; psychotherapy addresses the root causes and prevents relapse.
- Long-term management: Many mental illnesses, like hypertension and diabetes, are chronic diseases that require long-term management rather than one-time cure. Adhering to treatment and regular follow-up are crucial.

The most important point: If you or someone around you is experiencing mental health distress, the first step is to seek professional help. You can go to the psychiatry department (mental health department) or psychological clinic of a regular hospital for a comprehensive evaluation and diagnosis, and jointly develop the most appropriate treatment plan with the doctor.

**Q16. Which is more effective: psychiatric medication or psychotherapy?**

This is a very important and common question. Simply put, for most mental health issues, pharmacotherapy and psychotherapy are not competing "either/or" options but complementary and mutually reinforcing collaborative approaches. Which is more effective depends on the specific type of illness, severity, personal circumstances, and different stages of treatment.

Below I will explain the relationship between the two in detail from several dimensions to help you understand.

**Core Difference: Different Mechanisms of Action**

- Pharmacotherapy: Primarily regulates brain function through chemical substances (medications) acting on the brain's neurotransmitter systems (such as serotonin, dopamine, norepinephrine), thereby quickly and effectively relieving severe symptoms.
  - Analogy: Extinguishing a large fire (acute symptoms). It can quickly control the fire to prevent further damage, but usually does not address the cause of the fire.
- Psychotherapy: Primarily helps patients understand the root causes of problems, change unhealthy thinking and behavioral patterns, learn new coping skills, heal trauma, and enhance psychological resilience through conversation, behavioral training, cognitive adjustment, etc.
  - Analogy: Investigating the cause of the fire, eliminating potential fire hazards, and installing a new fire protection system. It aims to solve the problem fundamentally and prevent future occurrences, but takes time and cannot extinguish a raging fire immediately.

**Which is More Effective? A Case-by-Case Discussion**

1. Severe mental disorders (e.g., schizophrenia, manic/depressive episodes of bipolar disorder, major depression with psychotic symptoms)
   - Pharmacotherapy is usually the first choice and foundation. In these cases, patients have severe brain dysfunction, may be accompanied by hallucinations, delusions, severe emotional instability, etc., with significantly impaired psychological function, making it difficult to conduct effective communication and learning. Medications can first stabilize the condition, creating conditions for receiving psychotherapy.
   - Psychotherapy is crucial after the condition stabilizes, helping patients understand the illness, improve medication adherence, address stigma, and rebuild social and vocational functioning.
2. Moderate depression, anxiety disorders (e.g., generalized anxiety disorder, panic disorder, obsessive-compulsive disorder, post-traumatic stress disorder (PTSD), etc.)
   - "Dual approach" (pharmacotherapy + psychotherapy) is usually the gold standard, with better results than either single therapy.
   - Medications can quickly reduce distressing symptoms such as insomnia, palpitations, and extreme low mood, allowing patients to have the energy and emotional foundation to engage in psychotherapy.
   - Psychotherapy (especially Cognitive Behavioral Therapy, CBT) helps patients identify and change negative thinking and behavioral patterns that cause distress, fundamentally reducing the risk of relapse.
3. Interpersonal problems, personality disorders, developmental issues, mild to moderate emotional distress
   - Psychotherapy is usually the first choice and core. These problems are often closely related to personal growth experiences, personality formation, and interpersonal relationship patterns, which medications cannot change. Through psychotherapy, patients can gain in-depth understanding and lasting personality growth.
4. From the perspective of treatment goals
   - Rapid symptom relief: Medications usually take effect faster (2-4 weeks).
   - Preventing relapse and achieving long-term change: The effects of psychotherapy are more lasting. The coping skills and thinking patterns learned belong to the patient and can be beneficial for a lifetime.

**Summary Comparison Table**

| **Dimension** | **Pharmacotherapy** | **Psychotherapy** |
| --- | --- | --- |
| Target of Action | Brain biological function | Thinking, emotions, behavioral patterns |
| Onset Speed | Relatively fast (weeks) | Relatively slow (months) |
| Mechanism of Action | "Chemical" regulation | "Education" and "training" |
| Treatment Goal | Treat the symptoms, control acute symptoms | Address the root causes, solve fundamental problems, prevent relapse |
| Side Effects | May have physical side effects | May cause temporary emotional discomfort |
| Durability of Effects | Effects may diminish after discontinuing medication | Effects are more lasting; acquired skills are lifelong |
| Applicability | Essential for moderate to severe mental disorders | Various psychological problems, especially mild to moderate and personality-level issues |

**Most Important Conclusions and Recommendations**

- It's not "or," but "and": Don't oppose the two. For many illnesses, the combination of pharmacotherapy and psychotherapy is the most effective and comprehensive plan.
- Follow professional evaluation: The final choice of treatment, or how to combine them, must be determined by a psychiatrist or clinical psychologist after a detailed evaluation. They will develop the most suitable personalized treatment plan based on your specific diagnosis, severity, personal history, and preferences.
- Individual differences: Everyone responds differently to medications and psychotherapy. It is necessary to continuously communicate with your therapist to adjust the plan.
- Patience and persistence: Neither treatment is immediate; it requires time and patience.

In short, just as heart disease patients need to take medication to control blood pressure and change their diet and exercise habits, mental health also requires dual maintenance of "hardware" (medications) and "software" (psychology). Seeking professional help and fully cooperating with your doctor and therapist is the best way to recovery.

**Q17. Is exercise effective for mental illnesses?**

Yes, exercise is very effective for mental illnesses. A large number of scientific studies have shown that regular physical activity is a powerful, low-cost auxiliary means to treat and improve various mental health problems.

It cannot "cure" all illnesses, but as a non-pharmacological intervention, its effects are widely recognized.

**How Does Exercise Improve Mental Health? (Mechanisms of Action)**

Exercise has a positive impact on the brain through various physiological and psychological pathways:

**Neurochemical Changes**

- Release of "feel-good hormones": Exercise promotes the brain to secrete endorphins, natural painkillers and mood elevators that produce a "runner's high."
- Regulation of neurotransmitters: It increases the levels of key neurotransmitters in the brain, such as serotonin (5-HT) (related to mood, sleep, appetite, and the target of many antidepressants), norepinephrine (related to attention and stress response), and dopamine (related to pleasure and motivation). This helps improve mood and reduce stress.

**Promotion of Brain Structural Changes**

- Increase in Brain-Derived Neurotrophic Factor (BDNF): Exercise has been shown to significantly increase BDNF levels. BDNF is like "fertilizer for the brain," promoting the growth, survival, and connection of neurons, especially in the hippocampus, a region related to memory and learning. Patients with depression and anxiety disorders often have a reduced hippocampal volume, and exercise can help repair and grow it.

**Reduction of Physical Stress Response**

- Exercise can lower the levels of stress hormones (such as cortisol) and help the body regulate the response of the hypothalamic-pituitary-adrenal (HPA) axis, making you more adaptable to stress.

**Psychological and Behavioral Benefits**

- Distraction: Exercise can shift your attention away from disturbing thoughts and worries.
- Enhancement of self-efficacy: Setting and achieving exercise goals (such as running 5 kilometers, completing a training session) brings a sense of accomplishment and control, boosting self-confidence.
- Provision of social opportunities: Participating in group sports or going to the gym can provide social interaction and reduce loneliness.

**Specific Effects on Different Mental Illnesses**

- Depression: The most significant effect. A large number of studies have shown that regular exercise (especially aerobic exercise) has an antidepressant effect comparable to pharmacotherapy or psychotherapy, and is particularly effective for mild to moderate depression. It is recommended as a supplementary core treatment in many clinical guidelines.
- Anxiety Disorders: Exercise is a natural remedy for relieving anxiety. It can consume excess energy and tension caused by anxiety, and reduce panic attacks by improving the body's tolerance to anxiety-like symptoms such as palpitations and shortness of breath.
- Attention Deficit/Hyperactivity Disorder (ADHD): Exercise can immediately increase the levels of dopamine and norepinephrine in the brain, which are lacking in ADHD patients. This helps improve concentration, mood, and executive function.
- Post-Traumatic Stress Disorder (PTSD): Exercise can help patients reconnect their minds and bodies, reduce hyperarousal symptoms, and serve as a healthy coping mechanism to process traumatic memories.
- Schizophrenia: As an auxiliary treatment, exercise can help improve patients' cognitive function, reduce negative symptoms (such as lack of motivation, emotional blunting), and counteract metabolic side effects (such as weight gain) that may be caused by antipsychotic medications.

**Scientific Consensus and Recommendations**

- Not a substitute for treatment: For moderate to severe mental illnesses, exercise should not replace pharmacotherapy or psychotherapy, but should be an important auxiliary means that works synergistically with the main treatment plan.
- Dosage matters: The World Health Organization and many health institutions recommend that adults engage in at least 150 minutes of moderate-intensity aerobic exercise (such as brisk walking, jogging, swimming) or 75 minutes of high-intensity aerobic exercise per week. Even short periods of activity (such as 10 minutes of walking) can bring immediate mood improvement.
- Best exercise methods: Both aerobic exercise and resistance training (strength training) have been proven effective. The best exercise is the one you enjoy and can stick to for a long time.

**Summary**

| **Benefit** | **Explanation** |
| --- | --- |
| ✅ Powerful auxiliary | An effective supplement to pharmacotherapy and psychotherapy, especially for depression and anxiety disorders |
| ✅ Improves mood | Directly elevates mood by releasing endorphins, serotonin, and other chemicals |
| ✅ Reduces stress and anxiety | Lowers cortisol levels and enhances the body's adaptability to stress responses |
| ✅ Enhances brain function | Promotes BDNF secretion, improving cognition, memory, and attention |
| ✅ Boosts self-confidence | Brings a sense of accomplishment and self-control through achieving exercise goals |
| ⚠️ Not a substitute | Cannot fully replace professional medical intervention for moderate to severe illnesses |

In short, exercise is a scientifically proven, powerful tool that can significantly improve mental health and reduce symptoms of mental illnesses. Incorporating it into your life is an extremely valuable investment in your brain and emotions.

If you are struggling with mental health issues, it is best to consult your doctor or mental health professional before starting any new exercise program.

**Exercise to Improve Mental Health: Practical Starter Guide**

Regular exercise is a powerful tool for improving mood, reducing anxiety, and coping with depression. This guide provides simple and actionable steps to get started.

**I. Core Principles**

- Consistency over intensity: Exercising 3-5 times a week for 20-30 minutes each time is more effective than occasional high-intensity exercise.
- Choose exercise you enjoy: This makes it easier to stick to.
- Progress gradually: Start with small goals and gradually increase duration and intensity.
- Focus on the process, not the outcome: Feel the physical sensations and emotional changes brought by exercise.

**II. Recommended Exercise Types**

**Immediate Anxiety Relief/Mood Enhancement**

- Brisk walking: Effective in 10-15 minutes.
- Jogging: Moderate-intensity running for about 20 minutes.
- Cycling: Outdoor or indoor.
- Dancing: Free movement to music.

**Best for Long-Term Effects**

- Swimming: 30 minutes of moderate intensity.
- Elliptical trainer/Rowing machine: Low-impact full-body exercise.
- Strength training: Dumbbells, resistance bands, or bodyweight exercises.
- Yoga/Tai Chi: Practices that combine body and breath.

**III. Specific Implementation Plans**

**Beginner Plan (Week 1)**

- Goal: Establish an exercise habit.
- Schedule: Every other day for 15-20 minutes.
- Example:
  - Monday: Brisk walking for 15 minutes.
  - Wednesday: Home yoga/stretching for 20 minutes.
  - Friday: Brisk walking for 20 minutes.

**Advanced Plan (After 2-4 Weeks)**

- Goal: Reach the WHO-recommended amount of exercise.
- Schedule: 5 times a week, 30 minutes of moderate intensity each time.
- Example:
  - Monday: Jogging for 30 minutes.
  - Tuesday: Strength training for 20 minutes.
  - Wednesday: Rest or light walking.
  - Thursday: Swimming for 30 minutes.
  - Friday: Yoga for 25 minutes.
  - Saturday: Cycling for 40 minutes.
  - Sunday: Rest.

**IV. Strategies to Overcome Obstacles**

**When Lacking Motivation**

- 5-minute commitment rule: Tell yourself "just 5 minutes"—usually, once you start, you'll continue.
- Find an exercise partner: Mutual supervision and encouragement.
- Track exercise achievements: Use an app or journal to record progress.

**When Mood is Particularly Low**

- Choose gentle exercise: Such as walking, light yoga.
- Split into short sessions: Three 10-minute exercise segments are equally effective.
- Focus on breathing: Direct attention to breathing rather than exercise performance.

**V. Safety Precautions**

- Consult a doctor: If you have health issues or haven't exercised in a long time, consult a doctor before starting.
- Warm-up and cool-down: 5 minutes of warm-up before each exercise and 5 minutes of relaxation and stretching afterward.
- Listen to your body: Stop if you feel pain or discomfort; don't push yourself.
- Stay hydrated: Drink an appropriate amount of water before, during, and after exercise.

**VI. Expected Effects Timeline**

- Immediate: Mood improvement and reduced anxiety immediately after exercise (lasting 2-4 hours).
- After 2 weeks: Improved sleep quality and reduced daily stress.
- After 4-6 weeks: Elevated overall mood baseline and enhanced ability to cope with stress.
- After 3 months: Increased self-confidence and reduced emotional fluctuations.

Remember: Every small start is a victory. Even a 5-minute walk is an important step toward better mental health.

**Q18. What types of medications are commonly used to treat mental illnesses?**

The treatment of mental illnesses mainly regulates the balance of neurotransmitters in the brain through medication intervention, thereby controlling symptoms and improving function. Below are the common types of medications classified according to their main treatment goals and chemical structures:

**1. Antipsychotics**

Mainly used to treat psychotic symptoms (such as hallucinations, delusions, disorganized thinking) in schizophrenia, delusional disorder, schizoaffective disorder, etc., and also commonly used for manic episodes of bipolar disorder.

**Typical Antipsychotics (First Generation):**

- Mechanism: Primarily block dopamine D₂ receptors.
- Representative medications: Chlorpromazine, Haloperidol, Perphenazine.
- Characteristics: Effective for positive symptoms but prone to extrapyramidal side effects (such as muscle stiffness, tremors, restlessness).

**Atypical Antipsychotics (Second Generation):**

- Mechanism: Simultaneously regulate dopamine and serotonin (5-HT) systems.
- Representative medications: Risperidone, Olanzapine, Quetiapine, Aripiprazole.
- Characteristics: Effective for both positive and negative symptoms (such as emotional blunting, social withdrawal), with relatively fewer extrapyramidal side effects, but may cause metabolic side effects (such as weight gain, increased blood sugar and lipids).

**2. Antidepressants**

Mainly used to treat depression, and also widely used for anxiety disorders, obsessive-compulsive disorder, post-traumatic stress disorder (PTSD), etc.

**Selective Serotonin Reuptake Inhibitors (SSRIs):**

- The most commonly used first-line antidepressants.
- Mechanism: Increase the concentration of serotonin in the brain.
- Representative medications: Fluoxetine, Paroxetine, Sertraline, Escitalopram.

**Serotonin-Norepinephrine Reuptake Inhibitors (SNRIs):**

- Mechanism: Simultaneously increase the concentrations of serotonin and norepinephrine.
- Representative medications: Venlafaxine, Duloxetine.

**Norepinephrine and Specific Serotonergic Antidepressants (NaSSAs):**

- Representative medication: Mirtazapine. Has good sedative and sleep-improving effects.

**Other Types:**

Also include tricyclic antidepressants (TCAs, such as Amitriptyline) and monoamine oxidase inhibitors (MAOIs, such as Moclobemide), which are now less commonly used as first-line treatments due to significant side effects or many contraindications.

**3. Mood Stabilizers**

Mainly used to treat and prevent manic and depressive episodes of bipolar disorder.

- Representative medications:
  - Lithium: A classic mood stabilizer with clear effects in preventing relapse, but requires regular monitoring of blood lithium levels.
  - Certain antiepileptic medications: Valproate, Carbamazepine, Lamotrigine.

**4. Anxiolytics**

Used to quickly relieve anxiety, panic attacks, and insomnia symptoms.

**Benzodiazepines:**

- Mechanism: Enhance the effect of GABA (an inhibitory neurotransmitter), with rapid onset of action.
- Representative medications: Diazepam, Lorazepam, Alprazolam.
- Characteristics: Long-term use is prone to tolerance and dependence; usually recommended for short-term or as-needed use.

**Non-Benzodiazepines:**

- Representative medication: Buspirone. Has a slower onset of action but no risk of dependence. SSRIs and SNRIs are also first-line choices for treating chronic anxiety.

**5. Sedative-Hypnotics**

Mainly used to treat insomnia.

- Representative medications:
  - Non-benzodiazepines: Zolpidem, Zopiclone, sometimes called "Z-drugs," which are more focused on hypnotic effects than traditional benzodiazepines.
  - Melatonin receptor agonists: Ramelteon, used for difficulty falling asleep.
  - Orexin receptor antagonists: Suvorexant, helps with falling asleep and maintaining sleep.

**Important Notes:**

- Individualized treatment: The choice of medication must be determined by a psychiatrist based on the patient's specific diagnosis, symptoms, physical condition, medication side effects, and personal response. Do not take medication or adjust the dosage on your own.
- Onset time: Antidepressants and mood stabilizers usually take 2-4 weeks or longer to show effects; patience and persistence are required.
- Comprehensive treatment: Pharmacotherapy is usually more effective when combined with psychotherapy (such as cognitive behavioral therapy).
- Side effect management: All psychiatric medications may have side effects; communicate with your doctor in a timely manner to adjust the plan or manage discomfort.

**Q19. How Long Does It Usually Take for Psychiatric Medications to Take Effect?**

This is an extremely important question and one that concerns many patients and their families the most.

Simply put: Most psychiatric medications typically take 2 to 4 weeks to start showing core therapeutic effects, but it may take longer—usually 6 to 8 weeks or more—to fully take effect and reach a stable state.

However, this timeline is not absolute and is influenced by various factors. The following diagram clearly illustrates the typical onset timelines and influencing factors for different types of medications:

flowchart TD

A[Start of Psychiatric Medication Administration] --> B[Week 1: Side effects may appear first]

B --> C[Weeks 1-2: Early improvements

Sleep, appetite, anxiety]

C --> D{Medication Type Judgment}

D -- Anxiolytics/Hypnotics --> E[Fast: Minutes to hours]

D -- Antidepressants --> F[Slow: Onset in 2-4 weeks, stabilization in 6-8 weeks]

D -- Antipsychotics --> G[Mixed: Sedative effect is fast

Core therapeutic effect takes 2-6 weeks]

E --> H[Achieve stable therapeutic effect]

F --> H

G --> H

I[Influencing Factors] --> J[Medication type and dosage]

I --> K[Individual physiological differences]

I --> L[Severity of illness and medical history]

I --> M[Concurrent psychological treatment]

J & K & L & M -.-> F

J & K & L & M -.-> G

Note that in the timeline above, some improvements (such as sleep and appetite) will occur earlier, while others (such as increased motivation and improved interest) are core therapeutic effects that require more time.

**Differences in Onset Timelines Among Different Types of Medications**

**Antidepressants**

These are the most typical "slow-acting" medications. They usually take 2-4 weeks to start improving core symptoms such as mood, energy, and loss of interest. In the first 1-2 weeks, some side effects may appear first, or there may only be slight improvements (such as better sleep or appetite). Do not discontinue the medication prematurely because of this.

**Anxiolytics**

- Benzodiazepines: Such as alprazolam and lorazepam, take effect very quickly, usually relieving acute anxiety within minutes to an hour. However, they are usually used for short-term intervention due to the risk of dependence.
- SSRI/SNRI antidepressants: Also used in the treatment of chronic anxiety disorders, their onset speed is the same as that of antidepressants, taking 2-4 weeks or longer.

**Antipsychotics**

- For "positive symptoms" such as excitement, restlessness, hallucinations, and delusions, the sedative effect may appear within a few days, but the core therapeutic effect usually takes 2-6 weeks.
- For "negative symptoms" such as apathy and withdrawal, the onset time may be longer.

**Mood Stabilizers**

Such as lithium and sodium valproate, used in the treatment of bipolar disorder. The onset also takes 1-2 weeks or longer, requiring gradual dosage increase and stable blood concentration to reach the therapeutic range.

**Why Does It Take So Long?**

Psychiatric medications do not directly block pain signals like painkillers. Their mechanism of action is more complex, involving the regulation of neurotransmitter (such as serotonin, norepinephrine, dopamine) levels in the brain and long-term adaptive changes in receptor function. This process is slow and takes time for the brain to gradually establish a new, healthier balance.

**What Should You Do During This Period?**

- Be patient and confident: Do not discontinue the medication on your own just because you do not see obvious effects within one or two weeks. Discontinuing prematurely is one of the most common reasons for treatment failure.
- Take the medication as prescribed: Strictly follow the dosage and schedule prescribed by your doctor. Do not adjust the dosage or discontinue the medication without authorization.
- Attend regular follow-up appointments: Regularly communicate with your doctor to assess the therapeutic effect and side effects, so that the treatment plan can be adjusted in a timely manner.
- Keep a record of changes: You can simply record your mood, sleep, and side effects every day to provide a reference for your doctor during follow-up.
- Combine with psychological treatment: Medication combined with psychological treatment often achieves a 1+1>2 effect.
- Communicate with your doctor about side effects: If the side effects are unbearable, inform your doctor in a timely manner. The doctor can adjust the dosage, change the medication, or take other measures to relieve them. Do not endure it.

Important Note: The above information is for popular science purposes only and cannot replace professional medical advice. Everyone's situation is unique. For specific onset timelines and treatment plans, always follow the guidance of your attending psychiatrist.

**Q20. Will Taking Psychiatric Medications Damage Brain Function?**

This is an extremely important and common question. Simply put, under the guidance of professional doctors, the standardized use of psychiatric medications is an important means to treat diseases and protect brain function, and the therapeutic benefits far outweigh the potential risks of side effects. On the contrary, untreated mental illness itself causes continuous and serious damage to brain function.

Let me explain the principles and relationships in detail for you.

**1. How Do Psychiatric Medications Work?**

Most psychiatric medications (such as antidepressants, anxiolytics, and antipsychotics) work by regulating the balance of neurotransmitters (such as serotonin, norepinephrine, and dopamine) in the brain. Mental illnesses are usually associated with imbalances in these neurotransmitters, and medications help restore them to normal levels, thereby relieving symptoms.

**2. Where Does the Concern About "Damaging the Brain" Come From?**

This concern mainly stems from the side effects of some medications, especially those that may occur in the early stages of treatment or when the dosage is inappropriate:

- Sedation, drowsiness, and difficulty concentrating: Many medications have a sedative effect, making people feel "slow in thinking" and drowsy.
- Temporary memory loss: Some patients may experience impairments in short-term memory or working memory.
- Emotional blunting: A few people may feel that their emotions have become flat and they are less able to experience joy, anger, sorrow, and joy vividly.

But these side effects are usually:

- Temporary: They mostly appear in the early stages of taking the medication and gradually decrease or disappear as the body adapts.
- Reversible: These effects mostly subside after adjusting the dosage, changing the medication, or discontinuing the medication.
- Manageable: Doctors can minimize these side effects by gradually adjusting the dosage, changing the medication type, etc.

**3. The Most Important Concept: The Illness Itself vs. Medication Treatment**

This is the core of understanding this issue. Untreated mental illness causes clear and continuous damage to the brain.

- Depression/Anxiety: Being in a state of high stress, anxiety, and depression for a long time will lead to persistently high levels of cortisol (a stress hormone) in the body. This will damage the hippocampus (a key area responsible for memory and learning), leading to reduced connections between brain cells and even shrinkage of brain volume, directly resulting in cognitive decline and memory loss.
- Schizophrenia/Bipolar Disorder: These illnesses themselves involve neurotransmitter system disorders and neuroinflammation, which can lead to progressive decline in brain function, including overall impairments in thinking, emotion, and cognitive function.

The role of psychiatric medications is actually to "apply the brakes" and prevent the illness itself from causing further damage to the brain. By controlling symptoms and reducing stress and mental distress, medications provide an environment for the brain to repair and restore function. Many studies have found that the volume of the hippocampus in patients with depression can be partially restored after successful treatment.

**4. How to Use Medications Safely to Maximize Benefits and Minimize Risks?**

- Follow medical advice and never take medications without authorization: This is the most fundamental principle. Psychiatrists will select the most appropriate medication and a "start with a low dosage and gradually increase" plan based on your specific diagnosis, physical condition, age, and other factors.
- Communicate openly and honestly: During follow-up appointments, inform your doctor of any discomfort or concerns you have while taking the medication. Do not discontinue the medication on your own because of temporary side effects, as this can lead to a recurrence of the illness.
- Attend regular follow-up evaluations: Doctors will not only assess whether the condition has improved but also pay attention to medication side effects and make timely adjustments. For patients taking medications for a long time, doctors may recommend regular necessary physical examinations (such as liver and kidney function tests, electrocardiograms, etc.).
- Combine with psychological treatment and lifestyle interventions: Medication is like a "crutch" that helps you stand up. Psychological treatment teaches you "how to walk," fundamentally changing negative thinking and behavioral patterns. Combining with a healthy diet, regular exercise, and adequate sleep can better promote brain recovery.

**Conclusion**

Taking psychiatric medications as prescribed will not damage the brain; instead, it protects your brain from more serious damage caused by the illness.

Analogizing mental illness to "hypertension" or "diabetes" helps with understanding: Hypertension continuously damages blood vessels and the heart, and taking antihypertensive medications is to prevent this damage, not because the medications themselves are damaging the body. Similarly, psychiatric medications are "targeted drugs" for treating brain diseases, and their goal is to restore health, not cause harm.

If you have any doubts about the medications you are taking or will take, the best approach is to have an open and honest discussion with your attending doctor, who will provide you with the most professional and personalized answers.

**Q21. How to Cope with Side Effects of Psychiatric Medications, Such as Weight Gain and Drowsiness?**

I fully understand your concern about the side effects of psychiatric medications. Weight gain and drowsiness are among the most common side effects of many psychiatric medications (such as antidepressants, antipsychotics, mood stabilizers, etc.), and they can indeed seriously affect quality of life and confidence in continuing treatment.

The good news is that these side effects are completely manageable. The key is not to discontinue or reduce the dosage of the medication on your own, but to adopt systematic strategies in collaboration with your doctor. Below are specific, actionable coping methods, divided into two parts: "medical strategies" and "lifestyle management."

**I. Medical Strategies (Collaborating with Your Doctor is the Primary and Most Critical Step)**

This is the fundamental way to cope with side effects. Your doctor has rich experience and various tools to help you.

**Be Open and Honest in Communication, and Keep Detailed Records**

- Do not hide anything: During follow-up appointments, clearly tell your doctor that you are experiencing problems such as drowsiness and weight gain. Describe in detail their severity and impact on your life (for example: "I feel uncontrollably sleepy for 2 hours every afternoon, which affects my work").
- Record changes: Regularly measure and record your weight. Keep track of your energy fluctuations every day. This data can provide valuable information for your doctor to adjust the plan.

**Adjust the Medication Regimen (Must Be Under the Guidance of a Doctor)**

- Adjust the dosage: Sometimes slightly reducing the dosage can significantly reduce side effects while maintaining the therapeutic effect.
- Adjust the time of administration: If the medication causes drowsiness, your doctor may recommend taking all or most of the dosage before bedtime, so that the sedative effect occurs at night, thereby reducing the impact on daytime activities.
- Change the medication: Different medications within the same category have different side effect profiles. For example, among antidepressants, escitalopram and sertraline may have a stronger activating effect than paroxetine (and are relatively less likely to cause drowsiness); bupropion hardly causes drowsiness and weight gain, and may even lead to weight loss. Your doctor may switch to another more suitable medication based on your situation.
- Combine medications: Doctors sometimes prescribe other medications to counteract side effects. For example:
  - For weight gain: Metformin (to improve insulin sensitivity) or topiramate may be prescribed in combination, but this requires strict evaluation.
  - For drowsiness: A small dose of modafinil or other wakefulness-promoting medications may be prescribed in combination, but this is not the preferred option.

**II. Lifestyle Management (Take Proactive Measures for Effective Support)**

While collaborating with your doctor, actively adjusting your lifestyle is a powerful weapon to cope with side effects.

**Management Strategies for "Weight Gain"**

Weight gain caused by psychiatric medications is mainly related to increased appetite and changes in metabolism.

**Dietary Adjustments (Quality Over Quantity)**

- Counteract increased appetite: Medications may make you feel hungry all the time. You can prepare some low-calorie, high-satiety foods, such as cucumbers, tomatoes, celery, apples, etc., and eat these first when you feel hungry.
- Optimize the diet structure:
  - Increase protein and fiber: Ensure that each meal contains an adequate amount of lean meat, fish, eggs, soy products, and a large amount of vegetables, which can provide long-lasting satiety.
  - Reduce refined carbohydrates and high-sugar foods: Eat less white rice, white bread, noodles, desserts, and sugary drinks. These foods can easily cause blood sugar fluctuations and exacerbate hunger.
- Eat regularly: Have three meals at fixed times to avoid overeating due to excessive hunger.
- Drink plenty of water: Sometimes the body confuses the signals of "thirst" and "hunger." Drinking a glass of water before meals helps control food intake.

**Exercise Interventions (Any Movement is Beneficial)**

- Start with low intensity: If you lack energy, start with a 15-20 minute walk every day. The key is to "move" and develop a habit.
- Combine aerobic and strength training:
  - Aerobic exercise (such as brisk walking, jogging, swimming, cycling): Helps burn calories.
  - Strength training (such as lifting dumbbells, squats, push-ups): Increases muscle mass. Muscle is the body's "engine." The more muscle you have, the higher your basal metabolic rate, and the less likely you are to gain weight.
- Use "micro-exercises": Walk around while on the phone, take the stairs instead of the elevator—these add up to significant effects.

**Management Strategies for "Drowsiness"**

**Optimize Sleep Hygiene**

- Maintain a regular schedule: Even if you sleep deeply at night due to the medication, try to get up at the same time every day (including weekends) to stabilize your biological clock.
- Daytime nap strategy: If you are really too sleepy to persist, you can take a short nap of 20-30 minutes. Taking a nap for too long or too late can affect nighttime sleep.
- Reduce pre-sleep stimulation: Avoid using mobile phones and computers 1 hour before bedtime, as blue light inhibits the secretion of melatonin.

**Manage Daytime Energy**

- Utilize natural light: Open the curtains after getting up in the morning, or spend more time outdoors during the day. Sunlight helps maintain alertness.
- Strategic caffeine use: If needed, you can drink a small cup of coffee or tea in the morning to stay awake. However, avoid drinking it in the afternoon or evening to prevent affecting nighttime sleep and forming a vicious cycle.
- Light activity: When you feel drowsy, stand up and stretch, or walk for a few minutes—this is more effective than sitting and fighting drowsiness.

**Core Principles and Attitude Adjustment**

- Patience and time: The body needs time to adapt. Some side effects (especially drowsiness) will gradually decrease after taking the medication for a few weeks.
- Weigh the pros and cons: Always remember that the core goal of treatment is to stabilize emotions and improve mental state. Compared with the pain caused by the illness itself, managing side effects is usually a matter of "choosing the lesser of two evils." A stable mental state is the foundation for you to have the ability to manage weight and energy.
- Seek support: Tell your family or friends about the challenges you are facing. Their understanding and encouragement are very important. You can also consider joining a support group to communicate with people who have similar experiences.

In summary, your coping path should be:

- Primary action: Immediately make an appointment with your attending doctor to discuss the side effect issues.
- Simultaneously: Start recording your weight and energy diary to provide information for your doctor.
- Proactive management: Try adjusting your diet and exercise habits, and optimizing your schedule according to the above suggestions.

Managing the side effects of psychiatric medications is a process that requires joint efforts between doctors and patients. Through active communication and scientific methods, you can definitely minimize the impact of side effects on your life while ensuring the therapeutic effect, and ultimately achieve the best state of physical and mental balance.

**Q22. Can Taking Psychiatric Medications Lead to Addiction?**

This is an extremely important and common question. The simple and direct answer is: When used as prescribed by a doctor, most modern psychiatric medications offer far greater therapeutic benefits than risks and will not lead to what people usually understand as "addiction" (i.e., compulsive drug-seeking behavior to pursue pleasure). However, some medications do carry a risk of "physical dependence" and need to be used and discontinued with caution under the guidance of a doctor.

To explain clearly, we need to distinguish between two key concepts: addiction and physical dependence.

- Addiction: This is a complex chronic brain disease characterized by compulsive drug-seeking behavior. Even if the drug causes serious negative consequences (such as health, legal, or social relationship problems), the patient cannot control the urge to seek and use the drug. Its core is psychological craving and behavioral loss of control.
- Physical dependence: This is a normal physiological state after the body adapts to a certain drug. If the drug is discontinued suddenly or the dosage is reduced rapidly, the body will experience a series of uncomfortable withdrawal symptoms. This does not mean that the patient will lose control and seek the drug.

Most psychiatric medications are intended for treatment, not pleasure. Their role is to help the brain's chemical balance return to normal, thereby relieving painful symptoms.

To help you fully understand, I have compiled a clear comparison table of the dependence risks of different types of psychiatric medications.

**Guide to Comparing Dependence Risks of Various Psychiatric Medications**

This table is designed to help you understand the characteristics and risks of different categories of psychiatric medications, but it cannot replace professional medical diagnosis and treatment advice.

| **Medication Category** | **Common Representative Drugs** | **Main Uses** | **Dependence Risk** | **Key Explanations and Precautions** |
| --- | --- | --- | --- | --- |
| Antidepressants | Sertraline, Escitalopram, Fluoxetine, Venlafaxine | Depression, anxiety disorders, etc. | Extremely low | Almost no addictive potential. Onset is slow (2-4 weeks). When discontinuing, the dosage should be gradually reduced over several weeks to avoid "discontinuation syndrome" (such as dizziness, insomnia, irritability). This is physiological adaptation, not addiction. |
| Antipsychotics | Olanzapine, Risperidone, Quetiapine, Aripiprazole | Schizophrenia, bipolar disorder, etc. | None | No addictive potential. Does not produce euphoria. Must be taken strictly as prescribed. Sudden discontinuation may lead to a recurrence of the illness. |
| Mood Stabilizers | Lithium, Sodium Valproate, Lamotrigine | Bipolar disorder | None | No addictive potential. They are the cornerstone medications for managing severe mood swings and require regular monitoring of blood drug concentrations. |
| Sedative-Hypnotics | Zolpidem, Zopiclone | Insomnia | Moderate-high | High risk of dependence. Usually recommended for short-term use (no more than 2-4 weeks). Long-term use is prone to tolerance (needing a larger dosage to be effective) and physical dependence, with obvious withdrawal reactions when discontinued. Must be used under the guidance of a doctor. |
| Benzodiazepines | Diazepam, Alprazolam, Lorazepam | Anxiety, insomnia, epilepsy | High | High risk of addiction and dependence. They are effective weapons for quickly relieving acute anxiety and insomnia, but are extremely prone to tolerance and dependence. Long-term and excessive use is strictly prohibited. Must be used for a short period under the strict guidance of a doctor and discontinued gradually. |
| Stimulants | Methylphenidate, Amphetamine | Attention Deficit Hyperactivity Disorder (ADHD) | Moderate | High risk of abuse when used for non-medical purposes. However, in patients with ADHD, such medications can help the brain return to normal function, thereby reducing their risk of addiction in the future. Must be prescribed and monitored by a specialist. |

**Core Conclusions and Recommendations**

- Treatment ≠ Addiction: The vast majority of psychiatric medications (such as antidepressants and antipsychotics) are designed to correct imbalances in brain function and are not addictive in themselves.
- Doctor-patient communication is key: Whether to use, how to use, and how to discontinue the medication must be strictly followed as per the doctor's advice. Doctors will formulate the safest and most effective treatment plan based on your specific illness, physical condition, and medication characteristics.
- Be wary of high-risk medications: For sedative-hypnotics such as benzodiazepines and "Z-drugs," be aware of their dependence risks and discuss the duration of use and discontinuation plan with your doctor clearly.
- Do not discontinue the medication on your own: Sudden discontinuation of any medication can cause problems (recurrence of the illness or withdrawal reactions). Dosage reduction and discontinuation must be carried out gradually under the guidance of a doctor.

If you have any doubts about the medications you are taking or will take, the best approach is to communicate all your concerns openly and honestly with your attending doctor.

I have created a detailed comparison guide for you, clearly listing the dependence risks, common representative drugs, and key precautions of different categories of psychiatric medications.

In summary, please remember:

Taking psychiatric medications to treat illnesses under the guidance of a professional doctor usually does not lead to "addiction." The purpose of doctors prescribing these prescription drugs is completely different from that of drugs. Open and transparent communication between doctors and patients is the greatest guarantee for safe and effective treatment. If you or your family have any concerns about medication use, be sure to raise them with your doctor first.

**Q23. What is Modified Electroconvulsive Therapy (MECT)?**

Modified Electroconvulsive Therapy (MECT) is a method of treating mental illness by stimulating the brain with brief, appropriate pulses of electric current under the support of modern anesthesia technology, causing epileptiform discharges in the patient's central nervous system. It is called "modified" (or "without convulsions") because muscle relaxants are administered before treatment, preventing the severe generalized convulsions common in traditional electroconvulsive therapy, which greatly improves the safety and comfort of the treatment.

**Core Characteristics of MECT:**

- Anesthesia and muscle relaxation: Before treatment, an anesthesiologist administers intravenous general anesthesia and injects muscle relaxants. The patient will complete the treatment while asleep, without pain or memory throughout the process, and avoid physical injuries such as fractures and joint dislocations that may be caused by limb convulsions.
- Precise electrical stimulation: Advanced electroconvulsive therapy instruments are used to output short-pulse, low-energy electrical currents, resulting in more precise stimulation and less impact on cognitive function compared to traditional methods.
- Significant and rapid therapeutic effect: For severe mental disorders such as severe depression, manic episodes, and schizophrenia, especially in patients with suicidal tendencies, refusal to eat, catatonia, or extreme excitement and restlessness, MECT can often produce faster and more effective therapeutic effects than medications.

**Main Indications Include:**

- Severe depression, especially in patients with strong suicidal ideation or behavior, intractable insomnia, or refusal to eat.
- Manic or depressive episodes of bipolar disorder.
- Schizophrenia, especially in catatonic type or patients with obvious positive symptoms.
- Others: Such as intractable obsessive-compulsive disorder, Parkinson's disease, neuroleptic malignant syndrome, etc.

**Brief Description of the Treatment Process:**

- Pre-treatment evaluation: A comprehensive physical examination (including electrocardiogram, electroencephalogram, blood tests, etc.) and anesthesia evaluation are performed to rule out contraindications.
- Treatment process (takes about 10-15 minutes):
  - The patient lies down, and vital sign monitoring equipment is connected.
  - Intravenous anesthetics and muscle relaxants are administered.
  - After the patient falls asleep and the muscles are completely relaxed, the doctor applies electrical current through electrodes placed on the head.
  - The treatment is completed when the instrument detects that the brain has produced an effective epileptiform discharge (lasting only a few tens of seconds).
- Recovery period: After the treatment, the patient will wake up in the recovery room, similar to waking up from general anesthesia surgery. Temporary confusion, headache, muscle soreness, or difficulty with recent memory may occur, but these side effects are usually temporary and will gradually recover within a few days to weeks.

**Contraindications:**

There are relatively few absolute contraindications, mainly including increased intracranial pressure, recent stroke, intracranial hemorrhage, unstable cardiovascular disease, etc. Relative contraindications require careful risk-benefit assessment by doctors.

In summary, MECT is a safe, effective, and technically mature physical therapy method that has become an important weapon in the modern psychiatric treatment of severe mental disorders. It has shed the terrifying image of early electroconvulsive therapy and, under strict operating standards, can bring hope of rebirth to many critically ill patients.

If you or your family are considering this treatment option, be sure to have an in-depth communication with a professional psychiatrist, who will make the most professional judgment and recommendations based on the specific situation.

**Q24. For Which Diseases is Transcranial Magnetic Stimulation (TMS) Therapy Mainly Indicated?**

Transcranial Magnetic Stimulation (TMS) is a non-invasive neuromodulation technology that uses time-varying magnetic fields to induce induced electric fields in the skull, thereby non-invasively stimulating cerebral cortical neurons to achieve therapeutic purposes.

Its main scope of application includes the following types of diseases:

**I. Main Indications Approved by the US FDA**

**Treatment-Resistant Depression (TRD)**

- Core application: For patients with severe depression who have not responded well to at least two or more antidepressant treatments, TMS is a recognized effective treatment. It usually involves high-frequency stimulation of the left dorsolateral prefrontal cortex, which is associated with emotion regulation.

**Obsessive-Compulsive Disorder (OCD)**

- The FDA has approved specific TMS devices and protocols for the treatment of adult OCD. The target area for stimulation is usually the brain region associated with impulse control and habitual behavior, such as the supplementary motor area or prefrontal cortex.

**Smoking Cessation (Nicotine Addiction)**

- Specific TMS protocols are approved as an adjunct to smoking cessation, reducing cravings by stimulating brain regions associated with addiction and reward circuits.

**Anxious Depression**

- TMS treatment is approved by the FDA for patients with both severe depression and anxiety symptoms.

**Migraine with Aura**

- Specific forms of TMS devices are approved by the FDA for the prevention and acute treatment of migraine with aura.

**II. Potential Applications Supported by a Large Body of Clinical Research (Some Have Been Approved in Other Countries or Regions)**

**Other Mental Health Disorders**

- Anxiety disorders: Such as generalized anxiety disorder, post-traumatic stress disorder (PTSD), etc.
- Schizophrenia: Mainly used to treat negative symptoms (such as apathy, social withdrawal) or positive symptoms (such as hallucinations) in patients.
- Bipolar disorder: During depressive episodes, it can be used as an alternative treatment option (with caution to avoid switching to mania).

**Neurological Diseases and Rehabilitation**

- Post-stroke rehabilitation: Used for the rehabilitation of motor function, aphasia, dysphagia, etc., by stimulating the damaged brain region or the contralateral hemisphere to promote neural functional reorganization.
- Chronic neuropathic pain: Such as fibromyalgia, central pain, etc.
- Parkinson's disease: Used to improve motor symptoms (such as bradykinesia, rigidity) and non-motor symptoms (such as depression, cognitive impairment).
- Alzheimer's disease: Mainly targeting mild cognitive impairment, aiming to improve memory and executive function.
- Tinnitus: Low-frequency TMS stimulation of the auditory cortex to reduce tinnitus symptoms.

**III. Important Precautions**

- Not a first-line treatment: TMS is usually considered only after medication and psychological treatment have been ineffective or poorly tolerated.
- Individualized protocols: Treatment effects vary from person to person. Professional doctors need to conduct a comprehensive evaluation to determine the appropriate stimulation target, frequency, and intensity.
- Contraindications: Patients with metal implants in the body (such as aneurysm clips, cochlear implants, deep brain stimulation electrodes) and those with a history of epilepsy are usually prohibited from receiving TMS treatment.

In summary, as a promising non-pharmaceutical physical therapy method, the application of TMS is constantly expanding from the field of mental illness to neurorehabilitation and other fields, but all treatments must be performed under the guidance of a doctor.

I hope the above information is helpful to you! If you want to know more details about TMS treatment for a specific disease, I am happy to provide a more in-depth explanation.

**Q25. Can I Discontinue Psychiatric Medications on My Own After Symptoms Improve?**

This is an extremely important and common question. My answer is: Absolutely not.

Discontinuing psychiatric medications on your own is a high-risk behavior that may lead to serious consequences.

Let me explain the reasons and the correct approach in detail for you.

**Why You Cannot Discontinue the Medication on Your Own?**

**Symptom Improvement ≠ Cure**

Feeling that your symptoms have improved is exactly a sign that the medication is working. This is like how blood pressure returns to normal after taking antihypertensive drugs, but it does not mean that hypertension has been "cured." After discontinuing the medication, blood pressure will rise again.

Mental illnesses (such as depression, anxiety disorders, bipolar disorder, schizophrenia, etc.) usually require adequate dosage and course of treatment to stabilize brain function. Discontinuing the medication prematurely can easily lead to a recurrence of the illness.

**Extremely High Risk of Recurrence**

Suddenly discontinuing the medication will cause the neurotransmitter (such as serotonin, norepinephrine, dopamine) levels in the brain that have been stabilized to become disrupted again.

After a recurrence of the illness, subsequent treatment may become more difficult, requiring a longer time and higher dosage to achieve the previous effect, which is known as "dosage escalation" or "treatment resistance."

**Terrible Withdrawal Reactions**

Many psychiatric medications, especially antidepressants (such as paroxetine, venlafaxine) and anxiolytics (such as alprazolam, clonazepam), can cause severe withdrawal reactions, commonly known as "discontinuation syndrome," if discontinued suddenly.

Common withdrawal reactions include: dizziness, nausea, vomiting, insomnia, anxiety, irritability, paresthesias (such as electric shock sensations), flu-like symptoms, etc. These physical discomforts are often mistaken for a "recurrence of the illness," leading to incorrect medication use due to greater panic.

**Rebound or Even Worsening of the Illness**

In some cases, suddenly discontinuing the medication may cause a rebound of the illness, with symptoms more severe than before treatment.

**What is the Correct Approach?**

The only correct approach is: Gradually reduce the dosage of the medication until discontinuation under the guidance of your attending doctor.

This process is called "medication tapering." Based on your specific situation, the doctor will:

- Evaluate the therapeutic effect: Determine whether your condition has truly reached a stable stage.
- Formulate a plan: Tailor a very slow, step-by-step dosage reduction plan for you, which may take several weeks or even months.
- Monitor closely: During the dosage reduction process, the doctor needs to closely observe whether you have withdrawal reactions or signs of recurrence, and adjust the plan in a timely manner.
- Provide support: The doctor will also guide you on how to consolidate the therapeutic effect through psychological treatment, lifestyle adjustments, etc., during this period to prevent recurrence.

**Summary**

| **Incorrect Approach: Discontinuing the Medication on Your Own** | **Correct Approach: Dosage Reduction Under Doctor's Guidance** |
| --- | --- |
| ❌ High risk, prone to recurrence | ✅ Safe, low risk of recurrence |
| ❌ May lead to severe withdrawal reactions | ✅ Gradual dosage reduction to minimize discomfort |
| ❌ May lead to treatment resistance, making future treatment more difficult | ✅ Protect the therapeutic effect and leave room for potential future needs |
| ❌ Face difficulties and risks alone | ✅ Have professional support, safe and secure |

Please remember: Starting medication requires a professional diagnosis from a doctor, and stopping medication also requires professional guidance from a doctor. Please share your idea of discontinuing the medication with your attending doctor and make a joint decision with them.

If you have concerns about the side effects of the current medication or other reasons for wanting to discontinue it, please first communicate with your doctor. They may be able to adjust the plan or switch to another medication instead of stopping it directly.

Your health and safety are the top priorities. Wish you a speedy recovery!

**Q26. How Long Do I Need to Continue Taking Psychiatric Medications After the Condition Stabilizes?**

This is an extremely important and common question. There is no "one-size-fits-all" answer to the duration of psychiatric medication use; it depends on various factors.

Simply put, after the condition stabilizes, the medication usually needs to be continued for a considerable period of time, and for some patients, long-term maintenance treatment may be required. Discontinuing the medication without authorization is the main risk factor for a recurrence of the illness.

Below are the key factors determining the duration of medication use and general guidelines:

**Key Factors Determining the Duration of Medication Use**

**Type and Severity of the Illness**

- Severe Major Depression (MDD): Usually requires a longer maintenance period, especially for patients with multiple recurrences.
- Bipolar Disorder: The vast majority of patients need to take mood stabilizers for long-term maintenance treatment to prevent manic and depressive episodes.
- Schizophrenia Spectrum Disorders: Long-term or even lifelong medication is usually recommended to control symptoms and prevent recurrence.
- Anxiety Disorders (such as panic disorder, obsessive-compulsive disorder): Also require adequate dosage and course of treatment, with a maintenance period that may last up to 1-2 years or longer.

**Number of Episodes**

- First episode: After the symptoms completely disappear (clinical recovery), the medication usually needs to be continued for at least 6 to 12 months (maintenance period) before the dosage can be reduced very slowly under the guidance of a doctor.
- Second episode: Maintenance treatment for at least 2-3 years is recommended.
- Three or more episodes: Long-term or even lifelong medication is usually recommended, as the risk of recurrence is very high.

**Patient's Individual Circumstances**

- Psychosocial factors: If the patient continues to be in a high-stress environment or lacks social support, longer medication treatment may be required.
- Response to medication and side effects: If the medication is effective and has mild side effects, both the patient and the doctor are more likely to opt for long-term maintenance.

**General Timeline Reference**

| **Illness Situation** | **Recommended Maintenance Treatment Duration** | **Notes** |
| --- | --- | --- |
| Depression (first episode) | At least 6-12 months | Calculated after the symptoms completely disappear |
| Depression (multiple episodes) | 2-3 years or longer | The more recurrences, the longer the required duration |
| Bipolar Disorder | Long-term maintenance, usually several years or lifelong | Mood stabilizers are the cornerstone |
| Schizophrenia | Long-term maintenance, usually lifelong | Significantly reduces the recurrence rate |
| Anxiety Disorders | 1-2 years or longer | Combined with psychological treatment for better results |

**Extremely Important Precautions**

- Do not reduce the dosage or discontinue the medication without authorization: This is the most dangerous behavior. Suddenly discontinuing the medication may lead to discontinuation reactions (such as dizziness, nausea, irritability) and rebound of the illness (rapid recurrence of symptoms, which may be more severe). Dosage reduction must be carried out very slowly under the guidance of a doctor.
- "Stable condition" does not mean "cured": Medication is like drugs for controlling hypertension; it keeps blood pressure normal, but once the medication is discontinued, blood pressure will rise again. Psychiatric medications also control symptoms by maintaining the balance of neurotransmitters in the brain. Current medical standards cannot "cure" most mental illnesses, but they can be effectively controlled with medications, allowing patients to resume normal lives.
- Maintain close communication with your attending doctor: This is the core of all decisions. The doctor will comprehensively judge when to start reducing the dosage, how slowly to reduce it, and whether it can be discontinued ultimately based on your specific situation (symptoms, side effects, living conditions).
- Attend regular follow-up appointments: Even if you feel well, you should attend regular follow-up appointments.
- Communicate openly and honestly: Timely feedback any changes to your doctor, including mood swings, side effects, and plans for pregnancy.

In summary, the treatment with psychiatric medications is a long-term process. Maintenance treatment after the condition stabilizes is a key stage to consolidate the therapeutic effect and prevent recurrence. How long you specifically need to take the medication must follow the professional advice of your attending doctor, which is the most responsible approach to your health.

**Q27. Can Mental Illness Be Completely Cured?**

This is an extremely important and complex question. Simply put, the answer is: It depends on the specific type of mental illness, but for the vast majority of mental illnesses, the goal of modern medicine is to achieve "clinical remission" and "functional recovery," rather than "eradication" or "never recurring" in the traditional sense.

We can understand this question from several levels:

**1. Different Definitions of "Cure"**

First, we need to distinguish between two concepts of "cure":

- Clinical Remission: Refers to the basic disappearance of the patient's symptoms, the recovery of social functions (such as work, study, interpersonal communication) to the pre-illness level, and the ability to live a normal life. This is the main and realistic goal of current psychiatric treatment.
- Complete Cure: Refers to the complete elimination of the cause, the illness never recurs, as if it had never been contracted. This is an extremely challenging goal for most chronic mental illnesses.

**2. Cure Possibilities for Different Mental Illnesses**

There are many types of mental illnesses, and their prognosis (i.e., the course and outcome of the illness) varies greatly.

| **Major Disease Category** | **Cure Possibility and Goal** | **Key Explanations** |
| --- | --- | --- |
| Anxiety Disorders, Depressive Disorders  (such as generalized anxiety disorder, depression, panic disorder) | Very high | These illnesses are usually considered highly treatable. Through medication, psychological treatment (such as cognitive-behavioral therapy, CBT), and other methods, the vast majority of patients can achieve clinical remission and resume normal lives. However, they have the potential to recur, so learning to manage emotions and stress and prevent recurrence is a long-term goal. |
| Trauma and Stress-Related Disorders  (such as PTSD) | Relatively high | Through professional psychological treatment (such as Eye Movement Desensitization and Reprocessing, EMDR), many patients can significantly reduce symptoms and even achieve recovery. The treatment process involves processing and integrating traumatic memories. |
| Schizophrenia, Bipolar Disorder | Controllable but requiring long-term management | These severe mental illnesses are currently difficult to "completely eradicate." Like hypertension and diabetes, they are chronic illnesses. However, through standardized, long-term medication treatment, the symptoms of the vast majority of patients can be effectively controlled, achieving stable conditions and maximizing the recovery of social functions. Discontinuing the medication can easily lead to recurrence. |
| Neurodevelopmental Disorders  (such as Autism Spectrum Disorder, ADHD) | Cannot be "cured," but can be significantly improved | These are inherent differences in brain function that cannot be "eliminated." However, through behavioral interventions, educational training, medication treatment, and other methods, core symptoms can be greatly improved, helping patients develop strengths, adapt to society, and have a high quality of life. |
| Substance Use Disorders  (Addiction) | Goal is sustained recovery | Addiction is regarded as a chronic, relapsing brain disease. "Cure" usually means achieving and maintaining abstinence and rebuilding a healthy lifestyle. This is a long-term process, and recurrence is a common phenomenon in the recovery process, not a failure of treatment. |

**3. Treatment Philosophy of Modern Psychiatry**

Modern psychiatry no longer simply pursues the abstract concept of "complete cure," but focuses more on:

- Symptom control: Minimizing or eliminating painful symptoms to the greatest extent.
- Functional recovery: Helping patients restore work, study, social, and self-care abilities.
- Improvement of quality of life: Allowing patients to feel happy, dignified, and valuable.
- Prevention of recurrence: Reducing the risk of recurrence through maintenance treatment and a healthy lifestyle.

**Conclusion and Core Points**

- Do not pursue "eradication," but pursue "recovery": Treat mental illness as a manageable chronic health condition, with the goal of returning to a normal, fulfilling life.
- Treatment is key: Most mental illnesses will not heal on their own without treatment and may even gradually worsen. Early detection and intervention yield the best results.
- Comprehensive treatment is the key: The most effective method usually combines medication, psychological treatment, social support (family, community), and a healthy lifestyle (exercise, diet, sleep).
- Recurrence does not mean failure: Just like catching a cold again, the recurrence of a mental illness does not mean that the treatment is ineffective or that the individual has failed. It is just a signal reminding you to pay attention to your mental health again and seek professional help.

In summary, although it is difficult to achieve "complete cure and never recurring" in the traditional sense for many mental illnesses, through scientific modern medical means, the vast majority of patients can effectively control symptoms, achieve clinical remission and functional recovery, and have a high-quality life. This is like wearing glasses to correct vision; although myopia still exists, the clear world has returned.

If you or someone you care about is experiencing difficulties, be sure to seek professional help from a psychiatrist or psychotherapist.

**Q28. Will Mental Illness Recur After Recovery?**

This is an extremely important and common question. The simple and direct answer is: It is possible, but it is not inevitable.

"Recovery" from a mental illness is more accurately defined as the effective control of symptoms, allowing the patient to resume normal social functions and daily life, which is called "clinical remission." However, unlike illnesses that confer lifelong immunity after one infection (such as chickenpox), most severe mental disorders (such as depression, bipolar disorder, schizophrenia, etc.) are chronic and prone to recurrence.

The risk of recurrence is real, but it can be significantly reduced through scientific, systematic management, achieving long-term stability.

**Main Risk Factors for Recurrence**

- Discontinuing or reducing the dosage of medication without authorization: This is the main cause of recurrence. Psychiatric medications require a sufficient cycle to take effect, consolidate, and maintain. Many patients mistakenly believe that the "illness has been cured" after feeling better and discontinue the medication without authorization, leading to a rebalancing of neurotransmitter levels in the brain and a rapid recurrence of symptoms.
- Stressful life events: Major stress is a common trigger for recurrence. For example, job changes, financial difficulties, interpersonal conflicts, the death of a loved one, etc., can all bring great psychological pressure to the patient, thereby triggering the illness.
- Weak social support system: Lack of understanding and support from family and friends, or living in a family environment full of criticism and hostility (medically known as a "high expressed emotion" environment), will greatly increase the risk of recurrence.
- Unhealthy lifestyle: Irregular work and rest, long-term staying up late, lack of exercise, heavy drinking, or drug abuse will all cause serious damage to brain function and emotional stability.
- Comorbidity with other physical illnesses: Certain chronic physical illnesses may also affect mood and mental state.

**How to Effectively Prevent Recurrence? (Core of Recovery Management)**

Preventing recurrence is an active management process that requires joint efforts of patients, family members, and the medical team.

**Adhere to Maintenance Treatment**

- Strictly follow medical advice: This is the cornerstone of preventing recurrence. Even if you feel well, you need to take the maintenance dosage of the medication for a sufficient course of treatment under the guidance of a doctor, usually for several years or longer.
- Attend regular follow-up appointments: Regularly communicate with your attending doctor, report your situation, so that the doctor can adjust the treatment plan in a timely manner and nip signs of recurrence in the bud.

**Receive Psychological Treatment**

Medication controls symptoms, while psychological treatment helps patients eliminate the root cause. Through methods such as cognitive-behavioral therapy (CBT), patients learn to identify and change negative thinking patterns, master emotional management and stress coping skills, and improve their ability to handle interpersonal relationships and setbacks.

**Establish a Healthy Lifestyle**

- Regular work and rest: Ensure adequate sleep and maintain a stable biological clock.
- Balanced diet: Pay attention to nutrition, which is crucial for brain health.
- Exercise regularly: Exercise is a natural "antidepressant" that can effectively relieve stress and improve mood.
- Avoid alcohol and drugs: These substances will seriously interfere with the therapeutic effect of medications and exacerbate the illness.

**Build a Strong Support System**

- Communicate openly and honestly with family and friends: Let them understand your illness and needs, and gain their understanding and support.
- Join a support group: Communicating with people who have similar experiences can reduce stigma and gain valuable experience and encouragement.

**Learn to Identify Early Warning Signs of Recurrence**

Recurrence usually does not happen suddenly, but there are traceable "clues." For example:

- Depression: Poor sleep for several consecutive days, loss of interest in previously enjoyed activities, easy fatigue, irritability.
- Bipolar disorder: Decreased need for sleep, abnormally high energy, reckless spending, racing thoughts.
- Schizophrenia: Reappearance of suspicion, hearing faint voices, sleep disturbances.

Work with your doctor to list your own "early warning sign checklist." Once these signs appear, immediately activate an emergency plan (such as contacting your doctor, strengthening support, etc.).

**Summary**

There is a possibility of recurrence of mental illness after recovery, but this is more like managing a chronic illness, such as hypertension or diabetes. Although "cure" cannot be guaranteed, through systematic maintenance treatment, a healthy lifestyle, strong psychological support, and keen awareness of your own state, the vast majority of patients can effectively control the condition, minimize the risk of recurrence, and have a high-quality life.

The most important thing is always to establish and maintain a trusting, long-term cooperative relationship with your psychiatrist. He/she is your best ally on the road to recovery.

**Q29. How to Prevent the Recurrence of Mental Illness?**

Preventing the recurrence of mental illness is a systematic process that requires joint efforts from medical, psychological, lifestyle, and social aspects. The core lies in establishing a solid "relapse prevention system."

I have compiled a comprehensive and practical prevention guide for you.

**Comprehensive Guide to Preventing the Recurrence of Mental Illness**

Preventing recurrence is a "protracted war" that requires multi-dimensional collaboration, with its core pillars as shown in the following figure:

flowchart TD

A[Mental Illness Relapse Prevention System] --> B1[Medical Pillar

Taking Medication as Prescribed]

A --> B2[Psychological Pillar

Continuous Psychological Treatment]

A --> B3[Lifestyle Pillar

Healthy Lifestyle]

A --> B4[Social Pillar

Strong Support System]

A --> B5[Early Warning Pillar

Identifying Relapse Signs]

B1 --> C1[Maintaining Brain Chemical Balance

Avoiding Unauthorized Discontinuation of Medication]

B2 --> C2[Improving Cognition and Coping Patterns

Managing Negative Emotions]

B3 --> C3[Regular Work and Rest, Balanced Diet

Moderate Exercise, Abstaining from Addictive Substances]

B4 --> C4[Open Communication, Joining Communities

Reducing Stigma]

B5 --> C5[Recording Mood Swings, Early Detection

Formulating Emergency Plans]

C1 & C2 & C3 & C4 & C5 --> D[Achieving Long-Term Stability and Recovery]

**I. Medical Pillar: Adhering to Standardized Treatment**

This is the most important and fundamental link, like the foundation of a building.

**Taking Medication as Prescribed, Never Adjusting Without Authorization**

- Importance: Medications help maintain the balance of neurotransmitters in the brain, which is key to controlling symptoms and preventing recurrence.
- Action Guide: Even if you feel well, you must adhere to the dosage prescribed by your doctor. Any plan to reduce or discontinue the medication must be carried out slowly under the guidance of a doctor.
- Coping with Side Effects: If you feel unwell after taking the medication, communicate with your doctor in a timely manner to find a solution (such as adjusting the dosage, changing the medication) instead of giving up directly.

**Attending Regular Follow-Up Appointments and Communicating Proactively**

- Importance: Doctors can assess your recovery progress and detect signs of problems in a timely manner.
- Action Guide: Treat follow-up appointments as necessary "health maintenance." Prepare the questions you want to discuss in advance and honestly report your mood, sleep, appetite, and any concerns to your doctor.

**II. Psychological Pillar: Learning Self-Awareness and Management**

**Continuing Psychological Treatment**

Medication treats the "symptoms," while psychological treatment treats the "root cause." Cognitive-behavioral therapy (CBT), mindfulness therapy, and other methods can help you:

- Identify and change negative thinking patterns.
- Learn more effective emotional regulation and stress coping skills.
- Improve your awareness of your own state (i.e., "insight").

**Learning to Identify "Early Warning Signs" of Recurrence**

Recurrence does not happen suddenly, but is a process. There are usually some precursor symptoms, which vary from person to person. Common signs include:

- Changes in sleep: Insomnia or excessive sleep for several consecutive days.
- Changes in mood: Unprovoked irritability, anxiety, or persistent low mood.
- Changes in cognition: Difficulty concentrating, memory loss, confused thinking.
- Changes in behavior: Unwillingness to go out, avoiding social interaction, lazy living, neglecting personal hygiene.
- Changes in the body: Significant increase or decrease in appetite, unexplained physical pain or discomfort.

Action Guide: Review your past recurrence experiences with your doctor or counselor to summarize your own "exclusive early warning sign checklist." Once these signs appear, immediately activate a coping mechanism.

**III. Lifestyle Pillar: Establishing a Healthy Lifestyle**

The body is the container of mental health, and a stable lifestyle is an emotional stabilizer.

- Maintain a regular schedule: Try to go to bed and get up at the same time every day, ensuring adequate sleep.
- Eat a balanced diet: Eat more fruits, vegetables, whole grains, and high-quality protein, avoiding high-sugar and high-oil foods. The intestines and stomach are known as the "second brain," and their health directly affects mood.
- Insist on moderate exercise: 30 minutes or more of aerobic exercise (such as brisk walking, running, swimming) 3-5 times a week can effectively relieve stress and improve mood.
- Avoid addictive substances: Strictly abstain from alcohol, drugs, and other addictive substances. They will seriously interfere with drug metabolism and brain function and are important triggers for recurrence.

**IV. Social Pillar: Building a Strong Support System**

**Establishing a Supportive Relationship Network**

- Communicate with family: Openly and honestly share your situation and needs with trusted family members, letting them know how to better help you.
- Make friends: Maintain a certain amount of social activity to avoid long-term isolation.
- Seek peer support: Join patient mutual assistance groups. Communicating with people who have similar experiences can provide great emotional support and practical advice, greatly reducing the sense of "being alone."

**Managing Stress and Balancing Work and Life**

Set reasonable expectations and goals to avoid putting too much pressure on yourself.

Learn and practice relaxation techniques, such as deep breathing, meditation, yoga, etc.

Cultivate hobbies to make life hopeful and enjoyable.

**V. Formulating a Personalized "Emergency Plan"**

Work with your doctor and supporters to formulate a written plan, clearly stating:

- What are the early warning signs?
- What can I do myself when the signs appear? (For example: strengthening rest, practicing mindfulness, contacting friends)
- Who should I contact when self-regulation is ineffective? (List the contact information of your psychiatrist, psychotherapist, and family members)
- When do I need to go to the hospital immediately?

Finally, please remember: Recurrence does not mean failure; it just indicates that your current recovery plan needs some adjustments. Like diabetes and hypertension, mental illness is also a chronic illness that requires long-term management. Be kind to yourself, seek help actively, and you are fully capable of becoming the master of your own health, preventing recurrence to the greatest extent, and enjoying a high-quality life.

This guide covers the core strategies for preventing recurrence. Please remember that seeking professional help is a sign of strength, not weakness. If you or someone you care about is experiencing difficulties, be sure to contact a psychiatrist or psychotherapist.

**Q30. Can Mental Illness Be Inherited by the Next Generation?**

This is an extremely important and complex question. Simply put, the answer is: Yes, but not in the simple, definite way that most people imagine.

The inheritance of mental illness is more like a "genetic predisposition" rather than direct inheritance like single-gene diseases (such as Huntington's disease). This means that the next generation inherits not the illness itself from their parents, but a higher risk of developing the illness. Whether the illness ultimately develops is also greatly influenced by acquired environment, personal experiences, and other factors.

Let me explain the scientific principles in detail for you:

**1. Core Concept: Heritability**

Scientists use "heritability" to measure the proportion of the contribution of genetic factors to the risk of a certain illness, with values ranging from 0% to 100%.

- High heritability (60%-80%): Such as schizophrenia, bipolar disorder, autism spectrum disorder. This means that genetic factors play a very important role in these illnesses. If an immediate family member (such as a parent or sibling) has these illnesses, an individual's risk of developing the illness is significantly higher than that of the general population.
- Moderate heritability (40%-50%): Such as major depression, anxiety disorders. Genetic and environmental factors are almost equally important.
- Low heritability: For some specific phobias or stress disorders directly caused by extreme trauma, genetic factors account for a small proportion.

Important Note: Even if the heritability is as high as 80%, it does not mean that the child has an 80% chance of developing the illness. It only means that 80% of the differences in the risk of this illness in the population can be attributed to genetic differences.

**2. Inheritance Pattern: Not a "Single Gene" Issue**

Mental illness is not inherited in a Mendelian manner (i.e., determined by a single gene). It is the result of the combined action of hundreds or even thousands of minor-effect genes, each contributing a little to the risk.

- Polygenic risk: The more risk gene variants a person carries, the higher their genetic predisposition.
- Gene-environment interaction: This is the most critical link. People with a high genetic risk may never develop the illness if they grow up in a stable, supportive environment. Conversely, if they experience major stress, trauma, drug abuse, and other environmental risk factors, they are likely to "trigger" the illness.

For example: Genetic risk can be imagined as a loaded gun, but environmental factors are the hand that pulls the trigger.

**3. Examples of Genetic Risk for Common Mental Illnesses**

| **Disease Name** | **Risk in the General Population** | **Risk in First-Degree Relatives** | **Explanations** |
| --- | --- | --- | --- |
| Autism Spectrum Disorder (ASD) | ~1.5% | Approximately 5-20% | Very high heritability, with significant differences in risk among siblings. |
| Schizophrenia | ~1% | Approximately 10% | If both parents are affected, the risk for children can reach ~50%. |
| Bipolar Disorder | ~2% | Approximately 5-10% | The inheritance pattern overlaps partially with that of schizophrenia. |
| Major Depression | ~10-15% | Approximately 2-3 times higher than the general population | Very common, with significant effects of environmental triggering factors. |
| Anxiety Disorders | ~10-20% | Approximately 2-4 times higher than the general population | Predisposition is obvious, but personal experiences have a great impact. |

**4. Beyond DNA Sequence: Epigenetics**

Even if the gene sequence does not change, environmental factors (such as prenatal stress, childhood trauma, nutrition) can affect the "switch" of genes through "epigenetic" mechanisms (such as DNA methylation), and these changes may even be inherited by the next generation. This explains why the impact of traumatic experiences may persist in families even without direct genetic mutations.

**Summary and Recommendations for You**

- Risk does not equal fate: Having a family history of mental illness does mean a higher risk, but it is by no means a death sentence. Strong protective factors (such as stable family relationships, good social support, healthy coping skills, and timely psychological intervention) can greatly offset the genetic risk.
- Attention and awareness: Understanding the family history is to increase vigilance, not panic. If you know there is a high risk, you can pay attention to the mental health signs of yourself or your children earlier and seek professional help in a timely manner when problems arise, which can greatly improve the prognosis.
- Consult a professional: If you are planning to start a family and are very worried about the genetic risk, you can consider consulting a genetic counselor or psychiatrist. Based on your specific family situation, they can provide a more personalized and scientific risk assessment and recommendations.

In summary, mental illness is the product of the complex interplay of biological (genetic), psychological, and social environmental factors. Genetics lays the foundation for possibility, while life experiences jointly write the final result.

I hope this explanation is helpful to you. If you have more specific questions about a particular disease, I am happy to provide more information.

**Q31. How Can Patients with Mental Illness Better Reintegrate into Society?**

This is an extremely important and complex question. The rehabilitation and social reintegration of patients with mental illness require a systematic support system, involving the joint efforts of the patients themselves, their families, the medical system, and the entire society.

The core is: Rehabilitation is not only the disappearance of symptoms, but also the reconstruction of social functions, the realization of self-worth, and living a dignified life.

Below is a multi-dimensional support framework for patients to better reintegrate into society:

**I. Individual Level: Laying a Solid Foundation for Reintegration**

**Adhere to Standardized Treatment and Rehabilitation: This is the cornerstone of all efforts. Including:**

- Take medication as prescribed: Maintain stable conditions and prevent recurrence.
- Attend regular follow-up appointments: Maintain communication with psychiatrists and adjust treatment plans in a timely manner.
- Actively participate in psychological treatment: Learn emotional management, stress coping, and interpersonal skills, and change negative cognitive patterns.

**Skill Training and Reconstruction:**

- Life skills training: Such as independent living, personal hygiene, financial management, cooking, etc.
- Social skills training: Learn how to start conversations, maintain topics, express needs, and refuse through role-playing, group therapy, etc.
- Vocational rehabilitation: Including vocational assessment, skill training, job search counseling, simulated work scenarios, etc., to prepare for returning to the workplace.

**Cultivate Insight and Self-Acceptance:**

- Objectively understand your own illness, recognize the precursors of recurrence, and take the initiative to seek help.
- Reduce stigma and accept that you are "a person who is ill" rather than "a failure."

**II. Family Level: Providing a Warm Rear Base**

**Provide Emotional Support and Understanding: The family is the most important source of support for patients. Family members need to learn about the illness, understand the patient's pain and difficulties, and give unconditional care and patience, rather than criticism, accusation, or overprotection.**

**Create a Stable Family Environment: Maintain a regular lifestyle, reduce family conflicts and major stressful events, and provide a safe, peaceful, and predictable living environment.**

**Become a "Collaborative Caregiver": Assist patients in managing medications, observing changes in their condition, encouraging them to participate in rehabilitation activities, and maintaining close communication with the medical team.**

**III. Social Support System: Building Bridges for Reintegration**

This is currently the weakest but most critical link.

**Community Rehabilitation Services (Core Bridges):**

- Day hospitals/rehabilitation centers: Provide transitional structured activities to help patients gradually adapt to the rhythm of life after leaving the hospital.
- Clubhouse model: An internationally recognized successful model. It emphasizes "members" rather than "patients," and rebuilds self-confidence, friendship, and work ability through joint work (such as gardening, preparing lunch, managing libraries), without mandatory treatment procedures.
- Supported employment: Such as "sheltered workshops" or "supported employment." Employment counselors will accompany patients to interviews and work, and provide intensive support in the early stages of work to help patients adapt to the work environment.
- Peer support: Peer supporters who have recovered well provide experience sharing, companionship, and role models for other patients, reducing loneliness.

**Policy and Legal Protection:**

- Anti-discrimination legislation: Protect patients from discrimination in employment, education, medical care, etc.
- Social welfare: Provide reasonable disability allowances and medical subsidies to ensure that patients have no worries during rehabilitation.
- Flexible employment policies: Encourage enterprises to hire people with mental disabilities and provide incentives such as tax breaks.

**Public Education and Social Atmosphere:**

- Anti-stigma publicity: Popularize mental health knowledge through the media, public welfare activities, etc., to make the public understand that mental illness is a treatable illness like hypertension and diabetes, and eliminate misunderstandings and fears.
- Build inclusive communities: Encourage community neighbors to accept rehabilitated individuals with a normal attitude and invite them to participate in community activities. A friendly smile and normal interaction are the best support.

**Summary and Key Points:**

- This is a "systematic project": It requires the collaboration of individuals, families, communities, and the government. The lack of any link may hinder the rehabilitation process.
- The process is gradual: Reintegrating into society does not happen overnight and may be full of repetitions and setbacks. It is necessary to take small steps, gradually expanding the scope of activities from the family to the community, and then to the workplace.
- The goal is "a quality life": The goal of reintegrating into society is not necessarily to find a high-paying job, but to be able to live a life with friends, activities, dignity, and hope according to one's own wishes and abilities.

Ultimately, a society friendly to people with mental disabilities is not only helping them, but also creating a more inclusive, supportive, and resilient future community for all of us.

**Q32. How to Cope with Discrimination and Gossip Against Patients with Mental Illness?**

Facing discrimination and gossip against patients with mental illness is a process that requires strategy, patience, and courage. This is not only about safeguarding the dignity of others, but also about striving to create a more inclusive society. Below is a detailed coping guide divided by levels and scenarios.

**I. When Discrimination and Gossip Occur: How to Respond Immediately**

**1. Stay Calm and Assess the Situation**

First, quickly judge:

- Who is the other person? A stranger, colleague, friend, or family member? The closeness of the relationship determines your coping strategy.
- Is the environment safe? In public places or situations with potential conflict risks, prioritize your own safety.
- Is the patient present? If so, your primary task is to support him/her, not to argue with the other person.

**2. Choose Your Response Strategy (From Easiest to Most Difficult)**

**Strategy A: Express Personal Feelings (Gentle and Effective)**

Suitable for people with whom you want to maintain a relationship, such as friends and colleagues.

Example phrases:

- "What you said makes me feel a little uncomfortable."
- "I know some people going through similar situations, and they are actually working very hard, so we may not know the whole story."
- "Shall we change the subject?" (Simply and directly draw the line)

**Strategy B: Raise Doubts and Trigger Thinking**

Suitable for situations where you want to correct the other person's views.

Example phrases:

- "Why do you think that way?" (Let the other person explain their prejudice)
- "Would you say the same if it were cancer or heart disease? Mental illness is also an illness."
- "What you said is a stereotype; the actual situation is much more complex."

**Strategy C: Directly Correct and Provide Facts**

Use when you have relevant knowledge and the situation is appropriate.

Example phrases:

- "Actually, depression isn't something you can just 'cheer up' from; it has a physical basis, just like having a fever when you have a cold."
- "Modern medicine has effective treatment plans for many mental illnesses; they are just receiving treatment."
- "Using words like 'crazy' or 'psycho' is disrespectful. We can directly say 'he is experiencing a mental health problem.'"

**Strategy D: Firmly Set Boundaries**

For malicious, repeated discriminatory remarks.

Example phrases:

- "This is a discriminatory remark, and we cannot accept it here."
- "I don't want to discuss this topic about his illness anymore."
- "Please stop."

**II. How to Support Patients with Mental Illness Who Are Discriminated Against**

If your friend or family member is the victim, your support is crucial.

**Stand by Their Side Immediately: A supportive look, a nod, or simply standing closer to them can convey great strength.**

**Communicate and Validate Afterwards:**

- Listen: "You must have felt very bad about what that person said, right?"
- Validate: "What he said is completely wrong; it's not your fault."
- Empower: "You handled it very well; I'm proud of you."

**Provide Practical Help: Ask them how they want to cope with such situations. "Next time something like this happens, what do you want me to do? Defend directly, or leave together?"**

**Encourage Seeking Professional Support: If discriminatory remarks cause significant distress to the patient, encourage him/her to talk to a psychotherapist or support group.**

**III. Long-Term Strategy: Reducing Discrimination at Its Root**

Changing attitudes takes time and requires sustained effort.

**Educate Yourself, Then Educate Others:**

- Take the initiative to learn about mental health knowledge and understand the truth about common illnesses (such as depression, anxiety disorders, bipolar disorder).
- Popularize this knowledge in a sharing rather than preachy way on appropriate occasions. "I recently read an article that 1 in 4 people..."

**Pay Attention to the Power of Language:**

- Stop using insulting words like "psycho" or "crazy" to describe others in your own life and around you.
- Use "person-first language": For example, say "a person with schizophrenia" instead of "a schizophrenic." Emphasize that he is first and foremost a "person," not a "patient."

**Share Personal Stories (If Comfortable):**

If you or someone close to you has relevant experiences, sharing stories is the most powerful weapon to break down prejudices. True stories can arouse empathy and turn abstract "illness" into a concrete "person."

"My good friend once suffered from severe anxiety, and during that time he..."

**Utilize Social Media and Resources:**

- Share popular science articles or promotional videos released by the World Health Organization (WHO) and local mental health associations.
- Recommend relevant books, documentaries, or podcasts.

**Support Relevant Organizations and Policies:**

- Pay attention to and support non-profit organizations that advocate for the rights of people with mental disorders.
- Advocate for the establishment of a more comprehensive mental health support system and anti-discrimination policies in the workplace and community.

**IV. Legal and Rights Paths**

If discriminatory behavior is serious enough to constitute workplace discrimination, denial of educational opportunities, or infringement of legal rights, know that this is illegal.

- Preserve evidence: Record the time, place, person, and remarks.
- Seek help: Complain and seek help from the company's human resources department, school management, or relevant legal aid agencies.
- Resort to legal means: Understand the "Disability Rights Protection Law" or "Mental Health Law" in your country or region. Many laws include severe mental illnesses in the scope of protection and prohibit discrimination based on this.

**V. Self-Care: Protect Your Own Energy**

Fighting prejudice is an emotionally draining task.

- You are not a savior: You cannot change everyone's mind. Choose your battles and do not exhaust yourself in every argument.
- Seek support: Communicate these experiences with like-minded friends and encourage each other.
- Do what you can: If you are not in a good state, it is completely okay to avoid conflicts temporarily and prioritize your own mental health.

In summary, coping with discrimination is like a multi-front battle: there are "frontal confrontations" to fight for dignity in the moment, and "behind-the-scenes work" to achieve long-term change through education and legislation. Every time you choose to speak up, even if it's just a simple "that's not right," you are contributing to reducing a little prejudice and increasing a little understanding.

**Q33. Can I Date Someone with a Mental Illness?**

This is an extremely important and complex question. The answer is: Yes, but it depends on the specific circumstances and requires more understanding, patience, and preparation than a typical relationship.

Simply answering "yes" or "no" is irresponsible. The key lies in how you perceive and handle the unique challenges in this relationship.

Below, I will analyze it in detail from several aspects to help you think more comprehensively.

**Core Premise: Respect and Destigmatization**

First, we must recognize that "mental illness" is a broad term covering everything from common conditions like depression and anxiety to more complex ones such as bipolar disorder and schizophrenia. Every individual is unique, and their condition, symptom severity, treatment status, and impact on life vary greatly. Labeling them simply as "mentally ill" and thus denying their right to be loved is one-sided and unfair.

The core of a successful romantic relationship has always been love, respect, trust, and communication. These elements are equally important—if not more so—for partners where one has a mental illness.

**Potential Challenges**

Dating someone with a mental illness may present challenges not typically encountered in ordinary relationships:

- Impact of Symptoms: The illness itself may affect the other person's mood, energy, cognition, and behavior. For example, a person with depression may experience prolonged low mood and loss of interest in activities; someone with anxiety may worry excessively or avoid social interactions. This can impact your daily interactions and shared activities.
- Communication Barriers: During symptom episodes, the other person may struggle to clearly express their feelings and needs, or may misinterpret your words and actions.
- Caregiver Burnout: You may unconsciously take on the role of "caregiver" rather than "partner." Long-term investment of significant emotional support and energy can leave you feeling exhausted, stressed, and even resentful.
- Social Stigma: You may face misunderstanding, prejudice, or discrimination from others.
- Relationship Unpredictability: Fluctuations in the illness can lead to ups and downs in the relationship, requiring greater adaptability and stability.

**Key Factors for Success**

Despite the challenges, many such couples still maintain healthy, fulfilling relationships. Success typically depends on the following key factors:

- Is the other person actively treating and managing their condition? This is the most crucial prerequisite. Do they acknowledge their situation? Are they willing to seek professional help (psychological counseling, medication)? Are they learning to manage their symptoms? A partner who faces the illness proactively and takes responsibility for themselves is the foundation of a healthy relationship.
- Your knowledge and understanding: Are you willing to learn about their illness? Understanding related symptoms, triggers, and coping methods can help reduce misunderstandings and enable you to provide better support. You will recognize that sometimes their reactions are due to "the illness" rather than "their true nature."
- Strong communication and boundary-setting:
  - Open Communication: Encourage honest discussions about feelings, needs, and fears. When the other person is in a good state, talk together about how they would like you to respond when symptoms arise, and what support is most helpful.
  - Clear Boundaries: You must be clear that you are a partner, not a therapist. Your support cannot replace professional treatment. At the same time, you need to protect your own mental health, clarify what you can provide, and what you cannot. Learn to say "no" and set aside time for rest and self-care.
- A strong support system: You cannot do this alone. Ensure the other person has their own therapist and support network (family, peer support groups). Similarly, you need your own support system—friends, family, or your own therapist—to process the stress and emotions accumulated in the relationship.
- Realistic expectations: Accept that the relationship will have ups and downs and will not always be easy. Are you prepared to face these challenges? Are your expectations realistic?

**Questions You Need to Ask Yourself**

Before deciding to start or continue such a relationship, honestly ask yourself:

- Do I love this person, or do I have a fantasy of "saving" them? (A savior mentality is harmful to both parties)
- Do I have sufficient emotional maturity, patience, and resilience to cope with potential challenges?
- Can I provide support without sacrificing my own mental health?
- Do I respect them as a complete person, not just focus on their illness?
- If their condition does not improve significantly in the future, can I still accept and continue to love them?

**Summary**

Can I date someone with a mental illness? — Yes, but it is a path that requires more courage, wisdom, and effort.

It is by no means easy, but it is by no means impossible. The final decision is yours, based on your love for this person, your assessment of the actual situation, and your own level of preparation.

The most important principle is: A good relationship should make both parties better. If this relationship leaves you feeling constantly exhausted, losing yourself, or hindering the other person's recovery, then regardless of whether there is a mental illness involved, it may not be a healthy relationship.

If you are experiencing such a situation, seeking professional couples counseling or individual counseling can be extremely helpful.

**Q34. Can People with Mental Illness Get Married?**

This is an extremely important and complex question. The short answer is: Yes, but with prerequisites.

Under China's current Civil Code, there is no complete prohibition on marriage for all people with mental illness. Whether one can marry mainly depends on a core criterion: whether the person has "full capacity for civil conduct."

Below, I will explain in detail from legal, medical, and practical perspectives:

**1. Legal Perspective (Core Basis)**

- Legal Principles: Article 1048 of the Civil Code stipulates: "Marriage between lineal blood relatives or collateral blood relatives within the third degree of kinship is prohibited." It does not list "suffering from a disease medically considered inappropriate for marriage" as a prohibited circumstance. (Note: This is an important change after the implementation of the Civil Code on January 1, 2021, replacing the old Marriage Law.)
- Key Condition: Although no longer prohibited, Article 1053 of the Civil Code stipulates the obligation to disclose major illnesses.
  - If one party suffers from a major illness, they shall truthfully inform the other party before marriage registration.
  - If not truthfully informed, the other party may request the people's court to revoke the marriage.
  - The request to revoke the marriage shall be made within one year from the date of knowing or 应当 knowing the cause for revocation.

What is "full capacity for civil conduct"? This means that when entering into the legal act of marriage, the person can understand the nature and consequences of marriage, as well as the responsibilities and obligations they shall bear, and can make a true expression of will.

If the person's condition is stable, they have insight, and can rationally make the decision to marry and understand its consequences, then marriage is legally permitted.

If the person is in an acute episode, lacks insight, and cannot recognize their own actions (such as during an acute episode of severe schizophrenia or severe mania), they shall be deemed a "person with limited capacity for civil conduct" or a "person without capacity for civil conduct." In such cases, their legal act of marriage is invalid.

**2. Medical and Ethical Perspectives**

- Type and Status of the Illness: Mental illness is a broad category, and its severity and impact on cognitive function vary greatly.
  - Stable Period: Patients with conditions such as depression, anxiety, and bipolar disorder who have undergone systematic treatment, are in stable condition, and have good social functioning are fully capable of marrying and forming a family.
  - Severe and Acute Episodes: Some severe mental illnesses (such as schizophrenia) may severely affect the patient's judgment and reality-testing ability when uncontrolled, making marriage inappropriate at that time.
- Ethical Obligation of Disclosure: From a medical and ethical perspective, truthfully informing one's partner of their health status is a sign of respect and responsibility to each other. This lays the foundation for establishing a trusting, open, and mutually supportive marital relationship, and helps the partner better understand and support the patient in the future.
- Genetic Risks: Some mental illnesses have a certain genetic tendency. Before deciding to have children, it is wise for both spouses to consult a professional doctor.

**3. Practical Considerations and Suggestions**

Marriage is not only a legal union but also the beginning of real life. For people with mental illness and their partners, more considerations are needed:

- Communication and Support: Does the partner understand the condition? Are they willing and able to provide support if the condition fluctuates in the future?
- Economy and Care: Will the illness affect work ability? Does the family have sufficient financial and care resources?
- Risk of Relapse: Do both parties have a common understanding of the possibility of illness relapse and have a plan to cope with it?
- Family Pressure: Do both families understand and accept it? This may become an important source of pressure in the marital relationship.

**Summary and Suggestions**

| **Situation** | **Can Get Married?** | **Explanation and Suggestions** |
| --- | --- | --- |
| Stable condition with insight | Yes | Legally permitted. It is strongly recommended to truthfully inform the other party of the condition before marriage and ensure that the other party fully understands and supports it. |
| In acute episode | No | Legally an invalid marriage. Priority should be given to systematic treatment, and marriage should be considered after the condition stabilizes. |
| Marriage with concealment of major illness | Marriage can be revoked | After learning the truth, the other party has the right to apply to the court for revocation of the marriage within one year, and the marriage shall be deemed to have never existed. |

**Suggestions for Patients:**

- Prioritize Treatment: Make the stability and control of the condition the top priority.
- Be Honest in Communication: Choose an appropriate time to truthfully inform the partner of the situation before the relationship deepens.
- Seek Professional Advice: Before marriage, you can communicate with a psychotherapist or doctor to obtain professional guidance.

**Suggestions for Partners:**

- Take the Initiative to Learn: Learn about the illness, including its symptoms, treatment, and prognosis.
- Evaluate Yourself: Seriously consider whether you are ready to take on this additional responsibility and challenge.
- Family Communication: Communicate openly with the patient and their family to discuss future life plans.

In summary, mental illness is not an absolute barrier to marriage. Marriage based on honesty, understanding, support, and adequate preparation is completely achievable for patients with stable conditions. The law protects the rights of "freedom of marriage" and "informed consent."

**Q35. How to Get Along Better with Family Members with Mental Illness?**

I understand that you are looking for ways to get along better with family members who have mental illness. This is a process that requires great patience, understanding, and skill, and by proactively seeking advice, you have already taken a very important step.

Getting along with people with mental illness is centered on balancing "support" and "boundaries." Below, I will provide you with a detailed guide from four aspects: understanding the illness, communication skills, self-care, and seeking support.

I have prepared a mind map for you, summarizing the core points of getting along with family members with mental illness. Let me explain the specific methods of each part in detail:

**1. Learn In-Depth and Understand the Nature of the Illness**

- Obtain Accurate Information:
  - Learn about the specific symptoms, treatment methods, and prognosis of the illness your family member is suffering from through authoritative channels (hospitals, professional books, reliable websites).
  - Attend mental health lectures organized by hospitals or communities and ask professional personnel directly.
- Distinguish Between the Person and the Illness:
  - Remember: Behaviors such as aggression, indifference, and unpredictability are often symptoms of the illness, not the family member's true intention.
  - Adopt the mindset of "Depression makes them not want to get out of bed" rather than "They are too lazy."
- Manage Expectations:
  - The recovery from mental illness is usually a wave-like process with relapses, which does not mean treatment failure.
  - Celebrate small progress (such as being willing to go out for a 10-minute walk today).

**2. Improve Communication and Reduce Conflicts**

- Validating Listening:

Poor responses:

"Don't overthink it; things aren't that bad" → Denying feelings

Effective validation:

"It sounds like you're feeling very anxious; that must be tough" → Acknowledging feelings

"Can you tell me more about how you feel?" → Encouraging expression

"I" statement practice:

"I get worried when you don't answer the phone all day" instead of "You're always so irresponsible"

"I need a break; can we talk again in 10 minutes?" instead of "I can't stand you!"

- Crisis Communication Principles:
  - When the family member is in an acute episode: Stay calm, give them space, and do not argue about delusional content.
  - Simple and clear instructions: "Can you sit down and drink a glass of water first?"
  - Safety first: Know emergency contact information (crisis intervention hotline, emergency hospital).

**3. Set and Maintain Healthy Boundaries**

Example of boundary setting:

"I love you and am willing to support you, but I cannot accept you shouting at me. If you start raising your voice, I will leave the room temporarily and we can talk again when we are both calm."

Boundary between support and indulgence:

- Support: Reminding them to take medication, accompanying them to medical appointments, helping prepare healthy meals.
- Indulgence: Helping them avoid all social activities completely, accepting unreasonable financial demands.

**4. Self-Care: You Are Not a Superhero**

Prevent caregiver burnout:

- Schedule regular "rest time": At least a few hours a week that are completely yours.
- Maintain your own social circle and work; do not revolve your entire life around the patient.
- Pay attention to early warning signs such as your own anxiety, insomnia, and low mood.

Practical stress reduction methods:

- 5-minute deep breathing exercise every day.
- Maintain physical exercise, even a short walk.
- Write a diary to process complex emotions.

**5. Build a Support Network and Do Not Bear It Alone**

Seek Professional Guidance:

- Regularly communicate with the family member's therapist (after obtaining appropriate authorization).
- Consider family therapy to improve the overall communication pattern.
- You can also seek individual counseling to deal with caregiving stress.

Connect with Peer Support:

- Find family support groups (online or offline) to share experiences with people who have similar experiences.
- Learn practical skills in reliable online communities, but be cautious about information screening.

Family Collaboration:

- Clarify division of labor: Who is responsible for accompanying medical visits, who manages medication, who provides emotional support.
- Hold regular family meetings to avoid all responsibilities falling on one person.

**Common Mistakes to Avoid**

- Overprotection: Doing everything within the patient's capabilities for them, which instead impairs their ability to recover.
- Arguing about symptoms: Arguing directly about the content of hallucinations and delusions usually only increases conflicts.
- Ignoring your own needs: Ultimately leading to caregiver burnout, leaving both parties in trouble.
- Believing in stigma: Mental illness is a health issue like diabetes, and there is no need to feel ashamed.

**When to Seek Professional Help Immediately**

Seek immediate professional help if the following situations occur:

- Remarks or behaviors indicating possible self-harm or harm to others.
- Sudden deterioration of symptoms (such as not sleeping or eating for several consecutive days, completely refusing to eat).
- New worrying symptoms (convulsions, confusion).

Finally, remember: You are doing a great job. Taking care of a family member with mental illness is a long journey with setbacks and progress. True support is not perfect dedication, but finding a balance between understanding and boundaries—being an anchor for others without losing your own shore. There is no need to pursue perfection; maintaining learning, adjustment, and self-care is the best way to accompany them.

If you are willing to share more specific situations (such as which mental illness it is, the current main challenges), I can provide more targeted suggestions.

**Q36. How to Judge if a Family Member Is Experiencing a Relapse of Mental Illness?**

Judging if a family member is experiencing a relapse of mental illness requires combining the historical manifestations of the specific illness, current symptom changes, and professional medical standards. Since the early warning signs of relapse vary greatly among different mental illnesses, I will provide you with a comprehensive, actionable observation framework and action guide.

Typically, a relapse of mental illness does not occur suddenly but has a "prodromal period" with subtle early warning signs. Timely detection of these signs and intervention is key to preventing a full relapse.

**Core Observation Dimension: Comparison with the State Before the Last Episode/During Recovery**

You can compare the family member's current state with their state during the stable period of the illness from the following aspects to find signs of "significant regression."

**1. Emotional Changes**

Relapse of low mood (common in depression):

- Reappearance of persistent low mood, sadness, despair, and unexplained crying.
- Complete loss of interest in previously enjoyed hobbies and activities.
- Becoming unusually irritable and easily angered over trivial matters.

Relapse of elevated mood (common in manic/hypomanic episodes of bipolar disorder):

- Abnormally elevated, excited, euphoric mood, or extreme irritability.
- Excessive energy and significantly reduced need for sleep (e.g., only sleeping 2-3 hours but still being energetic).
- Inflated self-confidence, even to the point of grandiosity (e.g., believing one has special abilities).

**2. Changes in Thinking and Cognition**

- Reappearance of delusions (firmly believing in ideas that do not conform to facts, such as being monitored, persecuted, or guilty).
- Reappearance of hallucinations (hearing, seeing, or smelling sounds or things that do not exist).
- Confused thinking, illogical speech that is difficult to understand, or extremely rapid topic shifts.
- Inability to concentrate, significant memory loss, and difficulty making decisions.
- Thoughts of worthlessness or suicidal ideation (this is the most dangerous sign and requires immediate attention).

**3. Behavioral Changes**

- Withdrawal: Refusing to go out, avoiding social interactions, not communicating with family members, and being alone for long periods.
- Abnormal behavior: Unusual words and deeds that are inconsistent with previous personality.
- Impulsive behavior: During mania, this may manifest as reckless spending, risky investments, speeding, etc.
- Deterioration of daily functioning: Poor personal hygiene (not bathing, not changing clothes), inability to complete daily work or housework.
- Changes in sleep and appetite: Difficulty falling asleep, early awakening, or excessive sleep; sudden loss or increase in appetite.

**4. Changes in Physical Sensations**

- Unexplained physical discomfort such as headaches, backaches, and stomachaches, but no abnormalities found in examinations.
- Constantly feeling exhausted and lacking energy.

**5. Changes in Attitude Towards Treatment**

- Suddenly refusing to take medication, hiding medication, or vomiting medication.
- Refusing follow-up appointments or denying the need for continued treatment.
- Claiming "I am completely cured and no longer need medication."

**How to Act: Family Support and Response Steps**

The more and more severe the observed signs, the higher the possibility of relapse. Once a relapse is suspected, please follow these steps:

1. Stay calm and do not panic excessively: Relapse is a common challenge in the treatment of mental illness, not the end of the world. Your calmness is crucial for the patient.
2. Communicate gently and express concern:
   - Choose a quiet, private time and express your worries using "I" statements instead of accusations.
   - For example: "I noticed you haven't been sleeping well lately and seem tired; I'm a bit worried about you." Instead of: "Why aren't you sleeping again!"
   - Listen to their feelings and do not rush to deny or argue about their hallucinations or delusions.
3. Contact professionals immediately:
   - First, contact the patient's attending doctor or psychotherapist. Describe the changes you have observed in detail.
   - Follow the doctor's professional advice, which may include: adjusting medication dosage, scheduling a follow-up appointment as soon as possible, or going to the emergency department.
4. Ensure safety:
   - If the patient makes remarks, plans, or takes actions that indicate harm to themselves or others, this is a psychiatric emergency.
   - Please call the emergency number (120) immediately, or take them to the emergency department of the nearest hospital, and clearly inform the medical staff of the patient's mental illness history and current dangerous situation.
5. Assist in managing daily life: During the recovery period, help them maintain a regular schedule, prepare healthy meals, and remind them to take medication.
6. Take care of yourself: Supporting someone with a mental illness is a long and emotionally draining task. Family members also need rest and support; you can seek help from other family members or join a family support group.

**Self-Checklist for Early Warning Signs of Mental Illness Relapse**

Instructions: After comparing with the family member's state during the stable period of the illness, if you find that many of the following symptoms have reappeared or significantly worsened, please be alert and consider contacting a doctor.

**Emotions and Feelings**

- Persistent low mood, sadness, or sense of despair
- Loss of interest or pleasure in almost all activities
- Abnormal irritability, easy anger, or getting upset over trivial matters
- (For bipolar disorder) Abnormally elevated mood, excitement, or excessive energy
- (For bipolar disorder) Significantly reduced need for sleep but still being energetic

**Thinking and Cognition**

- Confused thinking, rambling speech, or difficulty being understood
- Poor concentration and memory loss
- Indecisiveness and difficulty making decisions
- Feelings of worthlessness or excessive guilt
- (Important!) Suicidal thoughts or plans
- (Important!) Reappearance of hallucinations (e.g., hearing insults) or delusions (e.g., being monitored)

**Behavior and Social Interaction**

- Refusing social interaction and avoiding family and friends
- Deterioration in personal hygiene (not bathing, not changing clothes)
- Decreased work efficiency and difficulty completing tasks
- Unusual words and deeds
- (For bipolar disorder) Impulsive behavior (e.g., reckless spending, taking risks)

**Physical Condition and Daily Routine**

- Insomnia (difficulty falling asleep, easy awakening) or excessive sleep
- Significant loss or increase in appetite
- Constantly feeling tired and lacking energy
- Unexplained physical pain

**Treatment Adherence**

- Refusing to take medication, hiding medication, or vomiting medication
- Refusing follow-up appointments or contacting doctors
- Claiming to be "completely cured"

**Emergency Situations (Act Immediately)**

Seek emergency medical help immediately if any of the following occur:

- Explicitly expressing a desire to harm oneself or end one's life
- Having specific plans or taking actions to self-harm
- Expressing a desire to harm others
- Completely losing control of behavior and being in danger

**Recommended Follow-Up Steps**

- ≤ 3 mild symptoms: Keep observing, strengthen communication, and record changes.
- ≥ 4 symptoms or appearance of important/emergency items: Contact the attending doctor immediately and describe the observed symptoms in detail.
- Any emergency situation: Seek emergency medical help immediately.

Important Note: This checklist is only an auxiliary tool for family observation and cannot replace professional medical diagnosis. The final diagnosis must comply with the doctor's guidance.

I have created a detailed "Early Warning Sign Self-Checklist" for you, which you can use as a practical tool for daily observation to help you monitor changes in your family member's state in a more organized manner.

The most important thing is: Trust your intuition. As a family member, you know their daily state best. If you feel something is wrong, even if you can't explain the specific reason, it is worth consulting a doctor. Early intervention is the most effective way to prevent relapse.

If you need to discuss a more specific situation, I am here at any time.

**Q37. What to Do When a Family Member Experiences Auditory Hallucinations?**

When a family member experiences auditory hallucinations, it is a sign that requires great attention, calm response, and timely professional help. Auditory hallucinations are one of the psychotic symptoms, which may be caused by a variety of reasons and should never be simply attributed to "overthinking" or "being possessed by evil spirits."

Below are detailed response steps and precautions:

**Step 1: Stay Calm and Do Not Panic**

Your reaction is crucial. Please remember:

- Do not argue or deny: Do not directly say "That's all fake" or "No one is talking at all." For the patient, these voices are a real experience. Denying their feelings will only make them feel isolated and misunderstood, thus becoming more resistant to communication and seeking help.
- Express concern and support: Use a gentle, non-confrontational tone to express your worries. You can say: "I heard you say you're hearing some voices; this must be disturbing/scaring for you, right?" Or "I care about you very much; can we work together to find a way to solve this problem?"
- Ensure safety: Assess the content of the auditory hallucinations. If the voices are commanding (e.g., ordering them to harm themselves or others), hostile, or insulting, immediately raise your vigilance, remove potentially dangerous items from the home (such as knives, medications), and seek emergency medical help immediately.

**Step 2: Initial Communication and Information Collection**

On the premise of staying calm, you can try to gently understand the situation, which can provide important information for the doctor:

- Content: What are the voices saying? Are they familiar or unfamiliar voices? Is it one voice or multiple voices?
- Frequency and duration: When did it start? Is it continuous or occasional?
- Triggering situations: Under what circumstances is it more likely to occur? (e.g., when alone, under stress, before going to bed)
- The patient's reaction: How do these voices make them feel? (Scared, angry, happy?) Do they talk to the voices?

Note: If the other party is very resistant or excited, do not forcefully press for answers to avoid escalating emotions. Take whatever information you can get.

**Step 3: Seek Professional Medical Help Immediately (The Most Critical Step)**

The reasons behind auditory hallucinations can be complex and must be diagnosed by a professional doctor. Common reasons include:

- Mental and psychological illnesses: Such as schizophrenia, bipolar disorder, severe depression with psychotic symptoms, etc.
- Organic diseases: Brain tumors, epilepsy, infections, endocrine disorders, etc.
- Substance influence: Alcohol or drug abuse/withdrawal.
- Others: Extreme sleep deprivation, severe stress, etc.

How to seek medical treatment:

- Preferred: Psychiatric hospitals (mental health centers) or the psychiatric/psychosomatic medicine departments of general hospitals. Doctors in these institutions are most experienced in handling such issues.
- If the situation is urgent (e.g., there is a risk of harming others or destroying property, or self-harm), directly call 120, or go to the emergency department of a general hospital with a psychiatric department.
- Clearly describe all the situations you have observed and the details of the auditory hallucinations you have learned to the doctor. The doctor's diagnosis is the only basis for formulating subsequent treatment plans.

**Step 4: Cooperate with Treatment and Family Support**

After diagnosis, family support is an important part of recovery:

- Urge compliance with medical advice and medication: Medication is usually the main means of controlling symptoms. Be sure to urge the patient to take medication on time and in the correct dosage. Do not adjust the dosage or stop taking medication without authorization, otherwise, it may easily lead to a relapse or deterioration of the condition.
- Regular follow-up appointments: Accompany the patient to regular follow-up appointments to allow the doctor to assess the efficacy and adjust the treatment plan in a timely manner.
- Create a recovery environment:
  - Maintain a regular schedule: A regular daily routine, healthy diet, and moderate exercise help stabilize emotions.
  - Reduce stimulation: Keep the family environment quiet and harmonious, and avoid excessive accusations and conflicts.
  - Distract attention: Gently encourage the patient to participate in some simple activities, such as walking, listening to music, or doing housework, to help them shift their attention away from the voices.
- Acceptance and patience: The recovery from mental and psychological problems is a long process, and there may be relapses. Please give your family member unconditional love and great patience.

**Summary: Do's and Don'ts**

| **Do's** | **Don'ts** |
| --- | --- |
| Stay calm and listen patiently | Argue, laugh at, or deny (e.g., "Stop overthinking!") |
| Express understanding and support (e.g., "This sounds terrifying") | Blame or attribute (e.g., "You brought this on yourself") |
| Seek professional medical help immediately | Resort to superstitious practices (e.g., consulting a "witch doctor," drinking talisman water), which will delay treatment |
| Ensure the safety of the patient and those around them | Ignore dangerous signs (e.g., commanding auditory hallucinations) |
| Urge regular medication and follow-up appointments | Adjust medication without authorization |
| Learn about the illness and take care of yourself | Overprotect or restrict, depriving the patient of all autonomy |

Finally, caregivers should also pay attention to their own physical and mental health. Supporting a family member with auditory hallucinations is extremely stressful. You can seek support from other family members or join relevant family support groups. Only when you stabilize yourself can you better be a reliance for your family member.

A family member experiencing auditory hallucinations is a clear health alert, but through scientific, timely treatment and the warm support of the family, most cases can be effectively controlled, and the patient can resume a normal life.

Below are national and regional psychological assistance hotlines that you can call for guidance if necessary:

- National Psychological Assistance Hotline: 12320-5
- Beijing Suicide Research and Prevention Center: 010-82951332
- Shanghai Psychological Assistance Hotline: 021-12320-5

**Q38. What to Do When a Person with Mental Illness Exhibits Suicidal Behavior?**

This is an extremely serious and urgent situation. When a person with mental illness exhibits suicidal behavior, taking correct and rapid action is crucial. Below is a clear, step-by-step response guide.

**Step 1: Respond to Emergencies (If the Behavior Is Occurring)**

Primary Principle: Ensure safety and immediately seek professional medical help.

Assess the urgency:

- If the patient is in the process of committing suicidal behavior (e.g., taking an overdose of medication, cutting wrists, hanging), or has lost consciousness, is bleeding heavily, or has difficulty breathing: Immediately call the emergency number (120). This is the top priority.
- If the patient has a clear suicidal plan and intention but has not yet taken action: Immediately seek help and do not leave them alone.

First aid on-site (while waiting for rescue):

- Stay calm: Your calmness is a comfort to the patient.
- Ensure environmental safety: Gently remove potentially dangerous items (such as knives, scissors, ropes, medications).
- Provide basic first aid: In case of bleeding, press the wound with a clean cloth; if medication has been taken, bring the medicine bottle or container to help the doctor quickly identify it.
- Do not handle it alone: If the situation is dangerous, ensure your own safety first and wait for professional personnel to arrive.

**Step 2: Communication and Companion**

The way you communicate with the patient during the entire process is very important.

- Directly ask: Calmly and concernedly ask directly. For example: "Did you just try to hurt yourself?" "I'm very worried about you now; can you talk to me about your pain?" Directly talking about suicide will not prompt them to act, but will instead give them a chance to confide.
- Listen carefully: Listen to their feelings and thoughts without judgment. Let them know their pain is seen. Do not say things that deny their feelings, such as "You just need to cheer up" or "Don't be so weak."
- Express concern and support: Clearly tell them: "You are very important to me," "You are not alone; I will be here with you," "We will work together to get through this difficult time."
- Do not argue or blame: Avoid preaching, quarreling, or trying to make them feel guilty with words like "How can you be so selfish and let your parents down?" This will only aggravate their negative emotions.

**Step 3: Seek Professional Support and Follow-Up**

After handling the emergency, subsequent systematic treatment and support are key to preventing recurrence.

- Medical institutions are the first choice:
  - Immediately take the patient to the emergency department of a general hospital to treat physical injuries.
  - At the same time, contact the hospital's psychiatric department (mental health department) for an emergency consultation. The doctor will assess their mental state and decide whether hospitalization is needed to ensure their safety.
- Continuous treatment:
  - Strictly follow medical advice: Ensure the patient takes medication on time and attends regular follow-up appointments. Suicidal behavior is often a sign that the illness is not effectively controlled, and the treatment plan may need to be adjusted.
  - Conduct psychological treatment: Psychological counseling or therapy (such as cognitive-behavioral therapy, dialectical behavior therapy) can help the patient address the negative thoughts that lead to suicidal ideation, learn emotional regulation and crisis response skills.
- Develop a safety plan:
  - Collaborate with the therapist to develop a detailed "crisis emergency plan." The content includes: identifying early warning signs of suicide, internal coping strategies (such as exercise, listening to music), a list of personal supports to turn to (family, friends), contact information for professional support (doctor, therapist), and crisis hotline and emergency information.
- Provide social support:
  - Companion and supervision: In the early stage of recovery, try not to leave the patient alone, especially when they are in low mood.
  - Create a safe environment: Properly manage medications, sharp objects, and other dangerous items at home.

**Important Resources**

Crisis intervention hotlines: This is a very important and anonymous support channel.

- Hope 24 Hotline: 400-161-9995 (national, 24 hours)
- Beijing Suicide Research and Prevention Center: 010-82951332 (24 hours)

Some regions also have local psychological assistance hotlines, which can be queried and saved in advance.

**Reminders for Family Members and Companions**

- It's not your fault: The patient's suicidal behavior is caused by their illness, not the family's mistake.
- Take care of yourself: The process of accompanying the patient is full of stress and emotional exhaustion. You also need rest and support, and may need to seek psychological counseling if necessary.
- Stay vigilant: Even if the situation improves, pay attention to early warning signs in their mood and behavior.

**Summary of Core Action Path: In case of emergency, first provide first aid; ensure safety, then communicate; send to hospital, follow medical advice; accompany often, provide more support.**

I hope this guide can provide you with clear help. Please remember that immediately contacting professional emergency and medical personnel is the core and correct choice for dealing with such situations.

**Crisis Response Guide for Suicide Attempts by People with Mental Illness**

**🚨 Take Immediate Action in Emergencies**

**Step 1: Assessment and Calling for Help**

- Behavior has occurred (e.g., taking medication, cutting): Immediately call 120 (emergency rescue).
- Behavior has not occurred but intention is strong: Immediately call 110 (police) or 120, and ensure they are not left alone.

**Step 2: On-Site Handling Principles**

- Preserve life first: Treat physical trauma first (stop bleeding, maintain airway patency).
- Remove dangerous items: Gently remove knives, medications, ropes, etc.
- Companion throughout: Never let them out of your sight.
- Stay calm: Your calmness is the best stabilizer.

**Effective Communication Methods (Do's & Don'ts)**

| **Do's** | **Don'ts** |
| --- | --- |
| Ask directly: "You're in pain; do you want to end your life?" | Avoid the issue: "Don't think about those useless things." |
| Listen and accept: "I'm here with you; your feelings are very important." | Judge and blame: "You're so selfish/How can you let your parents down?" |
| Give hope: "We'll work together to find a way; the difficulty is temporary." | Blindly comfort: "Cheer up; it's no big deal." |
| Provide support: "You're not alone; I'll help you." | Argue right and wrong: Argue with them about the benefits of living. |

**Path to Seeking Professional Help**

**Emergency Handling Process**

Yes

No/Strong intention

Discover Crisis

Has the behavior occurred?

Call 120 for emergency rescue

Call 110 or 120

Take to the emergency department of a general hospital

Request psychiatric consultation at the same time

Hospitalization or outpatient treatment according to doctor's advice

Yes

No/Strong intention

Discover Crisis

Has the behavior occurred?

Call 120 for emergency rescue

Call 110 or 120

Take to the emergency department of a general hospital

Request psychiatric consultation at the same time

Hospitalization or outpatient treatment according to doctor's advice

**Key Contact Information**

- Emergency Center: 120
- Police: 110
- Psychological Crisis Hotlines:
  - Hope 24 Hotline: 400-161-9995
  - Beijing Crisis Intervention Center: 010-82951332
  - Local Mental Health Center: _________(Please query and fill in advance)

**Long-Term Follow-Up Support and Prevention**

- Treatment adherence: Strictly follow medical advice, take medication on time, and attend regular follow-up appointments.
- Psychological treatment: Seek long-term psychological counseling to learn emotional regulation skills.
- Develop a safety plan: Collaborate with a doctor/therapist to clarify:
  - What are the early warning signs?
  - What can you do when emotional control is lost?
  - Who can you contact? (List 3 relatives/friends' phone numbers)
  - What are the professional help resources? (Save the above contact information)
- Environmental safety: Properly manage medications and sharp objects at home.
- Family member self-care: Caregivers also need rest and support; they can seek family support groups or counseling.

**Important Reminders**

- Directly talking about suicide will not induce behavior; on the contrary, it is the key to opening the door to seeking help.
- The patient's suicidal thoughts are symptoms of the illness, not the family's fault.
- Recovery is possible; systematic treatment and support are the path to recovery.

This guide cannot replace professional medical advice; please contact professional personnel first in a crisis.

**Q39. What to Do When a Person with Mental Illness Shows Violent Tendencies?**

This is an extremely important and urgent question. Handling violent tendencies in people with mental illness requires calmness, skill, and the correct intervention of resources. I will provide you with a comprehensive guide from emergency response to long-term management.

In the face of such a situation, please remember the primary principle: Ensure the safety of yourself, the patient, and everyone around you.

**Emergency Response Measures in Urgent Situations**

**Stay Calm and Assess Risks**

- Calm yourself: Take a deep breath and do not panic. Your panic may exacerbate the patient's excitement.
- Quickly scan the environment: Pay attention to whether the patient has objects in their hands or nearby that can be used as weapons (such as knives, sticks, heavy objects).
- Plan an escape route: Ensure you know where the nearest exit is to leave yourself a safe space.

**Ensure a Safe Distance and Escape**

- Maintain a safe distance: Keep at least an arm's length away and do not enter the patient's "attack range."
- Do not engage in physical confrontation: Unless you are a trained professional, avoid physical conflicts with the patient. Your goal is to escape and seek help, not to subdue them.
- Remove other personnel: Discreetly let other irrelevant personnel, especially children and the elderly, leave the scene immediately.

**Conduct Soothing Communication**

- Speak in a calm tone: Use a low, slow, and calm voice.
- Express empathy: Use sentences such as "I understand you're very angry/uncomfortable" and "I see you're in a lot of pain" to acknowledge their emotions, rather than denying or blaming them.
- Give space: Say "I won't hurt you" and "I'm here with you," but maintain a distance at the same time. Avoid direct eye contact, which may be perceived as a provocation.
- Listen: Let them express their anger and frustration without interrupting or arguing.

**Seek Emergency Professional Help**

If the situation gets out of control or is very dangerous, immediately call 110 (police) or 120 (emergency number).

Clearly explain to the operator: "There is a person with a mental illness here who is currently emotionally agitated and has violent tendencies; we need emergency help."

Provide the exact address and on-site situation.

Contact the patient's psychiatrist or psychotherapist: If they have an emergency contact number, call it immediately.

**Follow-Up Steps and Long-Term Management After Emergencies**

After the emergency is resolved, subsequent handling is crucial to prevent similar situations from happening again.

**Medical Follow-Up and Treatment Adjustment**

- Immediate follow-up appointment: Take the patient to see a psychiatrist as soon as possible. Violent tendencies may be a sign of a relapse or deterioration of the condition (e.g., schizophrenia, manic episodes, severe paranoid states).
- Evaluate the medication plan: The doctor may adjust the type or dosage of medication, especially adding some medications that help control impulsivity and aggression.
- Strictly follow medical advice: Ensure the patient takes medication on time and in the correct dosage; this is the basis for stabilizing the condition.

**Identify "Early Warning Signs" of Violent Behavior**

Most violent behaviors are predictable before they occur. Family members and caregivers need to learn to identify these early signs for early intervention:

- Emotional changes: Irritability, restlessness, anxiety, and large mood swings.
- Verbal signals: Threatening language, insults, incoherent speech, and talking about violent content.
- Behavioral signals: Restlessness, pacing, clenched fists, throwing small objects, and changes in sleep and eating patterns.
- Exacerbation of psychotic symptoms: Hallucinations (e.g., hearing commanding voices) and delusions (e.g., delusions of persecution) become more frequent and intense.

**Create a Safe Family Environment**

- Manage dangerous items: Properly store knives, scissors, ropes, medications, etc.
- Establish an emergency plan: Discuss and rehearse with family members what each person should do in case of an emergency, write down emergency phone numbers, and post them in a prominent place.
- Avoid direct conflicts: Do not argue with the patient about the authenticity of their delusions or hallucinations; this will only intensify conflicts. You can try to divert their attention, such as "Shall we eat something first/watch TV?"

**Caregiver Self-Care and Support**

- It's not your fault: The patient's violent behavior is caused by the illness, not their true intention, nor is it the caregiver's failure.
- Seek support: Join patient family support groups to communicate with people who have similar experiences, which can provide great emotional support and practical advice.
- Save energy: The caregiving work is long and arduous; ensure you get enough rest, and ask other family members to take turns if necessary.

**Mental Health Crisis Response Guide: When a Patient Shows Violent Tendencies**

**🚨 Emergency Situation - Take Immediate Action**

**1. Top Priority: Ensure Safety**

- Stay calm: Take a deep breath and calm yourself.
- Assess the environment: Pay attention to potential dangerous items.
- Plan an escape route: Confirm the nearest safe exit.
- Maintain a distance: Keep at least an arm's length of safe distance.

**2. Emergency Help Numbers**

- Life-threatening emergency: 110 (police)

Clearly state: "A person with a mental illness has violent tendencies and needs emergency help."

- Medical emergency: 120 (emergency number)
- Psychological crisis intervention: Psychological assistance hotline 010-82951332

**3. Effective Communication Skills**

✅ What to say:

- "I understand you're very angry/uncomfortable now."
- "I'm here with you, but we need to keep this distance."
- "We can all get through this safely."

❌ What to avoid saying:

- "Calm down!" (Command)
- "It's no big deal." (Minimizing feelings)
- "If you keep doing this, I'll..." (Threat)

**Follow-Up Measures After Emergencies**

**1. Medical Follow-Up**

- Immediately schedule a follow-up appointment with a psychiatrist.
- Describe the incident in detail to the doctor.
- Discuss adjusting the medication treatment plan.
- Ensure medication is taken on time and in the correct dosage.

**2. Early Warning Sign Identification (Early Intervention)**

- Emotional changes: Irritability, restlessness, anxiety.
- Verbal signals: Threatening language, insults, talking about violence.
- Behavioral signals: Restlessness, pacing, clenched fists, throwing things.
- Exacerbation of symptoms: Hallucinations and delusions become more intense.

**3. Family Safety Preparation**

- Properly store dangerous items (knives, medications, etc.).
- Develop a family emergency plan.
- Post emergency phone numbers in a prominent place.
- Arrange for caregivers to take turns resting.

**Caregiver Self-Care**

- Remember: Violent behavior is a symptom of the illness, not your fault.
- Seek support: Join family support groups.
- Save energy: Ensure adequate rest.
- Set boundaries: Protect your own physical and mental health.

**Important Contact Information**

| **Service Type** | **Phone Number** | **Service Hours** |
| --- | --- | --- |
| Emergency Police | 110 | 24 hours |
| Medical Emergency | 120 | 24 hours |
| Psychological Assistance Hotline | 010-82951332 | 24 hours |
| Attending Doctor | ________________ | ________ |
| Backup Contact | ________________ | ________ |

Please fill in and save your personal important contact information.

I have created a detailed response guide for you, which you can download or print for quick reference when needed. This guide covers all key steps from emergency response to follow-up management.

The most important thing is that you do not need to bear this alone. Utilizing professional resources and support systems can not only better help the patient but also protect your own physical and mental health. If you have more specific questions or want to learn more about a certain aspect, please feel free to tell me.

**Q40. What to Do If a Person with Mental Illness Refuses to See a Doctor?**

This is an extremely difficult but common situation. Persuasion requires great patience, skill, and empathy, with the core being to build trust rather than win an argument.

Below is a detailed action guide, hoping to provide you with help.

**Core Principle: Mindset First**

Before starting any conversation, please adjust your mindset first:

**Understand the Root Causes of Resistance**

The patient's refusal usually stems from:

- Stigma: Fear of being labeled as "crazy."
- Fear: Fear of unknown treatment, side effects of medication, or talking to doctors.
- Lack of Insight (Anosognosia): This is a core symptom of many mental illnesses. The patient's brain cannot recognize that they are ill. They may firmly believe "I'm not ill; you're the ones with problems." This is not stubbornness but a symptom itself.
- Despair: Believing "seeing a doctor won't help" and losing confidence in recovery.
- Fear of Losing Control: Worrying about losing autonomy once seeking medical treatment.

**You Are Not "Convincing," but "Connecting"**

The goal is not to refute them with logic, but to make them feel your support and understanding, so that they are willing to try to change for themselves.

**Manage Your Expectations**

Success rarely comes from a single conversation. This is a long process, and small progress (such as them being willing to discuss "sleep problems") is worth celebrating.

**Action Steps: From Communication to Intervention**

**Step 1: Create a Safe Communication Environment**

- Choose the right time: Have the conversation when they are in a relatively stable mood, you are alone, and there are no distractions. Never bring it up during an argument or when their symptoms are flaring up.
- Use "I" statements instead of "you" statements:
  - Do not say: "You always overthink; you must see a doctor!" (This is an accusation and an order)
  - You can try: "I noticed you haven't been sleeping well lately; I'm very worried about you." Or "Seeing you in so much pain makes me sad; can we work together to find a way?"
- Normalize and empathize:
  - "Feeling low and stressed is something many people go through; it's not your fault."
  - "If I were in your situation, I might feel very uncomfortable too."
  - "Seeking help is not a sign of weakness; on the contrary, it's the first step to being brave."

**Step 2: Focus on Specific Issues, Not the "Illness" Label**

Directly talking about words like "mental illness" or "depression" may trigger strong resistance. Instead, start with specific life problems that they may also be troubled by.

- Do not say: "I think you have depression; you need to see a doctor."
- You can say: "You don't seem interested in your favorite game lately; is it because you're not sleeping well and lack energy? Maybe a doctor can help us improve your sleep so you feel more energetic?"

Other entry points: Insomnia, changes in appetite, inability to concentrate, persistent headaches or stomachaches, stress from being unable to work, etc. Position seeing a doctor as a way to solve these specific problems.

**Step 3: Provide Options and Empower a Sense of Control**

Make them feel that they are part of the decision-making process, not passively accepting arrangements.

- Provide choices: "I found information about two doctors. One is Dr. Wang, who specializes in talk therapy; the other is Dr. Li, who is very experienced in medication adjustment. Which one would you like to learn about first?"
- Lower the threshold:
  - "We don't have to take medication; we just go talk to an expert first, listen to their opinions, and the final decision is always yours."
  - "I can go with you and wait outside, or we can go in together—whichever you prefer."
  - "How about we try online psychological counseling first? No need to go out; you can do it in your own room."

**Step 4: Utilize Someone They Trust**

Sometimes they may not listen to you, but the words of someone else they respect or trust (such as a friend, elder, or teacher) may be more effective. You can privately ask this person for help and persuade them in the same way.

**Step 5: Seek Professional Guidance Yourself**

While you are trying to persuade them, you yourself are in great need of support.

- Consult for yourself: You can first see a psychiatrist or psychotherapist alone. Tell them about your dilemma: "My family member may be ill but refuses to see a doctor; what should I do?" They can provide you with the most professional communication strategies and resources tailored to the specific situation.
- Join a support group: Look for mental health family mutual aid associations. Communicating with people who have the same experiences can greatly reduce your sense of loneliness and pressure, and provide valuable practical experience.

**If All Persuasion Fails: Intervention in Crisis Situations**

When the patient shows the following situations, safety is the top priority, and mandatory intervention may be needed:

- Explicitly threatening to harm themselves or others.
- Showing severe symptoms such as hallucinations and delusions that may lead to dangerous behavior.
- Being unable to eat or drink due to mental state, with life-threatening vital signs.

Measures that can be taken at this time:

- Call emergency numbers: In China, you can call 110 (police) or 120 (emergency), clearly explain the patient's mental state and current danger, and request assistance to send them to a psychiatric hospital.
- Involuntary hospitalization: According to the Mental Health Law of the People's Republic of China, when a patient "engages in behavior that endangers their own or others' safety, or poses a risk of harming themselves or others," even if they refuse, involuntary hospitalization treatment can be implemented after a diagnosis by a professional doctor. This is usually a last resort.

**Important Reminder: Caregiver Self-Care**

The persuasion process is extremely exhausting, and you must take care of yourself. You cannot pour water from an empty cup. Ensure you have your own rest time, social circle, and emotional outlet. Your health and stability are the most long-term and reliable support for the patient.

Please remember that your role is a "partner accompanying them through difficult times," not a "doctor correcting them." Your patience, understanding, and continuous love are in themselves a healing medicine.

**Guide to Persuading a Person with Mental Illness to Seek Medical Treatment**

**🤝 Core Mindset**

- Understand the root causes of resistance: Stigma, fear, lack of insight (a symptom, not stubbornness), despair, fear of losing control.
- Goal is to connect: Build trust rather than win an argument.
- Manage expectations: This is a process, and small progress is worth celebrating.

**🗣️ Communication Strategies**

**Create a Safe Environment**

- Timing: Choose a time when they are in a stable mood and in a private, undisturbed environment.
- Use "I" statements: "I noticed you're not sleeping well; I'm worried" vs. "You must see a doctor."
- Empathize and normalize: "Many people go through this; it's not your fault" "Seeking help is a sign of bravery."

**Focus on Specific Issues**

- Avoid labels: Do not mention "depression" or "mental illness."
- Start with specific troubles: Insomnia, changes in appetite, inability to concentrate, headaches, stress.
- Position as a solution: "A doctor may be able to help you improve your sleep so you feel more energetic."

**Empower a Sense of Control**

- Provide choices: Introduce the strengths of different doctors and let them choose.
- Lower the threshold: "Just talk first; the decision is yours" "I'll go with you or wait outside" "Try online counseling first."

**👥 Utilize External Support**

- Someone they trust: Ask a friend or elder respected by the patient to assist in persuasion.
- Professional guidance: Consult a doctor first to obtain targeted strategies.
- Support groups: Join family mutual aid associations to gain experience sharing.

**⚠️ Crisis Intervention (When It Occurs)**

- Immediate danger: Threatening to harm themselves or others, hallucinations/delusions that may lead to danger, inability to eat/drink.
- Take action:
  - Call 110 (police) or 120 (emergency).
  - Explain the mental state and danger.
  - Involuntary hospitalization may be required according to the Mental Health Law (last resort).

**Caregiver Self-Care**

- Protect your own health: Ensure rest, maintain a social circle, and release emotions.
- Recognize your role: You are a "partner accompanying them through difficult times," not a "doctor correcting them."
- Seek support: Your stability is the patient's most long-term support.

Patience, understanding, and continuous love are in themselves a healing medicine.

**Q41. Should Family Members with Mental Illness Be Allowed to Manage Money Independently?**

This is an extremely complex question with no uniform answer. It requires extremely careful evaluation based on specific circumstances. The core lies in balancing: respecting the patient's autonomy and personal dignity, and protecting their financial security and well-being.

Simply answering "yes" or "no" is irresponsible. The correct approach is to conduct a systematic evaluation and adopt the most appropriate management method for the current situation.

**Core Evaluation Framework: Key Dimensions to Consider**

Before making a decision, family members and medical professionals should jointly evaluate the following aspects:

**Diagnosis and Severity of the Illness**

- Type of illness: Is it depression, anxiety, schizophrenia, or bipolar disorder? Different illnesses have vastly different impacts on cognition and judgment.
- Current state: Is the patient in an acute episode, recovery period, or stable period of the illness? During the acute phase, judgment may be severely impaired; during the stable phase, functioning may be completely normal.
- Cognitive function: Does the illness directly affect the patient's memory, calculation ability, logical reasoning, and judgment of risks?

**The Patient's Ability to Handle Finances**

- Basic skills: Can they clearly calculate income and expenses, recognize currency denominations, and safely use ATMs or online payments?
- Planning ability: Can they formulate and implement a simple budget to ensure that basic living needs (rent, food, utilities) are prioritized?
- Risk awareness: Are they easily deceived, or do they have a tendency to impulsive spending or reckless spending (common during manic episodes)? Can they understand the consequences of complex financial behaviors such as investments and loans?

**Complexity and Risk of Financial Management**

- Scale of funds: Is it a small amount of daily expenses, or a large amount of assets or inheritance?
- Complexity of decisions: Is it necessary to make major financial decisions such as investments, purchasing real estate, or signing important contracts?

**Perfection of the Support System**

- Family support: Can family members provide appropriate, non-intrusive supervision and help?
- Professional support: Are there social workers, psychotherapists, or financial advisors who can provide guidance?

**Possible Financial Management Options (From Full Autonomy to Full Custody)**

Based on the evaluation results, a progressive or hybrid plan can be chosen, rather than a simple "all or nothing."

| **Plan** | **Applicable Situations** | **Advantages** | **Disadvantages** |
| --- | --- | --- | --- |
| 1. Full independent management | The illness is in a stable phase, cognitive function is intact, and there is a good history of financial decision-making. | Maximizes respect for autonomy and dignity, and promotes self-confidence and a sense of independence. | If the illness relapses, there may be significant financial risks. |
| 2. Joint management | The patient's ability is partially impaired but still has the willingness and ability to participate. | One of the most recommended models. Establish a joint account where major expenses require the consent of both parties, and small daily expenses can be decided independently. Provides protection while retaining autonomy. | Requires a high level of trust and good communication between both parties. |
| 3. Supported decision-making | The patient has the ability to make decisions but needs information and tool support. | The patient is still the decision-maker; family members or professionals only provide information, explain options, and assist in planning, but do not make decisions on their behalf. This is in line with modern disability rights concepts. | Requires a lot of patience and skill from the supporter. |
| 4. Power of Attorney | When the patient is capable, designate a trusted family member in advance to manage finances if they lose capacity in the future. | Has legal effect and is the best way to plan ahead. Authorized voluntarily by the patient when they are sober, and their wishes are respected. | Requires advance planning and a high level of integrity from the agent. |
| 5. Guardianship/Custody | Through legal proceedings, the court determines that the patient is incompetent and appoints a guardian to manage all their property. | Provides the strongest protection for patients with complete inability to take care of themselves. | A last resort. Completely deprives autonomy, may cause great psychological damage to the patient, and the process is complex and expensive. |

**Conclusion and Suggestions**

Instead of simply "allowing" or "not allowing," a dynamic management plan based on evaluation, communication, and legal protection should be established.

- Prioritize communication: Have an open and honest discussion with the patient about concerns and difficulties in financial management, and listen to their thoughts and wishes. The goal is cooperation, not control.
- Seek professional evaluation: A psychiatrist or clinical psychologist can conduct a professional evaluation of the patient's cognitive ability and decision-making ability, which is an important basis for making judgments.
- Consult legal experts: Understand the laws and regulations in your region (such as guardianship systems, power of attorney provisions), and choose the most suitable and least restrictive legal tool to provide protection.
- Adopt the principle of least restriction: Start with the least restrictive plan (such as joint management), and only consider stricter measures when absolutely necessary. The goal is to help the patient achieve financial autonomy as much as possible.
- Regular re-evaluation: The patient's condition will change, and the financial management method should be adjusted accordingly. Conduct regular re-evaluations and gradually relax restrictions when the situation improves.

Ultimately, the answer depends on the specific illness your family member is suffering from, the current stage they are in, and their personal cognitive function level. There is no shortcut to this matter; it requires love, patience, professional knowledge, and careful weighing.

**Q42. What Is the Fastest Way to Find a Lost Person with Mental Illness?**

When a person with mental illness goes missing, time is of the essence. The fastest and most effective way is to call the police immediately (dial 110) and at the same time mobilize relatives and friends to focus on searching in the area where the patient was last seen.

To help you act quickly and systematically, I have compiled a detailed emergency guide and preventive measures for you.

**Emergency Search and Prevention Guide for Missing Persons with Mental Illness**

**I. Golden 24-Hour Emergency Response Process**

**Step 1: Call the Police Immediately and Mobilize Relatives and Friends (Within 0-1 Hour)**

**Call 110 to Report the Missing Person Immediately**

- Key information: Clearly inform the police that the patient has a mental illness; this is an important basis for the police to prioritize handling.
- Provide detailed information:
  - Name, age, gender, height, weight.
  - Specific clothing when missing (color, style, shoes).
  - Clear recent photo (preferably an unretouched daily photo).
  - Exact time and location of last sighting.
  - Type of illness, medical history, and commonly used medications (whether dangerous behavior may occur).
  - Possible destinations or places they are used to going.
- Be sure to ask for: After reporting the case, be sure to record the police officer's badge number and the case number for follow-up.

**Simultaneously Mobilize Relatives and Friends to Organize a Search**

- Division of labor and cooperation: Immediately organize family members and friends to search separately in different directions, centered on the last place of disappearance.
- Key areas: Prioritize searching places familiar to the patient, such as frequently visited parks, supermarkets, stations, hospitals, etc.

**Step 2: Retrieve Surveillance to Lock the Direction (Within 1-4 Hours)**

Immediately go to surrounding stores, community property management, banks, and other units near the last place of appearance, request to view surveillance videos, and try to accurately lock the patient's walking direction and approximate range to provide key clues for the search and the police.

**Step 3: Online Dissemination to Expand the Scope (Ongoing)**

- Compile a missing person notice: Include the patient's photo, physical characteristics, time and location of disappearance, and contact phone number.
- Release channels:
  - Local Moments and WeChat groups: Spread very quickly, especially covering community groups and owner groups near the patient's place of residence and work.
  - Weibo: Use hashtags (such as #MissingPersonNotice#[City Name] Looking for Someone) and @ local public security, media, and public welfare influencers to leverage their public communication attributes.
  - Douyin/Toutiao: Utilize their "local" function based on geographic location push, which can effectively push to nearby users who may have seen the patient.
  - Public welfare platforms: Such as the "Baby Come Home" platform, which also has a missing person section.

**II. Utilizing Technology and Equipment (Prevention and Search)**

**Prevention Is Better Than Searching**

- Wear an identification card: Make a waterproof information card with name, emergency contact phone number, and illness information, and have them carry it with them (can be placed in a pocket, hung on a keychain, or sewn into clothing).
- Use anti-lost equipment:
  - GPS locator: Choose a lightweight device with long battery life, such as a bracelet, watch, or pendant, which can view the location in real-time on a mobile APP.
  - Anti-lost positioning shoes: Provided by some public welfare projects, with a positioning chip built into the shoes, which is not easy to lose.
- Daily filing: Take and file recent frontal photos of the patient, and record their key physical characteristics and habits.

**III. Follow-Up Prevention and Daily Management**

- Strengthen care: Try not to let the patient go out alone, especially when the condition is unstable.
- Communication and training: Daily repeatedly teach the patient to remember the home address, relatives' names, and phone numbers.
- Community registration: Maintain communication with the local community and police station, let them know the situation, and they can provide help if necessary.
- Seek medical treatment in a timely manner: Regular follow-up appointments to stabilize the condition are the fundamental way to reduce the risk of running away.

Please remember: Calling the police is the fastest and most effective first way to find a missing person with mental illness. Do not delay due to any concerns!

I have created a detailed emergency guide for you, covering the complete process from calling the police immediately to follow-up prevention. You can act immediately in accordance with the steps in the document, among which immediately dialing 110 and clearly stating the identity of "a person with a mental illness" is the most crucial step.

This guide also includes effective preventive measures such as using GPS equipment and making information cards. It is recommended that you carefully read the prevention section after finding your family member to prevent similar situations from happening again. I hope your family member can return safely as soon as possible!

**Q43. As a Family Member of a Person with Mental Illness, How to Relieve My Own Stress?**

As a family member of a person with mental illness, the pressure you bear and the care you provide are very real and worthy of recognition. This is a journey full of challenges, but you are by no means alone. Taking care of yourself is not selfish; it is the cornerstone of your ability to provide continuous support.

Below are some specific methods and suggestions to relieve your own stress, hoping to provide you with some help and direction.

**I. Adjust Cognition and Mindset: "Untie" Yourself**

- Allow yourself to have various emotions: Feeling tired, frustrated, angry, helpless, or even resentful is normal. These emotions are natural reactions to difficult situations and do not mean you do not love your family member. Acknowledge and accept these feelings instead of feeling guilty or criticizing yourself.
- Redefine "success": Adjust the standard of success from "curing the family member" to "providing continuous support and companionship." The recovery from mental illness is often a long and repetitive process; today's "bad" does not mean tomorrow's failure. Celebrate every small progress.
- Establish realistic expectations: Accept the fact that you cannot control everything. You cannot bear the illness on behalf of the family member, nor can you force them to get better. Your role is a supporter, not a savior. Focus on what you can control—your own reactions, the boundaries you set for yourself, and your willingness to seek help.
- Learn about the illness and eliminate fear: Understanding the relevant knowledge of the illness your family member is suffering from (etiology, symptoms, treatment methods, prognosis) can reduce anxiety and misunderstandings caused by the unknown. Knowledge can empower you, allowing you to better understand that the patient's behavior is not directed at you, but a manifestation of the illness.

**II. Seek External Support: You Don't Need to Bear It All Alone**

- Join a family support group: This is an extremely effective way to reduce stress. Communicating with people who have similar experiences, you will find that your emotions are deeply understood, and you can also gain valuable practical advice and experience sharing. This "peer support" can greatly alleviate the sense of loneliness.
- Seek professional help: Consider finding a psychotherapist or counselor for yourself. They can provide a safe, confidential space to help you process complex emotions, learn more effective coping strategies and communication skills, and prevent caregiver burnout.
- Communicate openly with friends and family: Choose one or two trusted friends or relatives and honestly tell them about your situation and feelings. Even if they cannot provide solutions, simple listening and companionship are great comforts. Clearly tell them what kind of help you need (such as helping cook a meal, temporarily taking care of for an hour, or just sitting with you).
- Utilize community and social resources: Understand what resources local community mental health centers, disabled persons' federations, and other institutions can provide, such as rehabilitation activities and temporary care services, to reduce your caregiving burden.

**III. Establish Clear Boundaries: Protect Your Physical and Mental Health**

- Learn to say "no": Recognize your own limits. Gently but firmly refuse requests that exceed your ability or energy. This is not indifference, but to be able to go further.
- Distinguish between "their business" and "my business": The patient's emotions and behaviors are "their business," and you do not need to be fully responsible for them. Your emotions and health are "my business" and need your priority attention. Avoid over-involvement or being completely wrapped up in the patient's emotions.
- Arrange "power-off" time: Every day or week, arrange a period of time that is completely personal and undisturbed. During this time, completely let go of the caregiver role and do anything that makes you feel happy and relaxed, even if it's just staring blankly, listening to music, or taking a bath.

**IV. Practice Self-Care: Specific Actions to Recharge Your Physical and Mental Energy**

- Maintain basic health:
  - Sleep: Try to ensure regular, adequate sleep.
  - Diet: Pay attention to balanced nutrition, and avoid overeating or loss of appetite due to stress.
  - Exercise: Engage in regular physical exercise, such as walking, running, or yoga. Exercise is an excellent way to release stress and can produce endorphins that make people feel happy.
- Practice relaxation techniques:
  - Deep breathing: When feeling anxious, try a few minutes of slow, deep breathing (inhale for 4 seconds, hold for 4 seconds, exhale for 6 seconds).
  - Mindfulness meditation: Learn simple mindfulness exercises through APPs (such as Tidal, Headspace) to help yourself focus on the present moment instead of dwelling on regrets about the past or worries about the future.
- Retain hobbies: Try to maintain hobbies that bring you a sense of accomplishment and happiness, whether it's gardening, reading, painting, or gathering with friends. This is an important part of your identity, reminding you that you are not only a "caregiver" but also yourself.

**V. Communication and Collaboration Within the Family**

- Family meetings: Hold regular family meetings to openly discuss each other's stress, feelings, and needs, share caregiving responsibilities together, and avoid all pressure focusing on one person.
- Communicate with the patient: When the patient is in a relatively stable state, try to communicate using "I" as the subject (for example, "I see you're sad; I feel bad too, and I want to help you") instead of blaming ( "You're always like this").

Please remember: Your physical and mental health is equally important as the patient's. The safety instructions on airplanes always remind us to put on our own oxygen mask first before helping others. This is a profound metaphor—only by ensuring your own well-being can you have more strength to support your loved ones.

You have done enough. Allow yourself to rest, allow yourself to seek help, and allow yourself to have moments of not being so "strong." This is a marathon, not a sprint.

If you feel that the pressure is unbearable, please be sure to call a psychological assistance hotline (for example: Beijing Psychological Assistance Hotline: 010-82951332) to seek immediate support.

If you are willing to share more specific situations (such as which type of illness the patient has, what your biggest trouble is), I can try to provide more targeted suggestions.

**Q44. If a child shows "being quiet, irritable, and school-avoidant", could there be mental health issues?**

Yes, a child displaying the combination of behaviors such as "being quiet, irritable, and school-avoidant" may indeed have underlying mental health concerns that require attention.

This set of symptoms is a crucial signal indicating the child may be experiencing internal distress or struggles that they cannot effectively express through words, thus manifesting through behaviors. It does not necessarily equate to a severe mental illness, but it is definitely a "yellow card warning" that parents should take seriously and actively address.

Below is a detailed analysis of potential causes and recommended responses.

**Possible Causes**

These behaviors are usually the result of intertwined factors, which can be roughly categorized into the following types:

**1. Psychological and Emotional Issues (the most important aspect to focus on)**

- Depressive mood/Depression: This is the most critical possibility to be vigilant about. Unlike adults who may directly express sadness, childhood depression often presents as irritability, irritableness (rather than low mood), decreased interest (lack of enthusiasm for previously enjoyed activities), refusal to attend school, silence, and physical complaints (such as headaches, stomachaches).
- Anxiety Disorders: Excessive anxiety (such as separation anxiety, social anxiety, generalized anxiety) can leave children in a state of high tension and fear. School avoidance may serve as an escape from stressors at school (such as exams, social interactions); irritability and silence are manifestations of unresolved internal pressure.
- Trauma or Stress-Related Reactions: Experiencing or being exposed to traumatic events (such as school bullying, domestic violence, the loss of a loved one, intense parental conflicts, severe criticism or humiliation) can leave children feeling scared and helpless, leading to withdrawal and emotional outbursts.

**2. Developmental and Environmental Factors**

- Excessive Academic Pressure: Failure to meet high expectations (from parents or themselves), learning difficulties, and declining academic performance can lead to feelings of frustration, which in turn trigger school avoidance and escape behaviors.
- Social Difficulties: Having no friends at school, being isolated or excluded by peers, or struggling with interpersonal relationships. Being quiet and avoiding school may reflect avoidance of the social environment.
- Family Factors: A tense family atmosphere, strained parental relationships, lack of effective parent-child communication, and overly harsh or neglectful parenting styles can all leave children feeling anxious and angry.
- Early Adolescent Changes: For older children (such as those in upper primary school), the onset of adolescence may begin, and hormonal changes can lead to increased emotional volatility.

**3. Other Conditions to Rule Out**

- Neurodevelopmental Disorders: Conditions such as Autism Spectrum Disorder (ASD) or Attention-Deficit/Hyperactivity Disorder (ADHD) may go unrecognized in early childhood. As academic and social demands increase, associated difficulties (such as communication barriers, executive function deficits) can become more prominent, leading to emotional and behavioral problems.
- Physical Illness: Certain chronic illnesses or discomfort (such as insufficient sleep, thyroid issues, anemia) can also cause fatigue, emotional instability, and poor concentration, which may manifest as school avoidance and irritability.

**Recommendations for Parents: How to Respond**

Your primary task is to observe, communicate, and seek professional help, rather than forcing behavioral changes.

**Create a Safe Communication Environment (the most important step)**

- Do not force the child to speak. Choose a moment when the child feels relaxed (such as after a walk or playing a game) and express your concern in a caring, non-judgmental tone: "I've noticed you don't seem very happy lately. Is there anything you want to talk about with Mom and Dad? No matter what happens, we will be here with you."
- Listening is more important than lecturing. If the child is willing to open up, listen carefully without interrupting, judging, or rushing to offer solutions. Make them feel understood and accepted.

**Gain a Deep Understanding of the Situation**

- Communicate patiently with the child and their teachers to learn about the child's performance and social interactions at school, and check for specific stressors (such as bullying, learning difficulties, conflicts with teachers).

**Seek Professional Evaluation (a key step)**

- If these symptoms persist for more than two weeks and significantly affect the child's daily life, learning, and social interactions, be sure to seek professional help.
- You can take the child to see a child psychiatrist or psychologist/counselor. Through professional assessment tools and interviews, they can make an accurate judgment to distinguish whether this is a temporary psychological distress or a more specific clinical issue.
- Seeing a doctor is not about "labeling" the child, but about understanding them and providing the most scientific help—just as it is normal to see a doctor for a cold.

**Adjust the Family Atmosphere and Expectations**

- Temporarily reduce rigid academic performance requirements to alleviate the child's pressure. Focus more on the child's emotional and mental health.
- Ensure the family is a warm, supportive, and safe haven. Avoid arguing in front of the child and provide them with unconditional love and support.

**Summary**

"Being quiet, irritable, and school-avoidant" are "help signals" from the child. While they do not necessarily indicate a severe mental illness, they strongly suggest the child is experiencing emotional or psychological distress.

Your response is crucial:

- Priority Action: Provide care, listening, and support rather than criticism and coercion.
- Key Decision: If symptoms persist, resolutely seek professional help from a child psychology or psychiatry specialist for a scientific evaluation.
- Core Goal: Identify the root cause behind the behaviors and help the child get through difficult times, rather than merely eliminating surface behaviors.

Early detection and active intervention are vital for the child's healthy growth.

If needed, I can provide you with a conversation script for communicating with the child, or explain in more detail how to select professional psychological resources.

**Q45. Can psychiatric medications be taken during pregnancy or while preparing for pregnancy?**

This is an extremely important and complex question. The core answer is: There is no one-size-fits-all solution. Whether to take medication requires a joint assessment by a psychiatrist and an obstetrician, who will make an individualized decision after weighing the risks to both the mother and the fetus. Under no circumstances should you stop or switch medications on your own.

Below is a detailed analysis of the pros and cons, risks, and considerations.

**Core Principle: Risk-Benefit Balance**

The core of the decision lies in weighing the risks of two aspects:

- Potential risks of medication: Adverse effects on fetal development (such as teratogenic risks, neonatal adaptation syndrome, etc.).
- Risks of untreated mental illness: Depression, anxiety, insomnia, mental stress, etc., can cause substantial harm to both the pregnant woman and the fetus.

The harm of untreated mental illness in the mother to the fetus may far outweigh the risks of medication used under professional evaluation.

**Overview of Risk Levels of Various Medications (for reference only, not medical advice)**

| **Medication Class** | **Representative Drugs** | **Pregnancy Risk Profile (FDA's old classification*)** | **Notes** |
| --- | --- | --- | --- |
| Antidepressants | SSRIs (Sertraline, Escitalopram) | C/D | Relatively the safest. Sertraline is usually the first-line choice. Use in the third trimester may be associated with temporary neonatal symptoms such as rapid breathing and irritability. |
|  | SNRIs (Venlafaxine, Duloxetine) | C | Similar risks to SSRIs, with slightly less data available. |
|  | Tricyclics (Amitriptyline, Nortriptyline) | C/D | Has extensive historical data but more side effects; now rarely used as first-line treatment. |
| Mood Stabilizers | Lithium | D | Teratogenic risk (mainly cardiac defects) in the first trimester, but risk decreases in the second and third trimesters. High risk of relapse if discontinued; blood lithium levels need close monitoring. |
|  | Valproate | X/D | Extremely high risk. Teratogenicity and adverse effects on fetal neurocognitive development (such as reduced IQ, increased risk of autism). Should be avoided during pregnancy if possible. |
|  | Carbamazepine | D | Teratogenic risk (neural tube defects). |
|  | Lamotrigine | C | Relatively safer mood stabilizer, but dosage adjustments may be needed. |
| Antipsychotics | Second-generation (Olanzapine, Quetiapine) | C | Increasing data available; relatively safe. However, caution is needed regarding metabolic side effects (gestational diabetes, weight gain). |
|  | First-generation (Haloperidol) | C | Has a long history of use and is considered one of the optional medications. |
| Anxiolytics | Benzodiazepines (Lorazepam, Diazepam) | D | May increase the risk of cleft lip/palate in the first trimester; may cause neonatal sedation and withdrawal syndrome in the third trimester. Should be used short-term, at low doses, or avoided. |

*Note: The FDA has eliminated the letter classification system and adopted more detailed descriptive labeling, but the old classification is still commonly referenced in clinical practice.

**Key Recommendations and Action Plan**

If you are preparing for pregnancy or are already pregnant, follow these steps:

**Planning for Pregnancy is the Golden Opportunity**

- Discuss your pregnancy plans with your psychiatrist before attempting to conceive.
- The doctor may adjust your treatment plan based on your situation, such as switching to a safer medication, reducing to the minimum effective dose, or even attempting a gradual dose reduction if your condition is stable.

**What to Do If You Become Pregnant Suddenly? Never Stop Medication on Your Own!**

- Immediately contact your psychiatrist and obstetrician.
- Suddenly stopping medication without medical supervision can lead to a sharp relapse or severe withdrawal reactions, which are more harmful to both you and the fetus.

**Multidisciplinary Team Collaboration**

- Your treatment team should include: a psychiatrist, an obstetrician (preferably one with experience in high-risk obstetrics), a pediatrician, as well as yourself and your family.
- Collaboratively develop a comprehensive treatment, monitoring, and delivery plan for the entire pregnancy.

**Strengthen Non-Pharmacological Treatments**

- Under the guidance of a doctor, psychotherapy (such as Cognitive Behavioral Therapy, CBT) is a very safe and effective auxiliary method during pregnancy, and can sometimes reduce the required medication dosage.

**Pregnancy Monitoring**

- If you are taking medications with specific risks, your doctor may recommend more detailed fetal ultrasound examinations (such as fetal echocardiography) to closely monitor the baby's development.

**Summary**

Yes, but it must be done under the close guidance and monitoring of professional doctors.

The primary goal is to maintain the mother's mental health and stability, as a healthy, emotionally stable mother provides the best environment for the fetus's healthy growth. Communicate all your concerns openly and honestly with your doctors to jointly make the most beneficial decision for you and your baby.

**Q46. Can psychiatric medications be taken concurrently with other medications?**

This is an extremely important and common question. The simple and direct answer is: Yes, but it must be done under the close guidance and supervision of a doctor.

When psychiatric medications are taken concurrently with other drugs (including prescription drugs, over-the-counter drugs, traditional Chinese medicine, and health supplements), complex interactions may occur. Taking them without professional guidance is very dangerous.

When combining psychiatric medications with other drugs, the following key aspects need to be considered:

**Pharmacodynamic Interactions: Mutual Effects of Drugs in the Body**

- Synergism (potentiation): Enhanced drug effects, which may lead to toxicity. For example, combining sedative medications (such as benzodiazepines) with opioid painkillers or alcohol can significantly enhance central nervous system depression, posing a life-threatening risk of respiratory depression.
- Antagonism (inhibition): One drug reduces or inactivates the effect of another. For example, certain anti-Parkinson's drugs may weaken the effects of antipsychotic medications.

**Pharmacokinetic Interactions: One Drug Affects the Absorption, Distribution, Metabolism, or Excretion of Another**

- The most important is liver metabolism: Many psychiatric medications are metabolized by the cytochrome P450 enzyme system in the liver. Certain drugs (such as Fluoxetine, Paroxetine, Haloperidol, etc.) can inhibit or induce the activity of these enzymes, thereby dramatically increasing or decreasing the blood concentration of co-administered drugs.
  - Increased concentration: Leads to excessive efficacy and a significantly higher risk of side effects.
  - Decreased concentration: Results in insufficient efficacy and disease recurrence.

**Side Effect Overlapping**

When two drugs with similar side effects are taken together, the incidence and severity of side effects can increase significantly.

- For example, both tricyclic antidepressants and certain antipsychotics have sedative and anticholinergic side effects (such as dry mouth, constipation, blurred vision, difficulty urinating), which can be exacerbated when used together.
- Many psychiatric medications and antihypertensive drugs can cause orthostatic hypotension (dizziness when standing up suddenly), and the risk is higher when used concurrently.

**Examples of Common Combinations Requiring High Alert**

| **Psychiatric Medication Class** | **Drugs Requiring Caution When Combined** | **Potential Risks** |
| --- | --- | --- |
| SSRI Antidepressants (e.g., Fluoxetine, Paroxetine) | Non-steroidal anti-inflammatory drugs (NSAIDs, such as Ibuprofen, Aspirin) | Increased risk of gastrointestinal bleeding |
|  | Statin lipid-lowering drugs (e.g., Simvastatin) | Increased risk of muscle soreness and rhabdomyolysis |
|  | St. John's Wort (a herbal supplement) | Risk of serotonin syndrome |
| MAOI Antidepressants (e.g., Phenelzine) | Tyramine-containing foods (cheese, cured meats, etc.), decongestants | Life-threatening hypertensive crisis |
| Benzodiazepines (e.g., Alprazolam, Diazepam) | Alcohol, opioid painkillers, sleeping pills | Severe sedation, respiratory depression, coma, death |
| Tricyclic Antidepressants | Antiarrhythmic drugs (e.g., Quinidine) | Increased risk of cardiac toxicity (QT interval prolongation) |
| Lithium (Mood Stabilizer) | Diuretics, NSAIDs | Increased lithium concentration, leading to lithium toxicity (tremors, confusion) |

**Safety Recommendations for Patients**

- Inform all doctors: When seeing any doctor (such as an internist, dentist), you must proactively and fully disclose all psychiatric medications, chronic disease medications, and even health supplements and herbal medicines you are taking.
- Use a fixed pharmacy: Try to pick up all your medications from the same pharmacy. Pharmacists are an important line of defense in identifying potential drug interactions.
- Never self-medicate: Do not purchase over-the-counter drugs or traditional Chinese medicine without consulting your psychiatrist or pharmacist first.
- Create a medication list: Carry a detailed list of your medications (including names, dosages, and administration methods) with you for emergencies.
- Closely observe reactions: After starting a new concurrent medication under medical guidance, closely monitor for any abnormal reactions (such as dizziness, drowsiness, nausea, palpitations) and promptly report them to your doctor.

**Summary**

Combining psychiatric medications with other drugs is a common clinical practice, but it is a complex "technical task" that should never be taken for granted. The only safe approach is to do so under the professional assessment and monitoring of a doctor who is familiar with your complete medical history and medication regimen.

If you have specific medication combinations to consult about, be sure to bring all your medication packaging to your doctor or pharmacist.

**Q47. Can I drink alcohol, coffee, or strong tea while taking psychiatric medications?**

This is an extremely important and common question. The simple and direct answer is: Strongly not recommended, and in most cases, should be strictly prohibited.

Drinking alcohol, coffee, or strong tea while taking psychiatric medications may pose serious health risks and side effects. Below is a detailed explanation of the reasons and recommendations.

**1. Alcohol (Alcoholic Beverages)**

Core Conclusion: Strictly prohibited.

- Exacerbates side effects: Both alcohol and psychiatric medications (such as antidepressants, anxiolytics, sedative-hypnotics, antipsychotics, etc.) inhibit the central nervous system. Taking them together produces a "synergistic inhibitory" effect, severely amplifying side effects such as drowsiness, dizziness, slowed reaction times, and respiratory depression—this is very dangerous.
- Impairs efficacy: Alcohol interferes with the metabolism of medications in the liver, which may cause blood drug concentrations to be too high (increasing toxicity) or too low (reducing efficacy), leading to disease recurrence or exacerbation.
- Worsens mood: Alcohol itself is a depressant. While it may temporarily make people feel relaxed, long-term use can exacerbate depression and anxiety—contradicting the treatment goals.
- Extremely dangerous combinations: Combining certain medications (such as monoamine oxidase inhibitor antidepressants) with alcohol (especially tyramine in red wine and beer) may trigger a hypertensive crisis, which is life-threatening.

Recommendation: Completely abstain from alcohol during treatment.

**2. Coffee (Caffeine)**

Core Conclusion: Exercise extreme caution; preferably avoid or strictly limit.

- Antagonistic effect: Caffeine is a central nervous system stimulant, while many anxiolytics (such as benzodiazepines) are sedatives. Taking them together is like a "tug of war," mutually canceling out effects. You may not feel the sedative effect of the medication, and the stimulant effect of coffee may also be diminished—instead, you may feel more flustered and anxious.
- Exacerbates anxiety and insomnia: Caffeine itself can cause palpitations, nervousness, anxiety, and insomnia—symptoms that are the core issues many mental health conditions aim to treat. Consuming caffeine may directly worsen your condition.
- Affects drug metabolism: Caffeine may interfere with the metabolic processes of certain medications in the body.

Recommendations:

- If you have an anxiety disorder, panic disorder, or sleep disorder, it is best to avoid coffee entirely.
- If you really want to drink coffee, be sure to consume a small amount (e.g., one small cup of weak coffee) and closely observe your physical and emotional reactions.
- Ideally, separate coffee consumption from medication intake by 2-3 hours.

**3. Strong Tea**

Core Conclusion: Similar to coffee—exercise extreme caution.

Strong tea also contains high levels of caffeine and theophylline, which are also central nervous system stimulants. Therefore, it poses similar problems to coffee:

- May counteract the effects of sedative medications.
- Theophylline and caffeine can cause increased heart rate and restlessness, exacerbating anxiety symptoms.
- May affect sleep quality.

Recommendations:

- Try to avoid drinking strong tea.
- You can choose weak tea or decaffeinated tea as appropriate.
- Similarly, it is recommended to separate tea consumption from medication intake.

**Summary and Core Recommendations**

| **Beverage** | **Recommended?** | **Main Reasons** | **Recommendations** |
| --- | --- | --- | --- |
| Alcohol (Alcoholic Beverages) | Strictly prohibited | Severely exacerbates side effects, impairs efficacy, worsens mood, and poses life-threatening risks | Completely abstain from alcohol during treatment |
| Coffee | Exercise extreme caution; preferably avoid | May counteract efficacy and directly worsen anxiety and insomnia symptoms | If consumed, ensure it is a small amount, weak coffee, and separate from medication intake |
| Strong Tea | Exercise extreme caution; preferably avoid | Similar to coffee—contains caffeine and theophylline, which can cause palpitations and anxiety | Switch to weak tea or herbal tea, and separate from medication intake |

**Most Important Principle:**

Be sure to consult your attending doctor or pharmacist about this matter. The intensity of interactions between different types of psychiatric medications and these beverages varies. Your doctor is most familiar with your condition and the characteristics of the medications you are taking, and can provide the most individualized and safe advice.

Your health is the top priority. Temporarily adjusting your dietary habits during treatment is for a faster and safer recovery.

**Q48. Are there any dietary restrictions for patients with mental illness while taking medication?**

This is an extremely important question! For patients with mental illness, dietary cooperation during medication is crucial for the effectiveness of treatment and the reduction of side effects. Different medications have different restrictions and precautions.

Below are the main dietary restrictions and considerations for different classes of psychiatric medications:

**Core General Principles**

- Strictly follow medical advice: The information below is for general popular science purposes; always adhere to the specific guidance of your attending doctor and pharmacist.
- Avoid alcohol: This is the most important and universal restriction. Alcohol can interact with the vast majority of psychiatric medications, potentially enhancing sedation (leading to dangerous coma), impairing efficacy, increasing liver metabolic burden, and even triggering severe adverse reactions.
- Use caffeinated beverages with caution: Caffeine in coffee, strong tea, energy drinks, etc., may exacerbate side effects such as anxiety, insomnia, palpitations, and restlessness—especially when taking anxiolytics or certain antidepressants.

**Dietary Restrictions by Medication Class**

**1. Monoamine Oxidase Inhibitors (MAOIs)**

- Representative drugs: Phenelzine, Tranylcypromine, etc. (currently less commonly used as first-line treatment but still in use)
- This class of drugs has the strictest dietary restrictions! During treatment, foods high in tyramine must be avoided, as they may trigger a hypertensive crisis (characterized by severe headache, vomiting, and a sharp rise in blood pressure—life-threatening).

**Absolutely Prohibited (High-Tyramine Foods):**

- Fermented and pickled foods: Cheese (especially aged cheese), yogurt, sour cream, fermented sausages (such as salami), pepperoni, kimchi, fermented tofu, soybean paste, preserved tofu.
- Alcoholic beverages: All beer, red wine, spirits—especially aged wine and improperly stored alcohol.
- Meats: Cured or air-dried meats, animal liver, spoiled meat.
- Fish: Cured, air-dried, or smoked fish (such as salted fish, caviar).
- Fruits: Overripe fruits (such as bananas, avocados), figs.
- Legumes: Fermented soy products (such as miso soup, fermented tofu), lentils.
- Others: Yeast extract (such as Marmite/Vegemite), large amounts of chocolate, certain beers and red wines.

**2. Tricyclic Antidepressants (TCAs)**

- Representative drugs: Amitriptyline, Doxepin, Clomipramine, etc.
- Avoid alcohol: Significantly enhances the sedative effects and central nervous system depression of the medication.
- Avoid grapefruit and grapefruit juice: Grapefruit inhibits the enzyme (CYP3A4) that metabolizes this class of drugs in the intestines, leading to abnormally high blood concentrations of the medication and an increased risk of side effects (such as dry mouth, constipation, dizziness, arrhythmia).
- Drink plenty of water and increase dietary fiber intake: This class of drugs has strong anticholinergic side effects, which can easily cause constipation and dry mouth. Eating more fruits, vegetables, and drinking plenty of water helps alleviate these symptoms.

**3. New-Generation Antidepressants (SSRIs/SNRIs, etc.)**

- Representative drugs: Fluoxetine, Paroxetine, Sertraline, Venlafaxine, etc.
- Avoid alcohol: Increases nervous system side effects (such as dizziness, drowsiness) and may exacerbate depressive and anxious moods.
- Use caffeine with caution: These medications themselves may cause anxiety, insomnia, and akathisia—caffeine can worsen these symptoms.
- Be aware of interactions with St. John's Wort (Hypericum perforatum): This is a herbal supplement commonly used to improve mood. It can reduce blood concentrations of SSRIs and other medications, impairing efficacy, while significantly increasing the risk of serotonin syndrome (a life-threatening severe reaction).

**4. Antipsychotic Medications**

- Representative drugs: Olanzapine, Quetiapine, Risperidone, Clozapine, etc.
- Control diet to avoid weight gain: Many atypical antipsychotic medications can significantly increase appetite, lead to weight gain, and elevate blood sugar and lipid levels. During medication:
  - Limit intake of high-calorie, high-sugar, and high-fat foods.
  - Establish regular eating habits and consume more healthy foods such as vegetables and whole grains.
  - Regularly monitor weight, blood sugar, and lipid levels.
- Avoid alcohol and caffeine: For the reasons mentioned above.
- Avoid overheating or strenuous exercise: Some medications may affect temperature regulation; excessive sweating in hot environments can lead to dehydration—be sure to replenish fluids.

**5. Mood Stabilizers (Lithium)**

- Representative drug: Lithium Carbonate
- Maintain a stable sodium intake: Lithium metabolism is closely related to sodium levels in the body. Suddenly reducing salt intake (such as switching to a strict low-sodium diet) or dehydration caused by excessive sweating or diarrhea can increase blood lithium concentrations, easily triggering lithium toxicity (characterized by severe vomiting, diarrhea, hand tremors, drowsiness, confusion, etc.).
  - Remember: Maintain your normal, stable daily salt intake—do not fluctuate drastically.
  - Replenish fluids and salt promptly after excessive sweating, fever, or diarrhea.
- Limit caffeine intake: Caffeine has a diuretic effect, which may affect lithium excretion and blood concentrations.

**6. Anxiolytics and Sedative-Hypnotics (Benzodiazepines)**

- Representative drugs: Diazepam, Alprazolam, Lorazepam, etc.
- Strictly avoid alcohol: Alcohol produces a synergistic inhibitory effect with this class of drugs, severely suppressing breathing and heart rate—possibly leading to coma or even death. This is one of the most dangerous combinations.
- Avoid caffeine: The stimulant effect of caffeine directly counteracts the anxiolytic and sedative effects of the medication.

**Summary and Recommendations**

- Primary principle: Alcohol is the "natural enemy" of almost all psychiatric medications—be sure to abstain.
- Consult your doctor: When receiving a new medication, proactively ask your doctor or pharmacist: "Are there any special dietary precautions or restrictions I need to follow while taking this medication?"
- Read the medication instructions: Carefully read the "Drug Interactions" and "Precautions" sections of the medication leaflet.
- Balanced nutrition: While avoiding prohibited foods, maintaining a balanced, healthy diet helps with overall physical recovery and counteracts certain metabolic side effects of medications (such as weight gain).
- Drink plenty of water: Ensure adequate fluid intake to promote drug metabolism and excretion, and alleviate certain side effects (such as dry mouth, constipation).

Note: The above content is for popular science purposes and cannot replace professional medical advice. Always take medications as prescribed by your doctor.

**LLM Model:Gemini 3.0 Pro**

**Q1. What is a mental illness?**

Mental Illness, also known as Mental Health Disorder, refers to a range of health conditions that affect an individual's thinking, emotions, feelings, or behavior.

These illnesses can cause distress to a person and lead to functional difficulties in important life areas such as social interactions, work, and family life.

Core characteristics include:

Wide-ranging impact: It can affect anyone, regardless of age, gender, ethnicity, or socioeconomic status.

Functional impairment: Patients face significant difficulties in coping with daily life and interpersonal relationships.

More than just a bad mood: It is different from temporary sadness or stress. Symptoms of mental illness are more persistent and severe, and significantly affect daily life.

Diverse types: There are many types of mental illnesses, with common ones including:

Depression: Persistent sadness and loss of interest in activities.

Anxiety Disorders: Excessive worry, fear, and panic, such as generalized anxiety disorder and social anxiety disorder.

Bipolar Disorder: Mood swings between the two extremes of mania and depression.

Schizophrenia: Disordered thinking and perception, which may involve hallucinations or delusions.

Eating Disorders: Such as anorexia nervosa and bulimia nervosa.

Post-Traumatic Stress Disorder (PTSD).

An important point:

Mental illness is a medical condition, not a sign of character flaw or personal weakness. It is usually the result of the interaction of multiple factors such as genetics, biology, environment, and psychology.

The good news is that most mental illnesses are treatable. Through psychotherapy, medication, lifestyle changes, or a comprehensive approach, patients can effectively manage symptoms, restore normal functioning, and live meaningful lives. If you or someone you know is struggling with this, seeking professional help is the crucial first step.



**Q2. What are the common types of mental illnesses?**

Hello, mental illnesses (also known as mental health disorders) cover a wide range and refer to a series of conditions that affect your emotions, thinking, and behavior. These illnesses can cause you distress in daily life and lead to difficulties in social, work, or family activities.

Here are some common types of mental illnesses, explained with classifications and specific examples:

1. Anxiety Disorders

This is the most common type of mental illness. It is characterized by persistent and intense anxiety, fear, or worry about certain things or situations.

Generalized Anxiety Disorder (GAD): Excessive and uncontrollable worry about various daily events or activities (such as work, health, family).

Panic Disorder: Recurrent sudden and intense feelings of fear (panic attacks), accompanied by physical symptoms such as palpitations, sweating, trembling, and difficulty breathing.

Social Anxiety Disorder: Extreme fear and anxiety in social situations, fear of being judged or evaluated by others.

Specific Phobias: Irrational and intense fear of specific objects or situations (such as heights, flying, animals).

2. Mood Disorders

Mainly characterized by significant and persistent disturbances in mood, including low mood or elevated mood.

Depressive Disorders: Mainly characterized by persistent sadness, emptiness, and loss of interest or pleasure in activities. Major Depressive Disorder is the most important type among them.

Bipolar and Related Disorders: Patients swing between two extreme emotional states: manic/hypomanic episodes with abnormally elevated mood and high energy, and depressive episodes with extremely low mood.

3. Schizophrenia Spectrum and Other Psychotic Disorders

Such illnesses are characterized by severe distortions of thought and perception, and patients may lose contact with reality.

Schizophrenia: A serious mental illness whose symptoms include hallucinations (hearing or seeing things that do not exist), delusions (firmly believing in ideas that do not conform to facts), disorganized thinking, and speech disturbances.

4. Obsessive-Compulsive and Related Disorders

Characterized by persistent, intrusive thoughts (obsessions) and/or repetitive behaviors (compulsions).

Obsessive-Compulsive Disorder (OCD): Patients are troubled by unwanted thoughts and fears (obsessions), which drive them to perform repetitive behaviors (compulsions) in an attempt to relieve anxiety.

5. Trauma- and Stressor-Related Disorders

Mental health problems that occur after experiencing or witnessing a traumatic or stressful event.

Post-Traumatic Stress Disorder (PTSD): After experiencing or witnessing a terrifying event, flashbacks, nightmares, severe anxiety, and uncontrollable memories of the event may occur.

6. Feeding and Eating Disorders

Characterized by unhealthy eating behaviors, excessive focus on weight, and distorted perception of body shape, which seriously affect physical health and psychological function.

Anorexia Nervosa: Extreme fear of weight gain, strictly restricting calorie intake through excessive dieting, exercise, etc., leading to a body weight significantly lower than the normal level.

Bulimia Nervosa: Recurrent episodes of binge eating, followed by inappropriate compensatory behaviors (such as vomiting, abuse of laxatives, excessive exercise).

7. Personality Disorders

Characterized by persistent and rigid patterns of thinking, emotion, and behavior that significantly deviate from cultural expectations and lead to impaired social functioning or personal distress.

Borderline Personality Disorder (BPD): Characterized by instability in emotions, interpersonal relationships, and self-image, often accompanied by impulsive behaviors.

Important Notes:

Complexity: This is a complex field, and symptoms of many illnesses may overlap.

Not a diagnostic tool: The above list is only a common classification and does not include all mental illnesses, so it cannot be used as a basis for self-diagnosis.

Seek professional help: If you or someone you know is experiencing mental health distress, it is crucial to seek help from a professional psychologist or psychiatrist. They can provide an accurate diagnosis and a personalized treatment plan.



**Q3. Is anxiety disorder a type of mental illness?**

Yes, Anxiety Disorder is a type of mental illness.

More precisely, it is clinically classified as a "Mental Disorder".

Here are some key explanations:

Authoritative classification: The two most authoritative international diagnostic criteria—the American Psychiatric Association's Diagnostic and Statistical Manual of Mental Disorders (DSM-5) and the World Health Organization's International Classification of Diseases (ICD-11)—clearly list anxiety disorders as a major category of mental disorders.

Distinction from normal anxiety: Everyone feels anxious, which is a normal emotional response. But anxiety disorder is different. It is characterized by excessive, persistent, and intense worry and fear. This emotional response is disproportionate to the actual situation and will seriously affect the patient's daily life, work, social interactions, and physical and mental health.

Common types of anxiety disorders: Anxiety disorder is not a single illness; it includes multiple types, such as:

Generalized Anxiety Disorder (GAD): Excessive worry about various things.

Panic Disorder: Recurrent sudden and intense feelings of fear (panic attacks).

Social Anxiety Disorder: Intense fear and avoidance in social situations.

Specific Phobias: Intense fear of specific objects or situations (such as fear of heights, fear of spiders).

Treatable: A very important point is that anxiety disorder, like many other illnesses (such as diabetes, hypertension), is a disease that can be effectively treated. Through professional psychotherapy (such as Cognitive Behavioral Therapy, CBT), medication, or a combination of both, the symptoms of most patients can be significantly improved, and they can return to normal life.

Treating anxiety disorder as a mental illness helps us correctly understand it, eliminate stigma, and encourage those in need to seek professional diagnosis and treatment.



**Q4. Is depression a type of mental illness?**

Yes, Major Depressive Disorder is a type of mental illness.

It is more than just ordinary low mood or temporary sadness; it is a complex disease that continuously affects an individual's emotions, thinking patterns, and physical health. Its main characteristics include:

Persistent low mood: Feeling sad, empty, or hopeless.

Loss of interest: Losing interest or pleasure in previously enjoyed activities.

Impaired cognitive function: Difficulty concentrating, memory loss, and difficulty making decisions.

Physical symptoms: Significant changes in appetite (increase or decrease), sleep disorders (insomnia or hypersomnia), and persistent fatigue.

Negative thinking: Feeling worthless, excessive self-blame or guilt, and even having thoughts of death or suicide.

Like colds and diabetes, depression is a disease that requires professional medical help. It is not a sign of weakness of character or lack of willpower. Through timely psychotherapy, medication, or a combination of both, depression can be effectively controlled and recovered.

If you or someone around you is experiencing related distress, please be sure to seek help from a professional doctor or mental health expert.



**Q5. What are the main causes of mental illness?**

The factors leading to mental illness are very complex. They are usually not caused by a single reason, but the result of the interaction of biological, psychological, social, and environmental factors. We can understand it from the following main aspects:

1. Biological Factors

Genetics: Many mental illnesses tend to cluster in families. If a close relative (such as a parent or sibling) of a person has a certain mental illness, his/her risk of developing the same illness will be relatively higher. But this does not mean that he/she will definitely get sick; it only means that genetics is an important risk factor.

Imbalance of brain chemicals: Neurotransmitters in the brain (such as serotonin, dopamine) are chemical substances responsible for transmitting signals. Imbalances of these chemicals are believed to be related to many mental illnesses. For example, depression and anxiety disorders are usually associated with low levels of serotonin.

Abnormalities in brain structure and function: The specific areas of the brain of patients with certain mental illnesses may be structurally or functionally different from those of normal people. For example, brain scans of patients with schizophrenia may sometimes show structural changes such as enlarged brain ventricles.

Perinatal damage or infection: Viral infections, malnutrition, stress during pregnancy, or complications during childbirth (such as neonatal hypoxia) may increase the risk of the child developing mental illness in the future.

2. Psychological Factors

Childhood traumatic experiences: Negative experiences in childhood, such as emotional neglect, physical or sexual abuse, school bullying, family violence, etc., are powerful risk factors for various mental illnesses. These traumas will profoundly affect an individual's personality development, emotional regulation ability, and ways of coping with stress.

Coping styles and personality traits: Certain personality traits, such as excessive pursuit of perfection, low self-esteem, neuroticism (tendency to feel anxious and worried), and poor ability to cope with stress, may also make people more likely to develop mental illness.

Thinking patterns: Negative, pessimistic, or rigid thinking patterns, such as the tendency to catastrophize problems, may also increase the risk of depression and anxiety disorders.

3. Social and Environmental Factors

Major life stress events: Sudden or persistent great pressure is an important factor inducing mental illness. For example, the death of a loved one, unemployment, divorce, severe financial difficulties, diagnosis of a serious physical illness, etc.

Social support system: Lack of effective support from family, friends, and the community, feeling lonely and isolated, will significantly increase the risk of mental health problems. A strong social support network is an important protective factor against mental illness.

Substance abuse: Abuse of alcohol and drugs will directly affect brain function, which may not only induce psychotic symptoms but also often coexist with diseases such as depression and anxiety (comorbidity).

Socio-cultural factors: People living in environments of poverty, discrimination, violence, or social unrest face higher risks to their mental health.

In summary, the occurrence of mental illness is the result of the interaction of the "Biopsychosocial" model. Biological factors such as genetics and brain structure may determine whether a person is "vulnerable", while psychological trauma and stress events in real life may be the "Trigger", ultimately leading to the onset of the disease.

Everyone's situation is unique, and the specific factors and weights leading to the disease are also different. If you or someone around you is experiencing mental health distress, it is very important to seek help from a professional psychological counselor or psychiatrist.



**Q6. Can insomnia or long-term staying up late increase the risk of mental illness?**

The answer is yes. A large number of scientific studies and clinical evidence show that insomnia or long-term staying up late (i.e., chronic sleep deprivation) will significantly increase the risk of developing various mental illnesses and may worsen existing mental health problems.

This relationship is complex and is usually described as a vicious circle of two-way relationship:

Sleep problems trigger mental illness: Lack of sleep will directly affect brain function, thereby increasing the vulnerability to mental illness.

Mental illness causes sleep problems: Core symptoms of many mental illnesses include insomnia, disrupted sleep patterns, etc.

Why does lack of sleep increase the risk of mental illness?

Our brain performs a series of key "maintenance" work during sleep. Long-term lack of sleep will interrupt these processes, leading to the following problems:

Impaired Emotional Regulation Ability

Overactivity of the amygdala: The amygdala is the emotional center of the brain (especially for fear and anxiety). When sleep-deprived, it becomes abnormally active, making you more sensitive to negative events and more likely to feel anxious, angry, and sad.

Weakened function of the prefrontal cortex: The prefrontal cortex is responsible for rational thinking, decision-making, and impulse control, and it can "brake" the excessive reaction of the amygdala. Lack of sleep will weaken its function, making it more difficult for you to control your negative emotions. Simply put, the "emotional brake fails".

Disordered Stress System

Long-term lack of sleep will keep the body's stress system in a state of alert, leading to increased levels of "stress hormones" such as cortisol. This not only makes you feel nervous and restless but also damages brain neurons, which will increase the risk of depression and anxiety disorders in the long run.

Impaired Cognitive Function

Sleep is crucial for memory consolidation, attention, and problem-solving abilities. Long-term staying up late will lead to slow thinking, difficulty concentrating, and decreased decision-making ability. These cognitive problems are stressors themselves and may exacerbate the cycle of negative thinking.

Impact on Neurotransmitters

Sleep affects the balance of key neurotransmitters such as serotonin (5-hydroxytryptamine) and dopamine, which are closely related to mood, motivation, and pleasure. Their imbalance is the biological basis of many mental illnesses such as depression and anxiety disorders.

Mental Illnesses Closely Related to Sleep Problems

Depression: This is one of the mental illnesses most closely related to sleep problems. About 80% of patients with depression have severe sleep disorders, especially early awakening (waking up in the early morning and being unable to fall asleep again). At the same time, people with long-term insomnia are several times more likely to develop depression than those with normal sleep.

Anxiety Disorders: Anxiety and insomnia often "go hand in hand". Anxiety's worries and racing thoughts make it difficult to fall asleep, and insomnia will exacerbate anxiety the next day, forming a vicious circle.

Bipolar Disorder: Changes in sleep patterns are a strong early warning signal for bipolar disorder episodes. During manic episodes, patients may not need to sleep for days and have high energy; while during depressive episodes, they may experience hypersomnia of staying in bed for a long time.

Post-Traumatic Stress Disorder (PTSD): Patients often cannot sleep soundly due to nightmares and hyperarousal.

Schizophrenia: Severe disruption of sleep rhythm is a common feature of schizophrenia.

Advice for You

If you are experiencing insomnia or distress due to long-term staying up late, please be sure to take it seriously, because it is more than just "not sleeping well".

Establish Good Sleep Hygiene Habits

Regular schedule: Try to go to bed and wake up at the same time every day, even on weekends.

Optimize the environment: Ensure the bedroom is dark, quiet, and cool.

Relax before bed: Avoid using electronic devices such as mobile phones and computers one hour before bed. You can try reading, listening to light music, taking a hot bath, or meditating.

Avoid stimulants: Avoid caffeine and alcohol in the afternoon and evening.

Stay Active During the Day

Expose yourself to natural light during the day, which helps regulate the biological clock.

Engage in regular physical exercise, but avoid strenuous exercise a few hours before bed.

Seek Professional Help in Time

If insomnia persists (for example, more than 3 nights a week for several weeks) and has affected your mood, work, and daily life, please consult a doctor or mental health expert in time.

Do not take sleeping pills casually by yourself; they should be used under the guidance of a doctor.

In short, placing high-quality sleep on an equal footing with a healthy diet and regular exercise is one of the most important steps in investing in physical and mental health.

**Q7. How to judge if you may have a mental illness?**

This is a very important question. Being aware that you may need help is the first step towards health. Please remember that self-judgment cannot replace professional medical diagnosis, but understanding some common signs can help you decide whether to seek professional help.

Core principle: The diagnosis of mental illness usually depends not only on "having a certain symptom" but also on the duration, severity of these symptoms, and their negative impact on your daily life functions (such as work, study, interpersonal relationships).

Here are some aspects you can observe. If you find that you have experienced significant and persistent changes in multiple aspects, then seeking professional evaluation is a wise choice.

1. Significant Changes in Emotions

This is the most easily detectable signal by yourself and others.

Persistent sadness or low mood: Feeling empty, hopeless, and losing interest in previously enjoyed activities (for example, for more than two weeks).

Extreme mood swings: Rapid fluctuations between extremely elevated mood (abnormally high energy, talkativeness, impulsivity) and extremely low mood.

Excessive worry or fear: Uncontrollable anxiety, panic about daily trivial matters, or feelings of dying or losing control.

Irritability or anger: Feeling irritable, frustrated for a long time, easily conflicting with others, and reactions beyond the normal range.

Emotional numbness: Feeling numb, not interested in anything, and emotionally distant from relatives and friends.

2. Difficulties in Thinking and Cognition

Difficulty concentrating: Hard to focus on work, study, or conversations, with confused or wandering thoughts.

Memory loss: Forgetting important things or having difficulty with daily memory.

Illogical or strange thoughts: Having paranoid thoughts (always thinking someone wants to harm you), unrealistic beliefs (such as thinking you have superpowers).

Difficulty making decisions: Feeling hesitant about simple daily decisions.

Extremely low self-evaluation: Persistently feeling worthless, full of guilt, and engaging in harsh self-criticism.

3. Changes in Behavior

Social withdrawal: Taking the initiative to avoid social occasions, preferring to be alone, and interrupting contact with friends and family.

Changes in sleep patterns: Severe insomnia (difficulty falling asleep, easy awakening at night, early awakening) or excessive sleep (always wanting to sleep, unable to wake up).

Changes in eating habits: Significant decrease or binge eating, leading to obvious weight changes.

Changes in energy levels: Feeling abnormally fatigued, not having the energy to do anything; or having excess energy and being restless.

Changes in coping styles: Starting to rely on alcohol, drugs, or other substances to cope with emotions.

Engaging in dangerous or impulsive behaviors: Such as reckless driving, reckless spending, unsafe sexual behavior, etc.

Talking about death or suicide: This is a very urgent signal that requires immediate help.

4. Physical Discomfort

Unexplained pain: Often feeling headaches, stomachaches, muscle aches, etc., but no cause can be found in physical examinations.

Rapid heartbeat, shortness of breath, dizziness, sweating: These may be symptoms of panic attacks.

Persistent fatigue: Feeling exhausted even with sufficient sleep.

What Should You Do If You Suspect You Have a Problem?

Seeking professional help is the key:

Psychiatrist: They are medical doctors who can make diagnoses, provide psychotherapy, and are the only professionals who can prescribe medications.

Therapist/Counselor: They provide talk therapy to help you understand and cope with your emotional, thinking, and behavioral patterns.

Where to find them? You can start with the "Psychiatry Department", "Psychology Department", or "Clinical Psychology Department" of general hospitals.

Communicate with someone you trust:

Tell your family or close friends how you feel. Obtaining their understanding and support is a form of healing in itself, and they can also help you find resources.

Do not stigmatize yourself:

Seeking help for mental health is as normal as seeing a doctor for a cold or getting a cast for a fracture. It does not mean you are "weak" or "flawed"; on the contrary, it is a responsible and brave act.

Please remember that you are not fighting alone. Many people will experience mental health challenges at some stage in their lives, and effective treatment and support can help you recover.

**Q8. Can self-assessment scales accurately judge whether you have a mental illness?**

The concise answer is: No.

Self-assessment scales (such as the Patient Health Questionnaire-9 for depression, Generalized Anxiety Disorder-7 for anxiety, etc.) cannot be used as an accurate basis for diagnosing mental illness. They are only screening tools, not diagnostic tools.

The difference is crucial and can be understood as follows:

A self-assessment scale is like a "thermometer": it can tell you that you may have a "fever" (i.e., you may have certain emotional distress or mental symptoms), but it cannot tell you whether the fever is caused by the flu, bacterial infection, or other more complex diseases.

A professional diagnosis is like a "doctor": The doctor will combine your "temperature" reading, conduct a detailed inquiry and examination, rule out other possibilities, and finally determine what illness you have and how to treat it.

Why Can't Self-Assessment Scales Be Used for Self-Diagnosis?

Complexity of diagnosis: The diagnosis of mental illness is a very rigorous and complex process that needs to be completed by a professionally trained psychiatrist or clinical psychologist. Professionals need to:

Conduct a detailed clinical interview: Fully understand your symptoms, medical history, personal growth experience, family situation, degree of social function impairment, etc.

Conduct differential diagnosis: Symptoms of many different mental illnesses may be similar, and it is also necessary to rule out mental symptoms caused by physical illnesses (such as thyroid problems, brain lesions, etc.).

Refer to diagnostic criteria: Conduct evaluations in strict accordance with the criteria in the internationally accepted Diagnostic and Statistical Manual of Mental Disorders (DSM-5) or International Classification of Diseases (ICD-11).

Limitations of scales:

Strong subjectivity: Emotions when filling out the scale and understanding of the questions may affect the accuracy of the results.

Unable to reflect the whole picture: Scales only focus on specific symptoms and cannot cover all complex situations of the individual.

May lead to misjudgment:

False positive: High score, but not meeting the diagnostic criteria for the disease, which may only be a temporary emotional fluctuation, causing unnecessary panic and anxiety.

False negative: Low score, but actually may have a certain atypical or complex mental disorder, which will delay the best time for treatment.

How Should We Correctly Use Self-Assessment Scales?

As a preliminary self-awareness tool: Help you better understand your recent emotional and psychological state and make vague "discomfort" more specific.

As a reference for seeking professional help: If you score high on the scale, or even if you score low but continue to feel distressed and it affects your normal life, this should be a clear "warning signal" indicating that you may need to seek professional help.

As an auxiliary material for communication with doctors: You can take the scale results to see the doctor, which can help you describe your distress to the doctor more systematically and allow the doctor to understand your general situation faster.

In short, please regard self-assessment scales as useful "signal lights" or "thermometers", but never as the final "judgment". If you have any doubts about your mental health, the most correct and responsible approach is to consult a professional psychiatrist or psychotherapist.

**Q9. Which department should you visit if you suspect you have a mental illness?**

If you suspect that you or someone else may have a mental illness, you should first choose to visit the Psychiatry Department (sometimes also called the "Psychological Medicine Department").

Why Choose the Psychiatry Department?

Professional diagnostic ability: Psychiatrists are medical professionals by training. They have the ability to make medical diagnoses of complex mental, psychological, and behavioral problems and rule out other physical illnesses that may cause these symptoms.

Prescribing authority: Only psychiatrists have the right to prescribe psychiatric medications. For many mental illnesses (such as depression, bipolar disorder, schizophrenia, etc.), medication is an important means to control symptoms and stabilize the condition.

Comprehensive treatment: Psychiatrists can provide comprehensive treatment plans including medication, physical therapy (such as transcranial magnetic stimulation), and psychological therapy recommendations.

Psychiatry vs. Psychological Counseling/Psychotherapy

Psychiatry: The core is "treating illnesses". Doctors mainly diagnose and treat mental illnesses through medical means (including medications).

Psychological Counseling/Psychotherapy: The core is "guidance". Psychological counselors or therapists mainly help clients solve psychological distress, improve emotions, and adjust cognitive and behavioral patterns through dialogue and behavioral training. They usually do not have prescribing authority.

Simple Selection Suggestions

Obvious symptoms affecting life: If you have severe emotional problems (such as persistent severe depression, strong suicidal thoughts), abnormal behaviors (such as hallucinations, delusions), or physiological function disorders (such as long-term insomnia, loss of appetite), prioritize the Psychiatry Department.

General psychological distress: If it is mainly troubles in life, work, interpersonal relationships, emotional stress, or personal growth issues, you can first consider psychological counseling.

Best Practice

Usually, the best way is for psychiatrists and psychotherapists to work together. Doctors are responsible for medications and condition control, and therapists are responsible for in-depth psychological guidance. After being diagnosed in the Psychiatry Department, you can decide whether you need to cooperate with psychological therapy according to the doctor's advice.

In short, if you suspect you have a mental illness, the first step to go to the Psychiatry Department of a general hospital to register is the most correct and reliable choice.

**Q10. How to distinguish between mental illness and other physical illnesses?**

This is a very important and profound question. In the past, people often strictly distinguished between mental illness and physical illness, but modern medical and scientific research shows that the boundary between the two is far more blurred than we think, and they are interrelated and mutually influential.

Simply put, we can understand their traditional differences and current connections from the following levels:

1. Different Main Manifestations (Traditional Distinction Method)

Mental Illness: Mainly affects an individual's thinking, emotions, perception, will, and behavior. These symptoms are usually internal and subjective, such as persistent sadness, inexplicable fear, hallucinations, delusions, social withdrawal, etc. It affects how a person perceives the world, thinks, and interacts with others.

Physical Illness: Mainly affects the function or structure of the body's organs, tissues, and systems. Symptoms are usually objective and measurable, such as fever, pain, tumors, fractures, elevated blood pressure, etc. It affects the physical operation of the body.

2. Different Focuses of Diagnostic Methods

Mental Illness: Diagnosis relies more on clinical observation, in-depth conversations with patients, and standardized assessment scales (such as DSM-5 or ICD-11). Doctors make diagnoses by collecting patients' descriptions of their own feelings and behaviors (subjective symptoms) and observing their external behaviors (objective signs). Although brain scans (such as fMRI) can show certain pattern differences, they cannot be used as routine diagnostic tools at present.

Physical Illness: Diagnosis relies more on objective biological indicators. For example, through blood tests, X-rays, CT scans, biopsies, etc., pathological changes of the body can be directly observed.

3. Cognitive Evolution of Physiological Basis (Key to Blurred Boundaries)

This is the core and most complex point in distinguishing the two. In the past, mental illness was considered a "disease of the mind" without a physiological basis, but now we clearly know:

All mental illnesses have their profound physiological (especially brain) basis.

Mental illness is a "disease of the brain": The brain is a physiological organ. The occurrence of mental illness is closely related to abnormalities in brain structure, imbalance of neurotransmitters (such as dopamine, serotonin), and abnormal function of neural circuits. For example, patients with depression may have low levels of serotonin in the brain, and patients with schizophrenia may have overactive dopamine systems in the brain.

Physical illness can cause mental problems: Many physical illnesses can directly or indirectly lead to mental symptoms. For example, hyperthyroidism may cause anxiety and irritability; chronic pain may lead to depression; patients after stroke may also have emotional and cognitive disorders.

Mental problems can cause physical symptoms: Mental stress and negative emotions can have a huge impact on the body through the neuro-endocrine-immune system. This is called "somatic symptoms". For example, long-term anxiety may lead to gastrointestinal discomfort (irritable bowel syndrome), headaches, palpitations, rashes, etc. The feeling of dying during a panic attack, with symptoms such as rapid heartbeat and difficulty breathing, are real physical reactions.

Summary and Comparison

To understand more clearly, we can summarize with a table:

Characteristics Mental Illness Physical Illness Intersection and Overlap Area

Main Manifestations Abnormal thinking, emotions, and behaviors Abnormal function or structure of body organs and tissues Mental illness is often accompanied by somatic symptoms (such as fatigue, pain); physical illness is also often accompanied by emotional problems (such as anxiety, depression).

Diagnostic Basis Mainly clinical interviews, behavioral observations, and psychological assessment scales Mainly objective indicators such as laboratory tests, imaging examinations, and pathological examinations Both need to combine the patient's subjective description and the doctor's objective observation.

Physiological Basis Abnormal structure and function of the brain, imbalance of neurotransmitters Pathological changes of various body organs (except the brain) The brain itself is part of the body. Mental illness is a disease that occurs in the complex organ of the brain.

Treatment Methods Psychological therapy, medication (regulating neurotransmitters), physical therapy (such as rTMS) Medication, surgery, radiotherapy and chemotherapy, rehabilitation training Many drugs (such as antidepressants) act on both the brain and the body; comprehensive treatment (treating both body and mind) has a better effect.

Social Cognition Often stigmatized and misunderstood as "weak willpower" More easily understood and accepted With the popularization of science, more and more people realize that mental illness, like other diseases, is a disease that needs treatment.

Conclusion

The core view of modern medicine is "Mind-Body Unity".

Distinguishing between mental illness and physical illness is more for the convenience of clinical diagnosis, classification, and selection of initial treatment plans. But in essence, mental illness is a type of physical illness, a disease specific to the complex organ of the brain.

Therefore, we should not oppose them. We should treat mental illness with a scientific and inclusive attitude to understand it, diagnose it, and actively treat it, just like treating hypertension and diabetes. Recognizing this is a key step in eliminating social prejudice and promoting mental health.

**Q11. Why do hospitals need to conduct multiple examinations for diagnosing mental illness?**

Okay, this is a very important and worthy question. The reason why hospitals need to conduct multiple examinations for diagnosing mental illness lies in the complexity of mental illness, the rigor of diagnosis, and the individual needs of treatment.

Simply put, unlike pneumonia (where infection can be seen through X-rays) or fractures (which are clearly visible on X-rays), mental illness does not have a single, objective biological indicator (Biomarker) that can directly confirm the diagnosis. It is a complex disease involving brain function, psychological state, behavioral performance, and social function.

Therefore, the purpose of multiple examinations is like a detective solving a case, needing to collect evidence from different angles, confirm each other, and finally piece together the complete truth. Specifically, there are the following main reasons:

1. Rule Out "Imposters": Conduct Differential Diagnosis

Many physical diseases, neurological diseases, and even substance abuse can present symptoms similar to mental illness. If these possibilities are not ruled out, misdiagnosis is very likely.

Physical diseases: For example, hypothyroidism may cause low mood, fatigue, and slow reaction, which looks like depression; while hyperthyroidism may cause tension, anxiety, irritability, and insomnia, which looks like anxiety disorder.

Neurological diseases: Brain tumors, epilepsy, encephalitis, Parkinson's disease, etc., may cause personality changes, hallucinations, delusions, or cognitive decline in the early stage.

Substance abuse: Abuse or withdrawal reactions of alcohol, drugs, or certain medications can also produce psychotic symptoms.

Therefore, examinations such as blood tests (such as thyroid function, liver and kidney function, electrolytes), electroencephalogram (EEG), and skull CT or MRI are mainly to rule out these "imposters" and ensure that the symptoms are indeed caused by mental and psychological problems.

2. Comprehensive Assessment of the Condition: Determine Diagnosis and Comorbidity

Mental illness itself often does not occur alone, and multiple diseases may coexist, which is called "comorbidity".

For example: A person may have both depression and anxiety disorder, or be mistaken for only depression during the depressive episode of bipolar disorder.

Assessment content: Doctors need to evaluate the patient's core symptoms, severity, course of development, and impact on social functions (work, study, interpersonal communication) through detailed conversations and professional psychological scales.

Psychiatric consultations, Mental Status Examinations (MSE), and various psychological assessment scales (such as Hamilton Depression/Anxiety Scale, Minnesota Multiphasic Personality Inventory, etc.) are used to accurately define the problem and determine which one or several mental disorders it is.

3. Assess Severity and Safety Risks

Diagnosis is not only about "labeling" but more importantly, understanding the severity of the disease, especially assessing the patient's risk of self-harm, suicide, or harm to others. This directly determines the urgency of the treatment plan and the intervention method (such as whether hospitalization is needed).

Psychiatrists will directly inquire about relevant thoughts and plans through in-depth clinical interviews, which is the most critical step in assessing safety risks.

4. Establish a Treatment Baseline

The results of various examinations (especially psychological scale scores and physiological indicators) can provide a "baseline level" for treatment. After the start of treatment, doctors can recheck these items regularly, and objectively evaluate the treatment effect by comparing changes in data before and after, and adjust the treatment plan accordingly (such as adjusting medication dosage or changing psychological treatment methods).

Summary

To summarize these examination items, it is like building a complete "evidence chain":

Examination Category Main Purpose Example of Examination Content

Clinical Interview Core of diagnosis: Understand the main complaint, medical history, personal history, family history Detailed conversation between doctor and patient/family members

Mental Status Examination (MSE) Assess the current mental state Observe appearance, behavior, speech, emotions, thinking, cognitive function, etc.

Psychological Measurement Quantitative assessment: Evaluate symptom severity and personality traits Self-Rating Depression Scale (SDS), Self-Rating Anxiety Scale (SAS), MMPI, etc.

Physical Examination Rule out physical diseases: Differential diagnosis Height, weight, blood pressure, heart rate, neurological examination, etc.

Laboratory Examination Rule out physical diseases: Differential diagnosis and assessment of physical condition Blood routine, liver and kidney function, thyroid function, blood sugar, electrolytes, etc.

Neuroimaging Examination Rule out organic brain lesions Skull CT, MRI, electroencephalogram (EEG), etc.

In short, conducting multiple examinations for mental illness is not excessive medical treatment, but a rigorous, scientific, and responsible performance of modern medicine. This comprehensive assessment method can maximize the avoidance of misdiagnosis and missed diagnosis, and formulate the safest, most effective, and personalized treatment plan for patients, ultimately helping them recover better.

**Q12. Is psychological assessment helpful for diagnosing mental illness?**

Of course it is helpful, and it is a very important auxiliary tool.

Psychological assessment plays a key role in the diagnosis of mental illness, but it should be clear that it cannot independently serve as the sole basis for the final diagnosis, but is part of the entire comprehensive assessment.

Simply put, if we compare the diagnosis process of a psychiatrist to solving a complex case, then psychological assessment is like the scientific investigation tools used by detectives (such as fingerprint analysis, DNA testing, etc.), which can provide very critical objective evidence and clues.

The following are the specific helps of psychological assessment in diagnosing mental illness:

1. Provide Objective and Quantitative Data

Unlike the relatively subjective communication interviews between doctors and patients, standardized psychological assessments (such as scales) can provide quantitative data.

Example: A person says "I feel very bad", which is a subjective description. But through the Beck Depression Inventory (BDI), the doctor can get a specific score, such as "25 points (moderate depression)", which makes the severity of symptoms objectively measurable and comparable.

2. Assist in Differential Diagnosis

Symptoms of many mental illnesses overlap and are easy to confuse. Psychological assessment can help distinguish between different diseases.

Example: Inattention may result from Attention Deficit Hyperactivity Disorder (ADHD), anxiety disorder, depression, or a certain stage of bipolar disorder. Through specific cognitive function tests and emotional assessments, doctors can better analyze the root cause and make a more accurate judgment.

3. Assess Specific Cognitive Functions

Some mental illnesses will significantly affect specific functions of the brain, such as memory, attention, executive function (planning, organizational ability), etc.

Example: For patients suspected of having early symptoms of Alzheimer's disease, neuropsychological tests can systematically assess their memory, language ability, and visual-spatial ability, providing important basis for early diagnosis.

4. Explore Personality Traits and Deep Psychological Conflicts

Some assessment tools (such as Minnesota Multiphasic Personality Inventory, projective tests, etc.) can reveal potential personality patterns, emotional regulation methods, and psychological conflicts that patients may not be aware of themselves. This is very valuable for understanding the causes of the disease and formulating personalized treatment plans.

5. Monitor Treatment Effect and Condition Changes

After the start of treatment, regular psychological assessments can objectively track the improvement of symptoms, evaluate whether the treatment plan is effective, and make timely adjustments.

Important Premises and Limitations

Cannot replace clinical interviews: The results of psychological assessment must be comprehensively interpreted by experienced psychiatrists or clinical psychologists in combination with detailed medical history, clinical observation, and in-depth interviews. Any assessment score divorced from the "person" is meaningless.

Results are affected by various factors: The patient's mood, fatigue level, motivation (whether they want to "pretend well" or "pretend bad"), cultural background, and educational level when filling out the scale may all affect the accuracy of the results.

Not a "gold standard": The diagnosis of mental illness does not have absolute biological indicators like measuring blood sugar or blood pressure. Psychological assessment is one of the most scientific auxiliary means currently, but not the only "confirmation" tool.

Summary

Psychological assessment is very helpful for diagnosing mental illness. It makes the diagnosis process more scientific, objective, and refined. It is not a magical test that can give a simple "yes" or "no" answer, but a powerful tool that provides decision support for doctors, deepens the understanding of patients, and makes treatment plans more targeted.

**Q13 What to Do If Different Psychiatrists Give Different Diagnoses?**

This is not an uncommon situation in clinical practice. If you encounter this, please do not be overly anxious or panicked. The diagnosis of mental health disorders is complex and nuanced. Different doctors may reach different conclusions based on their experience, perspective of observation, and the state you present during communication.

Here is a series of suggested steps to help you sort out your thoughts:

Step 1: Stay Calm and View Objectively

First, understand that a psychiatric diagnosis is a "tool" and "roadmap" to help doctors understand your distress and formulate a treatment plan for you—not a "label" that defines you. Differences in diagnosis do not mean one doctor is necessarily "right" or "wrong"; they may simply reflect different focuses of observation.

Step 2: Carefully Review and Organize Information

Before taking further action, organize the information you have.

Collect all materials: Gather the diagnosis reports, medical records, and medication prescriptions from both (or multiple) doctors.

Recall the consultation process: What main symptoms did you describe to each doctor? What was your emotional state at the time? What key questions did each doctor ask you?

Create a "symptom diary": If you don’t already have one, start keeping track. Each day, briefly note your mood swings, sleep quality, appetite, energy levels, any unusual thoughts or behaviors, and any important life events you feel are relevant. This will become valuable information for communicating with doctors.

Step 3: Analyze the "Differences" in Diagnoses

Carefully examine the diagnosis documents and try to understand the core of the differences:

Are the diagnoses completely different? For example, one diagnoses "Bipolar Disorder" and the other "Major Depressive Disorder." These are two distinct conditions that require careful attention.

Are the primary and secondary diagnoses or comorbid diagnoses different? For example, one diagnoses "Depression with Anxiety" and the other "Anxiety Disorder with Depression." This is actually very common, indicating you are experiencing both emotional distress, but doctors have different judgments on their primary and secondary relationships. In such cases, treatment plans (especially medications) may be very similar.

Are the severity or subtypes different? For example, one diagnoses "Mild Depression" and the other "Moderate Depression." This difference may stem from fluctuations in your state during consultations at different times.

Step 4: Choose One Doctor for In-Depth Communication

After organizing the information, you can select a doctor you trust more and with whom you feel more comfortable communicating for a follow-up visit.

Frankly express your confusion: You can directly and politely tell the doctor: "Hello, Doctor. I previously consulted another expert who gave a diagnosis of XX. This has left me a bit confused. Could you help me understand why you made your current diagnosis, and what the differences and connections are between these two diagnoses?"

Present the information you organized: Provide the doctor with your "symptom diary" and the diagnosis from the other doctor. A responsible doctor will welcome this information as it helps them understand your condition more comprehensively.

Step 5: Seek a Third-Party Authoritative Opinion ("Consultation")

If you are still very confused after communicating with the doctor, or if the treatment plans for the two diagnoses are completely opposite (for example, there are key differences in medication for Bipolar Disorder and Depression), it is necessary to seek a third-party authoritative opinion.

Choose a higher-level expert: Consider visiting an expert clinic or VIP clinic at the most authoritative mental health center or Grade A tertiary hospital in your area.

Bring all materials: When seeing the third expert, be sure to bring all medical records and diagnosis reports from the previous two doctors. This is not "challenging" the doctors, but providing the new expert with the most comprehensive decision-making information to assist them in differential diagnosis.

Step 6: Focus More on Treatment Plans and Effects

Sometimes, rather than obsessing over a "perfect" diagnostic label, it is more important to see which treatment plan is effective for your current main distress.

Compare the commonalities of treatment plans: Different diagnoses may have overlapping medications or treatment recommendations (for example, many antidepressants are also used to treat anxiety disorders).

Start with a low dose: Under the doctor’s guidance, regardless of the final diagnosis, treatment usually starts with a safe low dose, and your response is closely observed.

Prioritize "symptom relief": Has your sleep improved? Is your mood stable? Has your energy recovered? These are more practical measures than diagnostic labels.

Important Reminders:

You are the expert on your own feelings: Your subjective feelings and detailed descriptions are the most important basis for doctors’ diagnoses.

Treatment is a dynamic process: Psychiatric diagnoses and treatment plans may need to be dynamically adjusted according to the development of the condition. The initial diagnosis may only be a preliminary judgment.

Establish a trusting doctor-patient relationship: Finding a doctor you are willing to trust and cooperate with long-term is crucial for recovery.

In short, when facing different diagnoses, first calm down and analyze, then actively communicate with the doctor with sufficient preparation and specific questions, and seek a more authoritative third-party opinion if necessary. It is hoped that you can quickly find the most suitable diagnosis and treatment path and recover soon.

**Q14 Will Visiting a Psychiatrist Be Considered as Having a Mental Illness?**

Of course not.

Visiting a psychiatrist does not mean you will be diagnosed with a "mental illness." This is a very common misunderstanding.

You can understand it this way:

A visit is for evaluation, not conviction: Going to see a psychiatrist is like going to a pulmonologist for a persistent cough. The doctor needs to conduct a professional consultation and evaluation to determine the cause of your "cough." It may be just a mild cold, allergies, or bronchitis, and only in rare cases will it be severe pneumonia. Similarly, a psychiatrist will assess the difficulties you are currently facing (such as anxiety, insomnia, low mood, stress, etc.), identify the cause, and provide professional help.

The scope of diagnosis and treatment is very broad: Psychiatrists handle far more than the severe "mental illnesses" (such as Schizophrenia) that people traditionally associate with the field. In fact, the vast majority of people seeking help do so because of common life difficulties, such as:

Anxiety Disorders: Excessive worry and restlessness about the future.

Depression: Persistent low mood and loss of interest.

Sleep Disorders: Difficulty falling asleep, early awakening, frequent dreams.

Obsessive-Compulsive Disorder: Uncontrollable repetitive thoughts and behaviors.

Emotional distress caused by interpersonal relationship issues, academic or work pressure, etc.

Diagnosis is a rigorous and scientific process: Doctors will only give a corresponding "diagnosis" when your symptoms meet internationally recognized diagnostic criteria (such as DSM-5 or ICD-11). This diagnosis is a scientific conclusion aimed at better and more targeted treatment, not to label you. In many cases, you may only need some psychological counseling or lifestyle advice, and may not require medication or meet any "diagnostic criteria."

In summary: Courageously walking into a psychiatrist’s office is a positive step towards facing problems and caring for your mental health. It means you have the courage to seek professional help to improve your quality of life, and it is just as normal as going to the hospital when you are physically unwell. It does not mean you are "crazy" or "abnormal."

**Q15 What Are the Main Methods for Treating Mental Illnesses?**

There are various methods for treating mental illnesses, and personalized treatment plans usually need to be formulated based on an individual’s specific situation, type of illness, and severity. The best treatment results often come from a combination of multiple methods. Here are some of the main current methods for treating mental illnesses:

1. Psychotherapy

Psychotherapy, often referred to as "talk therapy," is a core component in the treatment of many mental illnesses. It involves communicating with a mental health professional (such as a psychologist, therapist, or counselor) to understand and address your thought patterns, emotions, and behaviors.

Main types include:

Cognitive Behavioral Therapy (CBT): Helps patients identify and change negative or irrational thought patterns and behaviors. CBT is highly effective for various issues such as depression, anxiety disorders, and substance abuse.

Dialectical Behavior Therapy (DBT): A type of cognitive behavioral therapy that focuses on teaching coping skills to help people manage intense emotions, improve interpersonal relationships, and is particularly useful for Borderline Personality Disorder.

Psychodynamic Therapy: Focuses on exploring unconscious thoughts and emotions to understand how past experiences influence present behaviors.

Interpersonal Therapy (IPT): Focuses on resolving interpersonal relationship problems and improving communication skills, often used to treat depression.

Family Therapy and Couples Therapy: Helps family members or partners improve communication, resolve conflicts, and jointly support the member with a mental illness.

2. Medication

Psychiatric medications cannot cure mental illnesses, but they can significantly control symptoms, making other treatments (such as psychotherapy) more effective.

Main types include:

Antidepressants: Used to treat depression, anxiety disorders, panic disorders, Post-Traumatic Stress Disorder (PTSD), etc. The most common type is Selective Serotonin Reuptake Inhibitors (SSRIs).

Anti-Anxiety Medications: Such as benzodiazepines, usually used for short-term relief of severe anxiety and insomnia, but may be addictive.

Mood Stabilizers: Mainly used to treat Bipolar Disorder (Manic-Depressive Illness) to control severe mood swings.

Antipsychotics: Mainly used to treat psychotic disorders such as Schizophrenia, but can also be used to treat Bipolar Disorder or as an adjunctive treatment for Major Depression.

Stimulants: Mainly used to treat Attention Deficit/Hyperactivity Disorder (ADHD).

3. Brain Stimulation Therapies

These therapies may be considered when psychotherapy and medication are ineffective, or in severe cases where rapid results are needed.

Electroconvulsive Therapy (ECT): Under general anesthesia, a brief electric current is delivered to the brain to induce a short, generalized seizure. ECT is one of the fastest and most effective treatments for severe depression, Bipolar Disorder, and Catatonia.

Repetitive Transcranial Magnetic Stimulation (rTMS): Uses magnetic fields to stimulate specific areas of the brain related to mood regulation, and is a non-invasive treatment mainly used for depression that is resistant to medication.

Vagus Nerve Stimulation (VNS): A pacemaker-like device is surgically implanted to send electrical pulses to the vagus nerve in the neck, which in turn affects brain activity.

4. Hospitalization and Day Treatment Programs

Inpatient Treatment: When the condition is severe, the patient may be at risk to themselves or others, or unable to care for themselves, requiring 24-hour monitoring and intensive treatment in a hospital.

Day Treatment/Partial Hospitalization Programs: Patients receive intensive treatment at a treatment facility during the day and return home at night. This provides an option for patients who need high levels of support but do not require 24-hour monitoring.

5. Lifestyle Adjustments and Support

These methods are usually used as supplements to professional treatment and are crucial for the recovery process.

Establish a regular daily routine: Including adequate sleep, a healthy diet, and regular physical exercise.

Learn stress management and relaxation techniques: Such as mindfulness, meditation, yoga, and deep breathing exercises.

Avoid alcohol and substance abuse: These substances can exacerbate symptoms of mental illness and interfere with the effectiveness of medications.

Join Support Groups: Sharing experiences and feelings with people who have similar experiences can reduce loneliness and provide emotional support and practical advice.

Summary

The most important step is to seek professional help. If you or someone you know is experiencing mental health issues, consult a doctor or mental health professional. They can provide an accurate diagnosis and recommend the most appropriate treatment plan based on the specific situation. Mental illnesses are treatable, and with appropriate intervention, most people can significantly improve their symptoms and live fulfilling, meaningful lives.

**Q16 Which Is More Effective: Psychiatric Medication or Psychotherapy?**

This is a very important and common question, but the answer is not "black and white." Simply put, psychiatric medication and psychotherapy are not mutually exclusive, and in many cases, a combination of the two is the most effective treatment plan.

Think of them as addressing a computer problem:

Medication is like repairing the "hardware." It directly adjusts imbalanced neurotransmitters (such as serotonin and dopamine) in the brain, correcting biological abnormalities and allowing the "computer motherboard" to function normally again.

Psychotherapy is like optimizing the "software." Through conversation and training, it helps you identify and change thought patterns, emotional responses, and behavioral habits that cause problems, install "anti-virus software" and "firewalls," and improve system operating efficiency.

To clarify further, we can compare them in the following aspects:

Core Comparison

Aspect	Medication (Pharmacotherapy)	Psychotherapy

Mechanism of Action	Physiological level: Regulates brain neurotransmitters and improves brain biological function.	Psychological and behavioral level: Changes cognitive, emotional, and behavioral patterns and enhances coping skills.

Speed of Onset	Relatively fast: Significant improvement in symptoms can usually be seen within a few weeks (especially for severe symptoms).	Relatively slow: Time is needed to establish a therapeutic relationship, learn and practice skills, and results are gradual.

Main Advantages	1. Rapidly relieves severe symptoms (such as suicidal tendencies, hallucinations).

2. Essential for severe mental illnesses (such as Schizophrenia).

3. Relatively standardized treatment plans.	1. Addresses the root causes of problems to achieve "fundamental cure."

2. Teaches long-term useful coping skills to prevent recurrence.

3. No medication side effects.

4. Promotes personal growth and self-awareness.

Main Limitations	1. May have side effects (such as drowsiness, weight gain, nausea, etc.).

2. Symptoms may recur after stopping medication; "treats the symptoms but not the root cause."

3. May require long-term medication, with a risk of withdrawal reactions.	1. Slow onset of effect for acute or severe symptoms.

2. Requires high patient initiative and insight.

3. Effectiveness depends heavily on the therapist’s skill and compatibility.

4. May have higher time and financial costs.

Long-Term Effects	Continued medication can control symptoms persistently.	The skills learned are useful for a lifetime, and the recurrence rate may be lower in the long run.

"Which Is More Effective" Depends on the Specific Situation

Situations where medication is preferred:

Severe mental disorders: Such as Schizophrenia and Bipolar Disorder. These diseases have clear biological bases, and medication is the cornerstone of controlling the condition and preventing deterioration. Psychotherapy is used as an adjunct after the condition stabilizes to help patients adapt to society and manage emotions.

Severe depression/anxiety: When symptoms are so severe that normal life and work are impossible, or even suicidal thoughts occur, medication can provide rapid "support," stabilize mood, and create conditions for psychotherapy.

Obvious physical symptoms: When psychological problems cause severe physical symptoms such as insomnia, loss of appetite, palpitations, and chest tightness, medication can relieve these discomforts more quickly.

Situations where psychotherapy is preferred:

Mild to moderate depression and anxiety: When symptoms are not yet severe, the effect of psychotherapy (especially Cognitive Behavioral Therapy, CBT) is comparable to that of medication, and it is more effective in preventing recurrence in the long run.

Stress-related disorders: Such as psychological distress caused by specific events such as a breakup, job loss, or the death of a loved one.

Personal growth, interpersonal relationship issues, perfectionism, etc.: These issues are rooted in thought and behavioral patterns, which are the core areas of psychotherapy.

Patients who are unwilling or unsuitable for medication: Such as pregnant women or those who are particularly sensitive to medication side effects.

"Strong Combination": Combination of Medication and Psychotherapy

This is currently the internationally recognized "gold standard" for treating moderate to severe mental disorders (such as depression, anxiety disorders, obsessive-compulsive disorder, etc.).

A classic metaphor: Medication is like a "crutch." When your leg (mental state) is fractured, it supports you to stand up and restore basic walking ability; psychotherapy is like "rehabilitation training," teaching you how to exercise muscles correctly, adjust your posture, and ultimately allow you to throw away the crutch and walk independently and healthily.

Medication rapidly stabilizes symptoms, allowing you to have the energy and ability to "think" and "change."

Based on this, psychotherapy addresses underlying issues, learns new skills, and fundamentally reduces the risk of future recurrence.

Conclusion and Recommendations

Do not view medication and psychotherapy as opposing "either/or" options, but rather as tools with different functions in a toolbox.

The most important first step is to seek a professional evaluation. You should:

Consult a Psychiatrist: They have prescribing authority, can accurately diagnose your condition, and determine if medication is needed and which type.

Consult a Clinical Psychologist or Psychotherapist: They specialize in assessing your mental state and providing appropriate psychotherapy plans.

Professional doctors will tailor an individualized treatment plan for you, including medication, psychotherapy, or a combination of both, based on your type of illness, severity, personal preferences, and living conditions.

**Q17 Is Exercise Effective for Mental Illness?**

Yes, numerous studies have shown that regular physical exercise is highly effective in improving and managing mental illnesses. It is often recommended as an effective adjunct to professional treatments such as psychotherapy and medication.

Exercise is effective for multiple reasons, covering both physiological and psychological levels:

Physiological Mechanisms:

Release of endorphins: Exercise stimulates the brain to release endorphins, natural "feel-good" chemicals that can relieve pain and improve mood.

Regulation of neurotransmitters: Regular exercise can increase the levels of serotonin, dopamine, and norepinephrine in the brain. Imbalances in these neurotransmitters are closely linked to various mental illnesses such as depression and anxiety disorders.

Reduction of stress hormones: Exercise helps lower the levels of stress hormones such as cortisol in the body.

Improvement of sleep: Mental illnesses are often accompanied by sleep problems. Regular exercise helps regulate the sleep cycle and improve sleep quality.

Promotion of neuroplasticity: Exercise can stimulate the growth of neurotrophic factors in the brain, promote the generation and connection of new neurons, and help the brain function more healthily and adaptively.

Psychological Mechanisms:

Enhancement of self-confidence: Achieving exercise goals can bring a sense of accomplishment, and seeing improvements in physical fitness can significantly boost self-esteem and confidence.

Provision of positive "distraction": Exercise can temporarily shift your attention away from negative thought cycles and focus on physical sensations.

Increase in social opportunities: Participating in group exercises or going to the gym can reduce loneliness and social isolation, which are factors that cause or exacerbate mental illnesses.

Improvement of coping skills: Exercise is a healthy way to manage stress and negative emotions, and can replace some unhealthy behaviors (such as overeating, excessive drinking, etc.).

For Which Mental Illnesses Is It Effective?

Depression: For mild to moderate depression, the effectiveness of exercise can even be comparable to that of antidepressants and psychotherapy.

Anxiety disorders and panic disorders: Exercise is one of the most effective ways to reduce anxiety symptoms.

Stress-related disorders: Exercise can effectively help manage and release daily stress.

Attention Deficit/Hyperactivity Disorder (ADHD): Exercise can improve attention and executive function.

Exercise Recommendations:

Types: Both aerobic exercise (such as brisk walking, running, swimming, cycling) and strength training (such as weightlifting) have been proven effective. The most important thing is to choose an exercise you enjoy and can stick to long-term.

Intensity and frequency: The World Health Organization recommends that adults engage in at least 150 minutes of moderate-intensity aerobic exercise or 75 minutes of vigorous-intensity aerobic exercise per week. Even 15-20 minutes of brisk walking every day can bring significant changes.

Consistency is key: The effects of exercise are not immediate; long-term adherence is required to see sustained improvements in mental health.

Important Reminder: Although exercise is highly beneficial, it cannot fully replace professional medical diagnosis and treatment. If you are experiencing severe mental distress, be sure to seek help from a doctor or mental health professional. Before starting a new exercise program, especially if you have underlying health issues, it is also advisable to consult a doctor.

**Q18 What Types of Medications Are Commonly Used to Treat Mental Illnesses?**

Hello, there are many types of medications used to treat mental illnesses, which are usually classified based on their main therapeutic goals and mechanisms of action. Here are the most commonly used classes of psychiatric medications in clinical practice:

1. Antidepressants

This is the most widely used class of psychiatric medications, mainly used to treat depression, but also commonly used for anxiety disorders, obsessive-compulsive disorder, Post-Traumatic Stress Disorder (PTSD), etc.

Mechanism of action: Mainly improves mood by regulating the levels of neurotransmitters (such as serotonin, norepinephrine, and dopamine) in the brain.

Common types:

Selective Serotonin Reuptake Inhibitors (SSRIs): Currently the first-line medications with relatively few side effects. Examples include Fluoxetine, Sertraline, Escitalopram.

Serotonin-Norepinephrine Reuptake Inhibitors (SNRIs): Effective for both depressive and anxiety symptoms. Examples include Venlafaxine, Duloxetine.

Tricyclic Antidepressants (TCAs): Older medications that are no longer first-line choices due to more side effects. Examples include Amitriptyline.

2. Antipsychotics

Mainly used to treat Schizophrenia, manic episodes of Bipolar Disorder, and other psychotic symptoms (such as hallucinations, delusions).

Mechanism of action: Mainly works by blocking dopamine D2 receptors in the brain.

Common types:

First-generation (typical) antipsychotics: Mainly target positive symptoms (such as hallucinations) but are prone to causing extrapyramidal side effects (such as muscle stiffness, tremors). Examples include Haloperidol, Chlorpromazine.

Second-generation (atypical) antipsychotics: More commonly used in clinical practice now, effective for both positive and negative symptoms, with fewer extrapyramidal side effects, but may cause metabolic issues (such as weight gain, elevated blood sugar). Examples include Risperidone, Olanzapine, Quetiapine, Aripiprazole.

3. Mood Stabilizers

Mainly used to treat and prevent manic and depressive episodes of Bipolar Disorder (Manic-Depressive Illness).

Mechanism of action: The mechanism is complex, and they are believed to stabilize mood swings by stabilizing nerve cell membranes and regulating multiple neurotransmitter systems.

Common medications:

Lithium: A classic medication for treating Bipolar Disorder.

Anticonvulsants: Many medications used to treat epilepsy have also been found to have mood-stabilizing effects. Examples include Sodium Valproate, Lamotrigine, Carbamazepine.

4. Anti-Anxiety Medications

Used to quickly relieve anxiety, tension, and insomnia.

Mechanism of action: Most work by enhancing the effects of gamma-aminobutyric acid (GABA), an inhibitory neurotransmitter, to produce sedative and anxiolytic effects.

Common types:

Benzodiazepines: Fast-acting and highly effective, but addictive and usually recommended for short-term use. Examples include Diazepam (Valium), Lorazepam, Alprazolam.

Others: Such as Buspirone, a non-benzodiazepine anti-anxiety medication with no addiction potential but a slower onset of action.

5. Medications for Attention Deficit/Hyperactivity Disorder (ADHD)

These medications are mainly used to improve attention and control impulsive behaviors.

Common types:

Central nervous system stimulants: Examples include Methylphenidate (Ritalin).

Non-central nervous system stimulants: Examples include Atomoxetine.

Very Important Reminders:

Follow medical advice: All psychiatric medications must be used under the professional guidance of a psychiatrist. Doctors will select the most appropriate medication and dosage based on your specific diagnosis, symptoms, physical condition, and individual differences.

Do not self-medicate or discontinue medication: Adjusting the dosage or suddenly stopping medication on your own may lead to recurrence of the condition or severe withdrawal reactions.

Combination of medication and psychotherapy: For most mental illnesses, a combination of medication and psychotherapy (such as Cognitive Behavioral Therapy, CBT) usually achieves the best treatment effect.

If you or someone around you is experiencing psychological distress, be sure to seek professional medical help.

**Q19 How Long Does It Usually Take for Psychiatric Medications to Take Effect?**

This is a very important question, but the answer is complex as it varies from person to person, medication to medication, and diagnosis to diagnosis. The most crucial point is that the following information is for reference only, and you should absolutely follow the guidance of your doctor or pharmacist.

Simply put, the onset time of psychiatric medications can range from a few minutes to several months.

Here is the approximate onset time by medication type:

1. Antidepressants

Representative medications: Selective Serotonin Reuptake Inhibitors (SSRIs) such as Fluoxetine (Prozac), Sertraline (Zoloft), Escitalopram (Lexapro), etc.

Onset time: These are among the medications that require the most patience.

1-2 weeks: Some patients may feel initial improvements, such as better sleep or appetite. However, emotional improvements may not be obvious, and some side effects (such as nausea, headaches) may even occur, which are usually temporary.

4-8 weeks: Most patients will experience significant therapeutic effects during this period. Doctors usually recommend taking the medication for at least this long to assess whether a medication is effective.

More than 8 weeks: If the effect is still not ideal, the doctor may consider adjusting the dosage or switching to another medication.

2. Anti-Anxiety Medications

The onset time varies greatly among medications in this category.

Benzodiazepines:

Representative medications: Alprazolam (Xanax), Lorazepam (Ativan), etc.

Onset time: Very fast, usually relieving acute anxiety or panic attacks within 15-60 minutes.

Note: These medications are prone to addiction and are usually used for short-term treatment or as needed, not for long-term regular use.

Other anti-anxiety medications:

Such as Buspirone, whose onset is more like that of antidepressants, taking 2-4 weeks to show effects.

3. Antipsychotics

Representative medications: Olanzapine, Risperidone, Aripiprazole, etc.

Onset time:

Hours to days: Sedative effects can be seen quickly for certain symptoms, such as agitation, restlessness, or insomnia.

Weeks to months: Core psychotic symptoms such as hallucinations and delusions take longer, usually 4-6 weeks or more to achieve optimal therapeutic effects.

4. Mood Stabilizers

Representative medications: Lithium, Sodium Valproate, Lamotrigine, etc., mainly used to treat Bipolar Disorder.

Onset time:

It usually takes 1-3 weeks or longer to reach a stable therapeutic concentration in the blood and start controlling mood swings.

Note: Medications like lithium require regular monitoring of blood levels to ensure they are within the effective and safe range.

Key Factors Affecting Onset Time

Individual differences: Metabolism, age, weight, and genetic factors all affect the absorption and response to medications.

Diagnosis and severity of symptoms: Different diseases and symptoms respond to medications at different rates.

Dosage: Doctors usually start with a low dose and gradually increase it based on your response and tolerance, which in itself takes time.

Adherence: Taking the medication on time and in the correct dosage every day is crucial. Irregular medication intake will seriously affect the therapeutic effect.

Placebo effect: Sometimes, the act of starting treatment itself can bring about some psychological improvements.

The Most Important Point

Please be patient and maintain close communication with your doctor.

Do not discontinue medication on your own: Even if you feel better or think the medication is not working, never stop taking it or change the dosage on your own. Suddenly stopping medication may lead to withdrawal reactions or recurrence of the condition.

Record your feelings and side effects: Tell your doctor how you feel, both good and bad, as this can help the doctor adjust the most suitable treatment plan for you.

Treatment is a process: Finding the right medication and dosage may require some time and trial. This is normal.

In summary, psychiatric medication treatment is a scientific and personalized process that requires a good relationship of trust and communication between doctors and patients.

**Q20 Will Taking Psychiatric Medications Damage Brain Function?**

This is a very important and common concern. The simple answer is:

When used rationally under the guidance of a professional doctor, psychiatric medications are intended to "repair" or "regulate" brain function, not "damage" it.

However, the answer to this question is complex and needs to be understood from several levels:

1. The Impact of Mental Illness Itself on the Brain

First, it is important to understand that untreated mental illnesses (such as severe depression, schizophrenia, bipolar disorder, etc.) themselves can have negative effects on brain function and structure. For example:

Chronic stress: Long-term, severe depression and anxiety can lead to persistently elevated levels of stress hormones (such as cortisol), which may damage the hippocampus (a key brain region responsible for learning and memory), leading to a reduction in its volume.

Neuroinflammation: A growing body of evidence suggests that many mental illnesses are associated with chronic inflammatory responses in the brain, which can impair neuronal function.

Dysfunction: In the diseased state, the brain’s neurotransmitter systems (such as serotonin, dopamine, norepinephrine) are inherently imbalanced or dysfunctional, leading to abnormalities in mood, thinking, and behavior.

From this perspective, the goal of medication treatment is precisely to reverse or alleviate these functional impairments caused by the illness itself.

2. How Medications "Repair" Brain Function

Psychiatric medications work by regulating neurotransmitters in the brain, with the aim of:

Restoring chemical balance: For example, the most common antidepressants, SSRIs (Selective Serotonin Reuptake Inhibitors), help improve mood and reduce anxiety by increasing the concentration of serotonin in the synaptic cleft.

Promoting neuroplasticity: Long-term use of certain medications can promote neurogenesis and the reconstruction of synaptic connections, which is believed to be a key mechanism for the long-term therapeutic effects of medications. They help the brain "rewire" to form healthier emotional and thinking patterns.

Think of psychiatric medications like insulin for diabetics: Diabetes is caused by impaired pancreatic function, and insulin is used to correct this dysfunction. Similarly, psychiatric medications are tools used to correct chemical imbalances in the brain.

3. Where Does the Fear of "Damaging the Brain" Come From? — Side Effects and Risks

The public’s fear of "damaging the brain" mainly stems from the side effects and potential risks of medications. These are indeed real and must be addressed:

Short-term side effects: In the early stages of taking medication, many people experience side effects that affect brain function, such as:

Drowsiness, fatigue, difficulty concentrating ("brain fog")

Dizziness, temporary memory loss

Emotional blunting (feeling "less emotional")

These side effects are usually temporary and will gradually diminish or disappear as the body adapts to the medication. Doctors can also address these issues by adjusting the dosage or switching medications.

Long-term risks: Certain medications do have some risks that need to be vigilant about with long-term use:

Tardive Dyskinesia (TD): This is a risk associated with long-term use of (especially first-generation) antipsychotic medications, characterized by involuntary, repetitive body movements. Modern new-generation medications have significantly reduced this risk.

Dependence and withdrawal reactions: Certain medications, especially benzodiazepines (commonly used for anxiety and sleep aid), may cause physical dependence with long-term use. Suddenly stopping the medication can lead to withdrawal reactions such as anxiety, insomnia, and palpitations. Therefore, any reduction or discontinuation of medication must be done slowly under the guidance of a doctor.

Metabolic effects: Some antipsychotic medications and mood stabilizers may affect metabolism, leading to weight gain, elevated blood sugar, and blood lipids, which can indirectly affect overall health.

Conclusion and Recommendations

Overall, we can draw the following conclusions:

Weighing the pros and cons is key: For moderate to severe mental illnesses, the damage to the brain and life caused by not treating them is usually far greater than the potential risks of rational medication use.

Medications are tools, not magic: Medications are powerful tools to help restore normal brain function, but they are not a panacea. The best treatment plan usually combines medication with psychotherapy (such as CBT), supplemented by a healthy lifestyle (exercise, diet, sleep).

Professional guidance is crucial:

Do not self-medicate or discontinue medication. Psychiatric medications are prescription drugs and must be prescribed by a professional doctor based on your specific situation.

Maintain communication with your doctor. If you are concerned about side effects, be sure to tell your doctor. Doctors have various strategies to address them, including adjusting the dosage, switching medications, or adding other medications to counteract side effects.

Be patient. Most psychiatric medications take weeks or even longer to fully take effect, and the body also needs time to adapt.

In summary, psychiatric medications are in a "race" against the damage caused by illness to the brain. Under the guidance of a professional doctor, they are not "poisons," but "allies" that help you regain control of your brain function and return to a normal life.

**Q21 How to Cope with Side Effects of Psychiatric Medications, Such as Weight Gain and Drowsiness?**

Hello, this is a very important and common question. Coping with side effects of psychiatric medications, especially weight gain and drowsiness, requires a comprehensive strategy, and the core principle is: Do not discontinue medication or adjust the dosage on your own—be sure to communicate with your attending doctor.

Here are some detailed coping strategies to help you:

I. The Most Important First Step: Communicate Frankly with Your Doctor

This is the cornerstone of all strategies. When you see your doctor, do not just say "I feel much better" or "It’s still the same"—specifically tell the doctor how the side effects are affecting your life.

Be specific in your description: "Since I started taking the medication, I have gained 5 kilograms and always feel hungry." "I need to sleep more than 10 hours a day, but I’m still groggy during the day and can’t concentrate at work."

Discuss solutions: After understanding your specific situation, the doctor may take the following measures:

Continue observation: Some side effects are more obvious in the early stages of taking the medication and may decrease as the body adapts.

Adjust the dosage: The doctor may try reducing the medication dosage while ensuring the therapeutic effect.

Adjust the time of administration: If drowsiness is an issue, the doctor may recommend taking the medication before bedtime at night.

Switch medications: If the side effects seriously affect your quality of life, the doctor may consider switching to another medication with a different side effect profile. For example, some medications are more likely to cause weight gain, while others have less impact.

Combine medications: In some cases, the doctor may prescribe another medication to counteract the side effects (such as a medication to control appetite or boost energy), but this requires careful evaluation.

II. Strategies for Coping with Weight Gain

Psychiatric medications usually cause weight gain for two reasons: increasing appetite and possibly affecting the body’s metabolism.

Adjust your diet (what to eat):

Increase protein and fiber: Protein (chicken breast, fish, soy products, eggs) and high-fiber foods (vegetables, fruits, whole grains) can provide a stronger sense of fullness and help you control total calorie intake.

Choose "low-energy-density" foods: For example, a large plate of vegetable salad has far fewer calories than a small piece of cake, but the former is more filling.

Prepare healthy snacks: Keep snacks like cucumber, cherry tomatoes, nuts (in moderation), and sugar-free yogurt on hand. When hunger strikes, avoid high-sugar and high-oil snacks.

Stay hydrated: Sometimes the body misinterprets "thirst" as "hunger." Drink plenty of water, or sugar-free tea and black coffee.

Change your eating habits (how to eat):

Eat regularly: Ensure three meals a day to avoid overeating at the next meal due to excessive hunger.

Mindful eating: Eat attentively, chew slowly, and savor the taste of food and the body’s satiety signals. This helps you stop in time when you feel "full."

Control portion sizes: Try using smaller tableware, or divide the food into individual portions when cooking.

Adhere to regular exercise:

Aerobic exercise: Brisk walking, jogging, swimming, cycling, etc., are effective ways to burn fat. Stick to it 3-5 times a week for more than 30 minutes each time.

Strength training: Weightlifting, push-ups, squats, etc., can increase muscle mass, and muscle is the main driver of calorie consumption, helping to improve the basal metabolic rate.

Find enjoyment: Choose an exercise method you like, whether it’s dancing, playing ball, or yoga—persistence is the most important thing. Start with low intensity and progress gradually.

III. Strategies for Coping with Drowsiness and Fatigue

Discuss adjusting the time of administration with your doctor: This is the most direct and effective method. If the medication causes daytime drowsiness, ask the doctor if all or most of the dosage can be taken before bedtime at night.

Optimize sleep hygiene:

Establish a regular schedule: Try to go to bed and wake up at the same time every day, even on weekends.

Create a good sleep environment: Keep the bedroom dark, quiet, and cool.

Relax before bed: Avoid using electronic devices such as mobile phones and computers before bed, as blue light can inhibit melatonin secretion. Try taking a warm bath, listening to soothing music, reading, or meditating.

Coping methods during the day:

Expose yourself to sunlight in the morning: Sunlight helps regulate the biological clock and lets the body know "it’s daytime now."

Consume caffeine in moderation: A cup of coffee or tea in the morning can help boost energy, but avoid consuming it in the afternoon or evening to prevent affecting nighttime sleep.

Engage in brief physical activity: When feeling drowsy, stand up and walk around, do some stretching exercises—this can quickly restore energy.

Planned naps: If you are very tired, you can take a 20-30 minute "power nap," but do not sleep for too long, as this may affect nighttime sleep.

Summary and Mindset

Acceptance and patience: Accept that side effects are part of the treatment process, and be patient—both the body’s adaptation and changes in lifestyle take time.

Record and feedback: Prepare a "health diary" to record your weight, sleep, mood, diet, exercise, and changes in side effects. This will be very helpful when communicating with your doctor.

Seek support: Do not bear it alone. Communicate your troubles with family, friends, or a psychotherapist—their understanding and support are important forces on the road to recovery.

You are not alone: Remember that these side effects are very common, and many people are actively looking for coping methods just like you. Your proactive behavior in seeking solutions is a very positive and powerful step.

The most important thing is to regard your doctor as a partner and jointly develop the most suitable treatment and recovery plan for you. Wish you soon find a balance and restore health and vitality!

**Q22 Can Taking Psychiatric Medications Lead to Addiction?**

This is a very common and important question. Simply put: The vast majority of psychiatric medications do not lead to "addiction" in the usual sense, but some medications do carry risks of "physical dependence" and "withdrawal reactions."

To understand this question clearly, we must distinguish between two key concepts: Addiction and Physical Dependence.

1. Key Concept Distinction

Addiction: This is a complex, chronic, relapsing brain disease. Its core characteristics are "loss of control" and "craving." Patients will compulsively seek and use a substance regardless of adverse consequences (such as impairing health, family relationships, work, etc.). This is usually related to the drug strongly activating the brain’s reward center (dopamine system), producing a sense of pleasure or "euphoria." For example, drugs such as heroin, cocaine, and methamphetamine are highly addictive.

Physical Dependence: This refers to the body adapting to the continuous presence of a drug. If the drug dosage is suddenly stopped or reduced, the body will experience a series of uncomfortable symptoms, known as "Withdrawal Symptoms." This dependence is physiological and not equivalent to addiction. Many common medications, such as certain blood pressure medications, and even the coffee we drink every day, can cause physical dependence and withdrawal reactions.

Core difference: The core of addiction is psychological compulsive craving and uncontrollable behavior, while the core of physical dependence is physical discomfort after stopping the drug. A person with physical dependence does not necessarily compulsively seek the drug.

2. Risk Analysis of Different Types of Psychiatric Medications

There are many types of psychiatric medications, and their risks of addiction and dependence vary.

A. Medications with Low Risk / No Addiction Risk

These medications are the mainstay of psychiatric treatment. They do not act on the brain’s "reward center," do not produce euphoria, and therefore have no addictive potential. However, some medications have physical dependence, and stopping them requires caution.

Antidepressants

Representative medications: Sertraline, Fluoxetine, Paroxetine, Escitalopram (SSRI class); Venlafaxine, Duloxetine (SNRI class).

Risk: No addiction, but significant physical dependence. If stopped suddenly, withdrawal reactions are likely to occur, including dizziness, nausea, anxiety, insomnia, fatigue, paresthesias (such as "brain tremors/electric shocks"), etc.

Correct approach: Must gradually reduce the dosage under the doctor’s guidance over several weeks or months until stopping completely.

Antipsychotics

Representative medications: Olanzapine, Risperidone, Quetiapine, Aripiprazole, etc.

Risk: No addiction. Suddenly stopping the medication may lead to recurrence or rebound of original psychotic symptoms (such as hallucinations, delusions), as well as some withdrawal discomfort, but this is not addiction.

Mood Stabilizers

Representative medications: Lithium Carbonate, Sodium Valproate, Lamotrigine, etc.

Risk: No addiction. The core risk of stopping the medication without authorization is recurrence of the disease.

B. Medications with Addiction Risk

These medications need to be used strictly under the doctor’s guidance, either short-term or as needed.

Benzodiazepines

Representative medications: Alprazolam (Xanax), Lorazepam (Ativan), Diazepam (Valium), Clonazepam, etc. Commonly used for anxiety and sleep aid.

Risk: Clear addiction and physical dependence. They can quickly relieve anxiety, sedate, and aid sleep with obvious effects, making it easy to develop psychological dependence. After long-term use, the body will develop tolerance (needing a larger dosage to achieve the original effect), and stopping the medication can trigger severe withdrawal reactions (severe anxiety, insomnia, palpitations, even seizures).

Correct approach: Strictly follow medical advice. Usually used for acute phase, short-term use (within 2-4 weeks). If long-term use is needed, the doctor will use other safer medications as substitutes or develop an extremely cautious medication plan. Never increase the dosage or stop the medication suddenly on your own.

Central Nervous System Stimulants

Representative medications: Methylphenidate (Ritalin), Concerta, etc. Mainly used to treat Attention Deficit/Hyperactivity Disorder (ADHD).

Risk: Potential risk of abuse and addiction. Because they can improve attention and energy, if abused (such as using more than the prescribed dosage), they may lead to addiction.

Correct approach: Safe and effective when used at the prescribed dosage under strict doctor control.

Conclusion and Recommendations

Following medical advice is key: Your doctor best understands your condition and the risks of the medication. Please take the medication strictly as directed by your doctor, and do not adjust the dosage or stop the medication on your own.

Do not "fear medication": For the vast majority of patients, antidepressants, antipsychotics, etc., prescribed by doctors are safe and non-addictive. They are effective tools for treating diseases, and their therapeutic benefits far outweigh potential risks.

Be vigilant of high-risk medications: Especially benzodiazepines (various "Valium"), it is important to have a clear understanding of their risks and only use them short-term when necessary.

Gradually reduce medication, never stop suddenly: Regardless of the type of psychiatric medication, if you need to stop taking it, you should communicate with your doctor and develop a slow, gradual dosage reduction plan to minimize discomfort from withdrawal reactions.

In summary, psychiatric medications are an important part of modern medicine. Under the guidance of professionals, they can be used safely and effectively to help countless people recover their health. Fear and misunderstanding of medications often cause greater harm than the medications themselves.

**Q23 What Is Modified Electroconvulsive Therapy (MECT)?**

Let’s explain in detail what Modified Electroconvulsive Therapy (MECT) is.

Modified Electroconvulsive Therapy (MECT), often simply referred to as Electroconvulsive Therapy (ECT), is a safe and highly effective physical treatment method in modern psychiatry.

The key lies in the phrase "modified," which specifically refers to the improved, modern version of electroconvulsive therapy—completely different from the terrifying scenarios depicted in early movies.

Core Definition

Simply put, MECT is a method of treating mental illnesses by delivering brief, appropriate electrical stimulation to the brain under general anesthesia and complete muscle relaxation, artificially inducing a "seizure-like discharge" (i.e., "synchronous discharge of cells" in the brain).

The key points are:

The "seizure" only occurs inside the brain and can be monitored by an electroencephalogram (EEG).

Due to the use of muscle relaxants, the patient’s body will not experience generalized severe convulsions—at most, slight, barely noticeable twitches may be observed in the extremities. This is the meaning of "modified" (without convulsions).

How Is It Different from "Old-Fashioned" Electroconvulsive Therapy?

Early (1930s-1950s) electroconvulsive therapy was performed on awake patients, causing severe generalized convulsions that could lead to fractures, tooth damage, or intense fear in patients. This is why it was portrayed as a punitive and cruel method in movies like "One Flew Over the Cuckoo’s Nest."

In contrast, modern Modified Electroconvulsive Therapy (MECT) has completely transformed this situation through the introduction of the following key technologies:

General anesthesia: Short-acting intravenous anesthetics are used, and the patient is in a sleep state throughout the treatment, without any pain or discomfort.

Muscle relaxation: Muscle relaxants are used to block the conduction of electrical stimulation from the brain to the body’s muscles, thereby avoiding physical convulsions and related complications.

Continuous monitoring: The treatment is performed under close monitoring of vital signs (such as electrocardiogram, EEG, blood oxygen saturation), ensuring the safety of the entire process.

Therefore, modern ECT is a humanized, risk-controllable medical method.

Why Is MECT Performed? What Is Its Mechanism?

Although the exact mechanism of ECT has not been 100% clarified, the mainstream theory is that it is like "restarting" a computer that is running chaotically.

"Rebalancing" of neurotransmitters: The treatment can rapidly and significantly change the levels of various neurotransmitters in the brain, such as serotonin, dopamine, and norepinephrine, which are closely related to mood regulation. Its effect is faster and stronger than that of oral antidepressants.

Improvement of neural circuit function: It can regulate and "remodel" dysfunctional neural networks in the brain, especially brain regions related to emotion and cognition.

Promotion of neuroplasticity: Studies have found that ECT can promote the growth of new neuronal connections, helping the brain restore healthy function.

Who Is Suitable for This Treatment?

ECT is usually not the first-line treatment option, but it is an extremely important and potentially life-saving treatment choice for the following situations:

Severe depression: Especially patients with acute symptoms such as severe suicidal tendencies, refusal to eat, or catatonia (not speaking, not moving). The onset of ECT is much faster than that of medication, which can save lives quickly.

Treatment-resistant depression: Patients who do not respond well or have no response to multiple medications.

Manic episodes: Especially manic patients who are extremely excited, impulsive, and aggressive—ECT can quickly control symptoms.

Schizophrenia: Particularly effective for patients with catatonia (catatonic stupor or excitement) or severe aggressive behavior.

Certain special situations: Such as severe mental disorders during pregnancy and childbirth (where medication use is limited), elderly patients who cannot tolerate medication side effects, etc.

What Are the Risks and Side Effects?

Although modern ECT is very safe, there are still some potential risks and side effects:

Most common side effects:

Short-term memory impairment: This is the most concerned side effect. Patients may forget things that happened before and after the treatment (retrograde amnesia) or have difficulty remembering new information (anterograde amnesia). For most people, these memory problems gradually recover within weeks to months after the end of treatment, but a small number of patients report long-term effects.

Transient confusion after treatment: After waking up, patients may feel confused and disoriented, which usually resolves within half an hour to an hour.

Headache, nausea, muscle soreness: These are relatively common temporary discomforts that can be treated symptomatically.

Anesthesia-related risks: Like any minor surgery requiring general anesthesia, there are anesthesia-related risks, but for patients with normal cardiopulmonary function, the risk is extremely low.

Summary

Modified Electroconvulsive Therapy (MECT) is a modern, safe, and efficient physical therapy in psychiatry. By delivering brief electrical stimulation to the brain under general anesthesia, it rapidly improves severe mental symptoms, and has an irreplaceable value especially in dealing with acute, critical, or treatment-resistant mental disorders. It is no longer the terrifying "electric shock" of the past, but a humanized treatment method performed under close monitoring.

If you or someone around you is considering this treatment, be sure to consult a professional psychiatrist for a comprehensive evaluation and communication to make the most suitable decision.

**Q24 For Which Diseases Is Transcranial Magnetic Stimulation (TMS) Therapy Mainly Indicated?**

Hello, Transcranial Magnetic Stimulation (TMS) therapy is a non-invasive neuromodulation technology that uses magnetic pulses to regulate neural activity in specific areas of the brain. Its scope of application is constantly expanding, and it can be mainly divided into two categories: officially approved indications and potential applications still under research.

I. Main Officially Approved Indications

These are treatment areas that have been proven effective and safe through numerous clinical trials and approved by authoritative institutions such as the US FDA, European CE, or China NMPA:

Depression: This is the most classic and widely used application of TMS. It is mainly used to treat adult patients with moderate to severe depression who have not responded well to medication or cannot tolerate medication side effects, i.e., "Treatment-Resistant Depression (TRD)."

Obsessive-Compulsive Disorder (OCD): For adult patients with moderate to severe OCD, especially those who have not responded well to traditional medication and psychotherapy, TMS can be used as an effective adjunctive or alternative treatment.

Migraine: Specific types of TMS devices (single-pulse TMS) have been approved for the prevention and acute treatment of migraine with aura.

Smoking Cessation: By stimulating brain networks related to addiction, TMS has been proven to help adults reduce smoking cravings and improve smoking cessation success rates.

Presurgical Brain Function Mapping: Before neurosurgical operations, TMS can be used to non-invasively locate key functional areas of the brain (such as language areas, motor areas), helping doctors develop more precise surgical plans to maximize the preservation of patient function.

II. Promising Research and Potential Application Areas

In addition to the above-approved indications, TMS has also shown great potential in the treatment research of many other neurological and mental diseases, but these are usually regarded as "off-label uses," and their effectiveness is still being further verified:

Anxiety Disorders: Including Generalized Anxiety Disorder (GAD), Post-Traumatic Stress Disorder (PTSD), etc.

Chronic Pain: Such as neuropathic pain, fibromyalgia, etc., by regulating pain-perceiving pathways in the brain to relieve symptoms.

Post-Stroke Rehabilitation: Used to improve motor dysfunction (such as limb hemiplegia) and aphasia after stroke.

Parkinson’s Disease: Mainly used to improve motor symptoms (such as bradykinesia) and depressive mood associated with the disease.

Tinnitus: Attempting to reduce tinnitus perception by inhibiting excessive activity in the auditory cortex.

Autism Spectrum Disorder: Research explores its potential to improve social function and reduce repetitive stereotyped behaviors.

Cognitive Impairment: Including mild cognitive impairment and Alzheimer’s disease, aiming to slow down cognitive decline.

Other Addictive Diseases: Such as alcohol dependence, cocaine addiction, etc.

Important Reminders

Not a first-line treatment: TMS is usually not the first-line treatment option and is generally considered when traditional treatments (such as medication, psychotherapy) are ineffective.

Individualized evaluation: Whether TMS therapy is suitable needs to be comprehensively evaluated by an experienced doctor (usually a psychiatrist or neurologist).

Contraindications: People with metal implants in the body (especially in the head, such as aneurysm clips, cochlear implants, metal stents, etc.) or cardiac pacemakers are usually contraindicated. Patients with a history of epilepsy also need to be carefully evaluated.

In summary, the core applications of TMS are the treatment of treatment-resistant depression and OCD, while showing broad application prospects in many other neuropsychiatric fields. If you or someone around you has related distress, it is recommended to consult a professional medical institution to find out if this technology is a suitable treatment option.

**Q25 Can I Stop Taking Psychiatric Medications on My Own After Symptoms Improve?**

This is a very important question, and the answer is: It is strongly recommended that you do not stop taking psychiatric medications on your own, even if you feel your symptoms have improved.

This is a crucial decision related to your health and safety, and it must be made under the professional guidance of your attending doctor (psychiatrist).

Here are the main reasons why you cannot stop taking the medication on your own:

Risk of Relapse

Symptom improvement is often a sign that the medication is working continuously to help restore the balance of neurotransmitters in your brain. Stopping the medication without authorization is equivalent to removing the support when "the condition is not yet fully cured," which is likely to cause the previous symptoms (such as depression, anxiety, hallucinations, emotional instability, etc.) to return.

The condition after relapse may be more severe than before, and treatment may be more difficult.

Withdrawal Reactions

Your body and brain have adapted to the presence of the medication. Suddenly stopping the medication will disrupt this balance, causing a series of physical and psychological discomforts known as "Withdrawal Reactions."

Common withdrawal reactions include:

Physically: Dizziness, headache, nausea, vomiting, diarrhea, cold-like symptoms, muscle soreness, fatigue, insomnia, vivid dreams, and some strange sensations such as "brain ringing" or "brain zaps."

Psychologically: Increased anxiety, irritability, severe mood swings, panic attacks, recurrence of depressive mood.

These withdrawal symptoms are sometimes mistaken for a "relapse," causing unnecessary pain and confusion.

Rebound Effect

In some cases, suddenly stopping the medication may not only cause the original symptoms to return but also "rebound" with greater intensity than before treatment, making the situation worse.

So, What Is the Correct Way to Stop Taking the Medication If You Want To?

Communicate Fully with Your Doctor

When you feel your condition is stable and have the idea of reducing or stopping the medication, the first and most crucial step is to make an appointment and have a detailed communication with your attending doctor.

Tell the doctor how you feel, what you think, and why you want to stop taking the medication.

Conduct a Professional Evaluation

The doctor will conduct a comprehensive evaluation based on your specific situation, including:

How long you have maintained stable symptoms (usually a long stable period is required).

The severity and course of your initial condition.

Your current life stress, social support system, etc.

Whether you have had a history of relapse after stopping the medication in the past.

Follow a Gradual Dose Reduction Plan

If the doctor evaluates that you can try to reduce the dose, he/she will formulate a personalized, gradual dose reduction plan for you.

This process is by no means achieved overnight. It usually means reducing the medication dose very slowly over weeks, months, or even longer, allowing your brain sufficient time to adapt and readjust.

The speed and method of dose reduction vary from person to person and medication to medication, and the doctor will give the most suitable plan for you.

Close Monitoring and Follow-Up

During the dose reduction period and the observation period after stopping the medication, you need to maintain close contact with the doctor and have regular follow-up visits.

Once any discomfort or signs of symptom fluctuation appear, you should immediately inform the doctor to adjust the plan in a timely manner.

Summary:

You can think of psychiatric medications like a cast for a broken bone. Even if you feel the bone no longer hurts and you can walk, you must have the doctor determine if the bone has truly healed and guide you on how to safely remove the cast and perform subsequent rehabilitation exercises.

Psychiatric medications provide the necessary support and stability for your "brain." When and how to safely "remove the support" must be decided by the professional who best understands your condition—your doctor.

**Q26 How Long Do I Need to Continue Taking Psychiatric Medications After the Condition Stabilizes?**

This is a very important question, but there is no simple, one-size-fits-all answer. How long you need to continue taking psychiatric medications after the condition stabilizes is a highly individualized decision that must be discussed jointly by you and your attending doctor.

Do not reduce or stop the medication on your own. This is the most crucial point. Suddenly stopping the medication may lead to severe withdrawal reactions (discontinuation symptoms) or rapid recurrence of the disease.

Here are the key factors that influence the doctor’s decision on the duration of medication use. Understanding these can help you communicate better with your doctor:

1. Type and Characteristics of the Disease:

First-episode depression/anxiety: If it is the first episode and symptoms have been completely relieved, doctors usually recommend continuing the medication for 6 months to 1 year after the condition stabilizes. This is called "consolidation phase treatment" and "maintenance phase treatment," aiming to prevent short-term recurrence.

Recurrent depression/anxiety: If you have a history of multiple relapses (e.g., 2 or more), the doctor may recommend longer maintenance treatment, which may be 2-3 years or even longer. For patients with frequent relapses or severe conditions, long-term or even lifelong medication may be required—treating mental illness as a chronic disease that needs long-term management, similar to hypertension or diabetes.

Bipolar Disorder: Long-term medication is usually required. Bipolar Disorder has a high recurrence rate, and mood stabilizers are the cornerstone of preventing recurrence and maintaining emotional stability. Most patients need lifelong medication.

Schizophrenia: Long-term maintenance treatment is usually required. Schizophrenia is a disease with a high recurrence rate, and the risk of recurrence is extremely high if the medication is stopped too early. The vast majority of patients need to take antipsychotic medications for a long time or even lifelong to prevent recurrence and maintain social function.

2. Severity of the Condition and Remission Status:

The more severe the condition, the slower the symptom remission, or if there are residual symptoms after stabilization, the longer the medication duration is usually required.

Ideal "condition stabilization" means not only the disappearance of core symptoms but also the recovery of your social function (such as work, study, and interpersonal communication abilities) to the level before the illness.

3. Patient’s Personal Situation:

Risk of recurrence: Having a family history, early age of onset, other mental or physical comorbidities, persistent stressors in life, etc., all increase the risk of recurrence, and the doctor may recommend a longer medication duration accordingly.

Response to medication and tolerance: If you respond well to the medication and have minimal or tolerable side effects, the feasibility of long-term use is higher.

Psychotherapy and social support: If you are also receiving effective psychotherapy (such as CBT) and have a good family and social support system, this helps reduce the risk of recurrence and may become a positive factor for adjusting the medication plan in the future.

Summary and Recommendations

Overall, the treatment with psychiatric medications is divided into several phases:

Acute phase treatment: Controlling and eliminating symptoms.

Consolidation phase treatment: Continuing medication after the condition stabilizes, usually for 4-9 months, to prevent short-term recurrence.

Maintenance phase treatment: For patients at high risk of recurrence, longer-term treatment to prevent long-term recurrence.

Please be sure to maintain regular follow-up visits with your psychiatrist, and frankly communicate your feelings, any side effects, and your concerns about the duration of medication use. The doctor will synthesize all information and work with you to formulate the most suitable treatment plan for your personal situation, with long-term health and quality of life as the ultimate goals. Stopping the medication must also be done by gradually reducing the dose over weeks or months under the doctor’s guidance.

**Q27 Can Mental Illnesses Be Completely Cured?**

This is a very important and complex question. Answering simply with "yes" or "no" may lead to misunderstandings. In the field of psychiatry, we more commonly use the concepts of "Recovery" or "Clinical Remission" rather than "Cure."

To better understand, you can compare it to some chronic physical diseases, such as hypertension or diabetes. These diseases cannot currently be "cured," but through scientific treatment and lifestyle management, patients can live completely normal lives, with symptoms well-controlled and quality of life not affected. Mental illnesses are similar in many ways.

Here is a more detailed explanation:

1. Different Definitions of "Cure"

For certain situations, "complete recovery" can be achieved: For example, a single episode of depression or adjustment disorder triggered by a specific life event (such as job loss or breakup). Through timely psychotherapy and/or short-term medication, many people can completely get out of the predicament, with symptoms disappearing and no recurrence for a long time or even lifelong. From this perspective, this can be considered "cured."

For more situations, the goal is "effective management" and "functional recovery": For many chronic or recurrent mental illnesses, such as schizophrenia, bipolar disorder, severe depression, and generalized anxiety disorder, their occurrence is related to multiple factors such as genetics, physiology, and social psychology—similar to hypertension and diabetes. In such cases, the goals of treatment are:

Control symptoms: Through medication and psychotherapy, minimize or even eliminate symptoms such as hallucinations, delusions, mood swings, and anxiety (achieve "clinical remission").

Restore social function: Be able to study, work, and establish and maintain interpersonal relationships normally.

Improve quality of life: Live a meaningful and satisfying life for oneself.

Prevent recurrence: Learn to recognize early signs of recurrence and know how to respond to prevent the disease from recurring severely.

2. What Is "Recovery"?

The modern psychiatric concept of "recovery" does not only refer to the disappearance of symptoms. It is a more comprehensive concept that emphasizes that even if certain vulnerabilities of the disease still exist, the individual can still:

Have hope and dreams.

Take control of their own life.

Participate actively in society.

Live a fulfilling and valuable life.

3. Key Factors Affecting Recovery

Whether a person can recover well from a mental illness is affected by many factors:

Early diagnosis and standardized treatment: Seeking professional help as early as possible leads to better outcomes. Adhering to the full-dose, full-course treatment recommended by the doctor is crucial.

Comprehensive treatment: A combination of medication, psychotherapy (such as CBT, DBT, etc.), and physical therapy usually yields the best results.

Social support system: The understanding and support of family, friends, and colleagues are valuable resources in the recovery process.

Personal subjective initiative: The patient’s willingness to learn about the disease, actively participate in treatment, and strive to adjust their lifestyle (ensure sleep, healthy diet, regular exercise, etc.) plays a decisive role in recovery.

Stigma: Overcoming the stigma of mental illness and facing it and dealing with it openly, like treating a cold or fever, is an important step towards recovery.

Summary

In short, although it may not be entirely accurate to describe many chronic mental illnesses as "cured" or "completely eradicated," this by no means implies that mental illnesses are "incurable."

Through scientific and systematic treatment, the vast majority of mental illnesses can be effectively controlled and managed. Patients can fully achieve "clinical remission" and "functional recovery," return to normal life, and even gain personal growth and a deeper life experience after facing the challenges of the disease.

The most important thing is not to lose hope. Actively seeking professional help is the first and most crucial step towards recovery.

**Q28 Will Mental Illnesses Recur After Recovery?**

This is a very important and common concern.

The simple answer is: Yes, mental illnesses can indeed recur after "recovery" or "clinical remission."

But this does not mean that "recovery" is ineffective or hopeless. The best way to understand this is to compare it to some chronic physical diseases, such as hypertension, diabetes, or asthma. Through treatment and lifestyle adjustments, patients can control symptoms well and live normal lives, but the underlying risk of the disease still exists. If treatment is interrupted or triggering factors are encountered, symptoms may reappear.

Recurrence of mental illness is similar. We can understand it in the following aspects:

1. Why Do Recurrences Happen?

Recurrence is usually not caused by a single factor, but by the combined effect of multiple factors:

Stopping or reducing medication on one’s own: This is the most common and main cause of recurrence. Many patients feel they are "better" and stop taking medication without authorization, but this often disrupts the chemical balance that has just been established in the brain, leading to a resurgence of the disease.

Experiencing major life stress: Such as job loss, breakup, death of a loved one, severe family conflicts, or work difficulties—these stressful events are powerful catalysts for recurrence.

Lack of social and family support: If the patient feels isolated and unsupported during recovery, this psychological pressure will increase the risk of recurrence.

Unhealthy lifestyle habits: Long-term staying up late, irregular work and rest, excessive drinking, and substance abuse all have a negative impact on mental state.

Discontinuing psychotherapy: Medication mainly controls physical symptoms, while psychotherapy (such as CBT) helps patients learn to manage emotions, cope with stress, and correct irrational thinking patterns. Discontinuing psychotherapy means "rustiness" in coping skills.

Nature of the disease itself: Certain mental illnesses (such as bipolar disorder, schizophrenia) have a relatively high inherent risk of recurrence.

2. How to Effectively Prevent Recurrence?

The good news is that recurrence is largely preventable. Recovering individuals can take many active measures to maintain stability and greatly reduce the risk of recurrence:

Adhere to medication as prescribed by the doctor (maintenance treatment): This is the cornerstone of preventing recurrence. Even if you feel well, you can only adjust the medication plan after discussing with your doctor. Usually, patients with a first episode need to continue taking medication for 1-2 years or even longer after symptoms disappear.

Maintain regular follow-up visits and psychotherapy: Regular communication with doctors or psychotherapists can not only monitor the condition but also continuously learn new coping strategies, handle small problems in life in a timely manner, and prevent them from evolving into major crises.

Learn stress management: Learn relaxation techniques such as mindfulness, meditation, and deep breathing, and develop healthy hobbies (such as exercise, music, painting) to find a healthy outlet for emotions.

Establish a healthy routine: Ensure adequate sleep, a balanced diet, and regular physical exercise. These are crucial for stabilizing mood and brain function.

Build a strong social support system: Maintain good communication with family and friends, and let them understand your situation and needs. Participating in support groups and exchanging experiences with other recovering individuals can also provide great psychological strength.

Recognize early signs of recurrence: This is very crucial. Understand what "signals" usually appear before your recurrence.

3. Recognizing Early Signs of Recurrence

Everyone’s early signs may be different, but common ones include:

Changes in sleep patterns: Insomnia, early awakening, or excessive sleepiness.

Increased mood swings: Becoming irritable, anxious, depressed, or abnormally excited.

Social withdrawal: Not wanting to see people or participate in activities you used to enjoy.

Changes in appetite: Loss of appetite or overeating.

Difficulty concentrating and memory loss.

Reappearance of some mild, previous symptoms.

Once you notice these signals, do not panic or blame yourself—treat them as a "yellow light warning" that needs timely handling.

4. What to Do If You Feel a Recurrence May Be Happening?

Seek professional help immediately: Contact your attending doctor or psychotherapist as soon as possible and tell them how you feel. They can help you adjust the treatment plan—sometimes just a slight adjustment of medication dosage or increasing the frequency of psychological counseling can nip the recurrence in the bud.

Inform family or trusted friends: Let them know you need support.

Review recent life: Has there been too much stress recently? Or have you stopped exercising? Identify possible triggering factors and make adjustments.

In summary, recovering from a mental illness does face the challenge of recurrence, but this is by no means a failure. Recovery is an ongoing process of management and learning, just like managing any chronic disease. Through adhering to treatment, living a healthy life, and seeking help actively, the vast majority of patients can achieve long-term stability and live a productive and satisfying life.

**Q29 How to Prevent the Recurrence of Mental Illnesses?**

Hello, this is a very important question. Preventing the recurrence of mental illnesses is a systematic process that requires the joint efforts of patients, families, and professionals. Recurrence does not mean failure, but part of the recovery process—the key lies in how to actively respond and prevent effectively.

Here are several core prevention strategies, which you can regard as a multi-layered protection system:

1. Adhere to Professional Treatment—The Cornerstone

Take medication as prescribed by the doctor: This is the most important link in preventing recurrence. Many recurrences of mental illnesses are related to reducing or stopping medication without authorization. Even if you feel you have "recovered," you must take the medication strictly as directed by the doctor. The role of medication is to stabilize the brain’s chemicals; stopping medication without permission may cause the condition to rebound rapidly.

Regular follow-up visits and psychotherapy: Communicate regularly with doctors and psychotherapists to keep them informed of your recent situation. This is not only to adjust medication but also to:

Learn coping skills: Psychotherapy (such as Cognitive Behavioral Therapy, CBT) can help you identify and change thinking patterns that lead to negative emotions and behaviors.

Handle psychological stress: Learn how to manage stress and resolve conflicts in interpersonal relationships.

Consolidate recovery effects: Therapists will help you consolidate the skills and positive changes learned during the recovery period.

2. Learn Self-Management—Take the Initiative to Master Your Own State

Recognize "early warning signs of recurrence": Recurrence usually does not happen suddenly; there are some early signs. Everyone’s warning signs may be different, but common ones include:

Changes in sleep patterns: Insomnia, early awakening, or excessive sleepiness.

Mood swings: Irritability, irritability, low mood, or abnormal excitement for no reason.

Decreased interest: Loss of interest in things you used to like and reluctance to socialize.

Physical discomfort: Unexplained fatigue, headaches, and changes in appetite.

Reappearance of old symptoms: Some mild symptoms that once troubled you reappear.

Recommendation: Try keeping an "emotion diary" to record your mood, sleep, stress, etc., every day. This helps you and your doctor detect changes early.

Maintain a healthy lifestyle: This is the foundation for enhancing physical and mental resilience.

Regular work and rest: Ensure adequate and regular sleep, which is a natural medicine for stabilizing mood.

Balanced diet: Avoid overeating and reduce caffeine and sugar intake.

Adhere to exercise: Regular physical exercise (such as walking, jogging, yoga) can release endorphins, a natural "antidepressant."

Abstain from alcohol, tobacco, and drugs: Alcohol and drugs will seriously affect the effectiveness of medication and directly damage the brain’s stability—they are major triggers for recurrence.

Effective stress management: Learn to identify stressors in life and find stress reduction methods that suit you, such as:

Mindfulness meditation and deep breathing exercises.

Cultivate a hobby that allows you to immerse yourself (such as gardening, painting, music).

Contact nature, such as walking in the park or hiking.

3. Build a Strong Social Support System—You Are Not Alone in the Fight

Communicate with family and friends: Let your trusted family and friends understand your illness, early warning signs of recurrence, and how they can help. Open and honest communication can reduce misunderstandings and make them allies on your road to recovery.

Participate in support groups: Communicating with people who have similar experiences can greatly reduce loneliness. In these groups, you can share experiences, encourage each other, and learn practical coping strategies from others.

Create a low-stress environment: Adjust your work and living environment as much as possible to avoid long-term exposure to high-pressure or conflict-ridden atmospheres. Learn to set boundaries and say "no" to unreasonable requests.

4. Develop a "Crisis Response Plan"—Be Prepared for Emergencies

Work with your doctor and family to develop a clear plan specifying what to do if you feel your condition is deteriorating or showing signs of recurrence. This plan may include:

Emergency contact list: Phone numbers of doctors, therapists, and trusted family/friends.

List of early warning signs of recurrence: List your own unique warning signs.

Steps to seek help: For example, "When I have insomnia for three consecutive days, I should call my doctor immediately" or "If my family finds that I start avoiding communication, they should remind me and accompany me to see the doctor."

Ways to soothe yourself: List activities that can calm you down when you feel unwell.

Finally, please remember:

Recovery is a continuous journey, not a destination. Preventing recurrence requires patience and effort, but by combining professional treatment, self-management, and strong social support, you can minimize the risk of recurrence and live a stable, fulfilling life. Seeking help is a sign of wisdom and courage—please do not hesitate.

**Q30 Will Mental Illnesses Be Inherited by the Next Generation?**

This is a very important and complex question. The simple answer is: Mental illnesses have a genetic tendency, but they are not absolutely inherited by the next generation.

We can understand it as follows: Heredity only increases the "risk" or "possibility" of the next generation developing the disease, not an inevitable outcome. This involves the complex interaction between genetic and environmental factors.

To better understand, we can look at it from the following aspects:

1. Genetic Factors

Scientific research has clearly shown that many mental illnesses, such as schizophrenia, bipolar disorder, major depression, and anxiety disorders, have a genetic basis.

Increased risk: If a person’s immediate relatives (such as parents or siblings) have a certain mental illness, their risk of developing the same or related diseases is higher than that of the general population.

Polygenic influence: The inheritance of mental illnesses is usually not determined by a single gene, but by the cumulative effect of multiple genes with small effects. You may inherit certain "risk genes," but this does not mean you will definitely develop the disease.

Different heritability for different diseases:

Schizophrenia and Bipolar Disorder have relatively high heritability. For example, if one parent has schizophrenia, the child’s risk of developing the disease is about 10% (compared to 1% in the general population). If one identical twin has the disease, the other’s risk is as high as 40%-50%.

Major Depressive Disorder also has a genetic tendency, but environmental factors may play a more important role.

2. Environmental and Other Factors

Genetic genes are like loading a gun, but it is often environmental factors that pull the trigger. With only genetic risk, the disease may never manifest without corresponding environmental "triggers." These factors include:

Growth environment: Negative experiences in childhood, such as emotional neglect, physical or sexual abuse, school bullying, domestic violence, etc.

Social stress: Long-term and significant pressure in studies, work, interpersonal relationships, etc.

Stressful life events: Major changes such as the death of a loved one, job loss, or serious accidents.

Substance abuse: The use of alcohol, drugs, etc., will significantly increase the risk of illness, especially in people with a genetic predisposition.

Physical health status: Certain chronic diseases or physiological damage to the brain may also induce mental problems.

Summary and Recommendations

Overall, the occurrence of mental illnesses can be seen as a "gene-environment interaction" model.

Heredity deals you a hand of cards, but how you play them largely depends on your environment, experiences, and choices.

What should you do if you have a family history of mental illness?

Do not be overly anxious: Remember, risk is not fate. The vast majority of people with a family history will not develop severe mental illnesses.

Improve health awareness: Learn about relevant early symptoms and pay attention to changes in your emotions and behaviors. If you feel persistent distress, do not hesitate to seek professional help early.

Cultivate a healthy lifestyle:

Learn effective stress management skills (such as mindfulness, meditation, exercise).

Maintain a regular schedule and adequate sleep.

Build a solid social support system (maintain good communication with family and friends).

Avoid alcohol and drugs.

Create a good environment for the next generation: If you plan to have children, pay more attention to providing them with a stable, loving, and safe growth environment, which can largely offset the risks brought by heredity.

Please remember that mental illnesses, like many other chronic diseases (such as hypertension and diabetes), are preventable and treatable. Understanding family history is to better prevent and manage risks, not to bear a heavy psychological burden.

**Q31 How Can Patients with Mental Illnesses Better Reintegrate into Society?**

This is a very important and far-reaching question. The social reintegration of patients with mental illnesses is a complex but hopeful process that requires the joint efforts of individuals, families, professionals, and the entire society. It is not a simple linear process of "cure" followed by "reintegration," but a continuous, multi-faceted recovery journey.

The following are key points from different levels that can help patients with mental illnesses better reintegrate into society:

I. Cornerstone: Adhere to Scientific and Systematic Professional Treatment

This is the premise and foundation for all social reintegration. Without stable conditions, nothing else is possible.

Follow medical advice and stabilize the condition: Taking medication on time and in the correct dosage is crucial for controlling symptoms and preventing recurrence. Have regular follow-up visits and communicate frankly with doctors about changes in the condition and medication side effects to adjust the treatment plan in a timely manner.

Receive psychotherapy: Medication can control symptoms, but psychotherapy (such as Cognitive Behavioral Therapy, Dialectical Behavior Therapy, Family Therapy, etc.) can help patients understand their illness, learn emotional management and stress coping skills, repair psychological trauma, and rebuild cognitive patterns.

II. Core: The Patient’s Own Efforts and Reconstruction

Internal motivation is the most powerful driving force for social reintegration.

Face the illness squarely and accept oneself: Correctly understand that mental illness is a chronic disease like hypertension and diabetes; it is not a character flaw or moral issue. Reducing self-stigma and accepting oneself as a sick person is the first step toward recovery.

Rebuild a regular life rhythm: Start with the simplest things, such as ensuring a regular schedule and diet, appropriate physical exercise, and taking care of personal hygiene. These seemingly trivial things can bring a sense of control and stability.

Set feasible short-term goals: Do not rush for success; set some achievable small goals. For example, "Take a 20-minute walk today," "Organize my room once this week," or "Try calling a friend." Completing each goal is a positive incentive.

Learn and practice social skills: If you have been out of society for a long time, your social skills may become rusty. You can start by interacting with family and trusted friends, or join some support groups to re-learn how to communicate and build relationships in a safe environment.

III. Key Support: Effective Help from Family and Friends

Family and friends are the patient’s most direct and important support system.

Learn relevant knowledge: Understand the knowledge of the patient’s illness and recognize that their behaviors and emotions may be affected by the illness, not intentional. This can reduce many unnecessary misunderstandings and conflicts.

Provide emotional support rather than overprotection: Give the patient unconditional love, patience, and encouragement. Listen to their feelings but avoid overindulgence and doing everything for them. Encourage them to make their own decisions and try new things, allowing them to regain confidence through practice.

Create a low-stress family environment: A stable, predictable, and safe family environment is crucial for recovery. Avoid accusations, quarrels, and excessive expectations.

Encourage and accompany participation in social activities: When the patient is ready, you can invite or accompany them to participate in small, relaxed family gatherings or friend activities to help them break isolation.

IV. Professional Assistance: Diversified Rehabilitation Services

In addition to hospital treatment, professional social rehabilitation services are a bridge to social reintegration.

Community rehabilitation centers/day hospitals: These institutions provide structured rehabilitation activities, such as handicrafts, music, gardening, sports, etc., to help patients have a regular place to go during the day, maintain functions, and establish connections with other recovering individuals.

Peer support groups: Mutual assistance groups composed of recovering individuals with similar experiences. Here, patients can share experiences, gain understanding and empathy, see role models of successful recovery, and greatly enhance confidence.

Vocational rehabilitation and supported employment: Professional vocational rehabilitation counselors can help patients assess their vocational interests and abilities, provide vocational skills training, and assist in finding "supported employment" positions—positions where employers understand and are willing to provide necessary support and convenience for patients (such as flexible working hours, low-stress working environments, etc.). Starting with volunteer work or part-time jobs is also a good transition.

V. Social Environment: Soil of Tolerance and Opportunities

Personal efforts and family support require an inclusive social environment to take root.

Eliminate stigma and popularize mental health knowledge: Through public education and media publicity, let more people understand the truth about mental illnesses, recognize that it is a disease that requires treatment, not a terrifying and shameful "madness."

Provide equal employment and educational opportunities: Governments and enterprises should formulate and implement anti-discrimination policies to provide equal learning and working opportunities for recovering patients. A job that is willing to accept them is the key to their economic independence and self-worth realization.

Improve the community support system: Governments should invest more resources to establish a sound mental health service network at the community level, including consulting centers, rehabilitation institutions, crisis intervention hotlines, etc., to make help easily accessible.

Summary

The road for patients with mental illnesses to reintegrate into society is a collaborative effort from the inside out.

For patients: It is a journey of "accepting oneself and making small steps forward."

For families: It is a process of "understanding, learning, and patient companionship."

For society: It is a responsibility of "eliminating prejudices and providing opportunities."

When these three forces work together, social reintegration is no longer an unattainable goal, but a hopeful path that can be steadily followed.

**Q32 How to Cope with Discrimination and Remarks Against Patients with Mental Illnesses?**

This is a very important and realistic question. When people face discrimination and unfriendly remarks against mental illnesses, they will feel hurt, angry, and helpless, which will undoubtedly increase the difficulties in the recovery process.

First, please allow me to express my understanding and support to you or someone you care about. There is no single "correct answer" to coping with this situation, but we can explore some effective strategies from several levels. The core principle is: Your safety and mental health are always the top priorities.

Level 1: Stabilize Yourself—Internal Coping Strategies

Before deciding how to deal with the outside world, take good care of your inner self.

Recognize that stigma stems from ignorance, not your fault.

Please remember that mental illness is a real health condition that requires scientific treatment, just like diabetes or heart disease. It is not a character flaw, weakness of will, or "overthinking." Others’ discrimination and remarks reflect their prejudices and ignorance, not your worth.

Allow yourself to have emotions and practice self-care.

Feeling hurt, angry, or frustrated is a completely normal reaction. Do not blame yourself for these emotions. Give yourself time and space to feel and process them, such as releasing pressure through journaling, talking to trusted people, exercising, or meditating.

Strengthen your support system.

This is the most powerful weapon against discrimination. Ensure that you have people around you who understand and support you—they can be family, close friends, partners, counselors, or members of support groups. When you are hurt, these people are your safety net.

Focus your energy on recovery.

Your top priority is your own health. Do not feel obligated to "educate" everyone who discriminates against you. Devote your precious energy to following the treatment plan, maintaining a healthy lifestyle, and doing things that make you feel good.

Level 2: Make a Choice—How to Directly Respond to Discrimination and Remarks

When you are directly faced with unfriendly remarks, you can choose different coping methods based on the specific situation, who the other person is, and your current state.

Before taking action, quickly assess:

Who is the other person? (A stranger, colleague, or close family member/friend?)

What is the occasion? (Public or private communication?)

How are you feeling? (Do you have the energy to respond now, or are you feeling vulnerable?)

What is your goal? (Do you want the other person to stop, or do you want them to understand?)

The following are specific coping methods that you can use flexibly like tools from a toolbox:

Strategy 1: Ignore and Disengage

Applicable situation: The other person is a stranger, internet troll, or you are exhausted and do not want to invest any energy.

Approach: You have every right not to respond or argue. Walk away silently, close the chat window, or block the other person. Your time and energy are more valuable than arguing with ignorance.

You can say: (Say nothing and leave directly) or simply: "I don’t want to talk about this topic."

Strategy 2: Set Clear Boundaries

Applicable situation: The other person is a colleague, relative, or friend you need to interact with, and you need them to stop this behavior.

Approach: Use calm, firm, but non-aggressive language to clearly tell the other person that their remarks make you uncomfortable and ask them to stop.

You can say:

"My health status is my privacy. I don’t want it discussed at work/family gatherings. I hope you respect that."

"What you just said made me very uncomfortable. Please don’t comment on my condition like that in the future."

"I know you may not have meant harm, but this statement is actually a prejudice. Can we talk about something else?"

Strategy 3: Brief Opportunity for Education

Applicable situation: The other person is someone you care about (such as a family member or close friend), you believe they are only misunderstood, and you have enough energy to explain.

Approach: Explain mental illness in a simple, analogical way. Avoid using complex professional terms.

You can say:

"Actually, mental illness is very similar to physical illness. The brain is also an organ, and it can get sick too. This requires the help of doctors and medication; it’s not something that can be solved by 'cheering up.'"

"Thank you for your concern. For me, it’s more helpful to view this as a health issue that needs to be managed."

Strategy 4: Firmly Challenge Stigma

Applicable situation: You feel in a good state, strong, and want to correct misinformation and speak up for yourself and others.

Approach: Directly point out the errors and prejudices in the other person’s remarks.

You can say:

The term "psycho" is highly discriminatory. My diagnosis is depression/anxiety disorder, which is a common health problem.

Linking violent behavior to mental illness is a very common but incorrect stereotype. The vast majority of patients with mental illnesses have no violent tendencies; on the contrary, they are more likely to be victims of harm.

Strategy 5: Seek Help Through Formal Channels

Applicable situation: Experiencing institutional discrimination at work, school, or other institutions, such as being fired or treated differently because of it.

Approach: Preserve relevant evidence (such as emails, message screenshots) and seek help from the company’s human resources department, the school’s administration, or relevant legal aid organizations and disability rights protection organizations.

Summary and Encouragement

Please remember that you are not alone in this fight. More and more people are working to eliminate the stigma of mental illness.

You have the right to choose not to respond. Silence is not weakness, but protection of yourself.

Your feelings are valid. Do not let others’ ignorance define you.

Seeking support is a sign of strength. Whether it is professional psychological support or warmth from friends and family, please actively embrace it.

Most importantly, please take good care of yourself. Your recovery and happiness are more important than any unfriendly remarks in the world.

**Q33 Can You Date Someone with a Mental Illness?**

Of course you can.

Dating someone with a mental illness, like dating anyone else, is ultimately about love, respect, communication, and mutual support. It may be easier to understand by analogy to any chronic physical illness (such as diabetes, asthma, or heart disease): it is a health condition that needs to be understood, managed, and supported, but it does not define a person’s entire being.

This is a complex but potentially very meaningful choice. The following are key considerations to help you think about this question:

Positive Aspects to Understand

They are first and foremost an individual: Their illness is only part of them, not their entire identity. They have their own personality, hobbies, dreams, and strengths. You fall in love with the person, not their illness.

It may bring deeper empathy and connection: People who have experienced inner struggles often have a deeper understanding of emotions. Accompanying them through difficult times can build an incredible sense of intimacy and trust.

It can make you a better, more mature partner: Such a relationship will urge you to learn patience, communication, and selfless love, giving you a deeper understanding of the complexity of human nature.

Realistic Challenges and Necessary Preparation

Learn and understand: You need to take the initiative to understand what their specific diagnosis is (such as depression, anxiety disorder, bipolar disorder, etc.). Understanding its symptoms, triggers, and treatment methods can help you better understand their behaviors instead of misunderstanding or blaming them.

Distinguish between "them" and "the illness": At certain times, you need to be able to tell which behaviors are symptoms of the illness (such as withdrawal and indifference during a depressive episode) and which are real problems in your relationship. This requires communication and patience.

Establish strong communication: You need to be able to talk openly about feelings—both yours and theirs. The other person needs a safe space to express their struggles, and you also need to be able to honestly share your exhaustion, worries, or needs without feeling guilty.

You are a partner, not a therapist: This is the most important point. Your role is to support, accompany, and love, not to treat. Encourage and support them to seek professional help (such as psychological counseling, medication, etc.) and persist in it. Leave the responsibility of treatment to professionals.

Take care of yourself: Supporting a partner struggling with a mental illness can be extremely energy-consuming. You must build your own support system (friends, family, or even your own counselor) to ensure you have time to rest and recover. If you are exhausted, you cannot provide any support.

Set healthy boundaries: Love is inclusive, but not without bottom lines. You need to clarify which behaviors you can accept and which you cannot. Mental illness cannot be an excuse for emotional abuse, manipulation, or irresponsible behavior.

Warning Signs to Be Alert To

In any relationship, you need to re-evaluate if the following situations occur, which have nothing to do with mental illness itself but with the health of the relationship:

The other person refuses to acknowledge the problem or any form of professional help.

The other person blames all problems on the illness and uses it as an excuse to hurt you or shirk responsibility.

Your own mental health and quality of life are seriously and continuously deteriorating because of this relationship.

The relationship becomes one-sided giving, and you feel more like a "caregiver" than a "partner."

Conclusion

Yes, but it requires wisdom and commitment.

It is completely possible to build a healthy, long-lasting relationship with someone with a mental illness. The key to success lies in both parties being willing to take responsibility for the relationship and their own health:

The other person needs to actively manage their condition.

You need to provide understanding and support while taking care of yourself.

Ultimately, every relationship has its unique challenges. Whether a relationship can succeed does not depend on whether one party has the "label" of mental illness, but on universal principles such as love, respect, communication, and joint efforts.

**Q34 Can Patients with Mental Illnesses Get Married?**

According to China’s current laws, patients with mental illnesses can get married, but they need to meet certain key conditions.

The core criterion is no longer "what illness they have," but whether the person has full capacity for civil conduct at the time of marriage registration and whether they have fulfilled the obligation of disclosure.

Specifically, there are the following points:

Core Condition: Having Full Capacity for Civil Conduct

According to the Civil Code of the People’s Republic of China, both parties to a marriage must be "fully voluntary."

This means that the person must be able to clearly understand the nature and consequences of marriage and independently and truthfully express their wishes.

If a patient with a mental illness is in a normal mental state or has a stable condition and can think independently and make decisions when applying for marriage registration, they are deemed to have full capacity for civil conduct and can get married.

On the contrary, if the patient is in an acute episode of the illness and cannot recognize their own actions or express their true wishes, they do not have capacity for civil conduct and cannot register for marriage.

Obligation to Disclose Major Illnesses

Article 1053 of the Civil Code stipulates: "If one party suffers from a major illness, they shall truthfully inform the other party before marriage registration; if they fail to do so, the other party may request the people’s court to revoke the marriage."

Severe, un cured mental illnesses are usually regarded as "major illnesses."

Therefore, the party with a severe mental illness has the obligation to inform the other party of the condition before marriage. The law protects the other party’s "right to know."

If they choose to conceal it, the other party has the right to apply to the court for revocation of the marriage within one year after learning the truth.

Summary:

Can get married: As long as the person is conscious, understands what marriage entails, and truly wishes to marry at the time of registration.

Need to disclose: If the illness falls under the category of a major illness, it must be disclosed to your fiancé/fiancée in advance. Concealing the condition may lead to the revocation of the marriage.

Overall, China’s law adopts a more humanized and respectful attitude towards patients with mental illnesses regarding marriage, changing from "prohibition" in the past to "protecting the right to know and voluntary marriage" now. The law protects every citizen’s freedom of marriage on the premise that the person can be responsible for their own decisions.

**Q35 How to Get Along Better with Family Members with Mental Illnesses?**

This is a very important and challenging question. Getting along with family members with mental illnesses is a path that requires great patience, love, and wisdom. It not only tests your family bond but also profoundly affects your own life.

Please remember that you are not facing this alone. The following are key principles and practical suggestions to help you and your family go further smoothly:

1. First, Arm Yourself: Learn and Understand

"Knowing yourself and your enemy" is the first step. The more you understand, the less fear and misunderstanding you will have.

Learn about the specific illness: What is their diagnosis? Is it depression, bipolar disorder, anxiety disorder, or schizophrenia? Each illness has very different symptoms, courses, and treatment methods. Read professional books, consult reliable medical websites (such as WHO, National Health Commission), and ask doctors for advice.

Separate the "person" from the "illness": This is the most important point. You are fighting against the "illness," not your family member. When he/she says hurtful things or behaves in an incomprehensible way, please tell yourself mentally: "This is the illness speaking, not their true intention." This can help you reduce the emotional drain of taking things personally.

Understand "stigma": Your family member may feel ashamed, inferior, or afraid because of the illness. This "stigma" will make him/her resist treatment and be unwilling to talk about the condition. Understanding this heavy psychological burden is the beginning of building trust.

Accept the long-term nature of recovery: The recovery process of mental illnesses is often long and fluctuating, like a winding line rather than a straight one. There will be progress and possible setbacks. Please be prepared for a "protracted war" and celebrate every small progress.

2. Adjust Communication Methods: Be a "Supporter" Rather Than a "Preacher"

Effective communication is the "lubricant" of relationships.

Use "I" statements: Avoid accusatory words starting with "you."

Instead of: "You’re so lazy for not getting up again!"

Say: "I noticed you didn’t get up today. I’m a little worried if you’re feeling unwell."

Focus on listening rather than rushing to give advice: Many times, what they need is not a solution, but a safe, non-judgmental space to talk. Listen carefully and respond with "Hmm," "I see," or "That must have been really hard," to show that you understand and empathize.

Choose the right time: Do not have serious conversations or discuss issues when he/she is emotionally agitated or showing obvious symptoms. Choose a time when both parties are relatively calm.

Stay calm and patient: Even if you feel frustrated or angry, try to control your emotions. Your calmness itself is a form of comfort. If you feel you are losing control, you can temporarily leave and tell yourself: "I need to calm down and come back later."

3. Set Healthy Boundaries: Protect Yourself to Better Protect Them

Love without boundaries is "spoiling," which will eventually exhaust you.

Clarify your bottom line: What behaviors can you not accept? Is it verbal abuse, physical violence, or excessive financial demands? You must clearly, calmly, but firmly tell the other person your bottom line. For example: "You can feel angry, but you cannot yell at me."

Distinguish responsibilities: Ultimately, their illness and emotions are their own responsibility. You can provide support, help, and love, but you cannot "cure" them or be responsible for their life. Encourage and assist them to seek professional treatment (see a doctor, have counseling), but do not make yourself the only "savior."

Protect your own life: Your life should not revolve solely around "taking care of the patient." Continue your work, hobbies, and social interactions. Spend time doing things that make you happy and relaxed. This is not only for yourself but also to give your relationship "breathing space."

4. Learn to Take Care of Yourself: You Are the Most Important "Resource"

If the caregiver collapses, the entire support system will crumble.

Acknowledge your emotions: Taking care of a sick family member will make you feel helpless, exhausted, angry, guilty, sad... These are all completely normal emotions. Accept them instead of suppressing them.

Seek your own support: Talk to friends, other family members, or a counselor. You also need a place to vent your stress and confusion. Finding a "caregiver support group" is also a very good option, where you will find many people in the same situation as you.

Ensure adequate rest: Get enough sleep, eat healthily, and exercise moderately. Your physical and mental health comes first.

5. Develop a Crisis Plan: Be Prepared for Emergencies

Prepare in calm times to avoid panic in storms.

Understand crisis signals: Work with doctors to learn which signs (such as talking about suicide, violent tendencies, loss of control) indicate that a crisis may be imminent.

Prepare a contact list: Keep the phone numbers of the attending doctor, psychotherapist, and local mental health center or crisis intervention hotline readily available.

Agree on a plan: When the family member’s condition is stable, you can try to agree with him/her: If an emergency occurs, what should we do? Who is his/her trusted contact? Which hospital should we go to?

Finally, please remember: Love is the foundation, but love alone is not enough. Scientific methods, healthy boundaries, and care for yourself are all indispensable.

This is a difficult journey, but through learning and effort, you can establish a new, healthier way of getting along with your family. Please be kind to yourself, because you are already doing very well.

**Q36 How to Judge if a Family Member Has a Relapse of Mental Illness?**

Hello! Caring about your family member’s mental health and being sensitive to signs of relapse is in itself a very important and caring first step. Early identification of signs of relapse and taking action is crucial for helping your family member get through difficult times smoothly and avoiding the deterioration of the condition.

The core principle for judging if a family member has a relapse of mental illness is: Compare their current state with their "baseline state" when the condition is stable. Everyone’s performance during the stable period is unique; once their behavior, emotions, or thinking patterns deviate significantly from this "normal" baseline, it is necessary to be alert.

The following are common early warning signs of relapse that you can observe from these aspects:

1. Significant Changes in Emotions

This is one of the easiest signals for family members to detect.

Elevated or depressed mood: Unreasonably abnormally excited, talkative, and energetic for no reason, or on the contrary, persistently low mood, pessimistic and hopeless, and lack interest in anything.

Mood swings and irritability: Temper becomes irritable, flies off the handle over trivial matters, or becomes overly sensitive, suspicious, and prone to conflicts with others.

Increased anxiety or fear: Shows persistent restlessness, inability to relax, and irrational fear of specific situations or things.

Emotional apathy: Feels numb, lacks enthusiasm for anything, and becomes emotionally distant from relatives and friends.

2. Changes in Behavioral Patterns

Behavior is a direct reflection of inner state, and these changes are usually specific.

Sleep problems: Insomnia (difficulty falling asleep, frequent awakening at night, early awakening) or excessive sleepiness are extremely common signals. For example, someone who used to sleep well stays up all night for several consecutive days, or sleeps all day.

Social withdrawal: Starts to avoid social interactions, refuses to meet people, locks themselves in the room, and declines to participate in family activities.

Changes in personal hygiene and appearance: Suddenly becomes unkempt, does not take a bath or change clothes for a long time, and the room is messy.

Changes in appetite: Overeating or loss of appetite, leading to significant weight changes in a short period of time.

Appearance of abnormal or stereotyped behaviors: May have some strange, repetitive movements or ritualistic behaviors.

Decreased medication adherence: Starts to resist, forget, or refuse to take maintenance treatment medication. This is a very dangerous and strong signal of relapse.

3. Abnormalities in Thinking and Speech

This part may be more subtle and requires careful listening and observation.

Confused thinking and poor concentration: When talking, you feel that the other person’s train of thought is "broken," jumps between topics, and is difficult to understand. Or shows inability to concentrate and memory loss in work or study.

Appearance of delusions or paranoid ideas: Firmly believes in ideas that do not conform to facts and cannot be persuaded. For example, firmly believes that someone is trying to harm him/her, or that people around are talking about him/her.

Abnormal speech: Increases self-talk, or the content of speech is bizarre and illogical.

Abnormal perceptions (hallucinations): Mentions seeing or hearing things that others cannot perceive (such as hearing voices or seeing figures out of thin air).

4. Physical Discomfort

Sudden changes in energy levels: Feels extremely tired, physically drained, or overly energetic and tireless.

Unexplained physical symptoms: Often complains of headaches, gastrointestinal discomfort, body pain, etc., but no specific cause can be found in physical examinations.

What Can Family Members Do After Detecting Signs of Relapse?

Stay calm and observe carefully: Do not panic or accuse immediately. First, observe and record silently for a few days to confirm that these changes are persistent, not accidental mood swings lasting one or two days.

Choose the right time for gentle communication: Find a time when both parties are relatively calm and relaxed, and start the conversation in a caring rather than questioning tone. You can say: "I noticed you haven’t been sleeping well/looking happy lately. I’m a little worried about you. Did something upsetting happen?" Avoid saying: "Did you have a relapse again?"

Encourage and assist in seeking medical treatment: This is the most important step. Gently remind him/her that these "uncomfortable" feelings may be signs of fluctuating conditions, and suggest or directly help him/her make an appointment for a follow-up visit with a psychiatrist. If the other party resists, you can emphasize "just go talk to the doctor to see how we can all feel better."

Pay attention to medication adherence: On the premise of not causing conflict, you can indirectly find out if the other party is taking medication on time. You can say: "Let me get you a glass of water. Is it time to take your medicine?" Create a supportive family atmosphere.

Handle emergencies: If the family member makes remarks or behaviors that harm themselves or others, or shows extreme emotional outbursts, this is an emergency. At this time, you should immediately seek professional help, including:

Contact their attending doctor.

If necessary, call the emergency number (such as 120) or the police (110), and clearly inform the other party that it is a mental health-related emergency.

Ensure the safety of the home environment and remove dangerous items.

Take care of yourself: As a family member, your physical and mental health is also important. This process may make you feel extremely stressed and exhausted. Please remember to seek support, whether from other family members, friends, or professional counselors. Only by taking care of yourself can you have the strength to better support your family member.

Important Reminder: The above information is for reference only and cannot replace professional medical diagnosis. The final judgment and treatment plan must be made by a professional psychiatrist. Your observation and timely action are crucial for providing doctors with important information and helping family members receive the best treatment.

**Q37 What to Do When a Family Member Experiences Hallucinations?**

When a family member experiences hallucinations, it is crucial to stay calm and adopt the right supportive approach. This not only helps the family member get through the current difficulties but also is the first step in seeking long-term solutions.

Hallucinations are absolutely real for the experiencer; directly denying or arguing with them ("There are no voices at all—you’re overthinking!") is usually ineffective and may even increase their anxiety, fear, and sense of isolation.

The following are specific coping steps and suggestions, divided into "Immediate Response" and "Long-Term Strategies".

I. Immediate Response: What to Do When Hallucinations Occur

When a family member tells you they are hearing something, you can do the following:

Stay Calm: Your calmness is an important emotional stabilizer for your family member. If you show panic, it will increase their fear. Take a deep breath and calm yourself down.

Don’t Argue or Judge: Don’t say "That’s not real" or "Stop imagining things." This will make them feel misunderstood. Remember, for them, this experience is real.

Show Empathy and Understanding: Acknowledge their feelings, not the reality of the voices. You can say:

"It sounds like this voice is making you feel uncomfortable/scared."

"Although I can’t hear it, I believe you can, and it must be distressing for you."

"I’m here with you—you’re not alone."

Provide Reassurance: In a gentle tone, tell them: "You are safe now, and I will protect you here." A simple physical touch, such as patting their back or holding their hand (if they are not resistant), can also convey support.

Gently Shift Focus: After their emotions have stabilized a little, you can try to gently redirect their attention to the real world.

"Shall we listen to some music together?"

"Would you like a glass of warm water or something you like to eat?"

"We can chat about something happy that happened today."

Assess for Safety: This is a very important step. You need to understand the content of the hallucinations:

If the voice is commanding, ordering the family member to harm themselves or others (for example, "The voice is telling me to hurt myself"), this is an emergency. You need to immediately seek professional help, such as calling the emergency number (such as 120) or taking them to the psychiatric emergency department of a hospital. Ensure that the family member is accompanied at all times during this period and remove dangerous items.

If the voice is only commentative or conversational, the risk is lower, but it still requires close attention.

II. Long-Term Strategies: What You Need to Know and Do

Hallucinations are usually a symptom of an underlying problem, not the illness itself. Therefore, in the long run, the most crucial thing is to identify the root cause and treat it.

Seek Professional Medical Help: This is the most critical step. Take your family member to see a doctor as soon as possible. You can choose based on the situation:

Psychiatry: If schizophrenia, depression, bipolar disorder, or other mental health issues are suspected, this is the first choice. Doctors can make a diagnosis and provide medication treatment plans.

Neurology: If the family member has other neurological symptoms (such as severe memory loss, abnormal limb movement, etc.), it may be necessary to rule out brain tumors, epilepsy, Alzheimer’s disease, and other problems.

Psychotherapy/Counseling: Psychotherapy (especially Cognitive Behavioral Therapy, CBT) can help patients learn skills to manage and cope with hallucinations, reducing their impact.

Keep a Log: Before seeing a doctor, you can help the family member record details of the hallucinations, which will provide valuable information for the doctor’s diagnosis:

Timing and frequency: When are they most likely to occur? (Day/night? When alone/under stress?)

Content: What are the voices saying? Is it one or multiple voices? Are they familiar or unfamiliar?

Emotional response: How does the family member feel after hearing the voices? (Scared, irritable, calm?)

Coping methods: How does the family member cope? What methods seem to work?

Be a Reliable Supporter:

Encourage and accompany treatment: Ensure that the family member takes medication on time and has regular follow-up visits. Medication treatment may take some time to take effect and may have side effects—your encouragement is crucial.

Create a low-stress environment: Stress and strong environmental stimuli may induce or worsen hallucinations. Try to maintain a calm and harmonious family environment.

Learn relevant knowledge: Understanding the illness your family member has been diagnosed with can help you better understand their behaviors and provide more effective support.

Take care of yourself: As a caregiver, your physical and mental health is also important. Find a support system (other family members, friends, or support groups) to ensure you have time to rest and recharge, avoiding "burnout."

Summary:

Do: Stay calm, show empathy, provide reassurance, redirect focus, assess risk, and seek professional help.

Don’t: Argue, mock, criticize, ignore, or bear all the pressure alone.

Please remember that you and your family member are not fighting alone. Hallucinations are a symptom that can be effectively managed through modern medical and psychological methods. Seeking help in a timely and correct manner is the first and most important step toward recovery.

**Q38 What to Do If a Patient with Mental Illness Shows Suicidal Behavior?**

This is a very serious and urgent situation. When a patient with mental illness shows suicidal behavior or expresses suicidal thoughts, the most important step is to immediately seek professional help and ensure their safety. Your role is to be a first responder and supporter in an emergency, not a therapist.

The following is a specific, step-by-step action guide:

Step 1: Take Immediate Action (When Danger Is Imminent)

If the patient is attempting suicide, has clearly stated they are going to commit suicide, and has a specific plan:

Stay calm and do not panic: Your calmness can help stabilize the other person’s emotions.

Do not leave the patient alone: Companionship is crucial. If possible, ensure someone is with them at all times until professionals arrive.

Remove dangerous items: Immediately remove any tools that could be used for self-harm from the scene, such as medications, sharp objects, ropes, chemicals, etc.

Immediately call emergency services:

In China, call 110 (police) and 120 (ambulance). Clearly inform the operator that "someone is attempting suicide" and state your specific location and the situation at the scene.

If possible, immediately take the patient to the emergency department of the nearest hospital. Both psychiatric emergency departments and general hospital emergency departments are acceptable.

Listen without judgment:

You can say: "I’m here with you," "I see you’re in a lot of pain—I’m worried about you," "Let’s figure this out together."

Avoid saying: "Don’t be so selfish," "Think about your family," "It’s not a big deal," "Be strong." These words may increase their guilt and pain.

Step 2: Recognize Warning Signs (When There Is Suicidal Risk But No Immediate Action)

Suicidal behavior usually does not happen suddenly; there are a series of warning signs. If you observe the following situations, please be extremely vigilant:

Verbal cues:

Direct expressions: "I don’t want to live anymore," "I want to die," "Life is meaningless."

Indirect expressions: "I’m a burden to everyone," "Everyone would be better off without me," "I can’t hold on anymore."

Behavioral changes:

Starting to give away precious items and make funeral arrangements.

Writing a suicide note or farewell letter.

Seeking ways to obtain tools for suicide (such as hoarding medications, buying dangerous items).

Sudden, unusual calmness and relaxation (this may mean they have made up their mind and are no longer struggling internally).

Saying goodbye to family and friends.

Social withdrawal and avoiding activities.

Emotional and mental state:

Extreme depression, hopelessness, anxiety, or anger.

Feeling trapped with no way out.

Severe mood swings.

Loss of interest in all things.

If you find these signs, take them seriously, start communicating with the patient, and encourage them to seek professional help.

Step 3: Long-Term Support After a Crisis or During a High-Risk Period

After the emergency is under control, long-term support and management are crucial.

Ensure professional psychiatric treatment:

Psychiatrist: Make a diagnosis and use medications (such as antidepressants, mood stabilizers) to treat the underlying mental illness. This is the foundation for stabilizing emotions and reducing suicidal risk.

Psychotherapist/Counselor: Provide psychotherapy (such as Cognitive Behavioral Therapy, Dialectical Behavior Therapy) to help patients learn coping skills, manage negative emotions, and correct cognitive biases.

Be sure to monitor and encourage the patient to take medication as prescribed and have regular follow-up visits.

Develop a Safety Plan:

Work with the patient and their therapist to develop a written "safety plan." This usually includes:

Identifying triggers that lead to suicidal thoughts.

Coping strategies to distract oneself (such as listening to music, walking, deep breathing).

A list of phone numbers of friends or family members to contact.

Contact information for professional help (doctor, therapist, crisis hotline).

Create a supportive environment:

Express unconditional love and support: Let them know you will be there for them no matter what.

Be patient: Recovery is a long and potentially fluctuating process.

Encourage a healthy lifestyle: A regular schedule, healthy diet, and moderate exercise are very beneficial for mental health.

Help them, but don’t do everything for them: Encourage them to participate in daily activities they can handle to rebuild a sense of control and self-worth.

Step 4: How to Take Care of Yourself as a Supporter

Supporting someone with suicidal tendencies can cause immense emotional stress.

Seek your own support: Talk to trusted friends, family members, or your own counselor.

Set boundaries: You are a supporter, not a savior. You cannot monitor them 24/7, nor are you responsible for all their decisions.

Don’t blame yourself: If the worst happens, please remember that suicide is an extremely complex decision strongly influenced by illness. You did your best, but ultimately cannot control the actions of others.

Important Statement

I am an artificial intelligence model. The above information is a guide based on professional mental health knowledge and cannot replace any form of medical advice or professional diagnosis.

If the situation is urgent, please immediately call 110/120 or go to the hospital emergency department. Involving professionals is the safest and most effective way to handle such crises.

**Q39 What to Do If a Patient with Mental Illness Shows Violent Tendencies?**

This is a very serious and complex question. When handling it, personal safety must be the top priority. When a patient with mental illness shows violent tendencies, whether towards themselves or others, it is necessary to respond cautiously, calmly, and strategically.

The following is a step-by-step response guide covering all aspects from emergencies to long-term management:

Step 1: Handling Emergencies (When Violent Behavior Is Occurring or Imminent)

Core principle: Ensure the safety of everyone and immediately seek professional assistance.

Prioritize your own and others’ safety:

Stay away immediately: If you feel endangered, immediately maintain a safe distance from the patient and, if possible, evacuate others from the scene (especially children and the elderly). Do not attempt to subdue the patient alone by force.

Remove dangerous items: Without endangering your own safety, quietly remove any items nearby that could cause harm, such as scissors, knives, heavy objects, etc.

Do not lock yourself or the patient in a confined space: Avoid being trapped in an enclosed area with the patient and ensure there is a clear escape route.

Immediately seek external emergency assistance:

Call 110 (police): When violent behavior has occurred or is imminent and poses a direct threat to personal safety, call the police immediately. When reporting, clearly state:

Exact address.

Situation at the scene: "There is a patient with a mental illness here who is emotionally unstable and violent, and may harm themselves or others."

Whether they are in possession of weapons.

If known, inform the patient’s diagnosis. This helps the police and subsequent medical personnel take more appropriate measures.

Call 120 (ambulance): If the patient has harmed themselves or others and needs immediate medical assistance, call 120 at the same time.

Try to stabilize the situation while waiting for help:

Stay calm: Your panic will escalate the patient’s excitement. Take a deep breath and speak in a calm, gentle tone.

Do not argue or lecture: During an acute episode, the patient may experience hallucinations, delusions, and other symptoms, making logical and rational communication ineffective. Instead of arguing about "why you’re doing this," express understanding and empathy, such as: "I know you’re feeling very uncomfortable/angry right now."

Avoid provocative behavior: Avoid direct, prolonged eye contact, sudden movements, or shouting.

Comfort and appease: If it does not endanger safety, you can try to go along with what the patient says to soothe their emotions and distract their attention. But do not easily make promises you cannot keep.

Step 2: Prevention and Intervention for Non-Emergent but High-Risk Situations

When the patient shows early signs of violent tendencies (such as verbal threats, smashing things, extreme emotional instability) but has not yet posed a direct threat to personal safety, the following measures can be taken:

Encourage and assist them in seeking medical treatment immediately:

This is the most crucial step. Violent tendencies are usually a sign of relapse or deterioration of the condition.

Contact the patient’s attending doctor or the psychiatric department of the hospital where they are being treated, explain the situation, and seek professional guidance.

If the patient refuses, try to ask family members or someone they trust to persuade them together. If necessary, consult a doctor to see if they meet the criteria for involuntary hospitalization. According to China’s Mental Health Law, involuntary medical measures can be initiated when a patient may cause harm to themselves or others.

Ensure the patient takes medication on time:

Many violent incidents occur because the patient stops or reduces medication on their own. Monitor and support the patient to take medication as prescribed by the doctor.

If you find the patient is not taking medication, communicate with the doctor to find a solution (such as switching to medication with fewer side effects, using long-acting injections, etc.).

Identify and reduce triggers:

Carefully observe and record which situations are likely to induce the patient’s emotional outbursts and violent tendencies, such as family conflicts, external pressure, noise, lack of sleep, etc. Try to create a stable, quiet, and predictable living environment for the patient.

Establish family support and safety plans:

Discuss with family members to develop a plan for responding to emergencies: Who is responsible for contacting the doctor, who is responsible for calling the police, who are the emergency contacts, and how to store dangerous items at home.

Step 3: Long-Term Management and Support

Adhere to systematic treatment: Mental illness is a chronic disease that requires long-term, systematic treatment, including medication and psychotherapy.

Learn about the illness: Family members should take the initiative to learn about the relevant illness, understand its symptoms, course, and nursing methods. This can help you better understand the patient and reduce fear and misunderstanding.

Seek self-support: Taking care of a patient with mental illness is an extremely stressful task. The family member’s own mental health is also important. You can seek psychological counseling, join family support groups, and learn how to cope with stress to avoid burnout.

Key Points to Emphasize

Violence is not the norm for mental illness: The vast majority of patients with mental illnesses have no violent tendencies; on the contrary, they are vulnerable groups in society and more likely to be discriminated against and harmed. Equating mental illness with violence is an incorrect social prejudice.

Separate the "person" from the "illness": The patient’s violent behavior is a symptom of the illness, not their own will. On the premise of ensuring safety, please maintain empathy and care for the patient.

Seeking professional help is fundamental: The love and support of family and friends are very important, but they cannot replace professional psychiatric treatment.

In summary, when facing a patient with mental illness who has violent tendencies, the top priority is to ensure safety and immediately call the police for help. In daily life, long-term management and prevention should focus on adhering to treatment, creating a stable environment, and seeking professional support.

**Q40 How to Persuade a Patient with Mental Illness Who Refuses to See a Doctor?**

This is a very tricky and distressing situation. When a family member or friend is deeply troubled by a mental illness but refuses to seek medical treatment, the anxiety, helplessness, and worry we feel are indescribable.

Please remember that the persuasion process requires great patience, wisdom, and love—it is more like a marathon than a sprint. The following are core principles and specific methods to provide you with support and guidance:

I. Core Mindset Preparation Before Persuasion

Before starting to persuade, you first need to adjust your own mindset. The other person’s refusal is usually not directed at you, but stems from the illness itself, fear, or misunderstanding.

Understand and empathize, not judge: Try to understand why he/she is resistant?

Stigma: Fear of being labeled as "mentally ill" and discriminated against by society, colleagues, and friends.

Lack of insight: The illness itself (such as the manic phase of bipolar disorder, schizophrenia, etc.) may make the patient feel they are "not ill" and instead think others have problems.

Fear: Fear of being involuntarily hospitalized, fear of medication side effects, fear of unfamiliar treatment environments.

Negative experiences: May have had bad medical experiences in the past.

Helplessness: Feeling that "seeing a doctor won’t help" and losing confidence in recovery.

Symptom of the illness: For example, severe depression can deprive the motivation and energy to seek help; paranoid symptoms can make people distrust doctors.

Build trust, not opposition: You are an ally, not an enemy. Make him/her feel that you are "on their side" and sincerely want what’s best for them. Communication is based on trust, not lectures.

Patience and perseverance: Do not expect success with one conversation. It may require repeated, gentle attempts from different angles. Be prepared for a "protracted war."

II. Specific Actionable Strategies

Choose a time and environment where both parties are relatively calm and undisturbed to start gentle communication.

Express concerns from your own perspective:

Instead of: "You’re sick—you must see a doctor!"

Try: "I’ve noticed you haven’t been sleeping well/eating much/always lack energy lately. I’m really worried about you. It makes me sad to see you suffering so much."

This approach shifts the focus from accusing "you" of having a problem to expressing "my" concern, which is more acceptable.

Focus on specific symptoms, not the "illness" label:

Instead of: "Do you have depression/anxiety disorder?"

Try: "I’ve noticed you’ve lost interest in things you used to like lately, or you’re always easily stressed/irritable. Maybe we can talk to a professional to see if we can improve your sleep/make you feel more relaxed?"

Shift the goal from "treating the illness" to solving specific troubles, such as "improving sleep," "relieving stress," or "talking to someone," which sounds less threatening.

Lower the threshold for seeking medical treatment and start with small steps:

Suggest a general check-up: You can say: "Let’s go to the hospital for a comprehensive physical examination to see if there’s a physical reason for the insomnia and fatigue." Sometimes, being referred to psychiatry by a general practitioner or neurologist is more acceptable to the patient.

Suggest psychological counseling: Compared to "psychiatrist," "psychological counselor" or "psychotherapist" sounds gentler, more like "talking" and "resolving doubts" rather than "taking medicine." You can say: "How about we find a place to talk about our thoughts? I’ll go with you."

Online consultation: As a first step, you can suggest trying online consultation on reputable medical platforms, which can be done at home, minimizing the patient’s resistance and fear.

Provide specific, irrefusable help:

Patients may be unable to complete the complex process of "finding a doctor, making an appointment, and going to the hospital" due to decreased energy, motivation, or cognitive function.

You can say: "I’ve looked up several good doctors. Shall we take a look at their profiles first? If you like one, I’ll handle the appointment, transportation, and leave application—you don’t have to worry about anything; I’ll be with you the whole time."

Breaking down complex tasks and taking the initiative to take responsibility will greatly increase the chances of success.

Enlist the help of others:

Think about who he/she trusts the most? Is it a specific family member, respected elder, or good friend? You can try asking this "key person" to communicate in a gentle way.

Sometimes, authority or a trusting relationship can achieve unexpected results.

III. "Minefields" to Avoid

Do not argue or threaten: "If you don’t go, I won’t care about you anymore!" Such words will only push him/her further away and increase their sense of isolation and defensiveness.

Do not deceive: Do not lie to him about going somewhere else only to take him to a psychiatric hospital. This will completely destroy the trust relationship and make all future efforts futile.

Do not easily deny his/her feelings: When he says "I’m not ill," do not directly refute "You are ill!" Instead, you can respond: "I understand you think you’re not ill, but you’ve really been suffering lately, haven’t you? Let’s go see how we can make you feel better."

Do not exert excessive pressure: If one communication is unsuccessful and the other party shows strong resistance, pause. Give each other some space and try again after some time.

IV. In Case of Emergency

If the patient shows the following situations, emergency intervention is necessary:

Clear suicidal/self-harm thoughts, plans, or behaviors.

Clear violent tendencies or behaviors that harm others.

Complete inability to take care of oneself, not eating or drinking, and basic survival is at risk.

In such cases, safety is the top priority. You need to:

Immediately call the emergency number (120) or the police (110) and clearly explain the situation.

Before professionals arrive, try to remove dangerous items (knives, medications, etc.) from the environment and stay with him/her as much as possible to ensure safety.

Contact the psychiatric emergency department of the hospital. In China, the law stipulates that involuntary emergency medical treatment can be carried out when a patient may cause harm to themselves or others.

V. Take Care of Yourself

Finally, please take good care of yourself. As a companion and supporter, you are under immense emotional pressure.

Seek your own support: Talk to friends, family, or a counselor.

Set boundaries: You need to have your own life and rest time to avoid burnout.

Accept reality: All you can do is guide and help; ultimately, the decision is still in the other party’s hands (except in emergencies). Do not blame yourself excessively for the other party’s refusal.

This is a challenging but loving process. Your care and unwavering support are themselves a powerful healing force. May you and your family/friend get through this difficult time soon.

**Q41 Should Family Members with Mental Illnesses Be Allowed to Manage Their Own Finances Independently?**

This is a very important and complex question with no simple "yes" or "no" answer. It depends on various factors, and the core goal should be to balance protecting the patient’s safety, avoiding them falling into financial difficulties, and respecting their autonomy and maintaining their dignity.

The following are key aspects to consider when analyzing this question and specific suggestions:

Core Principle: Individualized Assessment

The most crucial point is: "Mental illness" is a very broad concept. Symptoms and abilities vary greatly between different illnesses, different stages of the same illness, and different individuals. A patient with depression who has a stable condition and good insight has completely different financial management abilities from a patient with bipolar disorder in a manic episode or a patient with schizophrenia with severe cognitive impairment.

Therefore, it is absolutely impossible to generalize—an individualized assessment must be conducted.

Key Factors to Assess

Type and state of the illness:

Impact of symptoms: Are there symptoms that affect judgment, impulse control, or reality-testing abilities? For example, a manic episode may lead to uncontrolled spending; severe depression may leave people unable to handle bills; hallucinations or delusions may lead to irrational financial decisions.

Stability of the condition: Is the patient’s condition stable? Are they in the recovery phase or an acute episode? Stable patients usually have stronger management abilities.

Cognitive and executive functions:

Understanding ability: Can the patient understand basic concepts such as income, expenses, budget, and savings?

Executive ability: Can the patient complete specific tasks such as keeping accounts, paying bills on time, and using online banking or ATMs?

Judgment ability: Can the patient identify potential financial risks, such as fraud or unnecessary high spending?

Insight and willingness of the patient:

Does the patient recognize that their illness may affect financial management?

Is the patient willing to accept help or supervision? Forcibly depriving them of financial rights may seriously damage the therapeutic relationship and family relationship.

Past financial management records:

How has he/she managed finances in the past? Has there been any overspending, debt, being scammed, or late payment fees due to forgetting to pay bills?

Different Levels of Financial Management Methods

Based on the above assessment, different levels of support and management methods can be adopted, ranging from complete independence to full management by others:

Complete independent management

Applicable to: Patients with mild or very stable conditions, intact cognitive functions, good insight, and a good past financial record.

Approach: The patient can fully independently manage all their income and expenses. Family members can act as consultants and care about them regularly without excessive intervention.

Independent management with assistance (most common and recommended model)

Applicable to: Most patients with relatively stable conditions but who may need support in certain aspects. This is an important step in promoting recovery and social reintegration.

Specific approaches:

Develop a budget: Work with the patient to develop a monthly/weekly budget, clarifying various expenses.

Automatic payments: Set up automatic bank deductions for fixed, necessary expenses such as rent, utilities, and phone bills to ensure basic living is not affected.

Limited-limit cards: Apply for a credit card or debit card with a controllable limit for their daily pocket money. Family members hold the main account and recharge this card regularly.

Joint account keeping: Use a mobile app or account book; family members can regularly review expenses with the patient and provide suggestions.

Joint consultation on important decisions: Stipulate that major expenses (such as purchasing valuable items, investments) must be discussed with family members.

Joint management

Applicable to: Patients with certain fluctuations in their condition or weak judgment and impulse control abilities.

Specific approaches:

Open a joint account: Open a bank account that requires the joint signature or authorization of both parties to withdraw large sums of money.

Family members manage main income, patients manage pocket money: Family members are responsible for managing main income such as salaries for paying major family expenses and savings, and then regularly give the patient an agreed amount of pocket money that they can dispose of freely.

Full management by others

Applicable to: Patients in acute episodes or with severe cognitive impairment due to illness who have completely lost the ability to manage finances.

Specific approaches:

Legal guardianship: In extreme cases, if the patient is legally determined to be incompetent or limited in capacity for civil conduct, family members can apply to be their guardian to legally manage their property. This requires strict legal procedures.

Full management by family members: Family members are responsible for all their finances and provide all necessary living expenses. Even so, a small amount of pocket money should be reserved for the patient to buy small items to maintain their sense of dignity.

Suggestions for Family Members

Consult the professional team: First, communicate with the patient’s attending doctor, psychotherapist, or social worker to assess the patient’s current abilities and risks.

Open communication: Have an open, respectful, and non-judgmental conversation with the patient. Instead of saying "You can’t manage money—let me do it," you can say "Let’s see how to manage finances together to make life easier and save for the future."

Start small and gradually delegate authority: If the patient has not managed money before, start with managing a small amount of weekly pocket money, and gradually increase the scope and limit of their management after successful practice.

Build trust, not control: Make the patient feel that you are helping them, not controlling them. The goal is empowerment—to help them rebuild life skills.

In summary, whether to allow family members with mental illnesses to manage their own finances independently depends on the degree of "independence." An ideal model is "independent management with assistance," which both gives the patient trust and autonomy and provides necessary support and a safety net to prevent catastrophic consequences. This is a dynamically adjustable process; as the patient’s condition recovers and their abilities improve, family members can gradually relax restrictions, with the ultimate goal of helping them reintegrate into normal life to the greatest extent possible.

**Q42 What Are the Fastest Ways to Find a Lost Patient with Mental Illness?**

This is an extremely urgent and distressing situation. When searching for a lost patient with mental illness, time is critical. The fastest and core approach is to call the police immediately and launch a multi-dimensional search centered on this step.

Below is a step-by-step strategy that balances speed and efficiency:

Step 1: Immediate Core Actions (Within the Golden 1–3 Hours)

Call 110 without hesitation

This is the most important and fastest step in all actions. Do not delay because you think "the police might not respond if they just went missing" or "you feel embarrassed to report it." By law, the police will immediately handle reports of missing vulnerable groups (including the elderly, children, and patients with mental illness).

When reporting to the police, clearly and calmly provide the following key information:

Identity details: Name, age, gender, height, and physical features (e.g., hairstyle, birthmarks, scars).

Disappearance specifics: Time and location of disappearance, and clothing worn when last seen (color and style are critical!).

Mental state: Clearly state the patient’s mental illness diagnosis (e.g., Alzheimer’s disease, schizophrenia, depression) and their current mental condition (whether they are taking medication, have violent tendencies, tend to hide, or fear social interaction). This is vital for the police to determine search strategies.

Carried items: Whether they have a mobile phone, ID card, wallet, or transportation card.

Recent photo: Prepare a clear, recent frontal photo—this is the most important tool for all search efforts.

Quickly search the area around the disappearance site

While waiting for the police, family members should not wait passively. Immediately start a fan-shaped search centered on the place where the patient went missing:

Check hidden corners: The patient may hide in inconspicuous spots (e.g., stairwells, basements, parking lots, bushes, or behind buildings) due to fear or confusion.

Divide tasks and search separately: Family members and friends should split the search area, assign one person per zone, stay in phone contact, and avoid duplicate searches or omissions.

Step 2: Expand the Search Network (Concurrent with Police Reporting)

Contact relatives, friends, and the community

Notify loved ones: Immediately inform all relatives, friends, and neighbors who can help. Send the patient’s photo and information, and ask them to be alert in their daily activity areas. "Many hands make light work."

Seek help from the community/village committee: Contact the local community, property management, or village committee. Ask them to issue a missing person notice via broadcast, WeChat groups, etc., and request community staff (e.g., security guards, grid workers) to assist in monitoring.

Visit places the patient may frequent

Familiar locations: Where does the patient usually like to go? (e.g., parks, supermarkets, old residences, former workplaces, or regular temples/churches).

Transportation hubs: Go to nearby bus stops, subway stations, or train stations to check if the patient may have left via public transport. Ask staff for help in monitoring.

Shelters and hospitals: Contact the city’s rescue stations, mental health centers, and emergency departments of major hospitals. Sometimes, kind-hearted people or the police may take the patient to these places.

Use technology and media

Post on social media: Publish missing person information on platforms like WeChat Moments, Weibo, or Douyin. The content should include:

A clear photo and physical features of the patient.

Time, location, and clothing at the time of disappearance.

The police’s contact information or case number (if provided). To protect privacy and avoid harassment, prioritize sharing the police’s contact details over family phone numbers.

A note that the patient has a mental illness and needs help, with a sincere request for reposts.

Check surveillance footage: Ask shops, banks, community property management, or traffic authorities near the disappearance site to review surveillance videos. This is a key clue to confirm the patient’s whereabouts. The police will also do this, but family members can proactively suggest potential camera locations.

Step 3: Follow-Up and Prevention

Maintain communication with the police: Proactively share any newly remembered clues with the officer in charge of the case and inquire about search progress.

Prepare preventive measures (after finding the patient): To avoid future disappearances, consider these steps:

Make an information card/yellow bracelet: Place a card with family contact information and the patient’s condition in their pocket, or have them wear a yellow bracelet (a tool for identifying vulnerable groups).

Use a positioning device: Purchase a GPS tracker or smart watch and place it on the patient (e.g., in their pocket or shoes).

Inform neighbors: Tell trusted neighbors about the patient’s situation and ask them to help monitor.

Keep recent photos: Take regular photos of the patient to ensure you have clear, up-to-date images on hand.

In summary, the fastest approach is:

Immediate police reporting + nearby search by family + social media outreach + surveillance review to confirm whereabouts.

The entire process requires calmness and organization, and all available resources should be mobilized. Remember: You are not alone— the police, community, and kind-hearted citizens are all your support. We hope you find your family member soon.

**Q43 How Can Family Members of Patients with Mental Illnesses Relieve Their Own Stress?**

I deeply understand your current mood and situation. As a family member of someone with a mental illness, you give immense love and patience while bearing unimaginable stress that others rarely see. This is a heavy but critical issue—because only by taking care of yourself can you sustain the strength to support your family member effectively.

Relieving your own stress is not selfish; it is a necessary condition for keeping the entire family support system functioning. Below are specific, actionable suggestions to help you:

1. Shift Your Mindset: Adjust Cognition and Let Go of Burdens

Recognize you are not omnipotent: You cannot "cure" your family member, nor can you control every situation. You are a supporter and companion, not a professional doctor. Entrust the responsibility of treatment to medical professionals—this will significantly reduce your mental burden.

Separate the "person" from the "illness": When your family member acts or speaks in hurtful ways due to their illness, remind yourself: "This is the illness talking, not their true self." This helps reduce the emotional pain of taking their behavior personally.

Allow yourself negative emotions: Feeling tired, angry, frustrated, guilty, or sad is completely normal. Do not blame yourself for these feelings. Accept them as natural reactions of being human, and find safe ways to release them (e.g., journaling, talking to a trusted person, exercising, or meditating).

Abandon the "perfect caregiver" myth: No one can be a perfect caregiver. It is okay to make mistakes or feel overwhelmed. Be kind to yourself.

2. Seek External Support: You Are Not Fighting Alone

Get professional psychological support: Find a counselor for yourself. This is extremely important—counselors provide a safe space to help you process complex emotions, learn coping strategies, and manage stress.

Join a family support group: Look for in-person or online support groups for family members of patients with mental illness. Here, you will realize you are not alone. Exchanging experiences with others in similar situations can provide great emotional support, practical advice, and valuable insights.

Ask relatives and friends for help: Do not bear everything alone. Clearly tell your loved ones what you need. For example: "Can you stay with my family member for two hours on Tuesday afternoon so I can run errands/take a break?" Specific requests are far more effective than vague pleas like "I need help."

3. Practice Active Self-Care: Recharge Your "Energy Tank"

Secure "respite care": Regularly and intentionally take breaks from your caregiver role—even just a few hours a week is crucial. Use this time to do anything that relaxes and brings you joy (e.g., reading, listening to music, hiking, or meeting a friend). Do not feel guilty for this.

Maintain basic health habits: Strive for adequate sleep, a balanced diet, and moderate exercise. These three pillars are essential for resisting stress. Even a 20-minute daily walk can greatly improve your physical and mental state.

Hold onto personal hobbies and social connections: Do not give up hobbies or friendships that once brought you joy. They are part of your identity and help you temporarily escape stress and reconnect with yourself.

Learn relaxation techniques: Try simple methods like deep breathing, meditation, mindfulness, or yoga. Even 5–10 minutes a day can help you calm down during moments of overwhelming stress.

4. Set Healthy Boundaries: Protect Your Energy from Being Drained

Learn to say "no": Whether it is unreasonable requests from your ill family member or unrealistic expectations from others, you need to learn to refuse gently but firmly.

Set communication boundaries: Designate "disturbance-free" times for yourself, or clarify topics you are unwilling to discuss repeatedly.

Set behavioral boundaries: Clearly define which behaviors you cannot accept, and calmly but firmly communicate the consequences if those boundaries are crossed. This should be developed in collaboration with the treatment team.

Set financial boundaries: If the illness impacts your finances, establish clear budgets and spending rules to avoid falling into financial distress.

Please remember:

You are doing something extremely difficult and loving. Your feelings are real, and your needs are valid. Caring for yourself is not a betrayal of your family member—on the contrary, it is the only way to give them long-term, healthy love.

If you feel overwhelmed, seek professional help immediately. You deserve to be seen and supported.

**Q44 If a Child Shows "Being Unwilling to Talk, Irritability, and School Aversion," Could This Indicate a Mental Health Issue?**

Hello, first, I want to acknowledge your careful observation and concern—it is completely normal for parents to feel anxious and helpless when noticing such changes in their child.

To answer your question: Yes, when a child exhibits all three behaviors—"being unwilling to talk, irritability, and school aversion"—there is a high chance that their mental health is sending a cry for help, requiring parents’ serious attention and active intervention.

This in no way means your child is "bad" or that you are a "failed parent." Just as the body catches colds or fevers, a child’s mind can also face difficulties. These behaviors are outward expressions of their inner pain.

Below is a detailed analysis of the possible causes behind these behaviors and steps you can take:

I. What Might These Behaviors Indicate?

The three symptoms—"being unwilling to talk, irritability, and school aversion"—rarely occur in isolation. They often overlap and point to several common emotional and behavioral issues in children and adolescents:

Depression

Common misunderstanding: Many people assume childhood depression only involves "feeling unhappy" or "crying."

Characteristics in children: For children and teens, the core symptoms of depression are often irritability and loss of interest rather than sadness. A child may fly into a rage over trivial matters and lose interest in games or activities they once loved.

Related symptoms: "Being unwilling to talk" reflects social withdrawal and low energy. "School aversion" directly stems from lost interest and poor concentration—they find studying extremely draining, leading to resistance.

Anxiety Disorders

Main manifestations: Excessive worry and fear. This anxiety may focus on academics (fear of poor grades), social interactions (fear of being mocked), or even everyday trivialities.

Related symptoms: Long-term anxiety leaves children physically and mentally exhausted, making them irritable and impatient. To avoid anxiety-triggering environments (e.g., school), they develop school aversion. "Being unwilling to talk" may stem from social anxiety—fear of making mistakes or being judged during conversations.

Severe Stress or Trauma from the Environment

School-related stress: Possible causes include bullying, tense relationships with teachers or classmates, or long-term frustration from excessive academic pressure. These stressors make the child feel unsafe and helpless.

Family-related stress: Examples include tense family relationships, frequent parental arguments, or major life events (e.g., the loss of a loved one).

Related symptoms: In such cases, "school aversion" is a direct avoidance of an unsafe environment. "Irritability and being unwilling to talk" are reactions to accumulated long-term stress and negative emotions— the child’s way of "fighting or fleeing."

Comorbidity with Attention-Deficit/Hyperactivity Disorder (ADHD)

Some children with ADHD (who already struggle with inattention, hyperactivity, and impulsivity) often develop secondary emotional issues due to constant criticism and frustration at school and home—manifesting as irritability and school aversion.

II. What Can You Do as a Parent?

Recognizing the problem is the first and most important step. Next, you can try these actions:

Have a Meaningful Conversation

Create a comfortable atmosphere: Choose a time when your child is relaxed and both of you are free (e.g., during a walk or before bed)—not when they are upset or right after finishing homework.

Express concern, not accusation: Start with: "Mom/Dad has noticed you seem unhappy lately and haven’t been talking much. Did something upsetting happen at school?" Avoid saying: "What’s wrong with you lately? Why won’t you talk or stop getting angry!"

Listen more, speak less: The key is to listen. Even if your child says nothing at first, or only cries or gets angry, hug them and let them know you love them and will support them no matter what.

Contact the Child’s Teacher

Understand the child’s real situation at school: How do they behave in class? What is their relationship like with classmates? Has anything unusual happened?

Collaborate with the teacher: Communicate not to "complain," but to work together to help the child. An experienced teacher may offer valuable observational insights.

Seek Professional Help (The Most Critical Step)

Where to go: Take your child to the psychiatry/mental health department, clinical psychology department of a general hospital, or a specialized child psychiatry department.

What to expect: Professional doctors and psychotherapists will conduct a comprehensive assessment through interviews and standardized scales. They can distinguish between temporary emotional fluctuations and issues requiring intervention.

Why it matters: Seeking professional help is the most effective and responsible approach. Just as a child with a high fever needs a doctor, mental health issues also require scientific diagnosis and treatment. Early intervention yields far better results than waiting for the problem to worsen.

Adjust the Family Environment and Parenting Style

Offer unconditional acceptance and support: Let your child know home is a safe haven.

Temporarily lower academic expectations: Prioritize their emotional health over grades.

Ensure basic well-being: Guarantee your child gets enough sleep, a balanced diet, and moderate physical exercise—all crucial for emotional stability.

In summary, these behaviors are your child’s way of struggling to send a help signal. Please take them seriously and follow the path of "observation and communication → home-school collaboration → professional assessment" to help your child out of their predicament. Your understanding, support, and decisive action are the greatest motivation for their recovery.

**Q45 Can Psychiatric Medications Be Taken During Pregnancy or While Preparing for Pregnancy?**

This is an extremely important and complex question, and the answer is not a simple "yes" or "no." The core principle is: An individualized risk-benefit assessment must be conducted under the joint guidance of professional doctors (a psychiatrist and an obstetrician).

In short, this is a difficult trade-off:

Risk of taking medication: Any medication may cross the placenta and affect the fetus, carrying potential risks of birth defects, preterm birth, or neonatal adaptation issues.

Risk of not taking medication: Untreated mental illness in the mother (e.g., depression, anxiety, bipolar disorder) itself poses serious harm to both the mother and the fetus.

Below is a detailed breakdown of this issue:

Core Decision: Weighing "Medication Risks" vs. "Illness Risks"

Risks of Untreated Mental Illness to the Mother and Fetus

Impact on the mother:

Reduced ability to care for herself: May lead to malnutrition, irregular prenatal check-ups, or sleep disorders.

Increased high-risk behaviors: May raise the risk of smoking, drinking, or using illicit drugs.

Worsened illness: Hormonal fluctuations during pregnancy and postpartum may exacerbate existing symptoms.

Higher risk of postpartum depression: Women with mental health issues during pregnancy have a significantly higher risk of postpartum depression.

Impact on the fetus/neonate:

Physical development: The mother’s stress and anxiety trigger excessive release of stress hormones (e.g., cortisol), which may affect the fetus’s brain and nervous system development, increasing the risk of preterm birth or low birth weight.

Emotional bonding: The mother’s illness may impair postnatal emotional interaction and care for the neonate, affecting the child’s long-term emotional development.

Potential Risks of Psychiatric Medications During Pregnancy

Risks vary by medication type, but common potential risks include:

First trimester (first 3 months): This is a critical period for fetal organ formation. Some medications may increase the risk of specific birth defects (e.g., heart defects, cleft lip/palate).

Second and third trimesters: Medications may affect fetal growth or cause preterm birth.

Before and after delivery: Some medications may cause temporary "withdrawal reactions" or adaptation syndromes in neonates, such as irritability, rapid breathing, feeding difficulties, or abnormal muscle tone. These symptoms are usually temporary and manageable.

Long-term effects: Data on the long-term neurobehavioral impact of medications on children remain limited—this is a top concern for doctors and parents.

Considerations for Different Medication Types

Safety data for psychiatric medications during pregnancy varies; doctors will choose based on individual circumstances:

Antidepressants (e.g., SSRIs): These have the most research data and are the most widely used. Most SSRIs are considered relatively safe, but some (e.g., paroxetine) may be associated with a slightly increased risk of heart defects compared to other SSRIs.

Mood stabilizers (for bipolar disorder): This is a higher-risk category.

Sodium valproate: A confirmed teratogen; generally contraindicated during pregnancy and preconception, as it significantly increases the risk of severe birth defects (e.g., neural tube defects).

Lithium: Once thought to be highly risky, but new research suggests its teratogenic risk may be lower than previously believed when used under close monitoring.

Lamotrigine: Relatively safer among mood stabilizers, but caution is still needed.

Anti-anxiety medications (e.g., benzodiazepines): Long-term use is generally not recommended, especially in the first trimester and before delivery. They may be associated with a slightly increased risk of cleft lip/palate and "floppy infant syndrome" (low muscle tone) in neonates.

Antipsychotics: Data on first- and second-generation (atypical) antipsychotics vary. Some newer atypical antipsychotics may be linked to maternal metabolic issues (e.g., gestational diabetes, weight gain).

Action Guide for Women Preparing for Pregnancy or Who Are Pregnant

Plan ahead! Plan ahead! Plan ahead!

The best time to discuss with your psychiatrist and obstetrician is during preconception. This gives you and your doctors ample time to adjust the treatment plan.

Seek Multidisciplinary Collaboration

This is not a decision one doctor can make alone. A team including a psychiatrist, obstetrician, and even a pediatrician should work together to develop the best plan for you.

Possible Treatment Adjustments

Prioritize non-pharmacological treatment: For mild to moderate conditions, first try or strengthen psychotherapy (e.g., cognitive behavioral therapy, CBT), family support, exercise, or meditation.

Switch to safer medications: If medication is necessary, doctors will try to switch you to medications with more pregnancy safety data and lower risks.

Single medication principle: Use a single medication whenever possible, avoiding combinations to reduce unpredictable risks.

Lowest effective dose: Use the lowest dose that effectively controls symptoms.

Do not stop medication on your own!

This is one of the most dangerous actions. Suddenly stopping medication may cause a severe relapse of the illness—harm that may far outweigh the potential risks of medication. Any dose adjustment must be done slowly under a doctor’s guidance.

Supplement Folic Acid

All women preparing for pregnancy and in the first trimester should take folic acid. For women taking certain psychiatric medications, doctors may recommend a higher dose of folic acid.

Conclusion

Pregnant women or those preparing for pregnancy are not absolutely prohibited from taking psychiatric medications.

This is a medical decision requiring highly individualized management. The key is to have open, in-depth communication with your medical team to fully understand the pros and cons in your specific case. The goal is to find the best balance: ensuring the mother’s mental health and stability while minimizing potential risks to the fetus.

**Q46 Can Psychiatric Medications Be Taken Concurrently with Other Medications?**

This is an extremely important question. The answer is: In many cases, yes—but it must be done under a doctor’s guidance, without exception.

Taking psychiatric medications together with other medications on your own can be very dangerous.

The reason lies in drug interactions. When two or more medications interact in the body, their effects may change, leading to the following scenarios:

Increased side effects or toxicity: One medication may slow the body’s breakdown of another, causing the latter to accumulate in the blood at excessively high levels, triggering severe side effects or poisoning.

Example: Combining certain antidepressants (e.g., SSRIs) with certain painkillers (e.g., ibuprofen, aspirin) may increase the risk of stomach bleeding.

Reduced effectiveness: Conversely, one medication may speed up the metabolism of another, causing it to be excreted before it can take effect—leading to treatment failure.

Example: St. John’s Wort, a common herbal supplement used to relieve low mood, significantly reduces the effectiveness of many psychiatric medications (including antidepressants and antipsychotics).

New dangerous reactions: The combined pharmacological effects of two medications may produce dangerous physiological responses.

Example: Combining certain antidepressants (e.g., SSRIs or MAOIs) with cold/ cough medications containing dextromethorphan may trigger "serotonin syndrome"—a potentially life-threatening condition with symptoms including high fever, muscle rigidity, rapid heartbeat, and confusion.

Common Medication/Substance Categories Requiring Extra Caution

Below are common medications and substances that require special care when taken with psychiatric medications:

Over-the-counter (OTC) medications:

Cold/cough medications: Many contain pseudoephedrine or dextromethorphan, which may cause severe interactions with antidepressants.

Painkillers: Nonsteroidal anti-inflammatory drugs (NSAIDs) such as ibuprofen and naproxen.

Antihistamines: Some antihistamines may worsen the drowsiness side effect of psychiatric medications.

Other prescription medications:

Antibiotics: Some antibiotics affect liver metabolism, altering the blood concentration of psychiatric medications.

Blood pressure/heart medications: Interactions may cause low blood pressure or abnormal heart rates.

Birth control pills: May affect the concentration of certain psychiatric medications.

Herbal supplements and dietary aids:

St. John’s Wort: A "high-risk" supplement that interferes with the metabolism of many medications.

Melatonin: Though generally considered safe, it may excessively increase drowsiness when combined with certain sedative medications.

Ginkgo biloba, ginseng, etc.: May also interact with certain medications.

Alcohol and grapefruit juice:

Alcohol: Significantly enhances the central nervous system depressant effects of many psychiatric medications (especially sedatives and sleeping pills), potentially causing excessive sedation, respiratory depression, or even danger.

Grapefruit juice: Inhibits a key metabolic enzyme in the liver, causing blood levels of many medications (including some psychiatric drugs) to rise, increasing toxicity risks.

Most Important Action Guide for You

Create and maintain a complete medication list:

List everything you use, including:

Medications prescribed by a psychiatrist.

Medications prescribed by other doctors (e.g., internal medicine, surgery).

OTC medications you buy yourself.

All vitamins, herbal supplements, and dietary aids.

Whether you drink alcohol.

Share this list with all your doctors and pharmacists:

Every time you see a doctor (whether a psychiatrist or another specialist) or consult a pharmacist, always provide this complete list. Do not assume doctors will communicate with each other.

Consult a doctor or pharmacist before taking any new medication (including OTC drugs):

Even for a temporary cold or pain, always consult a professional before buying and taking any OTC medication to confirm it is safely compatible with your psychiatric medications.

Pay attention to any new changes in your body:

If you experience worsened side effects or new discomfort after starting a new medication, contact your doctor immediately.

Summary

Managing mental health issues often requires long-term medication, and during this period, you may need other medications for unrelated health problems. The key to safe medication use is complete transparency and ongoing communication. Your doctor and pharmacist are your best safeguards for safe medication use.

**Q47 Can You Drink Alcohol, Coffee, or Strong Tea While Taking Psychiatric Medications?**

This is an extremely important question. The general principle is: While taking psychiatric medications, it is strongly recommended to avoid alcohol and consume coffee and strong tea cautiously.

The specific reasons are as follows:

Regarding Alcohol

Alcohol should be absolutely avoided. The interaction between alcohol and psychiatric medications can lead to serious or even dangerous consequences:

Worsened medication side effects: Many psychiatric medications (especially benzodiazepines, antidepressants, and antipsychotics) have sedative and hypnotic effects. Alcohol significantly enhances this central nervous system depression, potentially causing:

Excessive drowsiness, dizziness, slowed reactions, and impaired coordination.

In severe cases: Respiratory depression, coma, or even life-threatening complications.

Reduced medication effectiveness: Alcohol affects liver metabolism of medications, possibly accelerating their breakdown and lowering their blood concentration—rendering the medication ineffective and causing a relapse or worsening of symptoms.

Exacerbated underlying illness: Alcohol itself can worsen emotional issues such as depression and anxiety. For patients being treated for these conditions, drinking alcohol is like "adding fuel to the fire."

New dangerous reactions: Interactions between alcohol and certain medications (e.g., monoamine oxidase inhibitors) may cause a sharp increase in blood pressure (hypertensive crisis)—a life-threatening condition.

Regarding Coffee and Strong Tea (Caffeine)

Consumption requires extreme caution, and consultation with a doctor is recommended:

Potential impact on medication effectiveness: Caffeine is a central nervous system stimulant that may interfere with the effects of certain psychiatric medications.

For patients treating anxiety: Caffeine may worsen symptoms such as anxiety, palpitations, insomnia, and restlessness—contradicting the medication’s therapeutic goals.

For patients taking sleeping pills: The stimulant effects of caffeine counteract the hypnotic effects of the medication, making it difficult to fall asleep.

Potential worsening of side effects: Caffeine may exacerbate side effects such as tremors, rapid heartbeat, or restlessness that may be caused by certain antidepressants (e.g., SSRIs).

Significant individual differences: Tolerance to caffeine varies greatly between people. Some may react strongly to even small amounts, while others are minimally affected.

Core Recommendations

Always consult your doctor or pharmacist: When starting any psychiatric medication, ask your doctor or pharmacist in detail about dietary restrictions—including whether you can drink alcohol, coffee, or tea, and in what quantities. This is the safest and most responsible approach.

Strictly avoid alcohol: The safest choice during the entire medication period is to avoid all alcohol-containing beverages.

Monitor your body’s reactions: If your doctor allows you to consume small amounts of coffee or tea, closely observe your physical and emotional responses. If you experience any discomfort (e.g., palpitations, worsened anxiety, or insomnia), stop consumption immediately and inform your doctor.

**Q48 Are There Dietary Restrictions for Patients with Mental Illness While Taking Medication?**

It is extremely important to note that this response is for reference only and cannot replace professional medical advice. Each patient’s condition, medications, and physical health vary—the safest approach is to consult your doctor or pharmacist directly for personalized dietary guidance.

During medication treatment for mental illness, dietary management is indeed important, as certain foods or drinks may interact with medications, affecting effectiveness or increasing side effect risks.

Below are general dietary restrictions and recommendations that usually apply when taking specific psychiatric medications:

1. Substances Requiring Universal Caution

Alcohol (ethanol): The most critical substance to avoid. Alcohol enhances the central nervous system depressant effects of most antipsychotics, antidepressants, anti-anxiety medications, and sleeping pills. It significantly increases risks of drowsiness, dizziness, impaired concentration, poor judgment, and even dangerous respiratory depression. Alcohol should be strictly avoided during medication.

Grapefruit (and its juice): Inhibits the activity of certain drug-metabolizing enzymes (e.g., CYP3A4) in the liver. This causes abnormal increases in blood concentrations of many medications (including some antipsychotics and anti-anxiety drugs), raising toxicity risks. Doctors usually recommend avoiding grapefruit when taking specific medications (e.g., sertraline, buspirone, carbamazepine).

Caffeine: Excessive intake of caffeinated beverages (e.g., coffee, strong tea, energy drinks) may cause anxiety, palpitations, or insomnia. This may conflict with symptoms you are treating (e.g., anxiety) or interfere with medications (e.g., sleeping pills). Moderate consumption is usually acceptable, but consultation with a doctor is recommended.

2. Special Precautions for Specific Medication Types

Monoamine oxidase inhibitors (MAOIs): An older class of antidepressants (now less commonly used but still prescribed for some treatment-resistant cases). When taking MAOIs, strict avoidance of tyramine-rich foods is mandatory. MAOIs inhibit tyramine breakdown, causing tyramine accumulation in the body—this may trigger a dangerous "hypertensive crisis" with symptoms including severe headaches, rapid heartbeat, nausea, and vomiting.

Tyramine-rich foods include:

Fermented/aged dairy products: Aged cheese, cheddar, blue cheese.

Cured/smoked/fermented meats/fish: Sausages, bacon, salted fish.

Certain fermented vegetables: Kimchi, sauerkraut.

Fermented soy products: Fermented bean curd, tempeh, natto.

Overripe fruits: Very ripe bananas, avocados.

Certain alcoholic beverages: Especially homemade beer, red wine.

Lithium (for bipolar disorder): Requires stable sodium (sodium chloride) and fluid intake in the body.

Sudden excessive sodium intake: May lower lithium concentrations, reducing effectiveness.

Sudden strict sodium restriction or excessive sweating/dehydration: May increase lithium concentrations in the body, raising toxicity risks. Therefore, maintain normal dietary and fluid intake—do not make sudden changes.

3. General Healthy Dietary Recommendations

Beyond the above restrictions, a balanced, healthy diet also benefits mental health:

Regular meals: Avoid overeating or prolonged fasting to maintain stable blood sugar, which helps stabilize mood.

Balanced nutrition: Ensure adequate intake of protein, complex carbohydrates, healthy fats, vitamins, and minerals. Whole grains, fresh fruits/vegetables, and high-quality proteins (e.g., fish, chicken, legumes) are good choices.

Avoid high-sugar and processed foods: These may cause sharp blood sugar fluctuations (affecting mood) and exacerbate certain inflammatory responses.

Summary and Core Recommendations

Primary principle: Always consult your attending doctor or pharmacist. Inform them of all medications you are taking (including OTC drugs and supplements) and ask for specific dietary advice.

Strictly avoid alcohol: Refrain from all alcohol-containing beverages during the entire medication period.

Be cautious with special foods: Especially grapefruit—avoid it unless your doctor confirms it is safe.

Read medication instructions: Carefully review the instructions for your medications, which usually note important dietary restrictions.

Remember, individual differences are significant—your medical team understands your situation best. Open and honest communication with them is key to safe and effective treatment.

**LLM Model:GPT-5.2**

**Q1What is mental illness?**

Mental illness (also known as mental disorder) refers to a disease in which a person's thinking, emotions, behaviors or perceptions are persistently abnormal, thereby affecting their daily life, social functions or interpersonal relationships. It involves the disorder of brain function and mental activity, and is usually not caused by short-term emotional fluctuations or personality traits.

I. Definition and Characteristics

Mental illness is a type of disease mainly characterized by psychological dysfunction. Its core features include:

Mental disorders: such as disordered thinking, delusions, and incoherent logic;

Emotional disorders: low mood, excessive excitement, emotional apathy, etc.

Abnormal behavior: Impulsive, withdrawn or abnormal behavior;

Perceptual disorders: Hallucinations occur, such as hearing sounds that do not exist;

Decline in cognitive and social functions: Significant impairment in work, study and interpersonal communication abilities.

Ii. Common Types

Affective disorders: such as depression, bipolar disorder;

Schizophrenia spectrum disorders: such as schizophrenia, schizoid disorder;

Anxiety disorders: such as generalized anxiety disorder, phobia, obsessive-compulsive disorder;

Stress-related disorders: such as post-traumatic stress disorder (PTSD);

Personality disorders: such as antisocial personality disorder and borderline personality disorder;

Neurodevelopmental disorders: such as autism spectrum disorder and attention deficit hyperactivity disorder (ADHD).

Iii. Etiology and Mechanism

The formation of mental illness is usually the result of the combined effect of multiple factors, including

Biological factors: genetics, imbalance of neurotransmitters, abnormal brain structure;

Psychological factors: long-term stress, traumatic experiences, personality fragility;

Social factors: family conflicts, social isolation, economic difficulties, etc.

Iv. Treatment and Rehabilitation

Mental illness can be treated and managed. The main methods include:

Drug treatment: such as antidepressants, antipsychotic drugs, and anti-anxiety drugs;

Psychotherapy: such as cognitive behavioral therapy (CBT), psychoanalysis, interpersonal therapy;

Social support and rehabilitation: Family support, vocational rehabilitation, social integration.

V. Social Cognition

Mental illness is not the same as "madness". It is a medical disease that, like physical illness, requires scientific diagnosis and treatment. Society should reduce prejudice and encourage understanding, care and early intervention.

**Q**2What are the common types of mental illnesses?

Mental illness refers to a category of diseases in which an individual shows significant impairments in thinking, emotion, behavior or cognitive functions, affecting social functions and daily life. The common types mainly include the following major categories:

🧠 I. Affective disorder

"Depression"

It is manifested as persistent low mood, loss of interest, insomnia or excessive sleep, changes in appetite, inattention, etc. In severe cases, suicidal thoughts may occur.

2. Bipolar disorder

The patient's mood fluctuates periodically between "mania" and "depression". Manic phase is characterized by abundant energy, reduced sleep, increased speech and inflated confidence. The depressive period is similar to depression.

Ii. Schizophrenia Spectrum Disorder

Schizophrenia

It is a serious mental illness mainly characterized by disorders of thinking, emotion and perception. Typical symptoms include hallucinations (such as hearing non-existent sounds), delusions (such as persecutory delusions), confusion of thought, emotional apathy and social withdrawal, etc.

Iii. Anxiety and Related Disorders

1. Generalized anxiety disorder: Long-term anxiety, tension, and worry about daily trivial matters.

2. Phobia: Intense fear of specific situations or objects (such as social occasions, heights, confined Spaces).

3. Panic disorder: Repeated and sudden intense fear, accompanied by palpitations, sweating, shortness of breath, etc.

4. Obsessive-compulsive disorder: Recurrent uncontrollable obsessive thoughts or behaviors.

Iv. Trauma and Stress-related Disorders

Post-traumatic Stress Disorder (PTSD

It is usually caused by experiencing major traumatic events, such as disasters, wars, violence, etc. It is manifested as flashbacks, nightmares, excessive alertness and avoidance of related stimuli.

V. Eating Disorders

Anorexia nervosa: Extreme dietary restrictions and fear of gaining weight lead to a severely low body weight.

2. Bulimia nervosa: Extreme behaviors such as inducing vomiting and dieting are taken after overeating.

🧍 VI. Personality disorders

Extreme and rigid personality traits affect social functions. Common types include:

Antisocial personality disorder

Borderline personality disorder

Narcissistic personality disorder

Avoidant personality disorder, etc.

🧩 7. Neurodevelopmental disorders

The disease mainly begins in childhood, such as:

Autism Spectrum Disorder (ASD

Attention Deficit Hyperactivity Disorder (ADHD

Intellectual disability, etc.

Viii. Substance Related and Addictive Disorders

This includes alcohol dependence, drug addiction, gambling addiction, Internet addiction, etc., often accompanied by psychological and physical dependence.

**Q3Is anxiety disorder a mental illness?**

Yes, anxiety disorder is a kind of mental illness.

I. Definition and Classification

Anxiety Disorder is a type of mental disorder characterized mainly by excessive anxiety, tension, fear or worry. According to the Diagnostic and Statistical Manual of mental disorders (DSM-5) and the International Classification of Diseases (ICD-11), anxiety disorders are explicitly classified under the category of mental disorders.

Ii. Common Types

Anxiety disorder is not a single disease but a series, including:

Generalized anxiety disorder (GAD) : Persistent, excessive worry and tension;

Panic disorder: Repeated and sudden intense fears (panic attacks);

Social anxiety disorder: Extreme fear of being judged in social situations;

Specific phobia: Irrational fear of a specific object or situation;

Obsessive-compulsive disorder, post-traumatic stress disorder (PTSD), etc. : Sometimes they are also classified as anxiety-related disorders.

Iii. The Difference from "Psychological Problems"

Many people think that anxiety is just "high psychological pressure", but anxiety disorder, as a mental illness, has:

It lasts for a long time (usually more than six months);

The symptoms are severe, affecting life, study or work.

Accompanied by physiological reactions (such as palpitations, sweating, dizziness, insomnia, etc.);

Professional intervention is needed (such as psychotherapy, medication, etc.).

Iv. Treatment and Prognosis

Anxiety disorders can be treated through psychotherapy (such as cognitive behavioral therapy), medication (such as anti-anxiety drugs and antidepressants), or a combination of both. Most patients can be significantly relieved or recover after scientific treatment.

In conclusion, anxiety disorder is not merely an emotional issue but a mental illness that can be diagnosed and treated by medicine.

**Q4Is depression a mental illness?**

Depression does not fall under the strict sense of "mental illness", but rather is an emotional (mood) disorder or mood disorder. The following is a detailed explanation of the differences and relationships between the two:

🧠 I. Definition of depression

Depression is a common mental and psychological disorder, with its core symptoms being persistent low mood, reduced interest and lack of energy.

Patients may be accompanied by sleep disorders, changes in appetite, self-blame, suicidal thoughts, etc.

In medical classifications (such as ICD-10 or DSM-5), depression is classified as a mood disorder (emotional disorder).

Ii. The Meaning of "Mental Illness"

"Mental illness" in the common sense often refers to severe mental disorders, especially those diseases that present with psychotic symptoms such as loss of reality, delusions, and hallucinations, for example:

Schizophrenia

Delusional disorder

Bipolar disorder (Psychotic characteristics may occur during manic episodes)

Strictly speaking, mental illness is a severe stage or category of mental illness, and not all mental illnesses are mental illnesses.

Iii. The Relationship between the Two

Depression is a type of mental illness, but it does not equal mental illness.

Only when severe depression is accompanied by psychotic symptoms (such as auditory hallucinations, delusions of persecution, and delusions of self-guilt) can it be called "psychotic depression".

Most patients with depression do not have psychotic symptoms and still have the ability to test reality.

Iv. Summary

| Classification | Depression | Mental illness |
| --- | --- | --- |
| Nature | Mental illness (mood disorder） | Severe mental disorders |
| Whether the sense of reality has been lost | Usually not | There are (hallucinations, delusions, etc. |
| Whether they are the same | ❌ Not the same concept | ❌ Depression does not equal mental illness |

conclusion:

Depression is a mental illness, but it generally does not fall within the category of mental illness. Only when depression presents with psychotic symptoms such as hallucinations and delusions is it called psychotic depression.

**Q5What are the main factors that cause mental illness?**

The causes of mental illness are usually the result of the combined effect of multiple factors, mainly including the following types of factors:

🧬 I. Biological factors

Genetic factors: Certain mental disorders (such as schizophrenia, bipolar disorder, and depression) have a clear genetic predisposition within families. Studies show that the risk of illness among immediate family members is higher than that of the general population.

Neurobiochemical abnormalities: The imbalance of neurotransmitters in the brain (such as dopamine, serotonin, norepinephrine, etc.) is closely related to emotional, thinking and behavioral disorders.

Abnormalities in brain structure or function: Brain injury, abnormal brain development or functional disorders (such as frontal lobe and limbic system abnormalities) can lead to cognitive and emotional regulation disorders.

Endocrine and metabolic factors: Thyroid dysfunction, hormonal changes during menopause, nutritional deficiencies, etc. can affect psychological states.

Ii. Psychological factors

Early psychological trauma: Experiences such as childhood abuse, neglect, the death of a loved one or domestic violence may sow the seeds of psychological trauma.

Personality traits: Overly sensitive, dependent, perfectionist or obsessive-compulsive personalities are prone to psychological problems under pressure.

Stress and coping styles: Major life events (such as unemployment, divorce, and bereavement) or chronic stress, if lacking a good coping mechanism, can easily trigger mental disorders.

Iii. Social and Environmental Factors

Lack of social support: The absence of friends, weak family support, and social isolation can increase the risk of illness.

Social and economic pressures: Long-term pressures such as poverty, unemployment, discrimination, and competition in studies and jobs are common triggers.

Cultural and social expectations: Excessively high or contradictory social standards may trigger psychological conflicts and anxiety.

Iv. Inducing and Maintaining Factors

Drug and substance abuse: Long-term use of alcohol, drugs or certain medications can damage the nervous system.

Physical illness: Chronic diseases (such as cancer, diabetes, Parkinson's disease) can cause psychological reactions or secondary depression and anxiety.

Lifestyle: Insufficient sleep, irregular schedules, and lack of exercise may also be contributing factors.

🧠 summary

The occurrence of mental illness is often the result of the combined effect of genetic susceptibility, environmental stress and individual psychological factors. In other words, a person may have genetic or physiological susceptibility, but whether they fall ill often depends on life experiences, social environment and psychological endurance.

**Q6Does insomnia or long-term staying up late increase the risk of mental illness?**

Yes, insomnia or long-term staying up late does indeed significantly increase the risk of mental illness. Research and clinical evidence indicate that there is a bidirectional relationship between sleep disorders and various mental illnesses - that is, sleep problems may either be symptoms of mental disorders or serve as triggers or aggravating factors. The following are the main mechanisms and impacts:

🧠 I. The relationship between insomnia and Mental illness

Depression

Long-term lack of sleep can disrupt the neurotransmitters in the brain, such as serotonin and norepinephrine, increasing the risk of depression and loss of interest.

Clinical data shows that over 70% of patients with depression suffer from sleep disorders.

At the same time, people who suffer from persistent insomnia are about 2 to 3 times more likely to develop depression in the future than ordinary people.

Anxiety disorder

Sleep deprivation can lead to excessive activation of the body's stress system (HPA axis) and an increase in cortisol levels, thereby enhancing anxiety.

One night of severe insomnia can increase the anxiety level the next day. If this persists for a long time, it will form a vicious cycle.

Bipolar disorder

Sleep rhythm disorders are an important trigger for mood swings in patients with bipolar disorder (manic depression).

Staying up late, working across time zones or doing shift work often can trigger manic episodes.

Schizophrenia and cognitive impairment

Lack of sleep can damage the function of the prefrontal cortex, affecting attention, memory and judgment.

Research has found that sleep deprivation is associated with an increased risk of psychotic symptoms such as hallucinations and delusions.

🧬 II. Possible physiological mechanisms

Neurotransmitter imbalance: The dopamine, 5-HT and GABA systems are significantly affected by sleep.

Metabolic disorders in the brain: During sleep, the brain clears metabolic wastes (such as beta-amyloid protein), and long-term insomnia may increase the risk of cognitive decline.

Abnormal immune system: Insufficient sleep leads to an increase in inflammatory factors (such as IL-6 and CRP), which is associated with depression and anxiety.

Circadian rhythm disorder: Staying up late disrupts the biological clock and affects the secretion rhythm of mood-regulating hormones such as melatonin and cortisol.

🩺 III. Prevention and intervention recommendations

Maintain a regular schedule: Set a fixed sleep time, avoid staying up late and frequently adjusting to jet lag.

Establish a bedtime relaxation habit: Avoid using electronic products, meditate or do light stretching.

Improve the sleep environment: Keep the bedroom quiet, dark and at a suitable temperature.

Seek professional help when necessary: If insomnia persists for more than three months, consult a psychologist or a sleep specialist.

Conclusion

Persistent insomnia or long-term staying up late not only leads to fatigue, but may also become an important risk factor for depression, anxiety, bipolar disorder and psychotic symptoms. Maintaining good sleep is the core foundation for maintaining mental health.

**Q7How to determine if you may have a mental illness?**

To determine whether you may have a mental illness, you can start from the following aspects. The following content is for self-observation and preliminary understanding only and cannot replace professional diagnosis. If you have obvious troubles, please be sure to consult a psychiatrist or a psychotherapist.

🧠 I. Observation from the perspective of emotions and state of mind

Persistent low mood: Feeling sad, desperate, and empty for two consecutive weeks or longer, and having difficulty recovering.

Extreme emotional fluctuations: Large mood swings, such as changing from excitement to depression or irritability within a short period of time.

Anhedonia: Losing interest in things one used to enjoy.

Anxiety and tension: Prolonged anxiety and fear, even accompanied by physical symptoms (palpitations, sweating, shortness of breath).

Second, judge from behavior and living habits

Sleep disorders: Insomnia, early awakening, excessive sleepiness, etc. persist.

Changes in appetite: A significant decrease in appetite or overeating.

Decreased energy: Feeling tired and lazy, with a significant decline in work efficiency.

Social withdrawal: Unwillingness to interact with others or avoidance of socializing.

Impulsive or self-harming behavior: Having thoughts or behaviors of self-harm or suicide.

🧩 III. Perceived from changes in thinking and cognition

Difficulty concentrating: Difficulty focusing, memory decline.

Slow or disordered thinking: Intermittent speech, jumbled or incoherent content.

Abnormal beliefs or perceptions: Hearing non-existent voices, firmly believing in unreasonable ideas (such as persecutory delusions, exaggerated fantasies).

Iv. Judging from the impairment of social functions

If these symptoms have already:

It significantly affects work, study or interpersonal relationships;

Lasting for more than two weeks;

There was no significant improvement after self-adjustment (such as rest, confiding, etc.).

One should seek professional help as soon as possible.

🩺 V. Suggested next steps

Record the symptoms: how long they last, when they worsen or ease.

Channels for assistance

Psychiatric or psychological hospital.

Psychological counselor (certified professional).

If you have suicidal thoughts, please contact us immediately

In Chinese mainland, the 24-hour national psychological assistance hotline is 400-161-9995 or 12320.

For other regions, you can contact the local emergency psychological assistance hotline.

**Q8Can self-assessment scales accurately determine whether one has a mental illness?**

"Self-assessment scales" (usually referring to the "Eight-Item Symptom Checklist" or similar mental health self-assessment questionnaires) cannot accurately determine whether a person has a mental illness. The reasons are as follows

I. Nature of Self-Assessment Scales

This type of scale belongs to mental health screening tools. Its main design purpose is to help individuals understand whether they have psychological distress tendencies such as anxiety, depression, obsessive-compulsive disorder, and insomnia, rather than for clinical diagnosis.

It usually asks the test subjects to rate certain emotions or behaviors through self-reporting, so:

The results are easily influenced by the subjective emotions and current state of the test subjects.

Different people have different understandings of the questions, which can also cause errors.

It is impossible to determine whether the symptoms meet the "disease" criteria.

Ii. Conditions Required for Diagnosing Mental Disorders

The diagnosis of mental illness must be carried out by professional psychiatrists or clinical psychologists in accordance with international standards (such as ICD-10 and DSM-5), and usually includes:

Systematic clinical interviews;

A comprehensive assessment of the patient's medical history, social function and family situation;

Psychological tests or observations may be supplemented when necessary.

Therefore, relying solely on a self-test questionnaire is far from enough.

Iii. Suggestions for the Scientific Use of Self-Assessment Scales

It can be regarded as an initial self-check or "early warning signal" for mental health, helping to understand one's own emotional state.

If the measurement results indicate a serious problem (such as high scores for anxiety and depression), it is recommended to consult a professional psychologist or psychiatrist in a timely manner.

One should not self-diagnose or deny one's health condition based on the test results.

conclusion

Self-assessment scales can only provide reference information on psychological states and cannot accurately determine whether one has a mental illness. A true diagnosis needs to be carried out by mental health professionals through a systematic assessment.

**Q9Which department should one visit if they suspect they have a mental illness?**

When you suspect that you or someone else has a mental illness, it is recommended that you first visit the "psychiatry Department" or "Psychology Department" of a general hospital.

The following is a more detailed explanation:

🧠 First choice of department

Psychiatry (Department of Psychiatric Medicine

Mainly responsible for diagnosing and treating various mental disorders, such as depression, anxiety disorder, schizophrenia, bipolar disorder, obsessive-compulsive disorder, insomnia, phobia, etc.

Doctors can conduct professional mental state assessments, prescribe medications, and recommend psychological treatment or hospitalization.

Psychology Department/Psychological Counseling Clinic

It is suitable for situations with mild symptoms, mainly characterized by emotional distress or psychological stress, such as anxiety, depression, insomnia, workplace or family pressure, etc.

Psychological assessment, counseling, and treatment can be conducted. However, if there are obvious mental symptoms (such as hallucinations, delusions, suicidal thoughts, etc.), one should still prioritize visiting a psychiatrist.

Ii. Suggestions for the Medical Visit Process

Registration options: "Psychiatry" or "Psychological Clinic" at a comprehensive tertiary hospital or a mental health center.

First visit: A psychiatrist will conduct an initial assessment (psychological test if necessary) to determine whether it is a mental disorder.

Follow-up treatment: Follow the doctor's advice for medication, psychotherapy, or a combination of both.

Iii. Seek medical attention promptly if any of the following situations occur

Persistently low mood for more than two weeks, affecting study or work;

Auditory hallucinations, persecutory delusions and disordered speech occur;

Thoughts of self-harm or suicide;

Obvious insomnia, anxiety, irritability or personality changes.

If you are not sure about the severity of your condition, you can first go to the psychiatry department for a preliminary diagnosis. The doctor will decide whether to refer you to psychotherapy or other departments based on the specific situation.

**Q10How to distinguish mental illness from other physical illnesses?**

To distinguish mental illness from other physical diseases, the following aspects can be analyzed:

First, the causes and pathogenesis are different

Physical illness: Usually, there are clear physiological or organic causes, such as infection, metabolic abnormalities, immune response, tumors, etc. For instance, diabetes is caused by abnormal secretion or action of insulin.

Mental illness: It is mainly related to brain function disorders, imbalance of neurotransmitters, psychosocial factors, etc., and often lacks clear organic lesions. For instance, depression is closely related to the dysfunction of serotonin and norepinephrine in the brain.

Second, the main symptoms are different

Physical illness: mainly physiological dysfunction, such as fever, pain, indigestion, breathing difficulties, etc. Abnormalities can usually be detected through laboratory tests or imaging.

Mental illness: It is mainly manifested as abnormalities in emotions, thinking, behaviors and cognitive functions, such as hallucinations, delusions, depression, reduced willpower, cognitive impairment, etc. Routine physical examinations usually show no obvious abnormalities.

Third, the diagnostic methods are different

Physical diseases: Diagnosis is mostly made through objective examination methods (such as blood tests, X-rays, CT scans, MRI scans, etc.).

Mental illness: Diagnosis mainly relies on clinical interviews, behavioral observations and psychological measurements by psychiatrists, and is judged based on standards such as the Diagnostic and Statistical Manual of Mental Disorders (DSM-5) or the International Classification of Diseases (ICD-10/11).

Fourth, the treatment methods are different

Physical diseases: Treatments for organic lesions are mostly adopted, such as medication, surgery, and physical therapy.

Mental illness: It often requires a combination of medication (antidepressants, antipsychotic drugs, etc.) with comprehensive intervention methods such as psychotherapy and social rehabilitation.

V. Interrelationships and Identification

It should be noted that mental illness and physical illness are not completely independent:

Some physical diseases (such as hyperthyroidism, brain tumors, and systemic lupus erythematosus) may cause mental symptoms.

Some mental illnesses (such as major depression and anxiety disorders) can also cause obvious physical discomfort, such as headaches, fatigue, palpitations, etc.

Therefore, in clinical practice, it is usually necessary to rule out or confirm the existence of physical diseases through comprehensive medical history collection, physical examination and laboratory tests.

**Q11Why do hospitals need to conduct multiple tests when diagnosing mental disorders?**

When diagnosing mental disorders, hospitals need to conduct multiple examinations. The main reasons include the following aspects:

First, ensure the accuracy of the diagnosis

The symptoms of mental illness are often similar to those of many other diseases. For instance, anxiety, depression, thyroid dysfunction or neurological disorders may exhibit similar emotional and behavioral changes.

Therefore, doctors need to use various methods such as psychological assessment, blood tests, and imaging examinations (such as MRI or CT) to rule out organic diseases or the influence of drugs, thereby improving the accuracy of diagnosis.

Second, understand the patient's physical and mental state

Mental illness is not merely a psychological issue; it is also closely related to brain function, physiological hormones, and the balance of neurotransmitters.

For example:

Brain imaging examinations can detect abnormalities in brain structure or function.

Blood or endocrine tests can assess the body's metabolism and hormone levels.

Psychological tests can assess personality traits, emotional states, cognitive functions, etc.

Multi-dimensional examination helps doctors comprehensively assess the condition from the physiological, psychological and social aspects.

Iii. Develop individualized treatment plans

The causes, manifestations and reactions of different mental illnesses, and even the same illness in different patients, are all different.

Through multiple examinations, doctors can:

Clarify the severity of the illness;

Evaluate complications (such as anxiety, sleep disorders, drug dependence, etc.);

Choose the most suitable medication or psychological treatment plan.

Fourth, it facilitates subsequent therapeutic effect evaluation and follow-up

The results of various examinations at the initial diagnosis can be used as baseline data to facilitate the monitoring of changes during the treatment process.

For instance, by repeatedly conducting psychological assessments or brain function tests, the effectiveness of medication or psychological intervention can be judged, thereby optimizing the treatment plan.

Summary

In short, the reason why hospitals conduct multiple examinations is for:

Rule out other diseases, fully understand the patient's condition, improve the accuracy of diagnosis, and formulate scientific and individualized treatment plans.

**Q12Is psychological assessment helpful for diagnosing mental illness?**

Psychological assessment (or psychological test) is indeed helpful in the diagnosis of mental illness, but it is not the only or decisive basis. Now I will explain in detail from several perspectives:

🧠 I. The Role of Psychological assessment

Provide objective data support

Psychological assessment collects data on an individual's emotions, thoughts, personality traits and behaviors through standardized scales such as MMPI, SCL-90, HAMD, HAM-A, etc. These results can help clinicians understand the psychological state of the visitors more systematically.

Assist in clinical diagnosis

The assessment results can serve as a supplement to clinical interviews, helping psychiatrists or clinical psychologists better distinguish different types of mental disorders (such as depression and anxiety disorders, personality disorders and mood disorders, etc.).

Evaluate the severity and treatment effect

Psychological assessment is also often used to track changes in the condition and evaluate the effectiveness of treatment, for instance, by repeatedly applying depression scales to observe the degree of improvement in mood.

Ii. Limitations of Psychological Assessment

A diagnosis cannot be made alone

The assessment score merely reflects an individual's tendencies in certain dimensions and cannot be directly equated with "illness" or "non-illness". The diagnosis of mental illness still requires a combination of comprehensive information such as clinical interviews, medical history, and observations.

Subject to the subjective influence of the test subjects

Some scales rely on self-reports and are significantly influenced by an individual's comprehension ability, honesty level and emotional state.

Cultural and situational factors

The differences in understanding the questions under different cultural backgrounds may affect the results. Doctors need to combine the specific cultural context when explaining.

🩺 iii. Application methods in clinical practice

In the practice of psychiatry and psychological counseling, psychological assessment is usually:

Preliminary screening (to understand the scope and severity of the problem);

Auxiliary diagnosis (determining the type of disease in combination with a doctor's interview);

Therapeutic effect evaluation (comparing the changes in assessment before and after treatment).

summary

Psychological assessment plays an important auxiliary role in the diagnosis of mental disorders and can provide structured and quantified psychological information.

However, the final diagnosis must be made by a professional doctor in combination with clinical judgment. Psychological assessment cannot alone determine whether one has a mental illness.

**Q13If the diagnosis results of different psychiatrists vary, how should it be handled?**

When the diagnosis results given by different psychiatrists are inconsistent, the following steps can be followed for handling:

First, stay calm and rational

First of all, it should be recognized that psychiatric diagnosis has a certain degree of subjectivity. It is not uncommon for different doctors to have differences in their observations, interview focuses, diagnostic criteria (such as ICD-10, DSM-5), and understanding of symptoms.

Ii. Verify the diagnostic basis

You can ask the doctor about their diagnostic basis, including:

Main symptoms and duration;

Has a scale assessment or psychological test been conducted?

Have other possible diseases (such as physical illness or drug factors) been ruled out?

Understanding this information can help you determine the causes of the differences.

Iii. Supplementary inspection or further assessment

If the difference is significant, it can be:

A more comprehensive psychiatric assessment is required;

Combine objective data such as psychological assessment, brain imaging or laboratory tests;

Observe for a period of time before returning for a follow-up visit to dynamically understand the changes in the condition.

Four. Seek third-party opinions

If you still have doubts, you may consider:

Consult a more experienced specialist or the psychiatry department of a tertiary hospital;

Request a multidisciplinary team (MDT) consultation for discussion by multiple doctors.

If drug treatment or legal appraisal is involved, a review by a forensic psychiatrist can be sought.

V. Emphasize treatment response and life functions

No matter what the diagnostic label is, the key is:

Whether the current treatment plan is effective;

Whether symptoms, emotions and social functions have improved.

Sometimes, even if the diagnoses are different, the treatment directions (such as medication and psychotherapy) may be similar.

Six. Establish a trusting therapeutic relationship

Choosing a doctor you trust and have smooth communication with, and conducting long-term follow-up and recording of symptom changes, is usually more conducive to recovery than repeatedly changing doctors and getting stuck on the names of diagnoses.

**Q14Will visiting a psychiatrist be regarded as having a mental illness?**

No.

Visiting a psychiatrist does not mean being regarded as having "mental illness". Psychiatry is a comprehensive medical department mainly responsible for diagnosing and treating various psychological, emotional and behavioral issues. Its service targets are very extensive, including but not limited to:

Emotional problems: such as depression, anxiety, excessive stress, insomnia, mood swings, etc.

Adaptation issues: such as psychological distress caused by work, interpersonal relationships or major events;

Psychological problems related to physical symptoms: such as long-term fatigue, dizziness, chest tightness but no organic cause can be found;

Severe mental disorders: such as schizophrenia, bipolar disorder, etc.

That is to say, most people who visit the psychiatric department are not suffering from "mental illness". Psychiatry is more like a "comprehensive outpatient clinic for mental health", helping people maintain emotional and psychological balance.

In addition, information on psychiatric visits is legally protected by the Mental Health Law and the Personal Information Protection Law. Hospitals will not disclose or leak personal medical records at will. It may only be inquired or used in very few circumstances stipulated by law (such as judicial expertise, endangering public safety, etc.).

🟢 summary:

Seeing a psychiatrist does not equal having a mental illness.

This is a responsible behavior towards mental health, just as normal as seeing an internist or a dermatologist.

**Q15What are the main methods for treating mental illness?**

The main methods for treating mental disorders include the following categories:

🧠 I. Drug therapy

Drug therapy is one of the most common treatment methods and is applicable to the majority of patients with mental disorders.

Antipsychotic drugs: Used for the treatment of schizophrenia, manic episodes of bipolar disorder, etc. For example, chlorpromazine, olanzapine, risperidone, etc.

Antidepressants: used for depression, anxiety disorders, obsessive-compulsive disorder, etc. For example, selective serotonin reuptake inhibitors (such as paroxetine, sertraline).

Anti-anxiety drugs: such as benzodiazepines (diazepam, etc.), are used for short-term relief of anxiety symptoms.

Mood stabilizers: such as lithium carbonate and sodium valproate, are used for the prevention and control of bipolar disorder.

Drug treatment should be carried out under the guidance of a psychiatrist, strictly adhering to the dosage and course of treatment to avoid side effects or dependence.

Ii. Psychotherapy

Psychotherapy (also known as psychological counseling) helps patients improve their thinking and behavioral patterns through conversation and communication. Common types include:

Cognitive Behavioral therapy (CBT) : Correcting incorrect cognitive patterns and negative thinking, and improving emotions and behaviors.

Psychoanalytic therapy: Exploring subconscious conflicts to help understand intrinsic motivation.

Interpersonal relationship therapy: Focusing on the relationship between interpersonal conflicts and emotional distress.

Family therapy: Promoting recovery by improving family communication and support.

Psychotherapy is suitable for mild to moderate mental disorders or as an adjunct to medication.

🧘‍♀️ III. Physical therapy

Including the use of physical methods to regulate brain functions:

Electroconvulsive therapy (ECT) : It is suitable for patients with severe depression, those at emergency suicide risk, or those who are unresponsive to medication.

Transcranial magnetic stimulation (TMS) and transcranial direct current stimulation (tDCS) : New non-invasive treatment methods, used to improve depression, anxiety and other conditions.

Iv. Rehabilitation and Social Support Therapy

Mental rehabilitation training: Helps patients restore their social skills, working abilities and self-care abilities in daily life.

Occupational therapy: Promoting psychological rehabilitation through appropriate work activities.

Social support: The understanding and assistance from family, friends and the community are crucial for preventing recurrence.

🧩 v. comprehensive treatment

Mental illness often requires the combined use of multiple methods: medication to control symptoms, psychotherapy to regulate emotions and behaviors, and rehabilitation treatment to promote social adaptation.

**Q16Which is more effective, psychiatric medication or psychotherapy?**

Which is more effective, psychiatric drug treatment or psychotherapy, depends on the type of disease, the severity of the condition and individual differences. Both have their own advantages and often work best when used in combination. The following is a detailed analysis:

🧠 I. Characteristics of drug therapy

1. Main functions

Drug treatment improves symptoms by regulating neurotransmitters in the brain, such as dopamine and serotonin.

It is often used for depression, anxiety disorders, schizophrenia, bipolar disorder, etc.

2. Advantages

It takes effect quickly and is highly effective for acute symptoms such as hallucinations, severe depression, and suicidal thoughts.

It can relieve the emotional and thinking disorders caused by physiological imbalance.

3. Limitations:

One cannot directly change cognitive patterns or coping styles.

Side effects may occur (such as drowsiness, weight changes, decreased sexual function, etc.).

If psychological treatment is not provided after drug withdrawal, the risk of recurrence is relatively high.

Ii. Characteristics of Psychotherapy

1. Main functions

Psychotherapy (such as cognitive behavioral therapy (CBT), interpersonal therapy, psychoanalytic therapy, etc.) improves psychological function by changing thinking patterns, emotional responses and behavioral habits.

2. Advantages

It can help patients understand the root cause of problems and enhance their self-regulation ability.

It has a remarkable effect on mild to moderate depression, anxiety, post-traumatic stress disorder (PTSD), etc.

It has no side effects of drugs and can enhance long-term psychological resilience.

3. Limitations:

It takes a long time to take effect and requires a stable therapeutic relationship and continuous investment.

It has limited therapeutic effect on severe psychotic symptoms such as hallucinations and delusions.

Iii. Combined Therapeutic Effect

Studies show that the combination of drug treatment and psychotherapy often yields the best results.

For example:

The remission rate of combined treatment for patients with moderate to severe depression is significantly higher than that of single treatment.

Medication can first stabilize emotions, while psychotherapy helps prevent recurrence and improve function.

✅ 4. Summary and suggestions

| Condition | Recommended Treatment Approach |
| --- | --- |
| Mild anxiety, mild depression | Psychotherapy as primary approach, medication may not be necessary |
| Moderate to severe depression, OCD | Combined medication and psychotherapy |
| Schizophrenia, bipolar disorder | Medication as primary approach, supplemented by psychological intervention |
| Trauma, emotional distress, personality issues | Psychotherapy as primary approach |

Conclusion

Drug treatment and psychotherapy are not in a relationship of "which is more effective", but rather complement each other. Drugs can regulate the biological basis, and psychotherapy can promote long-term psychological growth and recovery. The best plan should be jointly developed by psychiatrists and psychotherapists, based on a comprehensive assessment of the individual's condition.

**Q17Is exercise effective for mental illness?**

Exercise has a significant positive effect on mental illness and has been confirmed by a large number of studies to be effective in the prevention, auxiliary treatment and improvement of various mental disorders. The following will explain this point from several perspectives:

I. Effects on Depression

Exercise is regarded as one of the most effective non-pharmaceutical means of treating depression.

Research shows that regular aerobic exercise (such as brisk walking, running, and cycling) 3 to 5 times a week for 30 to 45 minutes each time can significantly alleviate depressive symptoms.

The mechanisms include:

Promote the secretion of neurotransmitters such as dopamine, serotonin and norepinephrine;

Increasing the level of brain-derived neurotrophic factor (BDNF) is beneficial for neural plasticity.

Improve sleep, enhance self-efficacy and social interaction.

Ii. Effects on Anxiety Disorders

Exercise can effectively relieve anxiety symptoms.

Regular exercise can reduce stress hormones such as cortisol.

Aerobic exercise, combined with mind-body exercises such as yoga and Tai Chi, can all help individuals better regulate their physiological arousal levels.

Long-term persistence can enhance one's tolerance to pressure.

Iii. Auxiliary Effects on Schizophrenia and Bipolar Disorder

For patients with schizophrenia, exercise can improve cognitive function and alleviate negative symptoms such as apathy and social withdrawal.

In bipolar disorder, moderate exercise can help stabilize emotions, improve sleep and regularity of life.

It can also reduce the risk of drug side effects such as metabolic syndrome.

Iv. Overall Benefits for Cognitive Function and Mental Health

Exercise can enhance the activity of the prefrontal cortex and the hippocampus, which is conducive to improving attention, memory and decision-making ability.

For the general population, exercise is also an important lifestyle for preventing mental illness and maintaining mental health.

V. Precautions

Exercise prescriptions should be formulated based on individual conditions to avoid stress responses caused by excessive exercise.

For patients with moderate to severe mental disorders, the treatment should be carried out under the guidance of professional doctors and psychologists.

The most ideal solution is a comprehensive approach of "exercise + psychological intervention + drug treatment".

Conclusion

Exercise has definite therapeutic and preventive effects on mental disorders, especially in the auxiliary treatment of depression, anxiety and schizophrenia, it can significantly improve symptoms, enhance the quality of life and social functions.

**Q18What types of drugs are commonly used to treat mental disorders?**

The commonly used drugs for treating mental disorders are mainly classified into the following categories, each targeting different mental symptoms or disease mechanisms:

I. Antipsychotics

Main uses: Treating manic episodes of schizophrenia, bipolar disorder, delusional disorders, etc.

Common medications

Typical antipsychotic drugs (first-generation) : chlorpromazine, haloperidol, perphenazine, etc.

Characteristics: The therapeutic effect is definite, but there are many side effects, such as extrapyramidal reactions (hand tremors, stiffness).

Atypical antipsychotic drugs (second generation) : risperidone, olanzapine, quetiapine, aripiprazole, clozapine, etc.

Features: It has relatively mild side effects and is more effective in improving negative symptoms such as apathy and withdrawal.

Ii. Antidepressants

Main uses: Depression, anxiety disorder, obsessive-compulsive disorder, panic disorder, etc.

Common medications

Selective serotonin reuptake inhibitors (SSris) : fluoxetine (Prozac), sertraline, citalopram, etc.

Features: It has relatively few side effects and is often the first choice.

Serotonin and norepinephrine reuptake inhibitors (SNris) : venlafaxine, duloxetine.

Tricyclic antidepressants (TCA) : amitriptyline, doxepin.

It has many side effects and is now less of the first choice.

Other types: mirtazapine, bupropion, etc.

Iii. Anti-anxiety drugs (Anxiolytics)

Main uses: Various anxiety disorders, tension, insomnia.

Common medications

Benzodiazepines: Diazepam (Diazepam), lorazepam (lora), alprazolam.

Features: Quick onset, but prone to dependence after long-term use.

Non-benzodiazepines: Buspirone, no obvious dependence.

Iv. Mood stabilizers

Main uses: Prevention of manic and depressive phases of bipolar disorder.

Common medications

Lithium salt (lithium carbonate) : A classic mood-stabilizing drug.

Antiepileptic drugs: sodium valproate, lamotrigine, carbamazepine, etc.

V. Hypnotics

Main uses: Insomnia, anxiety-induced insomnia.

Common medications

Benzodiazepines: temazepam, estazolam.

Non-benzodiazepine hypnotics: Zaleplon, Zopiclone.

Vi. Other adjuvant medications

Beta-blockers, such as propranolol, can alleviate palpitations and tremors caused by anxiety.

Antiparkinsonian drugs: Used to alleviate extrapyramidal reactions caused by antipsychotic drugs.

**Q19How long does it usually take for psychiatric drugs to take effect?**

The time it takes for psychiatric drugs to take effect depends on the type of drug, individual differences, and the disease being treated. The following are the approximate onset times of common psychiatric medications:

🧠 antidepressants (such as SSRI, SNRI, tricyclic drugs)

Onset time: It usually takes 2 to 4 weeks to notice a significant improvement in mood.

The reason is that these drugs work by regulating brain neurotransmitters such as serotonin and norepinephrine, and the neuroregulatory process takes time.

Note: Mild side effects (such as nausea and changes in sleep) may occur initially, but they usually subside within one or two weeks.

Anti-anxiety drugs

Benzodiazepines (such as lorazepam, alprazolam) :

Onset time: Anxiety can be relieved within 30 minutes to several hours.

Note: Only suitable for short-term use. Avoid reliance.

Non-addictive anti-anxiety drugs (such as buspirone) :

Onset time: It takes about 1 to 3 weeks to exert a stable effect.

Antipsychotic drugs (such as olanzapine, risperidone, aripiprazole)

Effective time

Positive symptoms such as hallucinations and delusions may start to improve within 1 to 2 weeks.

Negative symptoms such as emotional apathy and lack of motivation: Take 4 to 8 weeks or longer.

Dosage adjustment and tolerance vary from person to person.

Mood stabilizers (such as lithium salts, valproic acid, lamotrigine)

Onset time: Generally, it takes effect within 1 to 3 weeks.

Usage: It is often used for controlling manic or depressive episodes in bipolar disorder.

Summary

| Drug Class | Onset of Action (Approximate) | Remarks |
| --- | --- | --- |
| Antidepressants | 2-4 weeks | Patience is required for mood improvement. |
| Anxiolytics (Benzodiazepines) | Several hours | Rapid onset but carries risk of dependence. |
| Anxiolytics (Buspirone) | 1-3 weeks | Requires continuous administration. |
| Antipsychotics | 1-8 weeks | Improvement in negative symptoms is typically slower. |
| Mood Stabilizers | 1-3 weeks | Blood level monitoring is required (e.g., for Lithium). |

**Q20Will taking psychiatric drugs damage brain function?**

This is a very common but also easily misunderstood question. Let's explain it separately from a scientific perspective:

I. General Conclusion

Most psychiatric drugs, when used rationally and under the monitoring of doctors, do not damage brain function.

On the contrary, they are often aimed at restoring the neural functions that have been damaged by mental illness. Mental illnesses (such as depression, schizophrenia, bipolar disorder, etc.) themselves can cause disorders in neural networks and neurotransmitter systems, while drugs regulate these systems to normalize brain functions.

Ii. How do Drugs Affect the Brain

Psychiatric drugs (such as antidepressants, antipsychotics, anti-anxiety drugs, mood stabilizers, etc.) mainly work by influencing the neurotransmitter system, including:

Serotonin (serotonin)

Dopamine

Norepinephrine

γ -Aminobutyric acid (GABA

These chemical substances regulate functions such as mood, motivation, attention and sleep. The goal of the drug is to restore balance, not to damage the brain.

Iii. Is there a risk of "damage"

Long-term, high-dose or improper use of psychiatric drugs may indeed cause some side effects, but this is different from "damaging the brain" :

Reversible side effects: such as slow response, decreased attention, weight changes, drowsiness, etc., usually improve after discontinuing the medication or adjusting the dosage.

Irreversible damage is extremely rare: long-term and high-dose use of some older generation antipsychotic drugs (such as chlorpromazine) may lead to extrapyramidal symptoms (such as tardive dyskinesia), but modern drugs have significantly reduced the risk.

Abuse or self-discontinuation of medication: Repeatedly changing or increasing or decreasing the dosage of drugs on one's own may disrupt the neurotransmitter system and have a temporary negative impact on cognition.

Iv. Comparison with Mental Illness Itself

It is worth noting that untreated mental illness itself is more likely to cause damage to brain function.

For example:

Depression can lead to a reduction in the volume of the hippocampus and a decline in memory.

If schizophrenia is not controlled, it can lead to the loss of gray matter and cognitive decline.

Drug treatment can prevent these changes from worsening and even promote neurological recovery in some patients.

V. Summary and Suggestions

Taking medicine on time under the guidance of a doctor is safe and effective.

Do not increase or decrease the dosage of the medicine by yourself.

If there is obvious cognitive impairment or abnormal emotions, one should promptly report to the doctor and adjust the treatment plan.

Combining psychotherapy, regular schedules, exercise and social interaction is more helpful for restoring brain function.

**Q21How to deal with the side effects of psychiatric drugs, such as weight gain and drowsiness?**

Dealing with the side effects of psychiatric drugs (such as weight gain and drowsiness) requires a combination of doctor's guidance, lifestyle adjustments and drug management. The following are systematic coping strategies:

🧠 1. Understand the causes of side effects

Many psychiatric drugs (such as antidepressants, antipsychotics, and mood stabilizers) can affect the central nervous system or metabolic functions:

Weight gain: Often due to medication stimulating appetite, slowing metabolism or affecting insulin sensitivity;

Drowsiness: It usually results from the inhibitory effect of drugs on the central nervous system or the antihistamine effect.

Understanding the specific drug mechanisms (for example, olanzapine and chlorpromazine are more likely to cause weight gain, while sertraline is less so) can help discuss alternative or adjustment plans with your doctor.

Ii. Strategies for Dealing with Weight Gain

Diet management

Control the intake of sugar and refined carbohydrates;

Increase high-fiber and high-protein foods (such as soy products, fish, and chicken breasts);

Adopt the principle of "eating small meals frequently" to prevent overeating.

Regular exercise

At least 150 minutes of aerobic exercise per week (such as brisk walking, swimming, cycling);

Do strength training twice a week to increase your basal metabolism.

Monitor body weight and metabolic indicators

Regularly measure your weight and waist circumference;

Doctors may recommend checking indicators such as blood sugar and blood lipids.

Medication adjustment (Doctor's guidance required)

Sometimes, drugs with milder metabolic side effects (such as aripiprazole and ziprasidone) can be used instead;

Metabolic adjuvant drugs (such as metformin) may also be added as appropriate.

Iii. Strategies for Dealing with Excessive Sleepiness

Adjust the medication time

If the medicine causes obvious drowsiness, you can try taking it before going to bed.

When some medications are taken in divided doses, doctors can adjust the dosage distribution.

Daily routine

Keep a fixed wake-up time;

Try to get some sun exposure and do light activities during the day to stay alert.

Avoid superimposing sedative factors

Reduce the use of alcohol, antihistamines or other sedatives.

Discuss the adjustment plan with the doctor

If excessive sleepiness seriously affects work or study, it should be reported in time. The doctor may adjust the dosage or type of medication.

Iv. Psychological and Behavioral Support

Keep communicating with your doctor and family, and do not stop taking the medicine on your own due to side effects.

If weight changes or excessive sleepiness lead to low mood, one can seek psychological counseling.

Keep a diary of medication and side effects to help doctors evaluate and adjust the effects.

Warm reminder

Do not reduce or stop taking the medicine on your own, as this may cause a recurrence or withdrawal reaction. Any adjustments should be made under the guidance of a doctor.

**Q22Can taking psychiatric drugs lead to addiction?**

This question is very good and is also the one that many people care about the most when they start taking psychiatric drugs.

In simple terms: Most psychiatric drugs are not addictive, but a few require special attention.

1. Drugs that generally do not cause addiction

The following common types of psychiatric drugs do not cause addiction or dependence:

Antidepressants (such as sertraline, paroxetine, escitalopram, etc.)

It won't make people develop a psychological dependence of "wanting to keep eating".

If the medication is stopped too quickly, "withdrawal reactions" (such as dizziness and fatigue) may occur, but this is not "addiction", but rather an adaptation process of the body. The doctor will guide the gradual reduction of medication.

Antipsychotic drugs (such as olanzapine, risperidone, quetiapine)

It is used for the treatment of schizophrenia, bipolar disorder, etc.

It won't be addictive and won't make people "unable to do without the medicine".

Long-term medication is needed to prevent recurrence, which is different from "addiction".

Mood stabilizers (such as lithium carbonate, sodium valproate, lamotrigine, etc.)

It will not cause addiction.

It is mainly used to prevent mood swings (such as in patients with bipolar disorder).

Ii. Medications that need to be used with caution

There is a type of drug that may cause dependence. Usually, doctors strictly control the dosage and duration of use:

Benzodiazepine anti-anxiety drugs (such as alprazolam, lorazepam, diazepam, etc.)

It is mainly used for short-term relief of anxiety and insomnia.

Sudden discontinuation of medication after long-term and high-dose use may lead to withdrawal symptoms (such as anxiety, insomnia, palpitations, etc.).

Therefore, it should be used under the guidance of a doctor. Do not increase the dosage by yourself or stop taking the medicine suddenly.

Iii. Summary

The vast majority of psychiatric drugs are not addictive.

Only a small number of drugs (especially diazepam drugs) may cause dependence, but doctors will arrange their use reasonably.

If you are concerned about side effects or dependence issues, be sure to communicate with a psychiatrist and do not stop taking the medicine or adjust the dosage on your own.

**Q23. What is electroconvulsive therapy without convulsions?**

Modified Electroconvulsive Therapy (abbreviated as MECT) is a modified electroconvulsive treatment method, mainly used to treat certain serious mental disorders, such as depression, bipolar disorder, schizophrenia, etc. It is an improved version of the traditional electroconvulsive therapy (ECT).

I. Therapeutic Principle

Non-convulsive electroconvulsive therapy involves briefly applying a certain intensity of current to the patient's brain to trigger a controllable and brief electrical activity in the brain (equivalent to an artificially induced epileptiform discharge). This kind of discharge can cause changes in the levels of neurotransmitters (such as serotonin, dopamine, norepinephrine, etc.) in the brain, thereby improving the patient's mood, thinking and behavioral disorders.

Ii. Differences from Traditional Electroconvulsive Therapy

Traditional electroconvulsive therapy (ECT) can cause obvious convulsive reactions throughout the body, thus posing certain risks and discomforts.

The "no convulsions" feature is due to the use of short-acting anesthetics and muscle relaxants before MECT treatment. Patients receive electrical stimulation under anesthesia and do not experience obvious convulsive movements, significantly enhancing safety and comfort.

Iii. Treatment Process

The patient received short-acting general anesthesia and muscle relaxation under supervision.

The doctor placed electrodes on the head and provided appropriate electrical stimulation.

The entire process lasts only tens of seconds.

After waking up, patients usually have no obvious pain. Some may experience mild headaches or temporary memory loss.

Iv. Indications

Those with severe depression, especially those at risk of suicide;

Depressive or manic episodes of bipolar disorder;

Acute stage of schizophrenia

Patients with mental disorders who do not respond well to drug treatment.

V. Safety and Side Effects

Modern MECT has a relatively high level of security and poses extremely low risks under professional supervision. Common side effects include:

Transient memory impairment (usually recoverable);

Headache, fatigue;

Temporary discomfort caused by anesthesia.

✅ summary:

Electroconvulsive therapy without convulsions is a modern, safe and effective psychiatric treatment method. It adjusts brain function through brief electrical stimulation and is used to rapidly relieve severe mental symptoms. Compared with traditional electroconvulsive shock, it is safe and comfortable and has been widely used around the world.

**Q24Which diseases is transcranial magnetic stimulation therapy mainly applicable to?**

Transcranial Magnetic Stimulation (TMS) is a non-invasive neuroregulation technique that uses magnetic field pulses to stimulate the cerebral cortex and is mainly used in the fields of psychiatry and neurology. Its indications mainly include the following types of diseases:

🧠 I. mental illness

Depression

TMS is currently one of the main indications approved by the US FDA, especially suitable for patients with treatment-resistant depression who have a poor response to antidepressant drugs. Stimulating the left dorsolateral prefrontal cortex (DLPFC) can improve emotional regulation function.

Anxiety disorder

Including generalized anxiety disorder, obsessive-compulsive disorder (OCD), etc. TMS can regulate neural circuits related to anxiety and compulsive behaviors (such as the frontal lobe-striatum pathway), and has been approved in some countries for the adjuvant treatment of obsessive-compulsive disorder.

Schizophrenia

For patients with auditory hallucinations, TMS stimulation of the temporoparietal lobe can reduce the intensity and frequency of auditory hallucinations.

🧩 2. Neurological disorders

Rehabilitation after stroke

TMS can promote the reorganization of brain functions and improve functional disorders such as hemiplegia and speech disorders after stroke.

Parkinson's disease

By stimulating the motor cortex or prefrontal cortex, TMS helps improve bradykinesia and some non-motor symptoms.

Chronic pain syndrome

Such as neuropathic pain, migraine, fibromyalgia, etc., pain perception can be alleviated by inhibiting or enhancing the excitability of specific brain regions.

Epilepsy

Low-frequency TMS can reduce cortical excitability and is used to decrease the seizure frequency of some patients with refractory epilepsy.

Iii. Other potential Indications (Under Study)

Alzheimer's disease and mild cognitive impairment (MCI

Insomnia

Tinnitus

Addiction (such as smoking addiction, drinking addiction

Summary

The most definite clinical indication for transcranial magnetic stimulation therapy at present is treatment-resistant depression. Other areas such as obsessive-compulsive disorder, auditory hallucinations, post-stroke rehabilitation, and chronic pain are relatively mature or under research for adaptation. Whether it is applicable or not should be evaluated by a professional physician based on the condition.

**Q25Can one stop taking psychiatric medication on their own after symptoms improve?**

It is not recommended to stop taking psychiatric medications on your own after your symptoms have improved. The reasons are as follows

The condition may not yet be stable

Mental disorders (such as depression, anxiety disorders, bipolar disorder, schizophrenia, etc.) often require continuous treatment. The improvement of symptoms does not mean complete recovery. Medication helps stabilize the balance of neurotransmitters in the brain. Stopping the medication too early may lead to recurrence or relapse of the condition.

Sudden discontinuation of medication may trigger withdrawal reactions

If certain medications (such as antidepressants, anti-anxiety drugs, and antipsychotic drugs) are suddenly discontinued, withdrawal symptoms may occur, such as dizziness, insomnia, anxiety, mood swings, physical discomfort, etc., and may even lead people to mistakenly believe that the condition has worsened.

The dosage should be gradually reduced under the guidance of a doctor

Discontinuation or reduction of medication should be determined by a psychiatrist after assessing the stability of the condition, and a gradual dosage reduction plan should be formulated. During the process, the reaction should be closely observed. Doctors will adjust the speed and dosage according to individual conditions to ensure a safe transition.

Psychotherapy is as important as life support

In addition to medication, measures such as psychotherapy, regular sleep, exercise and social support are very important for consolidating the therapeutic effect. One cannot rely solely on subjective feelings to determine whether medication can be stopped.

Summary

After symptoms improve, do not stop taking the medicine on your own. You should consult a psychiatrist and let the doctor assess and formulate a scientific plan for reducing or stopping the medication. Only in this way can the risk of recurrence be minimized to the greatest extent and long-term stability be ensured.

**Q26How long should psychiatric medication continue to be taken after the condition stabilizes?**

The duration of continued use of psychiatric drugs after the condition stabilizes should be determined based on the specific type of disease, the severity of the disease course, and individual differences among patients. The general principles are as follows:

I. Depression

For the first episode: After the symptoms have completely subsided, it is usually recommended to continue taking the medication for 6 months to 1 year to consolidate the therapeutic effect and prevent recurrence.

For those with multiple relapses or a longer course of the disease: Maintenance treatment for more than two years is required. Some people may need to take medication for a long time or even for life.

Ii. Anxiety Disorders (such as generalized anxiety disorder, panic disorder, etc.)

After the symptoms are well controlled, it is usually recommended to continue the treatment for 6 to 12 months before gradually reducing or discontinuing the medication. Stopping the medication too early can easily lead to recurrence.

Iii. Bipolar Disorder

Even if one's mood is stable, it is necessary to maintain medication (commonly mood stabilizers) for a long time to prevent the recurrence of manic or depressive episodes.

Most patients require lifelong maintenance treatment.

Iv. Schizophrenia

Even if the symptoms are relieved, it is still recommended to maintain the treatment for at least two years. For those with multiple episodes or a long course of the disease, long-term or even lifelong medication is often required.

V. General Suggestions

Do not stop taking the medicine by yourself. You should gradually reduce the dosage under the guidance of a doctor.

Doctors will conduct a comprehensive assessment based on the risk of recurrence, past medical history, drug reactions, and the recovery of social functions.

Regular follow-up should be conducted after drug withdrawal to monitor for early signs of recurrence.

**Q27Can mental illness be completely cured?**

This issue requires specific analysis because whether mental illness can be completely cured depends on factors such as the type of the disease, its severity, individual differences, and treatment conditions. The following is the detailed description:

The prognosis of different types of mental disorders varies greatly

Diseases that can be completely cured or relieved for a long time

Many patients with neurotic disorders such as mild depression, anxiety, phobias and obsessive-compulsive disorder can recover to a healthy state after receiving standardized psychological treatment (such as cognitive behavioral therapy) and necessary medication.

Some acute stress disorders and adjustment disorders can also be fully recovered after the stressors are removed and psychological support is received.

Diseases that can be controlled but are difficult to be completely cured

Schizophrenia, bipolar disorder and other chronic mental illnesses usually require long-term or even lifelong drug treatment and psychosocial support. Although the possibility of a complete cure is relatively low, through systematic treatment, most patients can maintain stability and restore social functions.

Mental disorders related to physiological or degenerative factors:

Such as Alzheimer's disease and senile dementia, there is currently no cure in medicine. The only way is to delay the course of the disease and alleviate the symptoms.

Ii. Key Factors Influencing Cure and Rehabilitation

Early intervention: The earlier treatment is received, the better the prognosis is usually.

Comprehensive treatment: The combination of drug therapy, psychological therapy, and family and social support yields the best results.

The patient's active cooperation: adhering to treatment, taking medicine regularly, and maintaining good living habits.

Social environment and support: A social environment that is understanding, accepting and non-discriminatory is conducive to recovery.

Iii. Summary

In psychiatry, "cure" often means the disappearance of symptoms and the restoration of social functions, but it does not necessarily mean that the condition will never recur.

In other words, most mental illnesses can be effectively controlled, relieved for a long time or even close to cure, but cases of complete and permanent non-recurrence are rare. The key lies in scientific treatment, long-term follow-up and social support.

**Q28Will mental illness recur after recovery?**

Whether a mental illness will recur after recovery depends on the specific type of illness, the individual's condition and the management situation after recovery. The following is a general analysis:

One, it may recur

Most mental illnesses (such as depression, bipolar disorder, schizophrenia, etc.) have a certain risk of recurrence. Even if symptoms are relieved through systematic treatment, if regular medication, psychological support or a healthy lifestyle are not maintained in the later stage, the condition may still recur.

For example:

The recurrence rate of depression is approximately over 50%.

If bipolar disorder is not treated continuously, the recurrence rate can be as high as 70% to 90%.

If medication for schizophrenia is stopped too early, the recurrence rate may exceed 80%.

Ii. Common Triggers for Recurrence

Stopping or reducing medication without authorization: Many patients stop taking their medicine when they feel better, which leads to a recurrence of symptoms.

Excessive stress: Life stress events such as work, study and family can trigger recurrence.

Insufficient sleep or disrupted sleep schedules: It has a particularly significant impact on emotional disorders.

Drug abuse or alcohol consumption: It can interfere with the function of the nervous system and increase the risk of recurrence.

Lack of social support: Loneliness, lack of understanding and assistance may lead to the deterioration of the condition.

Iii. Methods to Reduce the Risk of Recurrence

Adhere to long-term maintenance treatment (medication + psychotherapy) under the guidance of a doctor.

Establish a regular daily routine, ensure adequate sleep and a healthy diet.

Learn stress management and emotion regulation skills, such as meditation, exercise, and psychological counseling.

Regular follow-up visits and timely adjustment of the treatment plan.

The support from family and friends can help identify the signs of recurrence at an early stage.

Iv. Conclusion

After recovery from mental illness, there is a possibility of recurrence. However, through scientific treatment, long-term maintenance, and good psychological and social support, the possibility of recurrence can be significantly reduced, and even remain stable for a long time.

**Q29How to prevent the recurrence of mental illness?**

Preventing the recurrence of mental illness is a long-term and systematic process that requires joint efforts at multiple levels, including physical, psychological and social support. The following are the main preventive measures and suggestions:

First, adhere to regular treatment

Take the medicine as prescribed

Mental illnesses (such as depression, bipolar disorder, schizophrenia, etc.) usually require long-term or even lifelong medication maintenance treatment. Stopping or reducing medication without authorization can easily lead to a recurrence of the condition.

Regular follow-up visits

Visit the hospital on time for follow-up and let the doctor adjust the dosage or treatment plan according to your condition.

Understand the side effects of drugs

Understand and report adverse reactions to the doctor in a timely manner to prevent self-discontinuation of the medication due to side effects

Second, maintain a good daily routine

Regular schedule

Maintain adequate sleep and avoid staying up late.

Balanced diet and moderate exercise

A healthy body contributes to emotional stability.

Avoid irritating substances

Quit drinking and smoking, and avoid stimulants such as caffeine or drugs.

Iii. Enhance psychological adjustment ability

Learn stress management skills

Such as deep breathing, meditation, mindfulness training, yoga, etc.

Build a positive self-awareness

Learn to accept your illness and don't blame yourself or feel anxious because of it.

Psychological counseling or psychotherapy

Under the guidance of a doctor, carry out interventions such as cognitive behavioral therapy (CBT) to enhance stress resistance and coping abilities.

Iv. Establish a social support system

Keep in touch with family and friends

Tell your relatives and friends about your situation to gain their understanding and support.

Join mutual aid groups

Communicating with people who have had the same experience can help reduce feelings of loneliness and prevent the condition from worsening.

Maintain moderate social activities

Participate in work, study or interest activities to enhance self-worth.

V. Identifying Early Signs of Recurrence

Pay attention to changes in emotions, sleep and behavior

Such as insomnia, increased anxiety, low mood or suspicion, etc.

Seek help in time

Once any abnormal signs appear, contact a doctor or psychologist as soon as possible for intervention.

Establish a crisis response plan

Discuss with your family and doctor how to handle the situation if it recurs.

Vi. Cooperation between Families and Society

Family members should understand disease knowledge to avoid blame and misunderstanding.

Society should reduce discrimination and prejudice to help patients reintegrate into society, find employment and live a better life.

**Q30Can mental illness be passed on to the next generation?**

Mental illness has a certain genetic predisposition, but it is not entirely determined by genetics. In other words, genetic factors can increase the risk of illness, but this does not mean that if parents have mental illness, their children will definitely fall ill.

The following is a more specific explanation:

I. The influence of Genetic factors

Studies show that some common mental disorders (such as depression, bipolar disorder, schizophrenia, autism spectrum disorder, etc.) all have a certain genetic basis.

If one of the parents is ill, the risk of their children developing the same disease is higher than that of ordinary people.

If both parents are ill, the risk will increase further.

But even so, most children still do not fall ill.

For example:

The risk of schizophrenia in the general population is approximately 1%.

If one of the parents is ill, the risk rises to approximately 10%.

If both parents are ill, the risk is approximately 30% to 40%.

Second, environmental and psychological factors are equally important

The occurrence of mental illness is usually the result of the combined effect of genetic factors and environmental factors.

Postnatal living environment, stressful events, growth experiences, social support systems, etc. can all significantly influence whether one falls ill.

For example:

Long-term stress, trauma and lack of emotional support may induce or aggravate the disease.

Good family relationships, regular living habits and psychological counseling can significantly reduce risks.

Iii. Prevention and Response

Even if there is a family history, the risk can be reduced through the following methods:

Maintain good living habits: regular schedule, moderate exercise, and healthy diet;

Attach importance to mental health: Learn to regulate emotions and seek psychological counseling in a timely manner;

Avoid abusing drugs and alcohol;

Early intervention: If any abnormality in emotions, thinking or behavior is detected, medical attention should be sought as soon as possible.

Summary

Mental illness has a certain genetic predisposition, but it is not destined to be inherited. Through a healthy lifestyle, positive psychological support and scientific intervention, the vast majority of people can avoid falling ill or maintain a good state.

**Q31How can patients with mental illnesses better reintegrate into society?**

The social reintegration of patients with mental disorders is a complex and systematic process, which requires not only the efforts of the patients themselves but also the support of multiple parties including families, society and the government. The following analysis is conducted from several aspects:

I. Psychological and Medical Support

Continuous treatment and rehabilitation

Patients should receive systematic and standardized treatment under the guidance of doctors, including drug therapy, psychological therapy and rehabilitation training. Preventing the recurrence of the disease is the foundation for a smooth reintegration into society.

Psychological counseling and self-awareness

Through psychological counseling, cognitive behavioral therapy and other methods, help patients correctly understand the disease, enhance their confidence and self-acceptance ability, and gradually shift from the "patient role" to the "social role".

Second, family acceptance and support

Understanding and tolerance

Family members should learn about mental health knowledge, avoid discrimination and blame, and encourage patients to participate in family activities again with a positive attitude.

Establish good communication

Family members should listen to the patient's thoughts, offer emotional support and help them deal with negative emotions such as anxiety and loneliness.

Iii. Social Support and Community Services

Community rehabilitation institution

Communities can establish mental rehabilitation stations and mental health service centers to provide employment training, interest activities and social skills training for patients.

Government and Social security

Improve medical insurance reimbursement policies, disability protection and employment support plans to alleviate the economic pressure on patients and their families.

Iv. Employment and Social Integration

Vocational rehabilitation and re-employment

Through forms such as vocational assessment, skills training, and supportive employment (such as protective factories and social enterprises), help patients gradually return to the workplace.

Social acceptance and anti-discrimination

The public should break the prejudice against mental illness through publicity and education and create an inclusive social atmosphere.

V. Self-Growth and Social Participation

Cultivate interest and a sense of purpose

Encourage patients to develop their interests and hobbies, participate in volunteer services and social activities, and enhance their sense of social connection.

The power of self-help groups

By sharing experiences and encouraging each other in mutual aid groups, self-efficacy and a sense of belonging can be enhanced.

Summary

The social reintegration of patients with mental disorders is a trinity process of "doctor - family - society". Only when the medical system, family care and social acceptance work together can patients truly restore their self-esteem, rebuild their lives and achieve comprehensive physical, mental and social recovery.

**Q32How to deal with discrimination and gossip from others about people with mental illnesses?**

Dealing with discrimination and comments from others towards people with mental illnesses requires a comprehensive response at the psychological, communication and social levels. You can start from the following aspects

I. Self-protection at the psychological level

Firm self-identity: Mental illness does not equal "weakness" or "abnormality"; it is a health issue that requires treatment. Understanding this can help reduce the sense of guilt and shame caused by external prejudice.

Establish a psychological defense mechanism: When facing prejudice and malicious remarks, learn not to "internalize" them as your own problems. Emotional support can be obtained through psychological counseling, support groups or confiding in trusted friends.

Focus on rehabilitation and growth: Concentrate on self-care, regular treatment and life goals rather than others' evaluations.

Ii. Communication and Education aspects

Respond rationally and choose the right time for communication: When facing people you don't understand or misunderstand, you can explain the essence of mental illness in a calm and objective way at an appropriate time. Mental illness, like diabetes, is a disease that requires professional treatment.

Reject malice and protect boundaries: For obviously aggressive or insulting remarks, you can directly express your rejection: "Such statements make me uncomfortable. Please respect me."

Lead by example and break stereotypes: Show through your words and deeds that you are still living, studying or working positively after falling ill, allowing others to see the true face of people with mental illness.

Iii. Social and Support Aspects

Seek Allies and support: Join mental health support organizations, online communities or volunteer groups, find groups that understand and respect you, and speak out together.

Utilize legal and institutional protection: If discrimination or discussion involves insults, defamation, workplace discrimination, etc., one can legally safeguard their rights and interests, such as by following the provisions of the Civil Code, the Employment Promotion Law, and other laws to protect their legitimate rights and interests.

Advocate mental health education: Disseminate scientific knowledge through public lectures, publicity or social media to promote the overall inclusiveness and understanding of mental health in society.

Iv. Summary

The key to dealing with discrimination lies in:

Don't let prejudice define yourself;

Respond rationally and speak out bravely;

Leverage professional and social forces.

Every patient with mental illness should have the right to be respected, understood and treated equally.

**Q33Can one fall in love with someone who has a mental illness?**

This is a complex but important issue. Whether one can fall in love with someone who has a mental illness depends on several key factors rather than a simple "can" or "cannot". The following is an analysis from several perspectives:

Mental illness does not mean that one cannot fall in love

Mental illnesses (such as depression, bipolar disorder, anxiety disorder, schizophrenia, etc.) do not mean that a person lacks the ability to love and be loved. Many patients are fully capable of establishing healthy intimate relationships when they are receiving treatment and their conditions are stable.

The key lies in the severity of the illness, whether it is under treatment, and whether one has self-control of emotions and communication skills.

If the other party can recognize their situation and actively cooperate with the treatment, then this relationship can be understood as "growing through challenges".

Second, love requires reason and boundaries

You need to be clear about the responsibilities and boundaries you can assume in this relationship.

You are not a therapist. Loving someone doesn't mean being able to "cure" him. Don't turn love into a "rescue mission".

Set healthy boundaries: If the other person is emotionally out of control, aggressive or overly dependent, you need to protect your own psychological safety.

Face problems together: Encourage each other to seek professional help and learn about diseases together.

Iii. Risks and Reality

Getting along with people suffering from mental illness may present some challenges:

Large mood swings, communication difficulties, and even possible behaviors that harm oneself or others;

When the illness flares up, the other party may temporarily lose their rationality or control.

Your mental health may also be affected.

Therefore, it is very important to assess whether both parties have the conditions to maintain a healthy relationship. If you find yourself exhausted or feeling scared, it's a sign that the relationship needs to be re-examined.

Four. Ideal Approach

Honest communication: Understand the other person's condition, the causes, and the treatment methods.

Support but not rely on: Accompany the other person, but keep your true self.

Seeking professional advice: If the relationship is complex, psychological counseling is a very helpful approach.

Summary

One can fall in love with someone who has a mental illness, but the prerequisite is:

The other party is undergoing treatment and is willing to face the disease.

You have sufficient psychological maturity and a sense of boundaries.

Both sides can jointly build a safe, stable and mutually respectful relationship.

If these conditions are lacking, then this relationship might hurt both of them.

**Q34Can people with mental illnesses get married?**

Sure. People with mental illnesses can get married if they meet the legal conditions. According to the provisions of the Civil Code of the People's Republic of China (formerly the Marriage Law) :

I. Legal Provisions

Article 1043 of the Civil Code clearly stipulates that marriage must be based on the complete willingness of both the man and the woman, and neither party has any disease that prohibits marriage.

Among them, "diseases prohibited from marriage" refer to serious infectious diseases that are medically considered unsuitable for marriage or should be postponed, rather than simple mental illnesses.

Ii. Mental Illness and Marital Capacity

Whether a person with mental illness can get married mainly depends on their capacity for civil conduct:

A person with full capacity for civil conduct: A mental illness patient who can independently judge and express their will (such as with mild symptoms and good control after treatment), and has the right to marry independently.

Persons without or with limited capacity for civil conduct: If a patient is unable to recognize or control their own behavior during the onset of the illness, they cannot effectively express their intention to marry, and the marriage registration authority may not handle the marriage registration.

Iii. In actual operation

When registering a marriage, the registration authority generally does not proactively request a mental health check, unless one party's family member or another person raises an objection. If both parties are willing and can clearly express their intention to get married, they can usually register for marriage in accordance with the law.

Iv. Summary

People with mental illnesses are not automatically deprived of the right to marry. As long as they have a clear consciousness and judgment ability when getting married and are willing to marry voluntarily, they can get married in accordance with the law. Only when a patient is in a severe period of illness where they are unable to take care of themselves or express their true intentions does they not meet the legal conditions for marriage.

**Q35How to get along better with family members suffering from mental illness?**

Getting along with family members suffering from mental illness requires understanding, patience and good communication. Here are some suggestions to help you better support them and also protect your own physical and mental health

I. Understanding and Acceptance

Learn relevant knowledge

Understand the symptoms, causes, treatment methods and recovery process of the diseases suffered by family members. Knowledge can reduce misunderstandings and fears, enabling you to face things more rationally.

Information can be obtained through doctors, psychological counselors or reliable mental health websites.

Accept illness and reality

Mental illness, like other chronic diseases, requires long-term management. Accepting that "he didn't mean it this way" helps reduce accusations and conflicts.

Ii. Communication and Interpersonal Skills

Stay calm and avoid arguing

When the other party is emotionally agitated or has a confused mind, do not argue with them or try to "persuade" them. At this moment, the most important things are safety and comfort.

Listening and empathy

To show understanding and concern, for example: "I can understand that you are feeling very bad now", rather than "You are overthinking it."

Empathy makes them feel accepted and thus willing to communicate.

Set boundaries

While supporting the other party, it is important to protect your own mental space. For instance, if their behavior makes you feel stressed, you can temporarily leave the scene or clearly express: "I need a little time to calm down." "

Iii. Encourage and assist in treatment

Encourage professional assistance

Encourage them gently to seek help from a psychologist, psychiatrist or psychological counselor instead of forcing them.

For example: "I'm worried that you haven't been sleeping well recently. Shall we go and see a doctor together?"

Cooperate with the treatment plan

Help them take their medicine on time, visit the doctor and record their mood changes, but don't control them too much. Make them feel that you are a partner rather than a monitor.

Iv. Self-care and Support System

Take good care of yourself

Getting along with people with mental illnesses may consume one's emotions. Make sure you have space to rest, socialize and confide.

Seek support

Join a family support group or seek help from a psychological counselor to learn how to deal with stress and emotions.

V. Patience and Hope

The recovery from mental illness is a tortuous and lengthy process, sometimes taking several years or even longer.

Your understanding, companionship and respect are often important forces for patients' recovery.

**Q36How to determine if a family member has a recurrence of mental illness?**

To determine whether a family member is experiencing a recurrence of mental illness, it is necessary to comprehensively observe changes in multiple aspects such as behavior, emotion, language, and living habits. The following are some common reference indicators and corresponding suggestions:

🧠 I. Emotional and psychological changes

Obvious mood swings: unexplained irritability, anxiety, depression, low mood or excitement.

Loss of interest: Losing interest in activities one usually enjoys, becoming indifferent or withdrawn.

Abnormal self-evaluation: Obvious self-blame, self-abasement, or conversely, exaggeration and overestimation of oneself.

Ii. Abnormal language and thinking

Strange speech content: delusions, thoughts of persecution, exaggerated remarks or talking to oneself occur.

Confused thinking: incoherent speech, unclear logic, and inability to communicate.

Excessive vigilance or suspicion: believing that others are harming or monitoring oneself.

Iii. Behavioral and Life Changes

Disrupted sleep schedule: Sudden lack of sleep or excessive sleepiness.

Decline in living ability: not washing up, not tidying up, not eating or having abnormal eating.

Social withdrawal: Refusing to communicate with family or friends and isolating oneself.

Impulsive behavior: Unprovoked irritability, destruction of property, self-harm or escape.

📉 4. Body signals

Excessive excitement or extreme fatigue.

There have been significant changes in diet and weight.

Self-reported physical discomfort without a clear medical cause (somatization).

🧭 V. suggestions for response and handling

Keep observing: Record the duration, frequency and intensity of the changes.

Avoid conflict: Don't argue or blame. Listen calmly and accompany.

Seek professional help: If the abnormality persists or worsens, contact a psychiatrist or psychological counselor as soon as possible.

Medication compliance: If family members are taking medication, it is important to pay attention to whether they are taking the medicine regularly as prescribed by the doctor. Stopping the medication without authorization is a common cause of recurrence.

Provide a safe environment: If there are dangerous signs such as self-harm or attacking others, seek first aid or call the police immediately.

**Q37What should be done when 37 family members experience auditory hallucinations?**

When family members experience auditory hallucinations, it is necessary to remain calm, show care and respond scientifically. The following are the handling suggestions:

🧠 1. Stay calm and understanding

Do not argue or deny the content of auditory hallucinations: Direct denial or defense may make the patient feel misunderstood or hostile. One could say: "I know you heard these voices, but I didn't. Let's figure out a way together."

Avoid irritating language: Do not blame, mock or force correction.

Maintain a gentle attitude: Communicate with them in a calm and caring tone to give them a sense of security.

🩺 2. Seek medical attention promptly for assessment

Auditory hallucinations are often one of the manifestations of mental or neurological disorders. One should seek professional medical help as soon as possible.

Psychiatry or psychology: Auditory hallucinations are commonly seen in schizophrenia, bipolar disorder, severe depression, etc.

Neurology Department: If epilepsy, brain tumors, Parkinson's disease and other causes are suspected.

Comprehensive examination: The doctor may recommend blood tests, imaging (CT/MRI), or electroencephalogram (EEG) examinations.

If a person with auditory hallucinations shows suicidal, self-harming or aggressive behavior, they should immediately call the emergency number (120 in Chinese mainland) or be sent to the emergency psychiatry department.

🧩 3. Assist in establishing a safe and regular life

Accompanying medical treatment: Family support is very important for treatment compliance.

Monitoring medication: Take the medicine as prescribed by the doctor and do not stop taking it on your own.

Create a quiet environment: Reduce noise, quarrels, and stimulating films and TV shows.

Maintain a regular schedule: get sufficient sleep, have a balanced diet, and engage in moderate activities.

Iv. Psychological Support and Rehabilitation

Family education and psychological Counseling: Understanding the essence of auditory hallucinations and learning communication methods.

Cognitive behavioral therapy (CBT) : It can help patients identify the triggers of auditory hallucinations and reduce fear.

Rehabilitation training and support group: Helping patients readjust to social life.

V. Be vigilant of danger signals

If the patient:

They think that "voice" commands them to do dangerous things;

Show extreme fear, confusion or irritability;

Suicidal or aggressive intentions appear

Please seek emergency medical assistance or call the police immediately.

**Q38What should be done if a patient with mental illness shows suicidal behavior?**

When patients with mental disorders exhibit suicidal behavior, immediate emergency intervention measures must be taken to ensure their life safety, and at the same time, professional medical and psychological support should be provided to them. The following are the processing steps and suggestions:

I. Immediate Response and Emergency Measures

Ensure safety

Immediately remove all items around the patient that might be used for suicide (such as knives, ropes, drugs, etc.).

Patients should not be left alone; they should be accompanied by family members or friends.

If the patient is committing suicide, immediately call the emergency number (120 in Chinese mainland) or the police (110).

Stay calm and communicate

Talk to the patient in a calm tone and avoid blaming, arguing or questioning.

Listen carefully to make patients feel understood and cared for.

Don't underestimate or deny their pain. For instance, avoid saying "Cheer up" or "It's no big deal."

Second, seek professional help

Contact a psychiatrist or a psychological crisis intervention agency:

Take the patient to a psychiatric hospital or the psychiatry department of a general hospital as soon as possible.

If you are unable to seek medical treatment immediately, you can contact the psychological assistance hotline

National 24-hour free psychological assistance hotline: 400-161-9995

Hotline of Beijing Psychological Crisis Intervention Center: 800-810-1117 or 010-8295-1332

Life Hotline: 400-821-1217

Continuous psychological and medication treatment

For patients with mental disorders such as depression, bipolar disorder and schizophrenia, they must receive long-term medication and psychological treatment and must not stop taking the medicine without authorization.

Family members need to supervise the patient to take medicine on time and assist with follow-up visits.

Iii. Family and Social Support

Family care

Family members should spend more time with the patient and pay attention to their emotional fluctuations and changes in behavior and speech.

Establish an open communication environment to enable patients to express their pain and needs boldly.

Social resources

You can contact the community health service center or mental health volunteer organizations for support.

If a patient has long-term risks, they may consider applying for a compulsory medical assessment or community mental health management.

Iv. Warning Signals and Prevention

Be vigilant about the following high-risk suicide signals:

Frequently expressing feelings of despair, helplessness or worthlessness;

Suddenly giving valuable items or making arrangements for the deceased;

The mood suddenly calmed down but there was long-term depression in the background.

There have been attempts at suicide before.

If the above signals occur, early intervention should be carried out, and medical treatment or seeking help from professional psychological institutions should be sought in a timely manner.

**Q39What should be done if a patient with mental illness shows violent tendencies?**

When patients with mental disorders show violent tendencies, it is necessary to balance safety, prevention, timely assistance and medical intervention. It can be handled from the following aspects

1. Safety first

Immediately ensure safety: If the patient shows signs of attacking others or self-harm, prioritize safeguarding your own and others' personal safety. If necessary, maintain a distance or leave the scene.

Remove dangerous items: Try to move knives, sticks, glass and other items that may be used for attack out of the patient's reach.

Avoid provocation or argument: Do not engage in intense arguments, criticism or anger with the patient. Stay calm and use a gentle tone.

Second, seek help in a timely manner

Seek professional help: If the situation is serious, call 120 (for emergency assistance) or 110 (to report to the police) immediately and ask for professional help.

Notify family members or caregivers: If the patient is undergoing treatment, contact the attending physician or mental health institution in a timely manner.

Community support: In China, you can contact the community health service center or the local mental health management institution. They have channels for crisis intervention and medical transportation.

Iii. Medical and Psychological Intervention

Assessing the condition: Violent tendencies often indicate a worsening of the condition or poor treatment compliance (such as drug withdrawal or missed medication).

Adjusting the treatment plan: The doctor may adjust the dosage of the medication or change the treatment plan.

Hospitalization: If the violent behavior persists or is uncontrollable, the doctor may recommend short-term compulsory hospitalization (as stipulated in the Mental Health Act).

Iv. Long-term Management and Prevention

Regular medication: Ensure that patients take their medication regularly under the guidance of a doctor.

Psychological and social support: Family members should offer understanding and support, and can cooperate with psychological counseling and rehabilitation training.

Early warning identification: Pay attention to early signs (such as emotional agitation, insomnia, and verbal threats), and early intervention can prevent the situation from worsening.

**Q40How to persuade a patient with mental illness who refuses to see a doctor?**

When a person with mental illness refuses to seek medical treatment, family members or friends often feel anxious and helpless. Persuading them to see a doctor requires skill, patience and empathy. Here are some practical suggestions:

First, maintain understanding and empathy

Avoid blaming or arguing: Don't directly say "You are ill" or "you need to see a doctor", as this can easily make patients develop a defensive or denying mentality.

Show concern rather than control: Express concern in a gentle tone, for example: "I've recently noticed that you seem to be under a lot of pressure and don't sleep well. I'm very worried about you."

Listen to the other person's feelings: Let the patient express their thoughts and fears, listen carefully, and do not rush to refute or explain.

Second, reduce psychological resistance to seeking medical treatment

Explain the purpose of seeking medical treatment: Tell them that seeing a doctor is not necessarily about "being labeled as mentally ill", but rather about alleviating symptoms and improving the quality of life.

Emphasizing privacy and confidentiality: It indicates that the psychologist or psychiatrist will strictly keep the information confidential and will not disclose the patient's condition at will.

Lower the threshold: It can be suggested to start with online consultation or a regular psychological clinic instead of going directly to a specialized mental health hospital.

Starting from physical symptoms: If the patient is reluctant to admit psychological problems, they can first say, "Recently, you often have headaches and insomnia. Let's go and see if there's something wrong with your body," and then have the doctor guide them.

Iii. Seek support and assistance

Family members should maintain a unified attitude: Family members should be consistent and not have one person urging the patient to see a doctor while the other goes along with the patient's refusal.

Seek guidance from a doctor or psychological expert: Family members can first consult a professional individually to understand how to communicate better.

Leveraging the power of third parties: such as trusted friends, teachers, and religious figures of the patient, sometimes can achieve a better persuasive effect.

In severe cases, intervention is required: If the patient shows self-harm, suicide or attacks on others, medical or public security assistance should be sought immediately. If necessary, forced medical treatment can be carried out.

Four. Long-term companionship and patience

Persuasion is not something that can succeed in one go; it requires repeated communication and observation.

Provide patients with sufficient security and support to help them believe that treatment can bring about change.

Be patient during the process, without being impatient or applying pressure.

**Q41Should family members with mental illnesses be allowed to manage their finances independently?**

Whether family members with mental illnesses should be allowed to manage their finances independently needs to be comprehensively evaluated based on specific circumstances. The following are the main considerations and suggestions:

I. Types of Mental Disorders and Stability of the Condition

The impact of different mental illnesses on judgment and self-control varies greatly:

Mild or stable conditions (such as the remission period of depression or mild anxiety disorder) usually allow for the maintenance of normal cognitive and decision-making abilities under the guidance of a doctor, and personal finances can be managed under appropriate supervision.

Severe or uncontrolled conditions (such as manic episodes or acute phases of schizophrenia) may carry risks such as impulsive spending, fraud, or losing money, and it is not advisable to independently manage large sums of money.

Ii. Risk Assessment and Guardianship Arrangements

Judgment ability assessment: It is recommended that a psychiatrist issue an assessment report to determine whether the individual has the ability to manage finances and sign contracts.

Guardianship or auxiliary guardianship system: If the assessment shows that the patient has partially or completely lost the capacity for civil conduct, the court may, in accordance with the law, appoint a guardian to manage or assist in the management of the property.

Financial management approach: Hierarchical control can be adopted, such as regularly providing petty cash, setting up a co-managed account, or using automatic transfer methods to ensure daily living needs.

Third, respect and protection should be given equal weight

Even if the patient has certain limitations, they should still:

Respect their basic right to choose and dignity;

Involve them in daily expense decisions to enhance their sense of self-worth and confidence in recovery;

At the same time, protect it from economic risks or exploitation by others.

Iv. Family Communication and Legal Advice

Family members should work together with doctors and social workers to formulate a financial management plan.

If there are disputes or a formal guardianship relationship needs to be established, you can consult a lawyer or the civil affairs department to handle guardianship or trust arrangements in accordance with the law.

Conclusion

Whether to allow family members with mental illness to manage their finances independently should be determined comprehensively based on the stability of their condition, their cognitive and judgment abilities, and their risk tolerance. For most cases, "partial autonomous management under supervision" is usually a balanced solution that takes into account both respect and safety.

**Q42What is the fastest way to find a person with mental illness who has gone missing?**

When a person with mental illness gets lost, the search must be carried out in a race against time. The following is the fastest and most effective way to search. It is recommended to carry out multiple actions simultaneously

1. Report to the police immediately (within the golden 30 minutes)

Call 110 to report the incident and provide information such as the patient's mental illness, the time and place of disappearance, physical features, and clothing.

By providing photos and medical records, the police can quickly issue a notice for assistance through the "Missing Persons Information System".

If a patient has an intellectual disability or a severe mental illness, the police can be requested to file a case immediately (within no more than 24 hours).

For cases where "people with mental disorders go missing" are classified as key groups, the public security authorities usually activate the "emergency search mechanism" after reporting the case.

Second, make use of the social investigation platform

The "National Anti-Fraud Center" App, "Toutiao Xunren", "Baidu Xunren", "Tencent Public Welfare Xunren Platform", etc. can be simultaneously pushed to multiple locations across the country.

Communities, streets and village committees also have channels for assisting in the investigation of missing persons with dementia, where they can register immediately.

Post missing person information on Moments, wechat groups, and short-video platforms (with clear photos, clothing features, the time and place of disappearance, and contact information).

Iii. Inspection of Key Locations

Hospitals, stations, subway entrances, Internet cafes, temples, parks, Bridges and areas near water bodies are common places where people gather or linger.

If there are surveillance resources (such as the public security Sky Net or shop cameras), you should request the police to retrieve them.

Taxi companies or bus surveillance can assist in tracking the whereabouts.

🧭 4. By means of science and technology

If the patient wears a smart positioning bracelet or mobile phone, use the positioning function immediately.

Family members can register and release information on the "Emergency Release Platform for Missing Children" of the Ministry of Public Security (special groups are also accepted).

V. Daily Preventive Measures

Make identity cards or location wristbands for patients, clearly stating their names, family member phone numbers and disease types.

Take recent photos for future reference at any time.

The community can establish an emergency contact group for people with mental disorders who get lost to enhance the response speed.

Summary

The fastest approach is - immediate police reporting + multi-platform collaboration investigation + community linkage + technical tracking.

Every minute counts. The simultaneous release of information through alarms and the network can significantly increase the recovery rate.

**Q43As a family member of a person with mental illness, how can one relieve their own stress?**

As family members of patients with mental illnesses, they often bear great psychological and emotional pressure. To relieve one's own stress, one can start from the following aspects:

I. Correct Understanding of Mental Illness

Understanding a patient's condition, symptoms and treatment methods can help reduce fear and misunderstanding.

Learn relevant knowledge: Understand the common types of mental illnesses (such as depression, bipolar disorder, schizophrenia, etc.), their causes and treatment processes.

Understanding the essence of the disease: Some behaviors of patients are not "intentional", but part of the illness. Understanding this can reduce conflicts and feelings of guilt.

Maintain realistic expectations: Treatment and recovery take time. Set reasonable expectations and avoid being impatient or overly anxious.

Second, take good care of yourself

While taking care of patients, one should also pay attention to their own physical and mental health.

Maintain a regular lifestyle: ensure adequate sleep, a balanced diet and moderate exercise.

Set aside personal time: Engage in some relaxing activities, such as listening to music, reading, taking a walk or communicating with friends.

Expressing emotions: You can keep a diary, confide in someone you trust, or seek professional psychological counseling.

Set boundaries: Be clear about your ability range and do not undertake care tasks that exceed your capacity.

Iii. Seek external support

You are not alone. You can make use of social and professional resources.

Family support: Share care tasks with other family members and face difficulties together.

Professional assistance: Seek help from a psychologist, social worker, or mental rehabilitation institution.

Support group: Participate in the patient's family support group, exchange experiences, and gain understanding and comfort.

Community resources: Pay attention to the assistance provided by local mental health centers, psychological hotlines or public welfare organizations.

Four. Adjust your mindset and relationships

Accept imperfection: Accept the limitations of yourself and your patients, and don't be too hard on yourself.

Maintain communication: Communicate with patients in a calm manner and avoid blaming them.

Focus on the positive aspects: Record the patient's small progress and also affirm your own efforts.

Cultivate patience and empathy: Understand that the recurrence of the illness is the norm and don't be easily disappointed.

V. When Professional Intervention Is Needed

If you experience any of the following situations, you should seek psychological support as soon as possible

Long-term insomnia, anxiety or depression;

Lose interest in life;

Show signs of irritability or despair;

I feel I can no longer continue to care for the patient.

Psychological counseling or therapy not only helps you release your emotions but also teaches you scientific coping strategies.

**Q44If a child shows symptoms such as being reluctant to talk, irritable and disliking school, could there be mental or psychological problems?**

Yes, when children show symptoms such as being taciturn, irritable, and disliking school, it may be related to mental or psychological problems, but it could also be influenced by other factors. The following are some possible causes and corresponding suggestions:

🧠 I. possible mental and psychological issues involved

Emotional disorder

Depression: The manifestations of depression in children are often different from those in adults, possibly presenting as taciturn behavior, irritability, decreased interest, and academic regression, etc.

Anxiety disorders: such as separation anxiety and generalized anxiety, children may become reluctant to study or silent due to worry or fear.

Adjustment disorder or psychological stress response

When children experience major life changes (such as transferring schools, parents' divorce, or excessive academic pressure), they may exhibit behaviors such as not speaking, throwing tantrums, and avoiding studies.

Developmental or communication-related disorders

Autism spectrum disorder or delayed language development can cause children to encounter difficulties in social interaction and communication, thus manifesting as being reluctant to talk and having unstable emotions.

Attention deficit hyperactivity disorder (ADHD) is accompanied by emotional problems

Some children feel frustrated due to inattentiveness and learning difficulties, showing signs of disliking school and irritability.

Ii. Other possible non-psychopathological factors

Family education methods: Being overly strict or neglecting communication can cause children to suppress their emotions.

Excessive academic pressure: Heavy study burdens and high expectations may lead children to develop a dislike for studying.

Peer relationship issues: Being excluded or bullied can also lead to silence and irritability.

🩺 iii. Parents' coping suggestions

Pay attention and listen: Communicate more with children, do not criticize or interrupt them, and understand their true feelings.

Observe the duration and severity: If the symptoms persist for more than two weeks or significantly affect study and life, seek medical attention as soon as possible.

Seek professional help: You can take your child to a child psychiatry department, a psychological counseling center or a school psychological counseling room for a professional assessment.

Family Support and Adjustment: Creating a safe, warm and understanding family atmosphere.

Avoid labeling or blaming: A child's negative emotions are often signals for help rather than "intentional disobedience".

**Q45Can pregnant women or those preparing for pregnancy take psychiatric drugs?**

Whether pregnant women or those preparing for pregnancy can take psychiatric medications depends on a comprehensive assessment of the severity of the condition, the type of medication, pregnancy risks, and the guidance of a doctor. The following is a general medical consensus:

I. General Principles

Weighing the pros and cons: If mental illness during pregnancy is left untreated, it may lead to depression, suicide, malnutrition, premature birth or abnormal fetal development. However, drugs may also pose certain risks to the fetus.

Therefore, whether to use medication should be jointly evaluated by psychiatrists and obstetricians.

Avoid self-discontinuation of medication: Sudden discontinuation of antidepressants, antipsychotics or antianxiety drugs may cause severe relapses or withdrawal symptoms, which is detrimental to both the mother and the baby.

Individualized treatment: Select the safest and lowest effective dose of medication based on specific diseases (such as depression, bipolar disorder, schizophrenia, etc.).

Ii. Common Drug Categories and Recommendations

| Drug Category | Feasibility and Precautions |
| --- | --- |
| Antidepressants (e.g., SSRIs) | Some (e.g., sertraline, fluoxetine) have substantial data for use in pregnancy and are generally considered lower risk; however, monitor for potential neonatal adaptation syndrome. |
| Anxiolytics (Benzodiazepines) | Avoid or use only short-term at low doses if necessary; use in early pregnancy may increase risk of fetal malformations; use near delivery can cause neonatal respiratory depression. |
| Antipsychotics (e.g., Quetiapine, Olanzapine) | Second-generation antipsychotics are relatively safer; Quetiapine is often a preferred option; monitor maternal weight and blood glucose levels. |
| Mood Stabilizers (e.g., Lithium, Valproate, Carbamazepine) | High risk! Valproate and Carbamazepine are associated with fetal malformations (e.g., neural tube defects); Lithium is associated with cardiac abnormalities. Avoid or change regimen in pregnancy. |

Iii. Suggestions for Preconception and Lactation

Before conception: Communicate with your doctor in advance and gradually adjust your medication before pregnancy. If necessary, postpone conception for a short period to stabilize the condition.

Lactation: Most psychiatric drugs can be secreted into breast milk, and the risks of lactation should be evaluated. If medication continues, choose drugs with higher lactation safety (such as sertraline, quetiapine, etc.).

Iv. Key Suggestions

Be sure to adjust your medication under the guidance of a doctor. Do not stop or change your medication on your own.

Mental health is as important as fetal health. Controlling the condition is often safer than completely stopping medication.

If you are taking medication and find out you are pregnant, you should immediately consult an obstetrician and a psychiatrist to formulate a safe transition plan.

**Q46Can psychiatric drugs be taken simultaneously with other medications?**

Whether psychiatric drugs can be taken simultaneously with other medications depends on the specific type of drug, dosage and individual circumstances. Overall, it is possible to take psychiatric drugs concurrently with other medications, but caution must be exercised as there is a risk of interaction. The following is the detailed description:

I. Possible types of drug interactions

Pharmacokinetic interactions

Some medications may affect the absorption, metabolism or excretion of psychiatric drugs in the body. For example:

When antidepressants (such as SSris) are used in combination with anticoagulants (such as warfarin), the risk of bleeding may increase.

When antipsychotic drugs (such as olanzapine) are used in combination with metabolic inhibitors (such as fluoxetine), it may lead to an increase in blood drug concentration and cause side effects.

Pharmacodynamic interactions

When different drugs act on the same physiological system, they may produce superimposed or antagonistic effects.

Sedative drugs (such as benzodiazepines) used in combination with alcohol or analgesics can enhance central inhibitory effects, leading to drowsiness and even respiratory depression.

The combination of antidepressants and monoamine oxidase inhibitors (MAOI) may cause high-risk "serotonin syndrome".

Ii. Common Combinations to Avoid

Antidepressants + MAOI drugs: They can be changed at least 14 days apart.

Lithium salts + diuretics/ACEI antihypertensive drugs: may cause lithium poisoning.

Antipsychotic drugs + antiarrhythmic drugs: Increase the risk of arrhythmia (prolonged QT interval).

Iii. Safe Medication Suggestions

Do not combine or stop taking the medicine on your own: All medication adjustments should be made under the guidance of a doctor.

When visiting the doctor, inform the doctor of all the medications you are currently taking (including Chinese patent medicines and health supplements).

Regular monitoring: For certain medications (such as lithium salts and valproic acid), blood drug concentrations and liver and kidney functions need to be checked regularly.

Pay attention to adverse reactions: If symptoms such as drowsiness, dizziness, palpitations, and confusion occur, seek medical attention promptly.

Iv. Summary

Psychiatric medications can be taken concurrently with other medications, but only after being evaluated and monitored by a doctor. There may be complex interactions among different drugs, and unauthorized combination may lead to serious consequences. It is recommended that a safe combination medication plan be formulated under the joint guidance of psychiatrists and pharmacists.

**Q47Can one drink alcohol, coffee or strong tea during the period of taking psychiatric medication?**

During the period of taking psychiatric medication, it is not recommended to drink alcohol or consume large amounts of coffee and strong tea. The following are the reasons and specific explanations:

I. Alcohol (Drinking)

No alcohol is allowed.

Drug interactions: Alcohol can affect the central nervous system and produce a superimposed effect with various psychotropic drugs (such as antidepressants, anti-anxiety drugs, antipsychotic drugs, sedative-hypnotics, etc.), which may lead to drowsiness, dizziness, confusion, slow response, and even coma.

Impact on therapeutic effect: Alcohol can interfere with drug metabolism, weakening or destabilizing the drug's efficacy.

Increased side effects: Some drugs (such as chlorpromazine, olanzapine, quetiapine, etc.) taken with alcohol may cause low blood pressure, liver function damage, etc.

Conclusion: Regardless of the dosage, alcohol should be avoided during the medication period.

Ii. Coffee and Strong Tea (Caffeinated Beverages)

It should be done in moderation or avoided.

Affecting sleep and anxiety: Caffeine has the effect of stimulating the central nervous system, which can aggravate symptoms such as anxiety, insomnia, and palpitations.

Drug metabolism interference: Some antidepressants (such as fluoxetine and paroxetine) can affect caffeine metabolism, enhancing the effect of caffeine and causing palpitations and hand tremors.

Conflict with antipsychotic drugs: Caffeine may weaken the effects of sedatives or antipsychotic drugs.

Suggestion: If it is necessary to drink, limit it (no more than 1 cup of light coffee or tea per day), and avoid drinking it in the evening.

✅ summary suggestions

| Beverage | Recommended? | Suggestions |
| --- | --- | --- |
| Alcohol | ❌ Not Recommended | No amount is considered safe. |
| Coffee | ⚠️ Use with Caution | Drink in small amounts and preferably during the daytime. |
| Strong Tea | ⚠️ Use with Caution | Opt for light tea and avoid drinking in the evening. |

**Q48What are the dietary restrictions for patients with mental disorders during the medication period?**

Patients with mental disorders do need to pay special attention to their diet during medication, as certain foods can affect drug absorption, metabolism or enhance drug side effects. The following are the main dietary taboos and precautions:

First, avoid foods that may affect the efficacy of the medicine

Avoid alcohol and alcoholic beverages

When psychiatric drugs (such as antipsychotic drugs, sedative-hypnotic drugs, and anti-anxiety drugs) are taken simultaneously with alcohol, it will enhance the central inhibitory effect, leading to drowsiness, respiratory depression or decreased judgment.

Avoid caffeine intake

Caffeinated beverages such as coffee, strong tea and cola may aggravate anxiety, insomnia and palpitations, and interfere with the efficacy of some antipsychotic or antidepressant drugs.

Avoid grapefruit and its juice

Grapefruit can inhibit liver metabolic enzymes (CYP3A4), leading to an increase in the blood concentration of certain drugs (such as clozapine, quetiapine, etc.), causing drug poisoning or enhanced side effects.

Ii. Dietary taboos for Specific Medications

When taking monoamine oxidase inhibitors (MAOI) as antidepressants

Foods rich in tyramine should be avoided, such as:

Aged cheese, cured meat, fermented soy products (douchi, soy sauce, stinky tofu)

Beer, red wine

Otherwise, it may lead to a hypertensive crisis.

Those who take lithium salts (lithium carbonate)

A stable intake of salt and water should be maintained to avoid lithium poisoning caused by sudden low-salt diets or excessive sweating.

Excessive consumption of caffeinated beverages is prohibited as it can promote diuresis and affect lithium concentration.

Those taking benzodiazepine sedatives (such as diazepam and lorazepam)

It is not advisable to drink alcohol. Also, avoid taking other sedatives or sleeping pills at the same time to prevent excessive inhibition of the central nervous system.

Iii. Nutrition and Weight Management

Some antipsychotic drugs (such as olanzapine and risperidone) can cause weight gain and metabolic abnormalities.

Suggestion

Reduce high-fat and high-sugar diets;

Increase the intake of vegetables, fruits and high-fiber foods;

Engage in moderate exercise and regularly monitor your weight, blood sugar and blood lipid levels.

Iv. Other Suggestions

Regular diet: Do not take medicine on an empty stomach, especially those with strong stomach irritation.

Follow the doctor's advice: Do not change your dietary habits or medication dosage by yourself.

Consult a doctor or pharmacist: If you need to take Chinese herbal medicine, health supplements or nutritional supplements, make sure they interact with psychiatric medications.

**LLM Model:GPT-5.2 Think**

**Q1.What is mental illness?**

mental disorders refer to a category of health problems characterized by significant and persistent abnormalities in thinking, emotion, perception, behavior or personality functions. These abnormalities can cause obvious pain or functional impairment in learning, work, interpersonal relationships and other aspects. The commonly used diagnostic criteria come from ICD-11 and DSM-5-TR (evaluated by professionals, not self-diagnosed).

Common types (Examples)

Emotional disorders: depression, bipolar disorder, etc

Anxiety-related disorders: generalized anxiety disorder, panic disorder, social anxiety, post-traumatic stress disorder (PTSD), etc

Psychotic disorders: such as schizophrenia and spectrum disorders

Obsessive-compulsive disorder and related disorders: obsessive-compulsive disorder, hoarding disorder, etc

Eating and feeding disorders: anorexia nervosa, bulimia nervosa, binge eating disorders, etc

Substance and addiction-related disorders: alcohol, drug, gambling addiction, etc

Neurodevelopmental disorders: autism spectrum, attention deficit hyperactivity disorder (ADHD), etc

Personality disorders: borderline type, antisocial type, etc

The actual diagnosis must be completed by a psychiatrist or clinical psychologist.

Possible causes (" bio-psycho-social "multi-factor model)

Biological factors: genetic susceptibility, changes in neurotransmitters and brain network functions, endocrine/immune influences, etc

Psychological factors: cognitive styles, coping styles, personality traits, early experiences and traumas, etc

Social factors: stressful events, family and interpersonal relationships, social support, poverty and discrimination, etc

It is usually the result of multiple factors working together rather than a single cause.

Common manifestations

Emotions: Persistent depression, excessive irritability, elevated mood and impulsiveness, etc

Cognition: Pay attention to memory difficulties, repeated negative thoughts, delusions, fixed thoughts, etc

Behavior: Avoidance, significant changes in sleep/diet, self-harm or impulsive behavior, etc

Physical symptoms: fatigue, palpitations, shortness of breath, and body pain, but no obvious organic cause was found upon examination

Function: Decreased academic/work efficiency, estranged relationships, and restricted daily life

When should one seek professional help

The symptoms have persisted for more than two weeks without relief

It has obviously affected study/work/family/social interaction

Thoughts or plans of self-harm or harming others arise

Loss of control over substance use or difficulty in withdrawal

Previously diagnosed but now experiencing a recurrence of symptoms

Evaluation and Diagnosis

Conducted by psychiatrists and clinical psychologists: medical history interviews, scale evaluations, and physical examinations/laboratory tests if necessary to rule out physical causes.

The diagnosis is based on the ICD/DSM criteria and takes into account cultural and individual differences.

Treatment and support

Psychotherapy: Cognitive behavioral therapy (CBT), mindfulness, exposure therapy, interpersonal therapy, family therapy, etc

Drug treatment: Antidepressants, mood stabilizers, anti-anxiety drugs, antipsychotic drugs, etc. (Follow the doctor's advice and have regular follow-ups)

Comprehensive intervention: sleep/exercise/diet management, stress and time management, peer assistance, occupational and social function support

Acute risk management: If there is a risk of self-injury or injury to others, seek immediate medical attention or contact local emergency medical services and crisis support

Common misunderstandings

"Bad mood = mental illness" ❌ : brief mood swings are not the same as disorders; The key lies in sustainability and functional impairment.

"Willpower will do" ❌ : Mental illness, like other illnesses, requires professional treatment and systematic support.

"Taking medicine can be addictive" ❌ : the compliance and safety of most psychiatric drugs are controllable under professional monitoring.

If you or someone around you is troubled by the above problems, it is wise to contact a local psychiatric clinic or psychological counseling service as soon as possible. In case of emergency danger, please contact your local emergency service or crisis hotline immediately for immediate assistance.

**Q2What are the common types of mental illnesses?**

The following is a quick overview that is easy to understand, classified by common types (mainly based on the DSM-5/ICD-11 approach; the names may vary slightly among different systems) :

Emotional (mood) disorders

Major depressive disorder: persistent depression, loss of interest, changes in sleep/appetite, self-blame, etc.

Bipolar disorder: The manic phase (energetic, sleep less, talkative/impulsive) alternates with the depressive phase.

Persistent depressive disorder (poor mood) : Symptoms are milder but more persistent.

Anxiety-related disorders

Generalized anxiety disorder: Long-term excessive worry and tension that makes it difficult to relax.

Panic disorder: Sudden intense fear accompanied by palpitations, shortness of breath and other panic attacks.

Social anxiety disorder: Intense anxiety and avoidance in social or watched situations.

Specific fear: Excessive fear of certain objects/situations (such as heights, animals).

Separation anxiety, agoraphobia, etc.

Trauma and stress-related disorders

Post-traumatic stress disorder (PTSD) : Re-experiencing (nightmares/flashbacks), avoidance, hypervigilance, negative emotions/cognitive changes.

Acute stress disorder, adjustment disorder, etc.

Compulsion and related disorders

Obsessive-compulsive disorder (OCD) : Recurring obsessive thoughts and compulsive behaviors aimed at reducing anxiety.

Trichotillomania, skin scratching disorder, hoarding disorder, etc.

Psychotic disorder

Schizophrenia spectrum disorder: delusions, hallucinations, disordered thinking/speech, emotional apathy, etc.

Paranoid disorder, transient psychotic disorder, etc.

Personality disorders (long-term stable impairment of personality and function)

Group A: Paranoid, schizoid, schizoid.

Group B: Antisocial, borderline, performative, narcissistic.

Group C: Avoidant type, dependent type, obsessive type.

Eating and eating disorders

Anorexia nervosa, bulimia nervosa, binge eating disorder, avoidance/restrictive eating, etc.

Disorders related to substance use and addiction

Disorders of alcohol, nicotine, stimulants, opioids, etc. Gambling disorders and other behavioral addictions.

Physical symptoms and related disorders

Somatic symptom disorders, disease anxiety disorders, transition disorders, etc. (with significant interaction between body and mind).

Dissociative disorder

Dissociative amnesia, depersonalization/reality-disintegration disorder, dissociative identity disorder, etc.

Sleep-wake disorder

Insomnia, narcolepsy, sleep apnea, circadian sleep-wake disorder, etc.

Disorders related to sexual function and sexual preference

Sexual dysfunction such as erectile dysfunction and low libido; Sexual preference disorder (bounded by harm/risk caused).

Neurodevelopmental disorders (commonly seen in childhood onset, but can also persist into adulthood

Autism spectrum disorder, attention deficit hyperactivity disorder (ADHD), learning disabilities, motor/tic (Tourette's) disorders, intellectual disabilities, etc.

Neurocognitive impairment

Mild/severe neurocognitive impairment (such as Alzheimer's disease, which leads to memory and functional decline).

Impulse control and conduct related disorders

Intermittent outbreak disorder, oppositional defiant disorder, conduct disorder, etc.

Tips

Many disorders often occur concurrently (such as anxiety + depression, substance use + mood disorders), and diagnosis requires professional assessment.

"Symptom resemblance" does not equal "yes". The duration, severity, functional impairment and the exclusion of other causes are all crucial.

If you or someone around you experiences persistent depression, intense anxiety, impaired reality tests, or obvious risks of self-harm or harm to others, please seek professional help as soon as possible. In case of an emergency, please contact your local emergency/police number or go to the nearest emergency department.

**Q3Is anxiety disorder a mental illness?**

Yes.

anxiety disorders fall under the category of "mental illnesses/mental disorders" and are officially classified as a group of common psychological/mental disorders in authoritative diagnostic systems such as ICD-11 and DSM-5, rather than "overthinking" or "poor willpower".

Brief key points

Unlike normal anxiety: Normal anxiety is a temporary response to stress; Anxiety disorders last longer, are more intense, and significantly affect functions such as learning, work, interpersonal relationships, or sleep.

Common types: generalized anxiety disorder, panic disorder (panic attack), social anxiety disorder, specific phobias, agoraphobia, separation anxiety, etc.

Diagnosable and treatable: The assessment is usually conducted by a psychiatrist or clinical psychologist. Effective treatments include psychotherapy (especially cognitive behavioral therapy (CBT)) and medication (such as SSRIs/SNRIs, etc.). Lifestyle and stress management are also very important.

Good prognosis: Most people can significantly alleviate or even recover after treatment.

If you or someone around you is affected by these troubles in their daily functions, seeking professional assessment as soon as possible is the best next step. If you experience self-harm or suicidal thoughts, please contact your local first aid and crisis support channels immediately.

**Q4Is depression a mental illness?**

Yes. Depression (major depressive disorder, etc.) is officially classified as a mental illness/mental disorder and is part of the "depressive disorder" spectrum in both the International Classification of Diseases (ICD-11) and the Diagnostic Manual of Mental Disorders (DSM-5) of the United States.

Simple key points

Typical characteristics: Significant depression or loss of interest/pleasure lasting for at least two weeks, accompanied by impaired functions such as sleep, appetite, energy, attention, and changes in self-evaluation.

The cause is the combined effect of multiple factors such as biological, psychological and social ones, and it is not just a matter of "being more open-minded".

Treatable: Common approaches include psychotherapy (such as cognitive behavioral therapy), medication (such as SSRIs/SNRIs), physical therapy when necessary (such as rTMS/ECT), and lifestyle support. The earlier the intervention, the better the prognosis.

If you have thoughts or plans of self-harm or suicide, please contact your local emergency or crisis intervention hotline as soon as possible and seek professional help.

**Q5What are the main factors that cause mental illness?**

Brief statement: Mental (psychological) disorders are usually not caused by a single factor, but rather the result of the combined effect of multiple factors such as susceptibility and stress (the bio-psycho-social model).

The main related factors can be classified as:

Biological factors

Genetic susceptibility: Individuals with the same or similar diagnoses in the family have a higher individual risk (not necessarily developing the disease).

Abnormal functions of the brain and neurotransmitters: such as imbalance in the regulation of dopamine, 5-HT, norepinephrine, GABA, etc.

Development and prenatal/perinatal effects: Infection during pregnancy, malnutrition, hypoxia, premature birth, low birth weight, etc.

Internal diseases and drugs/substances: Thyroid diseases, autoimmune diseases, epilepsy, head trauma; Alcohol, stimulants, steroids, certain prescription drugs, etc. may all trigger or aggravate symptoms.

Hormones and Sleep: Hormonal Fluctuations during Adolescence, Postpartum, and Menopause Long-term sleep deprivation or sleep rhythm disorder.

Psychological factors

Early attachment setbacks and traumatic experiences: neglect, abuse, school bullying, loss of a loved one, etc.

Personality traits and coping styles: Perfectionism, excessive self-blame, avoidant coping, low self-efficacy.

Cognitive biases and emotional regulation difficulties: such as catastrophic thinking, rumination, and poor impulse control.

Social and environmental factors

Long-term stress and major life events: high academic/work pressure, unemployment, divorce, heavy care burdens, financial difficulties.

Chronic adverse environments: poverty, discrimination, war/disaster, difficulties in migration and cultural adaptation, social isolation.

Family and interpersonal patterns: High conflict, excessive criticism or control, lack of support.

Modern environmental loads: Internet addiction, cyberbullying, information overload, prolonged sitting and lack of exercise, etc.

Induction-maintenance mechanism (common combination)

"Genetic susceptibility + long-term stress/acute trauma" can induce depression, anxiety and post-traumatic stress disorder.

"Sleep deprivation + substance use + biological susceptibility" can trigger manic or psychotic episodes.

Chronic pain/endocrine problems + negative interpretation can easily form a vicious cycle of anxiety and depression.

Protective and buffering factors (risk reduction)

Maintain a stable schedule, exercise regularly, have a balanced diet and get good sleep

Reliable social support (family, friends/peers/communities)

A safe growth environment and positive coping and problem-solving skills

Timely and accessible psychological counseling and psychiatric assessment

Warm reminder: If you or someone around you experiences significant depression/anxiety, loss of interest, insomnia or excessive sleepiness, a noticeable decline in attention, difficulty in performing daily functions, or signs of self-harm/suicidal thoughts, hallucinations or delusions that persist for more than two weeks, you should seek professional assistance as soon as possible. In case of emergency risk, please contact your local emergency/crisis hotline immediately.

**Q6Does insomnia or long-term staying up late increase the risk of mental illness?**

Short conclusion: Yes. Whether it is insomnia itself or long-term sleep deprivation (frequently staying up late and sleeping less than 6-7 hours), both are associated with an increased risk of subsequent depression, anxiety, substance abuse, and even psychotic experiences. The relationships are mostly prospective causal associations and are also related to sleep patterns and quality. (PubMed)

Key Evidence (Selected Excerpts

Systematic reviews/meta-analyses (prospective studies) show that some insomniacs later developed the disease

The odds ratio OR of depression is approximately 2.83.

Anxiety OR≈3.23

Substance abuse OR≈1.35;

Another included study suggested an increased risk of psychotic disorders (OR≈1.28). (PubMed)

Short sleep (generally less than 7 hours) is associated with an increased risk of depression in different populations (consistent among Asian and non-Asian people). (PMC)

Regarding the risk of "sleeping too much" : Some large-scale studies suggest that extremely long sleep (such as ≥10 hours) is also associated with a higher risk of major depression and generalized anxiety, but this consistency is poor across different studies (possibly reflecting that underlying diseases lead to "secondary long sleep"). (Nature)

Bipolar disorder is closely related to sleep: changes in sleep are often the precursor signals of emotional episodes (mania/depression), suggesting that sleep disorders and mood swings have predictive significance. (PubMed)

Long-term follow-up also found that chronic insomniacs have a higher risk of subsequently developing anxiety/depression, supporting the directionality of "insomnia → mood disorder", rather than just comorbidity. (PMC)

Possible mechanism (brief)

Neurotransmitters and stress axes: Insufficient sleep affects the 5-HT/ dopamine and hypothalamic-pituitary-adrenal (HPA) axes, increasing susceptibility.

Inflammation and circadian rhythm: Upregulation of inflammatory factors, circadian rhythm dislocation and social jet lag can all trigger or maintain emotional and cognitive symptoms.

(Overview of Review evidence). (PMC)

What can you do (key points based on evidence)

Prioritize insomnia itself: Cognitive behavioral therapy for insomnia (CBT-I) is the first-line option and its long-term effect is superior to that of sleeping pills alone. (Health)

Maintain a stable schedule: Try to keep it at 7 to 9 hours and keep a regular time for going to bed and getting up. Reduce blue light at night, caffeine and alcohol in the evening; Promote daytime light exposure and regular exercise. (PMC)

Seek medical attention promptly: If you experience difficulty falling asleep or maintaining sleep for at least 3 nights a week for at least 3 months, accompanied by daytime functional impairment, or if you have obvious emotional/anxiety symptoms or psychotic experiences (such as a strong sense of being victimized, auditory hallucinations), you should seek help from a psychiatrist or sleep specialist as soon as possible. (Health)

For those with bipolar disorder or a history of major depression: Regard changes in sleep as a warning sign of recurrence and work with a doctor to develop a "sleep-mood monitoring" and adjustment plan. (PubMed)

In a nutshell: Long-term insomnia and staying up late not only make people feel "mentally poor", but also significantly increase the risk of developing mental disorders such as depression and anxiety later on. Actively improving sleep and seeking standardized treatment can prevent or mitigate these risks to a certain extent. (PubMed)

**Q7How to determine if you may have a mental illness?**

The following guide can help you determine whether "I might have psychological or mental problems?" . It is not a diagnosis. When you have doubts, the safest thing to do is to talk to a professional as soon as possible.

I. First, conduct a self-check using four dimensions (simple yet practical)

Duration: Do these feelings/behaviors last for more than several weeks or recur repeatedly?

Degree of pain: Does it cause you obvious pain (anxiety, depression, irritability, etc.)?

Functional impairment: Does it affect sleep, study/work, interpersonal relationships or self-care? People with good mental health can usually cope with stress and exert their abilities. (World Health Organization)

Danger or reality test: Have there been suicidal/self-harming thoughts or plans, risks of harming others, hallucinations/delusions, extreme impulses, etc.? These belong to emergency warnings. (nimh.nih.gov)

Ii. Common signals of "May need help"

Emotions: Long-term and significant depression, emptiness, and irritability; Emotions fluctuate intensely.

Anxiety and physical and mental symptoms: Excessive worry, panic, avoidance; Significant changes in sleep/appetite and fatigue.

Cognition/Behavior: Inattentiveness, confusion; Social withdrawal There has been a significant decline in academic performance and work.

Perception/Reality: Delusions such as hearing things that are invisible, being victimized, or exaggerating.

All of these have been listed by authoritative institutions as signs that require attention. (nami.org)

Iii. Conduct a "preliminary screening" with reliable scales (still cannot be used as a diagnosis)

Depression/Anxiety: PHQ-9 (depression) and GAD-7 (anxiety) are commonly used self-rating scales in research and clinical practice, which can help determine the severity and whether further assessment is needed. (PMC)

Rapid comprehensive screening (commonly used in Taiwan) : BSRS-5 Mood Thermometer (5 questions +1 suicide additional question), which is convenient for assessing recent physical and mental distress. (Health 99 + website

Note: Scales can only indicate risks. A formal diagnosis requires a professional to conduct an interview assessment based on standards such as DSM-5-TR (if necessary, physical examinations/blood tests will be combined to rule out physical diseases). (psychiatry.org)

Iv. When should one seek medical attention or seek help as soon as possible?

Have suicidal or self-harming thoughts, plans or preparations;

Hallucinations/delusions, severe confusion or inability to take care of oneself;

Signs of mania (feeling energetic despite getting very little sleep, impulsive spending/sexual urges/risky behavior).

In such cases, please contact the emergency department or crisis resources immediately. For suicidal warning signs and assistance methods, please refer to NIMH. (nimh.nih.gov)

V. What to Do Next (Here's a simple process for you)

Conduct the above 4-dimensional self-check + a PHQ-9/GAD-7 or BSRS-5 copy.

If the score is high, symptoms persist for more than two weeks, or have already affected function, make an appointment with a psychiatrist or psychologist for an assessment.

At the same time, pay attention to the fundamentals: maintain a regular schedule, exercise, reduce alcohol/drug use, and keep socializing (these are often included in treatment recommendations, but they cannot replace professional assessment).

If you are taking medication or have a chronic disease, discuss with your family doctor or attending physician whether it is related to your mood (side effects of medication, thyroid/anemia, etc. may all affect your mood and concentration). (Mayo Clinic)

Vi. Immediate and Local Resources in Taiwan Region

The Ministry of Health and Welfare's Peace of Mind Hotline 1925 (Still Love Me) : 24-hour free psychological support and referrals when necessary.

Lifeline 1995, Teacher Zhang 1980: Providing Listening and Emotional Support.

Community mental health centers in each county and city can provide or refer psychological counseling and outpatient services. (mohw.gov.tw)

If you like, I can send you several reliable self-assessment questionnaire links right now and explain "what it represents and what to do next" based on your scores. If you have any thoughts of self-harm or harming others at this moment, please call 1925 directly or go to the nearest emergency room. Safety comes first. (dep.mohw.gov.tw)

**Q8Can self-assessment scales accurately determine whether one has a mental illness?**

Brief conclusion: No.

Most "self-assessment scales" (such as online quizzes, PHQ-9, GAD-7, etc.) are screening tools that can initially assess the "degree of possible risk/symptoms", but they cannot be used alone to confirm whether one has a certain mental illness. Whether there is a disease, what type it is, its severity and differential diagnosis, etc., must be determined by professionals through structured/semi-structured interviews, medical history and functional assessment. (apaservices.org)

Why does "self-testing" not equal "diagnosis"?

Tool positioning: The WHO and professional societies position such scales as "identification and triage" screening, used to indicate the population that needs further assessment, rather than providing a diagnosis. (World Health Organization

Limited accuracy and context-influenced: The sensitivity and specificity of the same scale may vary among different populations, languages, and cut-off scores, with false positives/false negatives. For example:

When the common cutoff score of the depression scale PHQ-9 is ≥10, the comprehensive sensitivity and specificity are approximately around 0.80, but there are significant differences among studies. The sensitivity of the calculation method for scoring is lower. (BMJ)

The pooled sensitivity of the Generalized Anxiety Scale GAD-7 in multiple studies was approximately 0.81, and the specificity was approximately 0.78 (with differences among different studies/languages). (ScienceDirect)

Self-reporting bias and comorbidities: Self-assessment is influenced by current emotions, social expectations, and differences in understanding the question. Moreover, common comorbidities (such as depression with anxiety, thyroid problems, substance use, bipolar disorder, etc.) can change the interpretation and require professional interviews and differentiation. Authoritative guidelines also emphasize that after identification, a comprehensive clinical assessment should be conducted to confirm the diagnosis and formulate a plan. (NICE)

Tips for the correct use of self-assessment scales

Treat it as a "thermometer" rather than a "diagnosis certificate" : A high score is a signal that "it needs to be evaluated", not a confirmed diagnosis. (apaservices.org)

Observe trends and functional impairment: If there are continuous changes in mood/sleep/appetite for ≥2 weeks, or if there are significant impacts on learning, work, and interpersonal relationships, seek medical attention promptly for assessment. Authoritative guidelines all recommend a systematic assessment and referral after a positive screening. (NCBI)

Give priority to using verified scales and have them interpreted by professionals (rather than random online tests) to reduce errors and unnecessary panic. (American Psychological Association

When should one seek help immediately (without waiting for self-testing)

If any of the following situations occur, please contact a professional or crisis resources as soon as possible: suicidal/self-harm thoughts and plans, hallucinations/feelings of being victimized, extreme excitement and sleep deprivation for several days, mental confusion, or people around you expressing concerns about your safety. (NCBI)

If you are in Taiwan: You can call the 24-hour Peace of Mind hotline 1925 (Still Love Me), or Lifeline 1995, or Teacher Zhang 1980 for immediate support when needed. (health.taichung.gov.tw)

**Q9Which department should one visit if they suspect they have a mental illness?**

The first choice of department for medical treatment is "Psychiatry" (also often referred to as "mind-body Department/Psychiatric Medicine Department").

Supplementary key points

For special groups such as children and adolescents, the elderly, and postpartum women, they can directly visit specialized clinics such as the Child and Adolescent Psychiatry Department, geriatric psychiatry department, and postpartum Emotional Clinic.

If accompanied by obvious neurological symptoms (such as repeated convulsions, persistent consciousness disorders, progressive memory decline, severe headaches, etc.), one can first visit the neurology department to rule out organic diseases, and then collaborate with the psychiatry department.

When experiencing acute or severe symptoms such as self-injury risk, hallucinations or delusions, extreme excitement or depression, or refusal to eat or drink, please go directly to the emergency department (psychiatric emergency department/general hospital emergency department) and try to be accompanied by a family member.

Before your visit, you can prepare

The time of symptom appearance and triggers, changes in sleep and appetite, past medical history and medication history, substance/alcohol use, family history of mental illness, etc. Bringing along the results of previous medical records is helpful for assessment.

Warm reminder: Psychological counseling is not the same as medical treatment. If mental illness is suspected, a psychiatric assessment should be the main approach, and psychological treatment should be referred if necessary.

If you are in crisis, please contact your local emergency number or psychological crisis intervention hotline immediately or go to the nearest emergency department.

**Q10How to distinguish mental illness from other physical illnesses?**

Here is a "clinically actionable" differentiation approach for you. The conclusion should be stated first: One cannot simply dichotomy based on the literal meaning of "spirit/body". Many systemic or neurological diseases start with changes in mood, behavior or cognition, and most mental disorders are essentially medical diseases of the brain. The key point of distinction lies in whether this is a "primary mental disorder" or a "mental symptom caused by organic/physical factors".

First, draw the judgment framework

Are there any "organic clues" (abnormal physical signs or examinations)?

Sudden onset and sharp fluctuations within a short period of time

Changes in consciousness level (drowsiness, poor orientation), delirium-like fluctuations

Fever, abnormal vital signs, and significant weight changes

Focal neurological signs (hemiplegia, speech/visual field defects, epilepsy)

Recent medication use/discontinuation, alcohol consumption and changes in substance use

If there are these clues: Prioritize screening for physical diseases or drug-related issues.

Whether it conforms to the typical spectrum of mental disorders (DSM-5/ICD-11 diagnostic dimensions)

Depression, bipolar disorder, anxiety, obsessive-compulsive disorder, schizophrenia spectrum, trauma-related, eating/sleeping/addiction, personality disorder, etc

Most cases have a progressive onset, with clear consciousness, normal vital signs, and mostly negative results in neurological examinations

Typical and persistent, with no abnormalities in physical examination/laboratory tests: tends to be primary mental disorder.

Ii. Commonly Used "Red Flag" and "Green Flag" in Clinical Practice

Red flag (indicating physical illness or drug/poison)

Acute onset (within hours to several days) or significant day-night fluctuations

Impaired consciousness/orientation: answering questions irrelevantly, fluctuating consciousness (delirium)

Visual or olfactory hallucinations are predominant, accompanied by severe headache, neck stiffness and convulsions

Fever, low/high blood pressure, tachycardia, hypoxia, hypoglycemia

The first appearance of mental symptoms in middle-aged and elderly people or neurological signs of any age

Drug-related: glucocorticoids, levodopa, anticholinergic drugs, stimulants, isotretinoin, etc

Substance/Alcohol: Poisoning or withdrawal

Green Flag (Biased towards primary mental disorders)

The disease progresses gradually from adolescence to young and middle-aged adults, with a course of several weeks or more

Clear consciousness, no abnormal vital signs, and negative neurological examination

There are similar mental disorders in the family

The symptoms are highly consistent with the classic spectrum (such as recurrent depression, typical obsessive thoughts + behaviors, etc.)

Iii. Examples of Confusing Situations

Thyroid diseases (hyperthyroidism → anxiety/insomnia/irritability; hypothyroidism → depression/cognitive decline

Autoimmune encephalitis (such as anti-NMDA receptor) : Acute mental symptoms in young people + epilepsy/autonomic nerve instability

Infection (syphilis, HIV, encephalitis), metabolism/endocrine (low sodium, low calcium, Cushing)

Vitamin deficiency (B12/ folic acid

Tumor/cerebrovascular disease/post-traumatic emotional and personality changes

Medications: Hormones, Parkinson's drugs, anti-tuberculosis drugs, and adverse reactions of certain antidepressants/anti-anxiety drugs

Substances: alcohol, sleeping pills, benzodiazepines, stimulants, marijuana, highly hallucinogenic substances

Iv. Concise Examination Procedure (Commonly Used in Outpatient and Emergency Departments)

Medical history and medication/substance history: Onset speed, diurnal fluctuation, history of infection/head trauma, family history, drugs and health supplements, alcohol consumption and medication.

Physical and neurological examination: body temperature, blood pressure, pulse, blood oxygen, pupil, meningeal irritation sign, focal signs.

Basic Laboratory (Try to do it when it is the first release or atypical)

CBC, liver and kidney function, electrolytes, blood glucose

TSH/ free T4, vitamin B12/ folic acid

Urine routine test + pregnancy test (for childbearing age), urine toxicity screening

ESR/CRP;" When necessary, syphilis, HIV, cortisol, ammonia, lactic acid

Further (when there is a red flag or abnormal physical examination results) : cranial imaging (CT/MRI), electroencephalogram, lumbar puncture, autoantibodies (suspected autoimmune encephalitis).

Standardized mental assessments (such as PHQ-9, GAD-7, YMRS, PANSS, etc.) were conducted simultaneously to quantify the severity of symptoms and follow-up.

V. How to Connect the Two

Distinguish delirium from psychotic disorders clearly: Delirium = acute, fluctuating, impaired attention/consciousness; Primary mental illness usually results in clear consciousness.

Remember the "VITAMINS" list of causes for exclusion: Vascular, Infection, Trauma, Autoimmune, Metabolic, Iatrogenic/Intox drugs/toxins, Neoplasm tumors, Seizure epilepsy.

Even if the diagnosis is primary mental disorder, comorbidities screening (sleep apnea, thyroid, anemia, drug interactions, etc.) is still necessary, as comorbidities are very common and can affect the therapeutic effect and safety.

Vi. When is it necessary to seek medical attention/emergency treatment as soon as possible

Suicidal or other harmful thoughts or behaviors, inability to take care of oneself

New mental symptoms accompanied by fever, headache, convulsions, progressive drowsiness or severe fluctuations

Mental symptoms occur during pregnancy or after childbirth

Significant behavioral and cognitive changes in the elderly in the short term

Vii. Practical Summary for You

First, list "organic/drugs/substances", and then consider the primary mental disorder.

Pay attention to four things: the onset speed, the state of consciousness, vital signs and neurological signs.

Conduct a set of basic laboratory tests, and perform low-threshold imaging or EEG for atypical/first-onset/middle-aged and elderly onset patients.

Typical cases with gradual onset, clear consciousness and negative test results are mostly primary mental disorders and should be transferred to the psychiatry department for standardized assessment and treatment.

**Q11Why do hospitals need to conduct multiple tests when diagnosing mental disorders?**

The hospital conducts "multiple tests" for the psychiatric department not to make a fuss over nothing, but to make the diagnosis more accurate and the medication safer. The core reasons are roughly as follows:

Exclude reversible "somatic causes"

Many internal medicine problems can disguise themselves as mental symptoms (such as thyroid dysfunction, anemia/vitamin B12 deficiency, infection, drug side effects, epilepsy, tumors, toxic substances from medicinal alcohol, etc.). Necessary physical examinations, laboratory tests and imaging can screen out such "pseudo-mental illness" and prevent misdiagnosis and mistreatment.

Mental disorders are highly "comorbid" and "heterogeneous"

Depression, anxiety, bipolar disorder, substance use disorder and personality disorder often overlap with each other. The manifestations of the same diagnosis also vary greatly among individuals. Multi-dimensional assessment (structured interviews, scales, family information, and past medical history) can more accurately classify types and severity.

Differential diagnosis requires a chain of evidence

Even for "low mood/hyperactivity/delusions", their causes may be completely different (depression vs...) Biphasic; Schizophrenia spectrum vs. organic psychosis. Multiple tests help establish/rule out hypotheses and form a reliable diagnosis.

Establishing a "baseline" facilitates follow-up and efficacy monitoring

The scale scores, laboratory indicators and adverse reaction monitoring (such as lithium salts, valproic acid, chlornitrogen, etc., which require blood drug concentration/blood routine/liver and kidney function) should all have baselines and dynamic comparisons to ensure effectiveness and safety.

Assess risks and functional impairments

Self-injury risk, reality-testing ability, and cognitive and social functions (work/study/family) determine the intensity and scenario of treatment (outpatient vs. Hospitalization, whether protective measures or rehabilitation support are needed.

Legal and ethical requirements

Major treatments (such as long-term medication, incapacity assessment, and legal appraisal) require traceable and verifiable assessment evidence to protect patients' rights and interests.

Common checks/evaluations that are often conducted include:

Systematic medical history collection and psychiatric specialist interviews (including family history, seizure rhythm, triggers, and substance use).

Standardized scales: such as PHQ-9/GAD-7, YMRS, PANSS, MOCA, etc. (select as needed).

Physical examination and basic laboratory tests: blood routine, biochemistry, thyroid function, B12/ folic acid, blood glucose and lipid, urine routine/toxicant screening; Sex hormones and infectious indicators when necessary.

Imaging/electroencephalogram: Head MRI/CT, EEG (when organic lesions, epilepsy, etc. are suspected).

Cognitive/neuropsychological tests: Differentiating dementia, attention/executive function disorders, developmental/learning problems.

Supporting information and long-term follow-up: Feedback from family members/colleagues, school/work records, symptom diaries/sleep records.

Psychiatric diagnosis relies on "comprehensive evidence". Multiple examinations are conducted to more accurately distinguish the cause of the disease, assess the severity and risk, and maximize the therapeutic effect and minimize the risk during the treatment process.

**Q12Is psychological assessment helpful for diagnosing mental illness?**

Brief conclusion: It is helpful, but a diagnosis cannot be made solely based on psychological assessment. Psychological assessment (such as scales, questionnaires, standardized personality and symptom scales, etc.) is of great value in discovering clues, evaluating severity and tracking therapeutic effects. However, psychiatric diagnosis still requires a comprehensive judgment by professionals based on systematic interviews, medical history and functional assessment. The American Psychiatric Association also explicitly emphasizes that these evaluations "can be used to enhance clinical decision-making and should not be the sole basis for clinical diagnosis."

What exactly can they do to help?

Screening and early identification: As the United States Preventive Services Task Force (USPSTF) recommends, depression screening for adults in primary care (commonly using PHQ-9/PHQ-2 as tools) should be conducted to bring potentially overlooked cases into further assessment and treatment processes. (uspreventiveservicestaskforce.org)

Quantifying severity and tracking changes: PHQ-9, GAD-7, etc. can provide baseline scores for comparison over time, helping to assess treatment response. (JAMA Network)

Improving diagnostic thinking and differentiation: Some comprehensive scales (such as MMPI-2/MMPI-2-RF) include validity scales, which can assist in identifying response patterns that exaggerate or mask symptoms, reducing the risk of misjudgment. (PMC)

A Glimpse of Evidence for Common Scales (with Examples) :

PHQ-9 depression screening: A meta-analysis of individual data (17,357 cases) showed that when the cut-off point was ≥10, compared with semi-structured clinical interviews, the sensitivity was approximately 0.88 and the specificity was approximately 0.85. This supports its use as a screening and severity assessment tool, but the diagnosis still needs to be confirmed by clinical assessment subsequently. (PubMed)

GAD-7 Generalized anxiety Screening: The original validation study shows that when the cut-off point is 10, the sensitivity is approximately 89% and the specificity is about 82%, making it suitable for clinical rapid screening and severity assessment. (JAMA Network)

But scales alone are not sufficient to make a diagnosis:

Diagnosis requires a comprehensive clinical assessment to confirm and distinguish overlapping symptoms related to bipolar disorder, PTSD, substance/physical facture-related states, etc. The NICE guidelines in the UK also require a complete assessment after screening prompts to confirm a diagnosis of depression. (NICE)

The scale can be influenced by cultural background, context, answering motivation and reaction bias. Even if tools with validity scales are used, they are only supplementary information. (PMC)

The optimal cut-off point and accuracy of the scale may vary depending on the sample and methodology. When interpreting, high-quality reviews or meta-analyses should be referred to, rather than choosing the best cut-off point from the same set of data in a "data-driven" manner. (JAMA Network)

A better clinical approach

1) First, conduct screening using short and reliable scales (such as PHQ-2→PHQ-9, or GAD-7). 2) If the screening is positive or there is clinical suspicion, conduct structured/semi-structured clinical interviews and functional evaluations to confirm the diagnosis. 3) Use the same scale to longitudinally track symptom changes and therapeutic effects. (uspreventiveservicestaskforce.org)

If you (or someone you care about) are experiencing obvious emotional or behavioral distress, the safest approach is to seek medical attention as soon as possible and use the results of the scale as the starting point rather than the conclusion of your discussion with a professional. If suicidal thoughts or behaviors occur, please contact local first aid and crisis support immediately.

**Q13If the diagnosis results of different psychiatrists vary, how should it be handled?**

Here is a practical handling list for you when you encounter inconsistent diagnoses from different psychiatrists. The core idea is to prioritize safety and the handling of current symptoms first, and then gradually clarify the diagnosis and long-term plan.

Let's start with the conclusion.

Psychiatric diagnoses often adjust over time and with the increase of information (for example, "provisional diagnosis/pending exclusion" is very common).

The focus is on the management of current symptoms and functional impairment, and the diagnosis is clarified in parallel.

The things to do immediately

Safety assessment: If you have suicidal thoughts or plans to harm others, severe hallucinations or delusions, manic impulses, withdrawal risks, etc., seek emergency help immediately or call the local emergency number. Don't be alone.

Don't stop taking the medicine by yourself first: When different doctors have different opinions, do not increase or decrease the dosage of the medicine by yourself or suddenly stop taking it. Please have one attending physician make the unified adjustments. If necessary, have a pharmacist conduct a compatibility review of the medication.

Systematically clarify the steps

Please have two (or more) physicians write down their respective bases

Ask the doctor to explain: the main evidence (interviews, scales, family observations), the timeline, the differentiating points from other diagnoses, and whether it is a comorbidity rather than mutually exclusive.

Ask whether different classifications of DSM/ICD are used, whether it is an "unspecified" or "provisional" diagnosis, and whether a differential diagnosis list (rule out) is set.

Sign the Information Agreement (ROI) and facilitate communication

Authorize different physicians to communicate with each other, or invite family physicians/case managers/psychologists to hold case conferences to reduce the information deviation of "each reading a section".

Prepare a standard document package

Symptom timeline (initial onset, exacerbation, triggers), previous diagnosis and treatment responses, medications and side effects, substance/alcohol/caffeine use, sleep schedule, major life events, family history.

Attached are the scales (such as PHQ-9, GAD-7, YMRS, PANSS, etc. selected by the physician) and functional indicators (academic/work/interpersonal).

Bring previous tests (thyroid, B12/ folic acid, liver and kidney function, urine toxicity screening, and brain and sleep assessment if necessary) to rule out somatic and drug-induced factors first.

Consider cultural and situational factors

Physicians can be invited to use culture-defined interviews (CFI) to discuss the impact of cultural expression, stressors, migration/language factors on symptoms.

Agree to conduct "longitudinal observation"

Agree on a follow-up and quantitative tracking of 6 to 12 weeks; Many diagnoses (such as bipolar disorder vs. major depressive disorder, psychoaffective disorder vs. schizophrenia) require observation of the seizure patterns and cycles before a conclusion can be made.

The balance at the therapeutic level

Separate the "diagnostic label" from the "treatment goal" : First, clearly define 3 to 5 short-term goals (sleep more than 6 hours, decrease in panic attacks, return to work/post, and reduce suicide risk).

Cross-disciplinary integration: Medication + psychotherapy (CBT, MBCT, family/interpersonal/insomnia treatment, etc.) + rehabilitation and lifestyle prescriptions (sleep schedule, exercise, substance reduction).

Key points of experimental treatment: Any drug or treatment trial should have a goal, schedule, assessment indicators and review points (for example, a reduction of ≥5 points in PHQ-9 after 4 weeks is considered effective).

When is it necessary to seek a second or third opinion

The diagnosis involves long-term medication (such as mood stabilizers, antipsychotic drugs) or legal/insurance/job suitability.

Different opinions lead to conflicts in treatment directions (for example, "Whether antidepressants need to be combined with emotional stabilizers").

Symptoms are disproportionate to side effects, treatment efficacy is poor or worsens.

Common "points of disagreement" cheat sheets

Severe depression vs. Bipolar disorder: Has there ever been a clear manic/hypomanic period? Family history Antidepressants induce excitement.

Schizophrenia spectrum vs. mood-related psychotic disorders: Synchrony/asynchrony of psychotic symptoms and mood episodes.

Obsessive-compulsive Disorder vs. Psychotic Disorders: Awareness and Resistance of Obsessive Thoughts.

Personality traits and trauma responses: Long-term stable adaptation patterns vs. Clearly triggered stress-symptom chains.

Caused by substance/physical illness: Time correlation with laboratory/toxicological screening.

Practical tools and suggestions

Use a symptom/sleep/mood diary or an App for daily scoring; Third-party observation is provided by family members or co-residents.

Select one attending physician as the "general coordinator" and the others as "advisors" to avoid duplicate prescriptions and conflicts of instructions.

Keep medical records and prescription lists, and carry them with you when seeking medical treatment outside.

**Q14Will visiting a psychiatrist be regarded as having a mental illness?**

No.

"Seeing a psychiatrist" does not equal "being diagnosed with mental illness". Seeking medical treatment is merely for assessment and assistance, just like seeing an internal medicine doctor, it is a medical procedure.

The commonly known "mental illness" mostly refers to diseases with psychotic symptoms (such as delusions and hallucinations). Psychiatry covers a wide range of issues, including anxiety, depression, insomnia, adjustment disorders, attention difficulties, addiction, emotional and interpersonal problems, etc. The vast majority of patients do not have psychotic disorders.

Whether there is a diagnosis or not needs to be determined by the doctor based on the standards and assessment results. It will not be automatically labeled as a diagnosis just because of registration.

Medical records are protected by privacy and medical ethics norms and, in principle, will not be disclosed to the public at will. It will only be shared in a few circumstances where you consent or as required by law and safety (such as when it seriously endangers your own or others' safety).

Seeking help early usually helps relieve symptoms more quickly and prevent deterioration. This, like seeing other departments, is a responsible form of self-care.

If you are concerned about a specific situation (such as job hunting, insurance, driving license or communication with the school), tell me your country/region and your concerns. Then I can explain the general practices and feasible protective measures more specifically.

**Q15What are the main methods for treating mental illness?**

The following explains the main methods of "treating mental illness" in plain but as accurate as possible by category (the combination of different diseases and the severity of the condition may vary, and it is usually carried out in a multidisciplinary and step-by-step manner (stepped-care)) :

Psychotherapy (talk therapy

Common schools of thought: Cognitive Behavioral Therapy (CBT), interpersonal psychotherapy (IPT), psychodynamic therapy, exposure and response prevention (OCD), family therapy, etc. A large number of guidelines consider evidence-based psychotherapy as a first-line treatment or a core component for anxiety, depression and other conditions. (nimh.nih.gov)

Drug therapy

Main drug categories: Antidepressants (such as SSRI/SNRI), anti-anxiety drugs/sedative-hypnotics, antipsychotic drugs (first/second generation), mood stabilizers (such as lithium salts, valproic acid, etc.), stimulants (ADHD). Whether to use medication and how to combine them need to be evaluated by a psychiatrist, and the adverse reactions and therapeutic effects should be followed up. (nimh.nih.gov)

Brain stimulation therapy (suitable for those with poor response to drug/psychological treatment or severe conditions)

ECT (Electroconvulsive Therapy) : It has definite therapeutic effects on severe depression, refractory or emergency dangerous conditions (such as catatonia, high risk of suicide, etc.). It should be carried out under anesthesia in a professional team and the risks and benefits should be fully informed. (Mayo Clinic)

rTMS (repetitive transcranial magnetic stimulation) : It has evidence-based support for moderate to severe depression and is one of the options for treatment-resistant depression. It is generally a non-invasive outpatient treatment. (nimh.nih.gov)

Others: tDCS and others are still under exploration and are usually used as auxiliary means. Evidence is accumulating. (The Guardian

Psychosocial and rehabilitation therapy

Psychological education, family and caregiver support, social skills training, vocational and academic support, case management, comprehensive intervention for substance use disorders, etc. It is particularly important in the long-term rehabilitation of the schizophrenia spectrum and severe mental disorders. (World Health Organization

Lifestyle and self-management (often as an adjunct to standard treatment)

Regular schedules and exercise, sleep management, stress reduction and mindfulness practice, nutrition and substance use management, digital intervention (such as compliant online CBT programs), etc. It is usually combined with professional follow-up. (nimh.nih.gov)

Crisis intervention and hospitalization

When there is a risk of self-injury or external injury, severe functional impairment, or the need for rapid and effective intervention, short-term hospitalization and crisis management can ensure safety and close monitoring, and quickly adjust the plan. After that, outpatient services and community support are usually continued. The overall approach is consistent with the guidelines, emphasizing safety and continuous care. (World Health Organization

The combination of "step-by-step treatment" and individualization

Most guidelines advocate that, based on the severity of the condition and the response, the "most effective and least invasive" measures should be adopted first, and then upgraded and combined when necessary (for example, "psychotherapy + medication" is often superior to single therapy). (NICE)

Warm reminder

The most appropriate treatment requires professional assessment and follow-up. Do not stop taking the medicine or change the prescription by yourself. In the event of an acute crisis (such as suicidal thoughts or plans), please contact your local emergency number or crisis hotline immediately, or go to the nearest emergency department for medical treatment.

**Q16Which is more effective, psychiatric medication or psychotherapy?**

Brief conclusion

Which one is more effective depends on the specific disease and its severity. The general rule is that in most cases, the combination of medication and psychotherapy works best. For some diseases, psychological treatment is given priority, while for others, medication is the basis and psychological treatment is used for reinforcement and consolidation.

How to choose: By disease type

Depression (Adult)

For moderate to severe depression, combined treatment (medication + psychotherapy, such as CBT) is usually superior to single treatment in alleviating symptoms and preventing recurrence. The guide also lists both as main options. (PubMed)

Anxiety disorders (social anxiety, generalized anxiety, panic, etc.)

The long-term effects of psychological treatments such as CBT are reliable, and they are less likely to rebound after discontinuation. Drugs (such as SSRIs/SNRIs) are also effective. A common practice is to choose the first option and decide whether to combine them based on the reaction. (PubMed)

Post-traumatic Stress Disorder (PTSD

Authoritative guidelines list trauma-focused psychotherapy (such as PE, CPT, EMDR, etc.) as a first-line treatment. Medication is usually an option when one is unwilling or unsuitable for psychotherapy or when residual symptoms still exist. (ptsd.va.gov)

Obsessive-compulsive Disorder (OCD

ERP (Exposure and Response Prevention) is the preferred psychotherapy. Drugs such as SSRIs and clomipramide are also effective. Severe functional impairment is often recommended as "medication +ERP". (PMC)

Schizophrenia/Psychotic disorder

Antipsychotic drugs are the cornerstone of treatment. On this basis, family intervention, CBT, rehabilitation and other psychosocial treatments can improve the recurrence rate, function and compliance. (NICE)

Bipolar disorder

Based on mood stabilizers/antipsychotic drugs; Incorporating psychotherapy (such as family-focused therapy, psychological education, IPT/CBT, etc.) can reduce recurrence and enhance function. (PMC)

Key points of practical decision-making

Objective: Alleviate current symptoms vs. prevent recurrence. Combination or sequential (medication first and then psychology/vice versa) can help reduce the risk of recurrence. (JAMA Network)

Side effects and availability: The drug may cause adverse reactions; Psychotherapy requires accessibility and time investment. Research on depression shows that the acceptability of psychotherapy and combination therapy is often superior to that of medication alone. (PubMed)

Personal preferences and past responses: Many guidelines encourage co-decision-making based on patient preferences, the severity of the condition, and comorbidities. (NICE)

A one-sentence summary

If it is PTSD, OCD, or some anxiety disorders: Prioritize or strongly consider psychotherapy (add medication if necessary). (ptsd.va.gov)

If it is moderate to severe depression, bipolar disorder, or schizophrenia: Medication is key, and combined with psychotherapy, it usually leads to better short-term and long-term outcomes. (PubMed)

**Q17Is exercise effective for mental illness?**

Brief conclusion: Effective, but usually most suitable as an "adjunctive treatment". The evidence for depression and anxiety is the strongest. It also has certain benefits for negative symptoms of schizophrenia, ADHD (Attention Deficit Hyperactivity Disorder), and PTSD. The depressive phase of bipolar disorder may be helpful, but the risk of triggering a manic phase should be avoided. Here is a clear and straightforward outline for you:

Depression: A large number of systematic reviews/meta-analyses have shown that exercise can significantly alleviate depressive symptoms, and the effect of higher-intensity exercise is even greater. Many clinical guidelines list exercise as one of the treatment options (especially for mild to moderate cases). (BMJ)

Anxiety and psychological distress: Among different groups of people, regular physical activity can moderately reduce anxiety and psychological stress. (PubMed)

Schizophrenia: There is evidence that aerobic, resistance training or yoga can slightly to moderately improve negative symptoms and depression/function-related outcomes. (Nature)

ADHD: Among children and adolescents, physical activity (especially aerobic or "high cognitive engagement" activities) can improve attention, executive function and core symptoms. (PubMed)

PTSD: As a bonus treatment, physical activity (including yoga) can improve PTSD symptoms, anxiety, depression and sleep, and the evidence is gradually increasing. (PubMed)

How much to do and how to do it?

The universal "adequate dose" provided by the World Health Organization:

150 to 300 minutes of moderate-intensity training (such as brisk walking or cycling) or 75 to 150 minutes of high-intensity training (such as jogging or interval running) per week, plus at least two strength training sessions per week. Starting from scratch is also beneficial. Proceeding step by step is the most important. (NCBI)

Here's a tip for getting faster results from depression or anxiety: Increase the intensity to a level where you feel a bit out of breath but can still speak short sentences (i.e., medium-high intensity). A slightly higher intensity within a feasible range often yields greater results. Group or outdoor activities can help with persistence. (BMJ)

Safety and Attention

It is by no means a substitute for medication or psychotherapy. Especially when suffering from moderate to severe depression, suicide risk, or psychotic symptoms, please use exercise as an adjunct under the guidance of a doctor. The NICE guidelines clearly list exercise as one of the treatment options, but it requires comprehensive management and follow-up. (NICE)

Bipolar disorder: Prioritize regular exercise during stable or depressive periods. Avoid high-intensity sleep and sleep disruption at night (to prevent triggering manic signs). The intensity and time period can be determined together with the doctor. (PMC)

Start with low intensity and short duration (for example, 10 to 15 minutes of walking each time), and increase by 10 to 20% per week. If any discomfort or worsening of symptoms occurs, stop immediately and communicate with a professional. (NCBI)

An easy-to-implement introductory solution (example)

Three times a week: 30 to 40 minutes of brisk walking/jogging/cycling (moderate to high intensity).

Twice a week: Full-body strength training (bodyweight squats, push-ups, resistance band rowing, etc., 10-12 times × 2-3 sets).

Daily: 5 to 10 minutes of flexibility/breathing exercises or short yoga flows for mood regulation and sleep.

The above combination is consistent with the current evidence, covering aerobic + resistance + psychosomatic three categories, and is beneficial to a variety of mental symptoms. (PubMed)

**Q18What types of drugs are commonly used to treat mental disorders?**

The commonly used drugs for treating mental disorders can mainly be classified into the following categories, and each category of drugs has specific therapeutic effects for different types of mental disorders

Antidepressants (antidepressants) :

Common drugs: Selective serotonin reuptake inhibitors (SSRIs), such as fluoxetine and sertraline, as well as tricyclic antidepressants (Tcas), such as amitriptyline, and monoamine oxidase inhibitors (MAOIs), such as phenylhydrazine.

Indications: It is used for the treatment of depression, anxiety, obsessive-compulsive disorder (OCD), etc.

Antipsychotic drugs (antipsychotic drugs) :

Common drugs: First-generation antipsychotic drugs (typical antipsychotic drugs), such as chlorpromazine and Haldol; Second-generation antipsychotic drugs (atypical antipsychotic drugs), such as olanzapine, risperidone, and clozapine.

Indications: Mainly used for the treatment of severe mental disorders such as schizophrenia, mania, and bipolar disorder.

Mood stabilizer

Common drugs: Lithium salts (such as lithium carbonate), sodium valproate, lamotrigine.

Indications: It is used for the treatment of bipolar disorder, especially for controlling manic episodes and preventing mood swings.

Anti-anxiety drugs

Common drugs: Benzodiazepines, such as diazepam, lorazepam, and alprazolam.

Indications: It is used to relieve symptoms such as anxiety disorders, phobias, and acute anxiety attacks.

Sedative-hypnotic drugs

Common medications: Zaleplon, zolpidem, etc.

Indications: It is used for the treatment of insomnia and other sleep disorders.

Medications for cognitive impairment

Common drugs: donepezil, memantine.

Indications: It is used for the treatment of Alzheimer's disease (senile dementia).

These medications can help relieve symptoms or control the progression of mental illness, but they usually need to be used under the guidance of a doctor to avoid side effects or drug interactions. In addition, drug therapy is often combined with other treatment methods such as psychotherapy (such as cognitive behavioral therapy) to achieve better therapeutic effects.

**Q19How long does it usually take for psychiatric drugs to take effect?**

The time it takes for psychiatric drugs to take effect varies depending on the type of drug, the specific condition of the patient and the severity of the illness. Generally speaking, different types of psychiatric drugs have different onset times:

Antidepressants: Most antidepressants, such as SSRIs (Selective serotonin reuptake inhibitors) and SNRIs (norepinephrine and serotonin reuptake inhibitors), usually take 2 to 4 weeks to show significant effects. Some patients may experience slight improvement within 1 to 2 weeks, but it usually takes 4 to 6 weeks to see significant improvement.

Anti-anxiety drugs: Benzodiazepines (such as diazepam and lorazepam) are often used to relieve anxiety symptoms in the short term and usually take effect within a few hours after administration. However, when SSRI drugs are used to treat anxiety disorders, it also takes 2 to 4 weeks to see the effect.

Antipsychotic drugs: For antipsychotic drugs used to treat schizophrenia or other severe mental disorders, some may improve acute symptoms within a few days, but the full effect may take several weeks to months to manifest. The new generation of antipsychotic drugs (such as risperidone and olanzapine) usually start to show efficacy within 2 to 4 weeks.

Mood stabilizers: For instance, lithium salts used to treat bipolar disorder may bring about improvement within a few days to a week, but it may take longer (several weeks) to fully stabilize the mood.

It should be noted that there are significant individual differences. Some patients may feel the effect of the drug more quickly, while others may need a longer time. At the same time, the side effects of drugs and dosage adjustments may also affect the treatment outcome. If the effect of the drug is not obvious or the side effects are significant, the doctor may consider adjusting the drug or treatment plan. Therefore, it is recommended to communicate with a psychiatrist regularly to monitor the efficacy and adverse reactions of the medication.

**Q20Will taking psychiatric drugs damage brain function?**

Whether taking psychiatric drugs will damage brain function depends on the type of drug, the dosage taken, the duration of use and the individual's response. Overall, psychiatric drugs are usually used to treat various mental health problems, such as depression, anxiety disorders, schizophrenia, etc., and these drugs are sometimes very necessary. The main function of psychiatric drugs is to improve functions such as mood, cognition and behavior by regulating neurotransmitters in the brain.

The types and effects of psychiatric drugs

Antidepressants (SSRIs, SNRIs, etc.)

The goal of this type of drug is to alleviate depressive symptoms by increasing the levels of neurotransmitters such as serotonin and norepinephrine in the brain.

Long-term use may cause some side effects, such as weight gain and sexual dysfunction, but it usually does not lead to serious nerve damage.

Antipsychotic drugs (such as typical and atypical antipsychotic drugs) :

This type of drug is used to treat schizophrenia and other serious mental disorders. Atypical antipsychotic drugs are generally considered to have fewer side effects, but some of them may cause movement disorders, metabolic problems (such as an increased risk of diabetes), etc.

Long-term use sometimes leads to what is called tardive dyskinesia, a drug-related motor control problem that may affect certain brain functions, but this situation is relatively rare.

Mood stabilizers (such as lithium salts, antiepileptic drugs, etc.) :

These drugs are used to treat mood disorders such as bipolar disorder. The side effects of long-term use include kidney function problems and weight gain.

Although these medications can help stabilize emotions, if taken in excessive amounts or not monitored regularly, they may also have adverse effects on the body.

Sleeping pills and anti-anxiety drugs (such as benzodiazepines) :

Sleeping pills and anti-anxiety drugs are effective in helping to relieve anxiety and insomnia in the short term, but long-term or excessive use may lead to dependence, memory problems and cognitive decline.

The influence of brain function

Most side effects of psychiatric drugs do not directly cause permanent brain damage, but some drugs may affect cognitive function or emotional stability during long-term use. For instance, certain medications may cause mild memory loss, inattention or emotional numbness, etc. For people who have been taking medication for a long time, especially those who do not regularly assess their drug use, some cognitive or emotional side effects may occur.

Conclusion

When used correctly, most psychiatric drugs do not cause serious damage to the brain. The use of drugs should be carried out under the guidance of a doctor, and it is very important to regularly assess the efficacy and side effects of the drugs. If there are any side effects or concerns, you should communicate with your doctor in a timely manner. Proper management of drug use can minimize possible negative impacts to the greatest extent.

**Q21How to deal with the side effects of psychiatric drugs, such as weight gain and drowsiness?**

Psychiatric drugs, especially antidepressants and antipsychotics, often cause some side effects, such as weight gain (obesity) and drowsiness. To better deal with these side effects, you can try the following methods:

1. Weight gain (getting fat)

Dietary control: Maintain a healthy and balanced diet and try to avoid foods that are high in calories and sugar. Increasing the intake of high-fiber foods (such as vegetables, whole grains, beans, etc.) can help control weight.

Increase physical activity: Regular aerobic exercises (such as walking, running, swimming or cycling, etc.) can help burn off excess calories. Exercise not only helps control weight but also improves mood.

Monitor weight: Regularly measure your weight and observe the impact of medication on it. If your weight increases excessively, discuss possible medication adjustments with your doctor in a timely manner.

Medication adjustment: Certain medications (such as some antipsychotic drugs) can affect appetite or metabolism. If there is a significant increase in weight, consider discussing with your doctor whether you can adjust your medication or switch to one with fewer side effects.

2. Excessive sleepiness

Adjust the time of taking medication: Some medications can cause drowsiness, especially when taken during the day. If possible, you can consider taking the medicine at night or adjust the dosage and time of taking it according to the doctor's advice.

Dispersed dosage: Some medications can be taken in multiple doses (such as once in the morning and once in the evening), which can help reduce daytime drowsiness.

Improving sleep quality: Maintaining a regular schedule and creating a quiet and comfortable sleeping environment can help enhance sleep quality and alleviate daytime sleepiness.

Avoid interactions with other sleepiness medications: If you are taking other medications (such as sleeping pills, anti-anxiety drugs, etc.), be aware that they may aggravate the side effects of sleepiness. Discuss the combined use of drugs with your doctor to see if there is a better option.

3. Communicate with the doctor

Regular follow-up visits: The side effects of medication may change over time, so it is necessary to communicate with a psychiatrist regularly and adjust the medication as needed.

Inform the severity of side effects: If side effects affect your daily life or health, be sure to inform your doctor. The doctor may adjust the type, dosage of the medication or switch to other drugs based on the situation.

Overall, when treating mental health issues, medication is an important part, but side effects should not be ignored either. Making appropriate lifestyle adjustments and following the doctor's guidance can help reduce the impact of side effects and maintain good physical and mental health.

**Q22Can taking psychiatric drugs lead to addiction?**

Psychiatric drugs (such as antidepressants, anti-anxiety drugs, antipsychotic drugs, etc.) do not necessarily lead to addiction, but this depends on the type of drug and the individual's response.

Antidepressants (such as SSris, SNRis, etc.) : These drugs usually do not cause physical dependence or addiction. They improve mood by regulating chemicals in the brain, but they do not cause strong dependence like some substances (such as opioids or sedatives). However, suddenly stopping the medication or reducing the dosage may cause some uncomfortable symptoms, which is known as "drug withdrawal syndrome".

Anti-anxiety drugs (such as benzodiazepines) : These drugs (for example, diazepam, alprazolam, etc.) may cause dependence or addiction, especially when used for a long time or in high doses. They mainly relieve anxiety through sedative effects, but if dependence develops, withdrawal symptoms may occur when the medication is stopped. Therefore, such drugs are generally recommended for short-term use and the dosage should be gradually reduced under the guidance of a doctor.

Antipsychotic drugs: These drugs are generally not addictive, but long-term use may lead to side effects (such as weight gain, metabolic abnormalities, etc.). Some antipsychotic drugs may affect the dopamine system in the brain, but they usually do not cause typical addictive behaviors.

In conclusion, most psychiatric drugs, when used reasonably under the guidance of a doctor, will not lead to addiction. However, certain drugs (especially benzodiazepines) may cause dependence when abused or improperly used. When taking medication, it is very important to follow the doctor's advice, have regular follow-up visits, and avoid stopping the medication or changing the dosage on your own. If you are concerned about drug dependence, it is recommended that you discuss alternative therapies or other treatment methods with your doctor.

**Q23. What is electroconvulsive therapy without convulsions?**

Electroconvulsive Therapy (ECT) is a mental treatment method, mainly used to treat some serious mental disorders, especially depression, bipolar disorder, schizophrenia, etc. When patients do not respond to other treatment methods, such as drug therapy, ECT may become an effective option.

Unlike traditional electroconvulsive therapy, the most distinctive feature of non-convulsive electroconvulsive therapy is that during the treatment, the current passing through the brain does not cause obvious body convulsions, which helps to reduce the impact on the patient's body. Moreover, it is usually carried out with the assistance of anesthesia and muscle relaxants, so the patient will not feel pain during the treatment.

Process

Anesthesia: The patient will first receive general anesthesia to ensure comfort and safety during the treatment process.

Electrical conduction: Through specially designed electrodes, brief electrical signals are sent to the patient's brain. The current can generate a type of "epileptiform" brain electrical activity, helping to regulate the balance of neurotransmitters in the brain.

Monitoring and care: After treatment, the patient will be sent back to the recovery area for observation until the anesthetic effect wears off to ensure there are no complications.

Indications

Electroconvulsive therapy without convulsions is usually used in the following situations:

Major depressive disorder (especially depression with suicidal tendencies)

An acute episode of bipolar disorder

Severe mental disorders such as schizophrenia

Mental disorders that do not respond to other drug treatments

Safety and side effects

Although ECT is a relatively safe treatment method, it may also cause some side effects, the most common of which is short-term memory loss, especially recent memory related to the treatment. Other potential side effects include headache, muscle aches, irregular heart rate, etc.

Overall, electroconvulsive therapy without convulsions is regarded as an effective and relatively safe treatment method, especially suitable for patients who have not responded to other treatments.

**Q**24Which diseases is transcranial magnetic stimulation therapy mainly applicable to?

The following answers are provided in two categories: "Clearly recognized by authoritative institutions/routinely used" and "Evidence growing (mostly non-indications/needs to be conducted in professional centers)" - to help you grasp the clinical certainty and accessibility

1. Has been explicitly recognized by an authoritative institution (there may be slight differences in the country/region)

Major depressive disorder (especially refractory) : As a non-invasive option after the ineffectiveness of medication and psychotherapy, it has entered the guidelines and routine practices of many countries. The NICE in England has long provided guidance on intervention procedures. Multiple system evidences support its security and effectiveness. (NICE)

Obsessive-compulsive disorder (OCD) : The U.S. FDA has approved specific devices/parameters for the adjuvant treatment of adult OCD. (FDA Access Data)

Migraine: The FDA approved single-pulse TMS (sTMS) early on for the acute treatment of migraine with aura. Some devices are also used for prevention and are specific equipment/parameters. (PMC)

Quitting smoking (smoking addiction) : The FDA has approved deep TMS as an adjunct to short-term smoking cessation in adults. (FDA Access Data)

The above-mentioned several items have also been summarized by authoritative medical resources for the general public as the main approved uses of TMS. (Mayo Clinic)

Ii. Evidence Growth/Carried out in professional centers (mostly "non-indications")

Auditory hallucinations in schizophrenia (AVH) : NICE has issued procedural guidance on "TMS for Auditory Hallucinations"; Recent randomized controlled trials have also shown that image-guided rTMS can reduce the intensity and frequency of AVH. (NICE)

Neuropathic pain: Multidisciplinary guidelines and reviews suggest that applying high-frequency rTMS to the motor cortex can bring about short-term analgesic effects, but the maintenance and population heterogeneity are still under study. (e-acn.org)

Post-stroke motor/language function rehabilitation, Parkinson's disease symptoms, tinnitus, post-traumatic stress disorder (PTSD), generalized anxiety, bipolar depression, etc. : There have been varying degrees of research and small-sample trials, but overall, they are still in the exploration stage and require individualized assessment and standardized plans. Reviews and position papers generally emphasize that "it may be effective, but the quality and reproducibility of the evidence vary." (ScienceDirect)

Summary (Key Points for Medical Decision-making)

"Mainstream and safe indications" : treatment-resistant depression, OCD, migraine (specific conditions/equipment), smoking cessation assistance. If you are seeking medical treatment for these issues, TMS is worth discussing seriously with your doctor. (NICE)

Situations where "transfer to a specialized center can be considered" include auditory hallucinations, intractable neuropathic pain, etc., which require an experience team and individualized navigation, parameter optimization, and it should be known that the therapeutic effects are mostly short-term and need to be maintained. (NICE)

The scope of application and contraindications (such as uncontrolled epilepsy, intracranial/cranial metal implants, etc.) should be verified by a qualified physician during the face-to-face consultation. The indications for different devices and coil types (such as deep TMS, single-pulse/repetitive TMS) strictly depend on their approved parameters and cannot be simply inferred. (www.rcpsych.ac.uk)

**Q25Can one stop taking psychiatric medication on their own after symptoms improve?**

Brief conclusion: Do not stop taking psychiatric medications on your own after your symptoms have improved. Whether to stop taking the medicine and how to do it must be carried out in conjunction with the doctor to formulate a "tapering" plan. Many medications need to be maintained for a period of time. Stopping the medication rashly may bring the risk of recurrence or withdrawal reactions. (NICE)

Why can't it stop by itself?

The risk of recurrence is high. The recurrence rate of diseases such as schizophrenia increases significantly after the discontinuation of antipsychotic drugs. Therefore, guidelines usually recommend maintenance treatment and close monitoring rather than reducing or discontinuing treatment without authorization. (Access Behavioral Health)

There is a syndrome of drug withdrawal or withdrawal. Stopping antidepressants too quickly can cause discomfort such as dizziness, insomnia, flu-like symptoms, and increased anxiety. Evidence-based guidelines clearly recommend "phased and gradual" reduction. (NICE)

It is particularly dangerous to stop certain medications immediately. Rapid discontinuation of lithium salts leads to a faster and more common recurrence than slow discontinuation, and there may also be a situation where the reaction deteriorates after re-administration. Benzodiazepines (diazepam) must not be stopped suddenly. The dosage should be reduced very slowly under professional supervision. (SpringerOpen)

The general principles of different drugs (only for direction, individualized) :

Antidepressants: Usually, the dosage is gradually reduced from week to month (some people may need a longer period) to minimize withdrawal reactions. The entire process is jointly determined by the doctor and the patient in terms of speed and duration. (NICE)

Antipsychotic drugs: In most cases, long-term maintenance is required, and it is not recommended to reduce the dosage on your own. Any adjustments should be made after the doctor has assessed the risk of recurrence. (Access Behavioral Health)

Mood stabilizers (such as lithium salts) : The dosage must be reduced extremely slowly; Rapid drug withdrawal is associated with a higher and earlier recurrence rate. (SpringerOpen)

Benzodiazepines (such as lorazepam, diazepam, etc.) : Sudden stop is strictly prohibited. The latest multidisciplinary guidelines recommend a reduction of 5% to 10% every 2 to 4 weeks, with a slower pace if necessary, and under clinical supervision. (ACMT)

How to safely discontinue medication with a doctor (General Steps) :

Explain your improvement and concerns, and make an appointment to discuss whether it's time for the "maintenance period to end and you can consider reducing or stopping the treatment". (NICE)

Develop an individualized gradual reduction plan (reduction range, interval, follow-up frequency, and response to discomfort). (NICE)

Identify withdrawal reactions vs. relapse: If core symptoms (such as low mood/anhedonia, auditory hallucinations, severe anxiety or insomnia, etc.) persist and worsen, return for a follow-up visit promptly. If necessary, slow down or reduce the dosage. (nswmentalhealthcommission.com.au)

In what situations should one "contact a doctor or the emergency department immediately"?

High fever, muscle rigidity and changes in consciousness (suspected malignant syndrome), severe rash/allergy, obvious suicidal thoughts or behaviors, significant reduction in white blood cells indicating infection signs, severe withdrawal (convulsions, etc.). These are common warnings of adverse reactions to emergency medication

Once it occurs, an urgent assessment is needed to determine whether to adjust or discontinue and handle it.

What should I do if it has stopped by itself?

Please contact the doctor who prescribed the medicine as soon as possible. Do not repeatedly increase or decrease the dosage or "make up the dose at one time" by yourself.

Record the discomfort that occurs and the timeline (what symptoms appear on which day the medication stops), and take it to the doctor for a check-up to facilitate the determination of whether it is a reaction to drug withdrawal or a recurrence.

The doctor may recommend resuming the previous effective dose before attempting to reduce it more slowly. (NICE)

**Q26How long should psychiatric medication continue to be taken after the condition stabilizes?**

Here is a general reference for you on "How long do you need to take common psychiatric medications after your condition stabilizes?" (please refer to the assessment of your clinic doctor for the exact duration)

Major depressive disorder (antidepressants)

First episode, complete remission: Usually lasts for at least 6 to 12 months. (NICE)

Higher risk of recurrence (≥2 episodes, residual symptoms, combined suicide risk, etc.) : It is recommended to maintain for ≥2 years, and some people may need longer maintenance treatment. (gpnotebook.com)

Anxiety disorder/Panic disorder (mostly SSRI/SNRI)

After the symptoms have eased, it is usually advisable to continue for 6 to 12 months before evaluating whether to reduce the medication. (American Association of Family Physicians

Obsessive-compulsive disorder (mainly SSRI)

After being effective, it should be maintained for at least 12 months and continued for a period of time after remission to prevent recurrence. (NICE)

Schizophrenia/First psychotic episode (antipsychotic drugs)

After the first episode subsides: Most societies recommend maintaining for at least about one year.

Repeated episodes or high risk: It often requires maintenance for 2 to 5 years or even longer, and some patients need long-term/continuous treatment. (Psychopharmacology Institute)

The APA 2021 guidelines also emphasize the importance of maintenance medication and individualized assessment. (UNC School of Medicine)

Bipolar disorder (mainly with mood stabilizers)

After reaching emotional stability, enter the maintenance period. Guidelines generally recommend at least two years. Many patients require long-term/continuous maintenance treatment (lithium salts/valproic acid/quetiapine/quinazole, etc. are selected on an individualized basis). (psychiatry-psychopharmacology.com)

When is it necessary to maintain it for a longer time?

Previous multiple relapses or early onset;

There are still residual symptoms after relief.

Combined with suicide/violence risks, substance use, pregnancy plans, etc.

It relapsed rapidly after drug withdrawal in the past.

The above factors are often used by various guidelines as the basis for extending the maintenance period. (gpnotebook.com)

Can it stop? How can I park more safely?

Do not stop taking the medicine by yourself or stop it suddenly. All medications should be gradually tapered under the guidance of a doctor.

Antidepressants: It is recommended to gradually reduce the dosage in stages to lower the risk of withdrawal syndrome. The speed of dose reduction is related to the duration and dosage of medication and can range from several weeks to several months. (NICE)

Antipsychotic drugs: To reduce recurrence, a very slow dose reduction (often over several months) is usually required, along with close follow-up. Reducing the dosage too quickly will increase the risk of recurrence. (Ovid)

Benzodiazepines (diazepam) : Authoritative joint guidelines recommend a reduction of 5-10% every 2-4 weeks, usually no more than 25% every 2 weeks, and the reduction should be even slower at the end of the low-dose period. (downloads.asam.org)

Summary (Remember one sentence)

Depression/anxiety: 6 to 12 months after remission; High recurrence risk: ≥2 years. (NICE)

Obsessive-compulsive disorder: At least 12 months. (NICE)

Schizophrenia/psychotic disorder: First ≥1 year; Multiple times for 2 to 5 years or longer/long-term. (Psychopharmacology Institute)

Bipolar: Usually maintained for a long time (at least 2 years or more). (psychiatry-psychopharmacology.com)

When stopping medication, it is essential to proceed gradually and work with your doctor throughout the process to formulate and adjust the plan. (NICE)

**Q27Can mental illness be completely cured?**

Brief conclusion: Some mental illnesses can be "completely cured", while in more cases, it is "long-term remission, functional recovery, occasional recurrence but controllable". Therefore, rather than pursuing a "one-time cure", a more realistic and scientific goal is to reduce symptoms, prevent recurrence, and restore learning/working/interpersonal functions and the quality of life.

How exactly should it be understood?

cure: Symptoms disappear and do not reappear for a long time, and no longer require treatment. It can be achieved in some individuals with adjustment disorders, acute stress responses, one-time mild to moderate depressive episodes, panic disorders, etc.

remission: Symptoms significantly alleviate or disappear, function recovers, but there is still a risk of recurrence. It is commonly seen in major depression, generalized anxiety, obsessive-compulsive disorder, post-traumatic stress disorder (PTSD), etc.

recovery: The core goal of modern mental health is to be able to lead the desired life (study, work, intimate relationships) even if only a few symptoms may remain.

Long-term management: For instance, bipolar disorder and schizophrenia spectrum disorder mainly focus on "reducing recurrence + protecting brain and social functions". Through maintenance treatment, long-term stability can be achieved.

The key factors influencing the outcome

Early medical treatment and adequate treatment (full course of medication/psychotherapy).

Combined factors: Substance use, chronic physical illness, and sleep disorders can lower the recovery rate.

Social support: Family understanding, peer support, and a stable learning/working environment.

Self-management: Regular schedule, exercise, stress management, and recurrence early warning plan.

Continuous follow-up and recurrence prevention: Even if there is improvement, the medication should be gradually reduced or maintenance treatment maintained as prescribed by the doctor.

Effective treatment options (vary from person to person, usually a "combination punch" is better)

Psychotherapy: CBT (Cognitive Behavioral Therapy), ERP (Obsessive-Compulsive Disorder), exposure therapy (panic/agoraphobia), traumay-focused therapy /EMDR (PTSD), interpersonal therapy (depression), family therapy, etc.

Drug treatment: antidepressants, mood stabilizers, antipsychotic drugs, etc. Evaluated and followed up for adjustment by a psychiatrist.

Social psychological support: Psychological education, vocational rehabilitation, peer support groups, and reasonable adjustment in school/workplace.

Lifestyle: Regular sleep, exercise, diet, reduce alcohol and addictive substances, digital detoxification, meditation/mindfulness.

Common misunderstandings

"Just rely on willpower to get better" → Mental disorders have multiple mechanisms including biological, psychological and social ones, and require professional intervention.

"Once you take medicine, you'll have to take it for the rest of your life." → Not necessarily. Many people can reduce or discontinue their treatment under the guidance of a doctor after a period of maintenance treatment.

"Diagnosis means characterization" → Diagnosis is for finding an effective treatment path, not a label.

If you or someone around you is struggling

When suicidal thoughts, plans or behaviors occur, or when safety cannot be guaranteed: Please contact the local emergency number immediately or go to the nearest emergency department. At the same time, inform a trusted person to accompany you to the medical treatment.

In non-acute cases, making an appointment with a psychiatrist or clinical psychologist for an assessment as early as possible, along with the time of symptom onset, triggers, previous treatment and family history, can significantly improve the efficiency of medical visits.

**Q28Will mental illness recur after recovery?**

Short answer: Yes. Many mental disorders have a certain risk of recurrence after remission or recovery, but the degree of risk varies depending on the type of disease, treatment compliance and lifestyle. Continuous treatment and good self-management can significantly reduce the risk. Here is a practical panoramic view (with evidence) for you:

Overview of recurrence of common diseases

Major depressive disorder (MDD) : If antidepressants are discontinued after remission, the risk of recurrence within one year is significantly higher than if medication is continued. The combined study estimated that the overall recurrence rate was approximately ≈39% (with significant differences among different populations). Continuous medication/psychological intervention can nearly halve the risk. (BMJ)

Bipolar disorder (BD) : The disease is highly recurrent, and the vast majority of patients require maintenance treatment (such as mood stabilizers) to prevent recurrence and restore social function. (PMC)

Schizophrenia spectrum disorder/first-episode psychosis (FEP) : Maintaining antipsychotic drugs can significantly reduce recurrence. Discontinuing or reducing medication can significantly increase the risk of relapse (but not everyone will relapse, and individualized assessment and close follow-up are required). (PubMed)

Anxiety disorders: Generally, they can be relieved for a long time, but there is a cumulative recurrence over many years (gradually increasing over time). Long-term consolidation and follow-up are still required even after cognitive behavioral therapy (CBT). (PMC)

Why does it recur?

The biological susceptibility of the disease itself is intertwined with stress triggers (such as sleep disorders, substance use, major life events, etc.).

Premature discontinuation or irregular medication use, cessation of psychotherapy, comorbidities (such as substance use disorders), persistent high stress and sleep problems all increase the risk. (BMJ)

How to Minimize the Risk of Recurrence (Executable List)

Adhere to maintenance treatment

Regularly assess the minimum effective maintenance dose and course of treatment of the medication with the doctor; Do not stop or reduce the dosage of the medicine by yourself. (PMC)

Evidence-based psychological intervention

Depression/anxiety: CBT, mindfulness-based cognitive therapy (MBCT), etc. can reduce recurrence. Bipolar: Systematic mood education/family education is helpful for compliance and recurrence prevention. (PMC)

Formulate a "Relapse Prevention Plan" (write it down and share it with your family)

Identify your triggers and early warning signs (such as shortened sleep, disrupted sleep schedules, significant mood swings, social withdrawal, faster/slower thinking, signs of abnormal beliefs, etc.); Many people will show warning signs 2 to 8 weeks before a recurrence, and timely intervention is crucial. (PMC)

Lifestyle

Regular sleep and schedule, stable daily rhythm, moderate exercise, limit alcohol intake/avoid prohibited drugs, and manage stress. The above factors are important for both emotional disorders and psychotic disorders. (PMC)

Follow-up and support network

Maintain regular follow-up with the doctor/therapist (even if you feel good); Let trusted family members or friends know your warning signs and response steps so that they can remind and assist you in seeking medical treatment as early as possible. (PMC)

When should one seek medical attention or contact emergency care as soon as possible

If you experience suicidal or other-harming thoughts or plans, a resurgence of obvious psychotic symptoms (experiences of being victimized or controlled, speech and thinking disorders), or abnormal excitement, impulsive consumption or rash behavior despite hardly sleeping for several consecutive days, you should seek medical attention immediately or call the local emergency number.

Remember one sentence: Mental illness "can recur but is preventable." By following the doctor's advice for long-term, individualized maintenance treatment, combined with psychological intervention and daily rhythm management, and early identification of warning signs, most people can reduce the recurrence rate to a low level and maintain a good quality of life. If you wish, I can help you draft a personalized recurrence prevention list based on the above key points (not involving privacy, only for self-management purposes).

**Q29How to prevent the recurrence of mental illness?**

Here is a "universal and actionable" relapse prevention list for you (applicable to common mental disorders such as depression, bipolar disorder, psychotic disorders/schizophrenia, etc.). You can just do it directly. If you or your family members are experiencing acute symptoms, please seek medical attention first or contact emergency resources (24-hour assistance in Taiwan is available at the end of the text).

Key points at the daily and therapeutic levels

Keep taking the medicine regularly and do not stop or reduce the dosage by yourself. Maintenance treatment is the key to reducing the risk of recurrence. Both schizophrenia and bipolar disorder are recommended to continue medication after remission, and regular evaluations with a physician should be conducted to determine if dosage or regimen adjustments are necessary. For those who have difficulty taking medicine regularly, it is advisable to discuss with the physician long-acting injections and other methods to improve compliance and reduce recurrence. (Wiley Online Library)

Regular follow-up visits and monitoring of side effects/physical health indicators. Weight, metabolism and cardiovascular risk should be tracked in a planned manner; These are also important considerations for recurrence and drug withdrawal. (NICE)

Create a personal "recurrence warning signal" list and share it with family members/co-residents. For example: poor sleep, increased anxiety/irritability, reduced concentration, decreased motivation, social withdrawal, aggravated abnormal thoughts, etc. Once it occurs, contact the treatment team as soon as possible, add appropriate medication or increase the frequency of meetings. (PMC)

Write a "Crisis and Recurrence Response Plan". It includes: who is the first contact person, the phone number of the doctor and the clinic, when it is necessary to go to the emergency department, and whether there are usage guidelines and storage methods for short-term "emergency drugs". (NICE)

Psychosocial intervention should be carried out in parallel. Family intervention, psychological education, cognitive behavioral therapy (CBT), and social rhythm/interpersonal therapy (IPSRT, especially suitable for bipolar disorder) can enhance compliance, identify early warnings, and reduce recurrence. (PMC)

Maintain a regular schedule and stable sleep. Insomnia is associated with disrupted sleep schedules and the recurrence of emotional/mental symptoms. Try to keep a fixed time for going to bed and getting up, and avoid staying up late and reversing day and night. (Frontiers)

Avoid alcohol and over-the-counter/recreational drugs; Use irritating substances (containing high caffeine) with caution. The use of substances can interfere with the efficacy of drugs and induce relapse. If it is difficult to quit, seek assistance related to addiction as soon as possible. (NCBI)

Stress reduction and emotion regulation training. Regular exercise, relaxation training, mindfulness, and a gradual plan for resuming work or school can help reduce the triggering factors. Learning support and communication skills for co-residents can also be significantly helpful. (NICE)

Care for chronic diseases and lifestyles. A balanced diet, weight management, quitting smoking and dealing with comorbidities (such as thyroid disorders, metabolic syndrome, sleep apnea) can enhance overall stability and medication tolerance. (NICE)

Bonus strategies for diagnosis (Discuss with the physician)

Schizophrenia/psychotic disorder: Maintenance antipsychotic drug treatment as per the guidelines; If there are compliance issues or multiple relapses, long-acting injections can be considered (multiple studies have shown that they can help reduce recurrence/rehospitalization). (Wiley Online Library)

Bipolar disorder: During the maintenance period, using mood stabilizers/antipsychotic drugs that were effective in the acute phase in the past as the foundation, combined with psychotherapy, can further reduce recurrence and improve compliance. Regular social and sleep rhythms (IPSRT) are particularly helpful in preventing bipolar episodes. (PMC)

Three steps you can take right away

Make a list of your warning signs (such as poor sleep, mood swings, social withdrawal, etc.) and tell a trusted person. (PMC)

Confirm with the attending physician: the maintenance period medication plan, whether compliance tools (reminders, medicine boxes, long-acting injections) are needed, and the date of the next follow-up visit. (Wiley Online Library)

Set a fixed schedule (going to bed and getting up at the same time), follow it for two consecutive weeks, and record your sleep and mood. If warning signals appear or the score rises, contact the treatment team as soon as possible. (Frontiers)

Need immediate assistance?

In Taiwan, if you have intense thoughts of despair or self-harm, please seek help immediately: 1925 Peace of Mind Hotline (24 hours a day, free of charge), or visit the nearest emergency department. (dep.mohw.gov.tw)

**Q30Can mental illness be passed on to the next generation?**

Brief answer:

Some mental illnesses "have a genetic predisposition", but they are not determined by a single gene like blood types and are not necessarily passed on to the next generation. Most of them are due to the combined effect of "multiple genes + environment" : if someone in the family is ill, it will increase the risk of the offspring, but most children will not fall ill throughout their lives. (nimh.nih.gov)

How exactly should we understand "whether it will be inherited"?

Schizophrenia: Twin and family studies suggest a relatively high heritability (about 70% - 80%), but it is not deterministic. Even identical twins are not a certainty. (PubMed)

The commonly used "family relative risk" in general epidemiological data is: if one parent is ill, the lifetime risk for the offspring is approximately 10% to 15%. If both parents are ill, the risk can reach approximately 40% (the baseline risk in the population is about 1%). These are group estimates used to understand trends rather than individual predictions. (EBSCO)

Bipolar disorder: The heritability is often estimated to be within the range of 60% to 90%. When a first-degree relative (parent or sibling) is ill, the risk for the offspring is significantly higher than that of the general population. (PMC)

Major depressive disorder: Heritability is approximately 30% to 50%. The illness of a first-degree relative can roughly increase an individual's risk by 2 to 3 times, but the environment and life events are equally important. (PMC)

The above-mentioned "heritability" is a statistical indicator, reflecting that the proportion of variation in a population is related to genetics. It does not equal the "probability of onset" at the individual level. (nimh.nih.gov)

What other factors are influencing it?

Environmental and stress factors (such as childhood adversity, major stress, substance use, infections/pregnancy complications, etc.) can interact with genetic susceptibility, influencing whether and when the disease occurs. (World Health Organization

What can I do if someone in my family is ill?

Informed and early identification: Inform the doctor of the family history; Paying attention to suspicious early signals and intervening early usually leads to better therapeutic effects. (nimh.nih.gov)

Reducing modifiable factors: maintaining a regular schedule, managing stress, avoiding alcohol/drug abuse, and building a support network, etc., have been proven to be beneficial for mental health and relapse prevention. (PMC)

Professional consultation: If you have concerns about fertility risks, you can discuss individualized risks and plans with a psychiatrist or genetic counselor. (nimh.nih.gov)

**Q31How can patients with mental illnesses better reintegrate into society?**

The following answer breaks down "reintegrating into society" into four levels: individual, family, work/school, community and system, and provides practical steps and a list. You can directly follow the "90-day Action Plan" and make minor adjustments as you go along based on your personal circumstances.

Core concept (First state the conclusion)

Recovery is a sustainable and long-term process, with the goal of "living well with illness" rather than "being completely asymptomatic".

Function first: Restore daily routine, social interaction and role functions first, and then pursue complete relief of symptoms.

Multi-party collaboration: myself + family members/friends + medical social workers + employers/schools + community resources.

The most effective evidence pathways: individualized supportive employment (IPS), supportive housing, psychotherapy (such as CBT/ACT), family psychological education, peer support, reasonable work/academic fit, and anti-stigma.

I. What can the parties do

Stabilize three things: keep a regular schedule (get up and go to bed at fixed times/have three meals a day), walk or do light exercise for 30 to 45 minutes every day, and set a small task (make the bed/water the flowers/cook).

Recurrence prevention package: Record early warning signs (such as insomnia, social withdrawal, faster/slower thinking, etc.), and list "If they occur → who to contact/what to do" (doctor, family member, crisis hotline, medical orders for adding/adjusting medications).

Treatment and follow-up: Adhere to outpatient visits and prescriptions. If you are troubled by side effects, bring specific examples back for discussion (do not stop taking the medicine on your own).

Psychotherapy: CBT/ACT/ family therapy/social skills training can help improve function and cope with stress.

Gradual exposure and regression: Start with low-difficulty social/work tasks and quantify your goals (e.g., meeting friends once a week → twice a week; replying to two messages a day).

Skills and Interests: Sign up for short-term courses (computer, language, handicrafts) to "warm up your brain".

Peer support: Join a group of patients or those in recovery and learn others' "living strategies while ill".

Digital tools: Use calendar/to-do/mood tracking apps (simple enough to stick with).

Second, what family members/friends can do

Communication principle: Less criticism, more description and listening; Use "My Message" (" I observed... ") I'm worried... "

Family psychological education: Understanding diseases, medications and recurrence signals; Practice crisis response.

Reduce the intensity of emotional expression (EE) within the family: Decrease criticism/high expectations, and set boundaries between "what to talk about and what not to talk about".

Assistance rhythm: Slice the big goal (job hunting → resume first → interview practice → part-time job).

Joint activities: One fixed low-pressure activity per week (walking, cooking, watching movies).

Iii. Returning to Work/School (The Key to Functional Recovery)

Give priority to "Supported Employment (IPS)" : Receive career counseling and medical collaboration while working (the best results are achieved if this service is available in your city).

Reasonable adaptation/convenience measures: flexible working hours, progressive working hours (half-day shift first), quiet workstations, clear task lists, written rather than oral instructions, delayed or phased completion of examinations, etc.

Disclosure strategy: Whether to disclose the diagnosis depends on the environment and needs. It is only necessary to state "there is a health condition that requires the following conveniences."

Rhythm and Review: A brief review with the counselor/supervisor every week: What can be done and where adjustments are needed.

Volunteer/Internship: As a "buffer zone" before the return, accumulate successful experience and connections.

Iv. Community and System (Can be jointly promoted by social Workers and family members)

Supportive housing/home support: Assistance with renting, finance and life skills.

Peer mentor and Day Rehabilitation Center: Offering group activities, vocational rehabilitation, and social training.

Anti-stigma Campaign: Participate in or advocate for community lectures, campus/enterprise mental health promotion.

Law and Rights: Understand disability status and reasonable accommodations, anti-discrimination regulations and subsidy channels (Policies vary by region. You can consult social workers or public health authorities).

V. Common Obstacles and Corresponding Strategies

Drug side effects → Do not stop taking the medicine; Record the time and intensity for follow-up fine-tuning (dressing change/dosage reduction/symptomatic treatment).

Reverse your schedule → First, fix your wake-up time and get exposed to the morning light, and stay away from caffeine and screens before going to bed.

Social anxiety → Gradual exposure + role-playing practice + carrying a "dialogue script".

Self-stigmatization → Communicate with peers, write personal "recovery stories", and distinguish "who I am" from "what I have experienced".

Recurrence signs → Activate the prevention package: Contact the doctor/family member, reduce the burden in the short term, and take sick leave and adjust the medication when necessary.

Vi. 90-Day Executable Action Plan (Can be directly followed)

Days 1 to 30 (Base)

Get up at the same time every day and walk for 30 minutes.

Organize the "Recurrence Prevention Package" and share it with family members/doctors.

One peer/group activity per week; Establish medicine boxes and reminders.

Contact career counseling/social workers to assess IPS or volunteer service points.

Days 31-60 (Small step Return)

Select 1 to 2 social roles (volunteer/part-time/course).

Set a pace of "2 hours of work/study ×3 days per week" and gradually increase it.

Practice low-conflict communication with family members; A family meeting once a week (15 to 30 minutes).

Days 61-90 (Consolidation and Expansion)

If the situation is stable, try a half-day class or a 50-70% academic load.

Confirm reasonable accommodations and written task lists with your employer/teacher.

Weekly review: Achievement of functional goals, stressors, and next steps for adjustment.

Vii. Crisis and Seeking Help

Seek medical attention immediately/Contact emergency resources: Self-harming or self-harming thoughts or behaviors occur, significant impairment in reality tests, and inability to take care of oneself.

Personal information card: Main diagnosis, medication, allergies, attending physician and emergency contact person.

Safety plan: Triggers, mitigation steps, contactable people and accessible safe locations.

Viii. Ways of Speaking to the Parties Involved (Reducing Harm)

Example of usage:

"I care about you and I see you working so hard." "

Let's think of a small step we can take today together.

"If you like, I can accompany you to the outpatient department/interview/registration." "

Avoid: labeling, denying efforts, and urging "get well soon".

Ix. Common Misunderstandings

"Must be completely asymptomatic to work/study" → No need; The function can be gradually restored with support.

"Once you take the medicine, you'll become addicted or stupid" → Inaccurate; Standardized medication has more advantages than disadvantages. If there are any side effects, adjustments can be made.

"Recurrence = failure" → Recurrence is part of the disease's characteristics and requires strategy adjustment rather than failure.

**Q32How to deal with discrimination and gossip from others about people with mental illnesses?**

The following "Practical countermeasure package" will help you protect yourself (or the person involved) and respond more effectively to and improve the environment when facing discrimination and comments from others about people with mental illnesses. Just take as needed.

1) First, keep yourself steady: Put "safety and energy" first

First, assess the occasion: Is it safe and worth responding to? If the other party is emotional or has a large number of people, it is better to withdraw first rather than confront them head-on.

There is no need to popularize knowledge on the spot: When you are offended, you have no obligation to educate others. First, protect your emotions and physical strength.

Three-step disconnection method: Become aware and trigger → take a deep breath/pause → Shift (change topic, leave, mute online/block).

2) Set boundaries: Be brief, clear and repetitive

You can directly use the following phrases (in tone from gentle to firm) :

Gentle reminder: "This makes me/us feel a bit uncomfortable. Let's change the topic."

It is inaccurate and unfair to equate mental illness with 'danger/laziness'.

Firm stop-loss: "I don't accept this statement." I'm going to end this conversation now.

In team Settings (group chats/meetings) : "We adhere to the principle of non-stigmatizing communication. This comment is inappropriate."

3) Provide a "short but powerful" factual substitute

When you are willing to respond, the principle is: one fact + one humanistic sentence

Mental disorders are very common and can be treated and recovered from. Please use "People with depression" instead of using labels to curse others.

"Symptoms do not equal people;" Most people with mental illnesses do not pose a danger to others.

Seeking treatment is a sign of responsibility, not being "sentimental".

4) Information disclosure should be "controllable, reversible and minimized".

Only inform those who need to know (for example, if it is necessary for reasonable convenience by the school or company), and keep the written materials.

Think clearly about the purpose, boundaries and possible impacts before deciding whether to share your medical history.

In the online environment, talk less about privacy and make good use of anonymity and blocking.

5) Operation lists for different scenarios

Family/Friends

First, agree on the communication rules: Do not label and listen before evaluating.

Post a brief popular science article or a medical visit guide, with one topic at a time to avoid being distracted.

Repetitive boundary: "I am willing to discuss treatment arrangements, but I do not accept personal evaluations."

School/Workplace

Record discriminatory words and deeds (time, place, exact words, screenshots/emails).

Understand and apply for reasonable conveniences (such as flexible working hours, quiet workstations, taking leave for follow-up visits, etc.).

Report to the tutoring center /HR/ superior according to the process. Seek legal or third-party mediation advice when necessary.

Medical setting

Take your companions as "communication supporters"; Write the questions on paper.

If you feel underestimated, you can apply for a change of doctor or a second opinion.

Cyberspace

First, take a screenshot as evidence, and then block or report it. Don't keep tugging at the other party's territory.

6) Prevent internalization of stigma: Build your Own "Psychological Armor"

Rewrite "Their words" into testable ideas:

Example: "Am I in a bad state?" → "I'm in the illness/recovery period and need support and structured arrangements."

Establish a support list: 3 people you can contact, 2 things that can stabilize your emotions, and 1 backup plan (such as an urgent follow-up visit).

Maintain a regular schedule and have regular follow-up visits. When symptoms arise, prioritize self-care over debating.

7) If you are an onlooker/Colleague: How to do "Micro-intervention"

On-stage correction: "I understand your concern, but confusing people with diagnosis is inaccurate." "

Turning to the rule: "Our team discusses facts and plans and does not evaluate individual medical histories."

Amplify support: Affirm the professional/academic contributions of the person involved and weaken the narrative of bias.

8) When will the processing be upgraded?

In the event of persistent harassment, threats, exclusion, or any impact on learning or work assessment, immediately follow the formal procedures (school appeal /HR compliance/legal consultation).

If the party concerned is at risk of self-harm or harm to others, or shows obvious signs of deterioration (such as extreme despair or planned speech), contact local emergency/crisis intervention resources directly or seek emergency treatment nearby.

9) Portable response card (stored in the mobile phone

"Please don't use mental illness as an insult." "

This is not in line with the facts and shows no respect for people. Let's change the topic."

"I'm willing to discuss how to do things well, not the medical history evaluation." "

I have kept this message. If I continue, I will go through the formal process.

10) Sustainable Change: Small-step long-termism

Advocate human-oriented language, share reliable popular science, and recommend supporting resources within a controllable range.

If you have the capacity, join or organize peer support groups or mental health advocacy activities to form a mutual assistance network.

**Q33Can one fall in love with someone who has a mental illness?**

Sure. It is entirely possible to fall in love with someone who has a mental illness. The key lies in understanding, respecting and supporting each other. If you have established a sincere relationship with the other person and are willing to support each other emotionally, then mental illness will not be an obstacle to a romantic relationship.

However, when in a relationship, the following points should be noted:

Understanding and support: Mental illness is a complex health condition that may affect emotions, behaviors, or ways of thinking. It is very important to understand and respect your partner's illness, and at the same time encourage them to receive professional treatment and support.

Communication and patience: Mental illness may cause emotional fluctuations or behavioral instability. Maintaining good communication and resolving conflicts and doubts in a timely manner are crucial for maintaining a healthy relationship.

Boundaries and Self-care: While helping your partner, it's also important to maintain your own emotional and mental health. Sometimes you may need to set boundaries for yourself to ensure that you don't feel overly stressed or anxious.

Seek professional resources: If you feel difficult, seeking psychological counseling or treatment support is a good choice. Professional help can provide more guidance and advice for your relationship.

In conclusion, it is feasible to establish healthy and loving relationships with people who have mental illnesses, provided that there is sufficient understanding, patience and support between each other.

**Q34Can people with mental illnesses get married?**

Sure. The vast majority of jurisdictions do not uniformly prohibit marriage just because one has a "mental illness". The key does not lie in the diagnosis itself, but in whether the parties involved have the ability to consent to marriage at the time of marriage (being able to understand the meaning, rights and obligations of marriage and express consent independently). Simply put:

The circumstances under which one can get married: In the stable treatment period of most mental illnesses, individuals can understand and independently decide to get married - usually without legal obstacles.

Possible restricted situations

During the acute attack period, when one is in a state of confusion or is affected by severe delusions/cognitive impairment and is unable to understand or express true intentions, the "agreement" made at the moment may be invalid.

If a person is declared by the court to have no or limited capacity for civil conduct and has their guardian handle major matters on their behalf, the consent of the guardian or the permission of the court may be required. In some places, marriage may be restricted in extreme circumstances.

Regional differences: Regulations on capacity for civil conduct, guardianship and marriage registration vary from country to country/region to region. In a few places, there are also obligations for health checks or declarations. The specific regulations should be subject to the local civil law and marriage registration provisions.

Practical suggestions

Have an open and honest communication with your partner about your illness, treatment and possible signs of recurrence, and discuss coping and support plans.

Maintain regular treatment and follow-up; Before choosing a date for registration or holding a ceremony, make sure it is in a stable period.

If it involves guardianship or capacity assessment, consult the local civil affairs/household registration authority or a lawyer as soon as possible about the operation procedures.

If you are concerned about genetic or medication and fertility issues, you can consult a psychiatrist, obstetrician or genetic counselor.

In conclusion, mental illness does not mean being unable to get married. As long as one has a clear and free consent capacity at the time of registration and complies with local legal procedures, they can usually get married in accordance with the law.

**Q35How to get along better with family members suffering from mental illness?**

Sure. When getting along with family members suffering from mental illness, the key points are "understanding - cooperation - boundaries - self-care". Here's a set of practical tips for you. You can start with what suits your family first.

First, separate "illness" from "person"

Learn more about your family members' diagnoses, common symptoms, triggers and misunderstandings (for example: depression ≠ laziness; anxiety ≠ being overly sensitive; psychotic symptoms ≠ "intentional"). When we look at behavior in the context of symptoms, people will be easier to get along with.

Record "Trigger - Response - Mitigation" : On an A4 sheet, write the trigger situation in the left column, the current performance in the middle column, and what methods are useful in the right column. The pattern can be seen in 2 to 3 weeks.

Second, be able to speak: Use the four-sentence template of "empathy + concreteness"

When the other person gets emotional, try to use the following rhythm (you can read it accordingly) :

Describe the facts

"I heard you couldn't sleep again last night..." "

Express empathy

This must be very tormenting. You've been holding on for a long time.

Just ask one small question:

What troubles you most now is whether your mind can't stop or your heart is tight?

Provide an optional next step (not mandatory) :

"Would you like to sit on the sofa for a while first? I'll do three minutes of breathing with you/bring the medicine box and check it once?" "

Iii. Concretize "Cooperation" (Family Support Agreement)

Write the following content on one page together with your family and post it in the public area:

Morning and evening routine: What time to get up/go to bed, check off the medication/scale, and take a 10-15 minute walk.

Crisis signal light

Green: Live as usual;

Huang: Unable to sleep for two nights, poor appetite, and starting to hide in the room - activate the "burden reduction" list (reducing household chores/work load) and contact a therapist;

Red: Thoughts that harm oneself or others, auditory hallucinations of instructions, loss of direction - follow the crisis process.

Crisis process (posted on the refrigerator) :

First, accompany, conduct a safety check (collect dangerous items), and contact the attending physician/clinic. Call the 1925 Peace of Mind hotline or the 1995 Lifeline for help when necessary. In case of immediate danger, call 119 directly. (Taichung City Government)

Four. Set "boundaries" Instead of "confrontation"

"Making things clear in advance" is more effective than "arguing afterwards"

For example: "I can chat with you for 20 minutes, but I need to sleep after 12 a.m. If I still feel uncomfortable, we'll follow the crisis process."

Refuse invalid arguments: When you have delusions or compulsions, don't force yourself to argue "true/false". You can respond: "I believe you feel very real; let's first reduce your current fear to 5/10 and then think of a way together."

V. Adhere to Professional Treatment (5 Small Things Family Members Can Do)

Medication and Follow-up Visits: Prepare a one-week medicine box and write down three questions you most want to ask before your follow-up visit.

Psychotherapy: Help schedule a fixed time slot to avoid last-minute cancellations.

Life structure: Regular schedule, sun exposure and light exercise (even if it's just 10 minutes).

Substance usage: Try to avoid alcohol, excessive caffeine, and illegal drugs.

Social connection: At least one non-therapeutic light social activity per week (walking, visiting convenience stores, or playing video games for 30 minutes all count).

Six. Caring for "Caregivers" : You also need to be aerobic

Set a "breathing space" : Set aside 1 to 2 hours each week exclusively for yourself (exercising, meeting friends, or daydreaming).

Division of labor within the family: Assign "emotional companionship", "transportation for follow-up visits", "medication and household chores" to different people.

Study group/family courses: You can learn communication skills and also reduce feelings of loneliness.

You can also call for help: Family members can also seek advice from the hotline.

Vii. Taiwan Resources (24/7 or Local)

Peace of Mind Hotline (Suicide Prevention) 1925 (Multilingual Service) - 24 hours. (Immigration Bureau

Lifeline 1995-24/7 Crisis and Psychological Support. (Taichung City Government)

Teacher Zhang's hotline: 1980 - Telephone and in-person counseling (during certain periods). (Teacher Zhang Foundation

In case of emergency, please call 119 (for medical assistance) or the nearest emergency psychiatric department.

Viii. Quick Response to Common Problems

Refuse to seek medical treatment/take medicine

"Seeking medical treatment is not about admitting 'I'm not normal', but about 'reducing the pain a little'; we first make an appointment for an 'assessment' rather than 'long-term treatment', and you have the final say." "

Emotional outburst (yelling/throwing things) :

Evacuate to a safe distance first; Repeat the requirements in a low and slow voice: "You really want to be heard. I'll listen. We'll continue in five minutes. For now, drink some water/wash your face."

Self-harm/suicide discussion:

Asking directly won't "corrupt" the other party. One can ask: "Do you have any thoughts of hurting yourself? How strong are you? Do you have any plans?" If the answer is "Yes and specific", immediately follow the crisis process and seek professional and first aid. (Taichung City Government)

If you wish, I can also help you make the "Family Support Agreement" into a one-page template, or reprioritize the above steps based on the specific circumstances of your family members (such as their biggest recent concern, whether they are taking medication, or whether they are seeing a doctor).

Final reminder: If you or your family members are at risk at the moment, please contact 1925/1995/1980 immediately or call 119 directly. You are not facing it alone. (Taichung City Government)

**Q36How to determine if a family member has a recurrence of mental illness?**

The following guide will help you determine whether a family member may experience a recurrence of mental illness in an observable and actionable way, and tell you what to do. If you feel that the other person or yourself is in immediate danger at this moment, please directly refer to "Emergency Signals and Ways to Seek Help" in Part 2.

1) Common "early warning signs before recurrence

Many relapses have a "change period" (which may occur a few days to several weeks earlier) before significant symptoms appear. Family members are usually the first to notice these changes

Sudden changes in mood/vitality: Feeling depressed or irritable, easily angered, with a significant decline in interest and motivation, and energy levels fluctuating. (Mental Health America)

Sleep and daily routine: Obvious insomnia, reversed day and night schedules, sudden excessive or insufficient sleep. (Mental Health America)

Behavior and social interaction: Avoiding family and friends, not going to work or school, and losing follow-up on daily responsibilities (bills, hygiene, appointments). (Mental Health America)

Thought and Perception: Reduced concentration, slower reaction, suspicious thoughts of being victimized or feeling "someone is following me/speaking ill of me"

Extremely sensitive to sound/light stimuli. (Mental Health America)

Function decline: Learning/work efficiency drops sharply, and self-care (washing up, eating, dressing) deteriorates significantly. (Mayo Clinic)

Disease-specific signals (taking psychotic disorders as an example) : Research shows that before a relapse, there are often "early warning signs" such as emotional fluctuations, social withdrawal, sleep disorders, and slightly odd/suspicious behavior, which sometimes appear several weeks before a relapse. If identified and intervened early, serious recurrence can often be kept out. (PMC)

The key point is that a single bad mood does not mean a recurrence, but rather that there are continuous, multiple changes compared to one's usual "baseline" that cause functional impairment.

2) Emergency Signals and Assistance Methods (Taiwan)

Please seek help immediately if any of the following situations occur:

Clear suicidal thoughts/plans/preparatory behaviors; Repeatedly expressing "There is no meaning in life" along with signs of action. (nimh.nih.gov)

There is a risk of injuring others, extreme loss of control over behavior, or severe hallucinations/delusions that threaten safety. (Mayo Clinic)

Emergency/crisis contact information in Taiwan:

Call 119 (ambulance/fire) for emergency medical assistance; Or call 110 (the police). (ait.org.tw)

24-hour Hotline 1925 (Still Love Me) (Ministry of Health and Welfare, Suicide Prevention/Emotional Support). (Taipei Times

Lifeline 1995 (24-hour Emotional/Crisis Support) (Life 1995

If your mobile phone has poor signal reception, you can dial 112 to transfer to 110/119. (Taipei City Government

3) How to determine more systematically whether it is relapsing

Stringing together the following four dimensions will be more reliable than "relying on feelings" :

Compared with the baseline: What is the "usual appearance" of this family member? Is the current change continuous for ≥1-2 weeks? Has it affected study/work/relationships/finances? (Mayo Clinic)

Number and intensity of signals: Among the above early warning signals, how many were hit? Is the intensity increasing? (For example: from mild insomnia to staying up all night.) (PMC)

Triggers and Medication: Have you recently stopped taking medication or missed any, experienced significant stress (bereavement, exams/job changes, staying up late), or increased alcohol or drug use? All of these will significantly increase the risk of recurrence. (NICE)

Past pattern: What unique signals did he/she show before his/her last recurrence? Everyone's "warning list" is different; identifying personalized signals is particularly important. (PMC)

4) Things that family members can do now (in order of priority

First, ensure safety: If there is a risk of suicide or injury from others, handle it as per Part 2. Do not leave anyone alone or remove dangerous items (large quantities of medicines, sharp objects). (nimh.nih.gov)

Gentle communication and reduced confrontation: Use "I" statements (such as "I've noticed that you've been sleeping very little recently. I'm worried you're tired"), and avoid labeling or arguing "Are you ill?" (PMC)

Contact professionals as soon as possible: Contact the original attending physician or the community mental health team; The NICE guidelines emphasize that early identification, rapid referral and family participation can improve prognosis. Bring the "change list" you have recorded when seeking medical treatment. (NICE)

Adhere to the four basic health care tasks: take medicine regularly, sleep regularly, keep a regular schedule/do light exercise, and reduce alcohol and substances. These several points are particularly crucial for preventing recurrence. (NICE)

Recording and monitoring: Write down "how many hours of sleep each day, mood 0-10, self-assessed anxiety 0-10, whether going out/working/contacting friends" on paper or mobile phone. Variable curves are more valuable for reference than single-point curves. Both research and guidelines recommend "early signal monitoring" as part of recurrence prevention. (PMC)

5) Simple "Recurrence Plan" template (Copyable)

Write the following contents together and stick them in your phone's memo or a public area at home:

My personal warning signs (for example: insomnia for two consecutive days, starting to suspect that colleagues are talking about me, not wanting to take a bath). (PMC)

How do I want my family to help (accompany me to the doctor, call the doctor on my behalf, help me organize my medicine box, and take a 20-minute walk together)? (PMC)

Professional contact: Name and phone number of the attending physician/clinic/social worker, date of the next follow-up visit. (NICE)

Support circle: Names and phone numbers of 2-3 trusted family members and friends.

Crisis steps: Suicidal thoughts occur → Who to call first → If unreachable, dial 1925/1995/119. (Taipei Times

6) Some minefields of "Don't do this"

Do not advise them to stop taking the medicine immediately or reduce the dosage on their own (it may trigger a rebound or recurrence). (NICE)

Don't use accusation or shame to push for medical treatment (counterproductive). Focus on "functionality and security" rather than "How could you do this again?" (PMC)

Don't attribute all anomalies to "laziness/lack of effort". When the function is impaired and persists, it should be treated as a health issue. (Mayo Clinic)

**Q37What should be done when 37 family members experience auditory hallucinations?**

Here is an "ready-to-use" list of ways to deal with family members' auditory hallucinations. First, ensure safety, then provide support and seek medical attention:

First, ensure safety (do it right away)

Assess whether there is a risk of self-injury or injury to others

There are "voices telling him/her to hurt himself/herself or others", extreme excitement, confusion to the point of not recognizing time and place, accompanied by high fever, head trauma, recent heavy drinking/medication or stopping medication, directly seeking emergency treatment. In Taiwan, please call 119 (for medical assistance/fire), and for police help, call 110. (Taipei City Police Department)

Temporarily remove dangerous objects: knives, scissors, medicines, ropes, heights, etc.

Companionship without argument: Respond to emotions with calm and brief sentences (" I see you scared, I'm here "), without arguing about "whether the voice is true or not/whether the content is right or wrong". First, catch your feelings, and then talk about seeking help. Face calmly, with moderate lighting and a quiet environment as much as possible.

How to talk to him/Her (immediate communication)

Use a low-stimulation environment: Turn off harsh music/TV and reduce the number of onlookers.

Affirm your feelings and draw boundaries: For example, "I know this makes you very uncomfortable; For your safety, let's contact the doctor/go to the hospital together."

Offer options rather than commands: For example, "Would you like to sit down for a drink of water or go to the balcony for some fresh air?"

If imperative auditory hallucinations occur (voices gives instructions), repeat the safety boundary and turn to first aid.

After it stabilizes a little: Seek medical treatment and follow up

Contact the psychiatry/psychosomatic medicine department as soon as possible for an assessment. Auditory hallucinations may be seen in psychotic disorders, major depression/bipolar seizures, delirium/dementia, epilepsy, substance influence, etc., and require professional judgment and treatment (medication + psychological/family support). The NHS and mental health agencies also recommend seeking an assessment as soon as possible if auditory hallucinations occur. (nhs.uk)

Small materials to be taken for medical treatment

The onset time of symptoms, triggers (stress/insomnia/medication changes/alcohol or drugs), whether there are directive contents, self-harming thoughts, previous diagnoses and medications, family history.

Regular sleep schedule: Prioritize sleep, eat regularly, and consume less caffeine and alcohol. Continue or adjust the medication as prescribed by the doctor. Do not stop taking the medicine on your own. Mental health institutions have detailed suggestions on coping techniques (such as orientation/ground orientation, distraction, and setting boundaries with "voices") that can be referred to. (rethink.org)

Family members can or should not do it

It can be done

Gently accompany and remind people to eat, drink and rest.

Jointly establish a "safety plan" : when to contact whom, when to go to the emergency room, and the way to store dangerous items at home.

Record "Triggers and Mitigation Methods" (when the sound becomes stronger and what activities weaken).

If you are a teenager, contact the school counseling and the child and adolescent psychiatry department/early intervention team as early as possible. (Oxford Health NHS Foundation Trust)

Don't do

Don't mock, label or force people to "cheer up".

Do not get entangled in debates with "sound content" or cater to its dangerous demands.

Don't leave him/her alone at home when it's obviously unsafe.

When must emergency assistance be sought immediately (emphasized again)

Hearing imperative auditory hallucinations can cause harm to others or oneself

Suicidal thoughts/plans have emerged

Disordered behavior, inability to take care of oneself, intense agitation or confusion of consciousness

Combined medical emergency signs: high fever, severe pain, convulsions, head trauma, suspected poisoning/withdrawal

Call 119/110 directly or go to the nearest emergency department. (Taipei City Police Department)

Emergency and support resources in Taiwan region

Emergency medical care/fire: 119; Police: 110 (24 hours). (Taipei City Police Department)

National Peace of Mind Hotline (Suicide Prevention) 1925 (Still Love Me), 24-hour, free of charge, anonymous. (Taichung City Health Bureau

(Supplementary) Some information mentions that Lifeline 1995 can provide emotional support (services are available at various locations; please refer to official or local announcements). (4710170.fs1.hubspotusercontent-na1.net)

If you are worried about your family's safety right now, please call 119/110 first, or choose one of the hotlines I listed above to get through to first and handle it step by step together with the operator. (Taipei City Police Department)

**Q38What should be done if a patient with mental illness shows suicidal behavior?**

If you (or someone around you) have suicidal thoughts or behaviors due to mental illness, the most important thing is to ensure your safety first and then connect with professional support as soon as possible. The following is a list of actionable steps

When there is immediate danger (actions, plans, tools are already in place, or safety cannot be guaranteed)

Call the emergency number immediately: In Taiwan, please dial 119 (for medical and fire services) or 110 (for police). These two numbers are available 24 hours a day (Taipei City Police Department)

Do not leave the client alone. Stay by their side (or via phone or video call) until a professional takes over.

Remove or lock up lethal means (drugs, sharp tools, ropes, heights, pesticides, etc.).

Listen in a non-judgmental way: less persuasion and less reasoning, and more listening to emotions and needs (such as "I'm here with you" or "Let's figure out a way together").

When there is still a risk but it is not immediate danger

Encourage and assist in contacting professional support (if the person concerned is unwilling to call, you can call on their behalf) :

1925 Peace of Mind Hotline (Still Love Me), 24-hour psychological support and suicide prevention provided by the Ministry of Health and Welfare. (Taichung City Health Bureau

1995 Lifeline, 24-hour Crisis Counseling. (LifeLine International)

Teacher Zhang Hotline 1980 (Suitable for both teenagers and the general public). (gsat.ntu.edu.tw)

Let's make a simple "safety plan" together (write it down, take a photo and keep it) :

Danger Warning: Under what circumstances do I usually get worse?

Self-soothing: Three things that can make me feel a little better (breathing, washing my face, taking a walk, listening to music).

Seeker: List 3 to 5 people you can contact in order (family member/friend/colleague/tutor) and write down their phone numbers.

Professional resources: Attending physician/Psychologist/social worker and the above-mentioned hotline.

Environmental safety: To whom should I temporarily entrust the high-risk items that I have access to?

Encourage medical attention and continuous treatment: Take medication regularly as prescribed by the doctor and return for follow-up visits. If necessary, assess hospitalization or day treatment. You can contact the community mental health center or the physical and mental health outpatient department of the hospital (the nearest one is preferred).

Follow-up care: Contact again within 24 to 72 hours after the crisis. In the following weeks, maintain regular follow-ups (via text message, phone call, or in-person meeting) to inquire about the side effects of medication and the arrangements for follow-up visits.

How to Communicate with the parties involved (Brief Tips)

When expressing feelings, don't argue: Use "I care about you" or "It sounds like you're really feeling bad" instead of "You're overthinking it".

Open-ended question + Repetition + Affirmation (OARS)

Open-ended: "Which moment have you been most unable to hold on to recently?"

Repeat: "You mean it's particularly hard to get through when it's late at night and quiet, right?"

Sure: "It's already very hard for you to say it out loud."

Avoid triggering phrases: Avoid blaming, downplaying or building hope on "being more optimistic" or "thinking about your family".

It can also be done as a family member or friend

Assist with medical visits and transportation, and accompany patients during outpatient services; Assist in organizing medications and providing medication reminders.

Establish daily support: keep a regular schedule, get some sun exposure, have a regular diet, and reduce alcohol and substance use.

Be aware of early warning signs such as sudden mood swings, saying goodbye/leaving a will, bidding farewell to beloved items, searching for suicide methods online, and sudden calmness. Once these occur, be vigilant and take the initiative to intervene.

If you are feeling like hurting yourself right now:

Dial 1925 or 1995 right now; If you have difficulty controlling yourself or are in danger, call 119/110. These are all 24-hour lifelines and emergency services. You don't have to bear it alone. (Taichung City Health Bureau

Note: The above-mentioned phone numbers are the official resource and emergency numbers currently in use in Taiwan. If you are outside Taiwan, please tell me your country/region and I will provide the corresponding local resources. (Taichung City Health Bureau

**Q39What should be done if a patient with mental illness shows violent tendencies?**

The following is a safety guideline that can be operated in real-world scenarios. If there is a clear risk of violence right in front of you, please read Paragraph 1 first.

What to do when there is danger in front of you (prioritize personal safety)

Evacuate immediately to a safe location, keep a distance from the person involved, and position yourself at the door or in an escape area.

Don't argue with or provoke them (such as blaming, threatening, mocking, or sudden physical contact).

In case of any emergency such as assault, armed injury or self-harm, call for help immediately: Taiwan region

Alarm: 110 Ambulance/Fire Service: 119. (Taipei City Government)

If they have self-harm or strong suicidal thoughts accompanied by the risk of agitation/violence, they can also contact the 24-hour psychological support hotline for immediate guidance

1925 Peace of Mind Hotline (Still Love Me), provided by the Ministry of Health and Welfare. (Taichung City Health Bureau

1995 Lifeline. (LifeLine International)

2) De-escalation communication method before escalation

Speak slowly and steadily, use short sentences, and give only one option at a time (for example: "Shall we sit down first and have some water?") "

Acknowledge your emotions and don't overcommit. (" I can tell you're feeling bad. Let's make the environment quieter first. ") "

Reduce irritation: Minimize gathering around, move sharp or heavy objects away, and avoid blocking their way with your body.

Give time and space: Allow silence, don't press for details, and don't rush to "reason".

Avoid forced constraints, unless guided by professionals or in immediate danger to life - incorrect constraints can intensify risks.

3) Post-event and ongoing handling

Contact a professional psychiatrist (psychiatrist, clinical psychologist, social worker) as soon as possible. Describe the behaviors that occurred, the triggers, medication compliance, etc.

Do not stop taking the medicine without permission or increase or decrease the dosage at will. If there are any side effects or efficacy issues with the medication, they should be evaluated by a physician.

Make a "safety plan"

Identify early warning signs (sudden changes in sleep, delusions of persecution, increased substance use, elevated irritability).

Clarify who will do what: who will make the call first, which hospital to go to, and what materials to bring (medical records, medication list).

Environmental safety: Locking or temporarily storing hazardous materials (knives, ropes, substances at risk of drug overdose, etc.).

Record the event (time, location, behavior, cause, and handling process) to facilitate medical assessment and subsequent support.

Family/caregiver support: Seek community resources and hotlines for care and self-care advice when necessary (1925/1995, etc.). (Taichung City Health Bureau

4) When is it essential to seek emergency medical assistance

There are specific threats or behaviors of harm (hitting others, destroying property, armed, self-harm).

Having a clear plan for self-harm or self-harm thoughts, or medicinal alcohol affecting judgment.

Confusion of consciousness, delusions/auditory hallucinations drive them to attack, making communication difficult.

In any situation that intuitively makes you feel "unsafe/about to get out of control" - please call 110/119 at this time, and you can also seek help from 1925/1995 for immediate guidance and companionship. (Taipei City Government)

5) Tips for caregivers

Rehearse safety plans in advance; Write the important phone numbers (110, 119, 1925, 1995) in a prominent place. (Taipei City Government)

Make an appointment with the attending team for the medical treatment route in case of crisis (the nearest emergency department/psychosomatic department), and prepare medical certificates and a list of previous prescriptions.

Maintain a regular schedule, supervise medication use, and reduce the triggers for alcohol/drug abuse; When necessary, collaborate with social workers to assess family support and resource referrals.

Even after the incident subsides, you should still take good care of yourself (take short breaks, receive peer support, and seek psychological counseling if necessary).

**Q40How to persuade a patient with mental illness who refuses to see a doctor?**

The following is a set of persuasive ideas that are both "effective and not overwhelming", suitable for use when a family member or friend you care about is temporarily reluctant to seek medical treatment. First ensure safety, and then gradually promote change.

First, determine the safety

If you experience any emergency risks such as self-harm or plans to harm others, severe confusion of consciousness, or inability to take care of yourself, please prioritize safety and immediately seek help from emergency resources: Taiwan's emergency police 110, fire/ambulance 119 (24 hours). (Taipei City Police Department)

If you are involved in suicide risks or are unsure of the severity, you can also call the 1925 (Still Love Me) Suicide Prevention hotline at the same time. This is a 24-hour service provided by the Ministry of Health and Welfare. (Taichung City Health Bureau

If you or the person involved are not in Taiwan or need English resources, you can call, text or chat at 988 Lifeline in the United States. (988 Lifeline)

When talking to him/her, use the four tricks of "motivational dialogue" (OARS)

This is an evidence-based communication method. The key point is to let the other party find the reasons for seeking medical treatment or help by themselves, rather than being "persuaded" by us. (Motivation Interview Official Website

Open questions

"Which period of time have been the most uncomfortable recently? Which one do you hope to improve in advance?" "

Sure (Affirmation) :

It's not easy for you to hold on until now. I can tell you're working hard.

Reflective listening

It sounds like the side effects of the medicine are very worrying to you, which is why you don't want to see a doctor, right?

Summary

"If you want to stabilize your sleep first and are willing to try a method that doesn't necessarily require medication, we can first contact the clinic where the meeting is held for an assessment." "

Specific and feasible small strategies (step by step

Start with "experiencing once" rather than "long-term treatment" : It is more acceptable to narrow the goal down to "make an appointment for an assessment/chat first". (HelpGuide.org)

Reduce medical treatment costs

You come to help with the reservation and transportation, accompany you to the scene or wait outside the door. (mind.org.uk)

Provide a sense of choice: Give 2-3 doctors/psychologists brief introductions and time slots, and let them decide which one to choose.

If you are concerned about medication, first choose a medical institution that mainly offers psychological counseling or physical and mental health assessment, and then discuss the medication as needed.

Remove the obstacles to each concern point one by one:

Worried about labeling/privacy → Explain medical privacy and options such as self-payment and night clinics.

Concerned about side effects → First, talk about "starting from a low dose and adjustable at any time during follow-up visits", emphasizing that it is not necessary to commit to continuous medication for now, and let a professional assessment take place.

Link "seeking medical treatment = achieving the life improvement that TA cares about" : Link seeking medical treatment to the goals TA cares about (sleep better, get through this month's work first). This is more useful than "going to see it for me/for my family". (HelpGuide.org)

Offer an alternative starting point: If you still resist going to the hospital at this moment, start with a hotline or online chat first, and then transition to the outpatient department. In Taiwan, you can first call 1925 or the local mental health center for consultation. (Taichung City Health Bureau

A dialogue script that can be directly copied

Opening: "I've been concerned about how hard you've been doing recently and want to know you better. What's bothering you the most recently, is it being unable to sleep or feeling down?" "

Empathy + reflection: "You're worried that the doctor will only prescribe medicine and you're also afraid of being labeled. No wonder you don't want to go."

Offer a choice: "Would you like to try an assessment first? I found three clinics with good reviews. We'll only choose one that suits us best. If you don't like it, we won't force it."

Connection goal: "If you can sort out your sleep first, you'll have more energy to go to work. Let's take this as our goal, okay?"

When faced with rejection: "I respect your decision. I'll be here, waiting for you to feel ready; if it gets worse one day, we'll figure out a way together."

Things not to do

Don't argue, label or threaten (" So what if you don't go ") - it will only strengthen resistance.

Don't pour out the list of suggestions all at once. Listen first, then focus. (Motivation Interview Official Website

The logistical work you can do (to be carried out immediately

Help him/her simplify the first step: organize the symptoms and questions to ask, prepare the health insurance card/past medical history, and arrange transportation and accompaniment. (mind.org.uk)

Prepare emergency and support contact information:

Taiwan: 110 (Police), 119 (Fire/Ambulance) Suicide Prevention Hotline 1925. (Taipei City Police Department)

Other: If you are outside Taiwan or need English assistance, you can also chat or text via 988 Lifeline in the United States. (988 Lifeline)

The government information page also lists the 113 domestic violence and sexual assault hotline and the number 0800-024-111 for foreigners in Taiwan. (GOV.UK)

**Q41Should family members with mental illnesses be allowed to manage their finances independently?**

Brief conclusion: A one-size-fits-all approach should not be adopted. Whether to allow family members with mental illness to manage their finances independently depends on their current decision-making ability, the stability of the illness course and the available safety measures. Give priority to "supportive decision-making" (preserving the autonomy of the parties involved as much as possible) rather than directly depriving them of financial rights, but at the same time, do a good job in risk control.

Suggested practices (from lenient to strict, advancing by risk)

Fully autonomous + mild support (at low risk)

Suitable for: stable condition, able to understand income and expenditure as well as consequences, and no history of serious impulsive consumption or fraud in the past.

Methods: Budget sheet, accounting App, automatic deduction of fixed bills, regular (such as monthly) family reconciliation.

Joint management/Partial limitation (Medium risk)

Suitable for: Occasionally impulsive or emotionally unstable, but capable of communication and cooperation.

Tool

Joint account (jointly signing for large expenditures)

Set the upper limit of transfer/single transaction amount and consumption reminder

Living expenses are distributed weekly or monthly

Prepaid card or supplementary card (with limit)

Large expenditures need to be confirmed with a second person during the "cooling-off period"

Designated agent/Entrusted management (High risk)

Suitable for: Frequent impulse spending, having been defrauded before, active manic/psychotic symptoms, and significantly impaired judgment.

Approach: Have a trusted family member manage the main account on your behalf. The party concerned only retains a small amount of cash/limit card. When necessary, discuss with social workers and hospital legal departments whether to handle an authorization letter/guardianship or have a compliant agency manage the payment on their behalf (in accordance with the regulations of the country where they are located).

Evaluation and decision-making checklist

Comprehension ability: Can you explain income, expenditure, debts and their consequences?

Consistency: Can a stable judgment be maintained on the same expense within a week?

Historical Record: Have there been any serious losses from extravagance, debt, or fraud? Frequency and amount?

Course of the disease and medication: Are the symptoms stable? Have you sought medical treatment or taken medicine on time?

Support network: Can someone regularly review income and expenditure together?

Inducing factors: Are there any impulses for online shopping, giving rewards or investing during the onset period?

Safety measures: Have limits, reminders, and double confirmations been set now?

If more than 2 to 3 items are unfavorable, it is recommended to first apply a "joint management/partial restriction" or a stricter plan, and then gradually delegate authority as the condition stabilizes.

Essential safety measures (regardless of the mode adopted)

Key account activation limits, early warnings and two-factor authentication; Large payments require confirmation from family members.

Regular reconciliation (at least once a month), and immediate handling of abnormal transactions.

Conduct scenario drills and contingency plans for common scams (handling scripts for calls from "customer service/public security, procuratorial and judicial authorities/investment teachers").

A cooling-off period (24 to 72 hours) is set for major expenditures.

Emergency plan for symptom fluctuations: Temporarily lower the limit/freeze large transfers + notify medical attention.

The key points of communicating with the parties involved

It is emphasized that the goal is to promote protection and self-reliance in parallel, not to deprive rights.

Jointly set quantifiable goals (such as zero overdue payments within three months and a certain amount of savings), and gradually delegate authority once the targets are met.

Write the rules into a concise agreement (including the quota, the frequency of reconciliation, and who has the final veto power), and review them regularly.

When should management be tightened immediately

There are signs of being defrauded or abnormal large or high-frequency transactions

Obvious mania, psychotic symptoms, and substance use problems

Discontinuation of medication or refusal to see a doctor, accompanied by impulsive consumption

Professional assistance

Physician/Psychologist: Assess decision-making ability, adjust treatment, and issue ability assessment opinions.

Finance/Law and Social Work: Establish compliant agency, guardianship or trusteeship mechanisms (regulations vary by region, on-site consultation is required).

One-sentence principle: Under the premise of ensuring safety, exchange the least restrictions for the greatest autonomy. When the condition is stable, delegate authority; when the risk increases, tighten it, and always carry out transparent, quantifiable and adjustable joint management.

**Q42What is the fastest way to find a person with mental illness who has gone missing?**

Here is a practical list of "shortest path and sequence" for you (applicable to emergency situations where patients with dementia/Alzheimer's disease, schizophrenia, bipolar disorder and other mental illnesses get lost). Doing it in sequence can significantly increase the recovery speed.

0-15 minutes: Things to do immediately

Report to the police (In Taiwan, dial 110; if you have any concerns about injury, you can also dial 119)

Clearly state: The patient's diagnosis, whether there is a history of suicide or impulsiveness, whether they bring medication, and whether they get lost.

Submit: recent clear photos, height and weight, hairstyle, clothing and shoes, personal belongings (mobile phone/Youyou card /iPASS/ UnionPay card), preferred routes, easy places to visit, and contact numbers of family members.

Apply for assistance in search notification and review of surveillance cameras at intersections (to be conducted by the police).

Locate everything that can be located

Mobile phones: Apple "Find" and "Family Share", Android "Find My Device".

Watches/wristbands /GPS bricks, in-car positioning, shared location groups.

Immediately save the location screenshot and timestamp and send them to the police.

Nearby "circular search" (home door → building → community → frequently visited by patients)

First, check the easily overlooked areas such as the stairwell, the top floor, the basement, public toilets, and refuge floors.

Convenience stores/supermarkets, temple entrances, parks and stations within the last one hour are preferred.

15-60 minutes: Synchronous diffusion

Traffic and station notifications

Station police station, MRT/Taiwan Railway/High Speed Rail service counter, bus station services, taxi fleet/ride-hailing platform (provide photos).

Inform about possible directions and clothing features, and leave a mobile phone number that can be dialed back.

Inspection of hospitals and shelters

Please make a phone call to the nearest emergency department, mental health hospital, nursing home, or police station for lost and found or temporary accommodation, and leave a photo with your name.

If there is a risk of self-harm, please mark it as a high-risk case and ask the other party to report it upon receiving a case with similar characteristics.

Community quick release (mainly private domain and community, pay attention to privacy when making public information)

LINE community groups, village head/management committee groups, neighborhood associations, local FB groups.

Send a "Search Assistance Card" (see the template below), and leave a photo, clothing, the last appearance point, the time, and the contact phone number. Avoid disclosing your full ID number or address.

1-6 hours: Data crossover and trajectory inference

Transaction/Access control/card trajectory

Recent transaction records of Youyou Card /iPASS, credit card/financial card (can help infer the movement route; police assistance if necessary).

Building access control, community camera timeline; Share possible intersections and time Windows with the police.

Community "targeted diffusion

Send private messages to stores, colleagues, religious sites or day care centers that the patient is familiar with.

Set up a unified contact person to avoid information omissions and harassing calls.

Expand the radius of physical search

Taking the last appearance point as the center, calculate the range that can be reached in 2 to 3 hours when the walker travels 2 to 4 kilometers per hour. Patrol along shady areas, under Bridges, bus shelters and 24-hour premises.

6-24 hours: Continuous tracking and media assistance

Go back to the police station to supplement the materials

New photos/clothing changes, the latest clues, location screenshots timeline; Please have the police update the notification.

Media and wider release (subject to risk determination)

For high-risk cases (loss of medication, intense delusions, extreme weather conditions), local media/radio assistance for search may be considered. Still avoid excessive exposure of privacy.

Key points for Communicating with the police/Passers-by (Brief and Useful)

Opening remarks: "This is a family member with mental illness. They might be lost and urgently need assistance now."

Characteristics: Height and body shape, clothing color, shoes, whether a mask/hat is worn, and whether speech is slow or self-talk.

Risks: Whether one is afraid of catching up, whether one might go further and further away, and whether there is a history of exposure/impulsiveness.

If you see someone: keep your distance, speak slowly, use affirmative sentences (" Let's go find a shady place to sit together "), avoid pulling, and call 110 immediately.

A directly replicable "Xie Xun Card" template

【 Search 】 Please help forward this

Name/Gender/Age:

Height, weight/body shape characteristics

Last show: YYYY-MM-DD HH:MM at (location)

Clothing: Top/coat/Pants/shoes/backpack

Personal belongings: Mobile phone (model/case color/whether it has location), cards (Youyou Card/credit card)

Disease warning: There is (Alzheimer's disease/schizophrenia, etc.), and one may get lost and be cautious of strangers

Please do not forcibly pull upon seeing this. Accompany me first and contact me immediately

Contact person: Name and Telephone: 09xx-xxx.-xxx

(Attach 1-2 clear photos)

Common Misunderstandings (Points that waste time)

Wait for 24 hours before reporting (incorrect) : High-risk cases can report to the police at any time and request assistance in search.

Only sharing on Moments: Be sure to follow the police, traffic and hospital notifications simultaneously.

Excessive and complex information: Designate a general contact person and a unified template.

Chasing or blaming: It is easy to trigger evasion/confrontation, leading to a second loss.

Post-event Prevention Checklist (Find Someone Faster Next Time)

Portable identification: waterproof identity card /QR search card, wristband, clothing sewing name tag.

Electronic fence: Mobile phones and watches share locations. Configure low-power consumption GPS for high-risk individuals.

Home environment: Door magnet/departure reminder, post "If you need assistance, please call ×××" on the door.

General Information Package: Prepare 5 clear recent photos from different angles, their features, a list of medications, and a list of frequently visited places.

**Q43As a family member of a person with mental illness, how can one relieve their own stress?**

Sure. Here's a set of "implementable and repeatable" stress-relieving practices for you first, suitable for family members who care for or accompany patients with mental illnesses. You don't have to do the full set at once. Just start with the easiest one or two items.

A list of self-relief and self-protection for family members

Establish a controllable daily/weekly routine

Three "stable anchor points" every day: getting up and going to bed regularly, walking or stretching for 10 to 15 minutes, and having a regular meal (neither taking care of nor eating at the same time).

"Emotional Health Check" Question 3: Is my current stress level between 0 and 10? Which part of the body is the tightest? What is one small thing I can do today?

Quick relaxation method: Square breathing (inhale for 4 seconds - pause for 4 seconds - exhale for 4 seconds - pause for 4 seconds, repeat for 2-3 minutes), 5-4-3-2-1 ground practice (say the visible/touchable/smelling/tasting/smelling things in sequence).

Draw the "boundary of responsibility"

Write down the list of "what I can be responsible for/what I cannot be responsible for", for example: I can accompany patients to the hospital, supervise medication reminders; I can't guarantee that the other person will always be emotionally stable, nor do I take on all the household chores. Align these boundaries with family members to avoid being exhausted by taking on everything.

Set up "pre-crisis - in-crisis - post-crisis" action cards

Before: Record the trigger factors and early warning signs (sudden changes in sleep, withdrawal, increased delusions/agitation, etc.).

Who will make the call, where to go, and where to put the medicine/health insurance card; Write the important contacts and their addresses on the same card and stick it in a fixed position.

After: A 24-48 hour recovery plan (catching up on sleep, having light meals, short walks, and only handling the most necessary household chores).

If there is a risk of self-harm or injury from others, or if you find it difficult to cope, please immediately seek help from local 24-hour resources (see resources at the end of the text). The Ministry of Health and Welfare's "Peace of Mind Hotline 1925" operates all year round. Lifeline 1995 and "Teacher Chang" 1980 also offer immediate psychological support. (Ministry of Health and Welfare

The "LEAP Sequence" for Communicating with Patients

Listen: First repeat what the other person has said (" I heard you feel... ") It's very tormenting." )

Empathize: Show understanding of emotions rather than arguing about the content.

Agree to a goal: Find a common small goal (sleep for six hours tonight first).

Partner: Break the task into two or three executable steps and set an appointment for the next checkpoint.

Arrange a "breathing window" for yourself

At least half a day of dedicated time per week (can be used in shifts with family members or through community respite/day care/home services).

In Taiwan, you can call the "1966 Long-Term Care Hotline" to learn about services such as respite care, transportation, and assistive devices. Family members can also use the "0800-50-7272 Family Caregiver Care Hotline" to obtain referrals for care plans and support groups. There are also maps of family caregiver support centers and resources in each county and city available for inquiry. (Ministry of Health and Welfare

Peer support and family groups

Regular communication with people who have had similar experiences can significantly reduce feelings of isolation and often help one learn more localized coping skills. You can look for mental health centers in each county and city, family support groups, or family groups and counseling provided by units related to depression prevention and treatment in Taiwan. (Ministry of Health and Welfare

Identify the red flag of "Caregiver exhaustion"

For more than two consecutive weeks: poor sleep, irritability, numbness, lack of enthusiasm for anything, frequent physical discomfort or a sense of hopelessness. When these arise, prioritize "self-protection" : shorten the care period, seek help from family members or external services promptly, and consider arranging your own psychological counseling (the list of cooperative institutions in each county and city can be found). (dep.mohw.gov.tw)

Immediate/Professional assistance in Taiwan (Save for Future reference)

24-hour peace of mind hotline: 1925 (Still love Me, free) (dep.mohw.gov.tw)

Lifeline: 1995 (Emotional Support/Suicide Prevention) (Life 1995)

"Teacher Zhang" Consultation: 1980 (Telephone and online/letter counseling; Please pay attention to service hours at each time slot) (Teacher Zhang Foundation)

Family Caregiver Care Hotline: 0800-50-7272 (Family Consultation, referral, Support Group) (Ministry of Health and Welfare)

Long-term Care Consultation and Respite: 1966 Long-Term Care Hotline (Service and Application Guidelines) (1966.gov.tw)

If you or your family members are at risk of acute self-injury or other injuries, please call the above-mentioned hotline or 110/119 immediately and try to stay in a safe environment for assistance.

**Q44If a child shows symptoms such as being reluctant to talk, irritable and disliking school, could there be mental or psychological problems?**

Short answer: It's possible.

When a child suddenly or persistently shows symptoms such as being taciturn, irritable, or disliking school, it may either be a temporary emotional or environmental response, or it could indicate mental and psychological or developmental and learning issues that require attention. The key lies in - duration, severity, and whether it affects learning/social/family functions.

Possible reasons (examples)

Emotional problems: Depression (low mood, loss of interest for ≥2 weeks), anxiety (excessive worry, physical discomfort, avoidance of going to school).

Attention and Behavior: ADHD (Distraction, impulsiveness), oppositional defiance.

Development-related: Autism spectrum (difficulty in social communication, stereotyped interests), language development or social communication disorders.

Learning difficulties: Obstacles in reading, writing and arithmetic learning, leading to aversion to study and emotional frustration.

Campus and family factors: bullying, interpersonal conflicts, academic pressure, tense parent-child interaction, major changes.

Physical and lifestyle issues: Insufficient sleep, excessive screen/gaming, chronic diseases and pain, thyroid/iron deficiency and other physical problems.

Feeling and Communication: Hearing/vision problems lead to less talking and avoidance.

The "Red Flag" that needs attention

If any of the following occurs, seek medical attention as soon as possible (child psychiatry/Psychosomatic/child health care/psychology) :

Obvious self-harming/suicidal thoughts or behaviors, and extremely desperate words

Significant depression or loss of interest lasting for ≥2 weeks, accompanied by obvious changes in sleep/appetite/energy

Significant decline in academic or social functions (such as a sudden drop in grades or a school refusal of ≥1 week)

Escalation of aggressive behavior, destruction of property, and running away from home

Retreat to the point of hardly communicating with anyone, or exhibit hallucinatory/delusional manifestations

Accompanied by physical emergencies such as fever, convulsions and severe headache

Quick self-check for parents (1 minute)

In the past month, if ≥3 items are "yes" and have affected study/family/peer relationships, it is recommended to assess:

Feeling down or anxious and tense for most of the day?

Significantly reduce talking or interacting with family and classmates?

Have you lost interest in an activity you once enjoyed?

Have you become more irritable, prone to outbursts or often talk back?

Obviously not wanting to go to school/frequently asking for leave/avoiding classes?

Poor sleep (difficulty falling asleep, early awakening) or disrupted sleep schedule?

Screen usage has significantly increased and is hard to stop?

Have there been any recent school or family stress events?

"Current Practices in Parent-Child Relationships and Schools

Empathize first, then ask the question: Find a quiet period and use an open-ended question - "Which thing has been bothering you the most recently?" Avoid pressing or blaming.

Stable sleep schedule and stress relief: Regular sleep, daily outdoor activities and exercise, limit screen time at night, and set small achievable goals.

Communicate with the school: Understand classroom performance and peer relationships, and ask if there can be phased academic burden reduction/support (such as seat adjustment, task decomposition).

Record changes: Simple logs (emotions/sleep/school performance/triggers) to provide clues during medical visits.

Check for physical factors: If necessary, conduct visual and hearing tests, basic physical examinations and tests (such as blood routine, iron, thyroid function, etc.) - Many emotional/learning problems are related to health.

When to consider professional assessment and treatment

The problem persists for more than 4 weeks and remains obvious. Or although less than 4 weeks, the intensity is high/the functional impairment is significant.

There are long-term concerns about development/learning (language, social skills, reading, writing and arithmetic).

The situation has not improved even after investigating bullying or family conflicts.

Treatment may include: parental guidance and parent-child training, psychotherapy (such as CBT/ play therapy), school support programs, and medication when necessary (determined by specialist assessment).

Medical Visit Preparation List (Bring it for Better Efficiency)

Timeline: When symptoms began, when they worsened, and the causes.

Functional impacts: Changes in academic performance, absence from classes, and getting along with peers/family members.

Schedule and screen: Sleep, exercise, usage duration and content.

Family history and past medical history: Emotional/developmental/learning issues, medication.

School feedback and sample works/assignments.

**Q45Can pregnant women or those preparing for pregnancy take psychiatric drugs?**

Sure, but it's necessary to "weigh the pros and cons, make individualized decisions, and follow up in a standardized manner". Many psychiatric drugs can be continued during pregnancy or the preconception period or initiated after assessment. Untreated moderate to severe depression, anxiety, bipolar or psychotic disorders themselves significantly increase the risk of premature birth, low birth weight, pregnancy complications, suicide and poor maternal and infant outcomes. Therefore, it is generally not recommended to stop taking the drugs rashly for the sake of "zero medication". Professional societies (such as the American College of Obstetricians and Gynecologists (ACOG)) recommend that prenatal plans be jointly developed with obstetrics and psychiatry, using single drugs at the lowest effective dose as much as possible, and regular prenatal check-ups and drug blood concentration/adverse reaction monitoring be conducted. (American College of Obstetricians and Gynecologists

General Principles (Preconception and pregnancy)

Don't stop taking the medicine or change it by yourself. ACOG does not recommend the random discontinuation of mood stabilizers (except valproic acid) during pregnancy to avoid recurrence and aggravation. (Project Teach NY)

Weigh "disease risk vs. medication risk". A large amount of evidence shows that most selective serotonin reuptake inhibitors (SSRIs) are generally safe during pregnancy and do not increase the risk of major congenital malformations. Stopping effective drugs often does more harm than good. (American College of Obstetricians and Gynecologists

Non-pharmaceutical intervention should be given priority for mild cases. For mild to moderate cases, psychological treatment (such as CBT and IPT) can be the first choice. Moderate to severe cases or a history of recurrence usually require a combination of medication and psychotherapy. Both NICE and ACOG hold this framework. (NICE)

Preconception period planning. If it is necessary to adjust to a safer and more effective drug, it is best to complete and stabilize it for a period of time before conception. For those using antiepileptic/mood stabilizers, folic acid (usually at a higher dose) should be added during the preconception period and strict contraception should be implemented until the protocol is determined. (The specific dosage for each country should follow the doctor's advice.)" (American College of Obstetricians and Gynecologists

Key points of each drug category

Antidepressants (SSRIs/SNRIs, etc.)

Available: Overall evidence supports the safety of SSRIs during pregnancy; Commonly used ones include sertraline and escitalopram, etc., on the front line of clinical practice. It should be noted that in the third trimester of pregnancy, there may be transient adaptation problems in newborns and a slightly increased risk of bleeding during pregnancy and childbirth. The obstetric team will conduct corresponding monitoring. (American College of Obstetricians and Gynecologists

How to choose: If a certain SSRI has shown clear efficacy and tolerance in the past, continuing with the original drug is usually better than changing the drug. When taking the medicine for the first time, those with more sufficient evidence or lactation data (such as sertraline) are often chosen. (American College of Obstetricians and Gynecologists

2) Benzodiazepines (anti-anxiety/sedative)

If possible, avoid using it or use it in small amounts for a short period: ACOG suggests avoiding or using it with caution, using it for a short period, and weighing the risks of dependence, sedation, rebound after drug withdrawal, and neonatal adaptation issues. (Project Teach NY)

3) Mood stabilizers

valproate: Strongly not recommended or contraindicated during preconception and pregnancy (significantly increases the risk of neural tube defects and developmental problems). If there is no alternative and it must be used, many countries require "Pregnancy prevention programs (PPP)" and strictly enforce contraception and regular evaluations. (GOV.UK)

lithium: It can be used under strict monitoring (regular blood lithium and kidney/thyroid function monitoring is required; clearance rate varies greatly during pregnancy, especially in the third quarter; fetal echocardiography assessment is recommended). Evidence of a slight increase in the overall risk of deformity varies, and decisions need to be individualized. (American College of Obstetricians and Gynecologists

lamotrigine: Currently, the pregnancy data is relatively more favorable, but the clearance rate during pregnancy has increased, and it is often necessary to increase the dose and monitor it. (American College of Obstetricians and Gynecologists

Carbamazepine/topiramate, etc. : There is a considerable amount of evidence associated with the risk of deformity. Alternative options should be evaluated during the preconception period. (American College of Obstetricians and Gynecologists

4) Antipsychotic drugs (mainly the second generation)

5) Large-scale studies and systematic reviews have shown that commonly used quetiapine, olanzapine, etc. have not observed an overall increased risk of congenital malformations. However, it may increase the weight/metabolic burden during pregnancy, and monitoring of gestational diabetes and weight is required. It is usually not recommended to change the medication during pregnancy when it is stable and effective. (The Lancet

5) Other options

Electroconvulsive therapy (ECT) : It can be safely implemented during pregnancy in a multidisciplinary team for patients with severe depression or psychotic symptoms who do not respond to medication or require rapid response. The guiding opinions list it as an option. (American College of Obstetricians and Gynecologists

Practical Checklist (For Those Planning to conceive or Already Pregnant)

Inform the obstetrics and psychiatry department as soon as possible of all the medications you are currently (or plan to) taking and their dosages, and bring along your previous medical records. (American College of Obstetricians and Gynecologists

Don't stop taking the medicine by yourself. If adjustments are needed, they should be completed first before conception and the stability should be observed. (American College of Obstetricians and Gynecologists

Monotherapy is superior to combination therapy. Use the lowest effective dose, follow up regularly and conduct laboratory monitoring (measure blood drug concentration if necessary). (American College of Obstetricians and Gynecologists

If you are taking valproic acid or other high-risk medications, immediately discuss alternative options and contraception /PPP with your doctor. (GOV.UK)

Prenatal checkup collaboration: Pay attention to blood pressure, blood sugar, and weight; For those using SSRI/SNRI, attention should be paid to postpartum hemorrhage and short-term observation of newborns during delivery. (South East London ICS)

Delivery and postpartum: Most pregnancies can be treated with medication, and there are also good records during lactation (such as sertraline), but individualized assessment and pediatric observation are still required. (NHS Somerset ICB)

One-sentence conclusion: Psychiatric drugs can be used during pregnancy or the preconception period under professional assessment. Many drugs (especially SSRIs, some antipsychotic drugs, lamotrigine, etc.) are generally safe during pregnancy, while valproic acid should be avoided. The key is to work with the obstetrics and psychiatry team to develop individualized plans, conduct continuous monitoring, and never stop taking the medication or change it at will. (American College of Obstetricians and Gynecologists

**Q46Can psychiatric drugs be taken simultaneously with other medications?**

Sure, but it must be after assessment by a doctor or pharmacist. Common interactions between psychiatric medications (such as antidepressants, anti-anxiety drugs, mood stabilizers, antipsychotic drugs, etc.) and other prescription drugs, over-the-counter drugs, and health supplements can either weaken therapeutic effects or cause serious adverse reactions. The following are the most important combinations to pay attention to (only the key points are listed) :

High-risk/avoidable combinations

Opioid painkillers combined with benzodiazepines (such as alprazolam and lorazepam) : Significantly increase the risk of respiratory depression and death. The FDA has issued the strongest "box warning". (U.S. Food and Drug Administration)

Serotonin-stimulating drugs such as SSRIs/SNRIs + other serotonin-stimulating drugs (triptan, MAOI, some cough suppressants dextromethorphan, etc.) : can cause serotonin syndrome (high fever, muscle rigidity, changes in consciousness). (FDA Access Data)

MAOI (phenylhydrazine, transbenzylcyclopropylamine; or high-dose MAO-B inhibitors for Parkinson's disease) + sympathomimetic drugs/congestion reducers (pseudoephedrine, etc.) or tyramine-rich foods: can cause hypertensive crisis. (Mayo Clinic)

Lithium salts + NSAIDs (such as ibuprofen) /ACEI/ diuretics: Increase lithium concentration and raise the risk of poisoning (tremor, ataxia, kidney damage). Blood lithium needs to be monitored and the dosage adjusted as appropriate. (nhsborders.scot.nhs.uk)

Valproic acid + lamotrigine: Significantly increases the risk of severe rashes. The dosage of lamotrigine should be greatly reduced and gradually increased. (FDA Access Data)

Carbamazepine liver enzyme inducers combined with hormonal contraceptives: Reduce contraceptive effectiveness. It is recommended to switch to an intrauterine device or add alternative methods as per the guidelines. (SPS - Specialist Pharmacy Service)

The use of multiple antipsychotic drugs (or in combination with certain antibiotics/antifungal/antiarrhythmic drugs) : increases the risk of QT prolongation/torsional ventricular tachycardia at the tip. Checklist and electrocardiogram assessment are required. (PMC)

Clozapine + drugs that affect CYP114A2/3A4/2D6 or changes in smoking status: The dosage needs to be adjusted according to the interaction (such as combined with fluvoxamine/ciprofloxacin, etc.). (FDA Access Data)

St. John's wort (Forsythia, a health supplement) + antidepressants/contraceptives/anti-rejection drugs/anticoagulants, etc. : It significantly weakens the efficacy of many prescription drugs by inducing CYP3A4 and can co-induce serotonin syndrome with antidepressants. (NCCIH)

The general principle of safety and suitability

Before adding any new medications (including Chinese patent medicines, health supplements, and cold medicines), consult first and make a list of all the medications you are currently using for your doctor/pharmacist.

Fix one pharmacy for dispensing medicine to facilitate the system's automatic interception of interactions.

Pay attention to warning symptoms: sudden drowsiness, slow breathing (opioid + benzodiazepine), high fever and sweating/muscle stiffness/changes in consciousness (serotonin syndrome), palpitations and syncope (QT prolongation), ataxia/vomiting/thirst and polyuria (lithium poisoning), etc. Seek medical attention immediately if any of these symptoms occur. (U.S. Food and Drug Administration)

Regular monitoring: For example, when using lithium, blood lithium and renal function should be checked. When drugs that may prolong QT are used in combination, an electrocardiogram should be performed. The initiation and escalation of lamotrigine slow down as per the instructions. (tewv.nhs.uk)

Do not stop taking medication on your own (especially antidepressants, benzodiazepines, and antipsychotic drugs) to avoid rebound or withdrawal syndrome. When dressing changes are needed, the doctor will cross-reduce or discontinue the dosage.

Avoid using alcohol in combination with sedatives (as it may aggravate drowsiness, falls and respiratory depression). (U.S. Food and Drug Administration)

**Q47Can one drink alcohol, coffee or strong tea during the period of taking psychiatric medication?**

Let's make the conclusion clear below (first explain "can it be", then "why/what to pay attention to") - applicable to anyone who is currently taking any psychiatric medication; There are significant individual differences. The final opinion should be based on that of your attending physician/pharmacist.

Alcohol (beer, wine, spirits)

Principle: Try not to drink.

Aggravating drowsiness and slowing down reactions: Sedatives (such as benzodiazepines like lorazepam/alprazolam), most antipsychotic drugs, mirtazapine/trazodone, etc., when combined with alcohol, are prone to falls, car accidents, and respiratory depression.

Emotion and suicide risk: Alcohol itself is a depression trigger, offsetting the effects of antidepressant treatment.

Liver burden: Drugs metabolized by the liver, such as valproic acid, carbamazepine, and fluvoxamine, together with alcohol, increase the risk of liver toxicity.

Dehydration/electrolyte issues: When lithium salts encounter alcohol, vomiting/diarrhea or excessive sweating, lithium poisoning is prone to occur (hand tremors, drowsiness, slurred speech, ataxia).

Blood pressure/Heart rate: When used in combination with stimulants (methylphenidate, amphetamine), it can mask intoxication, increase heart rate and blood pressure, and raise the risk of cardiovascular events.

MAOI (Monoamine Oxidase Inhibitor) Special note: Avoid alcoholic beverages rich in tyramine (draft beer, unpasteurized beer, and some aged wines), as they may induce hypertensive crisis.

If you really want to drink: After obtaining a doctor's permission, drink only a very small amount (such as ≤1 cup of standard alcohol) and do not take it at the same time as the medication. Replenish water before and after drinking and do not drive.

Coffee/strong tea (mainly caffeine)

Principle: Most people can "drink in small amounts, disperse and during the day". Stop when you feel unwell.

Aggravating anxiety and insomnia: Anyone prone to anxiety or insomnia should reduce or avoid caffeine, especially not drinking it within 6 to 8 hours before going to bed.

Interactions with specific drugs:

Clozapine and olanzapine: Caffeine can increase blood drug concentration, which may cause more drowsiness and palpitations. If you suddenly stop drinking coffee, the drug concentration will drop and the symptoms may rebound.

Fluvoxamine (an antidepressant) : It strongly inhibits CYP1A2 and can significantly increase caffeine levels in the blood → palpitations, hand tremors, and insomnia. It is recommended to drink as little as possible or not at all.

Lithium salts: Caffeine can enhance the removal of lithium. Regular consumption → low lithium levels. Suddenly stopping coffee can lead to an increase in lithium levels. Both situations may cause problems. Keep your coffee intake stable and inform your doctor if there are any changes.

Stimulants (ADHD medication)/bupropion (bupropion) : When combined with caffeine, it can aggravate palpitations, elevated blood pressure, and hand tremors. High doses of caffeine may also increase the risk of epilepsy (especially for bupropyl).

MAOI: Large amounts of caffeine may cause blood pressure fluctuations. It is safer to keep it at a low to moderate level.

Reference amount: For most people, it is safer to consume no more than 200 mg of caffeine per day (about 1-2 cups of hand-brewed or Americano or 2-3 cups of light tea). It should be lower or even avoided for those with insomnia/anxiety/heart discomfort.

When it comes to strong tea, one still needs to be cautious

Strong black tea or green tea taken together with iron supplements can affect absorption. If you are iron deficient due to medication and need to take iron supplements, there should be a 2-hour gap.

The tea polyphenols and tannic acid themselves are not a big problem. The main issue lies in the caffeine effect.

Practical Practices (for those taking medication)

First, ask your doctor: Please inform us of the names and dosages of the medications you are currently taking (especially: lithium salts, clozapine/olanzapine, valproic acid, carbamazepine, MAOI, fluvoxamine, benzodiazepine, stimulants, bupropyl).

If the doctor permits a small amount of alcohol consumption: limit it to occasionally and in small amounts, and avoid taking it with medication. Any dizziness, unsteady gait or palpitations should be stopped and reported.

Coffee/tea: Start with half a cup, drink only during the day, and observe if you feel palpitations/poor sleep/more anxious. Once you feel unwell, reduce the dosage or stop.

Maintain stable habits: Do not fluctuate in caffeine intake for lithium salts, clozapine, fluvoxamine, etc. If you want to make any changes, tell your doctor first.

Seek medical attention immediately if any of the following conditions occur: confusion, severe drowsiness or restlessness, persistent palpitations and chest tightness, high fever with excessive sweating, severe headache/sudden surge in blood pressure, hand tremors or ataxia (be alert to lithium poisoning/serotonin syndrome/hypertensive crisis, etc.).

**Q48What are the dietary restrictions for patients with mental disorders during the medication period?**

The following list compiles "points to note in diet when taking medication" by common categories of psychiatric medications. (Different medications vary greatly. Be sure to follow the instructions on your prescription and consult your doctor/pharmacist.)"

General

Alcohol: Most antidepressants, anti-anxiety drugs and antipsychotic drugs have a combined sedative effect with alcohol, affecting judgment and the risk of falls. In principle, it is advisable to avoid drinking alcohol.

Grapefruit/Grapefruit juice: It can inhibit CYP3A4 and increase the concentration of various psychotropic drugs in the blood. The effects of quetiapine and carbamazepine have been observed. Both manufacturers and clinical reviews recommend their avoidance. (Drugs.com)

Caffeine (coffee, tea, energy drinks) : Can aggravate anxiety/insomnia; There is a significant interaction with certain drugs (see below).

Health supplements/herbs: Especially St. John's wort, when used in combination with SSRI/SNRI, etc., can cause serotonin syndrome. It should be avoided to use them together on one's own. (Cleveland Clinic)

Specifically, in terms of drug category/drug name

Monoamine oxidase inhibitors (MAOI, such as phenylhydrazine, trans-phenylcyclopropylamine, etc.)

A strict "low-tyramine diet" must be followed: avoid aged/fermented/pickled/spoiled foods (such as aged cheese, sausages/salami, fermented soy products, kimchi, overripe bananas/avocados, red wine, home-brewed beer, etc.) to prevent hypertensive crisis. Attention should still be paid at least two weeks after stopping the medication. (Tobe and South Devon NHS Foundation

2) Lithium salts (lithium carbonate)

The salt content and drinking water should be stable: Sudden low salt intake or dehydration can increase lithium concentration and cause poisoning. Excessive salt intake or sudden irrigation of water may also reduce the therapeutic effect. Maintain a stable daily salt intake and adequate hydration (especially when sweating a lot, having a fever or diarrhea).

Caffeine intake should be "constant" : Changing caffeine habits (sudden increase or sudden stop) can affect lithium concentration. (hpft.nhs.uk)

3) Second-generation antipsychotic drugs (quetiapine, aripiprazole, olanzapine, clozapine, lurasidone, ziprasidone, etc.)

Grapefruit/Grapefruit juice: It should be avoided for CYP3A4 metabolizing drugs such as quintiapine. (Drugs.com)

Special requirements for taking with food:

ziprasidone should be taken with at least 500 kcal of main meals for stable absorption. Low-calorie meals or fasting can significantly reduce absorption. (PubMed)

lurasidone should be consumed with meals and at least about 350 kcal. (FDA Access Data)

Caffeine and clozapine/olanzapine: Caffeine can inhibit CYP1A2 and increase clozapine levels; A significant change in caffeine habits may require an adjustment in dosage. (PMC)

Weight/blood sugar/blood lipid: Such drugs often cause metabolic abnormalities. It is recommended to consume less sugar and refined carbohydrates, control calorie intake, and exercise regularly. (FDA Access Data)

4) Antidepressants

SSRIs/SNRIs and grapefruit: Some medications (such as sertraline and fluvoxamine) are not recommended to be used in combination with grapefruit to avoid elevated blood levels. (sunnybrook.ca)

Contraindicated in combination with St. John's wort (risk of serotonin syndrome). (Cleveland Clinic)

Caffeinated beverages: They can aggravate anxiety, palpitations and insomnia in some people. If fluvoxamine is used in combination, the caffeine level will increase significantly and needs to be restrained even more. (sunnybrook.ca)

Other tips

Regular, light and balanced: Prioritize fresh food and avoid overeating and extreme diets.

Read the sections of "Administration and Food" and "Interactions" on the drug instructions; If the instructions on your medication state "Should be taken with meals/avoid grapefruit/limit tyramine-containing foods", you must strictly follow them. The ziprasidone/lurasidone /MAOI/ lithium mentioned above are the most common representatives that are "strongly associated with diet". (PubMed)

**LLM Model:Claude Sonnet 4.5**

**Q1. What is mental illness?**

Mental illness (Mental Illness/Mental Disorder) refers to a category of health conditions that impair an individual's thinking, emotions, behavior, and social functioning, causing significant distress or impairment in daily life.

leading feature

Mental illness usually has the following characteristics:

Abnormal thinking: such as confusion, delusions, obsessive thoughts, etc

Emotional disorders: such as persistent sadness, anxiety, mood swings, etc

Behavioral changes: social withdrawal, impulsive behavior, self-harm, etc

Function impairment: affects daily functions such as work, study, and interpersonal relationships

Common types

Mental disorders: depression, bipolar disorder

Anxiety disorders: generalized anxiety disorder, phobia, panic disorder

Schizophrenia spectrum disorder

Obsessive-compulsive disorder and related disorders

Trauma and stress-related disorders: PTSD (Post-Traumatic Stress Disorder)

Eating disorders: anorexia, bulimia

substance use disorders

Neurodevelopmental disorders: Autism spectrum disorder (ASD) and Attention Deficit Hyperactivity Disorder (ADHD)

cause of formation

The causes of mental illness are complex and usually involve a number of factors:

Biological factors (genetics, brain chemistry imbalance)

Psychological factors (traumatic experiences, stress)

Social environmental factors (life events, interpersonal relationships)

therapeutic method

Medication: antidepressants, anti-anxiety drugs, etc

Psychotherapy: cognitive behavioral therapy, psychodynamic therapy, etc

Social support: Family support, mutual aid groups

Lifestyle adjustments: exercise, regular sleep, stress management

Important Notice

✓ Mental illness is a real medical condition, not a sign of character flaws or weakness

✓ Most mental illnesses can be treated

✓ It's important to seek professional help early

Patients with mental illness should not be discriminated against or prejudiced

If you or someone you know is experiencing mental health problems, it is recommended to consult a professional psychiatrist or psychotherapist.

**Q2. What are the common types of mental illness?**

There are many types of mental illness (psychological disorder), and here are some common classifications:

1. Mood disorders

Depression: persistent sadness, loss of interest, fatigue, low self-worth

Bipolar disorder: mood swings between mania and depression

Persistent depressive disorder: a long-term state of mild depression

2. Anxiety disorders

Generalized anxiety disorder: persistent excessive worry

Panic disorder: recurrent panic attacks

Social anxiety disorder: intense fear of social situations

Specific phobia: irrational fear of specific things

Obsessive-compulsive disorder (OCD): obsessive thoughts and compulsive behaviors

3. Psychotic disorders

Schizophrenia: hallucinations, delusions, disordered thinking

Dissociative affective disorder: psychotic symptoms with mood disorder

Delusional disorder: persistent delusions but relatively intact function

4. Trauma and stress-related disorders

Post-traumatic stress disorder (PTSD): A persistent reaction following traumatic events

Acute stress disorder: a strong reaction in the short term after trauma

5. Eating disorders

Anorexia nervosa: extreme restriction of food

Neurotic bulimia: clearing behavior after binge eating

Binge eating disorder: repeated binge eating without purging behavior

6. Personality disorders

Borderline personality disorder: emotional instability, impulsiveness, interpersonal problems

Antisocial personality disorder: disregard for the rights of others

Avoidant personality disorder: social inhibition, low self-esteem

7. Neurodevelopmental disorders

Attention Deficit Hyperactivity Disorder (ADHD): characterized by inattention, hyperactivity, and impulsivity

Autism spectrum disorder: social communication difficulties, repetitive behaviors

8. Substance-related disorders

Substance dependence and abuse: addiction to alcohol, drugs, etc.

Important reminder:

✅ Mental illness is a treatable medical condition

✅ If symptoms occur, seek help from a professional psychologist or psychiatrist

✅ Early intervention is generally more effective

✅ Treatment options include psychotherapy, medication, or a combination of both

**Q3. Is anxiety a mental illness?**

Yes, anxiety disorder belongs to the category of mental illness (mental disorder).

specify ：

Medical classification

Anxiety disorders are explicitly classified as mental disorders in both the International Classification of Diseases (ICD) and the Diagnostic and Statistical Manual of Mental Disorders (DSM) of the United States.

It is a major category of mental illness

Common anxiety disorders include:

Generalized Anxiety Disorder (GAD)

Panic disorder (panic attack)

Social anxiety disorder (social phobia)

Specific phobia

Separation anxiety disorder

agoraphobe

Important note:

Mental illness is not the same as mental illness

Mental illness: a broad concept that includes anxiety, depression, obsessive-compulsive disorder, and other mental health problems

Psychosis: usually refers to severe mental disorders, such as schizophrenia, patients may have hallucinations, delusions and other symptoms

Anxiety disorders can be treated

Through psychotherapy (such as cognitive behavioral therapy)

Combine with drug therapy if necessary

Most patients are able to improve effectively

If you or someone you know is experiencing anxiety, it is recommended to seek help from a professional psychologist or psychiatrist. Anxiety disorder is a common mental health problem, and active treatment can significantly improve the quality of life.

**Q4. Is depression a mental illness?**

Yes, depression is a mental illness.

specify ：

Medical classification

Depression is medically classified as:

A type of mental disorder

More specifically, it falls under the category of mood disorders.

Both the International Classification of Diseases (ICD) and the Diagnostic and Statistical Manual of Mental Disorders (DSM) provide clear definitions.

Why mental illness?

Depression is characterized by mental illness:

Affecting psychological functions-including emotions, thinking, cognition and behavior

Biological basis-involves an imbalance of neurotransmitters in the brain (such as serotonin, dopamine)

Professional diagnosis and treatment required-diagnosed by a psychiatrist or psychologist

Medical intervention is possible-both medication and psychotherapy are effective

Important:

Mental illness = 'psychosis' -many people confuse these two concepts. 'Psychosis' usually refers to a severe mental disorder (such as schizophrenia) with symptoms such as hallucinations and delusions, while depression is a mental illness, but usually does not belong to the category of 'psychosis'.

Depression is treatable-through standardised treatment, most patients can recover

There should be no stigma-depression is a disease like high blood pressure and diabetes, and needs to be addressed and treated

If you or someone you know has symptoms, seek professional medical help.

**Q5. What are the main causes of mental illness?**

The occurrence of mental illness is a complex process, usually caused by the interaction of a variety of factors, mainly including the following aspects:

1. Biological factors

Genetic factors: Family history is an important risk factor, and certain mental disorders (such as schizophrenia and bipolar disorder) have a clear genetic predisposition

Neurochemical abnormalities: Imbalance of neurotransmitters (e.g., dopamine, serotonin, noradrenaline, etc.)

Abnormal brain structure and function: structural changes or dysfunctions in specific areas of the brain

Hormone level changes: such as postpartum depression is associated with hormone fluctuations

Neurodevelopmental problems: abnormalities during fetal or early development

2. Psychological factors

Traumatic experiences: childhood trauma, abuse, neglect, etc

Major life events: stressful events such as the death of a loved one, divorce, unemployment, etc

Personality traits: certain personality traits may increase susceptibility

Cognitive patterns: negative thinking, cognitive distortion

How to cope: Lack of effective coping mechanism for stress

3. Social and environmental factors

Family environment: family relationship tension, poor communication, improper parenting style

Social support: Lack of social support network

Socio-economic status: poverty, unemployment, social inequality

Cultural factors: the influence of cultural background and social values

Life stress: long-term work stress, academic stress, etc

4. Other factors

Physical illness: Some physical illnesses may cause or worsen mental symptoms

Drug and substance use: alcohol, drug abuse

Nutritional status: Certain nutritional deficiencies may affect mental health

Sleep problems: Chronic sleep disorders

Biopsychosocial model

Modern psychiatry widely adopts the ** "biopsychosocial" integrated model** to understand mental disorders, which holds that these factors do not act in isolation but interact and work together. Each individual's condition is unique, and the weight of different factors may vary.

If you or someone close to you has mental health problems, it is recommended to seek professional psychological counseling or psychiatric help in time.

**Q6. Does insomnia or staying up late for a long time increase the risk of mental illness?**

The relationship between insomnia and chronic sleep deprivation and the risk of mental illness

Short answer: Yes, insomnia and chronic sleep deprivation significantly increase the risk of multiple mental disorders.

Increased risk

1. Depression

Insomniacs are 2-4 times more likely to develop depression than normal people

Chronic sleep deprivation can affect the balance of neurotransmitters such as serotonin and dopamine in the brain

Chronic insomnia may be an early sign or trigger for depression

2. Anxiety disorders

Sleep deprivation increases amygdala activity (the emotional center)

Reduce the prefrontal cortex's ability to regulate emotions

Resulting in increased risk of excessive worry and panic attacks

3. Bipolar disorder

Sleep disturbances may trigger manic or depressive episodes

The circadian rhythm disorder is closely related to the fluctuation of the disease

4. Schizophrenia

Chronic sleep disturbances may increase the risk of psychotic symptoms

In high-risk populations, it may accelerate disease progression

mechanism of action

🧠 Neurobiology impact

Decreased neural plasticity: affects the brain's ability to learn and regulate emotions

Neuroinflammation: Chronic sleep deprivation triggers inflammatory responses that damage neurons

Neurotransmitter imbalance: dopamine, serotonin, norepinephrine secretion disorder

⏰ Irregular circadian rhythm

Melatonin secretion abnormality

Cortisol (stress hormone) rhythm disorder

Affects mood and cognitive function

🔄 Stress Response System

Overactivation of the HPA axis (hypothalamic-pituitary-adrenal axis)

In a state of stress for a long time

Reduced ability to cope with stress

bidirectional relationship

It's important to note that sleep problems and mental illness are two-way streets:

Insomnia/staying up late ↔ mental illness

Sleep disorders can lead to mental illness

Mental illness is also often associated with sleep problems

Create a vicious circle

Other health risks

Chronic sleep deprivation also increases:

Cognitive decline (memory, attention, decision-making)

angiocardiopathy

Metabolic disorders (diabetes, obesity)

Immune function decline

propose

✅ Ways to improve sleep:

Regular sleep schedule: Go to bed and wake up at the same time every day

Sleep hygiene: Avoid screens, caffeine, and intense exercise before bed

Comfortable environment: Quiet, dark, cool bedroom

Relaxation techniques: Meditation, deep breathing, progressive muscle relaxation

Limit daytime naps to 30 minutes

When to see a doctor:

Insomnia lasts more than 3 months

严重影响 daily functions

Symptoms include low mood and anxiety

Self-regulation is ineffective

Bottom line: Insomnia and chronic sleep deprivation significantly raise the risk of mental health issues, making it essential to maintain healthy sleep habits. If you're experiencing persistent sleep problems, it's advisable to seek professional help promptly.

**Q7. How to tell if you may have a mental illness?**

There are several things to consider when determining whether you may have a mental illness. Here are some important warning signs:

Emotional and psychological

Persistent mood changes: feeling sad, anxious, empty, or hopeless for more than two weeks

Extreme mood swings: intense mood swings that are difficult to control

Loss of interest: Loss of interest and enthusiasm for activities you used to enjoy

Excessive worry or fear: Uncontrollable anxiety and panic

behavior change

Social withdrawal: avoiding friends, family, and social activities

Sleep problems: insomnia, drowsiness, or significant changes in sleep patterns

Dietary changes: significant increase or decrease in appetite and significant change in weight

Difficulty with daily tasks: a marked decline in the ability to work, study, or live

cognitive function

Attention and memory problems: difficulty concentrating, memory decline

Confused thinking: the logic of thinking is confused and it is difficult to express clearly

Perception anomaly: hallucinations or delusions

Physical symptoms

Unexplained physical symptoms: Headache, stomachache, fatigue, etc.

Decreased energy: constant fatigue and weakness

Other warning signals

Self-harm or suicidal thoughts: thoughts or actions that harm oneself

Drug or alcohol dependence: using substances to cope with emotions

Disconnection from reality: feeling disconnected from the environment or from yourself

Important reminder

Don't self-diagnose: The above symptoms can be caused by a variety of causes, and only a professional doctor can make an accurate diagnosis.

✅ When to ask for help:

Symptoms last more than two weeks and interfere with daily life

Thoughts of suicide or self-harm

Unable to cope with daily stress

People around expressed concern

Action suggestion

Consult a professional: psychologist, psychiatrist, or counselor

Talk to someone you trust: family, friends, or teachers

Use mental health hotlines: Many areas have 24-hour psychological assistance hotlines

Keep a record: Record symptoms, triggers, and duration to help describe them during your visit

Remember, seeking help is a brave and wise choice, and early intervention often works better. Mental health and physical health are equally important and deserve serious attention.

**Q8. Can the self-assessment form accurately determine whether you have a mental illness?**

Self-assessment tools cannot accurately determine whether a person has a mental illness and can only be used as a preliminary screening tool. Here are the reasons:

Limitations of self-assessment

1. Lack of professional assessment

In-depth clinical interviews are not possible

Unable to observe nonverbal behavior and emotional expressions

Lack of accurate assessment of symptom severity and duration

2. Strong subjectivity

Depending on self-knowledge and judgment, there may be bias

Your mood can affect your answers (e.g. you may overestimate symptoms when anxious)

Lack of medical knowledge may lead to misunderstanding the meaning of the question

3. Other factors cannot be ruled out

Unable to distinguish psychological symptoms caused by physiological diseases

The effects of drugs and substance use cannot be considered

It is difficult to distinguish between normal mood fluctuations and pathological symptoms

Diagnostic criteria

A diagnosis of mental illness requires:

✓ Professional doctor consultation-Psychiatrists conduct detailed interviews

✓ Standardized diagnostic criteria-such as DSM-5 or ICD-11

✓ Comprehensive assessment-Medical history, symptoms, and degree of functional impairment

✓ Ancillary tests when necessary-to rule out organic diseases

✓ Time dimension considerations-duration of symptoms and development pattern

Value of self-measuring meter

There is no definitive diagnosis, but there are positive effects:

📊 Raise mental health awareness

🔍 Find clues to potential problems

💡 Decide whether you need professional help

propose

If the self-test results are abnormal:

Don't self-diagnose or label

Seek professional help promptly-visit the psychiatry or psychology department at a certified medical institution

Stay objective-Use test results as a reference, not a diagnostic basis

Remember: Only a qualified psychiatrist can make an accurate diagnosis.

**Q9. Which department should I visit if I suspect a mental illness?**

When you suspect a mental illness, you should see the following departments:

Main treatment department

1. Psychiatry/Psychiatry

This is the most relevant department

Specializes in the diagnosis and treatment of various mental disorders, such as depression, anxiety, schizophrenia, bipolar disorder, etc.

2. Psychology/Counseling Clinic

Suitable for mild psychological problems

Provide psychological assessment and psychotherapy

Department names of different medical institutions

Different hospitals may have different names:

General hospital: psychiatry, psychosomatic medicine

Specialized hospitals: Mental health centers, psychiatric hospitals (with specialized departments)

Community hospital: Psychological counseling clinic

Visit advice

First visit: You can first schedule an appointment at the general outpatient clinic of psychiatry or psychology

Documents: Please bring any previous medical reports, medication records, etc.

Describe symptoms: Describe symptoms in detail, including how long they last and how they affect your life

Accompanying family members: If possible, it is recommended that family members accompany you to the clinic

exceptional case

Emergency (if you have a tendency to self-harm or harm others): Go directly to the emergency department or call 120

Adolescent patients: Some hospitals have a child and adolescent psychiatry

If you are not sure which department to go to, you can also ask at the hospital desk, the staff will give you guidance.

**Q10.How to distinguish mental illness from other physical illness?**

How to distinguish mental illness from other physical illness

This is an important and complex question, because the line between mental and physical illness is not always clear-cut. Here are some key points to distinguish:

I. Main Differences

1. Focus of symptom presentation

Mental illness: affects thinking, emotions, cognition and behavior

Emotional abnormalities (depression, anxiety, mania)

Cognitive impairment (confusion, delusions, hallucinations)

Behavioral abnormalities (social withdrawal, impulsive behavior)

Physical disease: mainly affects the body organs and physiological functions

Pain, fever, inflammation

Organ dysfunction

Physiological parameter changes

2. Diagnostic methods

mental sickness ：

It relies mainly on clinical interviews and symptom assessment

Psychological tests and scales

Diagnosis after excluding organic causes

physical illness ：

Laboratory tests (blood, urine, etc.)

Imaging studies (X-ray, CT, MRI)

Physiological function test

II. Complex situations to be noted

Body and mind interaction

Mental symptoms caused by physical illness

Abnormal thyroid function → anxiety or depression

Brain tumor → personality change

Vitamin deficiency → mood disorders

Mental illness causes physical symptoms

Depression → loss of appetite, insomnia, fatigue

Anxiety → Palpitations, sweating, gastrointestinal discomfort

Somatic symptom disorder → various physical pains

Mental and physical illness (both coexist)

colitis gravis

High blood pressure

Some skin conditions

III. Key steps in differential diagnosis

Comprehensive medical history collection

The time and order of symptom occurrence

Family history

Life stressors

Necessary medical checkups

Eliminate organic causes

Basic physiological index test

Professional assessment

Psychiatrists and internal medicine doctors need to work together when necessary

Don't self-diagnose

Dynamic view

Symptom progression

therapeutic reaction

IV. Important Reminder

⚠ Do not simply oppose the two:

Mental illness also has a biological basis (neurotransmitters, brain structure, etc.)

Many diseases involve both physical and mental aspects

Modern medicine emphasizes the "biopsychosocial" comprehensive model

⚠ Seek medical attention immediately if you experience any of the following symptoms:

Symptoms persist and interfere with daily life

Cannot determine the nature of the symptoms

Serious emotional or behavioral abnormalities

The physical discomfort does not subside

V. Practical recommendations

First visit: You can first see a general practitioner or a general practitioner for initial screening

Persistent symptoms: refer to the corresponding specialty according to the initial diagnosis

Don't hide your illness: mental illness needs professional treatment just like physical illness

Seek professional help: Don't rely on online self-diagnosis

**Q11. Why do hospitals need multiple tests to diagnose mental diseases?**

The diagnosis of mental illness requires a number of tests, mainly for the following reasons:

1. Exclusion of organic disease

Many physical illnesses can present with psychiatric symptoms:

Neurological diseases: brain tumors, encephalitis, epilepsy, etc. may lead to mental abnormalities

Endocrine disorders: Thyroid dysfunction, diabetes, and other conditions can affect mood and cognition

Metabolic diseases: electrolyte disorders, liver and kidney dysfunction, etc

Drugs/toxins: Side effects of certain drugs or substance abuse

These factors should be ruled out through blood tests, brain imaging (CT/MRI), and EEG.

2. Complexity of psychiatric diagnosis

No specific biological indicators: there is currently no clear test for mental illness such as blood sugar

Symptom overlap: Different mental disorders may have similar symptoms (such as anxiety, insomnia, and inattention)

A comprehensive assessment is required, incorporating medical history, symptoms, psychological evaluations, and behavioral observations.

3. Assessing the severity of the condition

Psychological scale assessment: depression scale, anxiety scale, cognitive function test, etc

Functional assessment: impact on social functioning and ability to perform daily activities

Risk assessment: safety assessment of suicide risk, violent tendency, etc

4. Develop individualized treatment plans

Basic health status: understand the patient's physical condition and determine whether certain treatments can be tolerated

Drug safety: Liver and kidney function, electrocardiogram and other tests to ensure drug safety

Genetic testing: Sometimes genetic testing for drug metabolism is performed to select the most suitable medication

5. Get a full picture of the patient

Family history survey: Mental illness has a genetic predisposition

Psychosocial factors: life events, stressors, interpersonal relationships, etc

Medical history: whether there is a history of similar attacks or treatment

6. Legal and regulatory requirements

Mental illness diagnosis needs to be rigorous to avoid misdiagnosis

Some diagnoses require specific diagnostic criteria and observation time

Common tests include:

✓ Blood tests (complete blood count, biochemical tests, thyroid function tests, etc.)

✓ Brain imaging (CT or MRI)

✓ Electroencephalogram (EEG)

✓ Psychological assessment scale

✓ electrocardiogram

✓ Cognitive function test

Summary: Multiple tests are performed to ensure accurate diagnosis, comprehensive evaluation of the condition, exclusion of other diseases, and safety of treatment. This cautious approach is responsible for the patient and helps to develop the most appropriate treatment plan.

**Q12. Is psychological assessment helpful for the diagnosis of mental illness?**

Psychological assessment is indeed helpful for the diagnosis of mental illness, but it is an auxiliary tool rather than a decisive basis. Let me explain in detail:

The main role of psychological assessment

✅ Helpful aspects

Provide objective data

Quantify symptom severity

Track disease trends

Reduce subjective judgment bias

Screening and initial assessment

Quickly identify potential issues

Identify key areas to focus on

For example: Depression Scale (PHQ-9), Anxiety Scale (GAD-7)

Add clinical information

Assess cognitive functions (memory, attention, etc.)

Assess personality traits

Symptoms that the patient did not report voluntarily were found

Help with differential diagnosis

Distinguish different diseases with similar symptoms

Identify comorbidities

⚠ boundedness

Cannot be diagnosed alone

The evaluation results should be combined with clinical interviews

The actual living situation of the patient must be considered

Cultural background affects assessment results

There may be a deviation

Patients may conceal or exaggerate symptoms

The status of the evaluation day will affect the result

Certain diseases can affect a patient's self-knowledge

Tool scope

Different assessments address different issues

Professional selection and interpretation is required

Location in real-world applications

The standard diagnostic process for mental illness usually includes:

🩺 Detailed clinical interview (core)

Psychological assessment (for reference only)

Necessary medical examination (to exclude physical illness)

👁 behavior observation

Medical and family history

sum up

Psychological assessment serves as a vital auxiliary tool in the diagnostic framework for mental disorders, providing valuable objective data. However, the final diagnosis must be made by a qualified psychiatrist after comprehensive evaluation of multiple factors. If you or someone you know is experiencing mental health challenges, it is advisable to seek assistance from a professional medical institution.

Are you particularly interested in a specific aspect of the psychological assessment? Or do you have questions about it?

**Q13. What should be done if different psychiatrists have different diagnoses?**

How to deal with different psychiatric diagnosis results

It's not uncommon for different doctors to give different diagnoses. Here are some systematic recommendations:

1. Understand the causes of diagnostic differences

The diagnosis may differ because:

Mental illness symptoms may overlap (e.g., anxiety and depression often coexist)

The disease is in different stages of development and manifests in different ways

Doctors have different concerns, theoretical schools, and clinical experiences

Patients vary at different times and provide different information

There are grey areas in the diagnostic criteria for some diseases

II. Specific processing steps

1. Seek third-party advice

Seek an assessment from a more experienced or specialist psychiatrist

Choose a chief physician from a psychiatric department or a specialized psychiatric hospital in a Grade A hospital

Consider seeking a consultation with an expert in the field of the disease

2. Detailed diagnostic comparison

Ask each doctor:

What is the basis for diagnosis?

Why are other possibilities ruled out?

Diagnostic certainty (confirmed/suspected/awaiting observation)

The effect of diagnostic differences on treatment plans

3. Pay attention to treatment feedback

Diagnosis ultimately serves treatment

Try a period of treatment and see the effect

The improvement of symptoms can be used to reverse verify the accuracy of the diagnosis

Regular follow-up and adjustment according to disease progression

4. Improve inspection and evaluation

Conduct standardized scale assessments (e.g., MMPI, SCL-90, etc.)

Electroencephalogram, imaging and other auxiliary examinations should be done when necessary

Provide complete medical and family history

Keep a detailed symptom diary for your doctor to reference

III. Key Notes

✅ What to do:

Keep an open mind: Don't stick to a diagnosis too early

Active communication: fully express all symptoms and concerns to the doctor

Save records: Keep all medical records, prescriptions, and medical histories

Family support: Have family members who know you accompany you to the doctor and provide objective information

Follow-up: Diagnosis of mental illness may require long-term observation

❌ Don't do:

Don't dismiss a doctor just because the diagnosis is different

Don't change doctors too often (unless necessary)

Do not mix medications prescribed by different doctors

Don't hide symptoms for fear of being labeled

Don't rely too much on online self-diagnosis

IV. Special circumstances

If it involves a major decision (such as hospitalization, suspension of study, legal appraisal, etc.):

Request formal multidisciplinary consultation (MDT)

Request for psychiatric forensic evaluation

Consult the hospital medical office or patient rights department

Seek the help of a medical dispute mediation agency when necessary

V. Practical Suggestions

Consider the following when selecting a primary care physician:

The professional qualifications and experience of the doctor

The level of detail and logic of the diagnosis

Whether the doctor is willing to listen and explain

Whether the treatment plan is comprehensive (not just medication)

Whether a good doctor-patient trust relationship has been established

Remember: A psychiatric diagnosis sometimes does require "diagnostic work" and time to observe. Rather than obsessing over the name of the diagnosis, focus on:

Is the symptom improving

Whether the quality of life is improved

Whether social function is restored

If the treatment is effective, there is no need to worry too much even if the diagnosis label is different. The diagnosis and treatment of mental illness is a dynamic process that requires the joint efforts of doctors and patients.

**Q14. Will you be considered to have a mental illness if you visit a psychiatrist?**

No. Seeing a psychiatrist = having a mental illness.

Important Distinction

The scope of psychiatric diagnosis and treatment is very broad

Psychiatry not only treats mental illness, but also includes:

Emotional problems: depression, anxiety, phobia

Sleep disorders: insomnia, drowsiness

Stress-related: adjustment disorder, acute stress response

Psychological distress: interpersonal problems, study and work stress

Neurosis: OCD, social phobia

Others: Attention deficit, eating disorders, etc

"Psychosis" is a specific concept

The medical term "mental illness" usually refers to:

schizophrenia

paranoiac psychosis

Certain stages of bipolar disorder (manic depression)

This is just a small part of the spectrum of mental illness.

Analogical understanding

just as ：

Excluding cardiac issues = heart attack (may present as palpitations or chest tightness)

To the gastroenterology department = gastric cancer (possibly just indigestion)

"Out of psychiatry" = mental illness (may be anxiety, insomnia)

actual situation

Many people seek medical help for mild anxiety, depression, and insomnia

Students seek help due to exam stress and social difficulties

Working professionals seeking career burnout and emotional management counseling

propose

If you have psychological or emotional distress:

Going to the doctor early is a sign of responsibility

A professional assessment is more accurate than worrying about yourself

Early intervention is often more effective

The medical record is protected and you don't need to worry too much

Seeking professional help is a sign of wisdom and courage, not weakness.

**Q15. What are the main methods of treating mental illness?**

The main methods of treating mental illness include the following categories:

1. Drug therapy

Antidepressants: for depression, anxiety, etc

Antipsychotic drugs: used to treat schizophrenia, mania, etc

Emotion stabilizers: treatment for bipolar disorder

Anti-anxiety medication: Relieve anxiety symptoms

Sleep aids: improve sleep disorders

2. Psychotherapy (talk therapy)

Cognitive Behavioral Therapy (CBT): Changing negative thought and behavior patterns

Psychoanalysis: Exploring the unconscious conflict

Interpersonal therapy: Improving interpersonal problems

Dialectical Behavior Therapy (DBT): Particularly effective for borderline personality disorder

Family therapy: Involves the participation of family members

Group therapy: Getting support in a group setting

3. Physiotherapy

Electroconvulsive therapy (ECT): Used for severe depression or other refractory conditions

Transcranial Magnetic Stimulation (TMS): a non-invasive brain stimulation technique

Phototherapy: Treatment of seasonal affective disorder

4. Social rehabilitation and support

vocational rehabilitation

social skill training

Day hospital or community rehabilitation center

Peer Support Group

5. Lifestyle adjustments

Regular exercise

healthy diet

Good sleep habits

Stress Management Tips

Avoid alcohol and drug abuse

Principles of comprehensive treatment

The best treatment for most mental illnesses is a comprehensive approach, usually combining medication and psychotherapy. The treatment plan needs to be based on:

Disease type and severity

Personal information and preferences

Prior treatment response

Side effect tolerance

Important reminder: If you or someone close to you has mental health problems, seek help from a professional psychiatrist or mental health professional who will develop a personalized treatment plan.

**Q16. Which is more effective, psychiatric drug therapy or psychotherapy?**

There is no simple "which is better" answer to the effectiveness of psychiatric medication versus psychotherapy, depending on a number of factors:

📊 Treatment outcomes for different diseases

Drug therapy has more advantages

Severe mental illness: schizophrenia, bipolar disorder

Major depression: especially with suicidal risk and psychotic symptoms

OCD: moderate to severe with medication

Psychotherapy has the advantage

Mild to moderate anxiety/depression

personality disturbance

Post-traumatic stress disorder (PTSD)

Eating disorder

The best effect is when you combine them

Moderate to severe depression: medication combined with cognitive behavioral therapy (CBT)

Anxiety disorder: medication controls symptoms + psychotherapy addresses the root cause

Bipolar disorder: mood stabilizers + psychosocial interventions

Research evidence

Meta-analysis shows:

For depression: monotherapy and psychotherapy are equally effective, and the relapse rate is reduced by 40-50% with combined treatment

For anxiety disorders, cognitive behavioral therapy (CBT) demonstrates superior long-term efficacy compared to monotherapy with medication.

For schizophrenia: must be based on medication, psychosocial intervention can improve function

Their respective advantages and disadvantages

| dimension | medication | mind-cure |
| --- | --- | --- |
| Speed of effect | Faster (2-6 weeks) | Slow (months) |
| Keep effect | Relapse is easy after stopping the drug | Longer-lasting effects |
| side effect | There may be side effects | Almost no side effects |
| usability | extensive | It requires a certain level of cognitive ability and motivation |
| cost | Relatively low | Relatively high |

Individualized selection considerations

Disease severity: Acute/Severe priority drugs

Prior treatment response: which approach has been effective

Patient preference: treatment adherence is important

Accessibility: Quality psychotherapy resources are limited

Side effect tolerance: Some patients are not suitable for drugs

Clinical consensus

Mainstream view of modern psychiatry:

It's not "either/or," it's integrative therapy

Medicine addresses the biological basis (neurotransmitter imbalance)

Psychotherapy addresses psychosocial factors (cognitive patterns, coping strategies)

Best practice: ✅ Use medication to quickly control symptoms during the acute phase

✅ Psychological therapy is added during the stabilization phase to consolidate the therapeutic effect

✅ Maintenance therapy during recovery period to prevent recurrence

In summary, just as diabetes requires medication for blood sugar control and lifestyle adjustments, mental illness treatment also needs a comprehensive bio-psycho-social approach. The specific plan should be tailored by a professional doctor based on individual circumstances.

**Q17.Is exercise effective against mental illness?**

Exercise does have significant therapeutic effects on mental illness, and a large number of scientific studies support this conclusion. Here is a detailed analysis:

Validity evidence

Depression

Mild to moderate depression: Exercise was as effective as antidepressants

Aerobic exercise for 30-45 minutes, 3-5 times a week, can significantly improve symptoms

Long-term adherence can reduce the recurrence rate

Anxiety Disorders

Reduce overall anxiety

Helps with generalized anxiety disorder and panic disorder

Anxiety relief can be felt immediately after exercise (lasting for several hours)

For other mental illnesses

Schizophrenia: improving negative symptoms, cognitive function and quality of life

Bipolar disorder: stabilize mood and reduce frequency of episodes

ADHD: Enhancing Attention and Executive Functions

Post-traumatic stress disorder (PTSD): Alleviating symptom severity

mechanism of action

Neurobiological level

Promote the release of neurotransmitters such as endorphins, serotonin, and dopamine

Stimulate the production of brain-derived neurotrophic factor (BDNF) to promote neural regeneration

Lower stress hormone (cortisol) levels

Psychosocial dimensions

Boost your self-efficacy and self-esteem

Provide opportunities for social interaction

Shift focus and break negative thought cycles

Effective exercise type

Aerobic exercise: running, swimming, cycling (best evidence)

Strength training: Just as effective

Yoga, Tai Chi: Particularly effective for anxiety and stress

Team sports: increase social support

Important reminder

⚠ Exercise is an adjunct and cannot completely replace professional treatment

For severe mental illness, medication and psychotherapy are required

Consult your doctor before starting an exercise program

Progress gradually and avoid overexertion

Recommended dose

The World Health Organization recommends:

At least 150 minutes of moderate-intensity exercise per week

Or 75 minutes of high-intensity exercise

The key is consistency and regularity, not intensity

Bottom line: Exercise is an effective tool for treating mental illness, but it should be part of a comprehensive treatment plan, not the only one.

**Q18. What kinds of drugs are commonly used to treat mental illness?**

The commonly used drugs for the treatment of mental diseases are mainly divided into the following categories:

1. Antipsychotic drugs

Used to treat schizophrenia, bipolar disorder manic episodes, etc

Typical antipsychotics: chlorpromazine, haloperidol, etc

Atypical antipsychotics: risperidone, olanzapine, quetiapine, aripiprazole, etc.

2. Antidepressants

Used to treat depression, anxiety disorders, etc

Selective serotonin reuptake inhibitors (SSRIs): Fluoxetine, Sertraline, Paroxetine, and others

Serotonin and norepinephrine reuptake inhibitors (SNRIs): venlafaxine, duloxetine, and others

Tricyclic antidepressants (TCAs): amitriptyline, imipramine, etc.

Other types: Mirtazapine, bupropion, etc

3. Anti-anxiety drugs

Used to treat anxiety, panic disorder, etc

Benzodiazepines: lorazepam, alprazolam, diazepam, etc

Non-benzodiazepines: butyrrolone, etc

4. Mood stabilizers

For the treatment of bipolar disorder

lithium salts

Antiepileptic drugs: sodium valproate, carbamazepine, lamotrigine, etc

5. Central Stimulants

For the treatment of Attention Deficit Hyperactivity Disorder (ADHD)

Methylenephthalate, amphetamine and so on

6. Sleep aids

For the treatment of insomnia

Benzodiazepine receptor agonists: zolpidem, dexzopiclone, etc.

Melatonin receptor agonists, etc

Important Notice

All psychotropic drugs should be used under the guidance of a professional psychiatrist

Do not increase or decrease the dose or discontinue the drug by yourself

Regular follow-up visits are required to monitor efficacy and side effects

Each drug has specific indications and contraindications

If you or someone you know has mental health problems, it is recommended to seek medical advice from a professional doctor.

**Q19.How long does it usually take for psychiatric drugs to take effect?**

The onset time of psychiatric drugs varies greatly depending on the type of drug and individual differences. The following is the general onset time of the main types of drugs:

antidepressant

Initial improvement: 1-2 weeks (e.g., sleep, appetite)

Significant improvement in mood: 2-4 weeks

Complete cure: 4-8 weeks, sometimes 12 weeks

Common medications: SSRI (e.g., sertraline, fluoxetine) and SNRI (e.g., bupropion, nortriptyline)

antianxietic

Benzodiazepines (e.g., lorazepam, alprazolam)

Time to take effect: 30 minutes to 1 hour (quick effect)

Usually used for short-term treatment

Other anti-anxiety medications (e.g., ditilidronate)

Effective in 2-4 weeks

antipsychotic drug

Acute symptoms: several days to 1-2 weeks

Complete cure: 4-6 weeks, sometimes longer

Used to treat schizophrenia, bipolar disorder, etc

Emotion stabilizers (e.g., lithium, sodium valproate)

Effective in 1-3 weeks

Stable effect: may take several months to adjust the dose

ADHD medicinal

Stimulants: 30 minutes to 2 hours (effective the same day)

Non-doping: 2-4 weeks

Important reminder

Do not stop taking the medication on your own: Even if you don't see immediate results, continue taking it as prescribed.

Individual differences: People have different reaction times

Side effects: May occur before the effect appears and need to be discussed with your doctor

Regular follow-up: The doctor will adjust the dosage or change the drug according to the effect

If there is no improvement after 4-6 weeks of medication, consult your doctor to adjust the treatment plan. Psychiatric treatment usually requires medication combined with psychotherapy for better results.

**Q20. Will taking psychiatric drugs damage brain function?**

The effect of psychiatric drugs on brain function

This is a very important question. Simply put: the rational use of psychiatric drugs under the guidance of a doctor usually does not damage brain function, but can help restore the normal operation of the brain. Here is a detailed explanation:

🧠 How the drug works

Psychiatric drugs work primarily by regulating neurotransmitters (such as serotonin, dopamine, norepinephrine) to help restore the brain's chemical balance, rather than "damaging" it.

✅ positive role

Protect brain structure

Untreated mental illnesses (such as depression and schizophrenia) can cause hippocampal atrophy and neuronal damage

Timely medication can prevent or reverse these pathological changes

Recovery of cognitive function

Improve attention, memory, and decision-making

Reduce the long-term damage to brain function caused by disease

⚠ Side effects to watch out for

Some medications may cause:

Short-term side effects: drowsiness, dizziness, temporary loss of concentration (usually adapts)

Considerations for long-term use: regular monitoring and dosage adjustment are required

Drug withdrawal reaction: sudden discontinuation may cause discomfort, and the dosage should be gradually reduced

🔑 Key principles

| Key points for safe medication | explain |
| --- | --- |
| follow the doctor's advice | Do not increase or decrease the dose or discontinue the drug |
| Regular checkups | Monitor efficacy and side effects |
| ind. th | The doctor will adjust the plan according to the situation |
| weigh the advantages and disadvantages | The benefits of treatment usually far outweigh the risks |

💡 Scientific consensus

Mainstream medical research shows that untreated mental illness causes far more damage to the brain than the risk of standard medication

Many patients experience significant improvement in brain function after treatment

Long-term studies have found no permanent brain damage from conventional doses of psychiatric drugs

propose

If you are considering medication or have concerns about your current medication:

Talk to your psychiatrist about your concerns

Understand the effects and side effects of specific drugs

Don't refuse necessary treatment out of fear

Evaluate treatment regularly

Remember: Mental illness can be treated, and proper medication is an important way to help the brain recover. If you have any questions, consult a professional doctor, not to stop taking the medicine.

**Q21. How to deal with the side effects of psychiatric drugs, such as weight gain and drowsiness?**

Strategies for coping with the side effects of psychiatric drugs

The side effects of psychiatric drugs do bother a lot of people, and here are some ways to deal with weight gain and drowsiness:

First principle

Never stop taking the medication or adjust the dosage without consulting your doctor. Always discuss with your doctor to develop a plan.

Medical response

1. Communicate with your doctor to adjust the plan

Adjust the drug dose (while ensuring efficacy)

Replace the same drug with a less side effect

Adjust the timing of medication (e.g., take before bedtime if drowsiness occurs)

Consider combination therapy to reduce the dose of a single drug

2. Periodic monitoring

Track weight, diet, and sleep

Regular follow-up to monitor side effects

🍎 Tips to combat weight gain

Diet

Control total calories and avoid high sugar and high fat foods

Increase your intake of vegetables, whole grains, and high-quality protein

Eat small, frequent meals and avoid overeating

Drink plenty of water and reduce sugary drinks

Exercise recommendations

At least 150 minutes of moderate-intensity exercise per week

Start with gentle exercises such as walking and swimming

Exercise can improve both mood and weight

Behavioral changes

Keep a food diary and notice your eating patterns

Avoid emotional eating

Get enough sleep (sleep deprivation can also lead to weight gain)

😴 Tips for staying alert

Adjust medication time

Consult your doctor to take the medication before bedtime

Avoid taking medication when you need to be highly focused

Lifestyle optimization

Keep a regular sleep schedule and wake up at a fixed time

Morning exposure to natural light helps you wake up

Moderate exercise increases energy

Avoid daytime naps (no more than 30 minutes)

Ways to refresh

Take a short break and do light activities

Keep the environment ventilated and bright

Drink coffee or tea in moderation (be careful not to conflict with certain medications)

⏱ Time dimension considerations

Tolerance period: Some side effects will naturally subside after 2-4 weeks of medication

Weighing the pros and cons: If the drug is effective, mild side effects may be acceptable

Long-term planning: discuss with your doctor to gradually reduce the medication when the condition is stable

⚠ When to seek immediate medical attention

A short-term increase in body weight (e.g., more than 5 kg in January)

Drowsiness seriously affects daily life or work

Other serious side effects

Emotional symptoms worsened

💬 mental accommodation

Adapting to treatment takes time and adjustment

Share experiences with support groups or fellow patients

Remember: Controlling the disease itself is the most important goal

Side effects are temporary, health is long-term

Keep in mind: Everyone reacts differently to medications, so personalized treatment plans are essential. Maintaining open communication with your doctor and honestly reporting side effects are key to finding the best treatment. Many side effects can be managed, so don't give up on treatment!

**Q22. Can I get addicted to psychiatric drugs?**

This question needs to be divided into different situations, and the addiction of different types of psychotropic drugs is very different:

🟢 Drugs with low addiction risk or no addiction

Antidepressants (e.g., SSRIs)

Almost no addiction, such as sertraline, fluoxetine, paroxetine, etc

No euphoria, no abuse tendency

However, sudden discontinuation of the drug may cause withdrawal symptoms (dizziness, nausea, etc.), which is not addiction, but physiological adaptation

antipsychotic drug

It is not addictive, such as risperidone, olanzapine, quetiapine

Used to treat schizophrenia, bipolar disorder, etc

Mental stability

It is not addictive, such as lithium salt, valproic acid sodium, etc

🟡 Drugs with some risk of addiction

Benzodiazepine anti-anxiety drugs

Such as alprazolam, lorazepam, diazepam, etc

Long-term use of high doses may lead to dependence

It should be used under the guidance of a doctor and should not be taken for a long time

The drug should be reduced gradually

Some sleep aids

Long-term use of drugs such as zolpidem may lead to dependence

🔴 Drugs with higher addiction risk

central stimulant

Methamphetamine (Ritalin) used to treat ADHD

It is safe to use it properly under the supervision of a doctor

💡 Key distinction: dependence vs. addiction

Physiological dependence: The body adapts to the drug, and sudden discontinuation can cause discomfort (e.g., antidepressants)

Addiction: psychological craving, compulsive drug use, and resorting to any means to obtain drugs

Safe medication use recommendations

Take medicine as prescribed: take the prescribed dose and time

Regular follow-up visits: Let your doctor evaluate the effectiveness and need of your medication

Do not stop taking the drug by yourself: when you need to stop taking the drug, you should gradually reduce the dosage

Do not share medication with others

Tell your doctor: If you have a history of substance abuse, tell your doctor in advance

Summary: Most psychiatric medications are safe and non-addictive when used under a doctor's guidance. However, certain anti-anxiety medications require careful use. The key is standardized treatment and regular follow-up.

If you have questions about a specific medication, consult your psychiatrist or pharmacist.

**Q23. What is a seizure-free electroconvulsive therapy?**

Modified Electroconvulsive Therapy (MECT), also known as modified electroconvulsive therapy, is a crucial psychiatric treatment.

fundamental

By briefly stimulating the brain with electric currents to induce widespread cortical discharges, MECT aims to treat mental disorders. Unlike traditional electroconvulsive therapy (ECT), MECT uses anesthetic drugs and muscle relaxants before treatment.

main features

What does "no convulsions" mean

General anesthesia: The patient receives a short-acting anesthetic before treatment

Muscle relaxation: use muscle relaxants to block neuromuscular conduction

Avoid convulsions: Although the brain still has epileptic-like discharges, the body will not have severe convulsions

Reduce injury: Avoid complications such as fractures and dislocations that may occur with traditional ECT

therapeutic process

Preoperative preparation: Fasting and water restriction, physical status assessment

Anesthesia induction: intravenous injection of anesthetic and muscle relaxant

Electrode stimulation: placing electrodes on specific areas of the head to deliver brief electrical pulses (typically lasting several seconds)

Monitoring: whole process ECG, blood pressure, blood oxygen monitoring

Awakening: The patient gradually wakes up after 5-10 minutes

principal indication

Major depression (especially in those who are unresponsive to medication)

Patients at high risk of suicide

Acute phase of schizophrenia

maniacalis insultus

catatonia

superiority

✓ High safety and few complications

✓ Fast onset, significant efficacy for some cases

✓ No pain for the patient

✓ Avoids physical damage to traditional ECT

Possible side effects

Short-term memory impairment (usually reversible)

Headache and nausea after treatment

muscular soreness

Temporary impairment of cognitive function

Important note: MECT is a specialized medical procedure that must be performed by qualified medical institutions and trained healthcare professionals. Treatment decisions should be made by psychiatrists after comprehensive evaluation of the patient's specific condition.

**Q24.What are the main diseases treated by transcranial magnetic stimulation?**

Main indications for transcranial magnetic stimulation (TMS) therapy

Approved Indications

1. Depression (the most important indication)

Resistant depression: moderate to severe depression that does not respond well to medication

Depression is approved by several countries for FDA

Typically stimulates the left dorsolateral prefrontal cortex (DLPFC)

2. Obsessive-Compulsive Disorder (OCD)

In 2018, the FDA approved it for treating obsessive-compulsive disorder.

For patients who do not respond well to drug therapy

3. Migraines

For migraine prevention and acute treatment

Specific types of TMS devices have been approved

Diseases with mature clinical application

4. Anxiety disorders

generalized anxiety

Social anxiety

panic disorder

5. Schizophrenia

Mainly used to treat positive symptoms such as hallucinations

Adjuvant therapy

6. Rehabilitation after stroke

Motor Function Recovery

laloplegia

Cognitive function improvement

7. Chronic pain

Neuropathic pain

Fibromyalgia

Complex regional pain syndrome

Disease in the research and exploration phase

8. Neurodegenerative diseases

Parkinson's disease (motor and emotional symptoms)

Alzheimer's disease (cognitive function)

9. Addiction disorders

Substance dependence (tobacco, alcohol, drugs)

Reduce cravings

10. Other mental disorders

Bipolar disorder (depressive phase)

Post-traumatic stress disorder (PTSD)

Eating disorder

11. Neurological diseases

epilepsia

tinnitus

MS

matters need attention

⚠ Contraindications include:

Metal implants in the body (e.g., pacemakers, cochlear implants)

Metallic foreign body in the brain

Careful evaluation is required in patients with a history of epilepsy

Important note:

TMS treatment must be administered by trained physicians in specialized medical institutions.

The treatment plan should be individualized

Multiple treatments are usually needed to see results

Specific indications may vary depending on national and regional approvals

It is recommended to consult a neurologist or psychiatrist for a comprehensive evaluation before considering TMS treatment.

**Q25. Can I stop taking psychiatric drugs by myself after symptoms improve?**

No! You should never stop taking psychiatric drugs on your own when your symptoms improve.

Why can't I stop taking the drug myself?

1. High risk of disease recurrence

Mental disorders (such as depression, bipolar disorder, schizophrenia, etc.) tend to recur

Symptom improvement does not equate to cure; it may simply be the result of medication control.

Suddenly stopping the drug can cause a relapse, even worse than before

2. Withdrawal reactions

Drug withdrawal may occur:

Dizziness, nausea, increased anxiety

Insomnia, irritability

Physical discomfort (sweating, shivering, etc.)

Symptoms like the flu

3. All the work before treatment is wasted

Each recurrence may increase the difficulty of treatment

It may take longer and a larger dose to get it under control again

The right way

✅ Adjust under a doctor's guidance

Regular follow-up: Let your doctor evaluate your condition

Gradual reduction: slow down the dosage as directed by your doctor

Observation: closely monitor symptom changes during drug reduction

✅ The timing of stopping the medication is determined by a professional doctor

The doctor will consider the following:

The duration of symptom relief (typically requiring several months to several years)

Medical history (number of episodes, severity)

Social function recovery

Drug type and dosage

✅ The importance of maintenance therapy

First attack: usually requires maintenance treatment for more than 6-12 months

Multiple recurrences: may take longer, even long-term medication

Chronic course: some conditions require lifelong maintenance therapy

Important Notice

Emergency: If you have stopped taking the medicine and feel unwell, contact your doctor immediately. Do not hide it.

Communication is key: If you have any concerns about medication (side effects, dependence, etc.), you should actively communicate with your doctor to find a solution

Remember: psychiatric medication adjustment is a process that requires professional judgment and close monitoring, and the risk of stopping medication on your own is far greater than the risk of continued treatment.

**Q26. How long should psychiatric drugs be taken after the condition is stable?**

Duration of maintenance treatment with psychiatric drugs

Psychiatric medications are usually taken for a long time after the condition is stabilized, which is called "maintenance treatment." The duration varies depending on the type of illness and individual circumstances:

General recommendations for different diseases

1. Depression

First attack: Continue taking the drug for at least 6-12 months after the symptoms are relieved

Second attack: maintenance therapy is recommended for 2-3 years

Three or more episodes: may require long-term or even lifelong medication

2. Anxiety

Maintenance therapy is generally required for 1-2 years

Some patients may take longer

3. Bipolar disorder

Long-term maintenance therapy is usually required (for several years or even a lifetime)

Prevent relapse of manic or depressive episodes

4. Schizophrenia

First attack: at least 2-5 years of maintenance therapy

Multiple recurrences: long-term or lifelong medication is recommended

⚠ Why can't you stop taking the medicine without permission?

High recurrence risk: premature discontinuation of medication significantly increases the recurrence rate (up to 60-80%)

Worsening of the condition: Recurrent symptoms may be worse than before

Increased treatment difficulty: Re-treatment may be more difficult and less effective

Withdrawal reactions: sudden discontinuation of the drug may cause physical discomfort

🔍 Individualized considerations

Disease type and severity

Occurrence count and frequency

Drug side effects

Patient compliance

Social support system

Merge with other diseases

How to stop taking the medicine

If discontinuation is considered, the following must be done:

Discuss with your doctor when to discontinue the drug

The gradual reduction usually takes several weeks to months

Monitor changes in the condition

Regular checkups

Seek immediate medical attention if symptoms of recurrence occur

Important reminder: The above is for general reference only. Do not stop taking the medication or adjust the dosage on your own. Any medication adjustment must be made under the guidance of a psychiatrist, who will develop a personalized treatment plan based on your specific condition.

Do you have a specific disease type or medication questions? I can provide more targeted information.

**Q27. Can mental illness be completely cured?**

Whether a mental illness can be completely cured is a complex question, and the answer depends on a number of factors, such as the type of illness, the severity of the illness, and the timeliness of treatment.

A condition that can be cured or long-term remitted

Some mental illnesses can be cured:

Depression: After standardized treatment for the first episode, about 60-70% of patients can fully recover

Anxiety disorder: Many patients can achieve symptom disappearance through psychotherapy and drug therapy

Post-traumatic stress disorder (PTSD) can be significantly improved or even cured with targeted treatment.

Adaptation disorder: usually good prognosis, most can fully recover

Long-term management required

Some diseases are more like a "chronic disease" management model:

Bipolar disorder: requires long-term medication and psychological support to stabilize and control

Schizophrenia: Some patients can return to normal life through treatment, but may need long-term medication

OCD: can be controlled to not affect life, but prone to relapse

Key factors affecting rehabilitation

✓ Early detection and treatment (the earlier the intervention, the better the effect)

✓ Standardized treatment plan (combination of medication and psychotherapy)

✓ Continue treatment (do not stop medication immediately when symptoms improve)

✓ Social support system (family understanding and support)

✓ Healthy lifestyle (regular sleep, exercise, stress reduction)

Important reminder

"Remission" does not necessarily mean that it will never recur, but:

Symptoms are under control

Be able to work and live normally

Have a good quality of life

If you or someone you know is experiencing mental health problems, it is recommended to seek help from a professional psychiatrist or psychotherapist. Modern medicine has effective treatments for most mental illnesses.

**Q28. Will the mental illness relapse after rehabilitation?**

There is a real possibility of relapse after mental illness rehabilitation, but it is not inevitable. The risk of relapse varies depending on the type of illness, the treatment and personal factors.

Recurrence Overview

Recurrent rate of common mental disorders:

Depression: About 50% of people who have a first episode will relapse, and the risk increases to 70-80% after multiple episodes

Bipolar disorder: high relapse rate, about 70-90%

Schizophrenia: the relapse rate is about 70-80% within one year after discontinuation

Anxiety disorder: recurrence rate of about 30-50%

Key factors affecting the outcome

Factors that increase the risk of recurrence:

✗ Stopping medication without permission or taking it irregularly

Life stressors (unemployment, divorce, loss of loved ones, etc.)

✗ Lack of sleep or irregular sleep

Alcoholism or drug abuse

✗ Lack of social support

History of multiple prior episodes

Measures to reduce the risk of recurrence:

✓ Adhere to maintenance therapy: Take medication as prescribed (typically for several months to several years)

✓ Regular follow-up: timely adjustment of treatment plan

✓ Psychotherapy: such as cognitive behavioral therapy (CBT)

✓ Identify warning signs: sleep changes, mood swings, abnormal thinking, etc

✓ Healthy lifestyle: regular sleep, moderate exercise, balanced diet

✓ Stress management: Learn coping skills

✓ Family support: Good social support system

Important reminder

Do not stop medication without guidance: Even if you feel fully recovered, gradually taper off under a doctor's supervision.

Recovery is possible: Many people live a high quality of life through continued management

Early intervention: If signs of recurrence are found, timely medical treatment can effectively control

Conclusion: Although there is a risk of recurrence of mental illness, the recurrence rate can be significantly reduced through long-term scientific management and active life adjustment, and many patients can achieve stable recovery.

In specific cases, it is recommended to consult a professional psychiatrist to develop a personalized rehabilitation plan.

**Q29. How to prevent the recurrence of mental illness?**

Comprehensive strategies to prevent relapse of mental illness

Preventing relapse of mental illness requires a multifaceted, long-term effort. Here are the key preventive measures:

1. Stick to standard treatment

Take medicine on time: Strictly follow the doctor's instructions, do not stop or reduce the dosage by yourself

Regular follow-up: Follow up regularly as required by your doctor and adjust your treatment plan as needed

Monitor side effects: inform the doctor of any adverse drug reactions in time and do not stop taking the drug without authorization

2. Identification of early warning signals

Watch for these warning signs:

Sleep pattern changes (insomnia or drowsiness)

Emotional fluctuations are noticeable

Social withdrawal or abnormal behavior

Changes in appetite and energy levels

Difficulty concentrating

Contact your doctor immediately if you notice

3. Establishing a healthy lifestyle

Maintain a regular sleep schedule: go to bed at the same time and get 7-8 hours of sleep

Moderate exercise: 30 minutes of aerobic exercise 3-5 times a week

Eat a balanced diet: Avoid alcohol, caffeine and drugs

Quit smoking and limit alcohol: These substances may trigger or worsen symptoms

4. Stress Management

Learn relaxation techniques (meditation, deep breathing, yoga)

Manage work and life

Avoid overexertion

Develop healthy coping strategies

5. Psychological support

Sustained psychotherapy: Cognitive Behavioral Therapy (CBT) and similar approaches can effectively prevent relapse.

Join a support group: Connect with others who have had similar experiences

Family education: Help family members understand the disease and provide appropriate support

6. Avoid triggers

Stay away from high pressure

Avoid major life changes (plan ahead if possible)

Avoid drug and alcohol abuse

Identify personal triggers

7. Build support networks

Stay in touch with family and friends

Let those you trust know about your condition and needs

Create an emergency contact list

Participate in community or interest group activities

8. Develop contingency plans

Written record: contact information of attending physician, emergency contact

List early warning signs

Know when to ask for help

Let your family know about this plan

9. Self-monitoring

Record your mood diary

Use the emotion tracking app

Periodic Self-Assessment

Stay aware of your state

Important reminder

Recurrence does not equal failure: Mental illness is recurrent, and timely intervention is crucial

Personalized plan: Each person's situation is different, and work with the doctor to develop a suitable prevention plan

Long-term management: Mental illness requires long-term management and should not be taken lightly

Seek professional help: Consult a doctor if you have any doubts or symptoms

Remember: Preventing recurrence is a continuous process that requires the joint efforts of patients, families and medical teams. Maintain a positive attitude and believe that through scientific management, the risk of recurrence can be effectively reduced and the quality of life can be improved.

If you have any specific questions or need advice on specific mental illnesses, consult a professional psychiatrist.

**Q30. Are mental illnesses inherited?**

Mental illness does have a genetic predisposition, but it's not simply "inevitable". Here's the breakdown:

The role of genetic factors

1. Polygenic inheritance pattern

Most mental illnesses are not determined by a single gene, but by a combination of genes and environmental factors

It's hereditary' susceptibility 'or' increased risk' rather than the disease itself

2. Heritability of common mental disorders

| Disease type | Heritability (contribution of genetic factors) | Risk Statement |
| --- | --- | --- |
| schizophrenia | 60-80% | The prevalence rate in the general population is 1%. If one parent is affected, the risk for children is about 10-15%. |
| bipolar affective disorder | 60-85% | If one parent is ill, the risk to the child is about 10-25% |
| depression | 40-50% | If parents are ill, the risk for children increases 2-3 times |
| anxiety neurosis | 30-40% | Genetic factors are weak, environmental factors are more important |
| Autism spectrum disorder | 80-90% | The genetic factor is strong, but the mechanism is complex |

Important Notice

✓ Not necessarily inherited

Even if the parents are ill, the children are more likely not to be

People without a family history of mental illness can also develop mental illness

✓ Environmental factors are also key

Life stress, traumatic experiences, social support, lifestyle, etc. can affect the onset

A good environment for growth can reduce risk

✓ Can prevent and intervene

Early identification of high-risk groups

Healthy lifestyle (regular sleep, exercise, social interaction)

Timely psychological support and treatment

propose

If you have concerns about family history:

Don't worry too much-increased risk does not mean certain disease

Maintain a healthy lifestyle-this is the best preventive measure

Focus on mental health-conduct regular self-assessment and seek professional help when necessary

Consider genetic counseling-If you're planning a family and have a significant family history, consult a specialist

Mental illness can be treated and managed, and modern medicine has many effective interventions. What matters is scientific understanding, not excessive worry or discrimination because of genetic factors.

**Q31. How can people with mental illness better reintegrate into society?**

The return of people with mental illness to society is a gradual process that requires the combined efforts of individuals, families, the medical system and society. Here are some key strategies:

I. MEDICAL AND REHABILITATION

1. Stick to standard treatment

Take the medicine as prescribed and do not stop or adjust the dosage without authorization

Regular follow-up and timely adjustment of the treatment plan

Participate in psychotherapy (such as cognitive behavioral therapy, group therapy, etc.)

Learn to recognize early signs of disease recurrence

2. Rehabilitation training plan

Participate in activities at the community rehabilitation center

Provide vocational skills training

Participate in social skills training

Rebuilding daily living skills (sleep schedule management, self-care ability, etc.)

II. Efforts at the individual level

1. Establishing a healthy lifestyle

Keep a regular schedule and get enough sleep

Moderate exercise (walking, yoga, tai chi, etc.)

Eat a balanced diet and avoid alcohol and tobacco

Develop hobbies (painting, music, gardening, etc.)

2. Set realistic goals

Start small and transition gradually

Don't rush it. Accept that recovery takes time

Track progress and build confidence

Learn to motivate yourself and think positively

3. Stress Management

Learn relaxation techniques (meditation, breathing exercises)

Identify stressors and develop coping strategies

Avoid taking on excessive responsibility

Seek support instead of bearing it alone

III. Social and interpersonal relationships

1. Rebuild your social network

Start with people you know (family, old friends)

Join a peer support group

Join community activities or volunteer groups

Take interest classes or social clubs

2. Communication skills

Learn how to disclose your illness appropriately

Don't make it public to everyone, but be honest with those close to you

Educate people around you about the reality of mental illness

Set boundaries and protect your mental space

IV. EMPLOYMENT AND EDUCATION

1. Job preparation

Assess your work abilities and interests

Choose the right amount of work and environment

Use employment assistance programs or sheltered employment

Consider flexible employment, part-time or home work

Understand the rights and interests of laws such as the Law on the Protection of Persons with Disabilities

2. Workplace Adaptation

Communicate with your employer or HR department if needed

Request reasonable accommodation

Create workplace support system

Learn to balance work and self-care

V. Family support

1. Family education

Family members learn about mental illness

Understand the patient's symptoms and behavior

Join a family support group

Avoid overprotection or high expectations

2. Create a supportive environment

Provide emotional support and encouragement

Assist in monitoring treatment compliance

Create a low-stress home atmosphere

Respect the independence and privacy of patients

VI. SUPPORT AT THE SOCIETAL LEVEL

1. Use community resources

Community Mental Health Service Center

Rehabilitation institutions and day care centers

Psychological support hotline

Patient Support Group

Government subsidies and welfare policies

2. Reducing social stigma

Know your rights and fight discrimination

Join anti-stigma campaigns

Change public perception through education

Share a recovery story (when you're ready)

7. Strategies for dealing with setbacks

1. Accept the possibility of relapse

Recurrence does not equal failure

Seek medical help promptly

Summarize experience and adjust rehabilitation plan

Keep hope and patience

2. Establish a crisis response plan

Display warning signals

Prepare an emergency contact list

Identify crisis intervention resources

Work with doctors to develop emergency plans

VIII. LONG-TERM MAINTENANCE STRATEGY

Self-monitoring: Regularly assess symptoms and functional status

Lifelong learning: Continuously improving coping skills

Maintain treatment relationships: Keep in touch with your healthcare team

Give back: Help others within your means and strengthen your sense of value

Important reminder

✅ Recovery is a personalized journey, and everyone's pace is different

✅ Seeking professional help is a sign of strength, not weakness

✅ Small steps are more important than perfection

✅ You're not alone. There are plenty of resources and people ready to help.

Reintegration into society is a journey, not a destination. What matters is staying hopeful, believing in recovery, and taking small positive steps each day. Many people with mental illness have successfully reintegrated into society and lived fulfilling lives—so can you!

If you or someone you care about is going through this process, it is recommended to first consult a professional psychiatrist or psychotherapist to develop a personalized recovery plan.

**Q32. How to deal with the discrimination and comments of others against people with mental illness?**

Deal with the discrimination and comments of others against people with mental illness

This is an important and intelligent problem. Here are some strategies from different perspectives:

1. If you are the patient

1. Protect your boundaries

Selective disclosure: You are not obligated to explain your situation to everyone

Leave promptly: When encountering malicious comments, allow yourself to leave or end the conversation

Find safe space: Build supportive social circles and avoid long-term exposure to environments filled with prejudice

2. Responding to specific situations

Responding to curiosity with ignorance: "Thanks for your concern, but this is my personal health issue."

Respond to a hostile comment: "This comment is hurtful. I don't accept this way of talking"

Facing the misunderstanding of "goodwill": "I understand your starting point, but mental illness may not be the same as what you think"

3. Build psychological defenses

Remind yourself that someone else's prejudice reflects their cognitive limitations, not your values

Record positive moments and provide supportive feedback

Discuss your feelings about discrimination with your therapist regularly

2. If you're family or friends

1. Direct protection

Stop the behavior immediately: "That's not appropriate. Please stop."

Private communication: "What you just said may unintentionally hurt them"

Set boundaries for the patient when necessary: "That's all for this topic"

2. Education and advocacy

Example of a gentle but firm response:

"Mental illness is a medical problem, like diabetes, and needs to be treated, not blamed"

"You may not realize it, but that kind of thing can really hurt"

"If you're interested, I can share some scientific information"

3. Emotional support

Ask the patient privately: "Are you okay? Do you need to talk?"

Value: "Your illness doesn't define who you are"

Companionship without overprotection

3. If you're a bystander

Speak up to break the silence

"I don't agree with that" (simple statement)

"It's not appropriate to talk about someone else's health"

Move the conversation or leave the room (not participating is an attitude)

Create an inclusive environment

Use accurate language in everyday conversations (avoid terms like "crazy" or "mad")

Share the right information and break the myth

Support related public welfare activities

IV. LONG-TERM STRATEGY

individual level

Keep learning: Stay informed about mental health and empower yourself with facts

Join a community: Online/offline support groups provide understanding and strength

Professional support: Psychological counseling to help deal with the trauma of discrimination

Legal awareness: seek legal aid for serious discrimination (e.g. employment discrimination)

social aspect

Join the anti-stigma movement

Share real stories in a safe way

Advocacy for mental health policies

5. Important Reminder

✅ can be done ：

Prioritize mental health over arguing with everyone

Distinguish between the ignorance that deserves education and the harm of malice

Create a "supporter alliance" to respond together

❌ Avoid doing:

Self-doubt due to others' prejudices

Overexert yourself to "educate" everyone

Isolate yourself or give up seeking help

Keep these core principles in mind

Your health and safety always come first

Prejudice is about someone else's problem, not your fault

Change takes time, but every voice counts

You don't have to face it alone. Professional and community support are important

If you or someone you care about is going through this, is there a specific situation that needs more specific advice? I can help you analyze the situation.

**Q33. Can you fall in love with someone with mental illness?**

This is a question worth exploring in depth. Simply put: yes, but it requires thorough understanding and preparation.

Reasons to consider

1. Mental illness does not mean that you cannot love

People with mental illness also have the right and ability to love and be loved

Many mental illnesses (such as depression and anxiety) can be well controlled with treatment

Mental illness is only part of a person, not all of them

2. The core of relationships is people themselves

What matters is the character, values, and attitude of the person towards relationships

Many people with mental illness can build healthy relationships after treatment

A reality that needs to be rationally faced

1. Understanding the disease

Disease type, severity, and treatment status

The other person's awareness of their illness (whether they recognize their condition)

Treatment compliance and stability

2. Potential challenges in relationships

Emotional fluctuations may be more frequent or intense

More understanding, patience, and support may be needed

May face social prejudice and external pressure

There may be times when the other person may not respond with the same emotion

3. What you expect from yourself

Draw a line: You're not a therapist and can't replace professional help

Protect yourself: Don't enter a relationship out of pity, and take care of your own mental health

Real assessment: Are you truly willing and able to face possible difficulties

Consideration

✓ Is the other person actively treating and managing their condition?

✓ Is there a genuine emotional connection between you, not based on "saving" or sympathy?

✓ Do you know about this disease and are willing to learn how to support?

✓ Are you mentally healthy and stable enough?

✓ Can you communicate honestly, including about illness?

most important of all

Love requires equality, respect and mutual support. Mental illness can be an additional challenge, but it is entirely possible to build a healthy relationship if both parties are willing to work hard and be honest with each other.

The key is not whether the other person has a mental illness, but:

Does the relationship make both people feel respected and supported

Are you both willing to work for the health of your relationship

Does the relationship foster growth for both parties rather than drain them

If you are facing such a choice, it is recommended to talk to your trusted friends and family, and consult a mental health professional if necessary.

**Q34. Can people with mental illness get married?**

As for whether people with mental illness can get married, I will answer you from the following perspectives:

Legal perspective

China legal provisions:

The Civil Code (effective January 1, 2021) has removed the prohibition in the original Marriage Law that "a person suffering from a medical condition deemed unsuitable for marriage" would be barred from marriage.

Mental illness is no longer legally prohibited from marrying

But the requirement of marriage must be that both parties have full capacity for civil conduct

Key judgment criteria:

If a person with a mental illness can recognize his or her own behavior and has full civil capacity, he or she can get married

If a person is unable to recognize his own behavior due to mental illness, he is a person without civil capacity or a person with limited civil capacity, and he is unable to register marriage independently

Medical perspective

There are many kinds of mental illness, and the condition varies greatly:

Patients with mild or recovered conditions, such as mild depression or anxiety, can marry after treatment and their condition stabilizes.

Patients with severe or acute conditions: such as those in the acute phase of schizophrenia, may temporarily lack the capacity to make marital decisions.

actual operation

At marriage registration:

The registration authority mainly examines whether the consciousness of the parties is clear and whether the will is true

Mental illness screening is not mandatory

If you cannot clearly express your true intention, you may postpone registration

propose

Condition assessment: Consult a professional doctor to evaluate your current condition and decision-making ability in marriage

Honesty: It is recommended to tell your spouse about your illness, which is related to the quality of life after marriage

Legal protection: If the other party conceals a serious illness, it may constitute a revocable marriage

Conclusion: The marriage rights of patients with mental diseases are protected by law, and the key lies in whether they have full civil capacity and real intention to get married.

**Q35. How to get along with family members with mental illness?**

Dealing with family members who have mental illness does require special understanding and skill. Here are some practical suggestions:

1. Learning about illness

Active learning: Learn about the symptoms, treatments, and progression of your family's illness

Distinguish disease from person: Understand that certain behaviors are manifestations of disease, not directed at you personally

Recognize limitations: understand that mental illness can not be solved by "thinking more"

2. Improve communication

Stay calm and gentle: Avoid an agitated, accusatory, or critical tone

Focus on listening: Allow ample space for expression without rushing to judge or offer solutions.

Use simple and clear language: Avoid complex or vague expressions

Focus on feelings rather than right or wrong: Accept their emotional experience

Avoid saying: "You're overthinking it," "Everyone else is fine," or "You're just too lazy."

3. Build a support system

Professional treatment: Encourage and assist family members to seek medical treatment and take medicine on time

Join a family support group: Talk to people who have had similar experiences

Seek psychological counseling: seek professional psychological support for yourself when necessary

Stay in touch with your doctor: Learn about your condition and how to manage it

4. Set reasonable boundaries

Protect yourself: Maintain your mental health while providing support

Draw clear boundaries: Set clear boundaries for harmful behavior

Learn to say no: Not all requests have to be met

Avoid overprotection: encourage independence within safe limits

5. Daily interaction skills

Keep a regular schedule: Helps establish a steady rhythm

Focus on positive changes: Recognize progress and effort in a timely manner

Avoid excessive focus on symptoms: create a relatively normal family atmosphere

Provide specific help, such as accompanying patients to the hospital and assisting with daily affairs

Identify crisis signals: Know when to respond

6. Take care of yourself

Accept your emotions: Feeling tired, frustrated or angry is normal

Protect your personal time: Do things you enjoy regularly

Maintaining social life: Don't isolate yourself completely

Physical and mental health first: make sure you get enough rest and nutrition

7. Keep a long-term perspective

Accepting the long-term nature of the illness: Recovery can be a long process

Celebrate small progress: Don't expect instant results

Stay hopeful: Believe in the power of treatment and support

Adjust the strategy regularly: respond flexibly to changes in the condition

Emergency handling

Seek professional help immediately if you experience any of the following:

Tendency to self-harm or harm others

Experience severe hallucinations or delusions

Unable to perform daily activities

Refusal to eat or basic care

Remember: You're not a professional doctor, and you don't need to or should shoulder all the responsibility alone. Dealing with a family member who has a mental illness is a marathon, not a sprint. Make sure to take care of yourself so you can better support your family.

**Q36. How to determine whether a family member has a relapse of mental illness?**

How to identify warning signs of a relapse in a family member with mental illness

It's important to recognize early signs of a relapse in mental illness to help with timely intervention. Here are some key observations:

🔍 Behavior change signal

1. Daily routine changes start to be disordered

Sudden change in sleep pattern (insomnia, drowsiness, or reversal of day and night)

Poor hygiene habits (not showering, not changing clothes)

Abnormal eating habits (sudden increase or decrease in appetite)

2. Social withdrawal

Avoid social activities and family gatherings

Unwilling to communicate with others and spend long periods alone

Lose interest in things you used to like

3. Mood swings

Emotional ups and downs or persistent depression

Increased irritability, anxiety, and fear

Crying or laughing without obvious reason

⚠ Severe symptom alert

Cognitive and perceptual abnormalities

Confusion: contradictory statements and unclear logic

Delusional symptoms: Unreasonable thoughts such as being suspected of being watched or persecuted

Hallucinations: hearing sounds, seeing things that aren't there

Difficulty concentrating and a significant decline in memory

dystropy

Make impulsive, unusual decisions

Tendency to self-harm or harm others

Drug or alcohol abuse

Stop taking prescription drugs

Practical observation method

Compare to baseline

Record the patient's stable phase as a reference

Pay attention to the degree of deviation from the usual state

Monitor duration of changes (be alert if it lasts more than 2 weeks)

Multi-dimensional observation

| dimension | Observation Points |
| --- | --- |
| language | Speech speed, content, and tone changes |
| expression | Are the eyes and facial expressions dull or overexcited |
| activity | Energy level, speed of action, goal orientation |
| interpersonal | Quality and frequency of family interactions |

🩺 Response suggestions

jump into action

Stay calm and supportive and avoid blaming

Ask about your recent feelings and concerns in a gentle way

Contact your doctor and describe any changes you've noticed

Ensure medication adherence by checking that medication is taken on time

emergency

Seek immediate medical attention if you experience any of the following symptoms:

⚡ Have clear suicidal or violent thoughts

⚡ Completely detached from reality

⚡ Skip eating for over 24 hours

⚡ Extreme agitation or complete immobility

preventive action

Regular follow-up: follow up regularly as prescribed

Stress management: Helps identify and reduce stressors

Establish a support system: family members should have a unified understanding and coping strategies

Educate yourself: understand the patient's specific diagnosis and typical pattern of recurrence

Crisis plan: Prepare emergency contact information and procedures in advance

⭐ Important reminder

Each person's recurrence pattern may be different. It's important to know your family's specific symptoms.

Early detection and early intervention can significantly reduce the severity of recurrence

Don't hesitate to seek professional help. Psychiatrists, psychotherapists, and community mental health services are all important resources.

Caregivers also need to pay attention to their own mental health

If you are currently facing this situation, it is recommended that you contact a medical professional as soon as possible, who can provide a targeted assessment and treatment plan.

**Q37.What should you do if family members have hallucinations?**

When a family member has hallucinations, it is a serious situation. Here are some specific suggestions for dealing with it:

🚨 Immediate action

1. Stay calm and safe

Don't panic or show fear

Assess the risk of self-harm or harm to others

In case of emergency, call 120 immediately

2. Seek medical care promptly

Book a psychiatrist or psychologist as soon as possible

Go to:

Psychiatry Department of General Hospital

Mental Health Center/Mental Specialty Hospital

Mental Health Clinic

Don't delay. Hallucinations can be a symptom of many diseases (schizophrenia, bipolar disorder, depression, neurological diseases, etc.)

How to communicate with family

The correct way:

Listen to their feelings and don't deny them right away

In a calm tone: "I understand this is real for you, but I didn't hear"

Express care: "I'm worried about you. Shall we go to the doctor together?"

Avoid arguing about the truth of the hallucination

Avoid doing:

❌ Laugh or blame

❌ Say "You're crazy" or "This can't be"

❌ Reinforce the hallucination (pretend you hear it too)

❌ Ignore or downplay symptoms

Daily Care Tips

Record symptoms

When and how often hallucinations occur

Content features (command-style, comment-style, etc.)

inducing factor

Symptoms (emotional and behavioral changes)

Create a safe environment

Reduce stressors

Keep a regular schedule

Avoid alcohol and drugs

Keep potentially dangerous items out of reach

Follow your doctor's advice

Take your medicine on time and do not stop taking it without permission

Regular checkups

Monitor for drug side effects

Offer emotional support

Companionship and understanding

Encourage participation in daily activities

Helps maintain social connections

⚠ Emergency medical care required

Hear a command that harms yourself or others

Showing violent tendencies or suicidal thoughts

Complete loss of the ability to judge reality

Unable to perform daily tasks

Symptoms suddenly worsen

Long-term recommendations

Learn about related diseases

Join a family support group

Focus on your mental health (caregivers also need support)

Create a crisis response plan

Remember: Auditory hallucinations are a medical condition, not a personality flaw. Timely professional treatment usually helps improve symptoms. Your support and understanding are crucial for your family's recovery.

If you need more specific advice or have any other questions, please let me know.

**Q38.What should we do if people with mental illness commit suicide?**

Measures to deal with suicidal behavior in patients with mental illness

When a suicidal behavior occurs in a person with a mental illness, immediate action is required. The following is a systematic response guide:

🚨 Emergency

1. Engaging in suicidal behaviour

Call the emergency number immediately (China: 120/110)

Keep patients from being left alone and remove dangerous items (medications, knives, ropes, etc.)

Stay calm and talk to them in a calm tone

Don't argue or try to convince them to "open up"

2. Having a clear plan for suicide

Contact a psychiatrist immediately or seek psychiatric care at a hospital's psychiatric department or psychological crisis intervention center.

24-hour monitoring and no one allowed to move alone

Contact the patient's attending physician to adjust the treatment plan

📋 Assess suicide risk level

High-risk signal:

A clear plan and time for suicide

Suicide tools prepared

Farewell behavior (dividing money, writing a will)

History of suicide attempt

Recently experienced a major life stressor

A profound sense of despair and worthlessness

💡 Specific steps to take

Short-term processing (0-72 hours)

Hospitalization: high-risk patients should be hospitalized immediately

Medication adjustment: Psychiatrists evaluate and adjust medication

Continuous monitoring: Family members take turns to care for the patient and remove all dangerous items

Set up a support system: notify family and friends to form a support network

Intermediate management (1 week to 1 month)

Regular medical treatment: take medicine as prescribed and have regular follow-up visits

Psychotherapy: combined with cognitive behavioral therapy (CBT) and dialectical behavioral therapy (DBT)

Identify triggers: Record what triggers mood swings and suicidal thoughts

Crisis plan: Develop a written crisis response card

Long-term prevention (ongoing)

Continue treatment: do not stop or reduce medication without advice

Daily routine: Keep a regular schedule, exercise, and socialize

Emotion monitoring: Use an emotion diary to track your mental state

Family education: Learn to recognize signs of relapse

🗣 communication skill

What to do:

✅ Listen carefully without interrupting

✅ Show care: "I'm worried about you. I want to help you."

✅ Ask directly: "Do you have any thoughts of harming yourself?"

✅ Acknowledge feelings: "I understand you're feeling really bad right now"

Do not:

"Don't think too much" and "You have to be strong" are empty words of comfort

❌ Disregard or ridicule their feelings

❌ Leave patient alone

❌ Commit to confidentiality (life safety first)

📞 Emergency help resources

Emergency number: 120 (medical) and 110 (police)

Psychological assistance hotline:

Nationwide: 12320 (health hotline)

Beijing: 010-82951332 (psychological crisis intervention hotline)

Shanghai: 021-62785533

Guangzhou: 020-81899120

Mental health institutions: local mental health centres, psychiatric departments in general hospitals

Important Notice

Life safety is always the first priority, and patients can be sent to the hospital against their will when necessary

Family members also need support, and caregivers should seek help when stressed

Suicide is not a moral issue, but a symptom of illness that requires medical intervention

Most suicides are preventable and timely intervention is crucial

If you or someone you know is going through this, seek professional help immediately. Remember: even in the darkest moments, help and hope are there.

**Q39. What should we do if a patient with mental illness shows violent tendencies?**

Measures to deal with violent tendencies in patients with mental illness

I. EMERGENCY MANAGEMENT

When danger is imminent:

ensure safety

Keep your distance to avoid angering the patient

Move others (especially children and the elderly) to a safe area

Remove any potential hazards (such as knives, sharp objects, etc.)

Seek help

Call emergency services (120) or police (110)

Make it clear that "violent behavior occurs in people with mental illness"

Some areas have mental health hotlines. You can call them first.

Don't fight

Avoid physical conflict

Do not try to force the patient to be subdued

2. Identify early warning signals

Watch for these signs:

Increased verbal threats or aggressive language

Excitement, restlessness, and intense mood swings

Worsening delusions or hallucinations (e.g., believing others are trying to harm you)

Refuse to take medication or treatment

Sleep disturbances worsen

Drug abuse (alcohol, drugs)

History of violence

III. PROFESSIONAL MEDICAL INTERVentions

Seek medical attention immediately:

Psychiatric Emergency

Take the patient to the psychiatric department of general hospital or psychiatric hospital

Compulsory medical treatment may be applied for when necessary (subject to legal procedures)

Adjust treatment plan

The doctor may need to adjust the dosage or type of medication

Assess the need for hospitalization

Follow up regularly and monitor the condition

Multidisciplinary collaboration

Psychiatrists, psychotherapists and social workers are involved

IV. Strategies for families and caregivers

daily management ：

Continue treatment: urge to take medicine on time and in the right amount

Reduce triggers: Avoid arguments, blame, and excessive criticism

Establish regular sleep patterns: A stable living environment helps stabilize emotions

Learn crisis intervention skills: Attend family training courses

communication skill ：

Stay calm and speak softly

Use simple, clear language

Give patients space and time

Avoid looking directly into the eyes (this may be perceived as a provocation)

Listen to the patient and show understanding

V. LONG-TERM MANAGEMENT

Create support system

Contact the community mental health center

Join a family support group

Learn about local mental health resources

Create a crisis response plan

Record the contact information of the attending physician

Get your medical records and medication list ready

Define family members' roles

legal protection

Understand the relevant provisions of the Mental Health Act

Apply for guardianship if necessary

If the public safety is seriously harmed, compulsory medical treatment may be applied

VI. Important Reminder

⚠ don't ：

Neglect or deny the existence of violent tendencies

Facing danger alone

Discontinuing medication or changing treatment plan without authorization

Moral judgment of the patient

✅ To achieve:

Treat violent tendencies as a disease symptom, not a moral problem

Seek professional help

Protect yourself and others

Adhere to long-term treatment and management

Key point: The violent tendency of mental illness patients can be controlled through standardized treatment and scientific management. Early identification, timely intervention and professional treatment are the key. If the situation is urgent or beyond the ability of family members, it is necessary to seek professional medical and public security department help immediately.

**Q40.How to persuade patients with mental illness to refuse to see a doctor?**

How to persuade a person with a mental illness to seek medical care

This is a process that requires patience and skill. Here are some practical tips:

1. Understand why you refuse medical treatment

Start by understanding the patient's possible concerns:

Stigma: Fear of being labeled, discriminated against

Loss of awareness of illness: not thinking you have a problem

Fear: Fear of the treatment process, side effects of medication

Past negative experiences: had an unpleasant experience of seeking medical treatment

Lack of trust: distrust of doctors or the healthcare system

II. Effective communication strategies

1. Choose the right time and place

Talk to the patient when he or she is relatively calm and emotionally stable

Choose a private, safe, and comfortable environment

Avoid persuasion during conflict or symptom flare-ups

2. Use empathetic communication

✅ "I noticed you haven't been sleeping well lately. I'm sure it's been tough for you"

❌ "You're sick and need to see a doctor"

Listen to their feelings and don't rush to deny or preach

3. Focus on specific symptoms rather than diagnosis

Talk about specific issues that can be improved: "Let's see how we can help you sleep better."

Avoid saying "you're crazy" or "you're not normal"

4. Highlighting the benefits of seeking medical care

From the patient's perspective: improve quality of life, restore work ability, reduce pain

Provide success stories (if appropriate)

5. Lowering barriers to healthcare

It is recommended to start with psychological counseling rather than going directly to psychiatry

Can accompany the patient to the doctor and provide support

"Only to get advice" "Listen to professional advice"

6. Proceed step by step

Step 1: Acknowledge some of the troubles

↓

Step 2: Willing to try to improve

↓

Step 3: Get a professional assessment

↓

Step 4: Follow the treatment plan

III. Practical Tips

Third-party perspective

"I had a friend who was in a similar situation and went to the doctor and he was much better."

Reduce defensive psychology

Experiential Suggestions

"We can go and see it once, and if we don't like it, we won't go."

Reduce commitment

Trust people

Patients should ask trusted friends, family members, and religious leaders to help persuade them

Sometimes it helps to have a doctor's advice

Written information

Provide popular science articles, videos, etc., so that patients can understand by themselves

Avoid preachy tone

IV. Matters needing attention

⚠ Things to avoid:

Coercion, threats, humiliation

Discussing illness in front of others

Make unrealistic promises like "it will be cured soon"

Neglect the patient's feelings and concerns

✅ Things to do:

Be patient. You may need to try several times

Respect the autonomy of patients (as long as it is safe)

Show care and support consistently

Take care of your emotions

V. EMERGENCY MANAGEMENT

Seek professional help immediately if you experience any of the following:

Suicidal or self-harming tendencies

Thoughts or actions that hurt others

Totally unable to take care of their basic needs

Severe hallucinations or delusions affect safety

This may require:

Call the psychological crisis hotline (e.g., 12320,010-82951332)

Contact your community mental health agency

Access to compulsory medical treatment when necessary (subject to legal procedures)

VI. LONG-TERM SUPPORT

Treatment is a process, and be prepared to provide ongoing support

Learn about related diseases to better understand patients

Join a family support group for experience and emotional support

Take care of your mental health and seek help if necessary

Remember: Change takes time, and your ongoing care and support is a powerful healing force in itself. If one attempt doesn't work, don't give up—stay connected and supportive, and wait for the next right moment.

**Q41. Should family members with mental illness be allowed to manage their money independently?**

This is a question that requires a comprehensive judgment based on specific circumstances, and cannot be generalized. The following are some factors to consider:

Key factors to evaluate

1. Illness status and cognitive ability

Is the condition stable? Is the treatment effective?

Do you have basic judgment and self-control?

How do you perceive and manage money?

2. Disease types and symptoms

Different mental illnesses affect different people (such as depression, bipolar disorder, schizophrenia, etc.)

Are there any risky behaviors such as impulse spending, delusions, and susceptibility to fraud?

The degree to which symptoms affect decision-making ability

Suggested handling

Progressive management (recommended)

✅ Small-amount autonomy: Manage daily pocket money and maintain dignity

✅ Co-account: Important account settings for co-management

✅ Limit Settings: Set spending limits and reminders

✅ Regular communication: Discuss financial plans instead of completely stripping away rights

Protective measures when risk is high

Family members help manage large amounts of money

Consider legal guardianship or a property trust

Regularly review and adjust management methods

Important principles

Respect autonomy: preserve their sense of participation and decision-making as much as possible under the premise of safety

Dynamic evaluation: Adjust management methods as the condition changes

Professional advice: consult a psychiatrist and legal professionals

Transparent communication: Avoid backroom dealings and build trust

The key is to balance protection and dignity, both to prevent risks and not to deprive patients of their basic rights. It is recommended to work with doctors and patients themselves (when they are able to judge) to develop appropriate plans.

**Q42. What is the fastest way to find a person with a mental illness who has gone missing?**

A fast way to find a person with a mental illness after they go missing

Time is of the essence when a person with a mental illness goes missing. Here are the fastest and most effective ways to find them, in order of priority:

🚨 First time (0-30 minutes)

1. Call the police immediately-the most important step

Call 110 to report the patient, who has a mental illness and is a missing person from a special group.

Provide details: name, age, physical description, clothing, time and place of disappearance

Emphasize the patient's possible risk behavior tendencies

The police will immediately activate the search mechanism and call in surveillance resources

2. Search on-site and ask nearby

Search immediately near the location (within 500 meters)

Ask nearby shops, security guards, and passers-by if they have seen it

Check the patient's usual or likely places of visit (park, station, former residence, etc.)

3. Mobilize relatives and friends to search separately

Quickly notify friends and family via WeChat or phone

Search by region

Keep in touch and report anytime

📱 Technology (running in parallel)

4. Use online platforms to spread

Post a missing person notice on WeChat Moments or Weibo (with photos, description, and contact details)

Join local WeChat groups and community groups

Post on platforms like Toutiao (with geolocation)

Use public welfare search platforms such as Toutiao Search for People

5. Retrieve surveillance footage

Ask the police to help locate the lost person and the surrounding surveillance

Contact the property management or mall to retrieve the surveillance footage

Track your movements

🏥 Professional resources

6. Contact rescue agencies

Call the local rescue station (civil affairs department)

Contact the emergency department of major hospitals (patients may seek medical care due to illness)

Report to the local mental health center

7. Media Help

Contact your local TV station or radio station

Contact the newspaper to publish a missing person notice

Many local media have public service search columns

⚠ Important Notice

Prime time: The first 24 hours after a loss are critical

Don't wait: No need to wait 24 hours to report, mental illness patients are a special case

Take photos: Prepare several recent clear photos of the patient

Stay calm: Panic can cloud judgment

Record info: Note all leads and people you've contacted

Preventive measures

Wear a lost-behind-hand bracelet (with contact information) for the patient

Stitch contact cards onto clothing

Consider using GPS

Carry patient photos and information cards with you

Remember: Reporting to the police is the fastest and most effective way. With professional equipment and experience, the police can greatly improve the chances of finding the person. At the same time, take multiple measures, use social forces and technical means, and race against time!

I hope the patient will return safely soon. If you need more specific advice, please let me know the details.

**Q43. As a family member of a mental illness patient, how to relieve your own stress?**

As family members of people with mental illness, they are under great psychological and life stress. Here are some effective ways to relieve stress:

Zen: Take care of yourself

Maintain basic life rhythm

Get enough sleep and eat regularly

Engage in moderate exercise (walking, yoga, swimming, etc.)

Keep your hobbies and don't give up your personal life completely

Set boundaries

Realizing you can't care for someone 24 hours a day is not selfish

Learn to say no and avoid overcommitting

Accept the fact that I've done my best

👥 Seeking support

Professional support

Consider seeking psychological counseling to deal with your emotions

Join a family support group and talk to people who have had similar experiences

Learn about the patient's condition from the doctor and reduce anxiety about the unknown

social support

Talk to a trusted friend (protect patient privacy)

Don't bear it alone. Ask other family members to share the burden

Find community care resources

📚 Learn and understand

Increase knowledge about diseases

Understand the characteristics and development of the patient's disease

Learn practical techniques for dealing with symptoms

Understand what is a disease symptom and what can change

Adjust expectations

Recovery can be a long process

Let go of the obsession with "making the patient as good as new"

Focus on small steps, not perfect results

💭 emotion management

Allow yourself to feel negative

Acknowledge that anger, sadness, and frustration are normal

Don't blame yourself for these emotions

Find safe ways to express your emotions (write a diary, exercise, etc.)

Mindfulness and relaxation techniques

Practice deep breathing, meditation

Try progressive muscle relaxation

Learn to live in the present without worrying too much about the future

Legal Notice | Real-life arrangements

Manage time wisely

Set your own "break time"

Seek daytime care or short-term substitute care if possible

Keep up your work and social activities and maintain a normal life

Financial Planning

Understand health insurance and government subsidies

Make a long-term financial plan to reduce financial stress

⚠ Watch for caregiver burnout

If you experience any of the following symptoms, seek help immediately:

Persistent fatigue and insomnia

Lack of empathy for the patient and resentment

Neglect your health

Social isolation, loss of interest in everything

Experience depressive or anxious symptoms

💪 Remember these principles

Your health matters too-Only by taking care of yourself can you better support patients

Seeking help is not weakness-it is a sign of wisdom and responsibility

Perfection is not the goal-doing "good enough" is already impressive

It's not your fault-the occurrence of illness is not anyone's fault

Caring for people with mental illness is a marathon, not a sprint. Remember to recharge yourself regularly so you can go further. If you feel overwhelmed, professional counseling can be a great help.

The fact that you're thinking about this shows you're a responsible family member. I hope these suggestions will help you.

**Q44. If children show signs of 'reduced speech, irritability, and aversion to learning', could this indicate psychological or mental health issues?**

Yes, children who are "uncommunicative, irritable and averse to learning" may indeed have mental and psychological problems.

Possible mental and psychological problems

1. Depressive disorder

Childhood depression is often characterized by social withdrawal, silence, and loss of interest.

Irritability (or quick to anger) is a hallmark of childhood depression, distinct from the sadness seen in adults.

Decline in academic performance and aversion to learning

2. Anxiety disorders

Separation anxiety, social anxiety, school phobia

May manifest as avoidance behaviors (unwillingness to talk, unwillingness to go to school)

Emotional stress leads to irritability

3. Adjustment disorder

After life changes (moving, transferring schools, family changes)

Emotional and behavioral problems

4. Attention Deficit Hyperactivity Disorder (ADHD)

Learning difficulties lead to aversion to learning

Emotional regulation problems lead to irritability

May have social difficulties

5. Autism spectrum disorder

Social communication barriers (not talking much)

Difficulty regulating emotions

Poor adaptation to the school environment

6. Trauma-related disorders

Experienced bullying, abuse, and severe post-traumatic stress

Emotional withdrawal, increased alertness

Warning signs to watch for

Symptoms last more than 2 weeks

Affects daily functions (learning, social, family life)

Self-harm, suicidal thoughts or behaviors

Sleep and appetite changes

Distinct personality change

Recommendations

jump into action ：

Professional assessment: Consult a child psychiatrist or psychotherapist

Detailed observation: Record the time, frequency, and triggers of symptoms

Exclusion of physical illness: Some physical illnesses can also cause similar manifestations

At the family level:

Create a safe, supportive home environment

Listen patiently and avoid criticism and blame

Learn about the school (whether there is bullying or academic stress)

Stay in touch with your teacher

School cooperation:

Communicate with the homeroom teacher and the psychology teacher

Apply for academic adjustment if necessary

Important reminder

⚠ Don't ignore these signs: Children often struggle to express their inner pain, and changes in behavior and emotions may be signs of help.

⚠ Avoid self-diagnosis: Only professionals can make accurate diagnoses.

⚠ Early intervention is crucial: When children's psychological issues receive timely and professional help, the prognosis is generally favorable.

It is recommended to seek professional child mental and psychological assessment as soon as possible to clarify the diagnosis and develop appropriate intervention plans.

**Q45. Can pregnant women or those who are trying to conceive take psychiatric drugs?**

Guidelines for the use of psychiatric drugs during pregnancy or preconception

General principles

It can be taken, but it requires professional evaluation and management. The key is to weigh the pros and cons:

Untreated mental illness can pose serious risks to both mother and child

Some drugs do have potential risks

Individualized assessment is needed, not a one-size-fits-all approach

Risk assessment points

Risks of not treating

Severe depression/anxiety affects pregnancy care

Suicide risk

Malnutrition, substance abuse

Premature birth, low birth weight

Postpartum depression worsens

Drug risk

Teratogenic risk (mainly in early pregnancy)

Neonatal adaptation syndrome

Long-term neurodevelopmental effects

Common psychiatric drug classifications

Relatively high security

antidepressant

✅ SSRI class: Sertraline, Fluoxetine (relatively safe, but requires monitoring)

⚠ Paxlovid: Early Pregnancy May Increase Risk of Heart Defects

antianxietic

⚠ Benzodiazepines (e.g., lorazepam): Short-term use may be acceptable, but long-term use requires caution

antipsychotic drug

✅ Atypical antipsychotics (e.g., olanzapine): Some studies indicate relative safety

Please pay special attention

mood stabilizer

⛔ Sodium valproate: High teratogenic risk, avoid during preconception

⛔ Carbamazepine: Risk of neural tube defects

Lithium salt: Risk of heart defects, but can be used under strict monitoring

Specific recommendations

Pregnancy Preparation

Consult a psychiatrist and an OB/GYN 3-6 months early

Assess whether the medication regimen needs to be adjusted

Optimize the dose to the minimum effective dose

Take folic acid supplements (5mg/day), especially if taking antiepileptic drugs

pregnancy

Do not stop taking the medicine by yourself (sudden discontinuation may cause the condition to recur)

Early pregnancy (0-12 weeks): a critical period for teratogenic sensitivity, requiring close monitoring

Regular prenatal checkups, including ultrasound screening

Monitor drug concentrations (if needed)

Postpartum/breastfeeding

Most psychiatric drugs enter breast milk

The balance between medication and breastfeeding needs to be assessed

Some medications (such as sertraline) are less concentrated in breast milk

Important reminder

Personalized decision-making: Everyone's situation is different, there is no standard answer

Multidisciplinary collaboration: joint management by psychiatry, obstetrics and gynecology, and neonatology

Be fully informed: Understand all risks and benefits

Ongoing monitoring: close follow-up throughout pregnancy and postpartum

🩺 Action suggestions

Ask now: If you're taking medication and planning to get pregnant

Medical history: detailed medication history and symptom records

🤝 Partner involvement: Family support is crucial

Avoid stopping medication on your own: it may be more risky than taking it

Important: The above information is for reference only and cannot replace professional medical advice. Please discuss your specific situation with your doctor and develop a personalized treatment plan.

Do you have any specific medications or symptoms you'd like to know about? I can provide more detailed information.

**Q46. Can psychiatric drugs be taken with other drugs?**

The combination of psychiatric drugs with other drugs

Psychiatric medications can be taken with other drugs, but special attention should be paid to drug interactions. Here are the key points:

I. BASIC PRINCIPLES

✅ Can be used together, but requires professional evaluation

Must be done under the guidance of a doctor

The safety of specific drug combinations needs to be evaluated

Dose or timing may need to be adjusted

II. Common interaction risks

🔴 High-risk portfolio

Antidepressants combined with certain painkillers (e.g., tramadol) may cause serotonin syndrome.

Lithium salts combined with diuretics or NSAIDs (e.g., ibuprofen) increase the risk of lithium toxicity.

Antipsychotic drugs + certain antibiotics (e.g., erythromycin) → arrhythmia

Benzodiazepines + opioids → respiratory depression

⚠ Watchlist

Antidepressants combined with anticoagulants (e.g., warfarin) increase the risk of bleeding

Antipsychotic drugs + antihypertensive drugs → low blood pressure

Emotion stabilizers + antiepileptic drugs → changes in drug concentration

III. Special Notes

📋 Inform your doctor about the following

All medications you are taking (including prescription and over-the-counter medications)

Health supplements and Chinese herbal medicines (such as St. John's wort, which may reduce the effectiveness of certain medications)

Vitamin supplements

Alcohol and other substance use

Clock 🕑 Time management for medication

Some medications need to be taken at intervals

Some need to be taken with food, some need to be taken on an empty stomach

Adjust the schedule of taking medicine as prescribed by your doctor

4. Common security combinations

The following combinations are generally safe (but still require a doctor's confirmation):

Antidepressant + regular blood pressure medication (monitoring required)

Anti-anxiety medication + anti-allergy medication (note: sedation may be added)

Emotional stabilizer + regular stomach medicine (some require interval)

5. Important Reminder

Medicine. Every time you visit:

Bring a complete medication list

Tell all your doctors that you are taking psychiatric medication

Consult a pharmacist before taking a new drug

🚨 Seek immediate medical attention if you experience any of the following:

Severe dizziness and palpitations

Abnormal bleeding or bruising

Severe nausea, vomiting

Confusion, muscle stiffness

Summary: The combination of psychiatric drugs with other drugs is common and feasible, but it must be done under the supervision of medical professionals. Do not stop or add drugs at will, and regular follow-up and blood drug concentration monitoring are important.

**Q47. Can I drink alcohol, coffee or strong tea while taking psychiatric drugs?**

Dietary precautions during psychiatric medication

Alcohol (strongly not recommended)

Why not drink?

Enhance sedation: Alcohol significantly enhances the sedative effects of antidepressants, anti-anxiety drugs, sleeping pills, etc., which may lead to:

Excessive drowsiness and sluggishness

Judgment and coordination skills are severely impaired

Respiratory depression (life-threatening in severe cases)

Reduced efficacy: Alcohol may interfere with drug metabolism and reduce the therapeutic effect

Side effects: dizziness, nausea, blood pressure fluctuations, etc

Liver burden: Alcohol and most psychiatric drugs require liver metabolism, and their concurrent use increases the burden on the liver.

Special risk: Drinking alcohol while taking certain medications (such as monoamine oxidase inhibitors, MAOIs) may cause dangerous blood pressure fluctuations.

Suggestion: Avoid alcohol completely during medication

Coffee (moderate)

Possible effects:

Drug interactions with anti-anxiety or sleeping pills: caffeine acts as a stimulant and may counteract the sedative effects of these medications.

Worsens anxiety: High caffeine intake may worsen anxiety, heart palpitations, and insomnia

Drug interactions with certain antidepressants: may increase neural excitability

Affects sleep: Disrupts sleep quality, which is critical for mental health

propose ：

Drink in moderation (1-2 cups per day)

Avoid drinking in the afternoon or evening

If palpitations, increased anxiety, or other symptoms occur, reduce or discontinue

Minimize intake of sedatives

🍵 Strong tea (note)

Possible effects:

Contains caffeine: Strong tea also contains caffeine and has similar effects to coffee but is usually milder

Effect on drug absorption: Tannic acid in tea may bind with some drugs and affect absorption

Excitatory effect: may counteract the effects of sedatives

propose ：

You can drink light tea and avoid strong tea

Take your medicine at least 1-2 hours apart from drinking tea

Avoid alcohol at night

General recommendations

Always consult your doctor: Different psychiatric medications may interact differently with these beverages. Please consult your attending physician or pharmacist for specific medication guidance.

Preferred choice: Boiled water or warm water is the best choice for taking medicine

Observation: If you experience any discomfort after drinking, stop immediately and inform your doctor.

Keep track of your habits: Tell your doctor about your eating and drinking habits so you can adjust your treatment plan

Emergency: If you drink alcohol or feel seriously unwell, seek medical attention immediately

Important note: The above are general recommendations. The specific situation varies according to the type of drug, dosage and individual constitution. Please follow the doctor's advice and do not adjust the medication or diet habits by yourself.

**Q48. What are the dietary restrictions for patients with mental illness during medication?**

Dietary restrictions for patients with mental illness during medication

People with mental illness should pay attention to their diet during medication, because some foods may affect the efficacy or produce adverse reactions. Here are the main precautions:

I. Dietary restrictions according to the type of drug

1. Monoamine oxidase inhibitors (MAOIs)

These drugs are the most strictly contraindicated and should avoid foods containing tyramine:

❌ Forbidden foods: aged cheese, pickled foods, fermented soy products (doubanjiang, stinky tofu), cured meat, smoked meat, red wine, beer, soy sauce, kimchi, etc.

⚠ Risk: May cause hypertensive crisis

2. Lithium-based medications

❌ Limit sodium: Avoid sudden changes in salt intake (too much or too little can affect lithium concentration)

💧 Stay hydrated: Drink 2000-3000ml daily to prevent dehydration

☕ Limit caffeine: Coffee and strong tea may increase lithium excretion

3. Antipsychotic drugs

🚫 Alcohol: May enhance sedation and increase risk of falls

☕ Avoid strong tea and coffee: They may interfere with drug absorption

🥤 Avoid grapefruit juice: It affects drug metabolism

4. Antidepressants (SSRIs, etc.)

🚫 Alcohol consumption is prohibited: It increases the burden on the liver and aggravates side effects

☕ Limit caffeine: It may worsen anxiety and insomnia

🥤 Avoid grapefruit juice: It may interfere with the metabolism of certain medications

II. General dietary precautions

✅ Suggest doing

Eat regularly and in appropriate amounts

Get a balanced diet and eat more fresh fruits and vegetables

Drink enough water to promote metabolism

Eat light, less oil and less salt

Be sure to take vitamin B complex supplements

❌ Should be avoided

Alcohol: Interacts with almost all psychiatric drugs

Grapefruit/yuzu: affects the liver metabolism of many drugs

Strong tea and coffee: Affect the efficacy and sleep

Spicy foods: May aggravate some side effects

Foods high in fat: Affect drug absorption

III. Special Notes

⏰ price index number of service expenditure

Some medications should be taken on an empty stomach

Some medications should be taken after meals

Follow your doctor's instructions

⚖ weight management

Some antipsychotics increase appetite

Control your calorie intake and exercise moderately

Monitor weight regularly

💊 drug interaction

Tell your doctor all medications you are taking (including Chinese medicine and health products)

Do not discontinue or adjust the dose without consulting a physician

IV. Important Reminder

⚠ Seek immediate medical attention if you experience any of the following symptoms:

Suffering from severe headache, palpitations, nausea and vomiting

Severe drowsiness or altered consciousness

Involuntary muscle movements

Fever, sweating, muscle stiffness

kindly reminder

Individual differences are large: different drugs and different patients may have different dietary restrictions

Follow your doctor's advice is the most important thing: follow your doctor's advice

Regular follow-up: monitor blood drug concentration and physical indicators

Detailed records: Record diet and body reactions to help doctors adjust the plan

Be sure to consult your doctor or pharmacist for personalized dietary guidance for your medication.

**LLM Model:Claude 4.5 Haiku**

**Q1. What is mental illness?**

I will use an Artifact of the Markdown document type to comprehensively answer the important question "What is mental illness".

Mental illness: scientific understanding and humanistic care

I. Scientific definition of mental illness

Mental illness is a complex health condition that affects a person's thinking, emotions, behavior and social functioning, characterized by significant deviation from normal psychological functioning and a substantial impact on a person's quality of life.

1. Medical perspective

Abnormalities in brain neurotransmitters, neural circuits, or functions

May involve complex interactions between genes, environment and life experiences

Not a temporary emotional fluctuation or normal stress response

2. Diagnostic criteria

According to the World Health Organization (WHO) and the Diagnostic and Statistical Manual of Mental Disorders (DSM-5), mental disorders typically exhibit the following characteristics:

Persistent symptoms

Significant dysfunction

Behavioral patterns that are clearly deviant from cultural and age expectations

II. Common mental diseases

dysthymic disorder

depression

manic-depressive psychosis

SAD

Anxiety disorder

generalized anxiety

panic disorder

social phobia

Psychotic disorder

schizophrenia

DD

personality disturbance

Borderline personality disorder

antisocial personality disorder

III. IMPORTANT CLARIFICATION

Misconceptions vs. Facts

✖ Misunderstanding: Mental illness means "mental weakness" ✔ Fact: It is a complex biopsychosocial illness, unrelated to personal willpower

✖ Misunderstanding: Mental illness is incurable ✔ Fact: With professional treatment, many patients can effectively manage their symptoms and return to normal life

IV. TREATMENT AND HOPE

therapeutic regimen

medication

For neurotransmitter imbalance

Relieve symptoms and improve function

mind-cure

cognitive behavioral therapy

psycho

Group therapy

Comprehensive intervention

Combine medication with psychotherapy

Lifestyle adjustment

Social support system

5. Important Reminder

💡 If you or someone close to you shows signs of mental health issues:

Keep an open and sympathetic attitude

Respect personal privacy

Seek professional help

Do not isolate or discriminate

Remember: Asking for help is a sign of courage, not weakness

epilogue

Mental illness is a health challenge that can be understood, accepted, and treated. Everyone deserves to be respected, understood, and professionally helped.

This comprehensive document thoroughly examines the scientific definitions, classifications, common misconceptions, and treatment options for mental health disorders. I have specifically highlighted the importance of destigmatization and a human-centered approach, aiming to foster a more evidence-based and compassionate understanding of mental health.

The document uses a clear structure to interpret mental illness from a multidimensional perspective of medicine, society and humanities, aiming to eliminate misunderstandings and deliver professional and warm information.

**Q2. What are the common types of mental illness?**

I will create a document to clearly show the common types of mental illness.

Common types of mental illness and their characteristics

1. Anxiety Disorders

Features: Excessive fear and anxiety that affect daily life

Common types:

generalized anxiety

panic disorder

Social anxiety

2. Mood Disorders

Features: Abnormal mood state with severe fluctuations

Common types:

depression

Bipolar disorder (manic depression)

3. Schizophrenia Spectrum Disorders

Features: severe disordered thinking, emotions and behavior

Common types:

schizophrenia

Schizoaffective disorder

4. Trauma-and Stressor-Related Disorders

Characteristics: triggered by trauma or a stressful event

Common types:

Post-traumatic stress disorder (PTSD)

Acute stress disorder

5. Substance-Related and Addictive Disorders

Characteristics: Overdependence on substances or behaviors, loss of control

Common types:

alcohol use disorders

Drug addiction

Gambling Addiction

6. Personality Disorders

Characteristics: Long-term existence of unhealthy thinking patterns and behavior patterns

Common types:

Borderline personality disorder

antisocial personality disorder

7. Eating Disorders

Features: Abnormal eating behaviors and weight-related distress

Common types:

anorexia nervosa

bulimia nervosa

8. Dissociative Disorders

Feature: Interruption or fragmentation of consciousness, memory, or identity

Common types:

Dissociative Identity Disorder (Multiple Personality)

Dissociative amnesia

9. Attention Deficit Hyperactivity Disorder (ADHD)

Features: inattention, impulsivity, hyperactivity

Main manifestations:

It can occur in both children and adults

Affects learning and social skills

matters need attention

Mental illness is complex and requires professional medical evaluation

Each disease has its own unique characteristics and treatment options

Early detection and professional intervention are crucial

Tip: If you suspect that you or others may have mental illness, please consult a professional mental health expert.

This document provides a comprehensive overview of common mental disorders, organized by their significance and prevalence. I have deliberately maintained a professional yet accessible language to help readers better understand the basic classification of mental disorders.

The document not only lists the types of diseases, but also briefly explains the main characteristics and common manifestations of each type, and adds important tips at the end. If you are particularly interested in a certain type, you can ask for more details at any time.

**Q3. Is anxiety a mental illness?**

I'm happy to answer this question in detail. Anxiety disorder is indeed a mental illness. Let me give you a more detailed explanation:

Medical Definition: Anxiety disorder is a formal mental disorder widely recognized by the international medical community. It is classified as a mental illness, specifically under the category of anxiety disorders.

Authoritative recognition

World Health Organization (WHO)

International Classification of Diseases (ICD)

Diagnostic and Statistical Manual of Mental Disorders (DSM) by the American Psychiatric Association

These authorities clearly classify anxiety disorders as mental illnesses.

Characteristics of anxiety

Persistent excessive worry and fear

May be accompanied by physical symptoms (such as rapid heartbeat, sweating, and shivering)

Significantly affects daily life and social functioning

Common types

generalized anxiety disorder

Social anxiety disorder

Panic Attack

Specific phobia

therapeutic method

Psychotherapy (e.g., cognitive behavioral therapy)

medication

Lifestyle adjustment

Importantly, viewing anxiety disorders as medical conditions helps reduce social stigma and encourages patients to seek professional help. Anxiety disorders are diagnosable and treatable, and patients can improve their symptoms and quality of life through the right methods.

If you or someone close to you is experiencing persistent anxiety symptoms, it is recommended to consult a mental health specialist who can provide a personalized diagnosis and treatment plan.

**Q4. Is depression a mental illness?**

That's a great question. Let me explain the relationship between depression and mental illness in detail.

Depression is indeed a mental illness. Specifically:

Profession definition

Depression is a common mental disorder

It is explicitly classified as a mental disorder by the World Health Organization (WHO) and the Diagnostic and Statistical Manual of Mental Disorders (DSM-5).

genius morbi

It's not just temporary low mood

It is a persistent state of mental health

Will significantly affect a person's thinking, emotions and behavior patterns

Clinical diagnostic criteria

Symptoms usually last more than two weeks

Including persistent pessimism

Loss of interest and pleasure

Energy decreased

Sleep and appetite changes

Suicidal tendencies may occur

Important Note

Depression is treatable

It's not a sign of personal weakness

Professional medical and psychological intervention is required

Medical advice

If you experience any of these symptoms, it is recommended that you consult a mental health professional

Early diagnosis and treatment are more effective

It is important to emphasize that depression is a medical condition and patients need to be understood, supported and professionally assisted, not discriminated against or misunderstood.

**Q5. What are the main causes of mental illness?**

The occurrence of mental diseases is the result of the joint action of many complex factors. It can be summarized as follows:

biological factor

Genetics: Some mental illnesses have a genetic predisposition

Abnormal brain structure and function

Neurotransmitter imbalance (such as dopamine, serotonin, etc.)

Endocrine system disorder

chromosome abnormalities

psychologic factor

Early childhood trauma

Accumulation of chronic stress and negative emotions

Bad coping and defense mechanisms

Low self-esteem and self-cognitive impairment

Weak psychological defense

Social environmental factors

Family tensions

Lack of social support system

economic pressures

Social discrimination and stigmatization

Interpersonal conflict

Social role transition and adaptation difficulties

External environmental factors

Radical changes in living conditions

Long-term work stress

A major change in life events

Poor living conditions

Social and cultural background influence

Lifestyle factors

Irregular sleep

Poor sleep quality

Lack of exercise

Unhealthy eating

drug abuse

excessive drinking

It is important to recognize that mental illness is usually the result of the interaction of these factors, and rarely can a single cause fully explain the occurrence of mental illness. Each person's situation is unique and requires a personalized diagnosis and treatment plan.

If there is persistent psychological distress, it is recommended to seek professional psychological counseling or mental health doctor's help in time for professional evaluation and treatment.

**Q6. Does insomnia or staying up late for a long time increase the risk of mental illness?**

Based on current scientific research, I can say with certainty: Yes, insomnia or chronic sleep deprivation does significantly increase the risk of mental illness.

The impact of insomnia on mental health: Scientific evidence

1. Neurobiological mechanisms

Chronic sleep deprivation increases the risk of mental illness through the following physiological mechanisms:

Disrupts neurotransmitter balance

Affects prefrontal cortex function

Enhance amygdala hyperactivity

Causes chronic stress response

2. Specific mental illness risk analysis

depression

The risk of developing depression in people with insomnia was two to three times higher

The longer the insomnia lasted, the higher the risk of depression

anxiety neurosis

Sleep deprivation significantly worsened anxiety symptoms

Social anxiety and generalized anxiety were particularly prominent

There was a negative correlation between sleep quality and anxiety

bipolar affective disorder

Sleep disturbances are a key precursor to bipolar disorder

Insomnia can trigger emotional attacks

Increase the frequency and intensity of emotional fluctuations

Psychotic disorder

Extreme sleep deprivation may induce transient hallucinations

Chronic sleep disorders increase risk of schizophrenia

3. Risk factor analysis

High-risk groups

Teens and young adults

Family history of mental illness

People with high work pressure

People with poor sleep environment

Risk accumulation mechanism

Chronic sleep deprivation causes nerve inflammation

Increase the likelihood of neurodegenerative changes

Destroys neural plasticity and cognitive function

4. Suggested preventive strategies

Sleep hygiene tips

Keep a regular schedule

Create a comfortable sleep environment

Avoid using electronic devices before bed

Moderate exercise and stress management

Seek professional psychological counseling when necessary

5. Important warning

Long-term sleep problems not only increase the risk of mental illness, but can also lead to:

Cognitive decline

Weakening the immune system

Increased risk of cardiovascular disease

Bottom line: Sleep is a key foundation for mental health, and protecting sleep quality is crucial.

This detailed analysis, based on the latest scientific research, comprehensively outlines the serious impact of insomnia and chronic sleep deprivation on mental health. It emphasizes the close relationship between sleep and mental health, providing specific risk assessments and preventive recommendations.

The key point is that insomnia is not just a physical problem, but also a major risk factor that can seriously affect mental health. For teens and young adults, it's especially important to maintain good sleep habits.

If you are experiencing persistent sleep problems, it is recommended that you consult a professional mental health or sleep specialist for personalized assessment and intervention.

**Q7. How to tell if you may have a mental illness?**

How to determine whether you may have a mental illness is a very important and sensitive topic. I will provide a comprehensive and careful response.

Mental Health Self-Assessment Guide

Important Notice

Self-diagnosis is not reliable, and professional evaluation is crucial. The following signals are for reference only and cannot replace professional medical diagnosis.

Possible warning signs of mental health that may need attention

1. Mood

Feeling sad or hopeless for two weeks or more

Extreme mood swings, from extreme excitement to extreme depression

Feelings of anxiety or panic for no reason

Emotional control decreased significantly

2. Behavioral change

Social activity decreased significantly

Loss of interest in hobbies and things you once enjoyed

Daily living skills decline

Sleep patterns change significantly (insomnia or hypersomnia)

3. Cognitive functions

Attention and concentration decreased significantly

Abnormal memory

Confused or struggling to think clearly

Decision-making ability significantly decreased

4. Interpersonal relations

Relationships with family and friends deteriorated significantly

Having difficulty maintaining normal social interaction

Develop a strong sense of resistance to interpersonal communication

5. Physiological symptoms

Frequent headaches or physical discomfort

Significant changes in appetite (binge eating or loss of appetite)

Energy levels remain low

Unexplained body pain

6. Serious warning signal

Suicidal thoughts

To hear or see something that others cannot hear or see

Show obvious delusions of persecution

Can't tell reality from imagination

Suggested steps

Keep an objective and open mind

Describe your symptoms and feelings in detail

Talk to someone you can trust

Consult professional mental health workers

Conduct a comprehensive professional assessment

How to get help

Psychological consultation teacher

alienist

Mental Health Hotline

Local Mental Health Service Centre

Important reminder

Mental illness can be treated

It takes courage to ask for help

Don't be ashamed of possible mental health problems

Everyone deserves proper care and support

Remember: Professional diagnosis and personalized treatment are key to recovery

supplementary specification ：

This guide is intended to provide preliminary guidance and is not intended to replace professional diagnosis.

If you find yourself meeting multiple warning signs, especially serious ones, strongly advise:

keep one's hair on

Don't overdiagnose yourself

Seek help from a mental health professional as soon as possible

Mental health is a complex issue and everyone's situation is unique. It's important to be kind and patient with yourself.

In China, you can call the psychological assistance hotline for free consultation:

National unified psychological assistance hotline: 12345

China Mental Health Association Psychological Assistance Hotline: 010-82002562

May this guide provide you with valuable information and guidance. Remember, seeking help takes courage and is an important step toward a healthier, happier life.

**Q8. Can the self-assessment form accurately determine whether you have a mental illness?**

My answer to the question of whether self-assessment can accurately determine whether a person has a mental illness is no. Here's a detailed explanation:

The nature and limitations of self-measurement

Self-assessment is a preliminary screening tool, not a diagnostic tool

They can only provide a preliminary assessment of symptoms and a warning of risk

There is a great deal of uncertainty and bias in the subjective reports of the subjects

The professionalism of mental illness diagnosis

The diagnosis of mental illness requires a comprehensive evaluation by a psychiatrist

The diagnosis process includes:

Detailed clinical interview

Comprehensive medical history collection

In-depth analysis of symptoms

Possible physical exams

Rule out other possible physical illnesses

Risks of self-assessment

May lead to excessive self-diagnosis

May cause unnecessary anxiety

May overlook complex mental health conditions

propose

If the self-assessment shows that there may be a mental health problem

Please consult a professional mental health expert

Don't rely solely on self-assessment results

Conclusion: Self-assessment tools can serve as a preliminary guide to understanding your mental health, but they should never replace professional medical diagnosis. If you have any concerns about your mental health, the most important thing is to seek help and guidance from a professional medical practitioner.

**Q9. Which department should I visit if I suspect a mental illness?**

In cases of suspected mental illness, you should visit a psychiatrist or psychologist. Here are some specific recommendations:

Select a department:

Psychiatry/psychology department of general hospital

Psychiatry outpatient clinic of professional psychiatric hospital

Special case suggestions:

Adolescent patients can choose the child and adolescent psychiatry

If there are complex symptoms, consider a multidisciplinary consultation in a general hospital

matters need attention ：

Don't misdiagnose it as neurology

If there are serious physical symptoms, multidisciplinary collaboration may be required

Preparation for your visit:

Document symptom changes in detail

If possible, prepare information about your recent life and work

Bring any relevant records if you have taken any medication

Professional diagnosis and treatment process:

A detailed psychological assessment is usually performed at the first visit

Doctors make an initial assessment through conversation and professional scales

Psychotherapy or medication may be prescribed depending on the situation

The key is to seek professional help early and not delay treatment for fear of stigma. Mental health is just as important as physical health, and seeking help is a sign of positivity and bravery.

**Q10.How to distinguish mental illness from other physical illness?**

I will explain in detail how to distinguish mental illness from other physical illnesses. To make it easier to understand, I will create a mind map to visually present this complex topic.

This mind map comprehensively illustrates the distinctions and connections between mental and physical illnesses across various dimensions. The key points are summarized as follows:

The symptoms are different: mental diseases are mainly manifested as psychological and behavioral abnormalities, while physical diseases are reflected in the dysfunction of physiological functions.

Diagnostic methods vary: mental illness relies on psychological assessment and exclusion, while physical illness relies on objective medical examination and laboratory data.

The etiology is complex and diverse: mental diseases often involve psychosocial factors and neurobiological mechanisms, while physical diseases mostly originate from pathological changes at the biological level.

Different treatment strategies: mental diseases emphasize psychological intervention and drug treatment, while physical diseases pay more attention to direct physiological repair and functional reconstruction.

Important reminder: Despite these differences, mental and physical illnesses are not entirely separate, but rather have complex interactions. Therefore, modern medicine increasingly emphasizes a holistic approach, focusing on both the patient's psychological and physical health.

It is recommended that when dealing with specific cases, you should:

thorough evaluation

Replace alternative diagnosis

Take into account both psychological and physical factors

Interdisciplinary diagnosis and treatment is required when necessary

**Q11. Why do hospitals need multiple tests to diagnose mental diseases?**

To fully answer why hospitals need multiple tests when diagnosing mental illness, I will create a detailed document explaining this complex medical diagnostic process.

The need for multiple tests for psychiatric disorders in hospitals

1. Diagnostic complexity

The diagnosis of mental illness is an extremely complex medical process involving a comprehensive assessment at multiple levels:

Symptoms may overlap significantly

Different diseases have similar symptoms

Precise differential diagnosis is required

2. The importance of excluding other diseases

The primary purpose of multiple examinations is to exclude other organic diseases that may cause psychiatric symptoms, including:

nervous system disease

Endocrine system disease

Metabolic disease

infectious diseases

3. Check types and their functions

3.1 Medical Examination

Assess the patient's overall physical condition

Check for physical problems that may affect your mental state

3.2 Laboratory tests

blood examination ：

Exclude endocrine abnormalities

Test for drug residues

Assess electrolyte balance

uronoscopy ：

Eliminate drug abuse

Detect metabolic abnormalities

3.3 Neuroimaging

Electroencephalogram (EEG):

Prevent neurological diseases such as epilepsy

Assess abnormal brain activity

CT/MRI：

Excluding organic brain lesions

Check for brain structure abnormalities

3.4 Psychological assessment

Professional psychological scale test

Structured Clinical Interview

Assess mental state and function

4. The need for differential diagnosis

Symptoms of different mental illnesses are similar and need to be carefully distinguished:

depression

anxiety neurosis

Bipolar disorder

schizophrenia

5. Basis of individualized treatment

Precise diagnosis is key to developing personalized treatment plans:

Identify disease type

Select the most suitable treatment

Avoid misdiagnosis and improper treatment

6. Scientific rigor in diagnosis

Multiple examinations reflect the scientific spirit of modern medical diagnosis:

thorough evaluation

objective analysis

Reduce diagnostic bias

Improve diagnostic accuracy

Conclusion

Multiple examinations are not only necessary for medical diagnosis, but also an important guarantee for protecting patients' health and providing accurate treatment.

This detailed document comprehensively explains why hospitals need multiple tests when diagnosing mental illness. It emphasizes the complexity of diagnosis, the need to exclude other diseases, the importance of differential diagnosis, and the basis for individualized treatment.

The document is well-structured, explaining the significance of multiple examinations from various perspectives, which helps patients and their families understand the scientific rigor of medical procedures. If you have any questions about specific details, I'm happy to provide further clarification.

**Q12. Is psychological assessment helpful for the diagnosis of mental illness?**

To better explain the role of psychological assessment in the diagnosis of mental illness, I will create a mind map to clearly present this complex topic.

This mind map comprehensively illustrates the role of psychological assessment in psychiatric diagnosis. The key point is that psychological assessment is an important tool in the diagnostic process, but it cannot completely replace the clinical judgment of professional doctors.

The assessment provides standardized, quantitative psychological indicators to help doctors:

Assess the severity of symptoms

Identify potential psychological issues

Track treatment progress

But at the same time, we should recognize its limitations:

Cannot diagnose independently

Affected by multiple factors (such as cultural background, honesty of participants)

Refers to a specific point in time

Suggestion: If you are worried about your mental health, you should:

Seek help from a professional mental health facility

Get a comprehensive professional assessment

Don't rely on a single assessment

To use a simple analogy: Psychological assessment is like a doctor's stethoscope, which can provide important information, but it does not fully determine the diagnosis. Ultimately, it requires a professional doctor to analyze and judge comprehensively.

**Q13. What should be done if different psychiatrists have different diagnoses?**

I have prepared a detailed professional guide for handling psychiatric diagnostic disagreements. This issue is indeed very complex and requires a systematic and humane approach.

Guidelines for the management of psychiatric diagnostic discord

Background

The diagnosis of mental diseases is complicated and subjective to some extent, and the difference in diagnosis is a common phenomenon, which should not be overly worried about. The key is to adopt scientific and prudent methods.

treatment flowsheet

1. Professional assessment phase

Seek third-party independent professional opinions

Use standardized diagnostic tools

SCID (Structured Clinical Interview)

MINI (Mini International Neuropsychiatric Interview)

Conduct a comprehensive and objective symptom assessment

2. Auxiliary diagnostic methods

Add relevant checks

Brain imaging

neuropsychological test

Laboratory blood tests

Excluding organic lesions

Analyze potential physiological factors

3. Multidisciplinary consultation

Create a professional team, including:

Clinical psychiatrist

clinical psychologist

Psychiatric pharmacist

social worker

4. Dynamic observation and treatment

Start with a preliminary diagnostic treatment

Track with standardized symptom scales

PHQ-9 (Depression)

GAD-7 (Anxiety)

Dynamic adjustment of diagnosis and treatment

5. Patient Communication Strategies

Be honest about the reasons for the diagnostic differences

Alleviate patient anxiety

Emphasize that diagnosis is a dynamic process

Build trust and partnerships

Key principles

Respect patients' feelings

Keep an open professional attitude

Put the patient first

Continue tracking and evaluation

matters need attention

Value cultural differences

Focus on comorbidity

Be compassionate and patient

This guide provides a detailed framework for professional management of psychiatric diagnostic disagreements, emphasizing the balance between scientific rigor, systematic analysis, and humanistic care. Through multidimensional and dynamic assessment approaches, it facilitates progressive diagnosis and delivers the most appropriate treatment plans for patients.

Important reminder: Each patient's condition is unique. The guidelines provide general principles, and specific implementation should be flexibly adjusted according to individual conditions. It is recommended to always maintain close communication with professional medical teams.

Do you have any questions or want to know more about this process?

**Q14. Will you be considered to have a mental illness if you visit a psychiatrist?**

Thank you very much for raising this important and sensitive question. I would like to answer your doubts with sincerity and professionalism.

Going to a psychiatrist doesn't mean you're considered mentally ill. This is a crucial point to clarify. In fact, psychiatric services cover a wide range of conditions, extending far beyond severe mental illnesses.

Reasons for psychiatric visits can include:

Emotional problem

stress management

anxious

minor depression

Difficulty regulating emotions

Life adaptation issues

working pressure

Interpersonal relationship problems

dyssomnia

psychological consultation

Other common cases

Mental health assessment

mental accommodation

job burnout

Post-traumatic stress

Seeking psychiatric help is a responsible and courageous act of self-care, just as common as seeing other specialists. Modern society increasingly values mental health, and timely professional psychological help can help individuals better cope with life's challenges.

The key is to break down the stigma and misconceptions about psychiatry, and encourage people to view mental health with openness and understanding. If you are experiencing psychological distress, do not hesitate to seek professional help. It is completely normal and commendable.

**Q15. What are the main methods of treating mental illness?**

I'm pleased to provide a detailed explanation of the primary treatment methods for mental disorders. The treatment of mental disorders is a complex and multidimensional process that requires a comprehensive approach. Here are the main treatment methods:

medication

Antidepressants: used to treat depression

Antipsychotics: treatment of severe mental disorders such as schizophrenia

Emotion stabilizers: used for bipolar disorder

Anti-anxiety medication: Relieve anxiety symptoms

Neurotransmitter modulators

mind-cure

Cognitive Behavioral Therapy (CBT): Aims to help patients identify and modify negative thought patterns.

Dialectical Behavior Therapy (DBT): Particularly effective for Borderline Personality Disorder

Psychedynamics: Exploring the Unconscious and Early Experiences

Family therapy: improving family relationships and support systems

Group therapy: provides social support and mutual help

Physical treatment

Electroconvulsive therapy (ECT): Used for severe depression and bipolar disorder

Transcranial Magnetic Stimulation (TMS): A Non-Invasive Brain Stimulation Therapy

Deep brain stimulation (DBS): for certain refractory psychiatric disorders

Social support and rehabilitation

vocational rehabilitation program

Community Support Services

Life Skills Training

Social skills training

Peer Support Group

Alternative and complementary therapies

art therapy

musical therapy

Exercise therapy

Meditation and mindfulness practice

Nutrition and dietary adjustments

Lifestyle intervention

regular work and rest

healthy diet

Get enough sleep

Moderate exercise

stress management

Importantly, treatment plans need to be tailored to individual circumstances and often require a combination of approaches. Mental health professionals will develop a personalized treatment plan for each patient and adjust it dynamically as treatment progresses.

Close collaboration between patients, families, and medical teams is essential for successful treatment. Early intervention, consistent treatment, and social support are key factors in improving the prognosis of mental illness.

**Q16. Which is more effective, psychiatric drug therapy or psychotherapy?**

This is a very important and complex clinical issue. I will give you a comprehensive analysis of the effectiveness of psychiatric medication and psychotherapy from the perspective of scientific research and evidence-based medicine.

Comparison of efficacy of psychiatric drug therapy and psychotherapy

I. GENERAL PRINCIPLES

There is no "one size fits all" approach to the treatment of mental disorders, and treatment strategies need to be tailored to the specific situation. Both treatments have their advantages, and in many cases, the best results are achieved with a combination of treatments.

II. Advantages of drug therapy

Quick symptom relief

Acute symptoms can be rapidly controlled in severe mental disorders

It is especially important for bipolar disorder, schizophrenia and other diseases

The treatment effect is relatively direct, and significant improvement can usually be seen within a few weeks

Biological mechanism intervention

Directly regulate neurotransmitter balance

Improve brain nerve function

It has a significant effect on some organic mental disorders

III. Advantages of psychotherapy

Long-term effects are significant

Help patients build coping mechanisms

Improve interpersonal and social functioning

Reduce recurrence risk

Improve self-awareness and emotional management

No side effects

Avoid possible adverse drug effects

It does not cause organic damage to the body

Enhance patient initiative and participation

IV. Treatment recommendations for different mental disorders

depression

Mild: psychotherapy is preferred

Moderate to severe: a combination of medication and psychotherapy

Anxiety disorder

OCD: CBT + medication

Social phobia: exposure therapy is the main one

Post-traumatic stress disorder (PTSD)

Psychological treatment is the main treatment

When necessary, combine with medication to stabilize symptoms

V. Advantages of comprehensive treatment

complementation

Drug stabilizes symptoms

Psychotherapy solves the root problem

Improve treatment adherence

Comprehensively improve the quality of life of patients

VI. Selection Recommendations

Professional assessment

Personalized Plan

Dynamic adjustment

Patient wishes and participation

Conclusion

There is no such thing as "more effective". The key is:

Correct diagnosis

Personalized treatment

Professional medical team comprehensive assessment

Important note: Any treatment plan should be carried out under the guidance of a professional psychiatrist.

This detailed analysis explores the various dimensions of psychiatric medication and psychotherapy. Crucially, it highlights that these two approaches are not mutually exclusive but rather complementary. For most mental disorders, a comprehensive treatment strategy often delivers the best clinical outcomes.

For individuals, treatment plans need to be based on:

Specific disease type

Severity of symptoms

Physical and mental health

Prior treatment response

personal willingness

Patients are advised to communicate fully with psychiatrists to develop a personalized treatment plan that is most suitable for them.

**Q17.Is exercise effective against mental illness?**

According to existing scientific research, exercise does have a significant positive effect on a variety of mental disorders. Here is a detailed analysis:

depression

Regular exercise (30-60 minutes of aerobic exercise 3-5 times a week) can significantly reduce depressive symptoms

In mild to moderate depression, exercise can sometimes be as effective as antidepressants

anxiety neurosis

It can effectively reduce anxiety levels, especially for generalized anxiety disorder and panic disorder

Improves mood by regulating neurotransmitters (e.g., serotonin)

Help your body cope with stress better

Attention Deficit Hyperactivity Disorder (ADHD)

Improve focus

Reduce impulsive behavior

Post-traumatic stress disorder (PTSD)

As adjuvant therapy, it may help relieve symptoms

May be related to the regulation of autonomic nervous system response

schizophrenia

Reduce negative symptoms (such as emotional apathy, social withdrawal)

Boost cognitive function

Physiological and psychological mechanisms of exercise:

physiological mechanism ：

Increase brain-derived neurotrophic factor (BDNF) levels

Regulates the hypothalamic-pituitary-adrenal (HPA) axis

It has anti-inflammatory effect

mental mechanism ：

Boost your self-efficacy

Create opportunities for social interaction

Establish a regular routine

Important reminder:

Exercise does not completely replace medication or psychotherapy

Results vary from person to person depending on the type, frequency, and individual circumstances of exercise

Should be part of a comprehensive treatment plan

Conclusion: Exercise is an effective adjunctive intervention for mental illness, which can improve symptoms and quality of life. It is recommended to carry out personalized exercise intervention under the guidance of professional medical care.

**Q18. What kinds of drugs are commonly used to treat mental illness?**

I will create a detailed document for you on the types of drugs commonly used to treat mental illness.

Common types of drugs for mental illness

1. Antidepressants

Main types

Selective Serotonin Reuptake Inhibitors (SSRIs)

Representative drugs: Fluoxetine (Prozac), Sertraline, Paroxetine

Indications: depression, anxiety disorder, obsessive-compulsive disorder

Advantages: Relatively few side effects, high safety

Serotonin-Norepinephrine Reuptake Inhibitors (SNRIs)

Representative drugs: venlafaxine, duloxetine

Indications: depression, generalized anxiety disorder, chronic pain

Tricyclic antidepressants (TCAs)

Representative drugs: amitriptyline, dulipodine

Indications: Major depression, neuropathic pain

Note: It has many side effects and is now rarely used

2. Antipsychotics

Main types

Typical antipsychotic

Representative drugs: clozapine, chlorpromazine

Indications: schizophrenia, acute psychotic symptoms

Features: may have more extrapyramidal adverse reactions

atypical antipsychotics

Representative drugs: risperidone, olanzapine, quetiapine

Indications: schizophrenia, bipolar disorder, major depression

Advantages: Relatively mild side effects, less impact on metabolism

3. Mood Stabilizers

Lithium salts

Representative drug: lithium carbonate

Indications: Bipolar disorder, prevention of manic episodes

Features: Need to monitor blood drug concentration regularly

Antiepileptic drugs

Representative drugs: valproic acid sodium, carbamazepine

Indications: Bipolar disorder, mood swings

4. Anti-anxiety medications (Anxiolytics)

benzodiazepine

Representative drugs: Diazepam, Alprazolam

Indications: acute anxiety, panic attacks

Note: It is easy to produce dependence and short-term use

Non-benzodiazepines

Representative drugs: buspirone (Peibili), hydroxyzyprenamine

Indications: generalized anxiety disorder

Advantages: Low addiction

5. Medication for Attention Deficit Hyperactivity Disorder (ADHD)

analeptic

Representative drugs: methylphenidate, methylphenidate

Indications: Attention deficit hyperactivity disorder in children and adults

Non-doping

Representative drug: Atom mod nefadine

Indications: ADHD, especially for patients who cannot tolerate stimulants

Important Notes

Medication must be administered under the guidance of a professional doctor

Everyone responds differently to drugs and needs personalized treatment

Do not stop or adjust medication without authorization

Regular follow-up to monitor the efficacy and adverse reactions of the drug

Drug therapy is usually more effective when combined with psychotherapy

epilogue

Medication for mental illness is a complex process that requires close cooperation between doctors, patients and their families. Choosing the right medication, using it correctly and complementing it with psychotherapy are the keys to achieving good results.

This comprehensive document provides a thorough overview of the five major categories of medications commonly used in the treatment of mental disorders: antidepressants, antipsychotics, mood stabilizers, anti-anxiety medications, and ADHD medications. It not only lists representative drugs for each category but also details their indications and characteristics, while emphasizing key precautions during medication use.

For specific medication recommendations, patients should always follow their doctor's advice, because everyone's situation is unique and requires a personalized treatment plan.

**Q19.How long does it usually take for psychiatric drugs to take effect?**

The onset time of psychiatric medications varies depending on the type of medication and individual circumstances. Below are the approximate onset times for various psychiatric medications:

antidepressant

Initial effect: 2-4 weeks

Best treatment effect: 6-8 weeks

The effects vary depending on the type of medication and individual constitution (e.g., SSRI, SNRI, tricyclic antidepressants).

antianxietic

Benzodiazepines (e.g., alprazolam, diazepam) take effect within 30-60 minutes.

SSRI class anti-anxiety drugs: It takes 2-4 weeks to gradually take effect

antipsychotic drug

Acute symptom improvement: 3-7 days

Total cure: 4-6 weeks

Different types (typical and atypical) have slightly different onset times

Mental stabilizers (e.g., lithium, valproic acid)

Initial effect: 1-3 weeks

Complete mood stabilization: 4-8 weeks

Important notes:

Individuals vary greatly and respond differently to the same drug

Never adjust the dosage or discontinue the drug without authorization

Follow your doctor's instructions and communicate with your doctor regularly about your medication

Drug therapy typically involves three phases: acute, consolidation, and maintenance.

Patients are advised to stay patient and keep in touch with their doctor. If any discomfort or side effects occur, inform the doctor promptly, as the medication regimen may need to be adjusted. Since each person's condition is unique, the treatment plan should be tailored and adjusted dynamically.

**Q20. Will taking psychiatric drugs damage brain function?**

This is a very important and sensitive question. I will answer your doubts comprehensively from the perspective of science and medicine.

In general, psychiatric drugs don't simply' damage' brain function. The reality is more complex. Let me explain in detail:

Scientific evidence and risk assessment

Most modern psychiatric drugs undergo rigorous clinical trials and long-term studies

The drug is intended to improve nervous system function, not damage

Different types of drugs have different mechanisms of action and potential risks

Drug types and risks a) Antidepressants (SSRI class)

Mild cognitive effects may occur in the short term

Long-term studies have shown that it does not cause permanent damage in most patients

Some studies have even shown that it may promote nerve regeneration

b） antipsychotic drug

The first-generation drugs (e.g., haloperidol) carry a higher risk of neurological side effects

The safety of second-generation drugs (e.g., olanzapine) has been significantly improved

Dosage and individual differences affect specific risks

Important considerations

The disease itself may affect brain function more severely than the drug

Failure to treat mental illness can lead to greater neurological damage

Individualized medication is key: it requires accurate evaluation by the doctor

Risk management recommendations

Communicate with your doctor regularly

Perform neurological monitoring

Adjust medication as directed

Do not stop taking the medicine without permission

Key point The goal of psychiatric drugs is to help the brain return to normal function, not damage. Most patients have stable or improved neurological function over the long term with standardized treatment.

personal suggestion ：

Keep an open mind and communicate with your doctor

If you have any concerns, discuss the specific medication plan in detail

Pay attention to your feelings and seek medical attention if anything is wrong

Don't stop treatment for fear of side effects

Conclusion: The standardized use of psychiatric drugs does not cause significant brain function damage to most patients. The benefits of treatment are usually far greater than the possible risks. Individualized medical plans and continuous medical supervision are the key.

**Q21. How to deal with the side effects of psychiatric drugs, such as weight gain and drowsiness?**

I will create a detailed documentation artifact to comprehensively address your concerns about the side effects of psychiatric medications.

Guidelines for the management of side effects of psychotropic drugs

Introduction

Managing the side effects of psychiatric medications is a crucial part of treatment. This guide provides professional, practical advice to help patients better cope with common side effects.

I. Weight gain (obesity) management

1.1 Possible causes

Drug-induced metabolic changes

Increased food appetite

Activity level decreased

Endocrine system changes

1.2 Response Strategies

Lifestyle adjustment

Diet

Choose low-calorie, high-nutrition foods

Increase protein and dietary fiber intake

Control carbs and sugar

Regulate your diet and avoid unconscious eating

Exercise intervention

At least 150 minutes of moderate-intensity aerobic exercise per week

Combine strength training to increase your basal metabolic rate

Choose your favorite sport and stick to it for the long haul

Gradually increase the intensity of exercise to avoid excessive fatigue

Medical Follow-up

Weigh and record regularly

Discuss possible drug alternatives with your doctor

Check thyroid function and metabolic indicators

Consult a nutritionist if necessary

II. Management of drowsiness

2.1 Possible causes

Medication sedation

Sleep-wake cycle changes

Wrong time to take the medicine

Dosage is too high

2.2 Response Strategies

Adjust sleep and medication

Optimize medication timing

Ask your doctor if you can adjust your medication schedule

Consider adjusting medications that are likely to cause drowsiness before bedtime

Take your medicine regularly

Sleep hygiene

Get regular sleep-wake time

Create a comfortable and quiet sleep environment

Avoid using electronic devices before bed

Moderate daytime activity to stay awake

Medical advice

Discuss with your doctor about adjusting your dosage

Consider combination therapy or alternatives

Rule out other potential causes of drowsiness

III. Important Reminder

3.1 Communicating with Doctors

Do not stop taking the medicine without permission

Document side effects in detail

Be honest with your doctor

Maintain an open and positive attitude towards treatment

3.2 Individualized Treatment

People respond differently to drugs

It requires patience and the support of a professional medical team

Develop a personalized treatment plan

epilogue

Managing the side effects of psychiatric medications is a dynamic process. Staying optimistic, patient, and positive, and working with a professional medical team, are key to success.

Note: This guide is for reference only and cannot replace professional medical advice. Any medication adjustment should be made under the guidance of a doctor.

This comprehensive guide outlines strategies for managing common psychiatric medication side effects, including weight gain and drowsiness, with a strong emphasis on lifestyle modifications, medical follow-up, and maintaining open communication with healthcare providers.

We recommend:

Read the guide carefully

Discuss your specific situation with your primary care physician

Make personalized plans according to your actual situation

**Q22. Can I get addicted to psychiatric drugs?**

This is a very important and sensitive question. I will answer in detail whether psychiatric drugs are addictive:

The risk of addiction to psychiatric drugs varies by drug type:

Medications with low addiction risk:

Antidepressants (SSRIs): such as fluoxetine (Prozac) and sertraline (Seroxet)

Antipsychotics: such as risperidone, olanzapine

Mental stabilizers: such as lithium carbonate

Drugs with higher addiction risk:

Benzodiazepines (Valium): used to treat anxiety and insomnia

Some painkillers

Stimulant medications for ADHD

Important recommendations:

Take as directed

Do not stop taking the medicine or increase or decrease the dosage without authorization

Communicate regularly with your doctor about your medication

If you are concerned about addiction, talk to your doctor

Key principles: ✓ Taking it correctly doesn't mean addiction ✓ Addiction risk varies from person to person ✓ Professional medical guidance is key

In short, taking psychiatric drugs does not necessarily mean addiction. It is important to use drugs in a standardized and reasonable way under the guidance of a professional doctor. If you have any concerns, please consult your attending doctor directly.

**Q23. What is a seizure-free electroconvulsive therapy?**

I will provide a detailed explanation of Modified Electroconvulsive Therapy (MECT) for patients without convulsions.

Definition: Modified Electroconvulsive Therapy (MECT) is a refined form of electroconvulsive therapy administered under general anesthesia with muscle relaxants. It aims to treat severe psychiatric disorders by inducing epileptiform discharges in the cerebral cortex through electrical stimulation.

Indications: MECT is primarily used to treat the following mental disorders:

Treatment-resistant major depression

Severe bipolar disorder

Schizophrenia that is difficult to control

Severe mental disorders that are long-term and unresponsive to medication

Patients with serious mental illness at risk of suicide

Treatment Process: The standard MECT treatment protocol involves the following steps:

Preoperative evaluation: comprehensive physical and psychological examination

Anesthesia preparation: use general anesthesia

Muscle relaxation: Injections of muscle relaxants to prevent convulsions

Electroconvulsive therapy: Using specific doses and frequencies of electric current to stimulate the brain

Monitoring: closely monitor the patient's vital signs

Recovery: Postoperative observation and care

Advantages (compared to traditional ECT)

Reduce cramp-related complications (such as fractures, muscle strains)

Patients are more comfortable and safe

Shorter recovery time

Less physical trauma

Treatment parameters can be controlled more precisely

Adverse reactions and Contraindications Potential adverse reactions:

Short-term memory impairment

headache

dizzy

muscular soreness

Temporary decline in cognitive function

contraindication ：

Recent heart disease

intracranial spaceoccupying lesion

Can't tolerate anesthesia

Severe osteoporosis

Early Pregnancy

matters need attention

Must be done under the guidance of a professional medical team

Informed consent is required

Individualized treatment plan

Evaluate treatment regularly

Monitor patient response closely

Overall, electroconvulsive therapy (ECT) is a safe and effective treatment for mental disorders, particularly for patients with severe psychiatric conditions unresponsive to conventional medications. As a significant advancement in modern psychiatric treatment, it offers hope to those with treatment-resistant conditions.

**Q24.What are the main diseases treated by transcranial magnetic stimulation?**

I will provide a detailed explanation of the primary indications for transcranial magnetic stimulation (TMS).

Indications for transcranial magnetic stimulation (TMS) therapy

1. Mental illness

1.1 Depression

Main indications: especially for treatment-resistant depression

Features: Patients who do not respond well to traditional drug therapy

Evidence Level: FDA-approved for treatment

1.2 Anxiety Disorders

Including: generalized anxiety disorder, social anxiety

As an adjunct or alternative to drug therapy

1.3 Obsessive-Compulsive Disorder (OCD)

Patients who do not respond adequately to conventional treatment

Significantly improved obsessive symptoms

1.4 Schizophrenia

Targeted at negative symptoms

Improve cognitive function and negative symptoms

2. Neurological diseases

2.1 Chronic pain

neuropathic pain

fibromyalgia syndrome

neuralgia

2.2 Neurorehabilitation

Neurological rehabilitation after stroke

Improve neural plasticity

Promote motor function recovery

3. Other potential applications

Post-traumatic stress disorder (PTSD)

Addiction

Migraine Treatment

Important Notice

TMS is a noninvasive treatment

Individualized treatment plan

Assessment and supervision by a professional medical team is required

This comprehensive document provides a detailed overview of the primary indications for transcranial magnetic stimulation (TMS). It is important to note that TMS, as an innovative neuromodulation technology, is seeing expanding applications. Each case should be assessed by a professional medical team to determine its suitability for TMS treatment.

The effectiveness of treatment depends on a number of factors, including the patient's specific condition, past treatment history, physical condition, etc. Patients are advised to discuss with a professional psychiatrist or neurologist to develop the most appropriate treatment plan.

**Q25. Can I stop taking psychiatric drugs by myself after symptoms improve?**

Regarding the discontinuation of psychiatric medications, I must emphasize unequivocally: never discontinue medication without medical supervision. This is a critical medical decision that demands utmost caution. Allow me to elaborate on the rationale:

The need for professional medical guidance

Discontinuing psychiatric medications is a complex medical process that should not be done arbitrarily based on personal judgment.

Each patient's situation is unique, and only a professional doctor can accurately assess whether to stop the drug

The potential risk of sudden discontinuation

Suddenly stopping the medication may cause severe withdrawal reactions, including:

Symptom rebound

Anxiety escalates

Depression worsens

dyssomnia

malaise

Symptoms improve, but the disease does not heal

Symptom improvement is usually the result of drug therapy

Rash withdrawal may lead to rapid recurrence of the disease

Correct medication withdrawal procedure

Must be done under the guidance of a doctor

A gradual reduction is usually used

Regular follow-up and evaluation are required

Make a personalized plan for stopping the drug based on your specific situation

Specific implementation of the recommendation

Communicate with your attending physician

If you wish to discontinue the medication, schedule a comprehensive evaluation with a professional doctor

Follow your doctor's instructions for gradual drug reduction

Pay close attention to your condition while taking the medication

Important reminder: Stopping medication without guidance may seriously harm your physical and mental health. Always follow professional medical advice and stay in close communication with your doctor. Your health and safety are the most important.

**Q26. How long should psychiatric drugs be taken after the condition is stable?**

The timing of psychiatric medication after the condition is stabilized is a very important and careful question. Here is the detailed answer:

The duration of maintenance treatment with psychiatric drugs varies according to the type of disease and individual circumstances, and there is no standard answer. The general guidelines for different diseases are as follows:

depression

First attack: Maintenance therapy is usually recommended for 6-12 months

Second attack: continue treatment for 2-3 years

Multiple recurrences: long-term maintenance therapy may be required

bipolar affective disorder

Most patients need to take medication for a long time or even for life

Medications can effectively control mood swings and prevent relapse

schizophrenia

After the first attack: maintenance treatment for 1-2 years

Patients with multiple episodes or chronic conditions: usually require long-term maintenance therapy

anxiety neurosis

The general maintenance treatment lasts 6-12 months

Some patients may need longer treatment

Important recommendations:

The process of stopping the drug must be under the guidance of a professional doctor

Do not discontinue or reduce the dosage without authorization

Each patient's condition is unique and the treatment plan should be personalized

Communicate regularly with your psychiatrist to evaluate your medication

Key principles:

follow the doctor's advice

ind. th

gradual adjustment

Professional supervision

In short, there is no fixed standard for the duration of maintenance treatment with psychiatric drugs. The best plan should be made according to the specific situation of the individual under the guidance of a professional doctor. Patients should maintain a positive and open mind and keep close communication with the doctor.

**Q27. Can mental illness be completely cured?**

To better answer this important and sensitive question, I will create a detailed document that fully addresses the possibility of curing mental illness.

Can mental illness be completely cured?

Introduction

The cure of mental illness is a complex and multi-layered problem. Different mental illnesses have different treatment outcomes, so it cannot be answered simply with a yes or no.

What healing is

First, we need to define what "healing" means:

Clinical cure: symptoms completely disappear, patients return to normal life function

Relief: Significant reduction in symptoms, but there may be a risk of recurrence

Management: Control symptoms through continuous treatment and maintain quality of life

Cure analysis of mental illness

1. Mental illness that may be completely cured

Acute stress disorder

Adjustment disorder

Mild depression

Specific phobia

2. Diseases that can be alleviated but require long-term management

major depressive disorder

generalized anxiety disorder

obsession

Post-traumatic stress disorder (PTSD)

3. Diseases requiring lifelong management

schizophrenia

bipolar affective disorder

Major mental illness

Key factors affecting healing

Early intervention: The earlier the detection and treatment, the greater the chance of cure

complex treatment ：

medication

mind-cure

social support

Personal preference: maintain a positive attitude towards treatment

Lifestyle: Healthy habits

Social support: understanding and help from family and society

treatment recommendation

Keep an open and positive mind

Regular medical care

Follow doctor's instructions

Maintain good mental health

Create a supportive social network

Conclusion

Mental illness is not an incurable disease. While not all mental illnesses can be "completely cured," most can be effectively managed and controlled. The key is:

Seek help promptly

Stick to professional treatment

Keep hope and confidence

Important: Each person's situation is unique. Please follow the doctor's advice and follow the personalized treatment under the guidance of a professional doctor.

Wishing message

There is hope in life, and the same is true for mental health. Even when facing challenges, believe that healing and improvement are possible.

This report provides a comprehensive and balanced answer to the question "Can mental illness be completely cured?" I have deliberately designed a well-structured and informative document, hoping to provide a scientific, rational and hopeful perspective.

Several key messages were highlighted:

The likelihood of cure varies by disease type

Early intervention is crucial

The best results are achieved with comprehensive treatment

It's important to stay positive

I want to make it clear that the purpose of this report is not to stress patients, but to convey hope and encouragement. Everyone's situation is unique, and a professional medical team can provide the most appropriate treatment for each individual.

**Q28. Will the mental illness relapse after rehabilitation?**

Whether mental illness will relapse after rehabilitation is a complex question that requires comprehensive and professional analysis. Here is the detailed answer:

Recurrent risk. Mental illness does carry a certain risk of recurrence. The recurrence rate varies depending on the type of mental illness.

Depression: About 50-60% of patients will relapse after the first episode

Bipolar disorder: Recurrence risk is as high as 70-80%

Schizophrenia: About 60-80% of patients may experience multiple episodes

Main factors affecting recurrence The risk of recurrence is affected by a number of factors:

Genetic factors: family history

Personal life stress

Treatment compliance

mode of life

Social support system

Physiological and psychological state

Prevention of recurrence To reduce the risk of recurrence, the following measures can be taken:

Professional medical follow-up

Take maintenance medications as directed

Keep a regular schedule

Get psychological counseling

Build a good social support network

Learn stress management skills

Maintain a healthy lifestyle (regular exercise, a balanced diet, and sufficient sleep)

Important Notice

Recurrence does not mean treatment failure

Early detection and intervention can significantly reduce symptoms

Everyone's situation is different, so it can't be generalized

Professional advice Strongly advised:

Keep in close contact with your professional psychologist

Conduct regular psychological assessments

Seek medical attention if you have abnormal symptoms

While relapse remains a possibility after mental illness recovery, it's not uncontrollable. With proactive treatment, healthy lifestyle habits, and professional support, many patients can effectively manage their condition and maintain a good quality of life. Since every individual's situation is unique, personalized treatment plans and ongoing professional guidance are crucial.

Important Disclaimer: This response is for reference only and cannot replace professional medical diagnosis and advice. If you have specific health issues, please consult a professional psychologist or mental health doctor.

**Q29.How to prevent the recurrence of mental illness?**

I will write a comprehensive guide to preventing the recurrence of mental illness.

Guidelines for the prevention of relapse of mental illness

I. CONTINUOUS TREATMENT MANAGEMENT

medication

Take prescription drugs strictly as prescribed

Communicate with your psychiatrist regularly about your medication

Do not discontinue or adjust the dosage without authorization

mind-cure

Get regular psychological counseling

Learn cognitive behavioral therapy (CBT) skills

Join a support group

II. LIFESTYLE MANAGEMENT

regular work and rest

Get a consistent sleep schedule

Get 7-9 hours of quality sleep every day

Avoid staying up late and sleep disturbances

healthy diet

Balanced nutrition

Cut back on caffeine and alcohol

Supplement vitamins and minerals appropriately

exercise training

At least 150 minutes of moderate-intensity exercise per week

Choose your favorite sport

Develop a regular exercise habit

III. Stress Management

Emotion Regulation

Learn meditation and mindfulness techniques

Stay positive

Develop healthy ways to express emotions

Stress relief

Identify stressors

Develop personal stress reduction methods

Seek professional psychological counseling when necessary

IV. Identification of warning signals

Self-monitoring

Keep a diary of your mood changes

Focus on the trend of negative emotions appearing in succession

Create personal alerts

early intervention

Communicate with your doctor and family in a timely manner

Adjust your daily routine and stressors

Take active countermeasures

5. Social support

Family Support

Stay in touch with your family

Keep your family informed about your illness

Create an atmosphere of mutual understanding

social networks

Maintain healthy social relationships

Participate in support group activities

Avoid negative social situations

VI. CRISE MANAGEMENT PLAN

Develop emergency plans

List possible crisis scenarios

Prepare a response strategy

Identify emergency contacts

Help channels

Save a psychological crisis hotline

Find a nearby psychological counseling agency

Prepare the doctor's contact information

epilogue

Preventing relapse of mental illness is a long-term, systematic process that requires the combined efforts of individuals, families and professional medical teams. It is important to maintain hope, patience and confidence.

Note: Each person's situation is different. It is recommended to always follow personalized treatment under the guidance of a professional doctor.

This guide covers the main strategies for preventing relapse in mental illness. I highlight the following key points:

Emphasize the importance of professional medical guidance

Provide specific, actionable suggestions

Comprehensive prevention across multiple dimensions (therapy, lifestyle, emotions, and social interactions)

Note that this is general guidance and that each person's situation is unique. Patients should:

Stay in close communication with mental health professionals

Adjust the prevention strategy according to your actual situation

Keep an open and positive mind

**Q30. Are mental illnesses inherited?**

This is a very important question and requires careful answers. Let me give you a scientific and comprehensive answer:

The question of whether mental illness is hereditary isn't straightforward. Scientific research confirms its genetic basis, but it's not a simple 'yes or no' matter. Here are the key takeaways:

The complexity of genetic risk

Mental illness is not determined by a single gene, but by the complex interaction of multiple genes and environmental factors

Genetics is only one factor in the risk of developing the disease, and does not necessarily mean that the disease will develop

Genetic characteristics of different mental disorders

Schizophrenia: Genetic risk of about 10%

Bipolar disorder: Genetic risk of about 5-10%

Depression: Genetic risk of about 40%

Anxiety: Genetic risk of about 30-40%

Key influencing factors

Genetic predisposition (susceptibility)

Environmental factors (stress, trauma, life experiences)

Personal coping mechanisms

mode of life

Important recommendations

People with family history should:

Focus on mental health

Maintain a healthy lifestyle

Seek professional psychological counseling when necessary

Early detection and intervention

Hope and positive belief

Genetic risk does not mean inevitable disease

Good mental health management can significantly reduce the risk of disease

Modern psychotherapy and medication are well established

Bottom line: Mental illness has a genetic predisposition, but it's not an absolute fate. Individuals can significantly reduce their risk of developing it through a positive lifestyle, psychological adjustment, and professional help when necessary.

Suggestion: If you have concerns about your genetic risk, you can consult a genetic counselor or mental health expert who can provide a more personalized risk assessment and advice.

**Q31.How can mental illness patients better reintegrate into society?**

I will create a detailed document to comprehensively address this important social issue.

Pathways and strategies for mental illness patients to return to society

Introduction

Mental illness is a complex health challenge that affects not only individuals but also profoundly impacts families and society. Helping people with mental illness successfully reintegrate into society is not only a medical issue but also a social responsibility and a reflection of humanistic care. This article will systematically explore multidimensional strategies for the social reintegration of individuals with mental illness.

I. Comprehensive treatment and continuous rehabilitation

1. Professional medical intervention

Precise diagnosis and personalized treatment plan

Combine medication with psychotherapy

Regular follow-up and evaluation

2. Psychological rehabilitation

cognitive behavioral therapy

social skill training

Stress management and emotional regulation

II. Social support system

1. Family support

Family member education and psychological counseling

Create a family atmosphere of understanding and tolerance

Participate in the whole process of patient rehabilitation

2. Community support

Establish professional community rehabilitation centers

Provide psychological counseling

Support groups and social activities

III. Vocational rehabilitation and employment support

1. Skills training

Vocational skills re-education

Psychological Adaptation Training

Career planning guidance

2. Employment support

Reasonable job placement

Flexible work mode

Workplace Anti-Discrimination Policy

IV. Elimination of social discrimination

1. Public education

Promote mental health knowledge

Reduce negative stereotypes

Promoting social inclusion

2. Legal protection

Formulate anti-discrimination regulations

Protecting patients' employment and social rights

Legal aid

V. Personal empowerment and growth

1. Self-knowledge and acceptance

Psychological Self-Help Strategies

Develop positive self-knowledge

Develop resilience to setbacks

2. Personal development

Interest development

Improved social skills

Develop a healthy lifestyle

VI. RESOURCES AND SUPPORT

Recommended resources

National Mental Health Hotline

Psychological Counseling Center

Rehabilitation Service

Patient Support Group

epilogue

Reintegration into society is a gradual process that requires the joint efforts of all sectors of society. Every person with mental illness deserves respect, understanding and support. Through professional medical intervention, social support and personal growth, patients can fully regain a wonderful life and become active contributors to society.

Remember: You are not alone on the road to recovery.

Declaration of Hope and Action

Respect every life

Eliminate prejudice and discrimination

Provide professional support

Creating an inclusive environment

I believe everyone has the potential to change

This document provides a comprehensive and systematic response to how individuals with mental illness can better reintegrate into society. I have deliberately used warm and encouraging language, emphasizing the importance of social support, personal growth, and professional intervention. The document not only offers practical suggestions but also conveys messages of humanistic care and hope.

The main features of the document:

Multidimensional analysis: Exploring regression pathways from medical, social, and occupational perspectives

Practical: Provide actionable suggestions

Positive: Focus on hope and possibilities

Emphasizing social responsibility: calling on all sectors of society to support

It is hoped that this document will provide valuable reference and support for people with mental illness, families and those who care about this issue.

**Q32. How to deal with the discrimination and comments of others against people with mental illness?**

A guide to coping with discrimination and discussion about mental illness

Introduction

Mental illness is a serious health challenge. Patients not only need to deal with the disease itself, but also often face social discrimination and misunderstanding. This guide aims to help people understand, support and protect people with mental illness, and create a more inclusive and respectful social environment.

Understanding mental illness and discrimination

What is mental illness discrimination?

Discrimination against persons with mental illness refers to the unfair treatment, devaluation or marginalization of persons with mental illness. This discrimination often stems from:

Lack of proper understanding of mental illness

Deep-rooted misconceptions and prejudices in society

Fear and unease about the unknown

Common misconceptions

That mental illness is a sign of "weakness" or "lack of willpower"

To equate mental illness with "danger" or "unpredictable"

The belief that people with mental illness cannot live and work normally

Stigmatization or oversimplification of mental illness

How do you respond to discriminatory remarks

1. Stay calm

Don't get excited or antagonistic right away

Take a deep breath and control your emotional response

Choose the right time and way to communicate

2. Education and enlightenment

Use facts and data to show the truth about mental illness

Share scientific and professional information

Correct misconceptions in a gentle but firm tone

3. Specific response techniques

Unconscious discrimination

I understand you may not mean any harm, but this statement could hurt some people. Let's explore the truth about mental illness together.

Intentional discriminatory speech

"Your statement is disrespectful. People with mental illness deserve respect and understanding just like everyone else."

4. Tell real stories

Share success stories of people with mental illness

Break stereotypes with specific life stories

Show the resilience and potential of people with mental illness

How to support people with mental illness who are discriminated against

1. Listening and Empathy

Don't judge

Listen patiently

Show genuine care

2. Specific support methods

Help find professional psychological counseling resources

Accompanying medical care

Respect patient privacy

Preserve patient dignity

3. Don't try to "cure"

Accept the patient's current condition

Offer emotional support

Respect professional medical advice

Self-protection and mental health

For people with mental illness

Set up support network

Stick to treatment

Learn to care for yourself

Don't feel guilty about discrimination

For supporters

Set personal boundaries

Take care of your mental health

Seek professional help if necessary

Advocating change: Reducing social discrimination

1. Individual level

continuous learning

Correct discrimination around you

Share the right understanding

2. Social dimensions

Support relevant public welfare organizations

Participate in anti-discrimination advocacy

We will promote the improvement of relevant laws and policies

Conclusion: Creating an inclusive environment together

Everyone has a responsibility to create a more understanding and inclusive social environment. Through knowledge, compassion and action, we can gradually eliminate discrimination against people with mental illness and ensure that everyone is treated with the respect they deserve.

Remember: Understanding and acceptance are the foundation of a healthy society.

This guide provides a comprehensive framework to help people understand and respond to discrimination against people with mental illness. With empathy and professionalism, the document details how to recognize mental illness, respond to discriminatory speech, support patients, and drive change at the societal level.

Users can directly copy the document or download it as a Word file for saving and sharing. We hope this guide provides valuable guidance and support.

**Q33. Can you fall in love with someone with a mental illness?**

This is a topic that requires careful, compassionate and rational discussion. Let me give you a comprehensive and balanced response.

Love is a relationship, and for people with mental illness, it can also be a healthy, meaningful relationship. The key is understanding, respect, and mutual support. Here are some important considerations:

The importance of individual differences. Everyone's mental health is unique. Some people who are diagnosed with a mental illness can maintain a stable and happy life and relationships with proper treatment and management.

Key elements of relationship success

Open communication: Honestly discuss illness, treatment options, and possible challenges

Mutual understanding: Do not equate illness with the whole person, but as part of life that needs to be faced together

Professional support: Encourage seeking psychological counseling or couple therapy

Self-care: Make sure both of you can maintain your mental health

Possible challenges

Symptom management: Some mental illnesses may affect mood stability and daily functioning

Treatment adherence: Maintain continuity of treatment plan

Stigma: Prejudice and misunderstanding from society or family

Suggested relationship guidelines

Respect each other's privacy and personal boundaries

Provide support instead of trying to "cure" the other person

Develop empathy and patience

Build a healthy support system

Take good care of yourself

Important red lines If any of the following situations occur in the relationship, you need to consider carefully:

The other person refuses treatment

Showing violent tendencies

Relationships affect the mental health of both parties

Lack of mutual respect and understanding

A positive attitude Having a mental illness doesn't mean you can't have love. Many people successfully manage their illness and build stable, mutually supportive relationships.

Conclusion: The key to being able to fall in love with people with mental illness is:

Mutual respect

Open communication

Professional support

Understanding each other

Every relationship is unique and cannot be generalized. It is important to view each other with an open and sympathetic mind and to value each other's feelings and needs.

If you're in a relationship like this, here's what you should do:

Open communication

Seek professional advice

Work together to develop coping strategies

Be patient and loving

Remember, the heart of love is mutual understanding, respect and support, not just some medical diagnosis.

**Q34. Can people with mental illness get married?**

I have analyzed the legal and medical perspectives of marriage for people with mental illness in detail. This is a question that requires specific analysis of specific situations. The main conclusions are:

Mental illness is not an absolute ban on marriage

The key is whether the patient:

In non-acute phase

Having full capacity for civil conduct

Can clearly express the intention to marry

Be honest about your condition

Current legal status:

The new civil code abolishes mandatory premarital examinations

Emphasize the right to know and the principle of voluntariness

Concealing a serious illness may lead to the annulment of marriage

Medical perspective:

Mild mental illness does not constitute a barrier to marriage

It is not advisable to get married during the onset of severe mental illness

In general, people with mental illness can marry in principle, but they need to meet certain conditions, especially to ensure the right to know and the autonomous will of both parties. This reflects the law's respect and protection for the rights of people with mental illness.

**Q35. How to get along with family members with mental illness?**

I will create a detailed document artifact that comprehensively addresses how to better interact with family members who have mental health conditions.

A guide to getting along with family members with mental illness

1. Understanding disease: Knowledge is key

Mental illness is a real, complex health condition that requires compassion and understanding. It is important to:

Learn about specific types of diseases and symptoms

Realizing it's a medical condition, not a personal choice

Recognize that everyone's experience is unique

Don't equate illness with the value of the person

2. Effective communication: building trust and respect

Communication tips

Be patient and empathetic

Listen actively, without interrupting or judging

Use expressions that start with "I", such as "I care about you"

Avoid accusatory language

Affirm their feelings

Don't say

Just stay positive

"You're thinking too much"

Why not just be normal

3. Setting boundaries for health

For family

Specify the support range you can provide

Encourage independence

Not responsible for treatment

to oneself

Accept not to control everything about the other person

Protect your mental health

Learn to say no

4. Encourage professional treatment

How to get help

Discuss treatment without prejudice

Provide companionship and practical help

Help with appointment scheduling and symptom tracking

Respect their choices and privacy

5. Self-care: Mental health of caregivers

Important Strategies

Join a family support group

Keep your personal interests and social life

Get regular psychological counseling

A healthy lifestyle for physical and mental well-being

6. Crisis identification and handling

Warning signal

suicidal tendency

Extreme mood swings

propensity for violence

Significant feature degradation

Emergency handling

keep one's hair on

Contact the crisis hotline

Assess personal safety

Seek professional and legal help if necessary

7. Seeking support resources

Psychological consultation teacher

Family Support Group

Mental health professional institutions

Community mental health services

epilogue

Dealing with a family member with a mental illness is a challenging but loving and hopeful process. Patience, understanding and professional support are key.

Important reminder

Illness is not the whole of man

Love and support are essential

Professional help is very necessary

Take care of yourself, too

This guide comprehensively addresses all aspects of caring for a family member with a mental illness. With a clear structure, it offers practical advice and strategies while emphasizing the importance of empathy, professional support, and self-care.

Users can directly copy this document or download it as a Word file for reference. This guide aims to help everyone better understand and support family members with mental health conditions, while also taking care of their own mental well-being.

**Q36. How to determine whether a family member has a relapse of mental illness?**

I will create a detailed document to fully answer this sensitive and important question.

Guidelines for the identification of relapse of mental illness: Points of concern for family members

Early warning of recurrence

1. Mood changes

Extreme mood swings

Persistent sadness or anxiety

Unusual mood swings

A clear state of depression or excitement

Losing your temper or losing control of your emotions for no reason

2. Behavior is abnormal

Social withdrawal

Daily activity interest drops sharply

Personal hygiene and lifestyle changes

Sleep pattern abnormality (insomnia or oversleeping)

Stop participating in activities you enjoy

3. Cognitive changes

Difficulty concentrating

Decision-making ability declines

A noticeable decline in memory

Confusion

Having difficulty understanding and processing everyday information

4. Decline in functionality

Work or study performance declined significantly

Unable to complete daily chores

Economic management ability declined

Interpersonal relationships deteriorated significantly

Having difficulty maintaining normal social life

5. Physical symptoms

Pain of unknown origin

conk

Abnormal changes in appetite or weight

Increased physical discomfort

Physical symptoms caused by mental stress

Suggested observation and treatment methods

Observation Strategy

Be patient and empathetic

Systematically record observed anomalies

Avoid criticism or blame

Maintain an open and supportive communication attitude

Specific action recommendations

Create symptom tracking records

Maintain frequent but non-intrusive communication

Encourage continued treatment and medication

Respect your family's privacy and feelings

Provide emotional support

Professional help channels

Contact your family doctor

Arrange psychiatric professional evaluation

Seeking psychological counseling

Join a family support group

Learn about local mental health resources

Important reminder

✓ Early detection is key ✓ Don't bear all the pressure alone ✓ Professional help is crucial ✓ Stay hopeful and patient

Warning of bad practices

Don't hide or deny the problem

Avoid coercion or control

Do not stop taking the medicine without consulting a doctor

Do not isolate patients

Don't blame or shame

Psychological adjustment suggestions

Take care of yourself

remain optimistic

Seek professional support

Set up support network

Get professional guidance

Please note: Each person's situation is unique. Please handle it carefully and respect professional advice.

To help your family track symptoms more systematically, I recommend using a simple but effective symptom tracking form. This will help you record changes and communicate with your doctor.

Mental Illness Symptom Tracker

| date | emotional state | behavior change | sleep quality | doings | Other observations | remarks |
| --- | --- | --- | --- | --- | --- | --- |
| 2025-11-07 |  |  |  |  |  |  |
| 2025-11-08 |  |  |  |  |  |  |
| 2025-11-09 |  |  |  |  |  |  |

filling explanation

Record once a day

Use objective descriptions

Record specific details

Maintain continuity

Don't make subjective judgments

Focus areas

Emotion fluctuation level

Daily function changes

Treatment compliance

Social interaction

On this topic, I want to highlight a few key points:

It's a process that requires a great deal of patience and compassion

Recurrence does not mean treatment failure, but requires adjustment of the treatment plan

Family support is crucial for recovery

Professional help is key

If you need more detailed advice or have specific situations to discuss, I'm always ready to help. Remember, everyone's situation is unique, and the most important thing is to stay hopeful and have professional support.

**Q37.What should you do if family members have hallucinations?**

When a family member has hallucinations, it is a situation that requires caution, compassion, and professionalism. Here are some specific suggestions:

Stay calm and supportive

Don't show fear or shock

Show your family your love and support

Let them know you're willing to help them

Listen and understand

Listen carefully to your family's description of the experience

Don't question or deny their feelings

Don't argue with hallucinations

Show empathy: "I hear you're going through something tough, and I'm here to support you"

Professional Medical Help

Contact a mental health professional as soon as possible

A psychiatric evaluation is recommended

Accompany family members to medical treatment and provide emotional support

If your family refuses to seek medical care, seek advice from a psychologist or your family doctor

security consideration

Pay close attention to your family's emotions and behavior

If the hallucinations involve self-harm or harm to others, seek emergency help immediately

Contact the crisis hotline if necessary

Record symptoms

Record the frequency, time, and content of hallucinations

This information can help doctors diagnose and develop treatment plans

Provide emotional support

Don't make auditory hallucinations a topic of shame

Be patient and compassionate

Make your family feel accepted and understood

Self-Care

Caregivers also need to pay attention to their own mental health

Seek support groups or counseling

Stay emotionally balanced

Important:

Hallucinations can be a symptom of many diseases

Only a professional doctor can make an accurate diagnosis

Early intervention and treatment are critical

Remember, everyone's situation is unique. Professional medical evaluation and personalized treatment plan are the most effective way to deal with hallucinations. Keeping love, patience and support for family members will help them better cope with this challenge.

**Q38.What should we do if people with mental illness commit suicide?**

This is a very serious and important topic. I will provide you with detailed, professional and specific crisis intervention guidelines.

Five steps to crisis intervention:

Ensure safety

Remove all potentially dangerous items (sharp objects, medications, ropes, etc.)

Don't leave the patient alone

Stay calm and build trust

Professional help channels

24-hour psychological assistance hotline: 400-161-9995

Beijing Psychological Assistance hotline: 010-82951332

120 emergency number (if there is actual self-harm)

Local Mental Health Center

Communication Principles

Listen patiently without judgment

Express care: "I'm worried about you"

Ask directly: "Do you really want to end your life?"

Affirm the value of life

Medical intervention

Admitted to a psychiatric hospital

Get a professional psychological assessment

Hospitalization is required when necessary

Follow-up

Continuous psychotherapy

Medication adjustments

Rebuilding the family support system

Important reminder: Crisis can be intervened, life can be saved, professional help is crucial

It should be emphasized that any suicidal thought should be taken seriously. Do not try to "solve" it alone, but seek professional help immediately. Every life is precious and deserves to be respected and loved.

**Q39. What should we do if a patient with mental illness shows violent tendencies?**

I will provide professional, comprehensive and compassionate guidance. This is a sensitive topic that requires careful handling.

Professional response guide to violence in mental illness

I. Safety prevention and assessment

1. Safety first

Stay calm and don't show fear or hostility

Keep yourself and others safe by keeping a safe distance when necessary

Do not attempt to restrain the patient alone

2. Early detection of warning signs

Emotional swings

The language becomes aggressive or threatening

Body language becomes aggressive

Significant anxiety and unease

Refusal to communicate or overdefensive

2. Immediate Response Strategies

1. Communication Principles

Speak in a gentle, calm tone

Use open, non-threatening body language

Maintain proper physical distance

Use statements that begin with "I", such as "I noticed you seemed uneasy"

Listen and show understanding

2. Emergency handling

Call professional medical assistance (120)

Contact your attending physician

Contact your local mental health agency if necessary

If there is any violent behavior, you can ask the public security for assistance

III. Pathways of professional intervention

1. Medical interventions

Adjust the drug regimen in time

Short-term hospitalization may be required

Assess whether mandatory medical care is needed

2. Psychological intervention

psychological consultation

cognitive behavioral therapy

domectic treatment

Emotion Management Training

IV. LONG-TERM MANAGEMENT RECOMMENDATIONS

1. Family support

Be patient and compassionate

Learn professional communication skills

Establish a stable living environment

Encourage regular routines and treatment

2. Social support

Seek professional support groups

Learn about patient rights

Reducing social discrimination

V. LEGAL PROTECTION

1. Patient rights protection

Respect the dignity of patients

Comply with the relevant provisions of the Mental Health Act

Initiate compulsory medical procedures when necessary

2. Risk assessment

Conduct regular professional psychological assessment

Develop a personalized crisis intervention plan

VI. Important Notes

:warning: Remember:

A tendency to violence does not mean that the patient is a "bad person"

This is a disease symptom that requires professional, compassionate treatment

Timely and professional intervention is key

:heart: Hope and Support Every life deserves respect and care. Professional assistance can help patients regain a healthy life.

The guide covers professional strategies for dealing with violent tendencies in people with mental illness. The key is to stay calm, professional and compassionate, always put safety first and seek professional medical help in time.

A few key suggestions:

Don't face it alone. Seek professional help

Stay calm and compassionate

Focus on safety, both yourself and your patients

Seek medical and psychological support

If you are facing this situation, we suggest:

Contact your attending physician immediately

Call 120 for emergency medical assistance

Contact your local mental health agency or public security authorities if necessary

It is a process that requires professionalism, patience and compassion. Every life is worthy of respect and care.

**Q40.How to persuade patients with mental illness to refuse to see a doctor?**

I have prepared a detailed strategy guide on how to persuade people with mental illness to seek medical care. This is a process that requires great patience, compassion, and professionalism. Here is a specific communication guide:

Guidelines for persuading people with mental illness to seek medical care

Core principles

Build trust and empathy

Eliminate the threat of medical treatment

Stimulate intrinsic motivation

Provide security support

Detailed communication strategy

Phase 1: Build trust

communication skill

Choose a time when the patient is relatively calm

Use non-judgmental language

Show genuine care

Specific display examples

"I've noticed you seem a little different lately"

"I care about how you're feeling and want to talk to you"

Let's find a solution together, okay?

Stage 2: Eliminate fear of medical care

Common fear sources

Negative stereotypes about psychiatry

Worried about being labeled

Fear of treatment

coping strategy

Compare medical care to a "mental health checkup"

Emphasize that doctors are professional helpers, not judges

Provide specific and moderate medical pathways

Phase 3: Communication Details

Forbidden terms

You need to see a doctor

"You're sick"

"You're not normal"

Recommended phrases

Let's find out why we feel so uncomfortable

"Your doctor can help us understand your feelings better"

Caring for your mental health is just as important as caring for your body

Phase 4: Action

Provide specific medical assistance

Help with reservations

Accompanying the patient

Learn about health insurance details

Crisis response

Prepare emergency contact information

Learn about local psychological crisis intervention resources

Stay calm and supportive

matters need attention

Respect the patient's feelings

Be patient

Guide, not force

Keep showing care

Important reminder

Everyone's situation is different

Tailor to individual needs

Seek professional psychological counseling advice when necessary

This guide provides a systematic, humanized approach to helping persuade people with mental illness to seek medical care. The key is to use compassion and professionalism to help patients feel respected and understood, not judged.

supplementary specification ：

This process may take several attempts. Don't give up easily

Everyone's situation is unique and requires flexible adjustments

If you feel overwhelmed, seek help from a professional counselor or mental health worker

**Q41. Should family members with mental illness be allowed to manage their money independently?**

This is a very complex and sensitive issue that requires careful and comprehensive analysis. I have outlined several dimensions of this issue previously, and I will now elaborate on the key considerations in more detail.

The core principle is to find a balance between protection and respect:

Assessment of Individual Differences Each psychiatric patient's situation is unique. The assessment should include:

Specific disease type

Severity of symptoms

Cognitive function status

Stability of drug therapy

Personal financial management history

The hierarchical management strategy can adopt a progressive management approach:

a) Fully independent management (applicable):

Symptoms stable

Long-term sustained treatment

Clear financial management skills

No obvious risk behavior

b) Partial supervision and management:

Set monthly spending limit

JA

Transparency of fund flows

Regular financial review

c) Joint management:

Trust family members to collaborate

Establish a clear financial decision-making process

Respect patients' right to participate

Risk prevention measures

Develop emergency plans

Set up financial alerts

Initiate legal guardianship proceedings when necessary

Professional advice path

Psychiatrist assessment

Psychological cognition test

Legal advisory services

Financial planning expert opinion

Key ethical principles

Respect for human dignity

Maximize autonomy

Provide necessary protection

Prevent economic exploitation

Conclusion recommendations: ✅ No one-size-fits-all standard ✅ Personalized assessment is critical ✅ Dynamic adjustment of management plan ✅ Continuous communication and trust

In practice, the following suggestions are recommended:

Get a professional and comprehensive assessment

Communicate fully with patients

Build trust step by step

Keep an open and flexible attitude

There is no absolute "yes" or "no" to this question. The key is to find a balance between protection and respect. Each family needs to make the most suitable plan according to their specific situation.

**Q42. What is the fastest way to find a person with a mental illness who has gone missing?**

I understand this is a very urgent and sensitive situation. For the missing of mental illness patients, I will elaborate on the fastest and most effective way to find them:

Call emergency services immediately (most critical step)

Call 110

Provide patient details:

surname and personal name

age

macroscopic features

Last seen

Mental illness type

Possible walking directions

Quick information sharing

Post a missing person notice on social media

WeChat Moments

microblog

trill

Local search group

Take a clear photo

Include police contact information

Collaborative Search and Rescue

Contact the patient's family/guardian

Notify the last medical facility the patient left

Call on residents in the surrounding community to assist in the search

Contact a professional rescue team (such as Blue Sky Rescue Team)

Tech-assisted location

Cooperate with police to retrieve surveillance footage

Locate the patient's phone (if possible)

Track using SkyNet

special remarks

Emphasize possible abnormal behavior of the patient

Describe possible hazards

Provide information about the risks of discontinuing the medication

Core principles:

Calling the police is the first and fastest way

The golden 24 hours are racing against time

Professional strength + social collaboration

The most important thing is to stay calm, act quickly, and use a combination of resources to ensure the safety of the patient to the maximum extent.

**Q43. As a family member of a mental illness patient, how to relieve your own stress?**

As family members of people with mental illness, it's important to reduce stress. Here are some specific, practical suggestions:

Stress reduction guidelines for family members of people with mental illness

Understanding and acceptance

1. Recognizing stress as normal

It's perfectly normal to feel stressed taking care of your family

Don't be hard on yourself. Stress is understandable and manageable

Understanding your emotions is effective and reasonable

2. Emotion Management

Keep an open mind and allow yourself to experience all kinds of emotions

Learn mindfulness meditation and deep breathing techniques

Keep a diary regularly to organize your inner feelings

II. Self-care

1. Physical health

Keep a regular schedule

Get moderate exercise (at least 150 minutes of moderate-intensity exercise per week)

Pay attention to a balanced diet and get enough sleep

2. Mental health

Schedule personal time to relax each week

Cultivate personal interests

Learn stress management techniques

III. Seeking Support

1. Social support

Join a family support group

Talk to trusted friends

Don't take all the pressure alone

2. Professional help

Consider seeking psychological counseling

Attend professional counseling sessions for family members

Seek family therapy if necessary

4. Set boundaries

1. Role boundaries

Define your scope of care responsibilities

Learn to say no and don't overgive

Keep personal life and space

2. Emotional boundaries

Do not equate the patient's condition with your own value

Distinguish between care and excessive tolerance

Keep your personal emotions separate

V. Use of resources

1. Social resources

Learn about local mental health services

Apply for relevant subsidies and assistance

Find professional caregiver training courses

2. Access to information

Learn about diseases

Stay updated with professional medical information

Attend relevant lectures and workshops

6. Keep Hope

1. Positive mindset

Believe in the possibility of treatment and recovery

Focus on patient progress, not just challenges

Stay optimistic and patient

2. Long-term planning

Develop a long-term family care plan

Flexibility and adaptability

Keep learning and growing

Tips: Take care of yourself so you can take care of your family.

This guide is designed to help family members of people with mental illness systematically manage stress. The key is to remember that self-care is equally important. Each family's situation is unique, so it's recommended to adapt flexibly to your actual circumstances. If you feel persistent and overwhelming stress, it's crucial to seek professional mental health support promptly.

It is important to emphasize that as a family member, your dedication and care are invaluable. It is important to be patient, compassionate, and kind to yourself over the long term.

**Q44. If children show signs of 'reduced speech, irritability, and aversion to learning', could this indicate psychological or mental health issues?**

For children who are "uncommunicative, irritable, and averse to learning", this may indeed be a potential signal of mental and psychological problems. Let me explain in detail:

Symptom Analysis

Not talking much: may indicate social withdrawal, self-isolation, or communication difficulties

Irritability: shows difficulty in regulating emotions, which may be an external manifestation of inner stress or emotional problems

School aversion: This may indicate a loss of motivation, excessive stress, or mental health issues.

Possible mental health issues

Depression: Children's depression may manifest as withdrawal and emotional fluctuations

Anxiety: can lead to social withdrawal and a decline in interest in learning

Adaptive disorder: Poor response to environmental changes or stress

Attention Deficit Hyperactivity Disorder (ADHD): Impacts Learning and Emotional Regulation

Autism spectrum disorder: Social and communication difficulties

Important recommendations

Don't label or diagnose children easily

A comprehensive assessment is critical:

Observe children's daily behavior

Understanding family and school environment

Communicate with children and understand their inner feelings

Consult a mental health professional if necessary

Professional advice

Be patient and caring

Create a safe and open communication environment

Seek help from schools and mental health professionals

Focus on the overall development of the child, not a single symptom

Important reminder: These symptoms don't necessarily indicate serious psychological issues, but persistent or severe symptoms require professional attention. Every child is unique and needs personalized understanding and support.

In short, these symptoms may indeed be a sign of psychological problems, but it is not possible to jump to conclusions. Timely, professional and loving attention is the most important.

**Q45. Can pregnant women or those who are trying to conceive take psychiatric drugs?**

I will create a document to answer this important medical question in detail.

Guidelines for the use of psychiatric drugs during pregnancy and pre-pregnancy

Core principles

The use of psychiatric drugs during pregnancy and pre-pregnancy is a complex medical issue and must follow the following key principles:

Do not discontinue or adjust medication without authorization

A comprehensive evaluation must be made under the guidance of a professional doctor

Personalized decision making is critical

Risk assessment framework

Potential risks of not treating

Untreated mental illness can have serious consequences for both mother and child:

Depression can lead to malnutrition

Anxiety can cause chronic stress

Severe mental disorders may impair a mother's ability to care for herself and her fetus

Possible risks of medication

Some psychiatric drugs may be:

Fetal malformation risk

Developmental abnormalities risk

Risk of neonatal adaptation disorder

Specific drug category recommendations

antidepressant

SSRI drugs (e.g., fluoxetine):

Relatively safe, but still requires individual assessment

Use with caution during late pregnancy and monitor newborns

antianxietic

The risk of short-term use of benzodiazepine medications is relatively low.

Long-term use requires strict evaluation

mood stabilizer

Lithium salt: high risk, usually not recommended

Valproic acid: higher teratogenic risk

Carbamazepine: requires detailed evaluation

During pregnancy

Talk to a psychiatrist 3-6 months in advance

Conduct a comprehensive medication risk assessment

The medication regimen may need to be adjusted

Consider alternative therapies (psychotherapy, etc.)

Key recommendations

Never stop taking the medicine on your own

Consult a psychiatrist and an OB/GYN

Periodic monitoring and evaluation

Establish multidisciplinary medical teams

epilogue

Every pregnant woman's situation is unique. Medication needs to be weighed carefully and a personalized plan developed by a professional doctor.

Important: This guide is for reference only and does not replace professional medical advice.

This guide provides a comprehensive and systematic overview of the complexities of psychiatric medication use during pregnancy and preconception. It emphasizes the importance of individualized, professional medical guidance and the need for a comprehensive risk assessment before any medication decision is made.

I created a document-style guide to make the information more structured and easier to understand. I recommend the following to users:

Read carefully

Discuss your specific situation with your professional doctor

Do not discontinue or adjust medication without authorization

**Q46. Can psychiatric drugs be taken with other drugs?**

I will provide a detailed explanation of this important question about the simultaneous use of psychiatric drugs and other drugs. I will create a detailed document to fully elaborate on this topic.

Guidelines for drug interactions of psychotropic drugs with other drugs

1. Basic concepts of drug interactions

Drug interactions are the possible effects of two or more drugs used together. This is especially important for psychiatric drugs, which usually act on the brain's chemical balance, and interactions can have serious consequences.

2. Main types of interactions

2.1 Pharmacokinetic interactions

Absorption phase: the drug may interact with each other in the absorption process

Distribution phase: change the distribution of the drug in the body

Metabolic phase: affects the metabolic process of the drug in the liver

Excretion phase: changes the rate at which drugs are excreted from the body

2.2 Pharmacodynamic interactions

Enhance or reduce the therapeutic effect of a drug

Cause additional side effects

Change the original therapeutic goal of the drug

3. Common psychiatric drug types and their potential interactions

3.1 Antidepressants

Selective serotonin reuptake inhibitors (SSRIs)

May interact with painkillers and anticoagulants

Use with caution with certain antibiotics

3.2 Anti-anxiety drugs

Benzodiazepines

Severe alcohol interaction

Other drugs that may affect the central nervous system

3.3 Antipsychotics

Medications that may affect the heart

Some antihistamines may have serious side effects

4. Safety Usage Tips

Do not adjust medication without authorization

Tell your doctor all medications you are taking

Get regular drug tests

Understand and identify potential adverse reactions

5. Special warning

⚠ Important reminder:

Everyone's in a different physical condition

Only professional medical personnel can accurately determine drug interactions

Different age groups and health conditions may have different reactions

6. Suggested process

Comprehensive physical examination before first use of psychiatric drugs

Give your doctor a detailed account of your medical history and medication

Follow your doctor's advice for regular checkups

Seek medical attention immediately if any abnormalities occur

7. Situations in which you should not stop taking the medication on your own

Stopping abruptly may cause withdrawal

The medication should be adjusted gradually under the guidance of a doctor

epilogue

The use of psychotropic drugs is a process requiring great caution and professional guidance. Individualized treatment is key.

Disclaimer: This guide is for reference only and does not replace professional medical advice.

This comprehensive guide thoroughly addresses the complexities of concurrent psychiatric medication use. It is important to note that while the article provides general guidance, individual circumstances vary significantly.

Key recommendations:

Be sure to keep in close contact with your doctor

Be truthful about all medications you are taking

Do not adjust medication without permission

Seek medical attention immediately if any abnormal reaction occurs

**Q47. Can I drink alcohol, coffee or strong tea while taking psychiatric drugs?**

Very good question. I will systematically analyze the effects of alcohol, coffee and strong tea on psychiatric medication.

Important precautions for alcohol and caffeine intake during psychiatric medication:

Alcohol risk

Alcohol significantly interferes with the metabolism and efficacy of psychiatric drugs

Possible dangers include: • Reduced drug action • Central nervous system depression • Abnormal drug metabolism • In severe cases, it may cause life-threatening danger

Caffeine Effects

Caffeine, as a central nervous system stimulant, may: • worsen anxiety symptoms • affect drug metabolism • cause side effects such as palpitations and insomnia

Specific risk

Antidepressants: Alcohol may reduce effectiveness

Anti-anxiety medications: Alcohol and caffeine may worsen side effects

Antipsychotics: Metabolism may be interfered with

Recommendation principles

Alcohol should be avoided during medication

The intake of coffee and strong tea should be strictly controlled

The most important thing is to follow the doctor's advice

Personalized suggestions

Each drug responds differently to alcohol and caffeine

Consult your doctor for specific conditions

Your doctor will give you professional advice based on your specific medication

In summary, for medication safety and treatment effectiveness, it is strongly recommended to: ✖ Avoid alcohol ✔ Be cautious with caffeine intake ❗ Always follow your doctor's instructions

**Q48. What are the dietary restrictions for patients with mental illness during medication?**

I have prepared a comprehensive answer for you, summarizing the dietary restrictions for patients with mental illness during medication. This summary covers the dietary precautions for different types of mental illness drugs, so as to help patients better understand the dietary issues they should pay attention to during medication.

Dietary restrictions for patients with mental illness during medication

1. Dietary restrictions for monoamine oxidase inhibitors (MAOIs)

Strictly prohibited foods

Aged cheeses (Cheddar, blue cheese)

Sausage, cured meats, and fermented meats (sausages, cured meats, salami)

Fermented soy products (miso, fermented tofu, fermented soybean)

Yeast extract (hemp extract)

Specific alcoholic beverages (red wine, beer, spirits)

Overripe fruit (banana, avocado, fig)

Specific legumes (peas, lentils)

Not fresh meat or fish

matters need attention

Avoid caffeinated drinks

Prevent tyramine accumulation and cause hypertensive crisis

2. Dietary precautions for lithium carbonate

main limitation

Limit sodium intake

Avoid drinking large amounts of water after excessive sweating

No alcohol

Be careful with caffeine

3. Tricyclic Antidepressants (TCAs)

dietetic contraindication

Alcohol is strictly prohibited

Avoid grapefruit juice

4. Selective Serotonin Reuptake Inhibitors (SSRIs) and Serotonin-Norepinephrine Reuptake Inhibitors (SNRIs)

matters need attention

Avoid alcohol

Limit grapefruit juice

5. Antipsychotic drugs

Dietary recommendations

Avoid alcohol

Control caffeine intake

Eat a low-fat, low-sugar diet

Prevent metabolic syndrome

6. Anti-anxiety drugs

Important Contraindications

Alcohol is strictly prohibited

7. Other important considerations

Lifestyle advice

Quit smoking

Stay hydrated

Balanced nutrition

Important reminder

Significant individual differences

Follow the personalized advice of your doctor or pharmacist

Communicate regularly with the medical team

Strongly recommended: Each patient should consult a professional medical staff based on their own medication situation to obtain the most suitable dietary guidance.

This comprehensive dietary guide outlines food restrictions for individuals with mental health conditions during medication. It's crucial to recognize that each person's situation is unique, making it essential to follow personalized recommendations from your doctor or pharmacist.

I want to emphasize that while this guide provides general guidance, it does not completely replace professional medical advice. Different mental illnesses, different medications, and even different specific varieties of the same drug may have subtle differences. Therefore, patients should:

Read the drug instructions carefully

Talk to your doctor

Consult a healthcare professional if you have any dietary questions

Do you have any questions about certain parts of the document? I'd be happy to explain further.

**LLM Model: DeepSeek-R1**

**Q1. What is mental illness?**

Mental illness refers to psychological or behavioral disorders that impair a person's thinking, emotions, behavior, or social interactions. These conditions often cause significant difficulties in daily life, work, or social settings. The spectrum of mental illnesses is extensive, ranging from common anxiety and depression to more complex conditions like schizophrenia and bipolar disorder.

The main characteristics of mental illness:

1. Affects thinking and emotions: may lead to persistent sadness, anxiety, confusion or hallucinations.

2. Behavioral changes: such as social withdrawal, impulsive behavior, or inability to complete daily tasks.

3. Physiological factors: may be related to brain chemistry imbalance, genetics or traumatic experience.

4. Social factors: Stress, environmental or interpersonal problems may also trigger or aggravate symptoms.

Common types of mental illness:

Depression: Persistent low mood, loss of interest or pleasure.

Anxiety disorder: excessive worry, fear, or panic attacks.

Schizophrenia: hallucinations, delusions and disorganized thinking.

Bipolar disorder: mood swings between extreme highs (mania) and extreme lows (depression).

Obsessive-Compulsive Disorder (OCD): Recurrent, uncontrollable thoughts or behaviors.

Post-traumatic stress disorder (PTSD): A severe emotional response triggered by traumatic events.

Treatment and Support:

Mental illness can usually be managed through psychotherapy, medication, or a combination of both. Social support, a healthy lifestyle, and early intervention are also important.

If you or someone you know may be experiencing mental health problems, seek professional help, such as a counselor or psychiatrist. Mental illness is treatable, and timely intervention can significantly improve quality of life.

**Q2. What are the common types of mental illness?**

There are many kinds of mental illness, and the common types include the following:

1. Anxiety Disorders

Generalized anxiety disorder (GAD): persistent and excessive worry.

Panic Disorder: Sudden, intense fear or discomfort.

Social Anxiety Disorder (SAD): An excessive fear of social situations.

Obsessive-Compulsive Disorder (OCD): Recurrent, uncontrollable thoughts or behaviors.

Post-traumatic stress disorder (PTSD): persistent fear and flashbacks following a traumatic event.

2. Mood Disorders

Major Depressive Disorder (MDD): Characterized by persistent low mood and loss of interest.

Bipolar Disorder: Characterized by mood swings between depressive and manic states.

3. Schizophrenia Spectrum Disorders

Schizophrenia: characterized by hallucinations, delusions, and disorganized thinking.

Delusional Disorder: Persistent delusions that are not grounded in reality.

4. Personality Disorders

Borderline Personality Disorder (BPD): marked by emotional instability and interpersonal difficulties.

Antisocial Personality Disorder (ASPD): marked by a lack of empathy and impulsive behavior.

Obsessive-Compulsive Personality Disorder (OCPD): characterized by perfectionism and a strong need for control.

5. Eating Disorders

Anorexia Nervosa: Extreme control over body weight and food intake.

Bulimia Nervosa: This eating disorder involves compensatory behaviors such as vomiting or excessive exercise after binge eating.

6. Substance-Related Disorders

Dependence or abuse of alcohol, drugs and other substances.

7. Neurodevelopmental Disorders

Attention Deficit Hyperactivity Disorder (ADHD): characterized by inattention and hyperactivity.

Autism spectrum disorder (ASD): Social and communication difficulties.

8. Dissociative Disorders

Dissociative Identity Disorder (DID): Multiple personalities.

Dissociative Amnesia: The loss of memory for significant events.

9. Sleep Disorders

Insomnia: Difficulty falling asleep or staying asleep.

Narcolepsy: Excessive daytime sleepiness.

10. Sexual Dysfunctions

Such as decreased libido, erectile dysfunction, etc.

The symptoms and severity of these diseases vary from person to person and usually require evaluation and treatment by a professional psychologist or psychiatrist. If you or someone close to you has symptoms, seek help promptly.

**Q3. Is anxiety a mental illness?**

Yes, anxiety disorder is a type of mental illness. It is classified as a mental disorder, specifically an emotional disorder. The main characteristics of anxiety disorder include excessive and persistent worry or fear, which may interfere with daily life, work, or interpersonal relationships.

Common anxiety disorders include:

1. Generalized Anxiety Disorder (GAD): Excessive worry about daily life.

2. Panic Disorder: Recurrent panic attacks.

3. Social Anxiety Disorder: Intense fear of social situations.

4. Specific Phobias: Extreme fear of specific objects or situations.

5. Separation Anxiety Disorder: Excessive anxiety about separation from a loved one.

Anxiety disorders can typically be effectively managed through psychotherapy (such as cognitive behavioral therapy), medication (like antidepressants or anti-anxiety drugs), or a combination of both. If you or someone you know is experiencing anxiety symptoms, it's advisable to seek help from a professional psychologist or psychiatrist.

**Q4. Is depression a mental illness?**

Yes, depression is a mental illness. It is a common mood disorder characterized by persistent low mood, loss of interest or pleasure, reduced energy, and changes in sleep and appetite. Depression not only affects a person's mood and mental state, but may also severely impact daily life, work, and relationships.

Depression has complex causes, which may involve genetic, biochemical, environmental, psychological and social factors. If you suspect that you or others are suffering from depression, it is recommended to seek the help of a professional psychologist or psychiatrist in time, so as to obtain an accurate diagnosis and appropriate treatment.

**Q5. What are the main causes of mental illness?**

The causes of mental diseases are usually multifactorial, involving the interaction of biological, psychological, social and other levels. The following are the main pathogenic factors:

1. Biological factors

Genetic factors: Some mental diseases (such as schizophrenia, bipolar disorder, depression) have obvious family aggregation, which may be related to genetic variation.

Neurochemical imbalance: Abnormal neurotransmitters (such as dopamine, serotonin, norepinephrine) in the brain may lead to mood or behavioral disorders.

Brain structure or function abnormalities: for example, structural changes may occur in certain areas of the brain (such as the hippocampus and prefrontal cortex) in patients with schizophrenia.

Infection or trauma: such as encephalitis, head trauma may cause mental symptoms.

2. Psychological factors

Personality traits: Certain personality types (such as high neuroticism or perfectionism) may increase the risk of depression or anxiety.

Childhood trauma: such as abuse, neglect, domestic violence, etc., may lead to psychological problems in adulthood (such as post-traumatic stress disorder, borderline personality disorder).

Poor coping skills: Lack of effective stress management skills can lead to anxiety or depression.

3. Social and environmental factors

Life stress: Major life events such as unemployment, divorce, and the death of a loved one can trigger mental illness.

Lack of social support: The lack of support from family and friends or isolation can exacerbate psychological problems.

Cultural and social environment: macro factors such as discrimination, poverty, war and so on may also affect mental health.

4. Other factors

Substance abuse: Alcohol, drugs and other substances may directly lead to mental disorders (such as substance-induced mental illness).

Chronic diseases: such as diabetes, cardiovascular diseases and so on may indirectly cause depression or anxiety.

sum up

Mental illness is often the result of multiple factors, not a single cause. For example, a person may have a genetic predisposition, but the disease may not develop until after a stressful event. Therefore, treatment and prevention also need to take these factors into account.

**Q6. Does insomnia or staying up late for a long time increase the risk of mental illness?**

Yes, insomnia or chronic sleep deprivation does increase the risk of mental illness. Here are some key scientific evidence and mechanisms:

1. The relationship between sleep and brain function

Emotion regulation: Sleep deprivation affects brain regions responsible for emotion regulation (such as the prefrontal cortex and amygdala), leading to mood instability, irritability or anxiety.

Cognitive function: Chronic sleep deprivation can impair attention, memory and decision-making, all of which are closely linked to mental health problems (such as depression and anxiety).

2. Increased risk of mental illness

Depression: Studies have shown that insomnia or poor sleep quality is one of the important triggers of depression. Long-term sleep deprivation may lead to the imbalance of neurotransmitters such as serotonin, which may lead to depressive symptoms.

Anxiety: Lack of sleep can exacerbate anxiety and even trigger anxiety disorders.

Schizophrenia: Long-term sleep disorders may be involved in the pathogenesis of severe mental disorders such as schizophrenia.

3. Disrupted biological clock

Staying up late for a long time can disrupt the body's circadian rhythm (biological clock) and affect the secretion of melatonin. This disruption can not only lead to sleep problems, but also cause or aggravate mental illness.

4. Inflammation and oxidative stress

Sleep deprivation increases inflammatory responses and oxidative stress in the body, which are associated with the development of a variety of mental disorders (such as bipolar disorder).

5. Research support

Multiple epidemiological studies have shown that people who are chronically sleep-deprived have a significantly higher risk of depression, anxiety and other mental disorders than those who sleep normally.

propose

Regular sleep schedule: Try to keep a regular sleep time and avoid staying up late.

Improve your sleep environment: reduce light and noise interference, and avoid using electronic devices before bed.

Seek help: If you have persistent insomnia or late nights, consult a doctor or mental health specialist.

In short, sleep is crucial for mental health, and chronic sleep deprivation or insomnia does increase the risk of mental illness.

**Q7. How to tell if you may have a mental illness?**

Assessing whether you may have a mental illness is a matter of caution. Here are some common signs and steps to help you make an initial assessment of your mental health:

1. Common signs of mental health problems

Here are some symptoms that may be related to mental illness and may require attention if they persist for a long time (more than two weeks) or significantly affect daily life:

emotion changes ：

Long-term feelings of sadness, anxiety, anger or low mood.

Emotional fluctuations are severe and uncontrollable.

Lose interest in things you used to like.

behavior change ：

Social withdrawal, unwilling to contact people.

Significant changes in sleep or eating habits (such as insomnia or oversleeping).

Difficulty concentrating or completing daily tasks.

Cognitive problems:

Confusion, memory loss or difficulty making decisions.

Unreasonable fears or paranoid thoughts.

Physical symptoms:

Unexplained headaches, stomachaches or other physical discomforts.

Decreased energy and long-term fatigue.

Extreme behaviors or thoughts:

Have thoughts of self-harm or suicide.

Experiencing hallucinations (such as hearing or seeing things that are not there) or delusions (such as believing things that are not true).

2. Steps for Self-Assessment

If you suspect you may have a mental health problem, try the following:

1. Record symptoms:

Write down any emotions, behaviors, or physical changes you notice, along with how long and how often they occur.

2. Use standardized tools:

Some mental health questionnaires (such as the depression self-rating scale and anxiety self-rating scale) can help you make a preliminary assessment. However, please note that these tools cannot replace professional diagnosis.

3. Communicating with others:

Talk to a trusted friend or family member about your feelings. They may provide objective feedback.

4. Observe function impact:

Assess whether these symptoms affect your work, study, or relationships.

3. When to seek professional help?

If you experience any of the following, you should consult a mental health professional (such as a psychologist, psychiatrist, or counselor) as soon as possible:

Symptoms have lasted more than two weeks without improvement.

Symptoms severely affect daily life.

Have thoughts of self-harm or harming others.

4. Notes

Avoid self-diagnosis: Mental illness requires professional evaluation, and self-judgment may not be accurate.

Reduce stigma: Mental illness is just as much a treatment as physical illness, and there is no shame or guilt involved.

Early intervention: The earlier you seek help, the more likely you are to recover.

If you have any doubts, it is recommended that you make an appointment with a professional doctor or psychologist as soon as possible for evaluation. You are not alone. Many people have recovered a healthy life through proper treatment and support.

**Q8. Can the self-assessment form accurately determine whether you have a mental illness?**

Self-administered measures (such as psychological assessment questionnaires) can serve as a preliminary screening tool to help individuals identify potential mental health problems, but they cannot replace professional diagnosis, nor can they be used alone to confirm mental illness. Here are the key reasons:

1. Limitations

Subjectivity: Self-reporting scales rely on individuals' self-reports and may be influenced by subjective biases (such as denial, exaggeration, or misunderstanding of the problem).

Limited scope: Scales are usually targeted at specific symptoms (such as depression, anxiety) and cannot cover the complex manifestations of all mental disorders (such as schizophrenia, personality disorders).

False positives/negatives: There may be misdiagnosis, such as misdiagnosing a brief emotional fluctuation as a disease, or ignoring hidden symptoms.

2. The need for professional diagnosis

Comprehensive assessment: Psychiatrists or clinical psychologists will make a diagnosis based on the results of the scale, interviews, medical history, behavioral observation and even physical examination (such as ruling out thyroid problems that cause depression).

Differential diagnosis: Many mental disorders have overlapping symptoms (such as anxiety and depression) that require expertise to distinguish.

3. Reasonable use of self-measurement forms

Initial screening: Helps individuals recognize the problem and seek professional help.

Track progress: Monitor symptom changes during treatment (must be done with a doctor).

4. Risk Warning

Self-labeling: Over-reliance on scales can lead to unnecessary anxiety or self-stigmatization.

Delayed treatment: Relying on the scale alone and ignoring professional assessment may miss the best time for intervention.

propose

If your self-assessment shows a potential problem or you continue to feel distressed, contact a mental health professional (e.g., psychiatrist, clinical psychologist) as soon as possible. They can provide an authoritative diagnosis and personalized treatment recommendations.

If needed, I can recommend some commonly used self-assessment tools (such as the PHQ-9 depression scale and GAD-7 anxiety scale) for reference, but keep in mind that they are only auxiliary tools.

**Q9. Which department should I visit if I suspect a mental illness?**

If you suspect that you or someone else has a mental illness, you should see a psychiatrist. Psychiatrists are specialists who diagnose and treat mental disorders, mood disorders, behavioral disorders, and other psychological problems.

Other related departments:

1. Psychology Department: For mild cases or when counseling or therapy is needed, the psychology department is the preferred choice. Services are typically provided by clinical psychologists or licensed counselors.

2. Neurology: If psychiatric symptoms are suspected to be linked to brain disorders (such as epilepsy or encephalitis), a neurologist's evaluation may be required.

3. Psychological outpatient clinics in general hospitals: Many general hospitals have psychological outpatient clinics to provide initial assessment and referral services.

Medical advice:

Emergency: If there is self-injury, injury to others, severe hallucinations or delusions, go to the emergency department or call the emergency number immediately.

Children or adolescents: You can choose child psychiatry or developmental and behavioral pediatrics.

If you have specific symptoms or are not sure, you can first consult the triage desk of the general hospital, and they will guide you to the appropriate department according to the situation.

**Q10.How to distinguish mental illness from other physical illness?**

Distinguishing mental illness from other physical (somatic) diseases usually requires a comprehensive consideration of symptoms, etiology, diagnostic methods, and treatment options. Here are some key differences:

1. Symptoms

mental sickness ：

It is mainly manifested as abnormal emotion, thinking, behavior or cognitive function, such as depression, anxiety, hallucination, delusion, inattention, etc.

Symptoms are often related to psychological state or social functioning, such as social withdrawal, emotional fluctuations, etc.

physical illness ：

It is mainly manifested as abnormal physiological functions, such as pain, fever, fatigue, organ dysfunction, etc.

Symptoms can often be detected through medical tests (such as blood tests, imaging tests) to detect objective abnormalities.

2. Causes

mental sickness ：

The etiology is complex and may involve genetic, neurochemical abnormalities (such as dopamine and serotonin imbalance), psychological trauma, environmental stress, etc.

There is usually no single cause, but a combination of factors.

physical illness ：

The causes are usually more specific, such as infection, trauma, metabolic abnormalities, tumors, etc.

Direct evidence can be found in the laboratory or imaging studies.

3. Diagnostic methods

mental sickness ：

The assessment primarily relies on clinical interviews, psychological evaluation scales, and behavioral observations (such as DSM-5 or ICD-11 criteria).

At present, there is a lack of objective biomarkers, and diagnosis is more based on subjective symptoms.

physical illness ：

It depends on laboratory tests (such as complete blood count and biochemical indicators), imaging (such as X-ray and MRI), or pathological biopsy.

Diagnosis is usually based on clear objective evidence.

4. Treatment

mental sickness ：

Treatment includes psychotherapy (such as cognitive behavioral therapy), medication (such as antidepressants, antipsychotics) and social support.

The goal is to improve psychological state and social functioning.

physical illness ：

Treatment includes drugs, surgery, physical therapy and so on, which directly target physiological abnormalities.

The goal is to restore or improve physiological function.

5. Overlap and comorbidity

Some diseases may involve both mental and physical aspects, such as:

Thyroid dysfunction: may lead to mood swings (such as anxiety or depression), but the root cause is endocrine problems.

Chronic pain: May lead to depression or anxiety, but the pain itself is a physical symptom.

This situation requires multidisciplinary collaboration (such as psychiatry and internal medicine) for comprehensive evaluation.

6. Common Misconceptions and Precautions

Don't ignore physical illness: Some mental symptoms can be early signs of physical illness (such as personality changes caused by brain tumors).

Avoid overlabeling: Not all emotional fluctuations or abnormal behaviors are mental disorders, which need to be judged in the specific situation.

If further differentiation is needed, it is recommended to seek the help of a professional doctor, especially a psychiatrist or general practitioner, who can give an accurate diagnosis through a comprehensive assessment.

**Q11. Why do hospitals need multiple tests to diagnose mental diseases?**

The hospital diagnosis of mental illness requires a number of tests, the main reasons include the following:

1. Exclude other diseases

The symptoms of mental disorders (such as low mood, anxiety, hallucinations, etc.) may resemble those of certain physical conditions (e.g., thyroid dysfunction, brain tumors, neurological disorders) or drug side effects. Blood tests and imaging studies (e.g., CT, MRI) can help rule out these physical conditions, ensuring accurate diagnosis.

2. Comprehensive assessment of the patient's physical and psychological status

The diagnosis of mental illness depends not only on the symptoms, but also on the overall health of the patient. For example:

Laboratory tests (such as complete blood count, biochemical indicators) can assess the patient's nutritional status, metabolic function, etc.

Electroencephalography (EEG) detects abnormal brain electrical activity, helping to diagnose epilepsy or certain mental disorders.

3. Clarifying the types and severity of mental illness

Mental disorders are numerous (such as depression, schizophrenia, bipolar disorder, etc.), and symptoms may overlap. Through psychological scale assessment, clinical interview, etc., doctors can more accurately judge the type and severity of the disease, so as to develop a personalized treatment plan.

4. Identification of comorbidity

Many patients may have multiple mental disorders (such as depression combined with anxiety) or mental disorders and physical diseases. Multiple examinations can help to detect these comorbidities and avoid missed or misdiagnoses.

5. Evidence-based care

Different mental illnesses may require different treatments (such as medication, psychotherapy, or physical therapy). Test results can help doctors choose the most appropriate treatment and monitor physical reactions during treatment (such as drug side effects).

6. Legal and ethical requirements

The diagnosis of mental illness may involve legal or insurance issues (such as disability assessment, insurance claims). Detailed examination records can provide an objective basis for these needs.

sum up

The purpose of multiple examinations is to ensure the accuracy, comprehensiveness and scientific nature of diagnosis, avoid misdiagnosis or missed diagnosis caused by a single assessment, and provide the most appropriate treatment plan for patients.

**Q12. Is psychological assessment helpful for the diagnosis of mental illness?**

Psychological assessment can play a certain auxiliary role in the process of diagnosing mental diseases, but it usually can not be used as the only basis for diagnosis. The following are the specific roles and limitations of psychological assessment in the diagnosis of mental diseases:

act on ：

1. Preliminary screening:

Psychological assessment can help identify whether an individual has symptoms or risk factors for certain mental disorders, such as depression, anxiety, and personality disorders.

For example, the Beck Depression Inventory (BDI) or Hamilton Anxiety Rating Scale (HAMA) can quantify the severity of symptoms.

2. Auxiliary diagnosis:

The results of the assessment can provide the doctor with objective data, combined with clinical interviews and other tests, to help form a more comprehensive diagnosis.

For example, the Minnesota Multiphasic Personality Inventory (MMPI) can aid in diagnosing personality disorders and schizophrenia.

3. Monitor disease changes:

Through regular assessment, changes in symptoms can be tracked and treatment or disease progression can be assessed.

4. Standardized assessment:

Psychological assessment tools are usually scientifically validated and provide standardized assessments that reduce subjective bias.

Limited:

1. Cannot diagnose independently:

The diagnosis of mental illness requires a combination of clinical interviews, medical history, behavioral observation, and medical examination (such as the exclusion of organic disease). The assessment results are only part of it.

2. Influence of cultural and social factors:

Some assessment tools may be affected by cultural background or language differences, resulting in biased results.

3. Subjectivity:

The assessment relies on individual self-reporting, and the results may be inaccurate if individuals intentionally conceal or misinterpret the questions.

4. Tool limitations:

Different assessment tools have different sensitivity and specificity, and some may be better suited for screening than for diagnosis.

sum up ：

Psychological assessment serves as a valuable tool for diagnosing mental disorders, but it should be used in conjunction with other clinical information. While it helps doctors identify issues and develop intervention plans more effectively, it cannot replace professional clinical judgment. If you or someone else has concerns about mental health, it is recommended to seek professional evaluation from a psychiatrist or clinical psychologist.

**Q13. What should be done if different psychiatrists have different diagnoses?**

When different psychiatrists have inconsistent diagnoses, the following steps can be taken to deal with the situation:

1. Seek professional advice from a third party

If the two doctors' diagnoses differ significantly, it is advisable to consult a third or even more psychiatrists, especially experienced specialists or those from specialized hospitals. Multiple opinions can help provide a more comprehensive assessment of the issue.

2. Review diagnostic basis

Understand the diagnostic criteria for each physician, including:

The assessment tools used (such as scales, questionnaires, clinical interviews, etc.).

Observed symptoms and behavioral manifestations.

The patient's medical history and family history.

If some doctors use a more comprehensive assessment method, their diagnosis may be more valuable.

3. Consider the complexity of diagnosis

The diagnosis of mental illness is sometimes subjective, especially for diseases with overlapping symptoms (such as depression and bipolar disorder, anxiety and post-traumatic stress disorder). Doctors may give different diagnoses depending on different priorities.

Patients can ask their doctor if there is a possibility of comorbidity (having two or more mental illnesses at the same time).

4. Focus on common ground in treatment plans

Even if the diagnosis is different, the treatment plan may be similar (such as drug therapy, psychotherapy, etc.). You can start with the common treatment method and observe the effect.

5. Record symptom changes

Patients or their families can record the changes of symptoms and their response to treatment in detail, which provides a more objective basis for subsequent diagnosis.

6. Seeking multidisciplinary team assessment

In complex cases, a multidisciplinary team of psychiatrists, psychotherapists, social workers, etc. can be sought to evaluate and synthesize the opinions of all parties.

7. Avoid frequent doctor changes

Frequent doctor changes may lead to a lack of continuity in diagnosis and treatment. It is recommended to stick with a trusted doctor for a period of time for treatment and follow-up.

8. Patient and family involvement

Patients and their families should actively participate in the diagnostic process, provide detailed information, and express questions or concerns about the diagnosis.

9. Consideration of cultural or linguistic factors

If there is a cultural or language barrier between the patient and the doctor, it may affect the diagnosis. You can seek a doctor who provides culturally sensitive services.

10. Stay patient

Diagnosis and treatment of mental illness can take time, and it is important to be patient and follow your doctor's advice.

If diagnostic differences cause serious distress, seek help from an ethics committee or other relevant body to ensure the patient's rights are protected.

**Q14. Will you be considered to have a mental illness if you visit a psychiatrist?**

A psychiatric visit doesn't equate to having a mental illness. Psychiatrists not only treat severe mental disorders (such as schizophrenia and bipolar disorder) but also address common mental health issues like anxiety, depression, insomnia, stress management, and interpersonal difficulties. Here are some key points:

1. The scope of psychiatry is broad:

Psychiatrists help patients with a range of psychological and emotional problems, from mild to severe.

Many people who visit psychiatry may be experiencing temporary emotional distress or need professional guidance.

2. Visit = Illness:

Just as you see a doctor for a cold, it is normal health management behavior to see a psychiatrist for psychological or emotional problems.

Taking the initiative to seek help is a sign of self-care, not a sign of being "sick".

3. Misconceptions about social concepts:

Due to misconceptions about mental health, some people may have a bias against visiting psychiatry, but this is unscientific.

Modern medicine is increasingly emphasizing the importance of mental health, and there is no essential difference between visiting a psychiatrist and visiting other departments.

4. Privacy protection:

Mental health records are usually confidential and not disclosed to others without the patient's consent.

If you or someone else is hesitant to see a doctor for fear of being misunderstood, rest assured: psychiatrists will help in a professional and respectful manner, and will not easily label patients. Mental health is just as important as physical health, and it is wise to seek help in time.

**Q15. What are the main methods of treating mental illness?**

There are many ways to treat mental illness, and usually require a combination of treatment based on the patient's specific condition, cause and personal needs. Here are the main treatments:

1. Psychotherapy

Cognitive Behavioral Therapy (CBT) helps patients identify and change negative thinking and behavior patterns, which is suitable for depression, anxiety, etc.

Psychodynamic therapy: the exploration of the influence of unconscious conflicts and childhood experiences on current behavior, often used for personality disorders or long-term psychological problems.

Interpersonal Therapy (IPT): A therapeutic approach focused on improving interpersonal relationships, effective for treating depression and social anxiety.

Family therapy: to solve problems in the family system and improve the interaction between family members.

Group therapy: Sharing experiences and support in a small group setting, suitable for addiction, post-traumatic stress disorder (PTSD), etc.

2. Pharmacotherapy

Antidepressants: such as SSRIs (selective serotonin reuptake inhibitors) for treating depression and anxiety.

Antipsychotic drugs: used for schizophrenia, bipolar disorder, etc.

Emotion stabilizers: such as lithium, used for bipolar disorder.

Anti-anxiety medications: such as benzodiazepines, which provide short-term relief from anxiety symptoms.

Stimulants: Used for Attention Deficit Hyperactivity Disorder (ADHD).

3. Physiotherapy

Electroconvulsive therapy (ECT): Used for severe depression or schizophrenia when medication is ineffective.

Transcranial magnetic stimulation (TMS): a non-invasive treatment for depression.

Vagus Nerve Stimulation (VNS): Used for treatment-resistant depression.

4. Lifestyle adjustments

Regular exercise: Improve mood and reduce anxiety.

Healthy eating: Foods rich in omega-3 fatty acids may be good for your mental health.

Get enough sleep: Sleep problems are often associated with mental illness.

Reduce alcohol and drugs: Avoid aggravating symptoms.

5. Social support

Community support: such as rehabilitation centers or mutual aid groups.

Occupational therapy: helps patients recover social functioning.

Family support: Family understanding and involvement is critical to recovery.

6. Alternative therapies

Art therapy: Expressing emotions through painting, music, etc.

Meditation and mindfulness: Reduce stress and anxiety.

Acupuncture: Some studies have shown an adjunctive effect on anxiety and depression.

7. Comprehensive treatment

A combination of approaches, such as medication, psychotherapy and lifestyle changes, is usually used to improve the effectiveness.

The effectiveness of each method varies from person to person. It is recommended to develop a personalized treatment plan under the guidance of a professional doctor. If you or others have specific needs, you can further discuss the appropriate treatment plan.

**Q16. Which is more effective, psychiatric drug therapy or psychotherapy?**

Psychiatric medication and psychotherapy each have their own advantages and applications, and which is more effective depends on the patient's specific situation, type of disease, severity, and personal preference. Here are the two comparisons and applications:

1. Psychiatric medication

Applicable to:

Suitable for moderate and severe mental diseases (such as schizophrenia, bipolar disorder, major depression, anxiety, etc.).

When symptoms severely affect daily life or there is a risk of self-harm or harm to others, the drug can quickly relieve symptoms.

superiority ：

Quick onset: Medications (such as antidepressants, antipsychotics) usually relieve symptoms within a few weeks.

For biological factors: Some mental illnesses are associated with a balance of neurotransmitters, and drugs can directly regulate these chemicals.

boundedness ：

Side effects may include weight gain, drowsiness, sexual dysfunction, etc.

Unable to address psychological or social factors (such as trauma, interpersonal problems).

2. Psychotherapy

Applicable to:

Suitable for mild to moderate mental disorders (such as mild depression, anxiety, post-traumatic stress disorder, etc.).

When problems are closely related to psychological or social factors (such as childhood trauma, stress, interpersonal relationships).

superiority ：

Long-term effects: Psychotherapy (such as cognitive behavioral therapy, psychodynamic therapy) can help patients change their thinking and behavior patterns and reduce the risk of relapse.

No side effects: suitable for patients who are sensitive to drugs or do not want to take drugs.

boundedness ：

It takes time to take effect. It may take weeks or months.

The effect is limited in severe symptoms (such as hallucinations, delusions).

3. Which is more effective?

Comprehensive treatment: For many diseases (such as depression, anxiety), a combination of medication and psychotherapy is often more effective than a single treatment. For example:

The drug quickly alleviates symptoms, and psychotherapy helps patients cope with the underlying problem.

Personalized selection:

Patient preference, disease type and severity are key.

For example, schizophrenia is usually treated primarily with medication, while mild anxiety may only require psychotherapy.

4. Research evidence

Depression: Studies have shown that medication and psychotherapy (such as cognitive behavioral therapy) are equally effective, but combined treatment is better.

Anxiety: Psychotherapy (such as exposure therapy) is better for long-term results.

Schizophrenia: drugs are the basis, and psychotherapy is used to improve function.

sum up

There is no absolute "more effective", but the choice or combination of the two should be based on the specific situation of the patient. It is recommended to develop a personalized treatment plan under the guidance of a professional doctor.

**Q17.Is exercise effective against mental illness?**

Exercise does have a positive effect on mental illness and has been shown in many studies to be an effective adjunct to treatment. Here are the main benefits of exercise for mental health:

1. Alleviating depression and anxiety

Science: Exercise promotes the release of endorphins (a natural "happy hormone") in the brain, while increasing levels of serotonin and dopamine, neurotransmitters that are closely associated with mood regulation.

Research support: Several studies have shown that regular aerobic exercise (e.g., running, swimming) and resistance training (e.g., weight lifting) can significantly reduce symptoms of depression and anxiety, and even be as effective as medication.

2. Improve cognitive function

Target population: Exercise can help improve attention, memory and executive function in patients with schizophrenia, bipolar disorder, etc.

Mechanism: Exercise stimulates the secretion of brain-derived neurotrophic factor (BDNF), which promotes neuronal growth and connectivity.

3. Reduce stress

Benefits: Exercise can lower cortisol (stress hormone) levels and help the body and brain recover from stress.

Recommended activities: Yoga, tai chi and other low-intensity exercise are especially suitable for relieving stress.

4. Improve social skills

Group sports: Participating in group sports (such as basketball, dance) can improve social isolation and enhance a sense of belonging, especially for people with autism spectrum disorder or social phobia.

5. Improve sleep

Relevance: Many people with mental illness have sleep disorders, and regular exercise can regulate circadian rhythms and improve sleep quality.

matters need attention

Individual differences: The effect of exercise varies from person to person, so it is necessary to choose the right type and intensity of exercise according to individual health status and interest.

Adjuvant therapy: Exercise cannot completely replace medication or psychotherapy, but can be part of a comprehensive treatment.

Progressive: For patients who lack exercise for a long time or have a serious condition, it is recommended to start with low intensity and gradually increase.

sum up

Exercise offers significant benefits for managing and rehabilitating mental illness as a low-cost, low-risk, and easy-to-implement intervention. If you or someone you know is experiencing mental health issues, try incorporating exercise into your daily routine and developing a personalized plan under the guidance of a professional doctor.

**Q18. What kinds of drugs are commonly used to treat mental illness?**

There are many kinds of drugs for mental illness, and the appropriate drugs are usually selected according to the type and symptoms of the disease. The following are the commonly used categories of drugs for mental illness and their main uses:

1. Antipsychotics

Use: Mainly used to treat schizophrenia, manic episodes of bipolar disorder, and other psychotic symptoms (such as hallucinations, delusions).

classify ：

First-generation antipsychotics (typical antipsychotics) such as chlorpromazine and haloperidol primarily block dopamine D2 receptors, though they are associated with numerous side effects, including extrapyramidal reactions.

The second generation (atypical antipsychotics) includes risperidone, olanzapine, and quetiapine. These medications have broader therapeutic effects and fewer side effects.

2. Antidepressants

Use: depression, anxiety, obsessive-compulsive disorder, etc

classify ：

Selective serotonin reuptake inhibitors (SSRIs), such as fluoxetine, sertraline, and paroxetine, are commonly used as first-line medications due to their fewer side effects.

Serotonin and norepinephrine reuptake inhibitors (SNRIs), such as venlafaxine and duloxetine, are effective in treating treatment-resistant depression.

Tricyclic antidepressants (TCAs), such as amitriptyline and clomipramine, are highly effective but may cause side effects like dry mouth and constipation.

Monoamine oxidase inhibitors (MAOIs), such as phenelzine, are less commonly used due to dietary restrictions and side effects.

3. Mood Stabilizers

Use: Mainly used to treat bipolar disorder (manic and depressive episodes).

Representative drugs:

Lithium: A classic medication that requires regular blood concentration monitoring.

Valproate sodium, carbamazepine: can also be used for epilepsy treatment.

4. Anti-anxiety medications (Anxiolytics)

Use: To relieve anxiety symptoms, such as generalized anxiety disorder, panic attacks.

classify ：

Benzodiazepines (BZDs), such as diazepam (Valium) and alprazolam, are fast-acting but highly addictive, making them unsuitable for long-term use.

Non-benzodiazepines: such as butorphanol and pregabalin, which are less addictive.

5. Sedative-Hypnotics

Use: To treat insomnia or relieve sleep disturbances for a short period of time.

Representative drugs:

Benzodiazepines: such as eszopiclone and triazolam.

Non-benzodiazepines: such as zolpidem and dexmedetomidine.

6. Stimulants

Indications: Primarily for Attention Deficit Hyperactivity Disorder (ADHD) and narcolepsy.

Common medications include methylphenidate (Ritalin) and amphetamine (Adderall).

7. Other drugs

NMDA receptor antagonists, such as ketamine, are used for rapid relief of treatment-resistant depression.

Anticholinergic agents: such as benztropine, used to relieve extrapyramidal reactions caused by antipsychotic drugs.

matters need attention ：

Individualized treatment: drug selection should be based on the specific symptoms, tolerance and comorbidity of the patient.

Side effect management: Regular follow-up visits to monitor drug side effects (such as weight gain, metabolic abnormalities).

Psychotherapy combination: Medication is usually combined with psychotherapy (such as cognitive behavioral therapy) for better results.

If you need more specific information about the drug or advice on the use of a particular disease, you can discuss it further!

**Q19.How long does it usually take for psychiatric drugs to take effect?**

The onset time of psychiatric medications varies depending on the type of medication, individual differences, and specific conditions. Below are the onset time ranges for some common psychiatric medications:

Antidepressants (e.g., SSRIs, SNRIs):

It usually takes 2-4 weeks to start to see results, and some patients may need 4-6 weeks to feel the obvious effect.

Full efficacy may take 6-8 weeks or longer.

Anti-anxiety drugs (e.g., benzodiazepines):

It works quickly, usually relieving acute anxiety symptoms within 30 minutes to an hour.

But such drugs are usually used for short-term treatment and long-term use can lead to dependence.

Antipsychotics (e.g., atypical antipsychotics):

Acute symptoms of schizophrenia or bipolar disorder may respond within a few days to 1-2 weeks.

Complete remission may take 4-6 weeks or longer.

Emotion stabilizers (e.g., lithium salts):

For bipolar disorder, it may take 1-2 weeks to start to work, and it may take weeks to months to fully stabilize mood.

Stimulants (e.g., methylphenidate for ADHD):

It usually takes 30 minutes to an hour to take effect, but long-acting preparations may take longer.

matters need attention ：

Individual differences: Metabolism, severity of disease and drug sensitivity vary from person to person, and the time of onset may vary.

Continue to take the medicine: even if you do not feel the effect in the short term, do not stop taking the medicine by yourself. Adjust the dosage or change the medicine as prescribed by the doctor.

Side effects: Some drugs may have side effects before they take effect, such as dizziness, nausea, etc., which are usually alleviated after adaptation.

If you have any questions about the effectiveness of the drug, it is recommended to keep in touch with your attending physician and review the effectiveness regularly.

**Q20. Will taking psychiatric drugs damage brain function?**

Whether psychiatric drugs can cause damage to brain function depends on a number of factors, including the type of drug, dosage, duration of use, individual differences, and adherence to medical advice. Here are some key points:

1. Types of drugs and their mechanisms of action

Antidepressants (e.g., SSRIs, SNRIs) typically improve mood by regulating neurotransmitters like serotonin and norepinephrine. While long-term use may enhance brain plasticity, excessive or inappropriate use can cause side effects such as emotional blunting.

Antipsychotics (e.g., typical and atypical antipsychotics) are primarily used to treat conditions such as schizophrenia. Some medications may affect the dopamine system, and long-term use could lead to movement disorders (e.g., tardive dyskinesia) or mild cognitive decline.

Sedatives (e.g., benzodiazepines): Long-term use may lead to dependence and cognitive decline (e.g., memory loss).

Emotion stabilizers (such as lithium) are effective for bipolar disorder, but blood levels should be monitored regularly to avoid toxicity.

2. Short-term and long-term effects

Short term: Medications are usually used to relieve acute symptoms, and side effects may include drowsiness, inattention, etc., but are mostly reversible.

Long-term: Some medications may have mild effects on brain structure or function, but the benefits of treatment usually outweigh the risks when weighed. For example, untreated mental illnesses (such as depression and schizophrenia) themselves can cause brain damage.

3. Individual differences

People respond very differently to drugs. Some people may be more sensitive to certain drugs, while others are more tolerant.

Age, underlying health conditions and genetic factors also play a role in how drugs affect the brain.

4. The importance of rational drug use

Follow the doctor's advice: take the medicine strictly according to the doctor's prescription and instructions, and avoid adjusting the dose or stopping the medicine by yourself.

Regular evaluation: Doctors usually regularly evaluate the efficacy and side effects of the drug and adjust the treatment plan if necessary.

Comprehensive treatment: combined with psychotherapy, lifestyle adjustment, etc., can reduce the dependence on drugs.

5. Research evidence

Most studies show that the rational use of psychiatric drugs has little negative effect on brain function and can effectively improve disease symptoms.

Some studies have suggested that long-term use of certain drugs may be associated with a slight decline in cognitive function, but these effects are usually mild and need to be weighed against the harm of the disease itself.

sum up

When used properly, psychiatric drugs usually do not cause significant damage to brain function, but may protect brain health by improving symptoms. The key is:

Choose the right medicine and dosage.

Regular monitoring and adjustment of treatment plans.

Combine with non-pharmacological treatment.

If in doubt, it is advisable to discuss the individual risks and benefits in detail with your attending physician.

**Q21. How to deal with the side effects of psychiatric drugs, such as weight gain and drowsiness?**

Managing side effects of psychiatric medications (such as weight gain and drowsiness) requires a comprehensive approach that includes medication adjustments, lifestyle modifications, and psychological support. Here are some practical recommendations:

1. Talk to your doctor

Medication adjustment: Some side effects may subside over time, but if they persist, consult your doctor about switching medications or adjusting dosages. For instance, certain antipsychotics like olanzapine can cause weight gain, whereas aripiprazole has a lesser metabolic impact.

Combination therapy: Doctors may add other medications to relieve side effects (such as metformin to control weight).

2. Manage weight

Dietary adjustments:

Reduce high sugar and high fat foods and increase protein and fiber intake.

Avoid overeating and eat regularly.

exercise habit ：

At least 150 minutes of moderate-intensity exercise (e.g., brisk walking, swimming) per week.

Combine strength training to increase your basal metabolic rate.

Monitor weight: Record weight changes regularly and detect problems in time.

3. Fight drowsiness

Adjust the time of taking the medicine: If the medicine causes drowsiness, try to take it before going to bed.

Regular sleep schedule: Keep a regular sleep schedule and avoid long naps during the day.

Moderate caffeine: A small amount of coffee or tea may help you stay alert, but avoid excessive amounts that interfere with your sleep at night.

4. Psychological support

Understanding side effects: Understand that side effects are part of treatment and avoid anxiety caused by weight or fatigue.

Seek support: Join a patient support group or talk to a counselor to reduce your stress.

5. Other considerations

Regular check-ups: monitor blood sugar, blood lipids and other indicators to prevent metabolic syndrome.

Avoid self-discontinuation: sudden discontinuation may aggravate the condition, so follow the doctor's instructions.

If side effects significantly impact your quality of life, consult your doctor promptly to discuss alternative options. Since reactions vary from person to person, personalized adjustments are crucial!

**Q22. Can I get addicted to psychiatric drugs?**

Whether psychiatric medications can lead to addiction depends on the type of medication, how it's used, and individual differences. Here are some key points to help you understand this issue:

1. Drug Classification

Drugs that may lead to addiction: Certain psychiatric drugs, such as benzodiazepines (e.g., diazepam, alprazolam) and some sedative-hypnotics, can lead to dependence or addiction with long-term or excessive use.

Low addiction potential: Most antidepressants (e.g., SSRIs, SNRIs) and antipsychotics (e.g., olanzapine, risperidone) are generally not addictive, but must be used strictly under medical supervision.

2. How to Use

Short-term use: Doctors usually prescribe short-term prescriptions based on the condition to reduce the risk of addiction.

Long-term use: Some medications may need to be taken long-term (such as antidepressants), but doctors will regularly evaluate, adjust the dose or change the medication to avoid dependence.

3. Individual differences

People's physical and psychological states are different, and their reactions to drugs are different. Some people may be more likely to develop dependence, while others may not.

4. The importance of following medical advice

Psychiatric drugs must be used under the guidance of a professional doctor. Do not increase or decrease the dosage or stop taking the drugs by yourself. Sudden withdrawal may lead to withdrawal reactions or relapse.

5. Alternative therapies

For patients concerned about addiction, doctors may recommend non-pharmaceutical therapies such as psychotherapy and lifestyle changes.

sum up

Not all psychiatric drugs are addictive. The key is to use them properly and follow the doctor's instructions. If you have any doubts, it is recommended to communicate with your doctor in detail to understand the risks and benefits of the drugs.

**Q23. What is a seizure-free electroconvulsive therapy?**

Modified Electroconvulsive Therapy (MECT) is an enhanced form of electroconvulsive therapy (ECT) primarily used to treat severe mental disorders such as depression, schizophrenia, and mania. Unlike traditional ECT, MECT employs anesthesia and muscle relaxants during treatment to prevent convulsions caused by electrical stimulation, thereby reducing treatment risks and side effects.

main features ：

1. No convulsion: Through anesthesia and muscle relaxants, patients will not have obvious convulsion reaction during the treatment.

2. High safety: Reduce the complications such as fracture and muscle strain caused by convulsion.

3. Significant efficacy: It has rapid and significant efficacy for refractory depression, schizophrenia and other diseases.

4. Wide range of application: It is suitable for patients who are ineffective or unable to tolerate the side effects of drugs.

therapeutic process ：

1. Anesthesia: The patient is under general anesthesia before treatment.

2. Muscle relaxation: Use muscle relaxants to prevent cramps.

3. Electrical stimulation: The brain is stimulated by precisely controlled currents to induce transient changes in brain electrical activity.

4. Recovery: The patient will gradually wake up after treatment and usually requires brief observation.

matters need attention ：

Treatment should be carried out in a professional medical institution, jointly operated by psychiatrists and anesthesiologists.

A full physical examination and evaluation is required before treatment.

Possible side effects include temporary memory impairment, headaches, etc., but are usually reversible.

MECT is a safe and effective treatment, particularly suitable for patients requiring urgent symptom relief.

**Q24. What are the main diseases treated by transcranial magnetic stimulation?**

Transcranial Magnetic Stimulation (TMS) is a non-invasive neuromodulation technique that modulates neuronal excitability by applying magnetic fields to the cerebral cortex. It is primarily indicated for the following conditions or symptoms:

1. Depression

Treatment-resistant depression: Transcranial Magnetic Stimulation (TMS) may serve as an alternative or adjunct therapy for patients unresponsive to pharmacotherapy.

Major depression: Particularly suitable for patients who cannot tolerate drug side effects or refuse electroconvulsive therapy (ECT).

2. Anxiety disorders

TMS can modulate brain regions associated with anxiety, including generalized anxiety disorder (GAD) and post-traumatic stress disorder (PTSD).

3. Schizophrenia

It is mainly used to improve negative symptoms (such as emotional apathy, social withdrawal) and some positive symptoms (such as auditory hallucinations).

4. Chronic pain

For conditions such as fibromyalgia and migraines, transcranial magnetic stimulation (TMS) can alleviate pain by modulating brain regions involved in pain perception, including the primary motor cortex.

5. Parkinson's disease

For the improvement of motor symptoms (such as tremor, rigidity) and non-motor symptoms (such as depression, cognitive impairment).

6. Neurorehabilitation

Rehabilitation after stroke: Promote motor function recovery.

Spinal cord injury: improve neural plasticity.

7. Obsessive-Compulsive Disorder (OCD)

For patients unresponsive to medication or behavioral therapy, TMS may help alleviate obsessive symptoms.

8. Tinnitus

Tinnitus symptoms were alleviated by regulating auditory cortex activity.

9. Other exploratory applications

Such as Alzheimer's disease, multiple sclerosis, addictive behaviors, etc., but more research is needed to support the efficacy.

matters need attention

TMS typically requires multiple sessions (e.g., once daily for 4-6 weeks).

Side effects are less common and may include headaches, scalp discomfort or temporary hearing sensitivity.

Not for patients with a history of epilepsy or intracranial metal implants.

The specific indications and efficacy of TMS may vary among individuals, and it is recommended to undergo evaluation and treatment under the guidance of a professional doctor.

**Q25. Can I stop taking psychiatric drugs by myself after symptoms improve?**

Whether to stop psychiatric drugs on your own after symptoms improve depends on the specific situation. Here are some key points:

1. The particularity of psychiatric drugs

Psychiatric drugs (such as antidepressants, anti-anxiety drugs, antipsychotics, etc.) usually need to be taken for a long time to maintain a stable therapeutic effect.

Suddenly stopping the drug may cause withdrawal reactions (such as dizziness, nausea, mood swings, etc.) or symptoms relapse.

2. Potential risks of stopping medication on your own

Relapse risk: Improvement of symptoms does not mean complete cure of the disease, and self-discontinuation of the drug may lead to recurrence.

Drug withdrawal: Some drugs need to be gradually reduced (such as antidepressants), and sudden withdrawal may cause discomfort.

Individual differences: Each person's condition and drug response are different, and doctors will adjust the medication plan according to the specific situation.

3. The right approach

Consult your doctor: When symptoms improve, talk to your doctor to see if you can reduce or stop the medication.

Gradual tapering: If you need to stop the drug, the doctor will usually make a plan to gradually reduce the amount to reduce withdrawal reactions and the risk of relapse.

Regular follow-up: even after stopping the drug, regular follow-up should be done to monitor the changes in the condition.

4. Exceptions

If the side effects of the drug are severe or allergic reactions occur, contact your doctor immediately, rather than stopping the drug on your own.

sum up

It is not recommended to discontinue psychiatric drugs by yourself. After the symptoms improve, you should communicate with your doctor to make a scientific plan to reduce or discontinue the drugs to ensure the safety and effectiveness of the treatment.

**Q26. How long should psychiatric drugs be taken after the condition is stable?**

Whether and how long psychiatric medications should be taken after the condition has stabilized depends on a number of factors, including the type and severity of the illness, history of relapse, and individual response to the medication. Here are some general guidelines:

1. Acute-phase treatment

After the symptoms are relieved, the drug should be taken for 4-6 months to consolidate the effect and prevent the recurrence of symptoms.

2. Maintenance therapy

For patients with a first episode and complete remission, doctors may recommend continuing the medication for 6-12 months, followed by a gradual reduction depending on the situation.

For multiple recurrences or chronic diseases (such as schizophrenia, bipolar disorder, etc.), long-term or even lifelong medication may be required to prevent recurrence.

3. Drug reduction or discontinuation

Drug reduction or discontinuation must be done under the guidance of a doctor, and the dosage should be reduced gradually to avoid a rebound or withdrawal reaction.

Close observation is still required after discontinuation, and some patients may need regular follow-up or re-medication.

4. Individualized adjustments

Doctors will adjust the medication plan according to the patient's condition stability, side effects of drugs, quality of life and other factors.

5. Psychological and social support

In addition to medication, psychotherapy, social support and a healthy lifestyle are important parts of maintaining stability.

Important reminder:

Do not stop taking the medicine or adjust the dosage without consulting your doctor.

Regular follow-up visits and communication with doctors to ensure the scientific and safe treatment plan.

If you or a family member are taking psychiatric medication, it is recommended to discuss the specific medication plan with your attending physician.

**Q27. Can mental illness be completely cured?**

The recovery from mental illness varies depending on the type of illness, individual differences, treatment methods, and support systems. Here are some key points:

1. Some mental illnesses can be completely cured

For example, some anxiety disorders, mild depression, post-traumatic stress disorder (PTSD), etc., may be completely recovered through psychotherapy, drug intervention or lifestyle adjustment, and no longer need continuous treatment.

2. Some mental diseases can be controlled but difficult to cure completely

For example, chronic mental diseases such as schizophrenia and bipolar disorder cannot be completely cured, but the symptoms can be effectively controlled through medication, psychotherapy and social support, and patients can live a relatively normal life.

3. Definition of cure

Clinical cure: symptoms disappear and function is restored, but long-term management may be required.

Complete cure: symptoms completely disappear and do not recur, which is rare in some diseases.

4. Key factors

Early intervention: The earlier the treatment, the better the effect.

Comprehensive treatment: a combination of medication, psychotherapy, social support and life adjustment.

Individual differences: Recovery varies from person to person.

5. Hope and Support

Even if not completely cured, many patients are able to significantly improve their quality of life through scientific treatment and support systems.

If you or someone close to you is experiencing mental health problems, it is recommended to seek professional help early and develop a personalized treatment plan.

**Q28. Will the mental illness relapse after rehabilitation?**

Whether a relapse occurs after mental illness recovery depends on a number of factors, including the type of illness, the individual's physical and psychological state, the treatment approach, the social support system, and lifestyle. Here are some key points:

1. Disease type

Recurrent diseases: such as depression, bipolar disorder, schizophrenia, etc., usually have a high risk of recurrence. For example, about 50% of patients with depression will relapse after recovery.

Single or situational disorders: such as certain anxiety disorders or post-traumatic stress disorder (PTSD), the risk of recurrence may be low if the triggers are eliminated and the treatment is thorough.

2. Treatment compliance

Continue treatment: Even if symptoms ease, doctors usually recommend continuing medication or psychotherapy for a while to reduce the risk of relapse. Stopping too early can lead to a rebound.

Psychological treatment: Psychological interventions such as cognitive behavioral therapy (CBT) can help patients establish coping mechanisms and reduce relapse.

3. Social support

Support from family, friends, and community is essential for recovery. A lack of support systems may increase the risk of relapse.

4. Lifestyle

A healthy lifestyle (such as regular sleep, a balanced diet, and moderate exercise) and avoiding stressors (such as excessive work or negative relationships) can help maintain mental health.

5. Individual differences

Recovery paths vary from person to person. Some may fully recover and never relapse, while others may require long-term management.

6. Early intervention

If signs of relapse (such as mood swings, sleep problems) are detected and addressed in time, the symptoms can be prevented from worsening.

sum up

Although relapse is possible after psychiatric rehabilitation, the risk can be significantly reduced through scientific treatment, good social support and healthy lifestyle. Patients and their families should maintain regular communication with doctors and develop a personalized long-term management plan.

**Q29. How to prevent the recurrence of mental illness?**

Preventing relapse is a comprehensive process involving medication, psychological support, lifestyle adjustment and social environment. Here are some key measures:

1. Stick to medication

Take medicine as prescribed: take the medicine prescribed by the doctor on time and in the right amount. Avoid stopping the medicine or adjusting the dose by yourself.

Regular follow-up: Keep in touch with your doctor to evaluate your condition and medication effects, and adjust your treatment plan in time.

Monitor side effects: Pay attention to the possible side effects of the drug and report to the doctor in time.

2. Psychotherapy and support

Psychological counseling or therapy: Regularly receive psychotherapy (such as cognitive behavioral therapy, supportive therapy, etc.) to help identify and cope with negative emotions or thought patterns.

Family support: The care and understanding of family and friends is crucial for recovery. You can attend family therapy or education courses.

Join a support group: Talk to others who have had similar experiences, share experiences, and reduce loneliness.

3. Healthy lifestyle

Regular sleep schedule: Keep a regular sleep schedule and avoid staying up late or getting too tired.

Eat a balanced diet: Eat nutrient-rich foods and avoid excessive caffeine, alcohol or high sugar foods.

Moderate exercise: Doing aerobic exercise regularly (such as walking, yoga, etc.) can help relieve stress and improve mood.

4. Stress Management

Identify stressors: Learn to identify stressors that may trigger relapse (such as work stress, interpersonal relationships, etc.).

Relaxation techniques: Practice deep breathing, meditation, or mindfulness exercises to help relieve anxiety and tension.

Time management: Arrange work and life reasonably to avoid overwork.

5. Avoid triggers

Avoid alcohol and drugs: These substances may worsen symptoms or interfere with the effect of the drug.

Reduce stimulation: Avoid excessive exposure to negative news or high-pressure environments.

6. Social support and vocational rehabilitation

Return to society: Gradually resume social activities and work, but avoid putting too much pressure on yourself.

Career counseling: If needed, seek vocational rehabilitation services to help readapt to the work environment.

7. Early warning and intervention

Recognize warning signs of relapse, such as mood swings, sleep problems, and social withdrawal, and seek medical attention.

Emergency plan: Work with your doctor or family to create an emergency plan that specifies what to do if it recurs.

8. Stay positive

Set small goals: Build confidence by accomplishing small goals.

Develop hobbies: Engage in activities you enjoy, shift your attention, and increase your happiness.

Preventing relapse requires the joint efforts of patients, families and medical teams. Since everyone's situation is different, it is recommended to develop a personalized prevention plan according to individual needs. If you have any questions or need further support, please consult a professional doctor or psychologist in time.

**Q30. Are mental illnesses inherited?**

Whether a mental illness is inherited to the next generation is a complex question, and the answer depends on the type of illness, genetic factors, environmental factors, and their interaction. Here are some key points:

1. Genetic factors

Some mental illnesses have a genetic predisposition: Studies of certain mental illnesses (such as schizophrenia, bipolar disorder, depression, autism spectrum disorder, etc.) suggest that genetic factors play an important role. If there is a family history of these diseases, the risk of offspring developing them may increase.

Polygenic inheritance: Most mental disorders are not determined by a single gene, but by the combined action of small variations in multiple genes (polygenic inheritance). The cumulative effect of these genes may increase the risk of disease, but does not necessarily mean that the disease will occur.

2. Environmental factors

Environmental triggers: Even with genetic predisposition, environmental factors (such as childhood trauma, stress, substance abuse, and lack of social support) often play a key role in triggering the disease. For example, a person may carry high-risk genes but may not develop the disease without environmental stress.

Epigenetics: Environmental factors can even influence gene expression through epigenetic mechanisms (such as DNA methylation), thereby indirectly affecting the occurrence of mental disorders.

3. Risk of specific diseases

Schizophrenia: If one parent is affected, the risk for children is about 10%; if both parents are affected, the risk can be as high as 40-50%.

Bipolar disorder: Children have a risk of about 5-10% when a first-degree relative (such as a parent or sibling) is ill.

Depression: Genetic risk is lower, about 30-40%, but environmental factors (such as stress or trauma) play a bigger role.

4. Prevention and intervention

Early screening: For people with family history, early symptoms can be monitored and timely intervention can be made.

Healthy lifestyle: Good psychological support, stress reduction, and avoidance of drug abuse can reduce the risk of onset.

Genetic counseling: If there is a family history of severe mental illness, genetic counseling can be considered to understand the specific risk.

sum up

Genetic predisposition to mental illness exists, but it's not absolute. It's the result of both genetic and environmental factors. Even with a genetic predisposition, adopting a healthy lifestyle and receiving psychological support can significantly reduce the risk of developing the condition. If you have specific concerns, it's advisable to consult a psychiatrist or genetic counselor.

**Q31. How can people with mental illness better reintegrate into society?**

Reintegration of people with mental illness into society is a complex but achievable process that requires multifaceted support and efforts. Here are some key steps and suggestions to help patients better reintegrate into society:

1. Professional treatment and rehabilitation

Continued treatment: Ensure that patients receive ongoing psychotherapy and medication (if needed) to stabilize the condition.

Rehabilitation plan: Develop a personalized rehabilitation plan, including psychological counseling, social skills training and vocational rehabilitation.

2. Social support system

Family support: Family members should show understanding, patience and companionship, and avoid discrimination or overprotection.

Friends and community: Encourage patients to stay in touch with friends, participate in community activities, and gradually rebuild their social network.

Support group: Join a patient support group to share experiences and reduce loneliness.

3. Career and social participation

Vocational training: Provide vocational training suitable for the patient's ability to help them re-enter the workforce or do volunteer work.

Workplace environment: Employers and colleagues should create an inclusive workplace and provide flexible work arrangements when necessary.

Social activities: Encourage patients to participate in interest groups, sports or art activities to enhance self-confidence.

4. Education and awareness

Public education: Reduce social misunderstanding and stigma of mental illness through publicity.

Patient education: Help patients understand their disease, learn to cope with stress and manage their emotions.

5. Policies and resources

Government support: Call on the government to provide more resources, such as community rehabilitation centers, employment assistance and medical security.

Legal protection: Ensuring the legal rights and interests of patients, such as anti-discrimination laws and privacy protection.

6. Self-acceptance and growth

Self-identity: Help patients accept their illness and recognize that recovery is a long-term process.

Set goals: Encourage patients to set small goals and achieve them step by step to enhance their sense of accomplishment.

7. Long-term attention and adjustment

Regular evaluation: Regularly evaluate the patient's rehabilitation progress and adjust the treatment and support plan in time.

Crisis intervention: Provide crisis intervention resources for patients and families to prevent relapse.

Through these measures, people with mental illness can gradually recover their social function and find the meaning of life and a sense of belonging again. Social inclusion and support are an indispensable part of this process.

**Q32. How to deal with the discrimination and comments of others against people with mental illness?**

In the face of discrimination and comments from others on people with mental illness, we can deal with it in the following aspects:

1. Stay calm and empathetic

Understanding others: Some people may have prejudices because they lack understanding of mental illness. Try to understand their ignorance, not confront it directly.

Avoid emotional reactions: They can escalate conflict. Stay calm and respond with facts and logic.

2. Education and science popularization

Share knowledge: Explain the nature of mental illness in simple, understandable terms, for example that it is a disease that needs to be treated like high blood pressure or diabetes.

Recommended resources: Recommended books, documentaries, or authoritative websites (such as the World Health Organization or content from professional medical institutions) to help others understand mental health issues.

3. Setting an example

Lead by example: Show respect and support for people with mental illness through your words and actions. For example, avoid using derogatory language in discussions (e.g., "crazy" or "mental").

Share stories: If appropriate, share stories of recovery or success for people with mental illness to break stereotypes.

4. Support patients

Listen and be there: If you're talking to the patient, give them emotional support and let them know they're not alone.

Seek help: Help patients seek professional help, such as counseling or medication.

5. Responding to malicious speech

Be clear: If someone's comments are malicious or offensive, be clear: "That's not appropriate. Mental illness requires understanding and respect."

Seek support: If discrimination occurs at school or work, report it to your supervisor, human resources department, or relevant agency.

6. Promoting social change

Participate in advocacy: Join or support mental health advocacy organizations to promote social awareness and tolerance of mental illness.

Voice: Share the right mental health knowledge on social media or in public to influence more people.

7. Self-care

Protect yourself: If you're a patient or caregiver, avoid getting too involved in arguments. Prioritize your own mental health.

Seek support: If you feel overwhelmed, talk to friends, family or professionals.

sum up

The key to combating discrimination and stigma lies in education, empathy, and action. By patiently explaining, setting positive examples, and supporting those affected, we can gradually shift societal biases. At the same time, it's crucial to safeguard our mental health and avoid letting negative emotions take hold.

**Q33. Can you fall in love with someone with a mental illness?**

It's possible to date someone with a mental illness, but it requires more understanding, patience, and support. Here are some key points to help you think and cope better with this situation:

1. Understanding and acceptance

Understanding the other person's illness: Different mental illnesses (such as depression, anxiety, bipolar disorder, schizophrenia, etc.) have different symptoms and effects. Knowing the other person's illness, treatment options, and possible challenges can help you better support them.

Accept the whole person: Mental illness is part of the person, but not all of them. Accepting their strengths and weaknesses, including their illness, is the foundation of a healthy relationship.

2. Communication and Support

Open communication: Encourage each other to share feelings and needs, while expressing your own feelings and boundaries. Open communication can reduce misunderstandings.

Provide support: Support the other person in receiving treatment (such as counseling, medication, etc.), but do not try to "cure" them. Your role is to accompany and support, not to replace professionals.

3. Self-care

Set boundaries: Caring for your mental health is just as important. If you're feeling overwhelmed or stressed, you may need to adjust your level of involvement or seek support.

Ask for help: If you feel confused or overwhelmed, seek help from a counselor or support group.

4. Relationship challenges

Emotional fluctuations: Mental illness can lead to emotional instability or behavioral changes that can strain relationships. You need to be mentally prepared and learn how to cope with these situations.

Long-term commitment: If the other person's mental illness requires long-term management, you need to consider whether you are ready for this long-term responsibility.

5. Love and Responsibility

Love is the foundation: If you truly love someone and are willing to be there for them through difficult times, the relationship can be very precious.

Responsibility and choice: But also be honest about whether you can handle the responsibility. If you feel overwhelmed, don't force yourself.

sum up

While dating someone with a mental illness is possible, it requires extra effort and understanding. The key lies in open communication, mutual support, and your genuine commitment to the relationship. If you choose to go on, remember to take care of yourself while offering support that's within your means.

**Q34. Can people with mental illness get married?**

In China, whether a person with a mental illness can get married mainly depends on whether their condition affects the legal validity of marriage and whether it complies with the relevant provisions of the Civil Code of the People's Republic of China. The key points are as follows:

1. Legal basis:

According to Article 1048 of the Civil Code, the circumstances that prohibit marriage include "direct blood relatives or collateral blood relatives within three generations" and "suffering from a medical condition that is not considered suitable for marriage".

For mental illness, if the patient is in the onset of the disease when he or she gets married, and the condition is so severe that he or she cannot recognize or control his or her own behavior (such as schizophrenia, severe depression, etc.), he or she may be deemed to have "medical reasons for not getting married", which may affect the legal validity of the marriage.

2. Marital validity:

If one party is unable to express their true will at the time of marriage due to mental illness (such as being coerced or unable to understand the meaning of marriage), the other party may apply for annulment of marriage.

If the illness worsens after marriage and the couple is unable to fulfill their obligations, the other party can file for divorce, but through legal procedures.

3. Practical recommendations:

Pre-marital medical examination: It is recommended that both parties undergo pre-marital examination to clarify the impact of illness on marriage.

Guardian consent: If the patient is in a mild condition or has stable treatment, the guardian or the patient may marry voluntarily.

Legal advice: It is recommended to consult a professional lawyer or civil affairs department to ensure the legality of the marriage.

In short, people with mental illness are not absolutely unable to marry, but it needs to be judged in combination with the severity of the illness, the treatment and the legal requirements.

**Q35. How to get along with family members with mental illness?**

Dealing with a family member who has a mental illness requires more patience, understanding, and skill. Here are some tips to help you better support them while taking care of your own emotions and mental health:

1. Understanding the disease

Learn about the condition: Learn about the characteristics, symptoms, and possible treatments for the mental illness your family member has. This can help you understand their behavior and reduce misunderstandings.

Avoid prejudice: Mental illness is not a "personality problem" or "weak willpower", but a health problem that requires professional intervention.

2. Stay patient and empathetic

Accept emotional fluctuations: Patients may have emotional instability or abnormal behavior. Try to keep calm and avoid blaming or criticizing.

Listen and be there: Even if their thoughts or words don't make sense, listen patiently and let them feel understood and supported.

3. Set realistic expectations

Avoid high expectations: Don't expect patients to "get better right away" or "be like they were before." Rehabilitation is a process that may require long-term treatment and adjustment.

Celebrate small progress: Focus on their positive changes and encourage them, even if it's a small one.

4. Establish healthy communication

Use "I" to express feelings: "I'm worried about you" instead of "you make me worried" to reduce the other person's defensive mentality.

Avoid arguments: If the other person is emotional, put the topic aside and talk when both parties are calm.

5. Encourage professional treatment

Accompanying medical care: If the other person wants to, they can accompany them to see a doctor or psychologist to help them better describe their symptoms.

Respect the choice: If the other person refuses treatment, gently express concern, but do not force it.

6. Take care of yourself

Set boundaries: While supporting your family, protect your own mental health and avoid over-sacrifice.

Seek support: Join a family support group, or talk to friends and a counselor to release stress.

7. Responding to crisis situations

Identify red flags: such as self-harm, suicidal tendencies, etc., and contact a professional organization or call the emergency number.

Make contingency plans: Know about nearby hospitals or crisis intervention resources in advance.

8. Create a supportive environment

Reduce stressors: Try to keep the family environment stable and quiet, and avoid too much stimulation or conflict.

Healthy habits: such as regular sleep, moderate exercise, and healthy eating are also good for mental health.

9. Avoid the "savior mentality"

You're not a doctor: Your role is to be a supporter, not a therapist. Let the professionals handle the professional stuff.

Accept limitations: Even if you give a lot, the illness may come back, and it's not your fault.

10. Long-term perspective

Recovery from mental illness is a marathon: It can take years or more, and it takes patience and hope.

Focus on the overall health of the family: Make sure the needs of other family members (including yourself) are met.

If you feel confused or helpless, contact your local mental health agency or counselor for more specific guidance. You're not alone-many families face similar challenges. Your care and support are already incredibly valuable to your loved ones.

**Q36. How to determine whether a family member has a relapse of mental illness?**

Determining whether a family member is experiencing a relapse of mental illness requires careful observation and scientific evaluation. Here are some key steps and precautions to help you identify possible signs of relapse:

1. Know the common symptoms of disease recurrence

The manifestations of relapse may vary among different mental disorders (e.g., depression, anxiety, schizophrenia, bipolar disorder, etc.). For example:

Depression: persistent low mood, loss of interest, changes in sleep or appetite, self-blame or suicidal thoughts.

Schizophrenia: hallucinations, delusions, disorganized speech, social withdrawal.

Bipolar disorder: extreme mood swings (manic or depressive), reduced need for sleep, impulsive behavior.

If you know the specific diagnosis of your family in the past, you can focus on the typical symptoms associated with the disease.

2. Observe behavioral changes

Emotional changes: sudden emotional fluctuations (such as irritability, anxiety, hyperexcitability or extreme sadness).

Social withdrawal: Reduce interaction with family or friends and avoid social activities.

Decline in daily functioning: inability to perform daily work, study, or household chores, and deterioration of personal hygiene.

Sleep and eating disorders: insomnia or drowsiness, sudden increase or decrease in appetite.

Speech or thought abnormalities: confused speech, difficulty expressing themselves, or unrealistic thoughts.

3. Compare past medical history

If the family has had a relapse, observe whether the current presentation is similar to the symptoms of the previous relapse.

Watch for new symptoms, which may indicate a change in the condition.

4. Listening and communication

Communicate with your family in a gentle, non-judgmental way and ask them how they feel and what they think.

Avoid direct questioning or criticism, and instead show care and support. For example: "I noticed you haven't been happy lately. Is there anything I can help you with?"

5. Record symptoms

Record the symptoms, frequency, and duration observed. This helps the doctor evaluate the condition.

Check for triggering factors (such as stressful events, medication changes, etc.).

6. Seek professional help

If you suspect a relapse, contact a psychiatrist or psychologist as soon as possible for evaluation.

If family members refuse medical care, try to contact community mental health services or crisis intervention hotlines.

7. Provide support

Be with your family and reduce their loneliness.

Help them stick to their treatment (such as taking their medication on time and returning to their doctor regularly).

Avoid overprotection or blame, and be patient and understanding.

8. Focus on your mental health

Caring for someone with a mental illness can be stressful for you, so remember to seek support (such as family, friends, and counseling).

If you're unsure whether your family member's symptoms indicate a relapse, it's advisable to consult a healthcare professional promptly. Early intervention can significantly improve the prognosis.

**Q37.What should you do if family members have hallucinations?**

Here are some steps you can take to help and support your family when they have hallucinations:

1. Stay calm and understanding

Don't panic: Auditory hallucinations can be caused by a variety of factors, such as mental health issues (such as schizophrenia), stress, medication side effects, or other physical illnesses. Keeping calm can help you cope better with the situation.

Show concern: Ask them how they feel in a gentle tone, and avoid denying or mocking their experience. For example: "I heard you heard some sounds. Can you tell me more?"

2. Assessing severity

Is it accompanied by other symptoms: If delusions are accompanied by delusions, emotional loss of control, abnormal behavior or a tendency to self-harm/harm others, seek professional help immediately.

Does it affect your daily life: If hallucinations make it difficult to live or work normally, seek medical attention as soon as possible.

3. Seek professional help

Psychiatrists or psychologists: Get in touch with a professional early for assessment and treatment. Doctors may recommend medication, psychotherapy, or other interventions.

Emergency: If delusions lead to dangerous behavior (such as self-harm or aggression), call emergency services or take the person to the hospital immediately.

4. Provide daily support

Companionship: Family members may feel lonely or afraid, and companionship can reduce their anxiety.

Reduce stress: Help create a quiet, stable environment and avoid excessive stimulation (such as noise or conflict).

Healthy habits: A regular schedule, a healthy diet and moderate exercise contribute to overall mental health.

5. Avoiding bad practices

Don't argue: Avoid denying their auditory hallucinations directly (e.g., "That sound doesn't exist"), which may make them feel misunderstood.

Don't ignore: Hallucinations can be a sign of a serious problem and should not be delayed.

6. Self-care

Take care of your emotions: Supporting a patient with hallucinations can be stressful or exhausting, so make sure you get enough rest and support.

7. Understand relevant knowledge

Learn about auditory hallucinations and mental health to better understand and cope with them.

If hallucinations are first or suddenly worse, it is recommended to seek medical attention as soon as possible to identify the cause and develop an appropriate treatment plan.

**Q38.What should we do if people with mental illness commit suicide?**

Suicidal behavior in people with mental illness is a very urgent and dangerous situation that requires immediate action. Here are the steps to take:

1. Stay calm and stay safe

Don't panic, try to keep calm and avoid irritating the patient.

If the patient is committing suicide (such as taking drugs, self-injury, etc.), stop and remove dangerous items (such as knives, drugs, etc.).

2. Seek professional help immediately

Call the emergency number (such as 120 or local emergency number) and explain the situation and request medical assistance.

Contact a psychiatrist or psychological crisis intervention hotline (such as China psychological assistance hotline: 010-82951332 or 800-810-1117).

3. Accompanying patients

Don't leave them alone. Stay with the patient and give emotional support.

Avoid lecturing or blaming, and show concern in gentle language, such as "I'm here for you" and "We can work together to find a solution."

4. Risk assessment

If the patient is willing to communicate, try to understand the seriousness of their suicidal intention (whether there is a plan, whether there is action).

But don't ask too many details, lest you aggravate their pain.

5. Provide support

Listen to the patient's feelings and do not interrupt or deny their emotions.

Express understanding, for example, "I know you're going through a tough time right now, and we can handle it together."

6. Follow-up

After the arrival of the first aid personnel, cooperate with the medical team to provide the patient's psychiatric history and current situation.

Ensure that patients receive professional treatment (such as hospitalization, psychological counseling or medication).

7. Preventing relapse

Pay attention to the emotional changes of patients for a long time and have regular follow-up visits.

Family members or caregivers can learn about mental health and identify warning signs (such as low mood or suicidal thoughts in speech).

Important reminder:

Don't ignore any suicide signals, even if the patient later claims "it was just a joke".

The confidentiality principle does not apply in emergency situations and should be informed to family or professionals when necessary.

If you are not sure how to deal with it, you can call the psychological assistance hotline or contact a professional organization for guidance. The safety of the patient's life is always the first.

**Q39. What should we do if a patient with mental illness shows violent tendencies?**

When a person with a mental illness shows violent tendencies, it is important to take appropriate measures to ensure the safety of the patient and others, as well as to respect the dignity and rights of the patient. Here are some suggestions:

1. Stay calm and stay safe

Don't irritate the patient: Avoid arguments or confrontation with the patient, keep a calm tone, and avoid sudden movements or loud speech.

Keep your distance: If the patient is agitated or aggressive, try to keep a safe distance from harm.

Remove hazardous materials: If possible, remove sharp objects or other objects that could be used as weapons from the patient's surroundings.

2. Assessment

Assess the severity: Observe whether the patient's behavior is a direct threat and whether it may harm himself or others.

Understanding triggers: If possible, try to understand what triggers the patient's violent behavior (such as hallucinations, delusions, side effects of drugs, etc.).

3. Seek professional help

Contact your doctor or psychiatrist: If the patient is being treated, contact their attending physician or psychiatric team immediately for professional guidance.

Call an emergency number: If the situation is urgent, the patient is unable to control his or her behavior, or has posed a threat to others, call the local emergency number (such as 110 or 120) immediately.

4. Comfort the patient

Listen and empathize: Try to listen to the patient's feelings, show understanding and concern, and avoid criticism or blame.

Provide support: Tell the patient that you are willing to help them and guide them to receive professional treatment.

5. Legal and social support

Understanding legal processes: In some cases, patients may be required to undergo treatment through legal processes (such as involuntary hospitalization under the Mental Health Act).

Reach out to community resources: Some areas have specialized mental health crisis intervention teams that can provide home assessment and support.

6. Long-term management

Continue treatment: Ensure that patients take their medicine on time and have regular follow-up visits to avoid worsening of the condition.

Family support: Family members can learn how to communicate with the patient and participate in the patient's rehabilitation plan.

7. Self-protection

Don't do it alone: If the patient's behavior is very dangerous, don't try to do it alone. Seek help.

Psychological support: Caregivers also need to pay attention to their own mental health and seek psychological counseling when necessary.

Important reminder:

Violent tendencies may be a sign of a worsening mental illness, or the result of medication side effects or other physical problems. Timely professional intervention is key. Don't ignore or delay.

If you have more specific situations to discuss, you can further describe, I will try to provide more targeted suggestions.

**Q40.How to persuade patients with mental illness to refuse to see a doctor?**

Persuading people with mental illness to seek medical help is a process that requires patience, understanding, and skill. Here are some tips to help you communicate more effectively with them:

1. Build trust

Listen and understand: First listen to the patient's concerns and feelings, do not rush to refute or deny. Let them feel respected and understood.

Show concern: Use gentle language to show your concern for them, for example, "I'm worried about you because I see you're not happy lately. I want to help you."

2. Addressing Misconceptions About Treatment

Explain the meaning of treatment: Explain the rationality of mental illness and treatment in simple and understandable language, for example: "Just as cold requires medicine, psychological problems also need professional help."

Overcoming fear: If patients are afraid of being labeled "mentally ill," emphasize that treatment is about helping them live better lives, not defining who they are.

3. Provide options

Respect autonomy: Avoid coercion and instead offer choices, such as: "We can first see a doctor, listen to their advice, and then decide whether to continue treatment."

Step by step guidance: You can start with simple psychological counseling or online consultation to reduce the patient's resistance.

4. Third-party support

Family or friend support: If the patient trusts a family or friend, ask them to help persuade.

Professional involvement: Sometimes doctors or counselors can communicate with patients by phone or home visits to reduce their resistance.

5. Highlighting the positive effects of treatment

Share success stories: You can mention examples of other people who have improved their lives through treatment, but avoid lecturing.

Focus on the future: Help patients imagine positive changes after treatment, such as: "If you feel better, we can do more of the things you like together."

6. Avoid negative emotions

Don't criticize or blame: Avoid saying things like "How can you be so stubborn?" which can increase the patient's psychological burden.

Be patient: If the patient refuses temporarily, try again after a while. Don't rush for success.

7. Emergency response

If a patient is at risk of self-harm or harming others, contact a professional or call emergency services immediately to ensure their safety.

Key points:

Patience and empathy are the most important.

Respect the patient's feelings and avoid coercion.

Guide gradually, starting with simple steps.

Seek professional help, if necessary, involving a doctor or psychologist.

If you have more specific scenarios or problems, I can tell you, I will provide you with more targeted advice.

**Q41. Should family members with mental illness be allowed to manage their money independently?**

Whether a family member with a mental illness should be allowed to manage money independently depends on the severity of the illness, cognitive ability, judgment, and stability of the illness. Here are some key factors to consider:

1. Severity of illness

Mild or stable: If a family member's mental illness symptoms are mild or stable and they are able to make rational decisions, they can try to manage their money independently, but with regular supervision and support.

Severe or acute phase: If the condition is severe (such as severe depression, acute onset of schizophrenia, etc.), it may not be able to make reasonable financial decisions, and even may cause economic losses or risks due to impulsive behavior. In this case, independent management is not recommended.

2. Cognitive and judgmental abilities

Assess whether the family has basic financial planning skills, such as budget management, debt control, and savings.

If their cognitive function is impaired (such as dementia, severe bipolar disorder, etc.), they may need assistance or complete management.

3. Support System

Is there a family member or professional (such as a social worker or financial advisor) who can provide regular supervision and support?

Consider a joint account or set a financial limit to balance independence and security.

4. Law and ethics

In some cases, it may be necessary to manage their finances through legal proceedings (such as guardianship or power of attorney) to ensure that their interests are not harmed.

Respect their autonomy and deal with it as little as possible.

5. Communication and trust

Communicate with family members to understand their wishes and needs.

If they are willing to accept help, they can be gradually guided to learn financial management skills.

propose ：

Try it in stages: Start with small amounts of money and see how well you manage it.

Professional assessment: Consult a psychiatrist or psychologist to assess their ability to manage independently.

Tools: Use budgeting software or auto-transfer features to reduce management complexity.

If the patient's condition is unstable or cognitive ability is significantly limited, it is recommended that a trusted family member or professional manage the patient while retaining some financial autonomy.

**Q42. What is the fastest way to find a person with a mental illness who has gone missing?**

The fastest way to find a person with a mental illness who has gone missing usually involves the following steps:

1. Call the police immediately

Contact local police: Provide the police with the patient's detailed information (such as name, age, physical characteristics, clothing, possible behavior, etc.) and explain the patient's mental state. The police can activate the emergency search mechanism, such as issuing a notice of assistance or using the surveillance system to track.

2. Mobilizing the strength of friends, family and community

Organize relatives and friends to search: go to the places where the patient often goes (such as parks, hospitals, supermarkets, etc.).

Use social media: Post a missing person notice on platforms such as WeChat, Weibo and Moments, with photos and contact information, to expand the reach.

3. Contact hospitals and relief stations

Notify nearby hospitals: Patients with mental illness may be sent to hospital due to an attack.

Contact the shelter: The patient may be taken in by the shelter.

4. Use professional search platforms

Posting on specialized platforms like "Baby Home" or "Blue Sky Rescue Team" enables rapid information dissemination.

5. Monitoring and transport hubs

Access surveillance: With police assistance, review surveillance footage near the patient's location.

Check stations, subway stations, etc.: Patients may try to leave by transportation.

6. Psychological intervention

Reassurance measures: After finding the patient, avoid irritating his emotions and contact a professional doctor for intervention.

matters need attention ：

Time is of the essence: the first 24 hours after a loss are the golden hours, and the faster you act, the better.

Stay calm: Family members need to stay calm and provide information to avoid missing key details.

If you need more specific help (such as how to write a missing person notice or contact local resources), you can communicate further!

**Q43. As a family member of a mental illness patient, how to relieve your own stress?**

As family members of people with mental illness, caring for your loved ones can be stressful. Here are some ways to ease your stress, which may help:

1. Accept reality and reduce self-blame

Understanding the illness: Recognizing that mental illness is a medical problem, not the fault of the patient or family. Accepting reality is the first step in reducing stress.

Avoid perfectionism: Don't expect too much from yourself and allow yourself to have imperfect moments.

2. Seek professional support

Psychological counseling: communicate with a psychological counselor or psychologist regularly to vent your inner stress and confusion.

Join a support group: Share experiences with other family members, support each other, and reduce loneliness.

3. Establish healthy self-care habits

Regular sleep: Get enough sleep and eat a healthy diet.

Exercise: Take a moderate amount of exercise every day, such as walking, yoga, etc., to help release stress.

Hobbies: Keep your hobbies and leave time for yourself to relax.

4. Set boundaries

Define your responsibilities: It's important to care for your patient, but it's also important to define your abilities and boundaries and avoid over-sacrifice.

Learn to say "no": Protect your mental health by saying no to extra responsibilities or requests when necessary.

5. Stay in touch with family and friends

Share your feelings: Don't isolate yourself. Share your feelings and stress with trusted friends and family.

Ask for help: Don't be afraid to ask others for help and share the responsibility of caring.

6. Learn about diseases

Understand the illness: By learning about mental illness, reduce the fear of the unknown and better cope with the patient's behavior.

Master coping skills: Learn how to communicate with patients and avoid conflict.

7. Practice mindfulness and relaxation techniques

Breathe deeply: When you feel stressed, try deep breathing or meditation.

Mindfulness practice: Focus on the present moment and reduce anxiety through mindfulness practice.

8. Give yourself a break

Take short breaks: Schedule short breaks, even if it's just a few minutes alone.

Short breaks: If possible, take occasional breaks from the caregiving environment and give yourself a break.

9. Focus on your emotions

Allow emotions to be released: When you feel angry, sad or helpless, allow yourself to express these emotions.

Keep a diary: Keep a diary to record your feelings and help you clarify your thoughts.

10. Seeking social resources

Use community services: Find out if there are local support programs or temporary care services for family members of people with mental illness.

Economic support: If you are under economic pressure, you can consult relevant institutions for subsidies or exemptions.

last

Caring for people with mental illness is a long road, but remember that your own mental health is just as important. You can't care for others well until you take care of yourself. If you feel overwhelmed, seek professional help.

If you need more specific advice or resources, let me know your specific situation and I'll do my best to help you.

**Q44. If children show signs of 'reduced speech, irritability, and aversion to learning', could this indicate psychological or mental health issues?**

Behavioral changes in children, such as "not talking, getting angry, and disliking school", may indeed indicate mental and psychological problems, but they may also be temporary manifestations caused by other factors. Here are some possible causes and suggestions for coping:

probable cause

Psychological problems:

Depression or anxiety: Children may be silent, irritable or refuse to go to school due to low mood or excessive worry.

Post-traumatic stress disorder (PTSD): Children who have experienced traumatic events (such as family breakdowns or school bullying) may exhibit similar behaviors.

Autism Spectrum Disorder (ASD): Some children with autism may experience communication difficulties and emotional challenges due to social challenges.

Attention Deficit Hyperactivity Disorder (ADHD): Irritability and school aversion may be symptoms of ADHD.

Environmental or stress factors:

Family problems: such as parental conflict, divorce, domestic violence, etc.

Academic pressure: excessive academic burden or learning difficulties may lead to aversion to learning.

Social problems: Being isolated or bullied by classmates may lead children to avoid social and school.

physiologic factor ：

Sleep deprivation or eating problems: Chronic sleep deprivation or malnutrition can affect mood and behavior.

Chronic diseases: such as thyroid dysfunction.

Signals to watch

Behavioral changes last longer (e.g., more than 2 weeks).

Along with other symptoms (such as changes in appetite, sleep problems, self-harm, excessive fear, etc.).

Complete loss of interest in daily activities.

Suggested response

Communicating with children:

Ask your child how they feel in a gentle, non-blaming way and see if there is a specific event that triggers these behaviors.

environment of observation ：

Check for stressors in the family, school, or social environment.

Seek professional help:

If you suspect a mental or psychological problem, it is recommended to consult a child psychologist or counselor as soon as possible.

School psychological teachers can also provide initial assessment and support.

Adjust your lifestyle:

Make sure your child has a regular schedule, a healthy diet and moderate exercise.

sum up

These behaviors may signal psychological issues, but they could also be a response to short-term stress. The key is to identify the root cause through patient communication and professional assessment, and provide appropriate support. If the situation persists or worsens, it's crucial to seek professional help.

**Q45. Can pregnant women or those trying to conceive take psychiatric drugs?**

Whether a pregnant woman or woman trying to conceive can take psychiatric drugs depends on the pros and cons of the situation and should be decided under the guidance of a professional doctor. Here are some key points:

1. Risk-reward trade-off

Necessity: If the mental illness of pregnant women (such as depression, anxiety, bipolar disorder, etc.) is not controlled, it may cause greater risks to the health of mother and child (such as premature birth, low birth weight, postpartum depression, etc.).

Drug risk: Some psychiatric drugs may have potential effects on fetal development (such as teratogenic risk, neonatal withdrawal symptoms, etc.), but not all drugs have the same risk.

2. Drug Classification and Safety

Antidepressants: Such as SSRIs (e.g., sertraline, fluoxetine) are generally considered relatively safe, though individualized assessment is required.

Antipsychotics: Some medications (e.g., olanzapine, quetiapine) may carry lower risks, but caution is advised.

Emotion stabilizers: such as lithium salt and valproic acid sodium are high risk during pregnancy and may cause fetal malformations, so they are usually not recommended.

Benzodiazepines (e.g., diazepam) may increase the risk of respiratory depression in newborns and should be avoided for long-term use.

3. Adjustments during pregnancy

If you plan to become pregnant, it is recommended to consult a psychiatrist in advance to assess whether medication needs to be adjusted or safer alternatives used.

Some medications may need to be tapered or discontinued, but avoid sudden discontinuation of medications that may lead to relapse.

4. Non-pharmaceutical interventions

Psychotherapy (e.g. cognitive behavioral therapy) can be used as an adjunct or alternative, particularly in mild to moderate symptoms.

5. Individualized decision-making

The doctor will make a personalized plan according to the specific condition of the pregnant woman, medication history, gestational age and other factors.

Regular prenatal check-ups and fetal monitoring (such as ultrasound) are necessary.

sum up

There is no absolute 'yes' or 'no' to taking psychiatric medications during pregnancy or preconception. The decision should be made under a doctor's guidance, weighing the risks and benefits for both mother and baby. Never adjust medications on your own—always consult with both a psychiatrist and an obstetrician.

**Q46. Can psychiatric drugs be taken with other drugs?**

Whether psychiatric drugs can be taken with other drugs depends on the specific combination of drugs and the individual condition of the patient. Here are some key points:

1. Drug interactions

Psychiatric drugs (such as antidepressants, antipsychotics, sedatives, etc.) may interact with certain drugs, resulting in enhanced, weakened or side effects.

for instance ：

The combination of SSRI antidepressants (e.g., fluoxetine) with certain painkillers (e.g., tramadol) may increase the risk of serotonin syndrome.

The combination of benzodiazepines (e.g., diazepam) and opioids may cause respiratory depression.

2. Individual differences

The patient's age, weight, liver and kidney function, metabolic capacity, etc., will affect the risk of drug interaction.

Some groups of people (such as the elderly or those with chronic diseases) are more susceptible to the effects of drug interactions.

3. Professional guidance

The combination of drugs must be adjusted under the guidance of a doctor or pharmacist.

Doctors will assess the risk based on the patient's medical history, medication record and drug characteristics, and adjust the dose or change the drug if necessary.

4. Common medications to watch for

Over-the-counter drugs: such as cold medicines and painkillers (containing acetaminophen or ibuprofen) may interact with psychiatric drugs.

Alcohol: The combination of psychiatric drugs may aggravate central nervous system depression.

Herbs or supplements: such as St. John's wort (which may reduce the effect of antidepressants) or melatonin (which may enhance sedation).

5. Recommendations

Do not adjust your medication on your own: even over-the-counter medications should be discussed with your doctor.

Keep a medication list: include all prescription, over-the-counter, and supplements for your doctor to evaluate.

Pay attention to side effects: such as dizziness, drowsiness, palpitations, etc., seek medical advice in time.

sum up

Psychiatric medications can be taken with other drugs, but must be strictly followed by a professional doctor to avoid potential risks of interaction. If in doubt, consult a doctor or pharmacist.

**Q47. Can I drink alcohol, coffee or strong tea while taking psychiatric drugs?**

Alcohol, coffee, or strong tea can have adverse effects on the effectiveness and body of psychiatric drugs. Here is a detailed analysis:

1. Drinking

Risk: Alcohol can interact with many psychiatric drugs (such as antidepressants, anti-anxiety drugs, sedatives, etc.), which may enhance the side effects of drugs (such as drowsiness, dizziness, respiratory depression, etc.), or even lead to severe poisoning reactions.

Recommendation: It is generally advised to avoid alcohol during medication, particularly when taking sedatives (e.g., benzodiazepines) or antidepressants (e.g., SSRIs, MAOIs).

2. Drink coffee or strong tea

Caffeine effects:

Excitatory effects: Caffeine may exacerbate the side effects of certain psychiatric drugs (such as anti-anxiety drugs), leading to anxiety, insomnia or palpitations.

Drug metabolism: Caffeine may affect the metabolism of certain drugs, thereby changing the effect.

propose ：

If the drug itself has a sedative effect (such as antipsychotic or anti-anxiety drugs), caffeine may counteract its effect.

Caffeine may worsen side effects (such as rapid heartbeat and elevated blood pressure) when taken with stimulant medications (e.g., ADHD drugs).

It is recommended to drink in moderation and observe your body's reaction. If you feel unwell, reduce or avoid drinking.

3. Individual differences

Everyone's constitution and drug reaction are different, so you should consult your doctor about whether you can drink alcohol or take caffeine, and adjust according to the contraindications in the drug instructions.

sum up

Avoid alcohol: especially when taking sedatives or antidepressants.

Be careful with caffeine: Watch your body's reaction and reduce or avoid it if necessary.

Follow the doctor's advice: always follow the doctor's specific advice.

**Q48. What are the dietary restrictions for patients with mental illness during medication?**

Dietary restrictions for people with mental illness vary depending on the type of medication and individual differences. Here are some common precautions and restrictions:

1. Alcoholic drinks

Contraindications: Alcohol may interact with psychotropic drugs (such as antidepressants, antipsychotics, sedatives, etc.), aggravating the side effects of drugs (such as drowsiness, dizziness, respiratory depression, etc.), and even leading to dangerous situations.

Suggestion: Avoid alcohol or alcoholic beverages during medication.

2. Caffeine (coffee, strong tea, energy drinks, etc.)

Why it's a no-no: Caffeine can make side effects of certain medications (like anti-anxiety or sedatives) worse, leading to anxiety, insomnia, or heart palpitations.

Suggestion: Reduce or avoid high caffeine foods and drinks.

3. High-tyramine foods (such as cheese, preserved foods, and fermented products)

Indications: Monoamine oxidase inhibitors (MAOIs), such as phenelzine and isocarbazepine.

Contraindication: The interaction between tyramine and MAOIs may trigger a hypertensive crisis (manifested as severe headache and sudden blood pressure spikes).

Suggestion: Avoid eating aged cheese, salted fish, sausage, soy sauce, beer and so on.

4. Grapefruit (juice)

Indications: Some antidepressants (e.g., fluoxetine, sertraline), antipsychotics (e.g., quetiapine), etc.

Contraindications: Grapefruit may interfere with drug metabolism, leading to increased blood drug concentration and increased risk of side effects.

Suggestion: Avoid grapefruit or grapefruit juice during medication.

5. High-fat diet

Indications: Certain antipsychotics (e.g., olanzapine, clozapine).

Contraindications: A high-fat diet may affect drug absorption or aggravate metabolic side effects (such as weight gain, dyslipidemia).

Suggestion: Maintain a balanced diet and avoid excessive intake of greasy food.

6. Tonic or herbal medicine

Contraindications: Certain herbs (such as St. John's wort) may interact with antidepressants, reducing their effectiveness or increasing side effects.

Tip: Consult your doctor before taking any supplements.

7. Other considerations

Regular diet: Avoid taking medication on an empty stomach (unless the doctor specifically states otherwise) to reduce gastrointestinal irritation.

Fluid replacement: Some medications may cause dry mouth or dehydration, so drink plenty of water.

Individual differences: Some patients may be sensitive to certain foods and need to adjust according to their own conditions.

Important Notice

The above contraindications are not applicable to all patients with mental illness, and should be adjusted according to the drugs taken and the guidance of the doctor.

If you feel unwell (such as dizziness, nausea, or allergies) while taking the medicine, seek medical attention immediately.

For more specific advice, please provide the name or type of medication the patient is taking, and I can help analyze it further!
