# Supplementary material for: Information quality, readability, and empathy of AI-generated public mental health information: a comparative evaluation of eight large language models
Source: Front Public Health. 2026 Jun 26;14:1859078. doi: 10.3389/fpubh.2026.1859078 (PMC13350173; doi:10.3389/fpubh.2026.1859078)
Supplement: Supplementary file 2 [file Supplementary_file_2.docx]

**Supplementary Table S1.** Model identifiers, access platforms, source status, and query information for the evaluated LLM chatbots

| **Model reported in manuscript** | **Official/public chatbot name** | **Access platform** | **Source status** | **Release or last-update information** | **Query route** | **Query date(s)** | **Query location** |
| --- | --- | --- | --- | --- | --- | --- | --- |
| GPT-5.2 | GPT-5.2 | ChatGPT official web interface | Proprietary / closed-source | Released 11 December 2025 | Official web interface | 1 February 2026 to 10 February 2026 | Lanzhou, China |
| GPT-5.2 Think | GPT-5.2 Thinking | ChatGPT official web interface | Proprietary / closed-source | Released as part of the GPT-5.2 family on 11 December 2025 | Official web interface | 1 February 2026 to 10 February 2026 | Lanzhou, China |
| Claude Sonnet 4.5 | Claude Sonnet 4.5 | Claude official web interface | Proprietary / closed-source | Released 29 September 2025 | Official web interface | 1 February 2026 to 10 February 2026 | Lanzhou, China |
| Claude Sonnet 4.5 Think | Claude Sonnet 4.5 (extended thinking enabled) | Claude official web interface | Proprietary / closed-source | Same underlying model release: 29 September 2025 | Official web interface | 1 February 2026 to 10 February 2026 | Lanzhou, China |
| Claude 4.5 Haiku | Claude Haiku 4.5 | Claude official web interface | Proprietary / closed-source | Released 15 October 2025 | Official web interface | 1 February 2026 to 10 February 2026 | Lanzhou, China |
| Gemini 3.0 Pro | Gemini 3 Pro (preview) | Gemini official web interface | Proprietary / closed-source | Released 18 November 2025 | Official web interface | 1 February 2026 to 10 February 2026 | Lanzhou, China |
| DeepSeek-R1 | DeepSeek-R1 | DeepSeek official web interface | Open-source / open-weight | Released 20 January 2025 | Official web interface | 1 February 2026 to 10 February 2026 | Lanzhou, China |
| DeepSeek-V3 | DeepSeek-V3 | DeepSeek official web interface | Open-source / open-weight | Introduced 26 December 2024; later updated as DeepSeek-V3-0324 on 25 March 2025 | Official web interface | 1 February 2026 to 10 February 2026 | Lanzhou, China |

All models were accessed through their official web interfaces in separate chat sessions. Memory/personalization functions were disabled when available. Web browsing, search, retrieval-augmented functions, and external tools were not enabled. “Think” modes refer to reasoning-enabled or extended-thinking configurations available in the corresponding chatbot interfaces. Release/update information was compiled from official release notes, changelogs, or product announcements.

**Supplementary Table S2.** Standardized questions and domain classifications of the 48 public mental health queries.

| **No.** | **Domain** | **Standardized question** |
| --- | --- | --- |
| 1 | Disease cognition | What is mental illness? |
| 2 | Disease cognition | What are the common types of mental illness? |
| 3 | Disease cognition | Is anxiety disorder a mental illness? |
| 4 | Disease cognition | Is depression a mental illness? |
| 5 | Disease cognition | What are the main factors that cause mental illness? |
| 6 | Disease cognition | Does insomnia or long-term staying up late increase the risk of mental illness? |
| 7 | Disease cognition | How can I tell whether I may have a mental illness? |
| 8 | Disease cognition | Can self-rating scales accurately determine whether someone has a mental illness? |
| 9 | Clinical diagnosis | Which department should I visit if I suspect that I have a mental illness? |
| 10 | Clinical diagnosis | How can mental illness be distinguished from other physical illnesses? |
| 11 | Clinical diagnosis | Why are multiple examinations needed when diagnosing mental illness in hospital? |
| 12 | Clinical diagnosis | Are psychological assessments helpful for diagnosing mental illness? |
| 13 | Clinical diagnosis | What should I do if different psychiatrists give different diagnoses? |
| 14 | Clinical diagnosis | Does visiting a psychiatric department mean that I will be considered to have a severe mental illness? |
| 15 | Treatment methods | What are the main treatments for mental illness? |
| 16 | Treatment methods | Which is more effective for mental illness, psychiatric medication or psychotherapy? |
| 17 | Treatment methods | Is exercise effective for mental illness? |
| 18 | Treatment methods | What types of medications are commonly used to treat mental illness? |
| 19 | Treatment methods | How long do psychiatric medications usually take to work? |
| 20 | Treatment methods | Can taking psychiatric medications damage brain function? |
| 21 | Treatment methods | How should side effects of psychiatric medications, such as weight gain and drowsiness, be managed? |
| 22 | Treatment methods | Are psychiatric medications addictive? |
| 23 | Treatment methods | What is modified electroconvulsive therapy? |
| 24 | Treatment methods | Which conditions are mainly treated with transcranial magnetic stimulation? |
| 25 | Rehabilitation management | Can I stop taking psychiatric medication by myself after my symptoms improve? |
| 26 | Rehabilitation management | How long should psychiatric medications be continued after the condition becomes stable? |
| 27 | Rehabilitation management | Can mental illness be completely cured? |
| 28 | Rehabilitation management | Can mental illness relapse after recovery? |
| 29 | Rehabilitation management | How can relapse of mental illness be prevented? |
| 30 | Rehabilitation management | Can mental illness be passed on genetically to the next generation? |
| 31 | Social and family | How can people with mental illness better reintegrate into society? |
| 32 | Social and family | How should discrimination and gossip about people with mental illness be handled? |
| 33 | Social and family | Can someone be in a romantic relationship with a person who has a mental illness? |
| 34 | Social and family | Can people with mental illness get married? |
| 35 | Social and family | How can I get along better with a family member who has a mental illness? |
| 36 | Social and family | How can family members tell whether a person with mental illness is relapsing? |
| 37 | Social and family | What should I do if a family member has auditory hallucinations? |
| 38 | Social and family | What should I do if a person with mental illness has suicidal behavior? |
| 39 | Social and family | What should I do if a person with mental illness shows violent tendencies? |
| 40 | Social and family | How should I persuade a person with mental illness who refuses to see a doctor? |
| 41 | Social and family | Should a family member with mental illness be allowed to manage money independently? |
| 42 | Social and family | What is the fastest way to find a person with mental illness who has gone missing? |
| 43 | Social and family | As a family member of a person with mental illness, how can I relieve my own stress? |
| 44 | Special issues | If a child becomes withdrawn, irritable, and unwilling to attend school, could this indicate a mental health problem? |
| 45 | Special issues | Can pregnant women or women preparing for pregnancy take psychiatric medications? |
| 46 | Special issues | Can psychiatric medications be taken together with other medications? |
| 47 | Special issues | Can alcohol, coffee, or strong tea be consumed while taking psychiatric medications? |
| 48 | Special issues | Are there any dietary restrictions for people with mental illness while taking psychiatric medications? |

The six domains were study-specific categories developed to summarize public mental health information needs.

**Supplementary Table S3.** Evaluation instruments and scoring metrics used in this study.

| **Domain** | **Instrument / metric** | **Assessment focus** | **Scoring approach / range** | **Interpretation / benchmark used in this study** |
| --- | --- | --- | --- | --- |
| Information quality | DISCERN | Quality, balance, and comprehensiveness of health information. | Total score: 16-75. | Higher scores indicate better quality. Categories: 63-75 excellent, 51-62 good, 39-50 average, 27-38 poor, and 16-26 very poor. |
| Information quality | Ensuring Quality Information for Patients (EQIP) | Quality and completeness of patient information. | Item-level scores are converted to a percentage: sum of scores / applicable items x 100. | Higher percentages indicate better patient information quality. Categories: 76%-100% high quality, 51%-75% good, 26%-50% fair, and 0%-25% poor. |
| Source transparency | Journal of the American Medical Association (JAMA) benchmark criteria | Transparency of online health information, including authorship, attribution, disclosure, and currency. | Four criteria scored 0 or 1; total score: 0-4. | Higher scores indicate greater source transparency. In this study, the score reflected spontaneous source transparency without explicit reference prompts. |
| Information quality | Global Quality Scale (GQS) | Overall quality and usefulness of health information. | Global score: 1-5. | Higher scores indicate better overall quality and usefulness. |
| Readability | Automated Readability Index (ARI) | Estimated reading grade level based on characters, words, and sentences. | Grade-level score. | Lower scores indicate easier text. A score <6 was considered consistent with the sixth-grade readability benchmark. |
| Readability | Flesch Reading Ease Score (FRES) | Ease of reading based on sentence length and syllable count. | Score: 0-100. | Higher scores indicate easier text. A score of 80-90 was considered consistent with the sixth-grade readability benchmark. |
| Readability | Gunning Fog Index (GFI) | Estimated years of formal education needed to understand the text. | Grade-level score. | Lower scores indicate easier text. A score <6 was considered consistent with the sixth-grade readability benchmark. |
| Readability | Flesch-Kincaid Grade Level (FKGL) | Estimated U.S. school grade level required to understand the text. | Grade-level score. | Lower scores indicate easier text. A score <6 was considered consistent with the sixth-grade readability benchmark. |
| Readability | Coleman-Liau Index (CL) | Estimated readability based on letters and sentences. | Grade-level score. | Lower scores indicate easier text. A score <6 was considered consistent with the sixth-grade readability benchmark. |
| Readability | Simple Measure of Gobbledygook (SMOG) | Estimated years of education required to understand the text based on polysyllabic words. | Grade-level score. | Lower scores indicate easier text. A score <6 was considered consistent with the sixth-grade readability benchmark. |
| Empathy | Five-point Likert empathy rating | Perceived empathic quality of each response. | Score: 1-5, where 1 = not empathetic and 5 = very empathetic. | Higher scores indicate stronger perceived empathy. Scores of 4 or 5 were classified as high-empathy responses. |

Abbreviations: ARI, Automated Readability Index; CL, Coleman-Liau Index; EQIP, Ensuring Quality Information for Patients; FKGL, Flesch-Kincaid Grade Level; FRES, Flesch Reading Ease Score; GFI, Gunning Fog Index; GQS, Global Quality Scale; JAMA, Journal of the American Medical Association; LLM, large language model; SMOG, Simple Measure of Gobbledygook. This table includes only the instruments and metrics used in the present manuscript.
